# Assembly of Complex 1,4‐Cycloheptadienes by (4+3) Cycloaddition of Rhodium(II) and Gold(I) Non‐Acceptor Carbenes

**DOI:** 10.1002/anie.202012092

**Published:** 2020-11-24

**Authors:** Helena Armengol‐Relats, Mauro Mato, Antonio M. Echavarren

**Affiliations:** ^1^ Institute of Chemical Research of Catalonia (ICIQ) Barcelona Institute of Science and Technology Av. Països Catalans 16 43007 Tarragona Spain; ^2^ Departament de Química Analítica i Química Orgànica Universitat Rovira i Virgili C/ Marcel⋅lí Domingo s/n 43007 Tarragona Spain

**Keywords:** cycloadditions, cycloheptadienes, decarbenation reactions, enyne cycloisomerizations, metal carbenes

## Abstract

The formal (4+3) cycloaddition of 1,3‐dienes with Rh(II) and Au(I) non‐acceptor vinyl carbenes, generated from vinylcycloheptatrienes or alkoxyenynes, respectively, leads to 1,4‐cycloheptadienes featuring complex and diverse substitution patterns, including natural dyctiopterene C′ and a hydroxylated derivative of carota‐1,4‐diene. A complete mechanistic picture is presented, in which Au(I) and Rh(II) non‐acceptor vinyl carbenes were shown to undergo a vinylcyclopropanation/Cope rearrangement or a direct (4+3) cycloaddition that takes place in a non‐concerted manner.

## Graphical abstract

Metal vinyl carbenes generated by retro‐Buchner reaction or an enyne cycloisomerization cascade react with 1,3‐dienes to afford complex 1,4‐cycloheptadienes in a formal (4+3) cycloaddition process. A combination of experiments and computations provides a full mechanistic picture in which two pathways, a vinylcyclopropanation/Cope rearrangement or a non‐concerted (4+3) cycloaddition, can compete depending on the substitution pattern of the substrate.

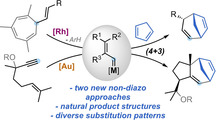

## Introduction

**Scheme 1:**
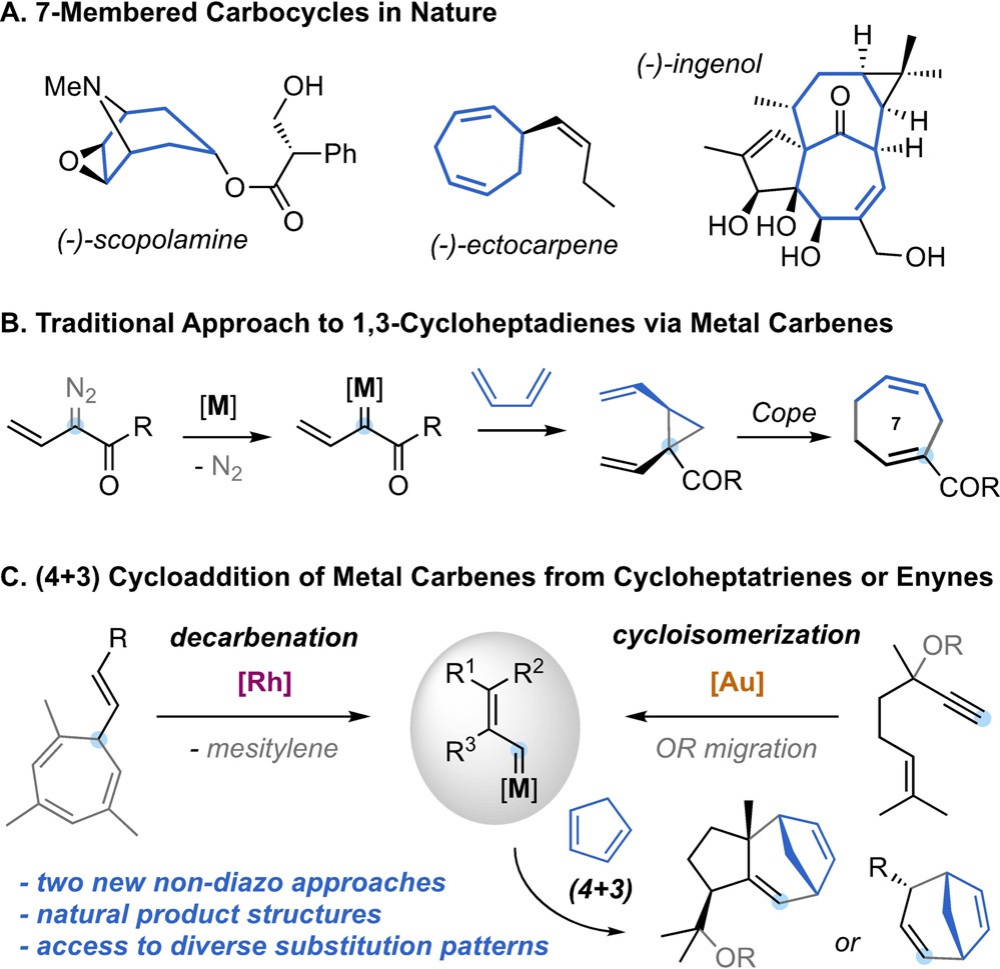
A. Examples of bioactive natural products containing 7‐membered carbocycles. B. Classical approach for the assembly of 1,4‐cycloheptadienes by diazo‐carbene chemistry. C. This work: formal (4+3) cycloaddition of dienes with non‐acceptor vinyl carbenes generated from cycloheptatrienes or alkoxyenynes.

7‐Membered carbocycles are important motifs present in a variety of natural products (Scheme 1, A).^[1]^ However, they can be challenging to synthesize through traditional cyclization pathways.[^1^, ^2^] The synthesis of these medium‐sized rings often relies on the use of cycloaddition strategies,^[3]^ ring expansions,^[4]^ ring‐closing metathesis,^[5]^ or cross‐coupling reactions.^[6]^ In particular, the divinylcyclopropane‐cycloheptadiene Cope rearrangement has been established as a versatile alternative for the construction of 1,4‐cycloheptadienes.^[7]^ The main challenge of this approach is the stereoselective assembly of the key *cis*‐divinylcyclopropane with the appropriate substitution pattern. In this regard, Davies and co‐workers pioneered the reaction of 1,3‐dienes with donor‐acceptor vinyl carbenes, generated from diazo compounds, to give cycloheptadienes after cyclopropanation and Cope rearrangement, through an overall formal (4+3) cycloaddition process (Scheme 1, B).^[8]^ This strategy was successfully applied by several groups for the synthesis of 7‐membered carbocycles,^[9]^ but it displays the inherent problems associated to diazo compounds,^[10]^ limiting the methodologies to the use of acceptor metal carbenes.^[11]^ Different gold(I)‐catalyzed (4+3) cycloadditions were developed over the years, mainly based on the reaction of 1,3‐dienes with allenes^[12]^ or vinyl carbenes generated from propargyl esters,^[13]^ cyclopropenes^[14]^ or by recombination of linear dienediynes.^[15]^ In our pursuit to identify new sources of non‐acceptor metal carbenes, we have established both 7‐vinyl‐1,3,5‐cycloheptatrienes (**1**) and 5‐alkoxy‐1,6‐enynes (**6**) as suitable precursors. The former can undergo metal‐catalyzed retro‐Buchner reactions (releasing aromatic units),^[16]^ and the latter can take part in a gold(I)‐catalyzed cycloisomerization/migration cascade sequence^[17]^ generating non‐acceptor vinyl carbene intermediates with diverse substitution patterns (Scheme 1, C). Here, we report the reactivity of these catalytically‐generated intermediates with 1,3‐dienes to give a diverse range of 1,4‐cycloheptadienes through a (4+3) formal cycloaddition process. We also present a complete mechanistic picture in which two main pathways for the cycloaddition process were studied both experimentally and theoretically. Our theoretical and experimental study shows that, depending on the substitution pattern of the substrates, a direct closing of a cationic intermediate is sometimes favored rather than the well‐stablished cyclopropanation‐Cope rearrangement pathway.

## Results and Discussion

We found that the reaction of **1 a** with 1,3‐cyclohexadiene in the presence of Rh_2_TFA_4_ at 40 °C gives cleanly the product of (4+3) cycloaddition **3 a** in excellent yield, as a single diastereoisomer (Table 1, entry 1). Notably, with this system, no traces of dinvinylcyclopropane **3 a′** were observed after 18 h. The same reaction can be carried out using gold(I) catalyst **A** with bulky JohnPhos ligand although with lower yield (Table 1, entry 5). In contrast, other Lewis acids, or even less electrophilic Rh(II) complexes, failed to give significant amounts of product **3 a** (Table 1, entries 6–8). Furthermore, the reaction could be carried out in a significantly wide range of temperatures, or without protective inert atmosphere, showing only a moderate drop in yield (Table 1, entries 2–4).

**Table 1:** Optimization and control experiments.^[a]^ 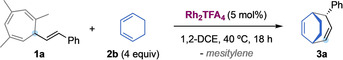

Entry	Deviations from standard conditions	Yield **3 a**
1	none	91 %
2	under air and non‐dry solvent	75 %
3	25 °C instead of 40 °C	82 %
4	80 °C instead of 40 °C	65 %
5	[Au] instead of Rh_2_TFA_4_	70 %
6	ZnBr_2_ (20 mol %) instead of Rh_2_TFA_4_	8 %
7	InCl_3_ (20 mol %) instead of Rh_2_TFA_4_	n/d
8	Rh_2_(esp)_4_ instead of Rh_2_TFA_4_	7 %

[a] Standard conditions: Reaction of **1 a** with **2 b** (4 equiv), in the presence of Rh_2_TFA_4_ (5 mol %), in dry 1,2‐dichloroethane (0.15 M), under Ar, at 40 °C, 18 h. [Au]=**A**=[(JohnPhos)Au(MeCN)]SbF_6_; n/d=not detected.

**Scheme 2:**
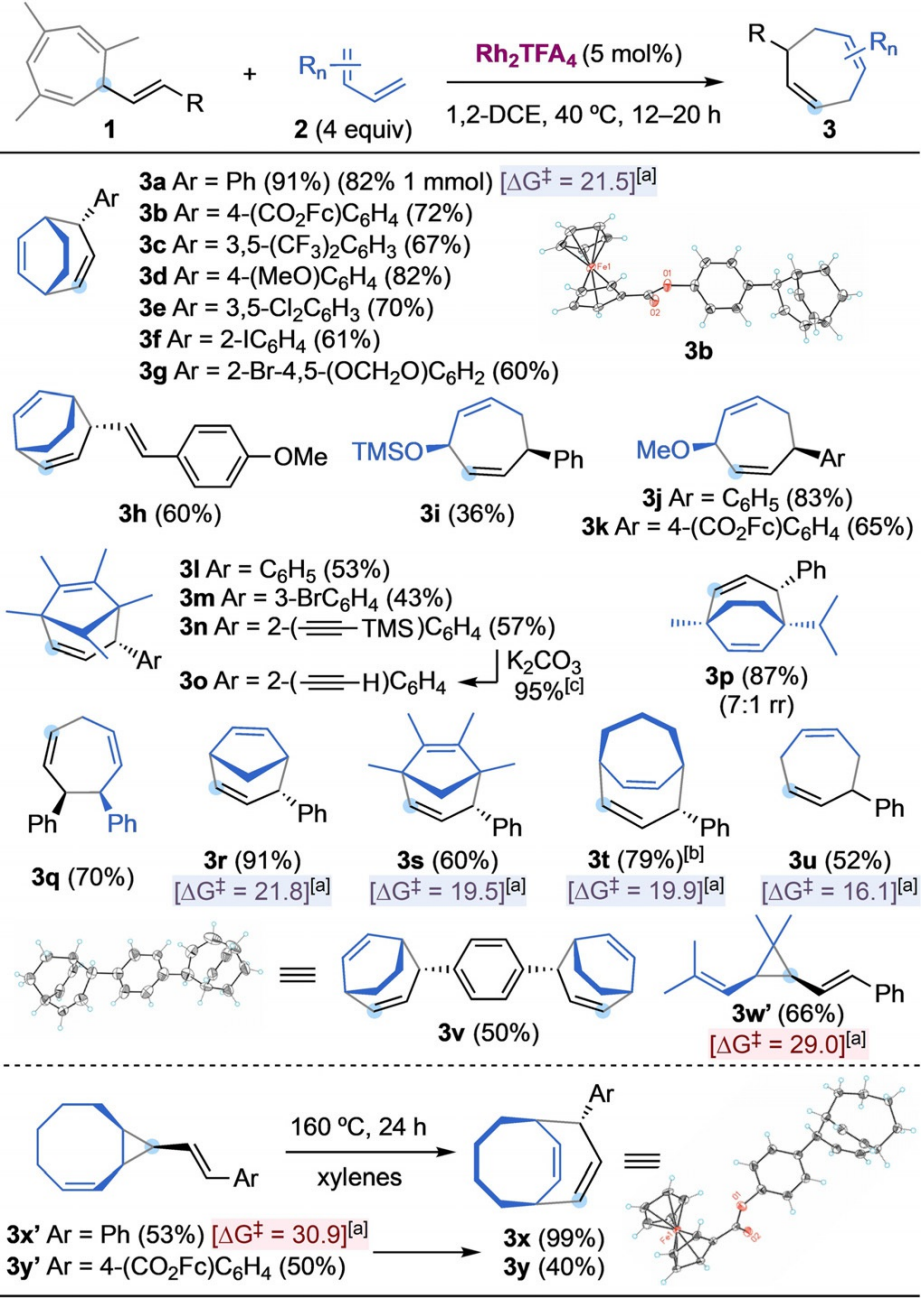
Reaction scope of the decarbenation/(4+3) cycloaddition sequence. [a] Activation energy (kcal mol^−1^ at 25 °C) calculated for the free Cope rearrangement of the corresponding dinvinylcyclopropanes **3′**. [b] At 60 °C for 20 h. [c] K_2_CO_3_ (2 equiv) in MeOH/DCM at 25 °C, 3 h. Fc=ferrocenyl.

A range of styryl cycloheptatrienes **1** were successfully employed, showing the tolerance to both electron‐rich and electron‐poor aromatic rings on the carbene fragment (**3 a**–**g**) (Scheme 2). Different aryl halides (**3 e**–**g**, **3 m**) and a dienyl carbene (**3 h**) could be transferred, and even a TMS‐alkyne was tolerated (**3 n**), which could be easily deprotected afterwards (**3 o**). A double decarbenation/(4+3) cycloaddition was also carried out giving directly **3 v** in 50 % yield as a single diastereoisomer, forging six new stereocenters in one step. The relative configuration of the bicyclic products was inequivocally confirmed by *x*‐ray analysis of **3 v** and ferrocene‐derivative **3 b**.^[18]^ Then we explored the scope of 1,3‐dienes. Non‐cyclic dienes could also be used (**3 q**), including different 1‐oxo‐1,3‐dienes which led to products **3 i**–**k**.^[19]^ The reaction of **1 a** with α‐terpinene afforded **3 p** in high yield and 7:1 ratio of regioisomers, corresponding to a more favorable cyclopropanation of the less‐hindered double bond, followed by a diastereospecific Cope rearrangement. Cyclic dienes of several sizes (5–7) were successfully employed in the (4+3) cycloaddition (**3 l**–**u**). On the other hand, we observed that the reaction of **1 a** with 1,3‐cyclooctadiene at 40 °C afforded only cyclopropane **3 x′**. A similar result was obtained when using 1,1,4,4‐tetramethylbutadiene (**3 w′**). The barrier of the free Cope rearrangement was calculated for several substrates leading to a simple predictive model: products of (4+3) cycloaddition were observed when the calculated barriers were lower that 22 kcal mol^−1^ (Scheme 2, Δ*G* ^≠^ in blue boxes), whereas cyclopropanes were isolated for higher barriers (red boxes).^[20]^ Thus, barriers of 29.0 and 30.9 kcal mol^−1^ were calculated for **3 w′** and **3 x′**, respectively. The mechanistic hypothesis was confirmed by obtaining 1,4‐cycloheptadienes **3 x** and **3 y** ^[18]^ by heating **3 x′** and **3 y′** up to 160 °C for 24 h.

**Scheme 3:**
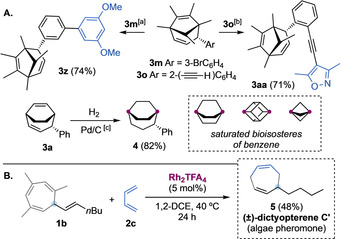
A. Diversification and reactivity of cycloheptadienes **3**. [a] 3,5‐Dimethoxyphenyl boronic acid, Cs_2_CO_3_, PdCl_2_/RuPhos (cat), dioxane/water (5:1), 80 °C, 2 h. [b] 4‐Iodo‐3,5‐dimethylisoxazole, Pd(PPh_3_)_2_Cl_2_ (cat), CuI (cat), triethylamine, 50 °C, 20 h. [c] H_2_ (1 atm, balloon), Pd/C (cat), ethanol, 25 °C, 18 h. B. Total synthesis of (±)‐dictyopterene C′.

Other cycloheptadienes **3 z** and **3 aa** were easily obtained from **3 m** and **3 o**, respectively, by cross‐coupling reactions (Scheme 3, A). It was possible to fully hydrogenate **3 a** under mild conditions, obtaining 2‐phenylbicyclo[3.2.2]nonane (**4**), which resembles an alternative saturated benzene bioisostere.^[21]^ Furthermore, to illustrate the potential of the methodology, starting from **1 b** and 1,3‐butadiene, we prepared in a single reaction flask dictyopterene C′ (**5**) (Scheme 3, B), a natural pheromone isolated from brown algae.^[22]^

Vinylcarbenes arising from the gold(I)‐catalyzed isomerization 5‐alkoxy‐1,6‐enynes **6** also undergo similar (4+3) cycloadditions (Table 2). Hydroazulenes **7** were obtained in one step, in a stereoselective manner, bearing the common core of the daucene family of natural products.[^23^, ^24^] We found that the selectivity between the formation of cycloheptadiene **7** or cyclopropane **8** is dependant on the sterics and electronics of the catalyst.^[25]^ Catalyst **A** provided the highest selectivity towards the formation of cyclopropane **8 a** (Table 2, entry 1: 1:2 **7 a**/**8 a**), while bulkier **B** gave a 1:1 ratio of **7 a**/**8 a** (Table 2, entry 2). A less donating phosphite ligand in catalyst **D** led to selective formation of cycloheptadiene **7 a** (Table 2, entry 4: 15:1 **7 a**/**8 a**). The same effect can also be observed in the different ratio of products obtained with catalysts **E** and **F**, where more σ‐donating triethylphosphine favored cyclopropanation product **8 a** in contrast with triphenylphosphine (Table 2, entries 5 and 6). Longer reaction times translated into lower yields, indicating decomposition, while the ratio of products was maintained (Table 2, compare entries 1 and 7). Finally, in the presence of Rh_2_TFA_4_ only unreacted 1,6‐enyne **6 a** was recovered (Table 2, entry 8).

**Table 2:** Catalyst screening for the gold(I)‐catalyzed reaction of 1,6‐enyne **6 a** with cyclopentadiene (**2 a**).^[a]^ 
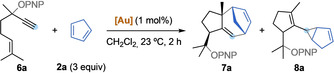

Entry	[Au]	Conversion	Yield **7 a**	Yield **8 a**
1^[b]^	A	100 %	26 % (7:1)	51 % (12:1)
2	B	100 %	40 % (5.7:1)	36 %
3	C	100 %	36 % (17:1)	12 %
4	D	100 %	60 % (5:1)	4 %
5^[c]^	E	100 %	40 % (4.7:1)	19 %
6^[c]^	F	91 %	27 % (4.4:1)	31 %
7^[d]^	A	100 %	18 % (8:1)	30 %
8^[d,e]^	[Rh] (5 mol %)	0 %	–	–

[a] Standard conditions: Reaction of **6 a** with **2 a** (3 equiv), in the presence of [Au] catalyst (1 mol %), in CH_2_Cl_2_ (0.2 M), at 23 °C, 2 h; NMR yields using tricholoroethene as internal standard, *d.r*. in parenthesis. [b] Isolated yields (as a mixture of **7 a** and **8 a**). [c] Addition of 1 mol % of NaBAr^F^_4_. [d] 24 h reaction time. [e] Reaction performed at 40 °C. PNP=*p*‐nitrophenyl, [Rh]=Rh_2_TFA_4_. 
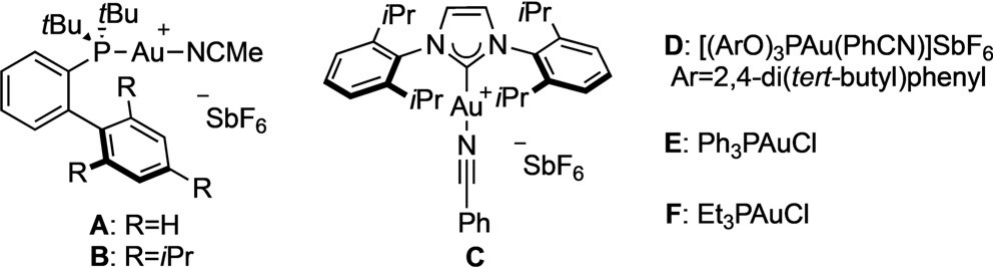

**Scheme 4:**
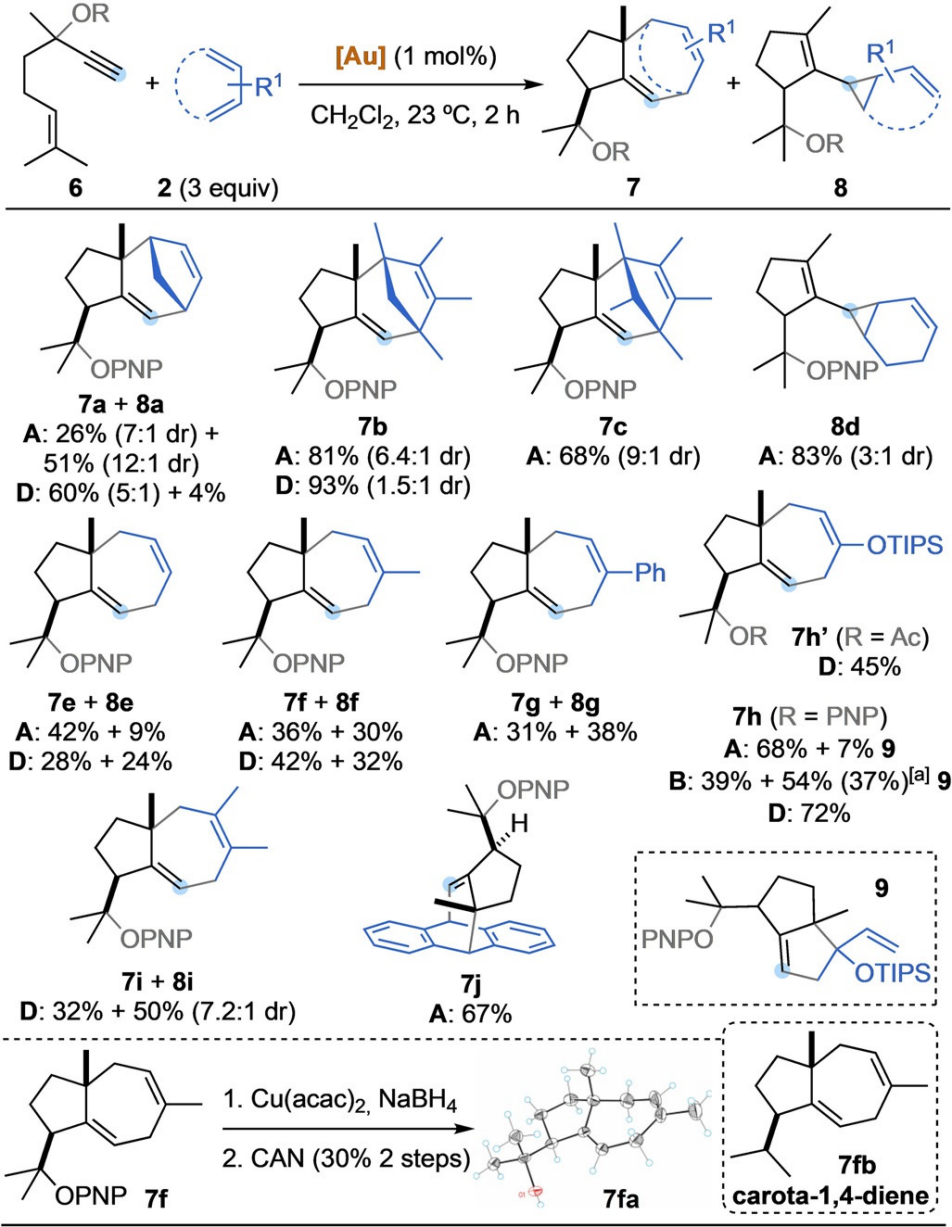
Reaction scope of the cycloisomerization/OR‐migration/(4+3) cycloaddition. Yields of both **7** and **8** are indicated for each substrate, unless only one of the two products (depicted) was obtained. [a] In parenthesis, isolated yield of **9** after treatment with HCl (cat) in EtOH for 24 h and purification. CAN = cerium ammonium nitrate.

We investigated the reaction scope using complexes **A** and **D** (Scheme 4). Tetramethyl and pentamethyl cyclopentadiene afforded exclusively (4+3)‐cycloaddition products **7 b** and **7 c**, respectively, with both catalysts. Interestingly, while the yield was slightly higher with phosphite complex **D**, JohnPhos catalyst **A** gave higher diastereoselectivity. In contrast, 1,3‐cyclohexadiene only afforded cyclopropane product **8 d**.^[26]^ Acyclic dienes also reacted in moderate to good yields. For 1,3‐butadiene, gold(I) complex **A** was more selective for the formation **7 e** than catalyst **D**, that essentially gave a 1:1 mixture of **7 e** and **8 e**. Introducing a methyl substituent at the 2‐position (isoprene) led to a drop in selectivity, still giving **7 f** as the main product with both catalysts. Exchanging the methyl by a phenyl (**8 g**), or adding a second methyl group (**8 i**) inverted the selectivity, giving cylopropanes as major products. Reaction with an electronically biased 2‐oxy‐1,3‐diene delivered **7 h** and **9** as a 10:1 mixture using catalyst **A**. Catalyst **D** afforded only (4+3) product **7 h** and bulkier complex **B** led to a 1:1.4 ratio of **7 h** and **9**, which is the product of formal (3+2) cycloaddition. Replacing the PNP migrating group by an acetate in the 1,6‐enyne led to a drop of yield, but maintaining the same tendency in selectivity observed before, obtaining only **7 h′**.^[27]^ Finally, antracene was found to be a good reaction partner for the (4+3) cycloaddition, giving **7 j** in 68 % yield as a single isomer. The PNP group in compound **7 f** was removed, delivering **7 fa** as a crystalline solid in 30 % yield over two steps. This allowed the confirmation of the relative configuration of the (4+3) product by *x*‐ray diffraction.^[18]^ Compound **7 fa** is a hydroxylated analogue of carota‐1,4‐diene (**7 fb**), a natural product isolated from *Rosa rugosa* leaves.^[28]^

**Scheme 5:**
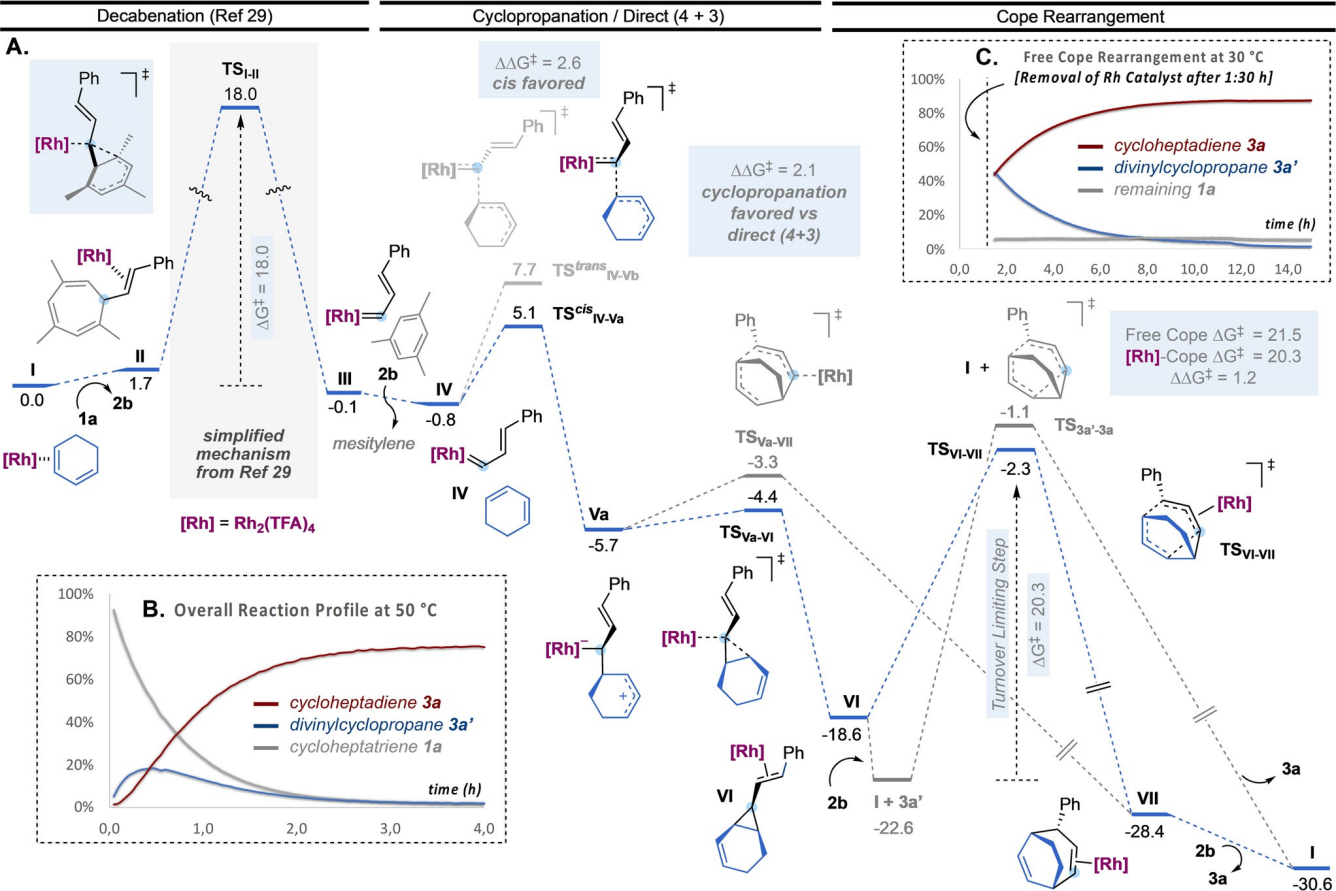
Overall mechanistic proposal for the decarbenation/(4+3) sequence based on both theoretical and kinetic observations. A. Free‐energy profile for the Rh(II)‐catalyzed (4+3) formal cycloaddition by decarbenation of **1 a** and reaction with **2 a** (kcal mol^−1^ at 25 °C). B. Overall kinetic profile of the reaction in TCE‐*d_2_* at 50 °C, followed by ^1^H NMR showing accumulation of intermediate **3 a′**. C. Kinetic profile of the free Cope rearrangement (after removing the Rh(II) catalyst) of **3 a′** to give **3 a** in CDCl_3_ at 30 °C followed by ^1^H NMR.

We studied the mechanism of the two systems by DFT calculations at 6‐311G++(d,p)(H, C, N, O, F, P) + LANL2TZ(Au, Rh)// B3LYP‐D3/6‐31G(d,p)(H, C, N, O, F, P) + LANL2DZ(Au, Rh) level of theory, taking into account the solvent effect (SMD=1,2‐dichloroethane or dichloromethane). For the first class of substrates, the process starts by the rhodium(II)‐catalyzed retro‐Buchner reaction of cycloheptatriene **1 a** (decarbenation) to give styryl carbene **III** releasing mesitylene (Scheme 5, A).^[29]^ An activation barrier of 18.0 kcal mol^−1^ was calculated from **I**, which was identified as local minimum. After exchanging mesitylene for 1,3‐cyclohexadiene (**IV**), the most favorable pathway corresponds to a stepwise cyclopropanation, in which the formation of *cis* open intermediate **Va** is kinetically more favored than the *trans* by 2.6 kcal mol^−1^, which would account for a perfect *cis* selectivity (80:1).^[30]^ The resulting open intermediate **Va** can evolve through two pathways: an almost barrierless cyclopropanation (**TS_Va‐VI_**) giving Rh(II)‐coordinated *cis*‐divinylcyclopropane **VI** or a direct (4+3) closing (**TS_Va‐VII_**), which affords Rh(II)‐coordinated final product **3 a** (**VII**). Although both pathways are energetically feasible, the cyclopropanation TS is lower in energy (ΔΔ*G* ^≠^=2.1 kcal mol^−1^). The resulting cyclopropane can further evolve through Cope rearrangement. Rh(II)‐coordinated divinylcyclopropane **VI** is in downhill equilibrium with free divinylcyclopropane **3 a′** + **I** (1,3‐cyclohexadiene η^2^‐coordinated to Rh_2_TFA_4_). This results in an activation barrier of 20.3 kcal mol^−1^ (larger, but rather similar than that for the retro‐Buchner step) for the Rh(II)‐coordinated Cope rearrangement (**TS_VI‐VII_**), which makes it the turnover‐limiting step of the entire process. The calculated energies are in agreement with the overall kinetic profile of the reaction (Scheme 5, B): following the reaction by ^1^H NMR, we observed an accumulation of intermediate cyclopropane **3 a′**. Alternatively, after decoordination from Rh(II), free cyclopropane **3 a′** can undergo a thermal Cope rearrangement (**TS_3a′‐3 a_**), with an only slightly higher energy barrier (ΔΔ*G* ^≠^=1.2 kcal mol^−1^). In order to prove the existence of the catalyst‐free pathway, we removed the Rh(II) catalyst after 1.5 h of reaction and then followed the evolution of the resulting mixture (Scheme 5, C). This showed a clean thermal conversion of intermediate divinylcyclopropane **3 a′** into 1,4‐cycloheptadiene **3 a** during 12 h at 30 °C. The successful removal of the Rh(II) catalyst was confirmed by the recovery of unreacted cycloheptatriene **1 a**. All in all, the entire mechanistic analysis is in agreement with the experimental observations, and explains why these vinyl Rh(II) carbenes generated from cycloheptatrienes lead cleanly to (4+3) cycloaddition products.

**Scheme 6:**
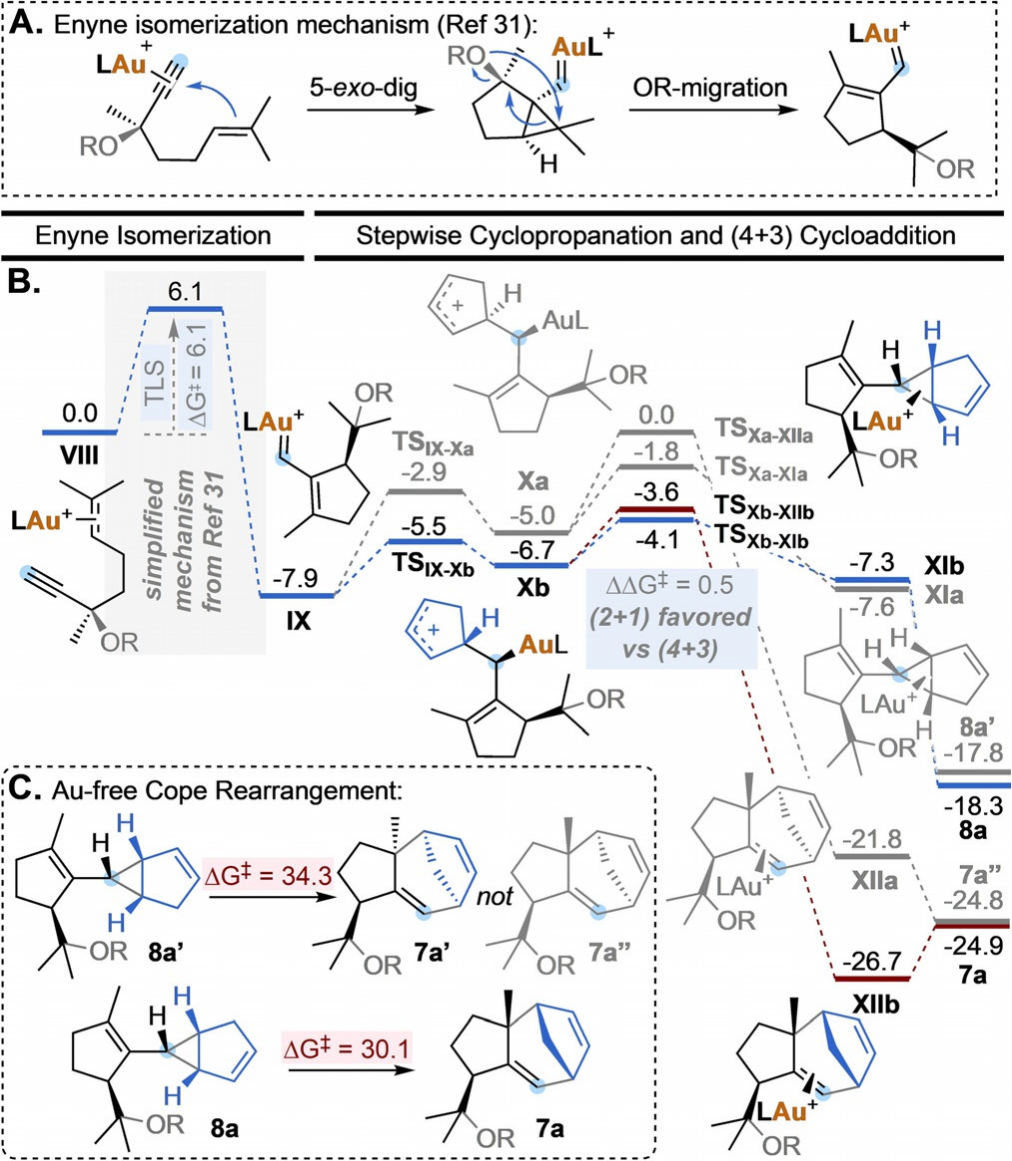
A. Enyne isomerization mechanism. B. Free‐energy profile for the Au(I)‐catalyzed cycloisomerization/OR‐migration/(2+1) and (4+3) cycloaddition cascade reaction of **6 a** with **2 a** (kcal mol^−1^ at 25 °C). L=JohnPhos, R=*p*‐nitrophenyl. C. Calculated energy barriers for the Au‐free Cope rearrangement of **8 a′** and **8 a** (kcal mol^−1^ at 25 °C).

A different scenario was found for the Au(I)‐catalyzed transformation of enyne **6 a** (Scheme 6). The first steps consist in an enyne cycloisomerization cascade involving a 5‐*exo*‐dig cyclization, followed by a 1,5‐migration of the OR group to form vinyl carbene **IX** (Scheme 6, A).^[31]^ This sequence contains the turnover limiting Step, with a overall energy barrier of 6.1 kcal mol^−1^ from local minimum **VIII**. Next, the intermolecular trapping of intermediate **IX** by cyclopentadiene (**2 a**) proceeds stepwise (Scheme 6, B).^[32]^ The two possible orientations of cyclopentadiene approaching carbene **IX** afford diastereomeric allyl carbocations **Xa** and **Xb** through **TS_IX‐Xa_** and **TS_IX‐Xb_**, respectively. These two TS differ in 2.6 kcal mol^−1^, which is consistent with the high diastereomeric ratio found experimentally in the final products.

Intermediates **Xa** and **Xb** can then evolve to deliver (2+1) or (4+3) cycloaddition products. Starting from most populated intermediate **Xb**, the formation of the cyclopropane ring in **XIb** through **TS_Xb‐XIb_** is favored by 0.5 kcal mol^−1^ over the 7‐membered ring‐closing towards **XIIb** through **TS_Xb‐XIIb_**. This translates into a 2.5:1 calculated ratio of (2+1)/(4+3) cycloaddition products **8 a** and **7 a** after decomplexation, in excellent agreement with the experimental results for this same system using catalyst **A** (2:1 **8 a**/**7 a** ratio, Table 2). Although the gold(I)‐catalyzed interconversion of **8 a** to **7 a** is energetically feasible and thermodynamically favored according to the calculated values (Δ*G* ^≠^=14.7 kcal mol^−1^, Δ*G*°=−6.6 kcal mol^−1^), we did not observe such transformation to take place experimentally.^[33]^ Finally, we calculated the energy barriers of the thermal Cope rearrangement (Scheme 6, B). Divinylcyclopropane **8 a** would have to overcome 30.1 kcal mol^−1^ to rearrange into **7 a**, while **8 a′** can only be converted into **7 a′** (and not **7 a′′**, which has the relative configuration observed experimentally) by a stereospecific Cope rearrangement, also with a high‐energy barrier of 34.3 kcal mol^−1^. These results indicate that, in contrast to that observed with products **3** (Scheme 2), densely substituted (4+3) cycloaddition products **7**, formed in the Au(I)‐catalyzed stepwise process, cannot be obtained by metal‐catalyzed nor thermal Cope rearrangement of the corresponding divinylcyclopropanes.

## Conclusion

To sum up, we have developed two new approaches for the synthesis of a wide variety of structurally complex 1,4‐cycloheptadienes. Both reactions rely on the formal (4+3) cycloaddition of non‐acceptor vinyl metal carbenes with 1,3‐dienes. The first approach involves the rhodium(II)‐catalyzed decarbenation or retro‐Buchner reaction of 7‐vinyl cycloheptatrienes, releasing a molecule of mesitylene. In the second approach, these intermediates are generated from 5‐alkoxy‐1,6‐enynes by a gold(I)‐catalyzed cyclization/migration cascade. We showed the potential of these two complementary methodologies in the rapid construction of molecular complexity, and in the total synthesis of natural compounds, such as dictyopterene C′. Furthermore, we present a complete mechanistic picture for both reactions, backed up by experiments and computations. We found that both a classical vinylcyclopropanation/Cope rearrangement sequence or a direct formal (4+3) cycloaddition are feasible, and the preference for each pathway depends on the substitution pattern of the substrates and intermediates.

## Conflict of interest

The authors declare no conflict of interest.

## Supporting information

As a service to our authors and readers, this journal provides supporting information supplied by the authors. Such materials are peer reviewed and may be re‐organized for online delivery, but are not copy‐edited or typeset. Technical support issues arising from supporting information (other than missing files) should be addressed to the authors.

Supplementary
