# Supplementary material for: Assembly of Complex 1,4‐Cycloheptadienes by (4+3) Cycloaddition of Rhodium(II) and Gold(I) Non‐Acceptor Carbenes
Source: Angew Chem Int Ed Engl. 2020 Nov 24;60(4):1916–22. doi: 10.1002/anie.202012092 (PMC7894532; doi:10.1002/anie.202012092)
Supplement: Supplementary file 1 — Supplementary [file ANIE-60-1916-s001.pdf]

Angewandte Chemie
Eine Zeitschrift der Gesellschaft Deutscher Chemiker

Supporting Information

Assembly of Complex 1,4-Cycloheptadienes by (4 + 3) Cycloaddition of Rhodium(II) and Gold(I) Non-Acceptor Carbenes

*Helena Armengol-Relats+, Mauro Mato+, and Antonio M. Echavarren**

anie_202012092_sm_miscellaneous_information.pdf

Table of Contents

1. General Considerations 2

2. Decagram-Scale Preparation of the Julia-Kocienski Reagent for the Synthesis of Vinyl Cycloheptatrienes 3

3. List and Preparation of 1,3-Dienes, 7-Alkenyl Cycloheptatrienes and Enynes 7

4. General Procedure A for the Synthesis of Second Generation Cycloheptatrienes via Julia-Kocienski Olefination 8

4.2 Characterization Data for the Different Styryl Trimethylcycloheptatrienes 9

5. Selected Optimization and Control Experiments 15

6. General Procedure B: Rhodium(II)-Catalyzed Decarbenation–(4+3) Cycloaddition 19

6.1 Characterization Data for the Different Cycloheptadienes (3) and Cyclopropanes (3') 20

7. General Procedure C: Gold(I)-Catalyzed Cycloisomerization–Migration–(4+3) Cycloaddition 33

7.1. Characterization Data for the Different Cycloheptadienes (7) and Cyclopropanes (8) 34

8. Mechanistic Studies on the Rh(II)-Catalyzed Decarbenation–(4+3) Cycloaddition 42

8.1 Kinetic Experiments 42

8.2 DFT Calculations 43

9. Mechanistic Studies on the Au(I)-Catalyzed Cycloisomerization–Migration–(4+3) Cycloaddition 49

9.1 Experiments 49

9.2 DFT Calculations 51

10. Synthesis of Natural Products and Diversification 56

11. X-Ray Crystal Data and Structure Refinement 62

12. NMR Spectra 66

13. DFT Energies and Coordinates 130

14. References 191

1. General Considerations

Unless otherwise stated, all of the reactions reported herein were carried out under argon atmosphere in solvents dried by passing through an activated alumina column on a PureSolv^{TM} Solvent Purification System (SPS, Innovative Technologies, Inc., MA). Anhydrous 1,2-dichloroethane (DCE) was purchased from Acros Organics and stored in an argon-filled glovebox. Yields refer to chromatographically and spectroscopically pure (^{1}H NMR) homogeneous material, unless otherwise stated. Thin layer chromatography was carried out using TLC aluminum sheets coated with 0.2 mm of silica gel (Merck Gf234) using short-wave UV light as visualizing agent and phosphomolybdic acid, $KMnO_4$ or acidic vanillin followed by heat as developing agents. Chromatographic purifications were carried out using flash grade silica gel (SDS Chromatogel 60 ACC, 40-60 µm) as the stationary phase manually, or using a CombiFlash®R_f instrument with normal phase disposable columns of different sizes (Teledyne Isco). Preparative TLC was performed on 20 cm x 20 cm silica gel plates (2.0 mm thick, catalogue number 02015, Analtech or 1.0 mm thick, catalogue number P02013 Analtech). NMR spectra were recorded at 23 ºC on a Bruker Avance 300, 400 Ultrashield or Bruker Avance 500 Ultrashield apparatus. Chemical shifts are reported in parts per million (ppm) downfield from tetramethylsilane, using the residual undeuterated solvent ($CHCl_3$ at 7.28 ppm ^{1}H NMR, 77.00 ppm ^{13}C NMR) or tetramethylsilane as reference. Coupling constants are reported in hertz (*Hz*). The following abbreviations were used to explain multiplicities: s = singlet, d = doublet, t = triplet, q = quartet, quint = quintuplet, m = multiplet, br = broad. Mass spectra were recorded on a Waters LCT Premier Spectrometer (ESI and APCI) or on a Autoflex Bruker Daltonics (MALDI and LDI), or a GC instrument (Agilent Network GC System) coupled with a MS (Agilent Technologies, inert XL MSD). FT-IR spectra (ATR) were recorded on a Bruker ALPHA System. Melting points were determined using a MP70 Melting Point System (Mettler Toledo). Unless otherwise stated, all reagents were purchased from commercial sources and used without further purification. Rhodium(II) trifluoroacetate dimer (min. 95% purity) was purchased from STREM Chemicals (45-1960) and used as received. For reproducibility reasons, it was stored in an Argon filled glovebox and all rhodium(II)-catalyzed reactions were set-up inside the glovebox under inert atmosphere. However, most of them are not significantly sensitive to air and can be carried out without a protective inert atmosphere, using rhodium(II) complexes stored in a desiccator.

2. Decagram-Scale Preparation of the Julia-Kocienski Reagent for the Synthesis of Vinyl Cycloheptatrienes

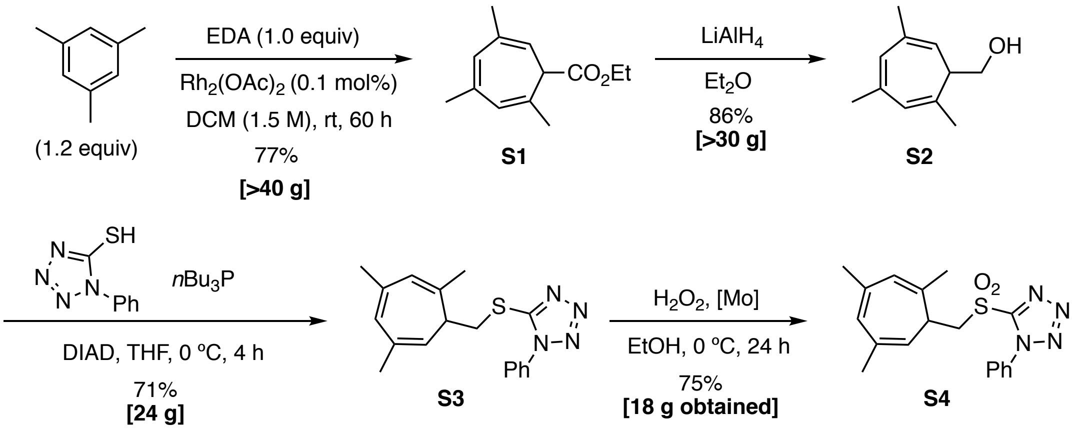

Ethyl 2,4,6-trimethylcyclohepta-2,4,6-triene-1-carboxylate (S1)

A dry two-necked 1 L round-bottomed flask equipped with a Teflon-coated magnetic stirring bar was charged with $Rh_2(OAc)_4$ (140 mg, 0.316 mmol, 0.10 mol%) and then evacuated and refilled with argon three times. Mesitylene (53 mL, 0.379 mol, 1.2 equiv) was added via syringe, dissolved in dichloromethane (211 mL, 1.5 M), and the mixture was degassed by bubbling argon through, while stirring, for 30-60 min. Ethyl 2-diazoacetate (40 mL, 0.316 mol, 1.0 equiv) was then added via automatic syringe to the stirring solution over 60 hours (*ca.* 0.5 mL/h) at room-temperature, before dichloromethane was removed in vacuum. Crude product was filtered through a big plug of silica gel, which was eluted with cyclohexane/EtOAc 9:1, until no more product came out. The solvent was removed in a rotatory evaporator and then, the product was dried in high vacuum overnight in order to remove excess mesitylene, leaving ethyl 2,4,6-trimethylcyclohepta-2,4,6-triene-1-carboxylate **S1** (50.1 g, 0.243 mol, 77% average yield of two runs at this scale) with enough purity for the next step (>90%).

^{1}H NMR (500 MHz, $CDCl_3$) δ 6.25 (s, 1H), 5.84 (s, 1H), 5.38 (d, J = 6.5 Hz, 1H), 4.29-4.19 (m, 2H), 2.86 (d, J = 6.5 Hz, 1H), 2.01 (s, 3H), 1.93 (d, J = 1.2 Hz, 3H), 1.90 (t, J = 1.1 Hz, 3H), 1.30 (t, J = 7.1 Hz, 3H) ppm.

^{13}C NMR (126 MHz, $CDCl_3$) δ 172.8, 138.1, 133.6, 129.6, 128.5, 125.3, 114.3, 60.6, 48.2, 24.3, 21.5, 20.3, 14.3 ppm.

HRMS (ESI Positive): calculated for $C_{14}H_{19}O_2$ $[M-H]^+$: 219.1380; found: 219.1379.

Complementary considerations

- Long addition times (0.5 mL/h) are required to get the maximum yield reported, which is especially desirable while working at large scale, but faster addition times can be used if required, in exchange of compromising the yields (around 50% at 2 mL/h). Also, the reaction can be run at smaller scales, keeping good yields while using the same 0.5 mL/h rate.

- Catalyst loading was evaluated down to 0.10 mol% without decrease of the yield, but this does not necessarily imply a lower limit.

- Rapid filtration through silica as described is enough to get a product pure enough to continue the synthesis (around >90%), and is particularly useful while working at large scale. Completely pure product can be obtained by flash column chromatography in SiO_2 eluting with cyclohexane/EtOAc 95:5-9:1. This may be required if a high-vacuum system for the removal of excess mesitylene is not available.

- Ethyl diazoacetate containing ≥13-15% of dichloromethane was employed (and this purity was used to correct the yield) as received from Sigma-Aldrich or ABCR.

(2,4,6-Trimethylcyclohepta-2,4,6-trien-1-yl)methanol (S2)

A dry three-necked 3 L round-bottomed flask equipped with a Teflon-coated magnetic stirring bar was charged with ethyl 2,4,6-trimethylcyclohepta-2,4,6-triene-1-carboxylate **S1** (45 g, 0.218 mol, 1.0 equiv) and after evacuating and refilling the flask with argon, it was dissolved in dry ethyl ether (1.1 L, 0.2 M). The flask was cooled down to 0 ºC with an ice bath, and then lithium aluminum hydride (10,76 g, 0.284 mol, 1.2 equiv) was carefully added in portions of 1 g, one every 3-5 minutes. The resulting suspension was stirred for 16 h whilst warming to room-temperature, and after confirming the disappearance of all the starting material by TLC (a new more polar spot appears, corresponding to the alcohol, which reveals clearly with acidic vanillin stain followed by heat), the mixture is cooled back down to 0 ºC before water is slowly added, up to 300 mL. Then, HCl 10% aqueous solution was added, while the mixture is stirred vigorously until completely dissolving all solids (*ca* 300 mL) in the aqueous phase (which becomes a white suspension; mechanical stirring may be necessary in large-scale). The ethereal phase (upper layer) was decanted and the aqueous phase extracted 2 times with diethyl ether (3x300 mL). The three organic fractions combined were washed with water (1x400 mL) and brine (1x400 mL), and after drying over anhydrous Na_2SO_4, the solvent was removed to give (2,4,6-trimethylcyclohepta-2,4,6-trien-1-yl)methanol **S2** (29 g, 0.177 mol, 86% yield) as a pale-yellow oil with enough purity for the next step (>90%).

^{1}H NMR (500 MHz, $CDCl_3$) δ 6.16 (s, 1H), 5.85 (s, 1H), 5.02 (d, J = 7.3 Hz, 1H), 3.73 – 3.67 (m, 2H), 2.53 (q, J = 7.7 Hz, 1H), 2.00 (d, J = 1.6 Hz, 3H), 1.99 (d, J = 1.4 Hz, 3H), 1.89 (d, J = 1.3 Hz, 3H) ppm.

^{13}C NMR (126 MHz, $CDCl_3$) δ 137.6, 135.0, 134.5, 129.0, 125.3, 117.4, 61.1, 45.4, 24.8, 22.6, 22.3 ppm.

HRMS (APCI Positive): calculated for $C_{11}H_{17}O$ $[M+H]^+$: 165.1274; found: 165.1281.

1-Phenyl-5-(((2,4,6-trimethylcyclohepta-2,4,6-trien-1-yl)methyl)thio)-1*H*-tetrazole (S3)

A dry two-necked 2 L round-bottomed flask equipped with a Teflon-coated magnetic stirring bar was charged with 2,4,6-trimethylcyclohepta-2,4,6-trien-1-yl)methanol **S2** (18 g, 110 mmol, 1.0 equiv) and 1-phenyl-1*H*-tetrazole-5-thiol (19.9 g, 112 mmol, 1.02 equiv) before the atmosphere was evacuated and refilled with argon three times. Everything was dissolved in anhydrous THF (600 mL, *ca*. 0.2 M) before it was cooled down to 0 ºC in an ice bath, and tri(*n*-butyl)phosphine (28.4 ml, 115 mmol, 1.05 equiv) was added via syringe in a single portion. To the resulting solution was added diisopropyl (*E*)-diazene-1,2-dicarboxylate, DIAD (23.1 ml, 115 mmol, 1.05 equiv) via syringe, steadily, in a single portion (over less than 1 min) while stirring vigorously (the orange color of DIAD disappears within seconds during the addition, until >1 equiv is added, then the orange color persists). The mixture was allowed to stir whilst coming to room-temperature over 12 h. 500 mL of water were added, and the aqueous phase was extracted 3 times with diethyl ether (3x400 mL). Combined organic fractions were washed with water once (1x300 mL) and with brine once (1x300 mL), solvent was removed, and the crude product was purified by CombiFlash column chromatography in SiO_2 (2x330 g, purified in two separate flash chromatography runs), using a gradient of (cyclohexane with 5% DCM) and (EtOAc) 9:1 to 8:2 as eluent giving 1-phenyl-5-(((2,4,6-trimethylcyclohepta-2,4,6-trien-1-yl)methyl)thio)-1*H*-tetrazole **S3** (25 g, 77 mmol, 70% yield) as a pale yellow oil.

^{1}H NMR (500 MHz, Chloroform-*d*) δ 7.59 – 7.51 (m, 5H), 6.13 (d, *J* = 2.0 Hz, 1H), 5.84 (t, *J* = 1.5 Hz, 1H), 5.12 (d, *J* = 7.9 Hz, 1H), 3.46 – 3.36 (m, 2H), 2.92 (q, *J* = 8.0 Hz, 1H), 1.97 (d, *J* = 1.4 Hz, 3H), 1.96 (d, *J* = 1.4 Hz, 3H), 1.84 (d, *J* = 1.3 Hz, 3H).

^{13}C NMR (126 MHz, $CDCl_3$) δ 155.00, 137.83, 135.87, 135.13, 134.16, 130.40, 130.12, 129.45, 125.69, 124.24, 118.73, 42.16, 32.34, 25.19, 22.89, 14.42.

GC-MS (EI): calc. for $C_{18}H_{20}N_4S$ $[M]^+$: 324.1; found: 324.1.

1-Phenyl-5-(((2,4,6-trimethylcyclohepta-2,4,6-trien-1-yl)methyl)sulfonyl)-1*H*-tetrazole, Julia-Kocienski Reagent (S4)

Under air, a two neck 2 L round-bottomed flask equipped with a magnetic stirring bar was charged 1-phenyl-5-(((2,4,6-trimethylcyclohepta-2,4,6-trien-1-yl)methyl)thio)-1*H*-tetrazole **S3** (22 g, 68 mmol, 1 equiv) and it was dissolved in HPLC grade ethanol (600 mL, *ca* 0.1 M) and the solution was cooled in an ice bath. A solution of ammonium molybdate tetrahydrate (8.1 g, 6.8 mmol, 10 mol%) in hydrogen peroxide (30% in water) (104 ml, 1.02 mol, 15 equiv) (prepared with only glass material) was added dropwise using a dropping funnel over 1 h, and the resulting mixture was further stirred for 20 h whilst coming to room temperature. After confirming complete conversion of **S3**, water (500 mL) was added, and the mixture was extracted three times with dichloromethane (3x400 mL) (if the two phases do not split, addition of 50 mL of brine can be helpful). Combined organic fractions were washed with water once (1x400 mL) and with brine once (1x400 mL), dried over Na_2SO_4 and concentrated under vacuum. Flash chromatography with CombiFlash in SiO_2 (220 g), using a gradient of (cyclohexane with 5% DCM) and (EtOAc) 9:1 to 8:2 as eluent gave 1-phenyl-5-(((2,4,6-trimethylcyclohepta-2,4,6-trien-1-yl)methyl)-sulfonyl)-1*H*-tetrazole **S4** (18.0 g, 50 mmol, 74 % yield) as a white solid, after sonicating with pentane.

^{1}H NMR (400 MHz, $CDCl_3$) δ 7.69-7.59 (m, 5H), 6.21 (s, 1H), 5.88 (s, 1H), 5.16 (d, *J* = 8.4 Hz, 1H), 3.74 (t, *J* = 8.4 Hz, 2H), 3.42-3.32 (m, 1H), 2.03-2.02 (m, 6H), 1.84 (d, *J* = 1.3 Hz, 3H) ppm.

^{13}C NMR (101 MHz, $CDCl_3$) δ 153.9, 138.2, 135.7, 133.1, 132.2, 131.4, 129.6, 129.5, 126.1, 125.2, 115.2, 54.3, 36.4, 24.7, 24.3 ppm.

HRMS (ESI Pos): calc. for $C_{18}H_{20}N_4NaO_2S$ $[M+Na]^+$: 379.1199; found: 379.1196.

MP 90-93 ºC.

FTIR (ATR) 3060, 3023, 2980, 2916, 2854, 1594, 1497, 1457, 1327, 1313, 1135, 1041, 786, 761, 690, 528 cm^{-1}.

3. List and Preparation of 1,3-Dienes, 7-Alkenyl Cycloheptatrienes and Enynes

1,3-Dienes (**2**) employed in this work were either commercially available, (used as received from commercial sources) or prepared according to literature procedures, and are listed below.

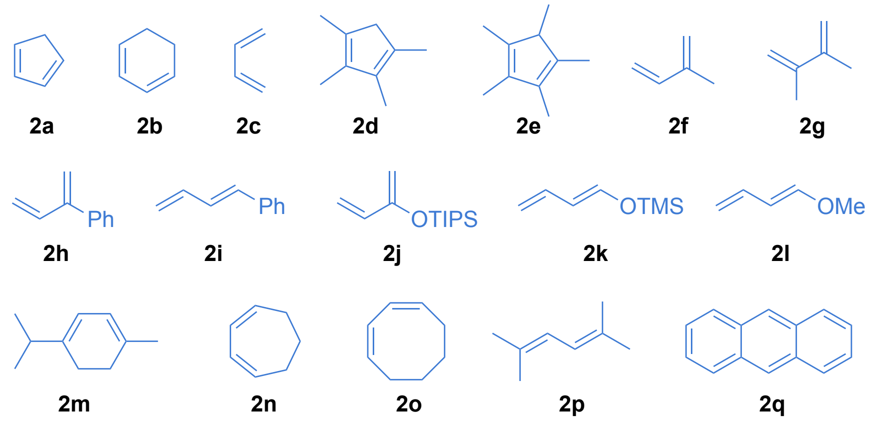

1,3-Cyclopentadiene (**2a**) was obtained by cracking and distillation of its commercial dimer, and used immediately afterwards. 1,3-Butadiene (**2c**) was purchased from Sigma-Aldrich (695580-250G) as a 20% solution in toluene, and used as received. Non-commercial dienes, **2h**[1] and **2j**[2] were prepared according to literature procedures. The synthesis of 7-vinyl-1,3,5-trimethyl-1,3,5-cycloheptatrienes **1** was carried out via Julia-Kocienski olefination with sulfone **S4** (see section above for its preparation) and the corresponding aldehyde as described in General Procedure A (see next section), as reported by us in previous publications.[3] The following cycloheptatrienes were used in this work.

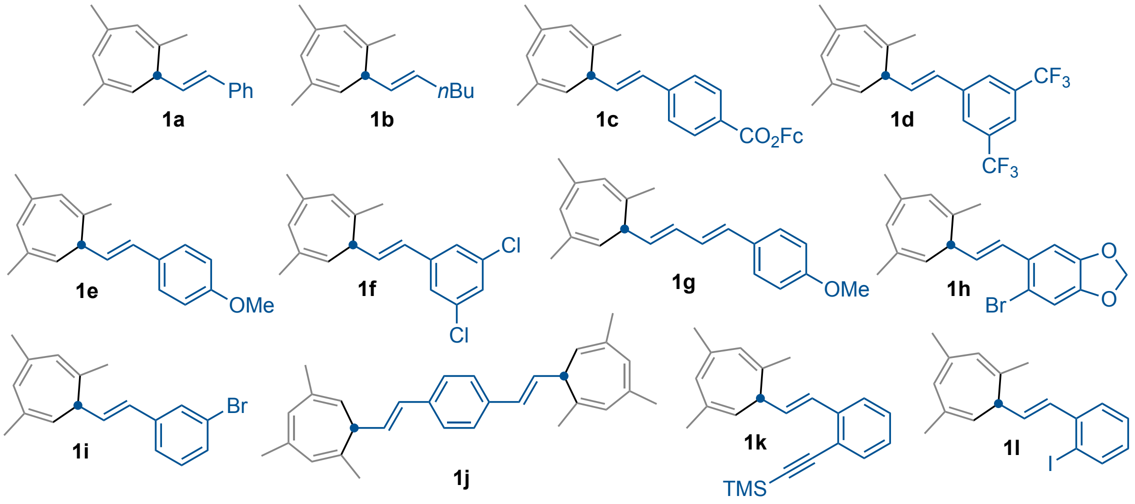

Finally, enynes **6**[4] and **6'**[5] were also prepared according to literature procedures.

4. General Procedure A for the Synthesis of Second Generation Cycloheptatrienes via Julia-Kocienski Olefination

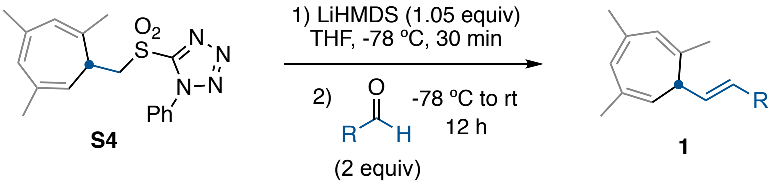

A dry screw-cap culture tube (small scale) or a round-bottomed flask (gram scale) equipped with a Teflon-coated stirring bar was charged with 1-phenyl-5-(((2,4,6-trimethylcyclohepta-2,4,6-trien-1-yl)methyl)sulfonyl)-1*H*-tetrazole **S4** (1 equiv) and dissolved under argon in anhydrous THF (0.1–0.2 M). The mixture was cooled down to -78 ºC using a cryocooler bath, and then, a freshly prepared solution of lithium bis(trimethylsilyl)amide (1.05 equiv) in anhydrous THF (*ca.* 0.5 M) was added dropwise. After stirring for 30 minutes at -78 ºC, to the resulting yellow solution was added the corresponding aldehyde (2.0 equiv), either neat via syringe (liquid substrates) or dissolved in anhydrous THF (solid substrates or small amounts, *ca.* 0.5 M) in a single portion (the mixture usually evolves from intense yellow to pale yellow-colorless). After 15 minutes, the cryocooler was turned off or the flask/tube was taken out of the bath, and the mixture stirred for 8–12 h while coming to room temperature. Water was added, and the mixture was extracted 3 times with diethyl ether. Combined organic fractions were washed twice with water and once with brine and concentrated under vacuum. Crude product was purified by flash column chromatography in SiO_2 using pentane or gradients of pentane/Et_2O as eluent to give the corresponding 7-alkenyl-1,3,5-trimethyl-1,3,5-cycloheptatriene **1**.

Complementary considerations

- 1,3,5-Trimethyl-7-alkenyl-1,3,5-cycloheptatrienes **1** should be stored under Ar in the fridge (5 ºC). No significant decomposition is observed over some weeks if stored under these conditions. Certain substrates undergo decomposition if kept in solution and/or under air. In some cases, cycloheptatrienes (especially the electron-rich ones) are slightly sensitive to the purification treatment, so a fast chromatography is recommended.

- For some cases, the norcaradiene tautomer, which is equilibrium with **1**, can also be observed by NMR.[6]

- Unless stated otherwise, the corresponding 7-alkenyl cyclohepatrienes were obtained as exclusively the *E* isomer (>20:1 by ^{1}H NMR).

4.2 Characterization Data for the Different Styryl Trimethylcycloheptatrienes

(*E*)-1,3,5-Trimethyl-7-styrylcyclohepta-1,3,5-triene (1a)

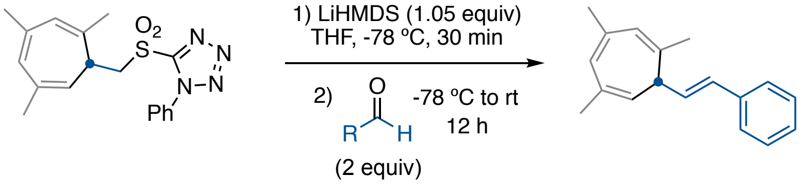

The title compound (pale yellow oil, 1.29 g, 97% yield) was obtained following General Procedure A from reagent **S4** (2.00 g, 5.61 mmol) and benzaldehyde (1.19 g, 11.2 mmol, 2 equiv) using LiHMDS (0.99 g, 5.89 mmol, 1.05 equiv) after purification by flash column chromatography on SiO_2 using pentane to pentane/Et_2O 99:1 as eluent.

^{1}H NMR (400 MHz, Chloroform-*d*) δ 7.40 (d, *J* = 7.0 Hz, 2H), 7.33 (t, *J* = 7.6 Hz, 2H), 7.27 – 7.22 (m, 1H), 6.46 (d, *J* = 2.3 Hz, 2H), 6.25 (s, 1H), 5.84 (s, 1H), 5.11 (d, *J* = 6.7 Hz, 1H), 2.89 – 2.81 (m, 1H), 2.06 – 2.04 (m, 3H), 2.00 (d, *J* = 1.2 Hz, 3H), 1.92 (s, 3H).

^{13}C NMR (101 MHz, $CDCl_3$) δ 137.95, 137.70, 135.04, 132.90, 130.33, 129.43, 129.30, 128.63, 128.48, 128.06, 127.06, 126.19, 124.14, 118.40, 77.34, 77.02, 76.71, 45.93, 24.65, 21.93, 21.84.

HRMS (APCI Positive): calculated for $C_{18}H_{20}$ $[M+H]^+$: 237.1638; found: 237.1637.

(*E*)-1,3,5-Trimethyl-7-(hex-1-en-1-yl)cyclohepta-1,3,5-triene (1b)

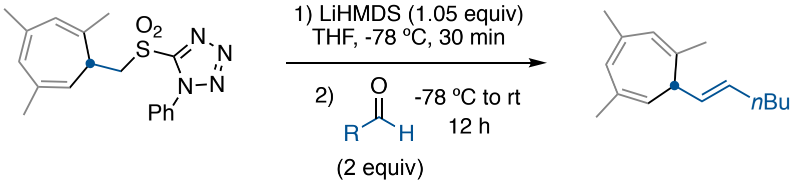

The title compound (pale yellow oil, 0.29 g, 97% yield) was obtained following General Procedure A from reagent **S4** (0.60 g, 1.68 mmol) and pentanal (0.29 g, 3.37 mmol, 2 equiv) using LiHMDS (0.30 g, 1.77 mmol, 1.05 equiv) after purification by CombiFlash column chromatography on SiO_2 using cyclohexane as eluent.

^{1}H NMR (500 MHz, Methylene Chloride-d_2,1.0:0.6 CHT/NCD equilibrium) δ 6.18 (d, *J* = 1.6 Hz, 1H), 5.72 (t, *J* = 1.5 Hz, 1H), 5.68 – 5.63 (m, 1H), 5.44 (ddd, *J* = 15.1, 6.8, 1.1 Hz, 1H), 4.97 – 4.93 (m, 1H), 2.52 (t, *J* = 7.6 Hz, 1H), 2.04 (qd, *J* = 6.8, 1.5 Hz, 2H), 1.97 (d, *J* = 1.3 Hz, 3H), 1.88 (d, *J* = 1.4 Hz, 3H), 1.82 (d, *J* = 1.2 Hz, 3H), 1.34 – 1.29 (m, 4H), 0.92 – 0.88 (m, 3H).

^{13}C NMR (126 MHz, CD_2Cl_2) δ 138.22, 136.37, 132.48, 131.74, 129.92, 129.38, 123.89, 119.92, 46.01, 32.05, 27.39, 24.60, 22.50, 21.76, 21.33, 14.00.

HRMS (GC-MS, EI): calculated for $C_{16}H_{24}$ $[M]^+$: 216.2; found: 216.2.

(*E*)-4-(2-(2,4,6-trimethylcyclohepta-2,4,6-trien-1-yl)vinyl)phenyl ferrocenecarboxylate (1c)

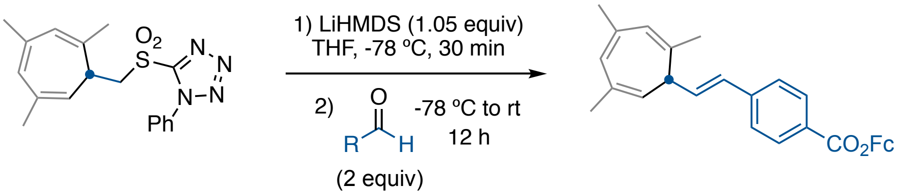

The title compound (amorphous orange foam, 0.19 g, 97% yield) was obtained following General Procedure B from reagent **S4** (0.15 g, 0.42 mmol, 1.0 equiv) and 4-formylphenyl ferrocenecarboxylate[3c] (0.16 g, 0.46 mmol, 1.1 equiv) using LiHMDS (75 mg, 0.44 mmol, 1.05 equiv) after purification by CombiFlash column chromatography on SiO_2 using cyclohexane to cyclohexane/EtOAc 8:2 as eluent.

^{1}H NMR (300 MHz, Chloroform-*d*) δ 7.45 – 7.40 (m, 2H), 7.16 – 7.11 (m, 2H), 6.42 (d, *J* = 6.1 Hz, 2H), 6.24 (s, 1H), 5.84 (t, *J* = 1.4 Hz, 1H), 5.11 (d, *J* = 6.7 Hz, 1H), 4.99 (t, *J* = 2.0 Hz, 2H), 4.52 (q, *J* = 1.8 Hz, 2H), 4.32 (s, 5H), 2.85 (t, *J* = 6.4 Hz, 1H), 2.04 (d, *J* = 1.2 Hz, 3H), 1.99 (d, *J* = 1.4 Hz, 3H), 1.91 (t, *J* = 1.1 Hz, 3H).

^{13}C NMR (75 MHz, $CDCl_3$) δ 170.34, 149.94, 137.99, 135.29, 135.03, 132.98, 129.55, 129.46, 129.32, 127.11, 124.19, 121.73, 118.34, 71.94, 70.66, 70.11, 69.97, 53.45, 45.90, 24.67, 21.95, 21.89.

HRMS (ESI Pos): calculated for $C_{29}H_{29}FeO_2$ $[M+H]^+$: 465.1512; found: 465.1507.

(*E*)-7-(3,5-Bis(trifluoromethyl)styryl)-1,3,5-trimethylcyclohepta-1,3,5-triene (1d)

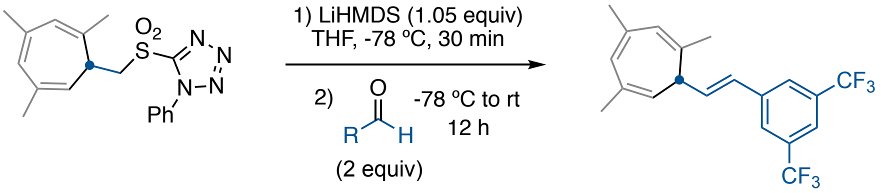

The title compound (colorless oil, 0.41 g, 85% yield) was obtained following General Procedure B from reagent **S4** (0.46 g, 1.21 mmol) and 3,5-bis(trifluoromethyl)benzaldehyde (0.63 g, 2.41 mmol, 2 equiv) using LiHMDS (0.23 g, 1.36 mmol, 1.05 equiv) after purification by CombiFlash column chromatography on SiO_2 using cyclohexane to cyclohexane to cyclohexane/EtOAc 99:1 as eluent.

^{1}H NMR (400 MHz, Chloroform-*d*) δ 7.76 (d, *J* = 1.7 Hz, 2H), 7.72 (s, 1H), 6.52 – 6.48 (m, 1H), 6.24 (s, 1H), 5.87 (t, *J* = 1.5 Hz, 1H), 5.12 (d, *J* = 7.1 Hz, 1H), 2.98 (t, *J* = 7.1 Hz, 1H), 2.04 (t, *J* = 1.8 Hz, 3H), 2.01 (d, *J* = 1.4 Hz, 3H), 1.93 (d, *J* = 1.3 Hz, 3H).

^{13}C NMR (126 MHz, Chloroform-*d*) δ 140.10, 138.29, 134.11 (d, *J* = 5.3 Hz), 129.62, 127.76, 126.30, 125.11, 124.83 (q, *J* = 5.0 Hz), 120.75 (q, *J* = 4.0 Hz), 121.6 (q, *J* = 273 Hz),117.38, 46.11, 24.99, 22.79, 22.43.

^{19}F NMR (376 MHz, $CDCl_3$) δ -63.10.

HRMS (APCI Positive): calculated for $C_{20}H_{18}F_6$ $[M+H]^+$: 373.1313; found: 373.1313.

(*E*)-7-(4-Methoxystyryl)-1,3,5-trimethylcyclohepta-1,3,5-triene (1e)

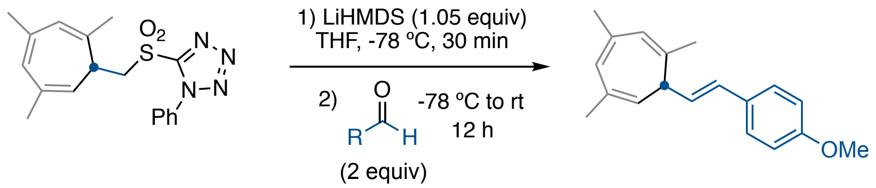

The title compound (pale yellow oil, 0.51 g, 93% yield) was obtained following General Procedure A from reagent **S4** (0.7 g, 1.96 mmol) and 4-methoxybenzaldehyde (0.54 g, 3.93 mmol, 2 equiv) using LiHMDS (0.35 mg, 2.06 mmol, 1.05 equiv) after purification by flash column chromatography on SiO_2 using pentane/Et_2O 99:1 as eluent.

^{1}H NMR (400 MHz, Chloroform-*d*) δ 7.37 – 7.31 (m, 2H), 6.91 – 6.86 (m, 2H), 6.43 – 6.29 (m, 2H), 6.26 (s, 1H), 5.84 (s, 1H), 5.13 – 5.09 (m, 1H), 3.84 (s, 3H), 2.81 (t, *J* = 7.3 Hz, 1H), 2.08 – 2.04 (m, 3H), 2.00 (d, *J* = 1.1 Hz, 3H), 1.92 (s, 3H).

^{13}C NMR (101 MHz, $CDCl_3$) δ 158.87, 137.94, 135.40, 132.73, 130.53, 129.78, 129.31, 127.29, 124.02, 118.76, 113.93, 55.29, 45.98, 24.66, 21.92, 21.76.

HRMS (APCI Positive): calculated for $C_{19}H_{21}O$ $[M–H]^+$: 265.1587; found: 265.1585.

(*E*)-1,3,5-Trimethyl-7-(3,5-dichlorostyryl)cyclohepta-1,3,5-triene (6f)

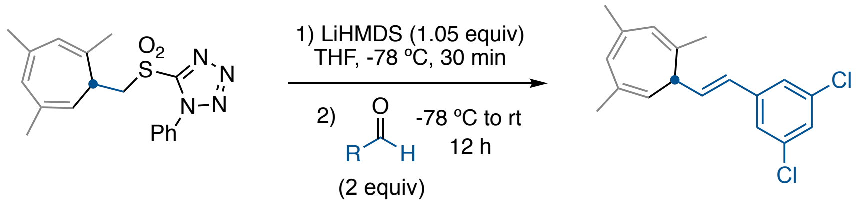

The title compound (colorless oil, 188 mg, 88% yield, 5:1 *E*/*Z*) was obtained following General Procedure A from reagent **S4** (250 mg, 0.70 mmol) and 3,5-dichlorobenzaldehyde (245 mg, 1.40 mmol, 2 equiv) using LiHMDS (123 mg, 0.74 mmol, 1.05 equiv) after purification by flash column chromatography on SiO_2 using pentane to pentane/Et_2O 99:1 as eluent.

^{1}H NMR (500 MHz, Chloroform-*d*) δ 7.20 – 7.16 (m, 3H), 6.38 (dd, *J* = 15.8, 8.2 Hz, 1H), 6.25 (d, *J* = 15.9 Hz, 1H), 6.19 (s, 1H), 5.81 (s, 1H), 5.04 (d, *J* = 6.9 Hz, 1H), 2.85 (t, *J* = 7.5 Hz, 1H), 1.99 (s, 3H), 1.94 (s, 3H), 1.87 (s, 3H).

^{13}C NMR (126 MHz, $CDCl_3$) δ 141.12, 138.28, 135.36, 133.85, 132.74, 129.63, 128.05, 127.35, 127.13, 124.91, 124.89, 117.73, 46.12, 25.02, 22.61, 22.40.

HRMS (APCI Positive): calculated for $C_{18}H_{19}Cl_2$ $[M+H]^+$: 305.0858; found: 305.0863.

7-((1*E*,3*E*)-4-(4-Methoxyphenyl)buta-1,3-dien-1-yl)-1,3,5-trimethylcyclohepta-1,3,5-triene (1g)

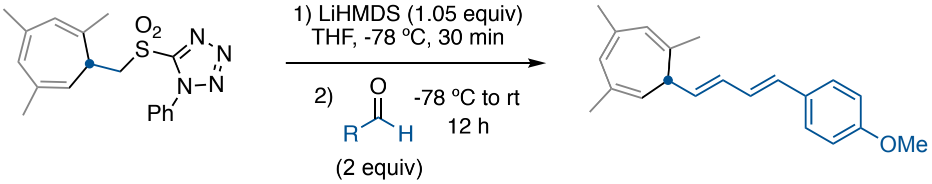

The title compound (yellow viscous oil, 1.75 g, 93% yield, 6:1 *E*/*Z*) was obtained following General Procedure A from reagent **S4** (2.3 g, 6.45 mmol, 1.0 equiv) and (*E*)-3-(4-methoxyphenyl)acrylaldehyde (2.09 g, 12.9 mmol, 2.0 equiv) using LiHMDS (1.13 g, 6.78 mmol, 1.05 equiv) after purification by flash column chromatography on SiO_2 using pentane to pentane/Et_2O 98:2 as eluent.

^{1}H NMR (300 MHz, Chloroform-*d*) δ 7.34 (d, *J* = 8.8 Hz, 2H), 6.88 (s, 2H), 6.70 (dd, *J* = 15.6, 10.2 Hz, 1H), 6.44 (d, *J* = 15.6 Hz, 1H), 6.29 – 6.39 (m, 2H), 5.99 (dd, *J* = 15.1, 8.5 Hz, 1H), 5.83 – 5.79 (m, 1H), 5.04 (d, *J* = 6.7 Hz, 1H), 3.83 (s, 3H), 2.75 (t, *J* = 7.6 Hz, 1H), 2.03 (s, 3H), 1.98 – 1.94 (m, 3H), 1.89 (s, 3H).

^{13}C NMR (126 MHz, $CDCl_3$) δ 159.38, 138.24, 133.18, 133.03, 133.01, 131.49, 130.91, 130.75, 129.56, 128.01, 127.75, 127.58, 124.41, 114.41, 55.66, 46.08, 25.00, 22.31, 22.28.

HRMS (APCI Positive): calculated for $C_{21}H_{25}O$ $[M+H]^+$: 293.1900; found: 293.1910.

(*E*)-5-Bromo-6-(2-(2,4,6-trimethylcyclohepta-2,4,6-trien-1-yl)vinyl)benzo-[*d*][1,3]dioxole (1h)

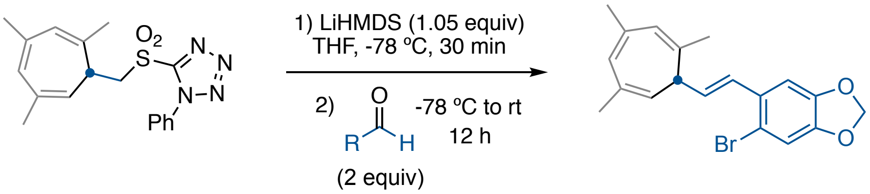

The title compound (pale yellow oil, 0.23 g, 90% yield, >20 *E*/*Z*) was obtained following General Procedure A from reagent **S4** (0.25 g, 0.70 mmol) and 6-bromobenzo[*d*][1,3]dioxole-5-carbaldehyde (0.32 g, 1.40 mmol, 2 equiv) using LiHMDS (0.123 g, 0.74 mmol, 1.05 equiv) after purification by CombiFlash column chromatography on SiO_2 using cyclohexane to cyclohexane/EtOAc 98:2 as eluent.

^{1}H NMR (500 MHz, Chloroform-*d*) δ 6.97 (d, *J* = 4.6 Hz, 2H), 6.63 (d, *J* = 15.7 Hz, 1H), 6.19 (s, 1H), 6.18 – 6.12 (m, 1H), 5.96 – 5.93 (m, 2H), 5.80 (s, 1H), 5.06 (d, *J* = 6.9 Hz, 1H), 2.87 (t, *J* = 7.6 Hz, 1H), 2.00 (s, 3H), 1.95 (d, *J* = 1.2 Hz, 3H), 1.87 (s, 3H).

^{13}C NMR (126 MHz, $CDCl_3$) δ 147.95, 147.86, 138.27, 133.50, 131.30, 130.99, 129.63, 129.23, 124.70, 118.44, 114.61, 112.94, 106.64, 102.03, 46.16, 25.03, 22.51, 22.39.

HRMS (APCI Pos): calculated for $C_{19}H_{20}BrO_2$ $[M+H]^+$: 359.0641; found: 359.0629.

(*E*)-7-(3-Bromostyryl)-1,3,5-trimethylcyclohepta-1,3,5-triene (1i)

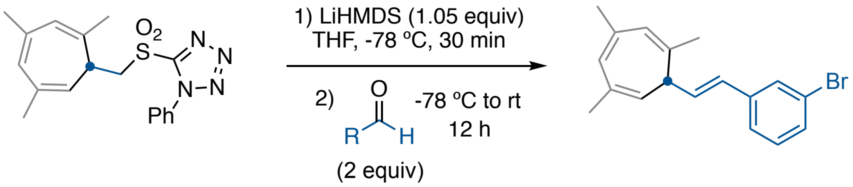

The title compound (pale yellow oil, 140 mg, 79% yield) was obtained following General Procedure A from reagent **S4** (200 mg, 0.56 mmol) and 3-bromobenzaldehyde (208 mg, 1.12 mmol, 2 equiv) using LiHMDS (99 mg, 0.59 mmol, 1.05 equiv) after purification by flash column chromatography on SiO_2 using pentane to pentane/Et_2O 99:1 as eluent.

^{1}H NMR (400 MHz, Chloroform-*d*) δ 7.54 (t, *J* = 1.8 Hz, 1H), 7.36 (ddd, *J* = 7.9, 2.0, 1.1 Hz, 1H), 7.28 (dt, *J* = 7.8, 1.4 Hz, 1H), 7.22 – 7.17 (m, 1H), 6.47 – 6.36 (m, 2H), 6.24 (d, *J* = 1.8 Hz, 1H), 5.85 (t, *J* = 1.4 Hz, 1H), 5.10 (dt, *J* = 6.9, 1.4 Hz, 1H), 2.87 (t, *J* = 7.3 Hz, 1H), 2.04 (d, *J* = 1.3 Hz, 3H), 1.99 (d, *J* = 1.5 Hz, 4H), 1.92 (t, *J* = 1.1 Hz, 3H).

^{13}C NMR (126 MHz, $CDCl_3$) δ 139.90, 137.95, 133.21, 130.99, 130.00, 129.91, 129.30, 128.98, 128.96, 128.85, 124.93, 124.37, 122.73, 117.89, 45.86, 24.67, 22.06, 22.00.

HRMS (APCI Pos): calculated for $C_{18}H_{20}{}^{79}Br$ $[M+H]^+$: 315.0743; found: 315.0744.

1,4-Bis((*E*)-2-(2,4,6-trimethylcyclohepta-2,4,6-trien-1-yl)vinyl)benzene (1j)

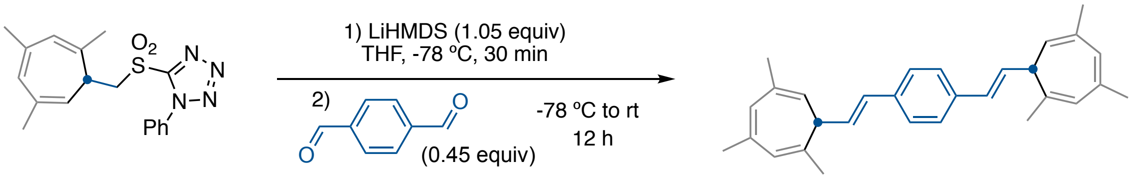

The title compound (viscous yellow oil, 139 mg, 94% yield) was obtained following General Procedure A from reagent **S4** (306 mg, 0.86 mmol, 1.15 equiv) and terephthalaldehyde (50 mg, 0.37 mmol, 0.45 equiv) using LiHMDS (149 mg, 0.90 mmol, 1.20 equiv) after purification by CombiFlash column chromatography on SiO_2 using cyclohexane as eluent.

^{1}H NMR (400 MHz, Chloroform-*d*) δ 7.30 (s, 4H), 6.42 – 6.34 (m, 4H), 6.22 – 6.20 (m, 2H), 5.80 (dt, *J* = 4.3, 1.5 Hz, 2H), 5.11 – 5.05 (m, 2H), 2.81 (t, *J* = 6.8 Hz, 2H), 2.00 (dd, *J* = 3.4, 1.2 Hz, 6H), 1.95 (dd, *J* = 5.3, 1.5 Hz, 6H), 1.86 (dt, *J* = 4.0, 1.1 Hz, 6H).

^{13}C NMR (101 MHz, $CDCl_3$) δ 139.83, 138.48, 136.79, 134.76, 131.66, 130.99, 130.67, 128.15, 125.95, 120.12, 47.83, 26.26, 23.53, 21.53.

HRMS (APCI Pos): calculated for $C_{30}H_{35}$ $[M+H]^+$: 395.2733; found: 395.2736.

(*E*)-Trimethyl((2-(2-(2,4,6-trimethylcyclohepta-2,4,6-trien-1-yl)vinyl)phenyl)ethynyl)silane (1k)

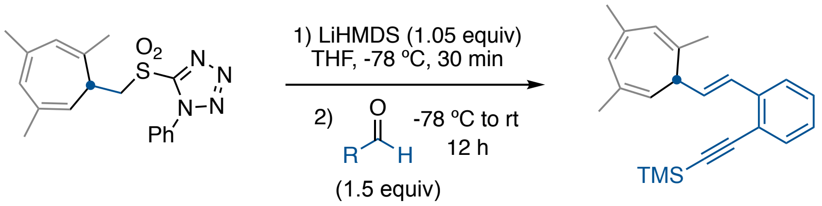

The title compound (pale yellow oil, 0.29 g, 78% yield) was obtained following General Procedure A from reagent **S4** (0.40 g, 1.12 mmol) and 2-((trimethylsilyl)ethynyl)benzaldehyde (0.34 g, 1.68 mmol, 1.5 equiv) using LiHMDS (0.20 g, 1.18 mmol, 1.05 equiv) after purification by CombiFlash column chromatography on SiO_2 using cyclohexane to cyclohexane/EtOAc 99:1 as eluent.

^{1}H NMR (500 MHz, Chloroform-*d*) δ 7.54 – 7.50 (m, 1H), 7.41 (ddd, *J* = 7.7, 1.4, 0.6 Hz, 1H), 7.27 (tdd, *J* = 8.0, 1.5, 0.6 Hz, 1H), 7.15 (td, *J* = 7.5, 1.3 Hz, 1H), 6.90 (dd, *J* = 15.9, 1.4 Hz, 1H), 6.47 (dd, *J* = 15.9, 7.7 Hz, 1H), 6.20 (s, 1H), 5.82 (t, *J* = 1.4 Hz, 1H), 5.14 (d, *J* = 7.0 Hz, 1H), 2.92 (t, *J* = 7.4 Hz, 1H), 2.00 (d, *J* = 1.3 Hz, 3H), 1.97 (d, *J* = 1.4 Hz, 3H), 1.87 (t, *J* = 1.0 Hz, 3H), 0.27 (s, 9H).

^{13}C NMR (126 MHz, $CDCl_3$) δ 141.74, 140.17, 136.89, 135.43, 134.90, 133.64, 131.26, 130.87, 129.92, 128.88, 126.95, 126.56, 123.57, 120.02, 105.78, 101.25, 48.01, 26.60, 24.25, 24.01, 1.92.

HRMS (APCI Pos): calculated for $C_{23}H_{27}Si$ $[M-H]^+$: 331.1877; found: 331.1873.

(*E*)-1,3,5-Trimethyl-7-(2-iodostyryl)cyclohepta-1,3,5-triene (1l)

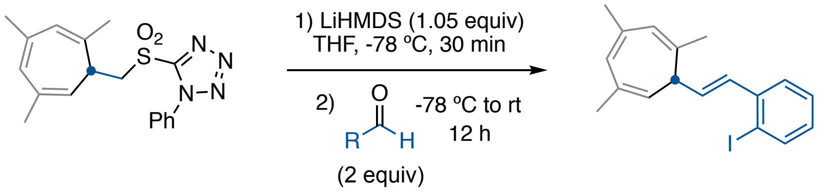

The title compound (pale yellow oil, 0.65 g, 79% yield) was obtained following General Procedure A from reagent **S4** (0.75 g, 2.10 mmol) and 2-iodobenzaldehyde (0.98 g, 4.21 mmol, 2 equiv) using LiHMDS (0.37 g, 2.21 mmol, 1.05 equiv) after purification by CombiFlash column chromatography on SiO_2 using cyclohexane to cyclohexane/EtOAc 98:2 as eluent.

^{1}H NMR (500 MHz, Chloroform-*d*) δ 7.81 (d, *J* = 9.0 Hz, 1H), 7.45 (d, *J* = 7.8 Hz, 1H), 7.28 (t, *J* = 7.5 Hz, 1H), 6.92 – 6.87 (m, 1H), 6.57 (d, *J* = 15.6 Hz, 1H), 6.28 – 6.18 (m, 2H), 5.82 (s, 1H), 5.09 (d, *J* = 6.8 Hz, 1H), 2.92 (t, *J* = 7.5 Hz, 1H), 2.01 (s, 3H), 1.97 (s, 3H), 1.89 (s, 3H).

^{13}C NMR (126 MHz, $CDCl_3$) δ 141.21, 139.77, 138.25, 134.29, 133.61, 132.87, 129.69, 128.93, 128.63, 126.93, 124.80, 118.31, 100.01, 46.06, 27.30, 25.09, 22.44.

HRMS (APCI Positive): calculated for $C_{18}H_{20}I$ $[M+H]^+$: 363.0604; found: 363.0607.

5. Selected Optimization and Control Experiments

Rh(II)-catalyzed decarbenation-cycloaddition sequence:

Optimization and control experiments

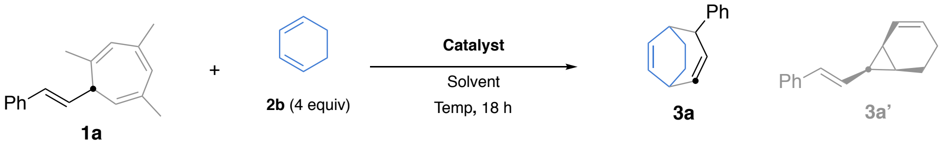

Entry	Catalyst	Solvent	Temperature	Yield/Conversion
1	**Rh_2TFA_4 (5 mol%)**	**1,2-DCE**	**40 °C**	**3a: 91% (83%) (1a: 100% conversion)**
2	Rh_2TFA_4 (5 mol%) **Under Air**	1,2-DCE	40 °C	**3a:** 75% (**1a**: 100% conversion)
3	Rh_2TFA_4 (5 mol%)	1,2-DCE	**25 °C**	**3a:** 82% (**1a**: 100% conversion)
4	Rh_2TFA_4 (5 mol%)	1,2-DCE	**80 °C**	**3a:** 65% (**1a**: 100% conversion)
5	**[Au(JohnPhos)(MeCN)]SbF_6 (5 mol%)**	1,2-DCE	40 °C	**3a:** 70% (**1a**: 100% conversion)
6	**$ZnBr_2$ (20 mol%)**	1,2-DCE	40 °C	**3a:** 8% (**1a**: 23% conversion)
7	**$InCl_3$ (20 mol%)**	1,2-DCE	40 °C	**3a:** n/d (**1a**: 100% conversion)
8	**$Rh_2(esp)_2$ (5 mol%)**	1,2-DCE	40 °C	**3a:** 7% (**1a**: 42% conversion)

We did a couple of preliminary attempts of inducing enantioselectivity on the transformation with a chiral Rh(II) catalyst and a Zn(II)-based system previously reported by us for another transformation.[3a] More studies on this regard are currently being carried out.

Inducing enantioselectivity attempts

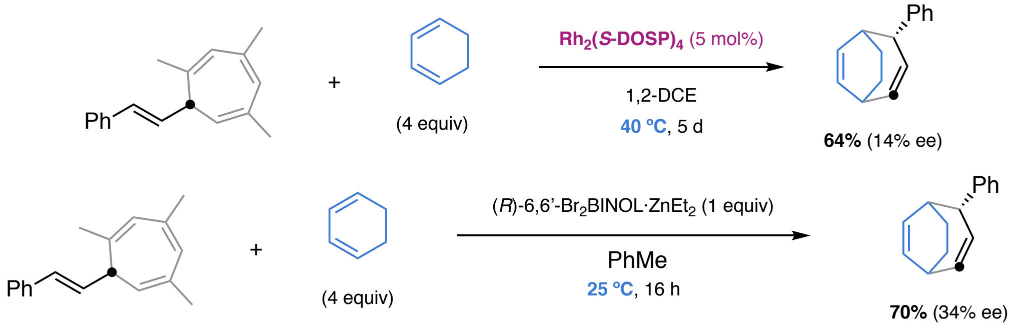

Optimization of the Au(I)-catalyzed cycloisomerization/cycloaddition Reaction:
In this section we included the initial optimization of the reaction of **6'** and **2j** divided by catalyst, solvent, ratio of reactants, catalyst loading/concentration and temperature screenings. The main side-product was found to be cyclopentene **9**.[7] Then we tested other migrating groups and found that OPNP was better migrating group than OAc, so we used **6** for the scope evaluation. Finally, we found that the reaction outcome was very dependent on the diene partner, so we did a catalyst evaluation with less electronically biased cyclopentadiene (**2a**), also included in this section.

Catalyst screening:

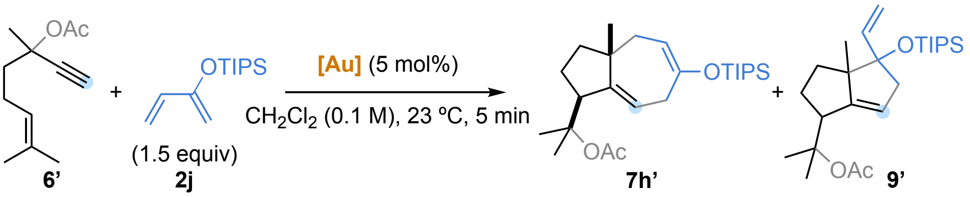

Entry	Catalyst	Yield 7h'	Yield 9
1	**A**	36%	2% + 4%
2	**B**	17%	14%
3	**C**	18%	-
4	**D**	33%	-
5	**G**	22%	2%
6	**H**	25%	-

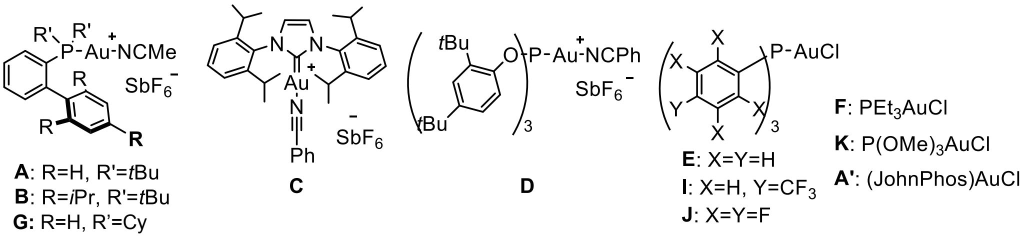

Solvent screening

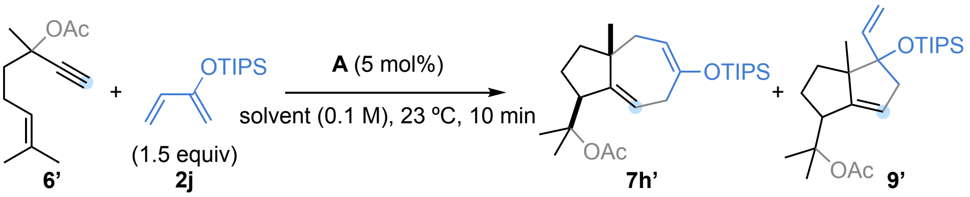

Entry	Solvent	Yield 7h'	Yield 9
1	CH_2Cl_2	36%	2% + 4%
2	EtOAc	22%	-
3	PhMe	23%	traces
4	1,4-dioxane	22%	-
5	acetone	-	-
6	$(CHCl_2)_2$	23%	-
7	heptane	39%	3% + 2%

Diene equivalents/temperature evaluation

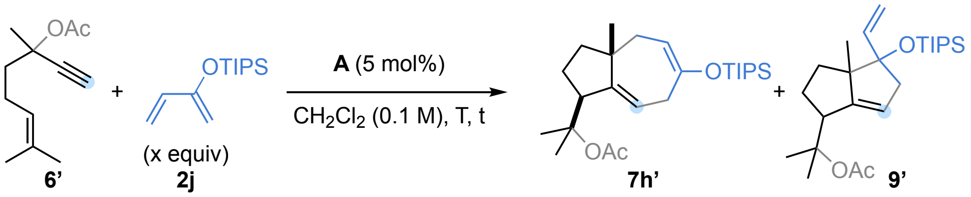

Entry	Diene equiv.	Temperature (°C)	time (min)	Yield 7h'	Yield 9
1	1.5	23	5	36%	2% + 4%
2	3.0	23	5	47%	2% + 5%
3	5.0	23	5	49%	2% + 5%
4	1.5	0	15	34%	6% + 10%
5	3.0	0	15	45%	3% + 7%

Second solvent screening

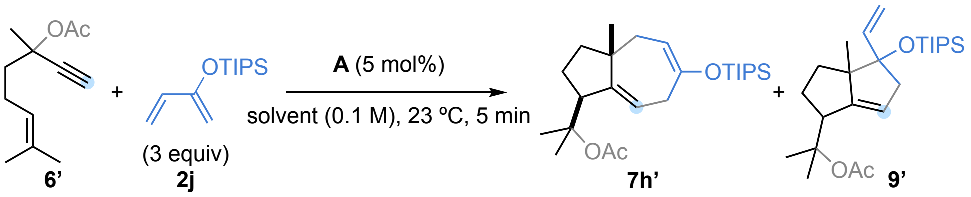

Entry	Solvent	Yield 7h'	Yield 9
1	CH_2Cl_2	47%	2% + 5%
2	CH_2Cl_2/heptane (1:1)	44%	-
3	$(CH_2Cl)_2$	41%	6% + 10%
4	$CHCl_3$	49%	-

Catalyst loading/concentration evaluation

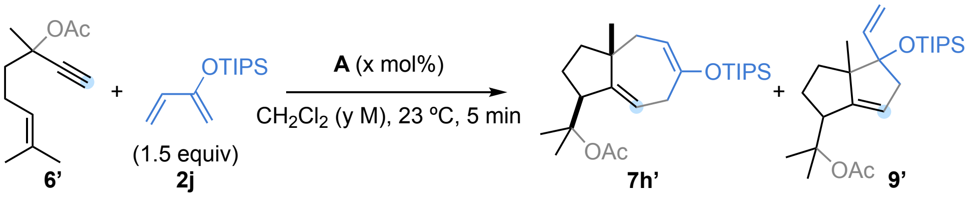

Entry	Catalyst mol%	Concentration (M)	Yield 7h'	Yield 9
1	5	0.1	36%	2% + 4%
2	5	0.2	41%	6% + 10%
3	5	0.5	38%	2% + 4%
4	5	1.0	35%	2% + 4%
5	2	0.1	40%	4% + 7%
6	1	0.5	38%	3% + 6%

Migrating group evaluation

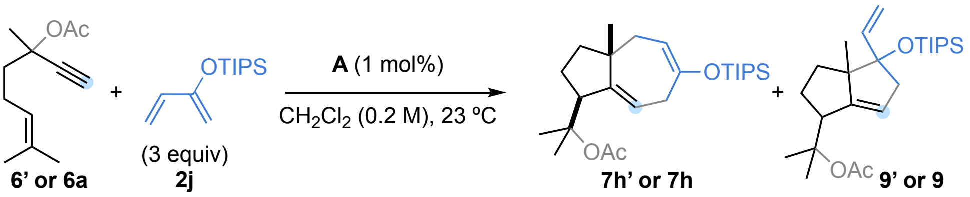

Entry	R^1	Yield 7h	Yield 9
1	Ac	47%	traces
2[a]	PNP	68%	7%
3[b]	Me	-	-
4[b]	Bn	-	-

a. 2h reaction time. b. Unknown products not matching **7** or **9** analogues.

Catalyst evaluation with cyclopentadiene (**2a**)

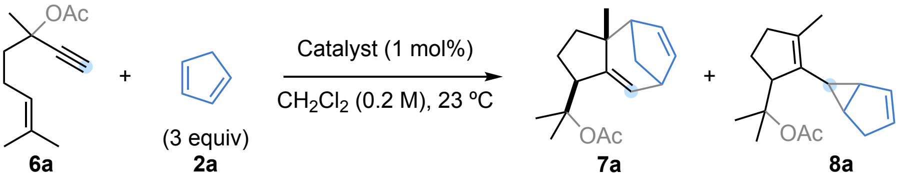

Entry	Catalyst	Conversion	Yield 7h	Yield 8h
1[a]	**A**	100%	26% (7:1)	51% (12:1)
2[a]	**B**	93%	40% (5.7:1)	36%
3[a]	**C**	100%	36% (17:1)	12%
4[a]	**D**	100%	60% (5:1)	4%
5[a,b]	**E**	100%	40% (4.7:1)	19%
6[a,b]	**F**	91%	27% (4.4:1)	31%
7[a,b]	**I**	86%	48% (5:1)	8%
8[c,b]	**J**	100%	18%	traces
9[a,b]	**K**	91%	44% (4.5:1)	13%
10[c,d]	$[Rh(TFA)]_2$	-	-	-
11[c]	$PtCl_2$	90%	traces	-
12[c]	$InBr_3$	83%	-	-
13[c]	$AuCl_3$	35%	9%	traces
14[c]	$AgSbF_6$	30%	-	-
15[c]	AuCl	33%	6%	traces
16[a,b]	**A’**	90%	25% (7.3:1)	42%
17[c]	**A**	100%	18% (8:1)	30%

a. 2 h reaction time. b. Addition of 1 mol% of $NaBAr^F{}_4$. c. 24 h reaction time. d. 40 °C.

6. General Procedure B: Rhodium(II)-Catalyzed Decarbenation–(4+3) Cycloaddition

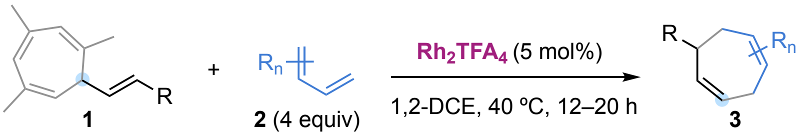

A screw-cap culture tube or microwave vial equipped with a Teflon-coated magnetic stirring bar was charged with the corresponding 1,3,5-trimethyl-7-styryl-1,3,5-cycloheptatriene **1** (1.0 equiv) and the corresponding 1,3-diene **2** (4 equiv). The vial was introduced in an argon-filled glovebox, and both reagents were dissolved in anhydrous 1,2-DCE (0.1 M), before $[Rh(TFA)_2]_2$ (5 mol %) was added. The vial was closed and taken outside the glovebox, and then stirred at 40 ºC for 12–20 hours. After confirming that the reaction was completed by TLC, the resulting mixture was concentrated in vacuum, adsorbed into silica gel, and purified by flash column chromatography or preparative TLC on silica gel, using pentane or gradients of pentane/diethyl ether.

Complementary Considerations

- For reproducibility reasons, the reactions were performed under Ar, using Rh(II) complexes stored in an Ar-filled glovebox. However, most of the reactions can be carried out under air atmosphere, with no special precautions taken to exclude water or oxygen, using a Rh(II) catalyst stored in a desiccator, and HPLC-grade solvent, without a very significant decrease of the chemical yield.

- NMR is the most reliable way to monitor the transformations. The reaction is complete when no starting 1,3,5-trimethyl-7-styryl-1,3,5-cycloheptatriene is left. TLC can also be used for this purpose: staining the plates with a solution of phosphomolybdic acid in ethanol allows visualizing the remaining starting material without heating the plate. The corresponding decomposition products of the starting cycloheptatriene (which have roughly the same R_f than the starting material), are usually only visible after staining and heating the plate. The presence of only the latter, indicates that the reaction is finished. The reaction product can be usually observed clearly by GC-MS, but it is not a good way to determine complete conversion of the cycloheptatriene.

- If the desired outcome for a substrate outside the reported scope is not achieved (especially if the main observed product is the divinylcyclopropane before Cope rearrangement), probably the first variable that should be screened is the reaction temperature. We found that 40–60 ºC generally gave the best yields for the reported substrates in a reasonable amount of time (12-20 h).

6.1 Characterization Data for the Different Cycloheptadienes (3) and Cyclopropanes (3')

(±)-(1*S*,4*R*,5*R*)-4-Phenylbicyclo[3.2.2]nona-2,6-diene (3a)

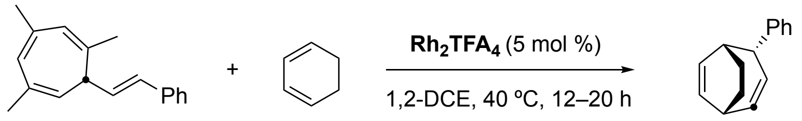

The title compound (pale yellow oil, 163 mg, 82% yield) was obtained following General Procedure B from (*E*)-1,3,5-trimethyl-7-styrylcyclohepta-1,3,5-triene (240 mg, 1.02 mmol) and 1,3-cyclohexadiene (324 mg, 4.02 mmol, 4 equiv) using $[Rh(TFA)_2]_2$ (13.6 mg, 3 mol %) after purification by flash column chromatography on SiO_2 using pentane as eluent.

^{1}H NMR (500 MHz, Chloroform-*d*) δ 7.28 – 7.24 (m, 2H), 7.22 – 7.16 (m, 3H), 6.43 (dd, *J* = 8.8, 7.4 Hz, 1H), 6.16 (ddd, *J* = 10.7, 8.3, 2.3 Hz, 1H), 5.58 – 5.49 (m, 1H), 5.35 (ddd, *J* = 10.9, 3.8, 1.8 Hz, 1H), 3.51 (td, *J* = 3.8, 2.4 Hz, 1H), 2.75 – 2.65 (m, 2H), 2.11 (ddt, *J* = 12.0, 9.7, 2.5 Hz, 1H), 2.06 – 1.99 (m, 1H), 1.93 (dddd, *J* = 13.5, 11.3, 6.8, 2.9 Hz, 1H), 1.75 – 1.67 (m, 1H).

^{13}C NMR (126 MHz, $CDCl_3$) δ 143.70, 138.70, 134.01, 130.77, 129.28, 128.65, 128.30, 126.49, 51.89, 39.94, 33.11, 30.84, 26.58.

HRMS (APCI Pos): calculated for $C_{15}H_{15}$ $[M-H]^+$: 195.1168; found: 195.1168.

(±)-(4-((1*R*,2*R*,5*S*)-Bicyclo[3.2.2]nona-3,6-dien-2-yl)phenyl ferrocenoylate (3b)

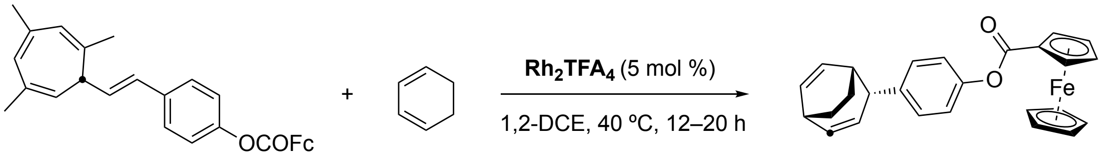

The title compound (orange solid, 36 mg, 72% yield) was obtained following General Procedure B from (*E*)-4-(2-(2,4,6-trimethylcyclohepta-2,4,6-trien-1-yl)vinyl)phenyl ferrocenoylate (55 mg, 0.118 mmol) and 1,3-cyclohexadiene (38 mg, 0.47 mmol, 4 equiv) using $[Rh(TFA)_2]_2$ (3.5 mg, 5 mol %) after purification by preparative TLC in 95:5 pentane/Et_2O (3 elutions).

^{1}H NMR (500 MHz, Chloroform-*d*) δ 7.24 – 7.20 (m, 2H), 7.09 – 7.03 (m, 2H), 6.43 (dd, *J* = 8.7, 7.4 Hz, 1H), 6.16 (ddd, *J* = 10.7, 8.3, 2.3 Hz, 1H), 5.59 – 5.53 (m, 1H), 5.34 (ddd, *J* = 10.9, 3.8, 1.7 Hz, 1H), 4.95 (t, *J* = 1.7 Hz, 2H), 4.48 (t, *J* = 1.8 Hz, 2H), 4.30 (s, 5H), 3.53 (td, *J* = 3.8, 2.3 Hz, 1H), 2.74 – 2.65 (m, 2H), 2.11 (ddt, *J* = 12.1, 9.6, 2.6 Hz, 1H), 2.06 – 1.99 (m, 1H), 1.93 (dddd, *J* = 13.5, 11.2, 6.8, 2.9 Hz, 1H), 1.75 – 1.67 (m, 1H).

^{13}C NMR (126 MHz, $CDCl_3$) δ 170.73, 149.60, 140.98, 138.80, 134.10, 130.62, 129.59, 129.22, 121.39, 72.34, 71.09, 70.72, 70.40, 51.27, 39.90, 33.11, 30.80, 26.48.

HRMS (ESI+): calculated for $C_{26}H_{24}NaO_2{}^{56}Fe$ $[M+Na]^+$: 447.1018; found: 447.1013.

MP: 120–124 °C.

(±)-(1*S*,4*R*,5*R*)-4-(3,5-Bis(trifluoromethyl)phenyl)bicyclo[3.2.2]nona-2,6-diene (3c)

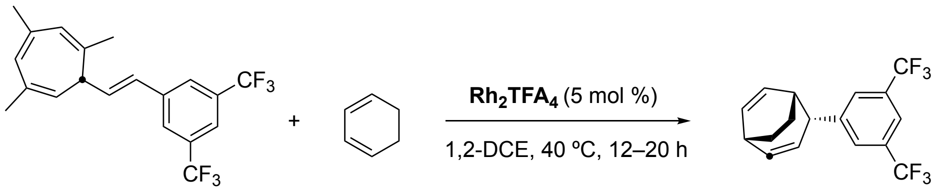

The title compound (colorless oil, 60 mg, 67% yield) was obtained following General Procedure B from (*E*)-7-(3,5-bis(trifluoromethyl)styryl)-1,3,5-trimethylcyclohepta-1,3,5-triene (100 mg, 0.27 mmol) and 1,3-cyclohexadiene (86 mg, 1.07 mmol, 4 equiv) using $[Rh(TFA)_2]_2$ (7.9 mg, 5 mol %) after purification by flash column chromatography on SiO_2 using pentane as eluent.

^{1}H NMR (500 MHz, Chloroform-*d*) δ 7.70 (d, *J* = 2.2 Hz, 1H), 7.63 (d, *J* = 1.7 Hz, 2H), 6.51 – 6.46 (m, 1H), 6.25 (ddd, *J* = 10.7, 8.4, 2.2 Hz, 1H), 5.47 – 5.41 (m, 1H), 5.28 (ddd, *J* = 10.9, 3.9, 1.8 Hz, 1H), 3.63 (td, *J* = 3.9, 2.3 Hz, 1H), 2.79 – 2.73 (m, 1H), 2.70 – 2.65 (m, 1H), 2.13 (ddt, *J* = 12.0, 9.5, 2.6 Hz, 1H), 2.06 – 1.94 (m, 2H), 1.76 – 1.69 (m, 1H).

^{13}C NMR (126 MHz, $CDCl_3$) δ 146.03, 139.78, 135.64, 131.60 (q, *J* = 31 Hz), 128.87 (br), 128.59, 127.97, 123.87 (q, *J* = 273 Hz), 120.63 (quint, *J* = 3.9 Hz), 51.12, 39.63, 33.10, 30.52, 26.24.

^{19}F NMR (471 MHz, $CDCl_3$) δ -63.11.

HRMS (APCI Pos): calculated for $C_{17}H_{15}F_6$ $[M+H]^+$: 333.1072; found: 333.1073.

(±)-(1*S*,4*R*,5*R*)-4-(4-Methoxyphenyl)bicyclo[3.2.2]nona-2,6-diene (3d)

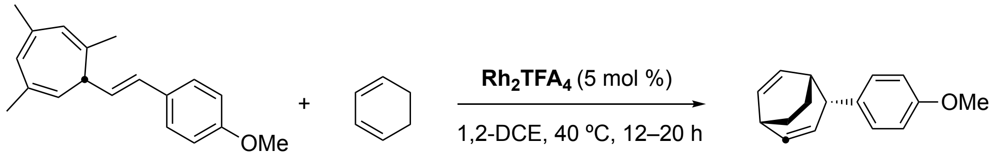

The title compound (pale yellow oil, 63 mg, 82% yield) was obtained following General Procedure B from (*E*)-7-(4-methoxystyryl)-1,3,5-trimethylcyclohepta-1,3,5-triene (90 mg, 0.338 mmol) and 1,3-cyclohexadiene (108 mg, 1.35 mmol, 4 equiv) using $[Rh(TFA)_2]_2$ (10.0 mg, 5 mol %) after purification by preparative TLC on SiO_2 using pentane/Et_2O 99:1 as eluent (3 elutions).

^{1}H NMR (400 MHz, Chloroform-*d*) δ 7.18 – 7.13 (m, 2H), 6.87 – 6.82 (m, 2H), 6.50 – 6.43 (m, 1H), 6.17 (ddd, *J* = 10.7, 8.3, 2.3 Hz, 1H), 5.61 – 5.55 (m, 1H), 5.36 (dddd, *J* = 10.9, 3.8, 1.8, 0.6 Hz, 1H), 3.82 (s, 3H), 3.50 (td, *J* = 3.8, 2.3 Hz, 1H), 2.75 – 2.67 (m, 2H), 2.14 (ddt, *J* = 11.8, 9.4, 2.5 Hz, 1H), 2.08 – 1.91 (m, 2H), 1.77 – 1.70 (m, 1H).

^{13}C NMR (101 MHz, $CDCl_3$) δ 158.04, 138.31, 135.58, 133.34, 130.78, 129.18, 129.05, 113.32, 55.23, 50.75, 39.69, 32.75, 30.48, 26.08.

HRMS (APCI Pos): calculated for $C_{16}H_{19}O$ $[M+H]^+$: 227.1430; found: 227.1426.

(±)-(1*S*,4*R*,5*R*)-4-(3,5-Dichlorophenyl)bicyclo[3.2.2]nona-2,6-diene (3e)

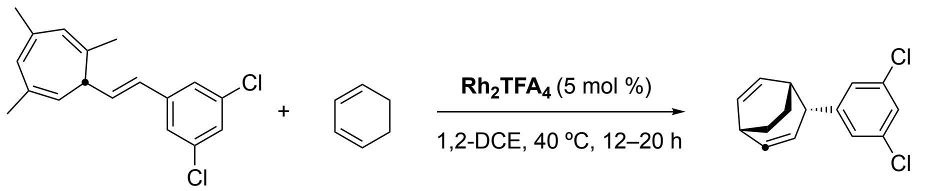

The title compound (colorless oil, 55 mg, 70% yield) was obtained following General Procedure B from (*E*)-7-(3,5-dichlorostyryl)-1,3,5-trimethylcyclohepta-1,3,5-triene (90 mg, 0.295 mmol) and 1,3-cyclohexadiene (95 mg, 1.18 mmol, 4 equiv) using $[Rh(TFA)_2]_2$ (8.7 mg, 5 mol %) after purification by flash column chromatography on SiO_2 using pentane as eluent.

^{1}H NMR (500 MHz, Chloroform-*d*) δ 7.17 (t, *J* = 1.9 Hz, 1H), 7.06 (dd, *J* = 1.9, 0.5 Hz, 2H), 6.44 (dd, *J* = 8.8, 7.4 Hz, 1H), 6.18 (ddd, *J* = 10.7, 8.3, 2.2 Hz, 1H), 5.53 – 5.47 (m, 1H), 5.24 (dddd, *J* = 10.9, 3.8, 1.8, 0.6 Hz, 1H), 3.44 (td, *J* = 3.8, 2.3 Hz, 1H), 2.74 – 2.68 (m, 1H), 2.65 (tdd, *J* = 6.9, 3.7, 1.3 Hz, 1H), 2.12 – 2.06 (m, 1H), 2.02 – 1.89 (m, 2H), 1.69 (dddd, *J* = 12.3, 10.8, 6.3, 4.4 Hz, 1H).

^{13}C NMR (126 MHz, $CDCl_3$) δ 147.12, 139.35, 135.14, 134.73, 129.09, 128.46, 127.21, 126.72, 51.02, 39.59, 33.06, 30.57, 26.31.

HRMS (APCI Pos): calculated for $C_{15}H_{15}Cl_2$ $[M+H]^+$: 265.0545; found: 265.0540.

(±)-(1*S*,4*R*,5*R*)-4-(1-Iodophenyl)bicyclo[3.2.2]nona-2,6-diene (3f)

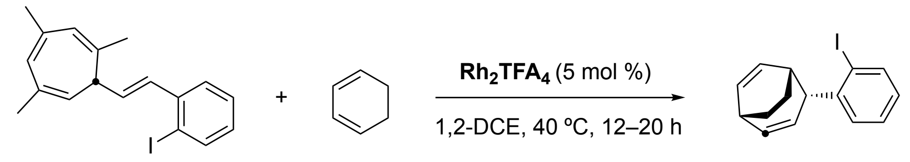

The title compound (viscous colorless oil, 70 mg, 61% yield) was obtained following General Procedure B from (*E*)-7-(2-iodostyryl)-1,3,5-trimethylcyclohepta-1,3,5-triene (130 mg, 0.359 mmol) and 1,3-cyclohexadiene (115 mg, 1.44 mmol, 4 equiv) using $[Rh(TFA)_2]_2$ (10.7 mg, 5 mol %) after purification by flash column chromatography on SiO_2 using pentane as eluent.

^{1}H NMR (500 MHz, Chloroform-*d*) δ 7.80 (dd, *J* = 7.9, 1.3 Hz, 1H), 7.22 (td, *J* = 7.5, 1.3 Hz, 1H), 7.13 (dd, *J* = 7.7, 1.8 Hz, 1H), 6.87 (ddd, *J* = 7.8, 7.2, 1.8 Hz, 1H), 6.42 (ddd, *J* = 8.6, 7.2, 1.0 Hz, 1H), 6.20 (ddd, *J* = 10.8, 8.3, 2.3 Hz, 1H), 5.50 – 5.44 (m, 1H), 5.31 – 5.23 (m, 1H), 3.76 (td, *J* = 3.6, 2.4 Hz, 1H), 2.79 (tq, *J* = 6.8, 3.8, 3.2 Hz, 1H), 2.74 – 2.68 (m, 1H), 2.12 (ddt, *J* = 11.9, 9.7, 2.5 Hz, 1H), 2.08 – 2.02 (m, 1H), 1.94 (dddd, *J* = 13.3, 11.6, 6.8, 2.9 Hz, 1H), 1.76 – 1.69 (m, 1H).

^{13}C NMR (126 MHz, $CDCl_3$) δ 144.78, 139.52, 138.53, 134.37, 130.56, 128.93, 128.47, 128.21, 101.43, 54.87, 36.81, 33.08, 30.65, 26.27.

HRMS (APCI Pos): calculated for $C_{15}H_{16}I$ $[M+H]^+$: 323.0291; found: 323.0291.

(±)-5-((1*R*,2*R*,5*S*)-Bicyclo[3.2.2]nona-3,6-dien-2-yl)-6-bromobenzo[*d*][1,3]dioxole (3g)

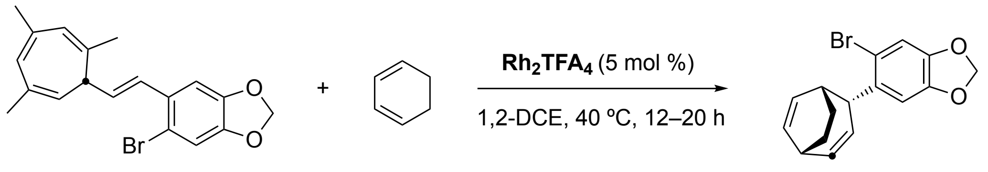

The title compound (pale yellow solid, 80 mg, 60% yield) was obtained following General Procedure B from (*E*)-5-bromo-6-(2-(2,4,6-trimethylcyclohepta-2,4,6-trien-1-yl)vinyl)benzo[*d*][1,3]dioxole (150 mg, 0.418 mmol) and 1,3-cyclohexadiene (134 mg, 1.67 mmol, 4 equiv) using $[Rh(TFA)_2]_2$ (12.4 mg, 5 mol %) after purification by flash column chromatography on SiO_2 using pentane/Et_2O 99:1 to 9:1 as eluent.

^{1}H NMR (500 MHz, Chloroform-*d*) δ 6.97 (s, 1H), 6.70 (s, 1H), 6.40 (ddd, *J* = 8.7, 7.2, 1.0 Hz, 1H), 6.16 (ddd, *J* = 10.7, 8.3, 2.3 Hz, 1H), 5.91 (s, 2H), 5.54 – 5.48 (m, 1H), 5.20 (dddd, *J* = 10.9, 3.9, 1.8, 0.6 Hz, 1H), 3.83 (td, *J* = 3.7, 2.3 Hz, 1H), 2.78 – 2.72 (m, 1H), 2.69 (q, *J* = 7.9, 6.5 Hz, 1H), 2.09 (ddt, *J* = 12.2, 9.6, 2.6 Hz, 1H), 2.02 – 1.95 (m, 1H), 1.90 (dddd, *J* = 13.7, 11.3, 6.8, 3.0 Hz, 1H), 1.69 (dddd, *J* = 12.2, 10.8, 5.9, 4.5 Hz, 1H).

^{13}C NMR (126 MHz, $CDCl_3$) δ 147.40, 147.02, 138.50, 135.39, 134.45, 130.45, 129.20, 114.23, 112.62, 110.93, 101.81, 49.81, 36.78, 33.04, 30.66, 26.09.

HRMS (APCI Pos): calculated for $C_{16}H_{16}{}^{79}BrO_2$ $[M+H]^+$: 319.0328; found: 319.0326.

MP: 102–105 ºC.

(±)-(1*S*,4*S*,5*R*)-4-((*E*)-4-Methoxystyryl)bicyclo[3.2.2]nona-2,6-diene (3h)

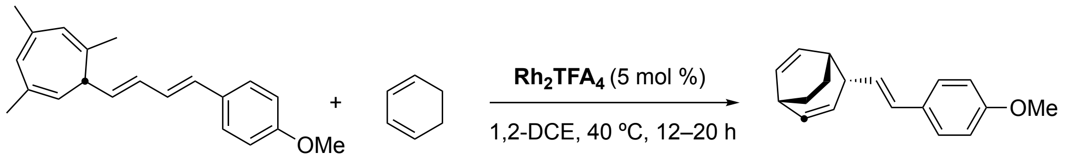

The title compound (pale yellow oil, 25 mg, 48% yield) was obtained following General Procedure B from 7-((1*E*,3*E*)-4-(4-methoxyphenyl)buta-1,3-dien-1-yl)-1,3,5-trimethylcyclohepta-1,3,5-triene (60 mg, 0.205 mmol) and 1,3-cyclohexadiene (66 mg, 0.82 mmol, 4 equiv) using $[Rh(TFA)_2]_2$ (6.0 mg, 5 mol %) after purification by flash column chromatography on SiO_2 using pentane/Et_2O (99:1 to 97:3) as eluent.

^{1}H NMR (500 MHz, Chloroform-*d*) δ 7.28 – 7.25 (m, 2H), 6.83 – 6.80 (m, 2H), 6.48 – 6.41 (m, 1H), 6.30 (d, *J* = 16.0 Hz, 1H), 6.06 (dd, *J* = 15.8, 8.0 Hz, 1H), 6.00 (ddd, *J* = 10.7, 8.3, 2.1 Hz, 1H), 5.89 (ddd, *J* = 8.4, 7.1, 1.0 Hz, 1H), 5.24 (ddd, *J* = 10.8, 4.1, 1.6 Hz, 1H), 3.78 (s, 3H), 2.98 (d, *J* = 7.6 Hz, 1H), 2.68 – 2.57 (m, 2H), 2.10 – 2.04 (m, 1H), 1.93 – 1.86 (m, 2H), 1.67 (ddd, *J* = 10.2, 7.9, 4.5 Hz, 1H).

^{13}C NMR (126 MHz, $CDCl_3$) δ 159.15, 139.30, 133.29, 130.91, 130.67, 130.16, 129.71, 128.90, 127.59, 114.26, 55.66, 49.75, 37.64, 33.15, 30.91, 25.88.

HRMS (APCI Pos): calculated for $C_{18}H_{21}O$ $[M+H]^+$: 253.1587; found: 253.1592.

cis-Trimethyl((4-phenylcyclohepta-2,6-dien-1-yl)oxy)silane (3i)

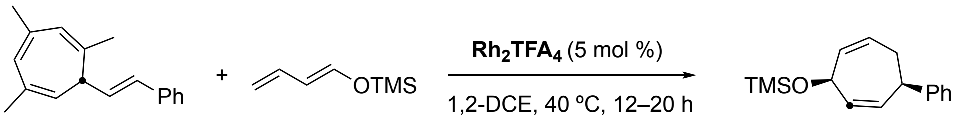

The title compound (pale yellow oil, 12 mg, 37% yield, >20:1 *cis*/*trans*) was obtained following General Procedure B from (*E*)-1,3,5-trimethyl-7-styrylcyclohepta-1,3,5-triene (30 mg, 0.127 mmol) and (*E*)-(buta-1,3-dien-1-yloxy)trimethylsilane (72 mg, 0.51 mmol, 4 equiv) using $[Rh(TFA)_2]_2$ (3.7 mg, 5 mol %) after purification by flash column chromatography on SiO_2 using pentane as eluent.

^{1}H NMR (500 MHz, Chloroform-*d*) δ 7.30 – 7.25 (m, 4H), 7.21 – 7.18 (m, 1H), 5.84 – 5.79 (m, 1H), 5.77 – 5.72 (m, 1H), 5.71 – 5.64 (m, 1H), 5.39 (dddd, *J* = 11.2, 4.1, 2.9, 0.9 Hz, 1H), 4.80 (s, 1H), 3.72 (dd, *J* = 6.5, 3.3 Hz, 1H), 3.12 – 3.03 (m, 1H), 2.75 (dt, *J* = 20.1, 7.0 Hz, 1H).

^{13}C NMR (126 MHz, $CDCl_3$) δ 140.82, 136.04, 130.81, 130.55, 128.54, 127.75, 126.74, 126.48, 72.07, 51.33, 28.98, 0.38.

GCMS (EI): calculated for $C_{16}H_{22}OSi$ $[M]^+$: 258.1; found: 258.2.

cis-3-Methoxy-6-phenylcyclohepta-1,4-diene (3j)

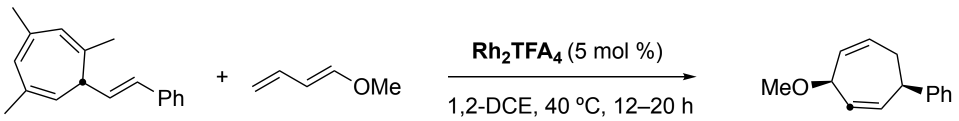

The title compound (pale yellow oil, 21 mg, 83% yield, >20:1 *cis*/*trans*) was obtained following General Procedure B from (*E*)-1,3,5-trimethyl-7-styrylcyclohepta-1,3,5-triene (30 mg, 0.127 mmol) and (*E*)-1-methoxybuta-1,3-diene (43 mg, 0.51 mmol, 4 equiv) using $[Rh(TFA)_2]_2$ (3.7 mg, 5 mol %) after purification by CombiFlash column chromatography on SiO_2 using a gradient between cyclohexane and 95:5 cyclohexane/EtOAc as eluent.

^{1}H NMR (500 MHz, Chloroform-*d*) δ 7.30 – 7.25 (m, 4H), 7.23 – 7.18 (m, 1H), 5.84 – 5.76 (m, 2H), 5.72 (dddd, *J* = 11.7, 6.1, 2.5, 0.7 Hz, 1H), 5.49 (ddd, *J* = 10.3, 5.9, 2.2 Hz, 1H), 4.43 (d, *J* = 5.4 Hz, 1H), 3.91 (tdd, *J* = 4.7, 3.9, 3.4, 2.0 Hz, 1H), 3.39 (s, 3H), 3.14 – 3.04 (m, 1H), 2.84 – 2.74 (m, 1H).

^{13}C NMR (126 MHz, $CDCl_3$) δ 133.83, 130.56, 130.32, 127.93, 127.91, 126.86, 80.89, 57.25, 47.82, 29.10.

GCMS (EI): calculated for $C_{14}H_{16}O$ $[M]^+$: 200.1; found: 200.1.

cis-4-(4-Methoxycyclohepta-2,5-dien-1-yl)phenyl ferrocenoylate (3k)

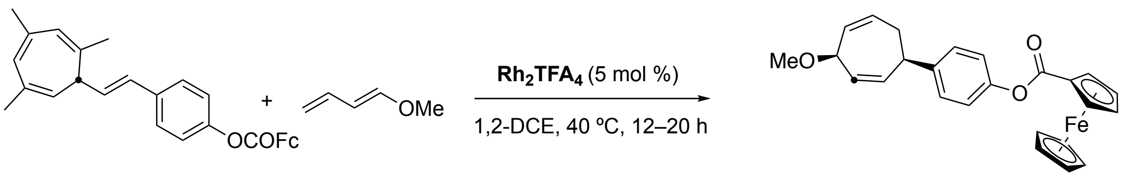

The title compound (amorphous orange residue, 48 mg, 65% yield, >20:1 *cis*/*trans*) was obtained following General Procedure B from (*E*)-4-(2-(2,4,6-trimethylcyclohepta-2,4,6-trien-1-yl)vinyl)phenyl ferrocenoylate (80 mg, 0.172 mmol) and (*E*)-1-methoxybuta-1,3-diene (58 mg, 0.69 mmol, 4 equiv) using $[Rh(TFA)_2]_2$ (5.1 mg, 5 mol %) after purification by CombiFlash column chromatography on SiO_2 using cyclohexane/EtOAc 95:5 to 9:1 as eluent.

^{1}H NMR (500 MHz, Chloroform-*d*) δ 7.36 – 7.29 (m, 2H), 7.10 – 7.04 (m, 2H), 5.84 – 5.75 (m, 2H), 5.72 (ddd, *J* = 11.9, 6.1, 2.5 Hz, 1H), 5.49 (dt, *J* = 11.2, 3.7 Hz, 1H), 4.96 (s, 2H), 4.49 (s, 2H), 4.43 (s, 1H), 4.30 (s, 5H), 3.92 (dt, *J* = 6.4, 3.7 Hz, 1H), 3.39 (s, 3H), 3.09 (d, *J* = 20.3 Hz, 1H), 2.78 (dt, *J* = 20.3, 6.9 Hz, 1H).

^{13}C NMR (126 MHz, $CDCl_3$) δ 170.62, 149.96, 137.94, 133.83, 131.25, 130.43, 128.06, 127.93, 120.96, 80.79, 72.44, 71.18, 70.85, 70.49, 57.22, 47.29, 29.08.

HRMS (ESI+): calculated for $C_{25}H_{24}NaO_3{}^{54}Fe$ $[M+Na]^+$: 449.1014; found: 439.1009.

(±)-(1*S*,4*S*,5*S*)-1,5,6,7,8-Pentamethyl-4-phenylbicyclo[3.2.1]octa-2,6-diene (3l)

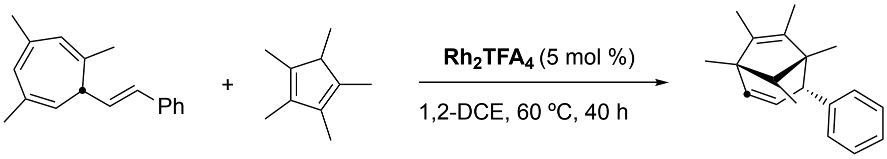

The title compound (pale yellow oil, 17 mg, 53% yield) was obtained following General Procedure B from (*E*)-1,3,5-trimethyl-7-styrylcyclohepta-1,3,5-triene (30 mg, 0.127 mmol) and 1,2,3,4,5-pentamethylcyclopenta-1,3-diene (52 mg, 0.38 mmol, 3 equiv) using $[Rh(TFA)_2]_2$ (3.8 mg, 5 mol %) after purification by flash column chromatography on SiO_2 using pentane as eluent.

^{1}H NMR (400 MHz, Chloroform-*d*) δ 7.28 – 7.18 (m, 3H), 7.07 – 6.99 (m, 2H), 6.12 (dd, *J* = 9.4, 2.4 Hz, 1H), 5.40 (dd, *J* = 9.4, 2.6 Hz, 1H), 3.17 (t, *J* = 2.6 Hz, 1H), 2.02 (q, *J* = 6.8 Hz, 1H), 1.62 (q, *J* = 1.2 Hz, 3H), 1.04 (s, 3H), 1.00 (s, 3H), 0.86 (q, *J* = 1.2 Hz, 3H), 0.78 (d, *J* = 6.8 Hz, 3H).

^{13}C NMR (101 MHz, $CDCl_3$) δ 142.04, 141.29, 140.37, 129.93, 129.14, 128.07, 127.41, 126.21, 57.10, 53.27, 52.54, 47.67, 18.39, 18.23, 12.26, 11.48, 10.46.

HRMS (APCI Pos): calculated for $C_{19}H_{24}$ $[M]^+$: 252.1873; found 252.1876.

(±)-(1*S*,4*S*,5*S*)-1,5,6,7,8-Pentamethyl-4-(3-bromophenyl)bicyclo[3.2.1]octa-2,6-diene (3m)

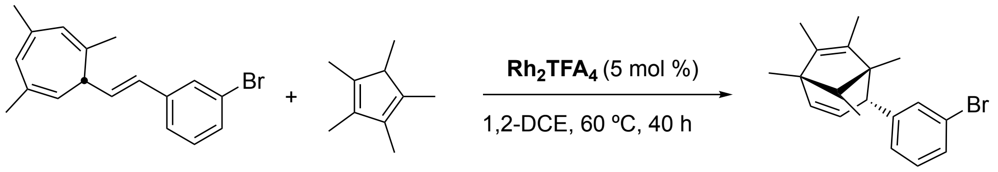

The title compound (colorless viscous oil, 18 mg, 43% yield) was obtained following General Procedure B from (*E*)-7-(3-bromostyryl)-1,3,5-trimethylcyclohepta-1,3,5-triene (40 mg, 0.127 mmol) and 1,2,3,4,5-pentamethylcyclopenta-1,3-diene (52 mg, 0.38 mmol, 3 equiv) using $[Rh(TFA)_2]_2$ (3.8 mg, 5 mol %) after purification by flash column chromatography on SiO_2 using pentane as eluent.

^{1}H NMR (500 MHz, Chloroform-*d*) δ 7.31 (ddd, *J* = 7.9, 2.1, 1.1 Hz, 1H), 7.11 (t, *J* = 1.8 Hz, 1H), 7.08 (t, *J* = 7.8 Hz, 1H), 6.91 (dt, *J* = 7.7, 1.4 Hz, 1H), 6.09 (dd, *J* = 9.4, 2.4 Hz, 1H), 5.30 (dd, *J* = 9.4, 2.6 Hz, 1H), 3.08 (t, *J* = 2.5 Hz, 1H), 1.96 (q, *J* = 6.8 Hz, 1H), 1.58 (q, *J* = 1.2 Hz, 3H), 0.99 (s, 3H), 0.94 (s, 3H), 0.85 (q, *J* = 1.2 Hz, 3H), 0.73 (d, *J* = 6.8 Hz, 3H).

^{13}C NMR (126 MHz, $CDCl_3$) δ 144.94, 142.20, 141.31, 133.18, 129.65, 129.30, 129.20, 128.87, 127.66, 122.07, 57.44, 53.72, 52.57, 48.07, 18.67, 18.57, 12.57, 11.79, 10.83.

HRMS (APCI Pos): calculated for $C_{19}H_{22}{}^{79}Br$ [M-H]: 329.0899; found: 329.0854.

(±)-Trimethyl((2-((1*S*,2*S*,5*S*)-1,5,6,7,8-pentamethylbicyclo[3.2.1]octa-3,6-dien-2-yl)phenyl)ethynyl)silane (3n)

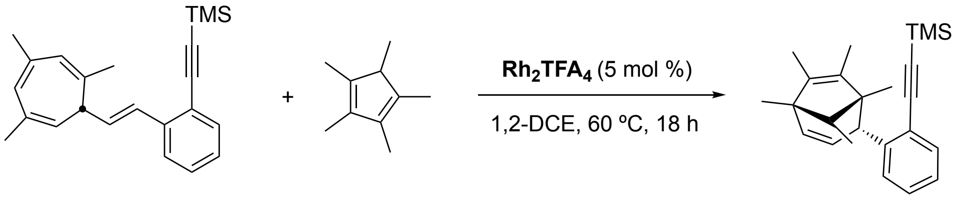

The title compound (viscous colorless oil, 34 mg, 57% yield) was obtained following General Procedure B from (*E*)-trimethyl((2-(2-(2,4,6-trimethylcyclohepta-2,4,6-trien-1-yl)vinyl)phenyl)ethynyl)silane (55 mg, 0.165 mmol) and 1,2,3,4,5-pentamethylcyclopenta-1,3-diene (90 mg, 0.66 mmol, 4 equiv) using $[Rh(TFA)_2]_2$ (4.9 mg, 5 mol %) after purification by flash column chromatography on SiO_2 using pentane as eluent.

^{1}H NMR (500 MHz, Chloroform-*d*) δ 7.41 (ddd, *J* = 7.5, 1.6, 0.6 Hz, 1H), 7.12 (dtd, *J* = 23.6, 7.4, 1.5 Hz, 2H), 6.81 – 6.75 (m, 1H), 6.04 (dd, *J* = 9.4, 2.5 Hz, 1H), 5.27 (dd, *J* = 9.4, 2.6 Hz, 1H), 3.90 (t, *J* = 2.6 Hz, 1H), 2.06 (q, *J* = 6.8 Hz, 1H), 1.60 (q, *J* = 1.2 Hz, 3H), 0.99 (d, *J* = 5.8 Hz, 6H), 0.93 (q, *J* = 1.2 Hz, 3H), 0.74 (d, *J* = 6.8 Hz, 3H), 0.25 (s, 9H).

^{13}C NMR (126 MHz, $CDCl_3$) δ 145.05, 140.95, 140.86, 132.26, 130.54, 129.43, 128.89, 128.49, 126.27, 124.04, 105.69, 98.35, 57.97, 55.35, 49.33, 48.04, 19.23, 18.71, 12.86, 11.67, 10.78, 0.34.

HRMS (APCI Pos): calculated for $C_{24}H_{33}Si$ $[M+H]^+$: 349.2346; found: 349.2346.

(±)-(1*S*,4*R*,5*R*)-5-Isopropyl-1-methyl-4-phenylbicyclo[3.2.2]nona-2,6-diene (3p)

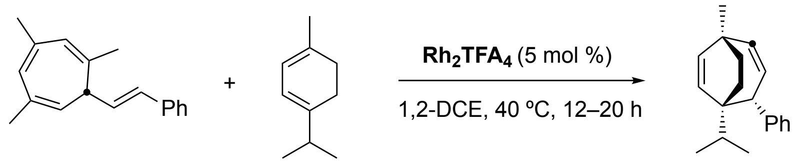

The title compound (viscous colorless oil, 37 mg, 87% yield, 7:1 rr) was obtained following General Procedure B from (*E*)-1,3,5-trimethyl-7-styrylcyclohepta-1,3,5-triene (40 mg, 0.169 mmol) and α-terpinene (1-isopropyl-4-methylcyclohexa-1,3-diene) (69 mg, 0.51 mmol, 4 equiv) using $[Rh(TFA)_2]_2$ (5.0 mg, 5 mol %) after purification by flash column chromatography on SiO_2 using pentane as eluent.

^{1}H NMR (500 MHz, Chloroform-*d*) δ 7.27 – 7.20 (m, 4H), 7.19 – 7.15 (m, 1H), 6.11 (dt, *J* = 9.2, 0.7 Hz, 1H), 5.66 (dd, *J* = 11.1, 2.2 Hz, 1H), 5.27 (d, *J* = 9.2 Hz, 1H), 5.24 (dd, *J* = 11.1, 4.5 Hz, 1H), 3.46 (dd, *J* = 4.6, 2.2 Hz, 1H), 2.00 – 1.95 (m, 1H), 1.77 – 1.65 (m, 2H), 1.52 (p, *J* = 6.8 Hz, 1H), 1.41 (ddd, *J* = 12.0, 10.5, 8.2 Hz, 1H), 1.17 (s, 3H), 0.98 (d, *J* = 6.8 Hz, 3H), 0.74 (d, *J* = 6.7 Hz, 3H).

^{13}C NMR (126 MHz, $CDCl_3$) δ 141.74, 140.88, 135.89, 133.45, 131.71, 130.26, 127.85, 126.73, 53.11, 43.68, 39.74, 35.33, 32.73, 28.40, 25.54, 17.78, 16.60.

HRMS (APCI Pos): calculated for $C_{19}H_{25}$ $[M+H]^+$: 253.1951; found: 253.1949.

***cis*-6,7-Diphenylcyclohepta-1,4-diene (3q)**

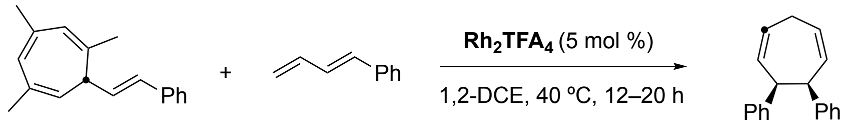

The title compound (viscous colorless oil, 22 mg, 70% yield, >20:1 *cis*/*trans*) was obtained following General Procedure B from (*E*)-1,3,5-trimethyl-7-styrylcyclohepta-1,3,5-triene (30 mg, 0.127 mmol) and (*E*)-buta-1,3-dien-1-ylbenzene (50 mg, 0.381 mmol, 3 equiv) using $[Rh(TFA)_2]_2$ (3.8 mg, 5 mol %) after purification by CombiFlash column chromatography on SiO_2 using cyclohexane as eluent.

^{1}H NMR (500 MHz, Chloroform-*d*) δ 7.20 – 7.10 (m, 6H), 6.91 – 6.79 (m, 4H), 5.91 (dddd, *J* = 10.8, 7.6, 3.1, 1.6 Hz, 2H), 5.76 (ddd, *J* = 11.1, 6.0, 2.9 Hz, 2H), 4.08 (s, 2H), 3.36 (dp, *J* = 19.8, 3.0 Hz, 1H), 2.80 (dt, *J* = 19.8, 7.6 Hz, 1H).

^{13}C NMR (126 MHz, $CDCl_3$) δ 141.91, 133.55, 130.03, 128.44, 127.79, 126.70, 50.64, 28.35.

HRMS (APCI Pos): calculated for $C_{19}H_{19}$ $[M+H]^+$: 247.1481; found: 247.1480.

(±)-(1*S*,4*R*,5*R*)-4-Phenylbicyclo[3.2.1]octa-2,6-diene (3r)

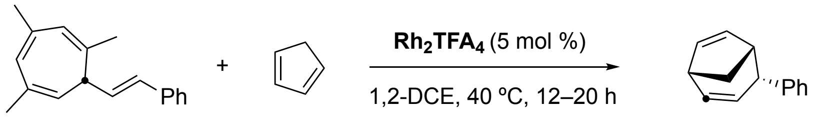

The title compound (colorless oil, 29 mg, 94% yield) was obtained following General Procedure B from (*E*)-1,3,5-trimethyl-7-styrylcyclohepta-1,3,5-triene (40 mg, 0.169 mmol) and freshly cracked/distilled 1,3-cyclopentadiene (45 mg, 0.68 mmol, 4 equiv) using $[Rh(TFA)_2]_2$ (5.0 mg, 5 mol %) after purification by flash column chromatography on SiO_2 using pentane as eluent.

^{1}H NMR (500 MHz, Chloroform-*d*) δ 7.28 – 7.24 (m, 2H), 7.21 – 7.15 (m, 1H), 7.15 – 7.10 (m, 2H), 6.35 (ddd, *J* = 5.6, 2.8, 0.7 Hz, 1H), 6.30 (dddd, *J* = 9.7, 6.3, 2.5, 1.1 Hz, 1H), 5.37 (dddd, *J* = 9.6, 2.6, 1.8, 0.7 Hz, 1H), 5.22 (ddd, *J* = 5.7, 2.8, 0.6 Hz, 1H), 3.69 (dt, *J* = 5.0, 2.6 Hz, 1H), 3.00 (tdd, *J* = 4.8, 2.7, 1.9 Hz, 1H), 2.68 – 2.63 (m, 1H), 2.19 – 2.14 (m, 1H), 2.10 (dt, *J* = 9.5, 0.8 Hz, 1H).

^{13}C NMR (126 MHz, $CDCl_3$) δ 142.28, 141.70, 135.02, 129.95, 128.43, 128.13, 128.08, 126.51, 47.11, 44.69, 44.22, 38.73.

HRMS (APCI Pos): calculated for $C_{14}H_{13}$ $[M-H]^+$: 181.1012; found: 181.1010.

(±)-(1*R*,4*S*,5*S*)-1,5,6,7-Tetramethyl-4-phenylbicyclo[3.2.1]octa-2,6-diene (3s)

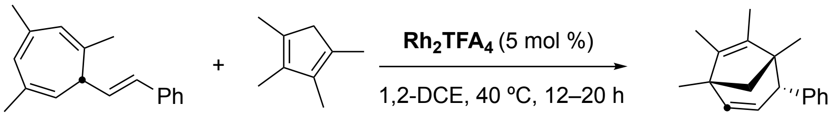

The title compound (colorless oil, 21 mg, 60% yield) was obtained following General Procedure B from (*E*)-1,3,5-trimethyl-7-styrylcyclohepta-1,3,5-triene (35 mg, 0.148 mmol) and 1,2,3,4-tetramethylcyclopenta-1,3-diene (54 mg, 0.44 mmol, 3 equiv) using $[Rh(TFA)_2]_2$ (4.4 mg, 5 mol %) after purification by flash column chromatography on SiO_2 using pentane as eluent.

^{1}H NMR (500 MHz, Chloroform-*d*) δ 7.23 – 7.16 (m, 3H), 7.00 – 6.95 (m, 2H), 6.08 (ddd, *J* = 9.4, 2.5, 1.3 Hz, 1H), 5.39 (dd, *J* = 9.4, 2.6 Hz, 1H), 3.19 (t, *J* = 2.5 Hz, 1H), 1.82 (d, *J* = 9.1 Hz, 1H), 1.61 (q, *J* = 1.2 Hz, 3H), 1.59 (d, *J* = 1.4 Hz, 1H), 1.11 (d, *J* = 4.3 Hz, 6H), 0.82 (q, *J* = 1.2 Hz, 3H).

^{13}C NMR (126 MHz, $CDCl_3$) δ 144.14, 142.05, 139.58, 132.93, 129.95, 128.79, 127.84, 126.59, 58.71, 51.18, 50.35, 45.36, 23.07, 21.74, 12.24, 10.60.

HRMS (APCI Pos): calculated for $C_{18}H_{21}$ $[M-H]^+$: 237.1638; found: 237.1636.

(±)-(1*S*,4*R*,5*R*)-4-Phenylbicyclo[3.3.2]deca-2,9-diene (3t)

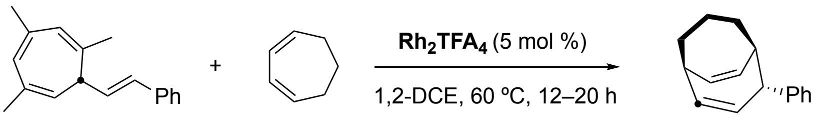

The title compound (colorless oil, 35 mg, 79% yield) was obtained following General Procedure B from (*E*)-1,3,5-trimethyl-7-styrylcyclohepta-1,3,5-triene (50 mg, 0.212 mmol) and 1,3-cycloheptadiene (80 mg, 0.85 mmol, 4 equiv) using $[Rh(TFA)_2]_2$ (6.3 mg, 5 mol %) after purification by flash column chromatography on SiO_2 using pentane as eluent.

^{1}H NMR (500 MHz, Chloroform-*d*) δ 7.28 – 7.23 (m, 4H), 7.18 (ddt, *J* = 6.3, 4.8, 3.0 Hz, 1H), 6.25 – 6.18 (m, 1H), 5.88 – 5.78 (m, 2H), 5.65 – 5.59 (m, 1H), 3.47 (dt, *J* = 4.0, 2.0 Hz, 1H), 2.86 – 2.80 (m, 1H), 2.42 (dddd, *J* = 7.7, 6.5, 2.7, 1.5 Hz, 1H), 2.34 (tdt, *J* = 14.0, 12.8, 4.3 Hz, 1H), 1.92 (ddt, *J* = 13.5, 5.8, 3.9 Hz, 1H), 1.82 (dp, *J* = 14.5, 3.7 Hz, 1H), 1.70 (tdd, *J* = 13.6, 4.2, 2.5 Hz, 1H), 1.61 (tdd, *J* = 12.9, 3.6, 2.0 Hz, 1H), 1.56 – 1.51 (m, 1H).

^{13}C NMR (126 MHz, $CDCl_3$) δ 145.61, 139.59, 135.37, 132.81, 128.95, 128.52, 128.38, 126.39, 48.42, 43.07, 36.81, 33.43, 28.23, 24.14.

HRMS (APCI Pos): calculated for $C_{16}H_{19}$ $[M+H]^+$: 211.1481; found: 211.1479.

6-Phenylcyclohepta-1,4-diene (3u)

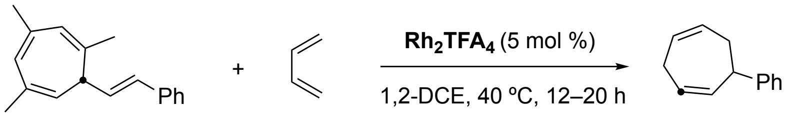

The title compound (colorless oil, 19 mg, 53% yield) was obtained following General Procedure B from (*E*)-1,3,5-trimethyl-7-styrylcyclohepta-1,3,5-triene (50 mg, 0.212 mmol) and 1,3-butadiene (20% w/w solution in PhMe, 0.35 mL, 1.05 mmol, 5 equiv) using $[Rh(TFA)_2]_2$ (6.3 mg, 5 mol %) after purification by flash column chromatography on SiO_2 using pentane as eluent.

^{1}H NMR (400 MHz, Chloroform-*d*) δ 7.36 – 7.27 (m, 4H), 7.26 – 7.20 (m, 1H), 5.92 – 5.68 (m, 4H), 3.76 (ddt, *J* = 10.3, 3.4, 1.9 Hz, 1H), 3.07 – 2.89 (m, 2H), 2.62 (dddt, *J* = 13.5, 10.2, 4.9, 1.5 Hz, 1H), 2.51 – 2.43 (m, 1H).

^{13}C NMR (101 MHz, $CDCl_3$) δ 146.27, 134.92, 129.78, 129.69, 128.37, 127.56, 126.13, 43.96, 35.30, 28.17.

HRMS (APCI Pos): calculated for $C_{13}H_{15}$ $[M+H]^+$: 171.1168; found: 171.1168.

(±)-1-((1*R*,2*R*,5*S*)-Bicyclo[3.2.2]nona-3,6-dien-2-yl)-4-((1*S*,2*S*,5*R*)-bicyclo[3.2.2]nona-3,6-dien-2-yl)benzene (3v)

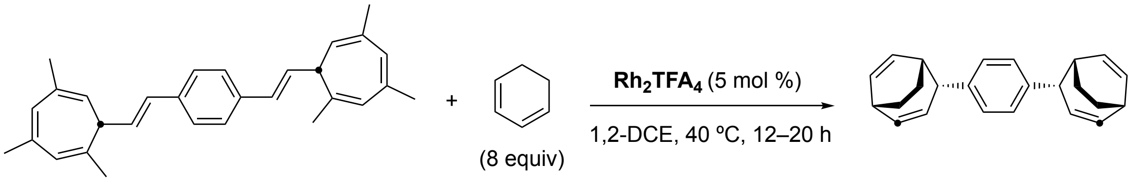

The title compound (white solid, 30 mg, 50% yield, >20:1 dr for the carbon atoms linking to the phenyl ring) was obtained following General Procedure B from 1,4-bis((*E*)-2-(2,4,6-trimethylcyclohepta-2,4,6-trien-1-yl)vinyl)benzene (75 mg, 0.190 mmol) and 1,3-cyclohexadiene (122 mg, 1.52 mmol, 8 equiv) using $[Rh(TFA)_2]_2$ (11.3 mg, 10 mol %) after purification by flash column chromatography on SiO_2 using pentane as eluent.

^{1}H NMR (500 MHz, Chloroform-*d*) δ 7.08 (d, *J* = 1.8 Hz, 4H), 6.42 (ddt, *J* = 8.8, 7.3, 1.3 Hz, 2H), 6.13 (dddd, *J* = 10.8, 8.4, 2.4, 1.2 Hz, 2H), 5.54 (dd, *J* = 8.7, 7.1 Hz, 2H), 5.33 (dddd, *J* = 11.0, 3.6, 1.7, 0.8 Hz, 2H), 3.47 (q, *J* = 3.6 Hz, 2H), 2.72 – 2.64 (m, 4H), 2.10 (ddt, *J* = 12.1, 9.6, 2.5 Hz, 2H), 2.04 – 1.97 (m, 2H), 1.92 (dddd, *J* = 13.5, 11.2, 6.8, 2.9 Hz, 2H), 1.74 – 1.66 (m, 2H).

^{13}C NMR (126 MHz, $CDCl_3$) δ 141.50, 141.48, 138.60, 138.59, 133.80, 131.03, 129.45, 128.18, 51.58, 39.90, 39.87, 33.11, 30.87, 26.58.

HRMS (APCI Pos): calculated for $C_{24}H_{27}$ $[M+H]^+$: 315.2107; found: 315.2109.

MP: 147–149 °C.

***cis*-((*E*)-2-(2,2-dimethyl-3-(2-methylprop-1-en-1-yl)cyclopropyl)vinyl)benzene (3w')**

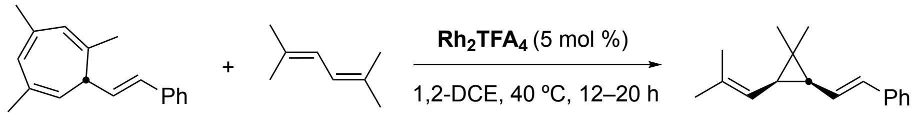

The title compound (colorless oil, 19 mg, 66% yield, >20:1 *cis*/*trans* ratio) was obtained following General Procedure B from (*E*)-1,3,5-trimethyl-7-styrylcyclohepta-1,3,5-triene (30 mg, 0.127 mmol) and 2,5-dimethylhexa-2,4-diene (42 mg, 0.38 mmol, 3 equiv) using $[Rh(TFA)_2]_2$ (3.8 mg, 5 mol%) after purification by flash column chromatography on SiO_2 using pentane as eluent.

^{1}H NMR (400 MHz, Chloroform-*d*) δ 7.36 – 7.28 (m, 4H), 7.22 – 7.16 (m, 1H), 6.54 (d, *J* = 15.7 Hz, 1H), 6.10 – 5.99 (m, 1H), 5.11 (dtd, *J* = 6.6, 2.8, 1.4 Hz, 1H), 1.79 – 1.77 (m, 3H), 1.72 (d, *J* = 1.3 Hz, 3H), 1.67 – 1.63 (m, 2H), 1.21 (s, 3H), 1.13 (s, 3H).

^{13}C NMR (101 MHz, $CDCl_3$) δ 138.17, 135.24, 130.40, 128.61, 128.45, 126.47, 125.66, 119.58, 33.48, 30.43, 28.73, 25.74, 24.03, 18.57, 16.41.

HRMS (APCI Pos): calculated for $C_{17}H_{23}$ $[M+H]^+$: 227.1794; found: 227.1795.

(*endo*,*Z*)-9-((*E*)-Styryl)bicyclo[6.1.0]non-2-ene (3x')

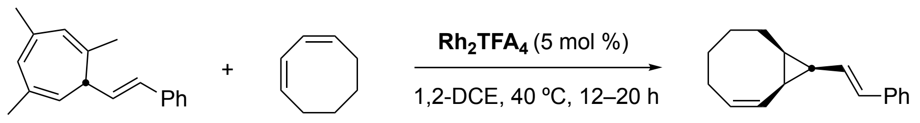

The title compound (colorless oil, 15 mg, 53% yield, >20:1 *cis*/*trans* ratio) was obtained following General Procedure B from (*E*)-1,3,5-trimethyl-7-styrylcyclohepta-1,3,5-triene (30 mg, 0.127 mmol) and (1*Z*,3*Z*)-1,3-cyclooctadiene (55 mg, 0.51 mmol, 4 equiv) using $[Rh(TFA)_2]_2$ (3.8 mg, 5 mol%) after purification by flash column chromatography on SiO_2 using pentane as eluent.

^{1}H NMR (400 MHz, Chloroform-*d*) δ 7.36 – 7.27 (m, 4H), 7.22 – 7.16 (m, 1H), 6.57 (d, *J* = 15.7 Hz, 1H), 5.95 (dd, *J* = 15.8, 9.8 Hz, 1H), 5.85 (dddd, *J* = 11.4, 6.9, 4.8, 2.4 Hz, 1H), 5.46 – 5.39 (m, 1H), 2.49 (dddt, *J* = 16.2, 8.9, 7.0, 1.8 Hz, 1H), 2.13 – 1.97 (m, 2H), 1.89 – 1.77 (m, 3H), 1.70 – 1.60 (m, 1H), 1.55 – 1.46 (m, 1H), 1.42 – 1.32 (m, 1H), 1.29 – 1.19 (m, 1H).

GCMS (EI): calculated for $C_{17}H_{20}$ $[M]^+$: 223.1; found: 223.2.

(±)-(1*R*,6*S*,9*R*,*Z*)-9-Phenylbicyclo[4.3.2]undeca-7,10-diene (3x)

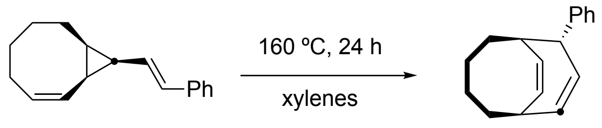

The title compound (pale yellow oil, 13 mg, 90% yield) was obtained by dissolving **7x'** (15 mg, 0.127 mmol) in xylenes and heating at 160 °C for 24 h, after purification by flash column chromatography on SiO_2 using pentane as eluent. This can also be performed as a one pot procedure, swaping the solvent of the cyclopropanation from 1,2-DCE to xylenes, and heating the crude mixture at 160 °C for 24 h.

^{1}H NMR (500 MHz, Chloroform-*d*) δ 7.31 – 7.28 (m, 2H), 7.26 – 7.22 (m, 2H), 7.18 – 7.13 (m, 1H), 5.77 (ddd, *J* = 11.3, 7.9, 1.3 Hz, 1H), 5.71 (ddt, *J* = 12.1, 6.5, 1.2 Hz, 1H), 5.66 – 5.58 (m, 2H), 3.50 (d, *J* = 6.2 Hz, 1H), 3.10 (dtdt, *J* = 7.9, 6.1, 2.7, 1.4 Hz, 1H), 2.64 (dt, *J* = 8.4, 4.0 Hz, 1H), 2.14 – 2.05 (m, 1H), 1.93 – 1.89 (m, 2H), 1.81 – 1.65 (m, 4H), 1.59 – 1.53 (m, 1H).

^{13}C NMR (126 MHz, $CDCl_3$) δ 145.91, 134.36, 132.84, 131.85, 130.09, 129.05, 128.17, 126.27, 51.45, 44.96, 40.43, 39.27, 33.48, 27.57, 24.52.

HRMS (APCI Pos): calculated for $C_{17}H_{19}$ $[M\text{-}H]^+$: 223.1481; found: 223.1482.

4-((*E*)-2-((*endo*,*Z*)-Bicyclo[6.1.0]non-2-en-9-yl)vinyl)phenyl 1-ferrocenoylate (3y')

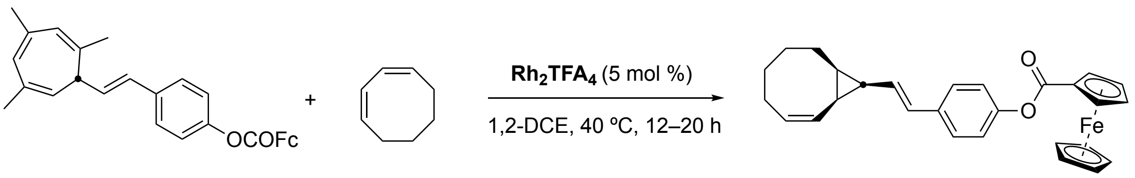

The title compound (pale yellow oil, 22 mg, 50% yield, >20:1 *cis*/*trans* ratio) was obtained following General Procedure B from (*E*)-4-(2-(2,4,6-trimethylcyclohepta-2,4,6-trien-1-yl)vinyl)phenyl ferrocenoylate (45 mg, 0.097 mmol) and (1*Z*,3*Z*)-1,3-cyclooctadiene (42 mg, 0.39 mmol, 4 equiv) using $[Rh(TFA)_2]_2$ (2.9 mg, 5 mol%) after purification by flash column chromatography on SiO_2 using pentane as eluent.

^{1}H NMR (400 MHz, Chloroform-*d*) δ 7.41 – 7.33 (m, 2H), 7.14 – 7.08 (m, 2H), 6.57 (d, *J* = 15.8 Hz, 1H), 5.96 – 5.82 (m, 2H), 5.47 – 5.42 (m, 1H), 4.98 (t, *J* = 2.0 Hz, 2H), 4.53 – 4.50 (m, 2H), 4.32 (s, 5H), 2.49 (ddd, *J* = 16.2, 8.6, 6.6 Hz, 1H), 2.12 – 1.99 (m, 2H), 1.92 – 1.75 (m, 4H), 1.68 (dd, *J* = 11.5, 7.7 Hz, 2H), 1.55 – 1.46 (m, 1H), 1.41 – 1.34 (m, 1H), 1.26 – 1.19 (m, 1H).

^{13}C NMR (101 MHz, $CDCl_3$) δ 170.36, 149.47, 136.29, 135.76, 129.48, 128.79, 126.48, 123.32, 121.65, 71.89, 70.65, 70.19, 69.95, 30.95, 30.03, 25.17, 25.13, 23.05, 22.97, 21.71.

(±)-4-((1*S*,6*R*,7*R*,*Z*)-Bicyclo[4.3.2]undeca-8,10-dien-7-yl)phenyl ferrocenoylate (3y)

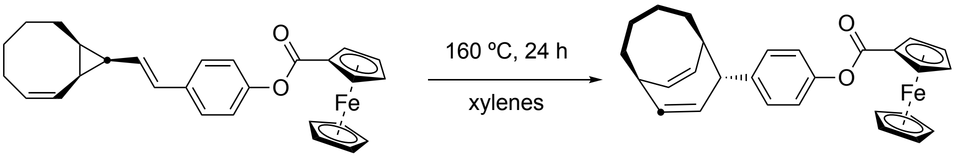

The title compound (pale yellow oil, 9 mg, 45% yield) was obtained by dissolving **7y'** (22 mg, 0.049 mmol) in xylenes and heating at 160 ºC for 24 h, after purification by preparative TLC on SiO_2 using pentane as eluent.

^{1}H NMR (500 MHz, Chloroform-*d*) δ 7.35 – 7.30 (m, 2H), 7.08 – 7.03 (m, 2H), 5.81 – 5.75 (m, 1H), 5.74 – 5.69 (m, 1H), 5.67 – 5.58 (m, 2H), 4.94 (dt, *J* = 3.9, 1.9 Hz, 2H), 4.47 (dt, *J* = 6.7, 2.0 Hz, 2H), 4.28 (d, *J* = 4.1 Hz, 5H), 3.52 (d, *J* = 6.0 Hz, 1H), 3.11 (d, *J* = 5.6 Hz, 1H), 2.65 (s, 1H), 2.09 (ddt, *J* = 14.2, 8.9, 4.8 Hz, 1H), 1.93 (ddd, *J* = 7.2, 5.8, 3.2 Hz, 2H), 1.79 – 1.66 (m, 4H), 1.58 (dd, *J* = 9.9, 4.9 Hz, 1H).

^{13}C NMR (126 MHz, $CDCl_3$) δ 170.68, 149.47, 143.16, 134.44, 132.92, 131.82, 130.05, 129.97, 121.19, 72.16, 70.99, 70.74, 70.28, 50.89, 44.98, 40.46, 39.19, 33.46, 27.57, 24.53.

HRMS (ESI Pos): calculated for $C_{28}H_{28}NaO_2{}^{56}Fe$ $[M+Na]^+$: 475.1331; found 475.1329.

MP: 130–135 ºC.

7. General Procedure C: Gold(I)-Catalyzed Cycloisomerization–Migration–(4+3) Cycloaddition

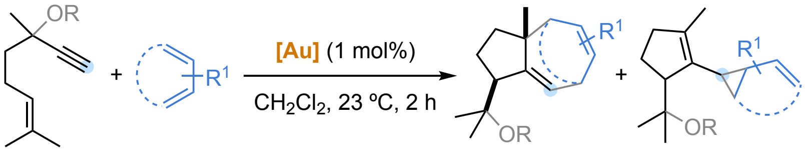

6 **2** (3 equiv) **7** **8**

A solution of enyne **6a** or **6'** (1 equiv) and diene **2** (3 equiv) in CH_2Cl_2 (0.2 M) was placed in a 2 or 5 mL screw-cap vial. Au(I) catalyst (1 mol %) was added in one portion and the mixture was stirred at room temperature for 2 h. The reaction was monitored by TLC or GC-MS and after total consumption of the starting material, it was quenched by adding one drop of triethylamine. The solvent was evaporated at low pressure and the crude material was purified by silica gel column chromatography to give **7**, **8** or a mixture of both.

7.1. Characterization Data for the Different Cycloheptadienes (7) and Cyclopropanes (8)

(±)-(1*S*,3*R*)-3-Methyl-1-(2-(4-nitrophenoxy)propan-2-yl)-1,2,3,3,4,7-hexahydro-4,7-methanoazulene (7a)

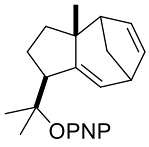

Prepared following general procedure C, by reaction of **6** (27.3 mg, 0.10 mmol, 1 equiv.) with **2a** (19.8 mg, 0.30 mmol, 3 equiv.) using catalyst **D**. Column chromatography (silica gel, 100% cyclohexane to 100:1 cyclohexane/EtOAc) delivered the title compound together with traces compound **8a** as a pale-yellow oil (21.7 mg, 4.8:1 dr, 64% yield).

Some ^{1}H and ^{13}C NMR signals of the minor isomer could be assigned.

^{1}H NMR (400 MHz, $CDCl_3$) δ 8.19 – 8.07 (m, 2H, *overlapping with minor*), 7.09 – 7.00 (m, 2H, *overlapping with minor*), 6.33 (ddd, J = 5.6, 2.7, 0.8 Hz, 1H), 6.19 (dd, J = 5.7, 3.1 Hz, *1H minor isomer*), 5.97 (dd, J = 5.7, 2.7 Hz, *1H minor isomer*), 5.89 (dt, J = 5.4, 1.6 Hz, 1H), 5.66 (dd, J = 5.6, 3.1 Hz, 1H), 2.82 – 2.76 (m, *1H minor isomer*), 2.74 (dd, J = 5.1, 3.1 Hz, 1H), 2.73 – 2.65 (m, 2H), 2.52 (t, J = 3.5 Hz, *1H minor isomer*), 2.03 (d, J = 9.9 Hz, 1H), 1.92 – 1.82 (m, 1H), 1.82 – 1.76 (m, 1H), 1.72 (dddd, J = 12.8, 8.7, 6.9, 1.0 Hz, 1H), 1.39 (s, 3H), 1.38 (s, 3H), 1.37 (s, *3H minor isomer*), 1.36 (s, *1H minor isomer*), 1.33 (d, J = 0.7 Hz, 3H), 1.29 – 1.24 (m, 1H), 1.21 (dd, J = 12.3, 7.0 Hz, 1H), 1.08 (s, *3H minor isomer*).

^{13}C NMR (101 MHz, $CDCl_3$) δ 162.5, 142.8, 142.5 (*minor*), 141.8, 138.3, 135.2 (*minor*), 130.4 (*minor*), 130.3, 129.9, 125.3, 125.3 (*minor*), 122.1 (*minor*), 122.1, 8m4.6, 84.2 (*minor*), 54.9 (*minor*), 54.1, 50.3, 46.5 (*minor*), 45.4, 42.9 (*minor*), 41.1 (*minor*), 40.2, 39.4, 37.5 (*minor*), 32.4, 27.6, 25.7, 25.7, 25.6 (*minor*), 25.5, 24.2 (*minor*), 22.5 (*minor*).

HRMS (ESI+): m/z calc. for $[C_{21}H_{25}NNaO_3]^+$: 362.1727, found: 362.1730.

Analogous reaction with catalyst **A** delivered product **7a** as a 1:2 mixture with **8a**, 26% (7:1) and 51% (12:1) calculated yields and *dr* respectively. Some ^{1}H NMR signals of both isomers of **8a** could be assigned.

^{1}H NMR (500 MHz, $CDCl_3$ δ 8.19 – 8.07 (m, 2H, *all isomers overlapping*), 7.09 – 7.00 (m, 2H, *all isomers overlapping*), 6.33 (ddd, J = 5.6, 2.7, 0.8 Hz, 1H **7a** *major*), 6.19 (dd, J = 5.7, 3.1 Hz, *1H **7a** minor*), 5.97 (dd, J = 5.7, 2.7 Hz, *1H **7a** minor*), 5.89 (dt, J = 5.4, 1.6 Hz, 1H **7a** *major*), 5.66 (dd, J = 5.6, 3.1 Hz, 1H **7a** *major*), 5.64 – 5.60 (m, *1H **8a** minor*), 5.57 (ddd, J = 5.6, 2.3, 1.1 Hz, 1H **8a** *major*), 5.37 (d, J = 5.3 Hz, *1H **8a** minor*), 5.34 (ddd, J = 5.3, 2.9, 1.8 Hz, 1H **8a** *major*), 2.86 (dq, J = 9.3, 1.4 Hz, , 1H **8a** *major*), 2.81 – 2.75 (m, *1H **7a** minor*), 2.74 (dd, J = 5.1, 3.1 Hz, 1H **7a** *major*), 2.72 – 2.63 (m, 2H **7a** *major*), 2.53 – 2.50 (m, *1H **7a** minor*), 2.48 – 2.38 (m, 1H **8a** *major*), 2.38 – 2.23 (m, 1H **8a** *major and* minor), 2.13 (dd, *J* = 16.6, 9.7 Hz, 1H **8a** *major*), 2.05 – 1.97 (m, 2H **8a** *major*), 1.93 – 1.89 (m, 2H, **8a** *major* and overlapping), 1.89 – 1.84 (m, *all isomers*

overlapping), 1.82 – 1.77 (m, *all isomers overlapping*), 1.75 (t, J = 1.4 Hz, 3H, **8a** *major*), 1.47 (s, 3H **8a** *major*), 1.39 (s, 3H **7a** *major*), 1.38 (s, 3H **7a** *major*), 1.37 (s, 3H **8a** *major*), 1.32 (d, J = 0.7 Hz, 3H **7a** *major*), 1.29 – 1.24 (m, 2H, *overlapping signals*), 1.21 (dd, J = 12.4, 6.9 Hz, 3H **7a** *major*), 1.08 (s, *3H 7a minor*).

^{13}C NMR (126 MHz, $CDCl_3$) δ 162.7, 162.5, 143.4, 142.8, 142.5, 141.9, 141.8, 138.3, 135.2, 130.3, 130.2, 130.1, 129.9, 129.8, 129.4, 129.4, 129.2, 126.0, 125.4, 125.3, 125.3, 125.2, 124.7, 122.1, 122.1, 121.7, 121.7, 121.0, 120.8, 120.4, 117.6, 87.3, 84.5, 58.3, 56.4, 54.9, 54.1, 50.3, 46.5, 45.4, 44.1, 42.9, 41.0, 40.1, 39.4, 37.7, 37.5, 37.2, 32.8, 32.3, 31.3, 29.3, 27.6, 27.1, 25.9, 25.7, 25.6, 25.6, 25.5, 25.4, 25.3, 24.9, 24.4, 24.2, 24.2, 22.8, 22.5, 21.9, 21.7, 20.1, 15.3, 15.2.

(±)-(1*S*,3*S*)-3,4,5,6,7-Pentamethyl-1-(2-(4-nitrophenoxy)propan-2-yl)-1,2,3,3,4,7-hexahydro-4,7-methanoazulene (7b)

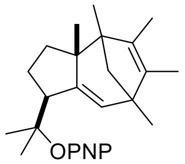

Prepared following general procedure C, by reaction of **6** (55.7 mg, 0.20 mmol, 1 equiv.) with **2d** (73.3 mg, 0.60 mmol, 3 equiv.) using catalyst **A**. Column chromatography (silica gel, 100% cyclohexane to 100:1 cyclohexane/EtOAc) delivered the title compound as a pale-yellow oil (65.4 mg, 81% yield, 6.4:1 dr).

Some ^{1}H NMR signals of the minor isomer could be assigned.

^{1}H NMR (500 MHz, $CDCl_3$) δ 8.18 – 8.11 (m, 2H), 7.10 – 7.03 (m, 2H), 5.79 (t, J = 1.7 Hz, 1H), 5.62 (d, J = 1.7 Hz, *1H minor isomer*), 2.80 (t, J = 9.4 Hz, *1H minor isomer*), 2.63 (t, J = 9.5 Hz, 1H), 1.93 (d, J = 9.6 Hz, 1H), 1.81 – 1.72 (m, 2H), 1.73 – 1.66 (m, 2H), 1.60 (d, J = 1.2 Hz, *3H minor isomer*), 1.57 (q, J = 1.2 Hz, 3H), 1.54 (d, J = 1.2 Hz, *3H minor isomer*), 1.53 (q, J = 1.3 Hz, 3H), 1.41 (s, 3H), 1.37 (d, J = 2.6 Hz, 3H), 1.25 (dd, J = 9.6, 1.6 Hz, 1H), 1.21 (d, J = 0.7 Hz, 3H), 1.13 (s, 3H), 1.06 (s, 3H), 1.04 (s, *3H minor isomer*), 0.94 (s, *3H minor isomer*).

^{13}C NMR (126 MHz, $CDCl_3$) δ 162.5, 147.0, 142.9, 136.5, 132.5, 125.3, 125.2, 122.3, 84.6, 53.7, 52.4, 51.5, 48.2, 46.9, 32.9, 25.9, 25.6, 24.9, 21.6, 21.0, 19.9, 12.7, 10.7.

HRMS (ESI+): m/z calc. for $[C_{25}H_{33}NNaO_3]^+$: 418.2353, found: 418.2341.

Analogous reaction with catalyst **D** delivered product **7b** in 93% yield and 1.5:1 dr.

(±)-(1*S*,3*S*)-3,4,5,6,7,9-Hexamethyl-1-(2-(4-nitrophenoxy)propan-2-yl)-1,2,3,3,4,7-hexahydro-4,7-methanoazulene (7c)

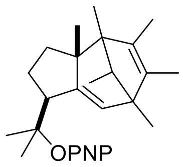

Prepared following general procedure C, by reaction of **6** (54.7 mg, 0.20 mmol, 1 equiv.) with **2e** (82.0 mg, 0.60 mmol, 3 equiv.) using catalyst **A**. Column chromatography (silica gel, 100% cyclohexane to 100:1 cyclohexane/EtOAc) delivered the title compound as a pale-yellow oil (55.8 mg, 68% yield, 9:1 dr). Some ^{1}H NMR signals of the minor isomer could be assigned.

^{1}H NMR (500 MHz, $CDCl_3$) δ 8.18 – 8.11 (m, 2H), 7.08 – 7.02 (m, 2H), 5.79 (d, J = 1.8 Hz, 1H), 5.65 (d, J = 1.7 Hz, *1H minor isomer*), 2.80 (t, J = 9.4 Hz, *1H minor isomer*), 2.64 (t, J = 9.3 Hz, 1H), 2.16 (q, J = 6.7 Hz, 1H), 1.82 – 1.72 (m, 2H), 1.72 – 1.64 (m, 2H), 1.55 (q, J = 1.2 Hz, 3H), 1.50 (t, J = 1.2 Hz, 3H), 1.41 (s, 3H), 1.37 (s, 3H), 1.19 (d, J = 0.8 Hz, 3H), 1.00 (s, 3H), 0.94 (d, J = 1.9 Hz, 3H), 0.88 (s, *3H minor isomer*), 0.67 (d, J = 6.7 Hz, 3H).

^{13}C NMR (126 MHz, $CDCl_3$) δ 162.6, 143.6, 142.6, 142.1, 138.8, 128.7, 125.3, 122.3, 84.6, 54.8, 53.9, 50.5, 49.7, 49.7, 33.1, 25.9, 25.7, 24.7, 21.5, 18.2, 15.9, 12.9, 11.2, 11.0.

HRMS (ESI+): m/z calc. for $[C_{26}H_{35}NNaO_3]^+$: 432.2509, found: 432.2505.

7-(2-Methyl-5-(2-(4-nitrophenoxy)propan-2-yl)cyclopent-1-en-1-yl)bicyclo[4.1.0]hept-2-ene (8d)

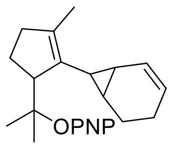

Prepared following general procedure C, by reaction of **6** (54.7 mg, 0.20 mmol, 1 equiv.) with **2b** (48.1 mg, 0.60 mmol, 3 equiv.) using catalyst **A**. Column chromatography (silica gel, 100% cyclohexane to 100:1 cyclohexane/EtOAc) delivered the title compound as a colorless oil (58.7 mg, 83% yield, 3:1 dr, *endo*/*exo* cyclopropane isomers unassigned). Some ^{1}H NMR signals of the minor isomer could be assigned.

^{1}H NMR (400 MHz, $CDCl_3$) δ 8.30 – 8.00 (m, 2H, both isomers), 7.12 – 6.92 (m, 2H, both isomers), 5.97 – 5.92 (m, *1H minor isomer*), 5.92 – 5.85 (m, 1H), 5.56 (ddd, J = 10.0, 6.0, 2.3 Hz, 1H), 5.47 (ddd, J = 9.1, 6.3, 2.1 Hz, *1H minor isomer*), 3.38 (d, J = 9.4 Hz, 1H), 3.28 (d, J = 9.3 Hz, *1H minor isomer*), 2.30 (q, J = 9.0, 8.1 Hz, 2H), 2.14 – 2.02 (m, 2H), 1.97 – 1.86 (m, 3H), 1.82 (t, J = 1.3 Hz, 3H), 1.79 – 1.73 (m, 1H), 1.72 – 1.65 (m, 1H), 1.64 – 1.57 (m, 2H), 1.46 (s, 3H), 1.41 (s, *3H minor isomer*), 1.34 (s, 3H), 1.25 (s, 3*H minor isomer*).

^{13}C NMR (101 MHz, $CDCl_3$) δ 162.6, 141.3, 133.8, 126.5, 126.0, 125.4, 125.3, 121.0, 87.3, 56.5, 37.3, 25.8, 25.7, 24.9, 24.3, 22.9, 19.5, 18.4, 15.5, 14.0.

HRMS (ESI+): m/z calc. for $[C_{22}H_{27}NNaO_3]^+$: 376.1883, found: 376.1877.

Analogous reaction with catalyst **D** gave a complex mixture of products, including **8d** and many not characterized side-products.

(±)-(1*S*,3*R*)-3-Methyl-1-(2-(4-nitrophenoxy)propan-2-yl)-1,2,3,3,4,7-hexahydroazulene (7e)

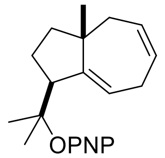

Prepared following general procedure C, by reaction of **6** (54.7 mg, 0.20 mmol, 1 equiv.) with **2c** (162 mg, 20% in hexane, 0.60 mmol, 3 equiv.) using catalyst **A**. Column chromatography (silica gel, 100% cyclohexane to 100:1 cyclohexane/EtOAc) delivered the title compound together with compound **8e** as a colorless oil (34.1 mg, 4.7:1 ratio, 51% overall yield: 42% + 9% calculated yields).

Some ^{1}H and ^{13}C NMR signals of **8e** could be assigned.

^{1}H NMR (500 MHz, $CDCl_3$) δ 8.21 – 8.08 (m, 2H *overlapping with **8e***), 7.08 – 7.01 (m, 2H *overlapping with **8e***), 5.85 (dtd, J = 5.9, 2.3, 1.1 Hz, 1H), 5.68 – 5.59 (m, 1H), 5.55 (dtdd, J = 11.6, 4.3, 2.9, 1.1 Hz, 1H), 5.32 (ddd, J = 17.1, 10.3, 8.3 Hz, *1H **8e***), 5.00 (dd, J = 17.1, 1.7 Hz, *1H **8e***), 4.80 (dd, J = 10.3, 1.7 Hz, *1H **8e***), 3.19 – 3.11 (m, 1H), 3.08 – 2.97 (m, 1H), 2.94 – 2.83 (m, 1H), 2.34 (dt, J = 15.7, 2.9 Hz, 1H), 2.08 (dddd, J = 15.7, 7.9, 1.9, 0.8 Hz, 1H), 1.81 – 1.75 (m, 1H), 1.75 – 1.74 (m, *3H **8e***), 1.60 – 1.55 (m, 1H), 1.56 – 1.53 (m, 1H), 1.50 – 1.44 (m, 1H), 1.43 (s, *3H **8e***), 1.41 (s, 3H), 1.37 (s, 3H), 1.34 (s, *3H **8e***), 1.13 (s, 3H), 1.00 – 0.91 (m, *2H **8e***), 0.85 (ddt, J = 10.7, 4.3, 2.2 Hz, *2H **8e***).

^{13}C NMR (126 MHz, $CDCl_3$) δ 162.4 (***8e***), 161.8, 149.9, 142.6, 141.8 (***8e***), 140.7 (***8e***), 134.5 (***8e***), 127.3, 127.3 (***8e***), 125.4, 125.3 (***8e***), 122.2, 121.5, 121.2, 112.0 (***8e***), 86.9 (***8e***), 86.0, 58.6 (***8e***), 54.6, 46.9, 41.6, 41.5, 37.8 (***8e***), 30.7, 27.3, 26.4, 25.9 (***8e***), 25.5 (***8e***), 24.2 (***8e***), 24.1, 23.1, 22.4 (***8e***), 20.6 (***8e***), 17.3 (***8e***), 15.1 (***8e***).

HRMS (ESI+): m/z calc. for $[C_{20}H_{25}NNaO_3]^+$: 350.1727, found: 350.1721.

Analogous reaction with catalyst **D** delivered a mixture of **7e** and **8e** in 28% and 24% calculated yields.

(±)-(1*S*,3*R*)-3,6-Dimethyl-1-(2-(4-nitrophenoxy)propan-2-yl)-1,2,3,3,4,7-hexahydroazulene (7f)

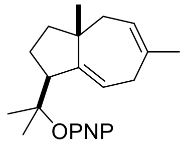

Prepared following general procedure C, by reaction of **6** (137 mg, 0.50 mmol, 1 equiv.) with **2f** (102 mg, 1.50 mmol, 3 equiv.) using catalyst **A**. Column chromatography (silica gel, 100% cyclohexane to 100:1 cyclohexane/EtOAc) delivered the title compound together with compound **8f** as a colorless oil (131.5 mg, 1.2:1 ratio, 77% overall yield: 42% + 35% calculated yields).

^{1}H NMR signals of both compounds could be partially assigned by analogy.

^{1}H NMR (400 MHz, $CDCl_3$) δ 8.22 – 8.02 (m, 4H, **7f** + **8f**), 7.11 – 7.05 (m, 2H), 7.03 – 6.97 (m, 2H), 5.77 (ddd, J = 5.4, 4.4, 2.2 Hz, 1H, **7f**), 5.46 – 5.41 (m, 1H, **7f**), 5.31 (dd, J = 17.2, 10.6 Hz, 1H, **8f**), 4.85 (dd, J = 17.2, 1.3 Hz, 1H, **8f**), 4.75 (dd, J = 10.5, 1.3 Hz, 1H, **8f**), 3.23 – 3.04 (m, 2H), 2.90 – 2.82 (m, 2H), 2.38 – 2.10 (m, 4H), 2.01 (dd, J = 15.3,

8.3 Hz, 1H), 1.96 – 1.78 (m, 3H), 1.76 (t, J = 1.4 Hz, 3H), 1.70 (t, J = 1.8 Hz, 3H), 1.59 – 1.50 (m, 3H), 1.40 (s, 6H), 1.37 (s, 3H), 1.36 (s, 3H), 1.10 (s, 3H), 1.09 (s, 3H), 1.05 (dd, J = 8.6, 4.4 Hz, 1H, **8f**), 0.71 (dd, J = 6.6, 4.4 Hz, 1H, **8f**).

^{13}C NMR (101 MHz, $CDCl_3$) δ 162.4, 161.9, 149.8, 147.0, 142.6, 141.9, 141.2, 134.1, 133.1, 125.3, 122.2, 122.1, 122.1, 120.9, 120.5, 109.6, 86.7, 86.0, 58.8, 54.6, 46.3, 41.4, 41.1, 37.7, 35.5, 26.9, 26.5, 26.4, 26.3, 25.4, 25.3, 24.5, 24.0, 23.2, 22.9, 22.5, 16.6, 15.4.

HRMS (ESI+): m/z calc. for $[C_{21}H_{27}NNaO_3]^+$: 364.1883, found: 364.1877.

Analogous reaction with catalyst **D** delivered a mixture of **7f** and **8f** in 42% and 32% calculated yields.

(±)-(1*S*,3*R*)-3-Methyl-1-(2-(4-nitrophenoxy)propan-2-yl)-6-phenyl-1,2,3,3,4,7-hexahydroazulene (7g) and (8g)

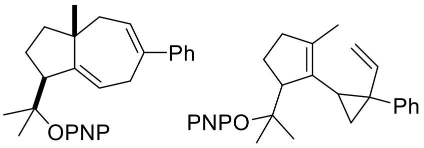

Prepared following general procedure C, by reaction of **6** (27.3 mg, 0.10 mmol, 1 equiv.) with **2h** (39.1 mg, 0.30 mmol, 3 equiv.) using catalyst **A**. Column chromatography (silica gel, 100% cyclohexane to 100:1 cyclohexane/EtOAc) delivered a mixture of **7g** and **8g** as a colorless oil (28.0 mg, 1:1.2 ratio, 69% overall yield: 31% + 38% calculated yields).

^{1}H NMR signals of both compounds could be partially assigned by analogy.

^{1}H NMR (500 MHz, $CDCl_3$) δ 8.19 – 8.14 (m, 3H), 8.14 – 8.08 (m, 2H), 7.36 – 7.28 (m, 6H), 7.25 – 7.17 (m, 6H), 7.13 – 7.05 (m, 2H), 6.93 – 6.86 (m, 2H), 6.10 (dd, J = 17.2, 10.5 Hz, 1H, **8g**), 6.00 (ddt, J = 8.5, 4.3, 1.3 Hz, 1H, **7g**), 5.87 (td, J = 5.0, 2.2 Hz, 1H, **7g**), 4.93 (d, J = 1.2 Hz, 1H, **8g**), 4.90 (dd, J = 17.2, 1.2 Hz, 1H, **8g**), 3.43 – 3.30 (m, 2H, **7g**), 3.19 (tt, J = 7.9, 2.6 Hz, 1H), 3.14 (s, 0.5H), 2.49 (ddd, J = 14.8, 1.8 Hz, 1H), 2.38 (d, J = 9.4 Hz, 1H), 2.28 (dd, J = 15.0, 8.5 Hz, 1H), 2.23 – 2.14 (m, 0H), 2.11 – 2.01 (m, 1H), 1.98 – 1.92 (m, 1H), 1.91 (t, J = 1.5 Hz, 1H), 1.86 – 1.80 (m, 1H), 1.78 (t, J = 1.4 Hz, 3H, **8g**), 1.76 (dd, J = 6.8, 5.0 Hz, 1H), 1.70 – 1.67 (m, 1H), 1.61 – 1.58 (m, 1H), 1.57 – 1.49 (m, 2H), 1.41 (s, 3H), 1.39 (s, 3H), 1.37 (s, 3H), 1.28 (s, 3H), 1.27 – 1.25 (m, 3H), 1.18 (s, 3H).

^{13}C NMR (126 MHz, $CDCl_3$) δ 162.2, 161.9, 149.8, 145.3, 144.1, 142.6, 141.7, 140.0, 139.4, 132.1, 128.4, 128.3, 128.3, 128.3, 127.8, 127.6, 127.6, 127.1, 126.7, 126.7, 126.3, 126.1, 126.0, 125.6, 125.4, 125.3, 122.2, 122.2, 121.3, 120.8, 120.1, 112.2, 87.0, 85.9, 56.9, 54.9, 46.1, 41.3, 37.7, 37.5, 33.8, 32.3, 29.8, 29.0, 26.4, 26.2, 25.5, 25.1, 25.1, 25.1, 24.8, 24.5, 23.5, 21.6, 21.6, 21.2, 15.5, 15.5, 15.3.

HRMS (ESI+): m/z calc. for $[C_{26}H_{29}NNaO_3]^+$: 426.2040, found: 426.2032.

(±)-2-((1*S*,3*R*)-3-Methyl-6-((triisopropylsilyl)oxy)-1,2,3,3,4,7-hexahydroazulen-1-yl)propan-2-yl acetate (7h')

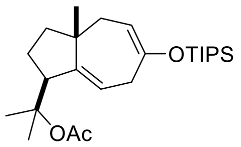

Prepared following general procedure C, by reaction of **6'** (9.7 mg, 0.050 mmol, 1 equiv.) with **2j** (34.0 mg, 0.15 mmol, 3 equiv.) using catalyst **D**. Column chromatography (silica gel, 100% cyclohexane to 100:1 cyclohexane/EtOAc) delivered the title compound as a colorless oil (9.5 mg, 45% yield).

^{1}H NMR (500 MHz, CD_2Cl_2) δ 5.48 (ddd, J = 6.1, 4.1, 2.3 Hz, 1H), 4.95 (ddd, J = 8.9, 3.5, 1.5 Hz, 1H), 3.25 (t, J = 8.4 Hz, 1H), 3.10 – 2.91 (m, 2H), 2.24 – 2.16 (m, 1H), 1.94 (s, 3H), 1.89 (dd, J = 15.1, 8.9 Hz, 1H), 1.72 – 1.64 (m, 2H), 1.51 – 1.48 (m, 2H), 1.47 (s, 3H), 1.40 (s, 3H), 1.20 – 1.12 (m, 3H), 1.10 – 1.07 (m, 18H), 0.08 (s, 3H).

^{13}C NMR (126 MHz, $CDCl_3$) δ 170.6, 150.1, 149.9, 117.6, 104.3, 85.6, 53.5, 46.3, 41.3, 38.2, 35.9, 26.0, 25.4, 23.9, 22.9, 22.7, 18.2, 12.8.

HRMS (ESI+): m/z calc. for $[C_{25}H_{44}NaO_3Si]^+$: 443.2952, found: 443.2956.

(±)-Triisopropyl(((1*S*,3*R*)-3-methyl-1-(2-(4-nitrophenoxy)propan-2-yl)-1,2,3,3,4,7-hexahydroazulen-6-yl)oxy)silane (7h)

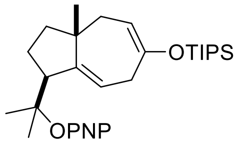

Prepared following general procedure C, by reaction of **6** (13.7 mg, 0.050 mmol, 1 equiv.) with **2j** (34.0 mg, 0.15 mmol, 3 equiv.) using catalyst **D**. Column chromatography (silica gel, 100% cyclohexane to 100:1 cyclohexane/EtOAc) delivered the title compound as a pale yellow oil (18.0 mg, 72% yield).

^{1}H NMR (400 MHz, CD_2Cl_2) δ 8.20 – 8.09 (m, 2H), 7.14 – 7.05 (m, 2H), 5.75 (ddd, J = 5.5, 4.4, 2.3 Hz, 1H), 4.97 (dd, J = 8.9, 3.5 Hz, 1H), 3.19 – 3.09 (m, 1H), 3.10 – 2.92 (m, 2H), 2.23 (dq, J = 15.0, 3.0 Hz, 1H), 1.93 (ddt, J = 15.2, 9.0, 1.0 Hz, 1H), 1.82 – 1.71 (m, 1H), 1.59 – 1.40 (m, 3H (overlap with water peak)), 1.39 (s, 3H), 1.37 (s, 3H), 1.22 – 1.13 (m, 3H), 1.12 (s, 3H), 1.11 – 1.05 (m, 18H).

^{13}C NMR (101 MHz, CD_2Cl_2) δ 162.2, 150.3, 150.2, 125.5, 122.5, 118.7, 104.6, 86.3, 55.0, 46.5, 41.6, 38.5, 36.1, 26.8, 26.4, 24.1, 23.1, 18.3, 18.3, 13.1.

HRMS (ESI+): m/z calc. for $[C_{29}H_{45}NNaO_4Si]^+$: 522.3010, found: 522.3012.

Analogous reaction with catalyst **A** delivered a mixture of **7h** and **9** in 68% and 7% calculated yields.

Triisopropyl((6-methyl-4-(2-(4-nitrophenoxy)propan-2-yl)-1-vinyl-1,2,4,5,6,6-hexahydropentalen-1-yl)oxy)silane (9)

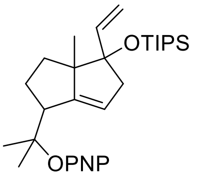

Prepared following general procedure C, by reaction of **6** (54.7 mg, 0.20 mmol, 1 equiv.) with **2j** (136 mg, 0.60 mmol, 3 equiv.) using catalyst **B**. Column chromatography (silica gel, 100% cyclohexane to 100:1 cyclohexane/EtOAc) delivered a mixture of the title compound together with compound **7h** as a colorless oil (54% and 39% NMR yields, respectively). The mixture was dissolved in EtOH (2 mL) and 2 drops of concentrated HCl were added. The solution was stirred for 24 h at 23 °C. Then it was diluted with water and extracted with DCM. The organic layer was washed with a saturated solution of $NaHCO_3$ and brine. Column chromatography (silica gel, 100% cyclohexane to 100:1 cyclohexane/EtOAc) delivered compound **9** as a colorless oil (36.8 mg, 37% yield).

^{1}H NMR (500 MHz, $CDCl_3$) δ 8.17 – 8.12 (m, 2H), 7.07 – 7.00 (m, 2H), 5.87 (ddd, J = 17.6, 10.9, 0.9 Hz, 1H), 5.38 (dt, J = 3.8, 1.9 Hz, 1H), 5.05 (dd, J = 10.9, 1.3 Hz, 1H), 4.97 (dd, J = 17.6, 1.3 Hz, 1H), 2.98 (dd, J = 15.0, 4.2 Hz, 1H), 2.75 – 2.69 (m, 2H), 2.10 – 1.96 (m, 2H), 1.70 – 1.56 (m, 1H), 1.40 (s, 3H), 1.39 (s, 3H), 1.25 (s, 3H), 1.11 (d, J = 7.4 Hz, 3H), 1.05 (br s, 19H).

^{13}C NMR (126 MHz, $CDCl_3$) δ 162.3, 155.7, 142.9, 126.0, 125.4, 121.9, 120.1, 119.2, 112.8, 86.8, 84.2, 62.9, 49.9, 46.6, 31.0, 30.8, 25.3, 24.8, 19.8, 18.5, 18.5, 17.9, 13.5, 12.8.

HRMS (ESI+): m/z calc. for $[C_{29}H_{45}NNaO_4Si]^+$: 522.3010, found: 522.3004.

(1S,3aR)-3,5,6-Trimethyl-1-(2-(4-nitrophenoxy)propan-2-yl)-1,2,3,3,4,7-hexahydroazulene (7i)

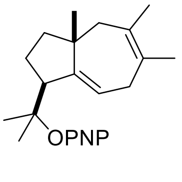

Prepared following general procedure C, by reaction of **6** (57.2 mg, 0.21 mmol, 1 equiv.) with **2g** (49.3 mg, 0.60 mmol, 2.9 equiv.) using catalyst **D**. Column chromatography (silica gel, 100% cyclohexane to 100:1 cyclohexane/EtOAc) delivered the title compound together with compound **8i** as a colorless oil (61.2 mg, 1:1.6 ratio, 82% overall yield: 32% + 50% calculated yields).

Note: **7i** and **8i** are susceptible to oxidation, thus they are not stable to prolonged exposure to air at room temperature, neither dry or in solution. NMR signals appear in the aldehyde region when **7i** and **8i** decompose.

^{1}H NMR signals of both compounds could be partially assigned by analogy.

^{1}H NMR (400 MHz, $CDCl_3$) δ 8.21 – 8.07 (m, 4H, **7i** + **8i**), 7.12 – 6.97 (m, 4H, **7i** + **8i**), 5.63 (ddd, J = 5.3, 4.3, 2.0 Hz, 1H, **7i**), 4.71 (dd, J = 1.9, 0.8 Hz, 1H, **8i**), 4.66 (p, J = 1.4 Hz, 1H, **8i**), 3.13 – 2.95 (m, 3H, **7i** + **8i**), 2.68 (d, J = 23.9 Hz, 1H, **7i**), 2.48 (d, J = 14.0 Hz, 1H, **7i**), 2.39 – 2.26 (m, 2H, **7i** + **8i**), 2.23 – 2.12 (m, 2H, **7i** + **8i**), 1.96 – 1.88 (m,

2H), 1.83 (dt, J = 8.3, 2.8 Hz, 1H), 1.77 (t, J = 1.4 Hz, 3H, **8i**), 1.75 – 1.71 (m, 3H, **7i**), 1.68 – 1.65 (m, 3H, **7i**), 1.64 – 1.58 (m, 2H), 1.55 (d, J = 1.5 Hz, 3H, **8i**), 1.47 – 1.42 (m, 2H), 1.38 (s, 3H, **8i**), 1.38 (s, 3H, **7i**), 1.37 (s, 3H, **7i**), 1.35 (s, 3H, **8i**), 1.34 – 1.30 (m, 1H), 1.24 – 1.19 (m, 2H), 1.13 (s, 3H, **8i**), 1.06 (s, 3H, **7i**), 0.59 (dd, J = 6.6, 4.5 Hz, 1H, **8i**).

^{13}C NMR (101 MHz, $CDCl_3$) δ 162.1, 162.0, 149.4, 148.5, 142.3, 142.1, 140.6, 133.8, 128.1, 127.0, 125.2, 125.1, 125.0, 123.4, 122.7, 122.0, 121.9, 121.6, 121.4, 108.7, 86.9, 85.7, 59.3, 54.6, 48.0, 45.5, 41.0, 37.7, 36.7, 25.8, 25.7, 25.5, 25.2, 24.7, 24.5, 23.9, 23.8, 23.7, 23.4, 23.3, 21.6, 20.6, 19.7, 18.8, 15.3.

HRMS (ESI+): m/z calc. for $[C_{22}H_{29}NNaO_3]^+$: 378.2040, found: 378.2053.

(±)-(1*S*,3*R*)-3-Methyl-1-(2-(4-nitrophenoxy)propan-2-yl)-1,2,3,3,4,9-hexahydro-4,9-[1,2]benzenobenzo[*f*]azulene (7j)

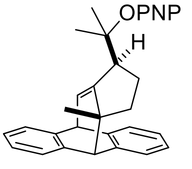

Prepared following general procedure C, by reaction of **6** (54.7 mg, 0.20 mmol, 1 equiv.) with anthracene (**2q**) (107 mg, 0.60 mmol, 3 equiv.) using catalyst **A**. Column chromatography (silica gel, 100% cyclohexane to 100:1 cyclohexane/EtOAc) delivered the title compound as a white foam (60.7 mg, 67% yield).

^{1}H NMR (500 MHz, $CDCl_3$) δ 8.14 – 8.05 (m, 2H), 7.30 – 7.23 (m, 3H (overlap with $CHCl_3$ residual peak), 7.17 – 7.05 (m, 5H), 6.97 – 6.90 (m, 2H), 6.19 (dd, J = 8.2, 1.9 Hz, 1H), 4.24 (d, J = 8.2 Hz, 1H), 3.87 (s, 1H), 2.69 – 2.61 (m, 1H), 1.77 – 1.66 (m, 1H), 1.65 – 1.57 (m, 1H), 1.52 – 1.43 (m, 2H), 1.29 (s, 3H), 1.25 (s, 3H), 0.98 (s, 3H).

^{13}C NMR (126 MHz, $CDCl_3$) δ 162.1, 147.8, 144.1, 143.6, 142.6, 142.1, 138.9, 129.3, 128.8, 128.0, 126.0, 125.8, 125.7, 125.7, 125.4, 125.2, 123.5, 122.3, 84.7, 56.4, 55.4, 47.4, 45.6, 35.7, 26.1, 25.8, 25.1, 23.8.

HRMS (ESI+): m/z calc. for $[C_{30}H_{29}NNaO_3]^+$: 474.2040, found: 474.2039.

MP (EtOAc): 81-84 °C.

8. Mechanistic Studies on the Rh(II)-Catalyzed Decarbenation–(4+3) Cycloaddition

8.1 Kinetic Experiments

Kinetic profile of the overall reaction in TCE-*d2* at 50 ºC, followed by ^{1}H NMR, showing accumulation and consumption of *cis*-divinylcyclopropane intermediate, and then consumption to give rise cleanly to cycloheptadiene **3a**.

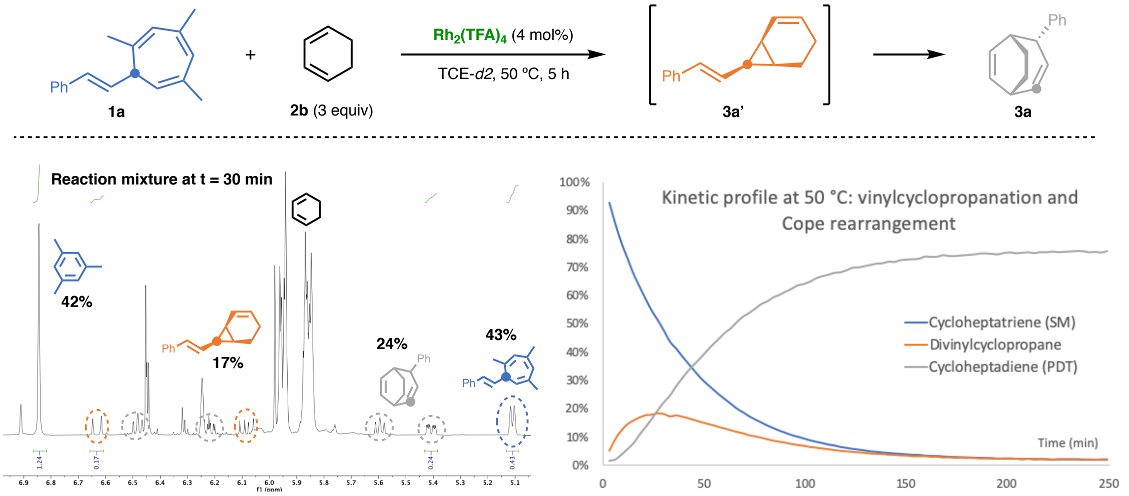

Interrupted reaction: Removal of the Rh(II) catalyst and kinetic profile of free Cope rearrangement followed by ^{1}H NMR at 30 ºC in $CDCl_3$. The reaction was set up according to general procedure B (0.2 mmol of **1a**), and was run at 30 ºC for 1 h. After this time, quick flash column chromatography in pentane allowed the complete removal of the Rh(II) catalyst. The resulting mixture was concentrated in vacuum at 25 ºC, and redissolved in $CDCl_3$ (around 1:30 h total time from the initial addition of the catalyst). The kinetic profile of the free Cope rearrangement was then monitored by NMR.

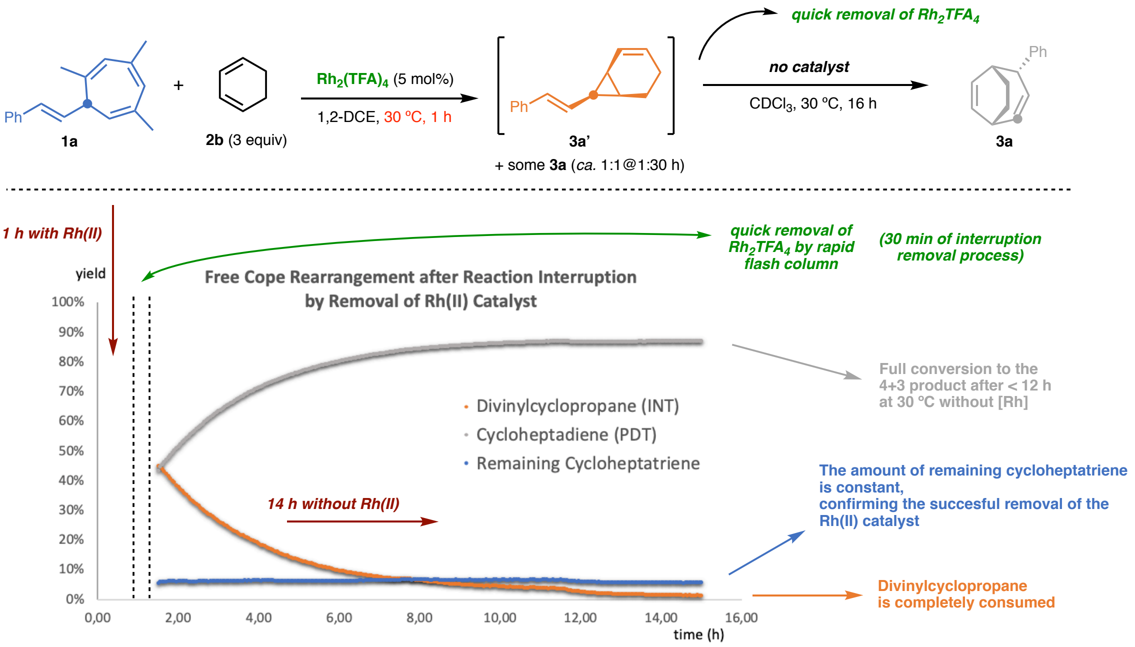

8.2 DFT Calculations

All DFT calculations were carried out with the Gaussian 09 package.[8] The B3LYP[9] functional was used for optimizations and frequency calculations, combined with the LANL2DZ[10] (and the associated pseudopotential) basis set for rhodium and gold, and the 6-31G(d,p)[11] basis set for all the other atoms. Frequency calculations were analyzed to characterize the nature of the stationary points as minima (no imaginary frequency) or transition state (one imaginary frequency). Additionally, relaxation of transition states towards previous and next intermediates was used to verify the connectivity of the transition states. Finally, potential energies were refined using the LANL2TZ[12] basis set for rhodium and gold, and the 6-311++G(d,p)[13] for all the other atoms. Solvation Model Based (SMD)[14] was used to simulate dichloroethane (for rhodium reactions) or dichloromethane (for gold reactions) solvent throughout all calculations. Unless otherwise stated, all the energies presented are potential (E) and free energies (G) in solution at 298.15 K and 1 atm in kcal/mol. Optimized geometries were visualized using CYLView.[15]

All energies are relative to Rh_2TFA_4 coordinated to 1,3-cyclohexadiene (**I**), the resting state of the catalytic cycle. The following scheme shows the full mechanistic picture for the process. This summarizes the most relevant and favorable (lower $\Delta G^{\ddagger}$) pathways found. For each specific part, more details will be provided afterwards, with alternative and higher energetic pathways, discussed as separate figures.

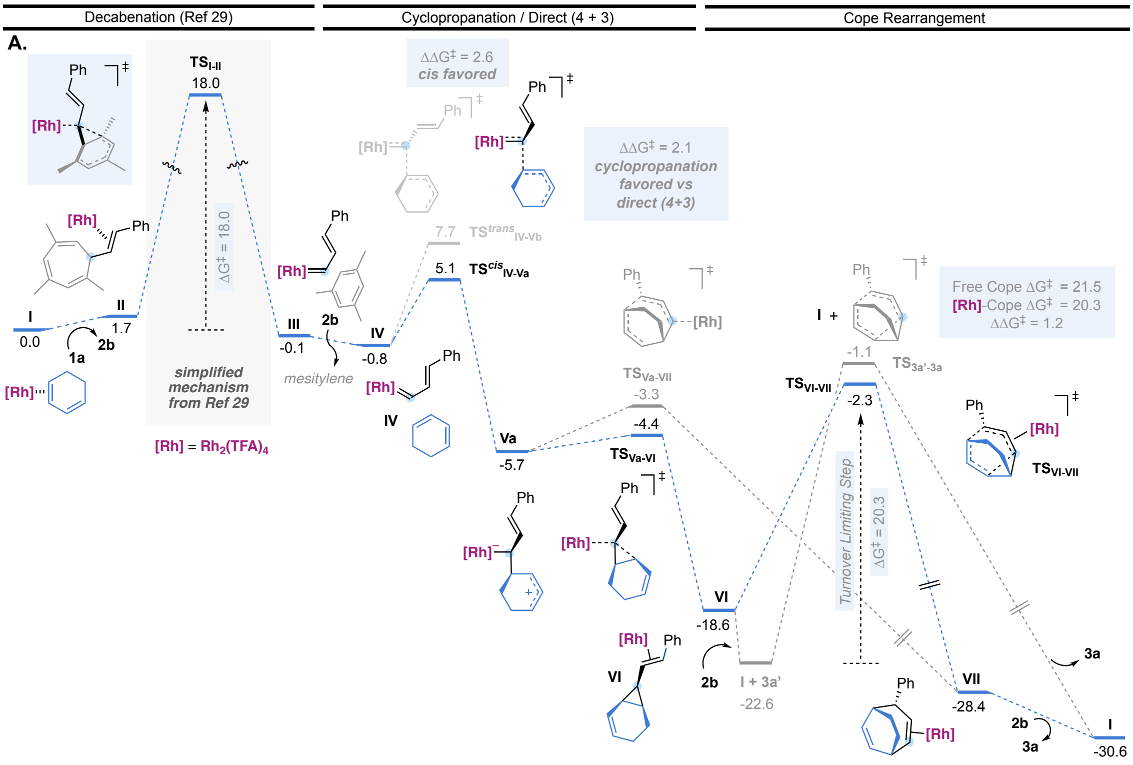

1. Decarbenation or Retro-Buchner Reaction: The first part of the process is the Rh-catalyzed decarbenation of cycloheptatriene **1a** to give carbene **III**, already described by us in a previous publication.[3c]

2. Cyclopropanation vs Direct (4+3): Two different energetically reasonable outcomes were found for the reaction of carbene **IV** with 1,3-cyclohexadiene **2b**, and will be discussed in detail in the next section. The most favorable one is depicted on this scheme: a stepwise cyclopropanation, in which the formation of the *cis* open intermediate

is more kinetically favored than the *trans,* by 2.6 kcal/mol, which would account for a perfect *cis* selectivity (80:1).

The resulting open intermediate can evolve through two pathways: a barrierless cyclopropanation, giving the corresponding *cis*-divinylcyclopropane **VI** (**3a'**), or a direct (4 + 3) closing, which would afford directly the final cycloheptadiene product **VII** (**3a**). Although both pathways are energetically viable, the cyclopropanation TS is 2.1 kcal/mol lower in energy.

3. Cope Rearrangement: **[Rh]-3a'** intermediate **VI** can undergo a Cope rearrangement through $\mathbf{TS_{VI\text{-}VII}}$, with $\Delta G^{\ddagger}$ of 16.4 kcal/mol. However, in the presence of an excess of 1,3-cyclohexadiene, intermediate **VI** is in downhill equilibrium with free divinylcyclopropane **3a' + I** (Rh_2TFA_4 coordinated to 1,3-cyclohexadiene), resulting in a total activation barrier of $\Delta G^{\ddagger}$ = 20.3 (larger, but rather similar, than that for the retro-Buchner step), making it the turnover-limiting step of the entire process.

Alternatively, after decoordination from the Rh(II), free divinylcyclopropane **3a'** can also undergo a "free Cope" thermal rearrangement giving cycloheptadiene **3a**. The barrier for this process was found to be only slightly higher ($\Delta\Delta G^{\ddagger}$ = 1.2) than that for the Rh(II)-coordinated Cope rearrangement: $\Delta G^{\ddagger}$ = 21.5. As a further alternative, intermediate **VI** could go back to open intermediate **Va**, and then react through the direct (4+3) mechanism, with an overall activation barrier of $\Delta G^{\ddagger}$ = 19.3.

Considering any of the scenarios above (or the likely combination of all, given how close in energy they are), the experimental kinetic profile of the reaction, in which an accumulation of intermediate **3a'** is observed, is consistent with the theoretical results.

Concerted vs Stepwise Cyclopropanation

Four different reactivity outcomes were found for rhodium carbene **III** with 1,3-cyclohexadiene: *cis* and *trans* concerted pathways, and *cis* and *trans* stepwise pathways.

The concerted pathway was found when the second alkene of the diene is oriented towards to the rhodium complex (proximal, **IV'**). The stepwise pathway was found when the second alkene is away from the rhodium complex (distal, **IV**). Apparently, the positive charge of the cationic intermediate is more stabilized if the second alkene is away from the metal complex.

Although the stepwise *cis* TS is below in energy than the concerted one, both of them are most likely involved in the reaction ($\Delta\Delta G^{\ddagger}$ = 1.2 kcal/mol; 8:1 stepwise/concerted). This is a reasonable scenario, considering that according to our previous calculations,[3c] the cyclopropanation of cyclohexene (almost no stabilization of the positive charge in the open intermediate) took place in a concerted manner, and the cyclopropanation of styrene (highly stabilized open intermediate) in a stepwise fashion; 1,3-cyclohexadiene sits in the middle of the two.

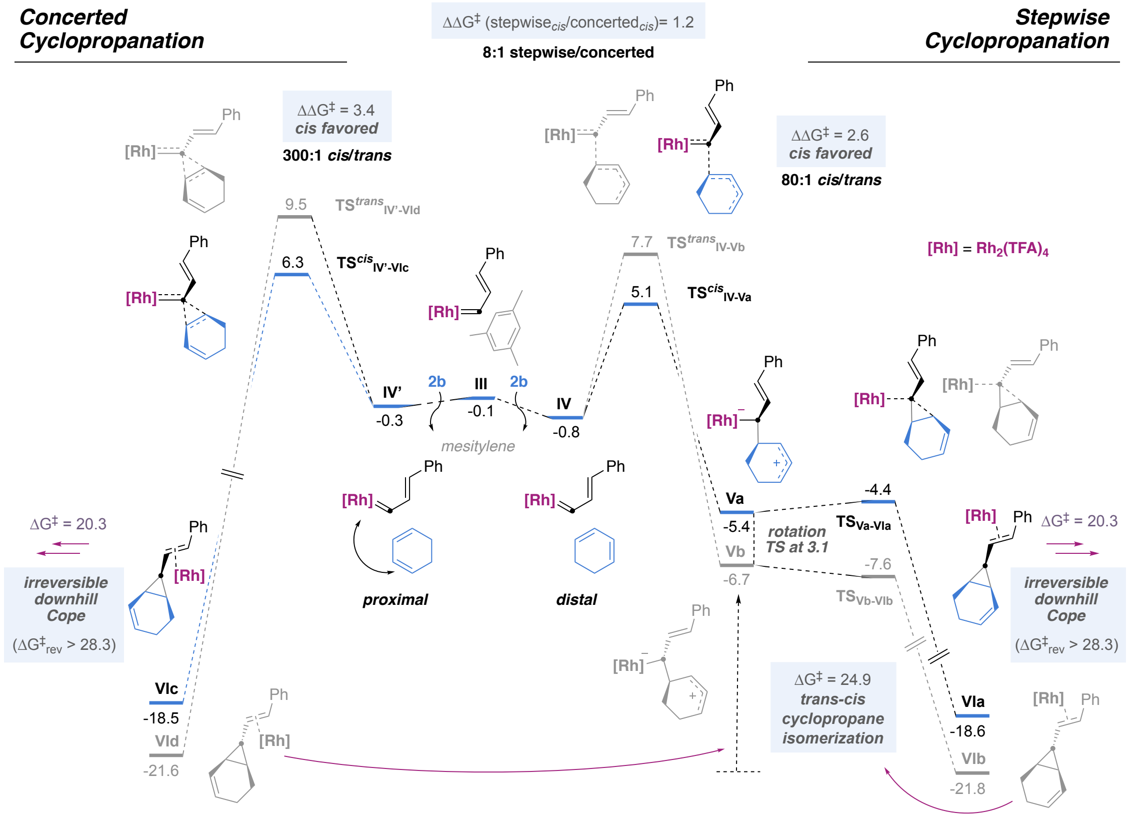

As mentioned before, the *cis* cyclopropane intermediates **VIa**/**VIc** can evolve though a $\Delta G^{\ddagger}$ = 20.3 barrier, which goes downhill towards the formal (4+3) cycloaddition product **3a**, irreversibly ($\Delta G^{\ddagger}_{rev}$ = 28.3–29.5).

If some amount *trans* cyclopropane is formed, it could potentially isomerize back ($\Delta G^{\ddagger}_{trans\text{-to-}cis}$ = 24.9 kcal/mol, see next scheme) to the *cis* open intermediate (or *cis* cyclopropane), converging towards the same only diastereoisomer of **3a**, which is the only product observed experimentally.

cis-trans Isomerization and Possible Fates of Cyclopropanes

The *cis* and *trans* isomers can interconvert through reversible opening of the cyclopropane ring and a *cis-trans* rotation. After rotation, the open intermediates can close back to the corresponding cyclopropane through a barrierless process.

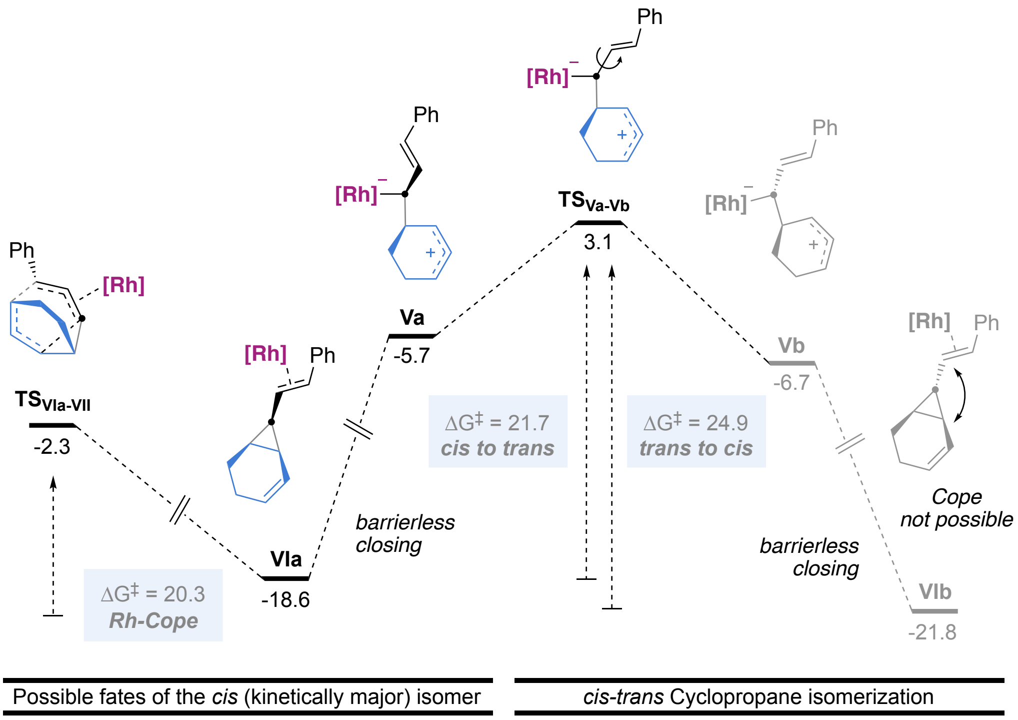

The activation barriers for the isomerization are rather high (ΔG‡ = 21.7–24.9 kcal/mol), but still reasonable while working at 40 ºC.

The fact that the Cope rearrangement is both irreversible and has a lower activation barrier than the isomerization, (together with a kinetically preferred *cis* selectivity) accounts for the clean reactivity observed experimentally (no *trans* divinylcyclopropane observed).

Study of the Free Cope Rearrangement of Different Substrates

While studying the scope of the transformation, we observed that certain substitution patterns or ring sizes on cyclic 1,3-dienes behaved differently, as usual for the Cope rearrangement. (*This is not a model for the entire reaction, but a rather simplistic model for the free Cope rearrangement of the divinylcyclopropane intermediates studied, which correlates with the observed experimental result)*.

First, for cyclic 1,3-dienes, ring size has a significant influence. Smaller 5- and 6-membered rings evolve smoothly to the (4 + 3) product, even when the alkenes are highly substituted (**3s**). Moving up to a 8-membered ring, the corresponding divinyl cyclopropane **3x'** is obtained at 40 ºC. Heating this product at 160 ºC, makes it evolve into cycloheptadiene **3x**, as it was confirmed by x-ray diffraction of analogous **3y**.

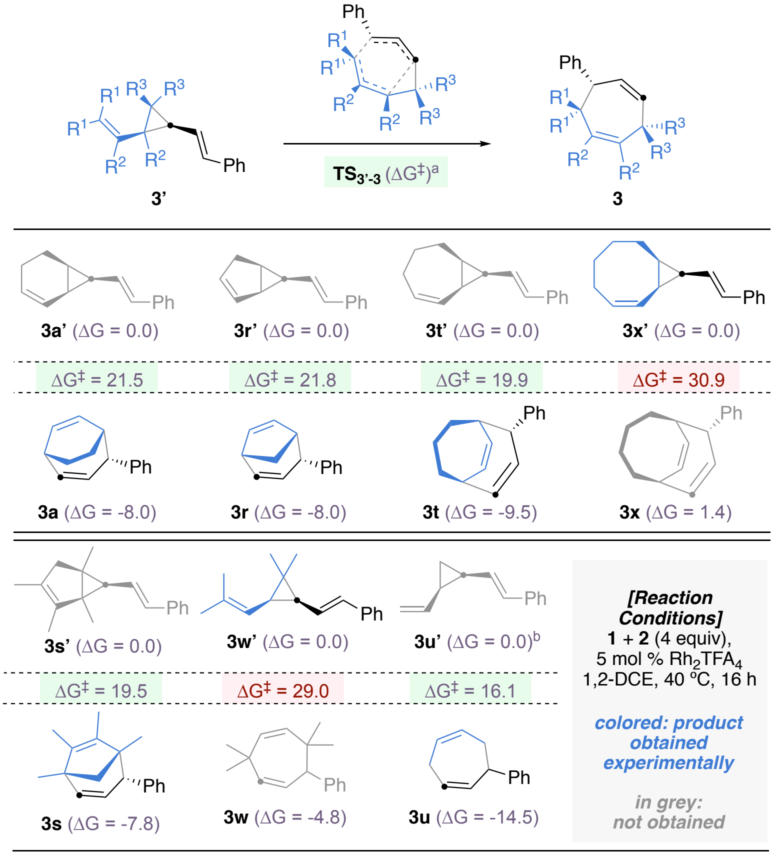

[a] Free energies for the free Cope TS, without [Rh] coordination.

Regarding non-cyclic dienes, 1,1,4,4-tetramethylbutadiene leads to the formation of divinylcyclopropane **3w'**, which gives no conversion even after heating to 130 ºC (decomposition takes place faster than Cope rearrangement). Running the same calculation for simple 1,3-butadiene, a much lower activation barrier is found.

This model could serve as a quick and simple way to predict what product one should expect for a particular substrate. It allowed us to successfully identify reaction partners such as 1,3-butadiene and 1,3-cycloheptadiene as precursors of (4+3) products under mild conditions, before running the reactions in the laboratory.

Finally, a simple diastereospecific Cope model was employed to further assign the relative stereochemistry of oxygenated products **3i–k** (since nOe NMR experiments were not conclusive, and crystallization of **3k** was not successful).

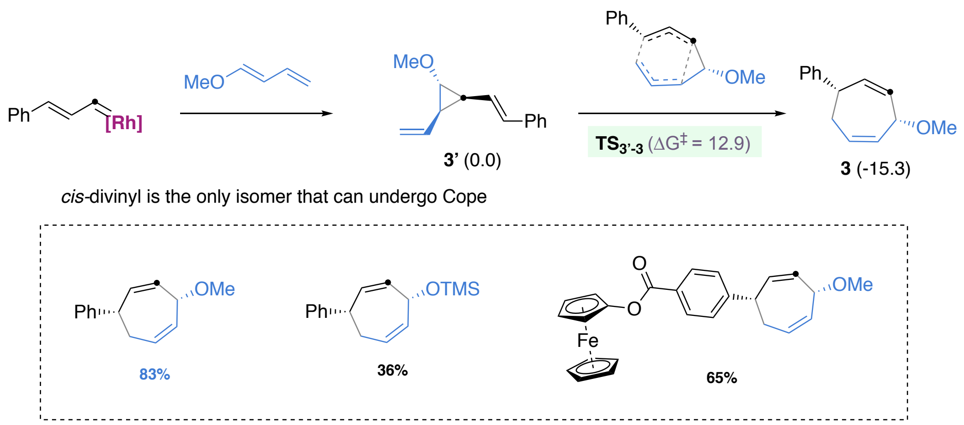

Assuming a cyclopropanation of the much more reactive enol ether double bond, and considering that the *cis* isomer of **3'** is the only one that can undergo a Cope rearrangement (or direct (4+3) ring closure), the intermediate would evolve to give exclusively *cis*-1,4-cycloheptadienes **3** through **TS**$_{3'-3}$.

9. Mechanistic Studies on the Au(I)-Catalyzed Cycloisomerization–Migration–(4+3) Cycloaddition

9.1 Experiments

We monitored the reaction between PNP-enyne **6a** (1 equiv) and TIPSO-diene **2j** (3 equiv) with catalyst **D** (2 mol %) in $CDCl_3$ (0.1 M) at 0 °C by ^{1}H-NMR. No intermediate species could be detected under these conditions.

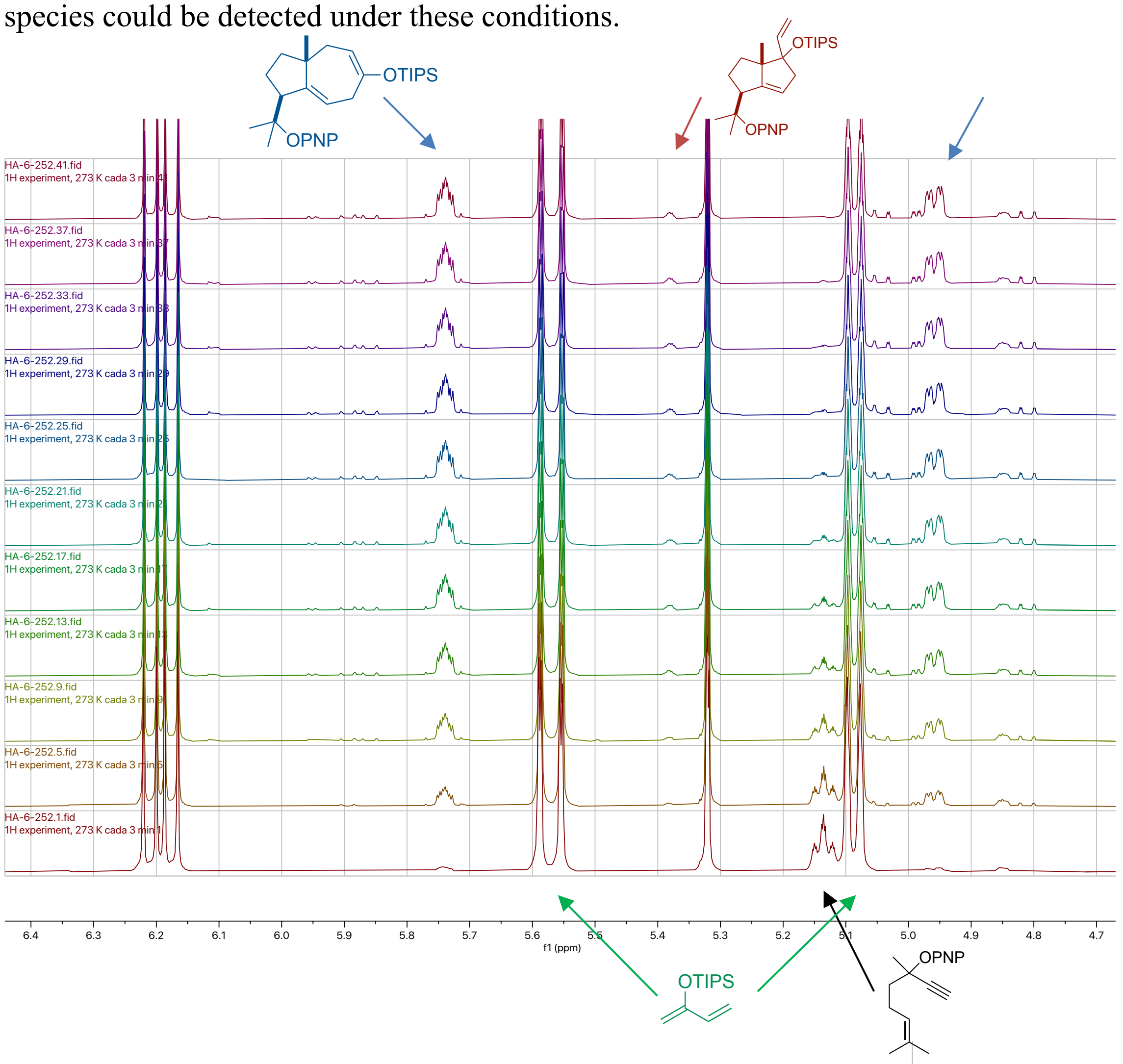

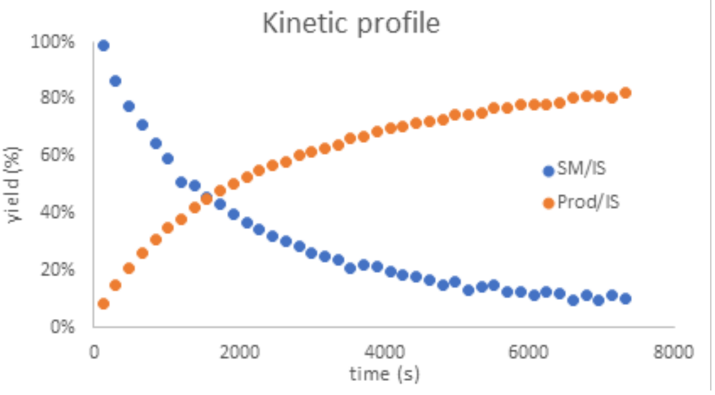

Same result was observed when monitoring the reaction at -20 ºC (although with longer reaction time).

Additionally, we studied the thermal decomposition of products **7f** and **8f** under different conditions.

- A solution of **7f** and **8f** in $(CDCl_2)_2$ was gradually warmed up in an NMR tube in the presence of trichloroethene as internal standard. The mixture of products was stable up to 120 ºC in $(CDCl_2)_2$, when both compounds fully decomposed after 12 h. No interconversion of **8f** into **7f** was observed.

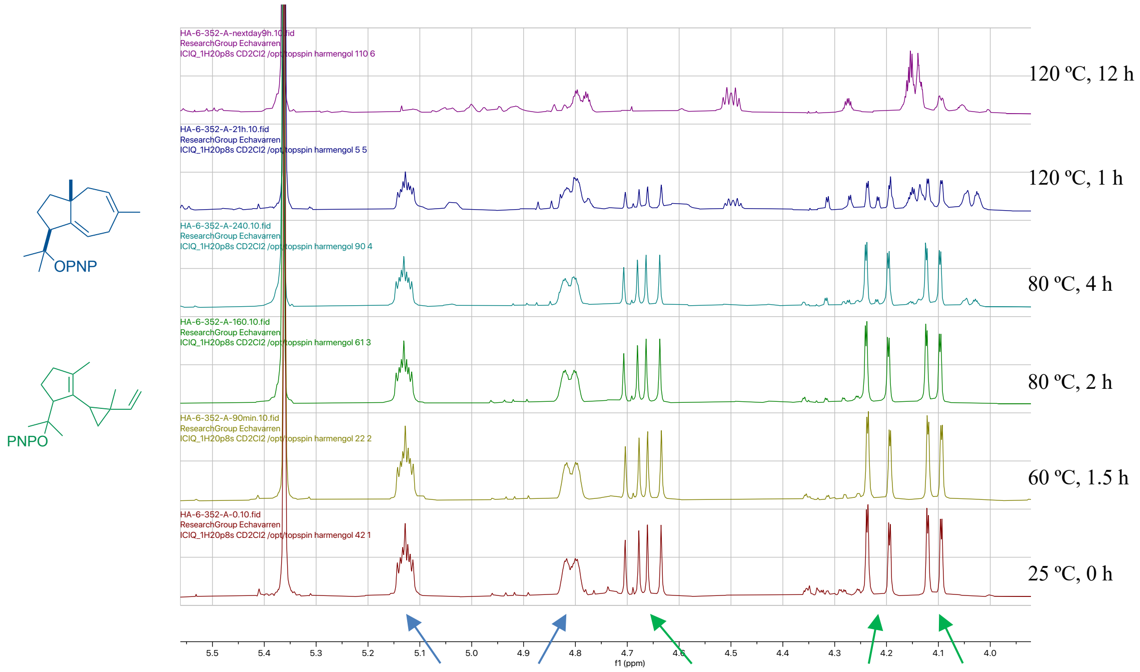

- To a solution of **7f** and **8f** in $(CDCl_2)_2$ was added catalyst A (5 mol %) and the mixture was gradually warmed up in an NMR tube in the presence of trichloroethene as internal standard. Some decomposition could be observed at 60 ºC after 20 h. Although in this case **7f** had a slower decomposition than **8f**, no interconversion of **8f** into **7f** was observed.

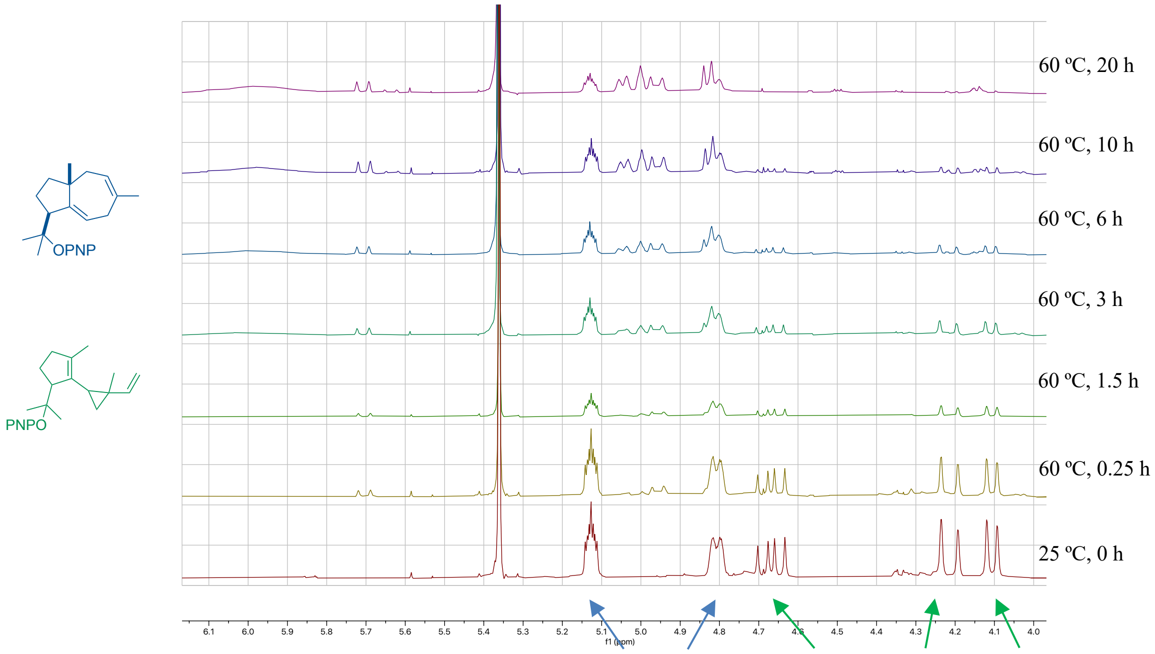

9.2 DFT Calculations

For computational details, see the beginning of section 8.2. Additionally, in all the section R = *para*nitrophenyl and L = JohnPhos.

9.2.1 Computational study of the reaction with cyclopentadiene (2a)

The first sequence of steps of the gold(I) catalyzed cascade reaction is the cycloisomerization and 1,5-OR-migration to deliver intermediate **IX**, which was previously studied by our group.[16] Thus, we focused on the intermolecular trapping of carbene **IX**. However, we calculated the first step of the cycloisomerization/migration sequence in order to establish the relative zero of energy and the highest energy barrier of the catalytic cycle. The following scheme shows a summary of the calculations performed for the gold(I) catalysis, which will be discussed in detail in this section.

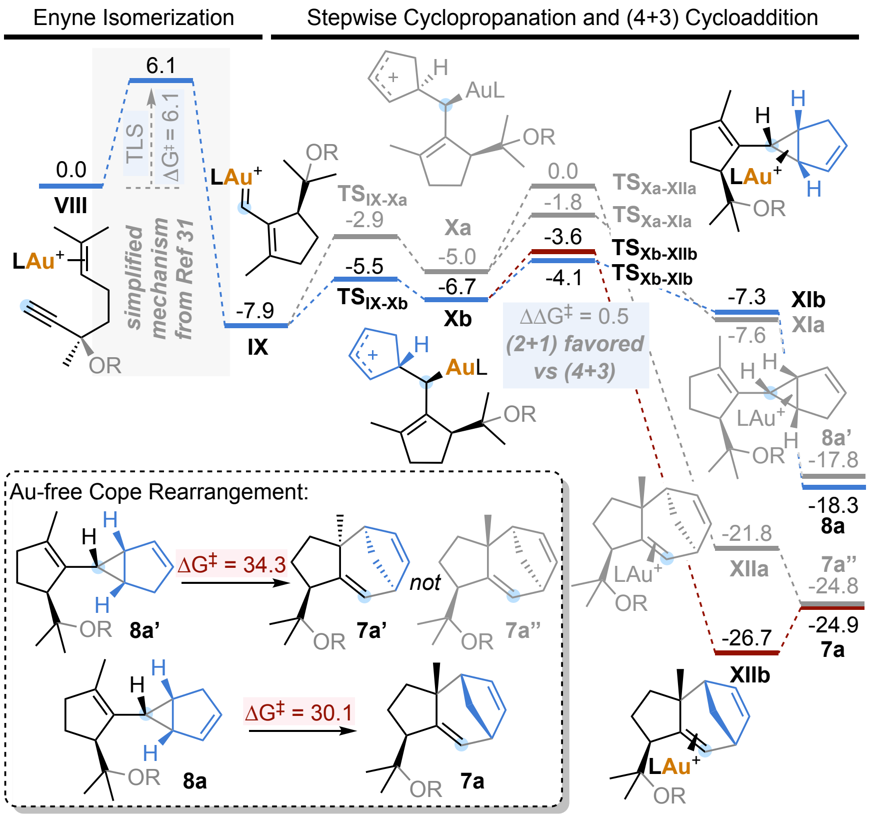

Establishing the zero energy:

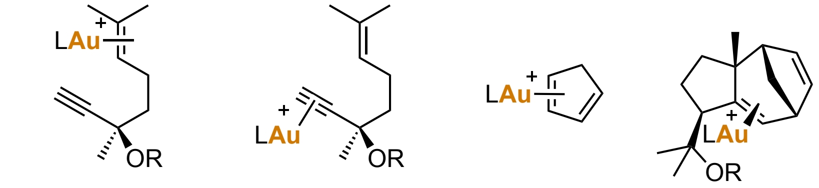

VIII (6a-Au alkene complex)	6a-Au alkyne complex	Cp-Au	XIIb
0.0	2.5	1.1	-1.8

From the gold(I) complexes with the starting materials, the lowest energy found was for the complex of **6a** through the alkene moiety, and thus this was established as the relative zero energy of the system. However, **XIIb**, corresponding to product **7a** bound to gold, has lower energy than free **7a** (-1.8 kcal/mol), indicating mild product inhibition. This means that for the second catalytic cycle to start, additional 1.8 kcal/mol will have to be overcome. Thus, in the second catalytic cycle, the highest energy barrier of the system, which is the 5-exo-dig cycloisomerization with 6.1 kcal/mol, will increase to 7.9 kcal/mol.

In this system, the product inhibition does not translate into a detectable slowing down of the reaction, because 7.9 kcal/mol is still a relatively low energy barrier to overcome at 25 ºC. This explains why experimentally the reaction is completed in less than 2 h and the NMR-monitoring does not exhibit the typical product inhibition kinetic profile.

Enyne isomerization:

As found in our previous report, the rate determining step towards intermediate **IX** is the 5-*exo*-dig cycloisomerization of enyne **6a**. Thus, we extrapolated the reported calculations to our system (using JohnPhos as the ligand for gold) to find the energy of the rate determining step of the cycle.

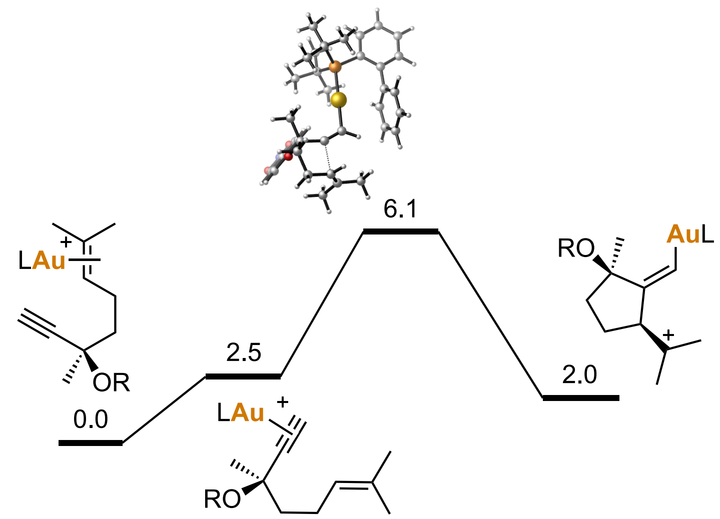

We did not calculate the 1,5-OR-migration process, because this was discussed in detail in our previous publication.[4]

Intermolecular trapping of carbene I:

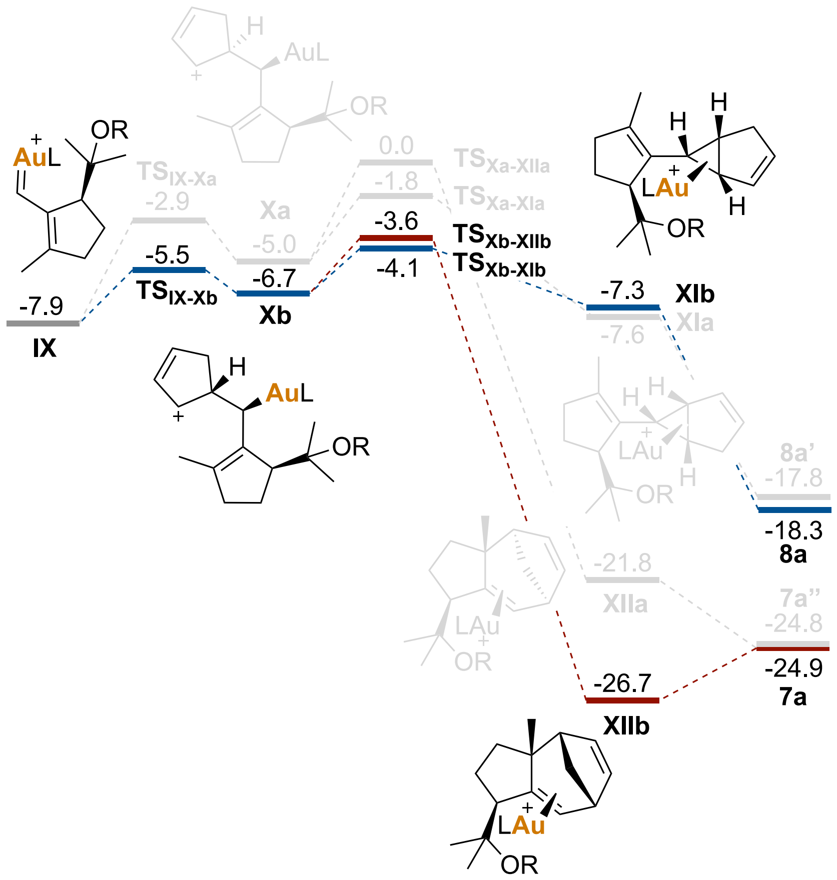

As explained in the main text of this publication, cyclopentadiene can approach carbene **IX** from two different faces, leading to intermediates **Xa** and **Xb**. We found that the pathway towards **Xb** is more favorable by 2.6 kcal/mol. **Xa** can evolve towards intermediates **XIa** and **XIIa** *via* **TS**$_{\mathbf{Xa\text{-}XIa}}$ and **TS** $_{\mathbf{Xa\text{-}XIIa}}$ respectively, with a difference of 1.8 kcal/mol. Analogously, **Xb** can lead to **XIb** and **XIIb** *via* **TS**$_{\mathbf{Xb\text{-}XIb}}$ and **TS** $_{\mathbf{Xb\text{-}XIIb}}$, with very similar energy barriers. Final decomplexation delivers products **7a**, **7a''**, **8a** and **8a'**.

We also attempted to calculate a concerted pathway for the formation of intermediates **XI** and **XII**. However, we could not find any transition state connecting carbene **XI** with the cyclized products in a concerted manner. The carbene trapping is highly dependent on the diene nature and thus other dienes might follow a different mechanism, as found in other DFT-studies for the trapping of Au(I) carbenes.[17]

Au-free Cope rearrangement:

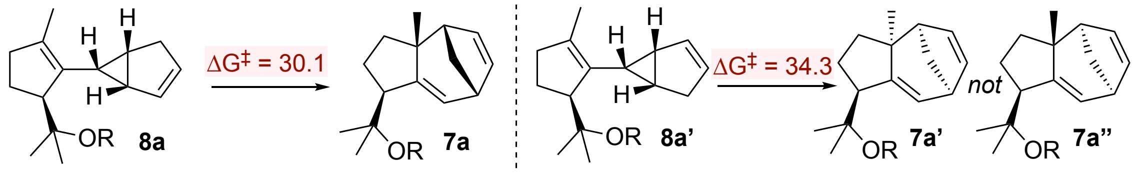

Computationally, we found that cyclopropane **8a** can undergo a concerted thermal Cope rearrangement to deliver **7a** *via* a TS of 30.1 kcal/mol. Analogously, **8a'** can lead to **7a'** through an energy barrier of 34.3 kcal/mol. Interestingly, in this last case, **8a'** has to adopt a less stable conformation in order for the rearrangement to take place, explaining the higher energy barrier. Additionally, **7a''** cannot be obtained by rearrangement of neither **8a** and **8a'**, since the thermal Cope rearrangement follows a stereospecific mechanism. Thus **7a''** can only be formed by stepwise 4+3 cycloaddition of intermediate **IX**.

The energy barriers do not differ much from the one found for cyclopropane **3x'** (30.9 kcal/mol), however, we found experimentally that decomposition is faster than the Cope rearrangement for these substrates.

9.2.2 Computational study of the reaction with cyclohexadiene (2b)

Intrigued by the different reactivity observed between dienes **2a** and **2b**, we decided to study the reaction with the later also by means of DFT calculations.

Establishing the zero energy:

In this case, the gold(I) complex with **2b** was established as zero energy. In contrast to the previous section (with **2a**), gold(I) complex **XVIb** (with side product **7d''**) was found to be slightly higher in energy (only by 0.8 kcal/mol) than the complex with starting material **2b**. Thus, there is no product inhibition in this case.

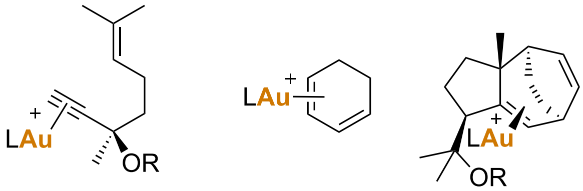

6a-Au alkyne complex	Ch-Au	XVIb
1.8	0.0	0.8

Intermolecular trapping of the gold(I) carbene:

The first sequence of steps is again the isomerization of **6a**, discussed in the previous section. Thus, we focused on the intermolecular trapping of the vinylcarbene with **2b**, in intermediate **XIII** + **2b**. As found for **2a**, the diene can attack with two different orientations. The most favored one (right pathway) affords carbocation **XIVb** *via* **TS**$_{\mathbf{XIII\text{-}XIVb}}$. This intermediate can then evolve to form the 3-membered or the 7-membered carbocyclic products *via* **TS**$_{\mathbf{XIVb\text{-}XVb}}$ and **TS**$_{\mathbf{XIVb\text{-}XVIb}}$ respectively. The difference in

energy of these two transition states is of 5.7 kcal/mol, which would account for an exclusive formation of **XVb** (and not **XVIb**) and ultimately cyclopropane **8d** after decomplexation (as observed experimentally).

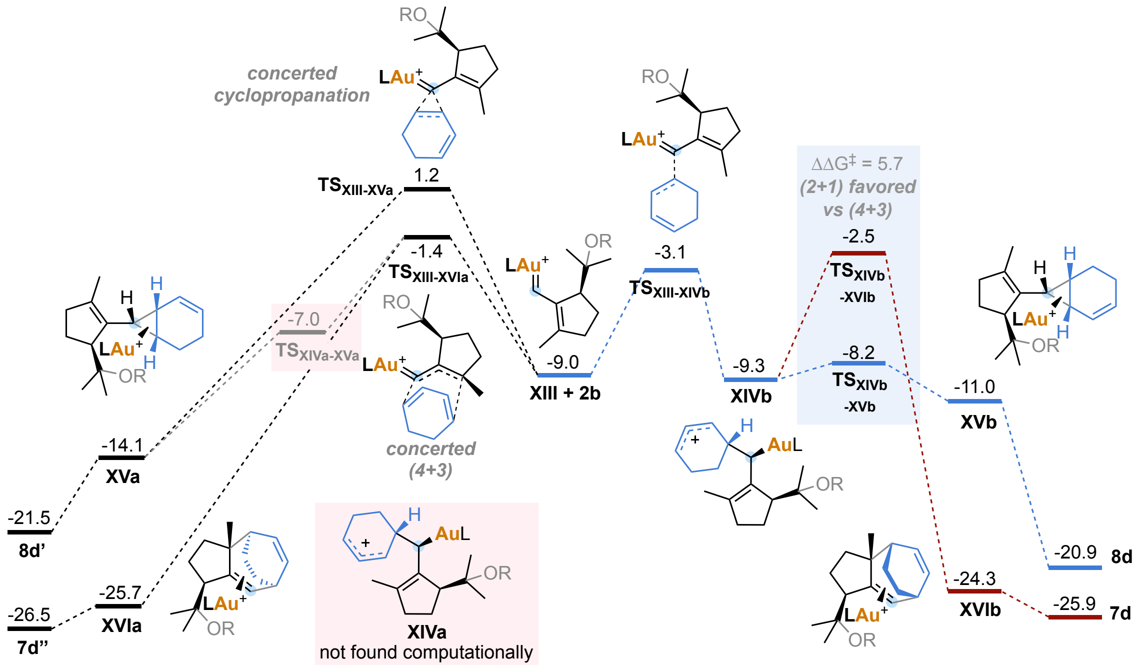

For the less favored attack of the diene to the carbene (left pathway), a more complex scenario was found. It is worth mentioning that this would in any case only lead to the minor products of the reaction. Analogous intermediate **XIVa** was not found, and we were only able to allocate the (2+1) and (4+3) concerted cycloadditions. Interestingly, in this case the concerted (4+3) pathway *via* $\mathbf{TS_{XIII\text{-}XVIa}}$ is lower in energy than the concerted cyclopropanation *via* $\mathbf{TS_{XIII\text{-}XVa}}$ by 2.6 kcal/mol. We attempted to calculate the stepwise process but we could only find a guess for a potential $\mathbf{TS_{XIVa\text{-}XVa}}$ with an energy of -7.0 kcal/mol, by freezing the coordinates involved in the ring-closing transformation. We also tried to find a concerted pathway for the (2+1) and (4+3) cycloaddition reactions towards **XVb** and **XVIb** but we were not successful.

Au-free Cope rearrangement:

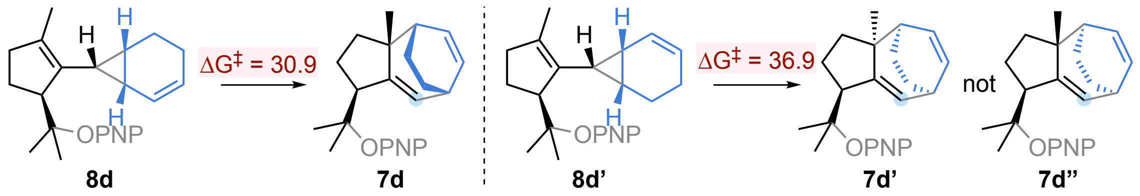

As for the reaction with cyclopentadiene, we calculated the energy barriers of the thermal Cope rearrangement for divinylcyclopropanes **8d** and **8d'**. Similar values were found compared to **8a** and **8a'**, being of 30.9 kcal/mol for **8d** rearranging into **7d** and 36.9 kcal/mol for **8d'** rearranging into **7d'** (and not **7d''**).

10. Synthesis of Natural Products and Diversification

10.1 Total Synthesis of (±)-Dictyopterene C' (5)

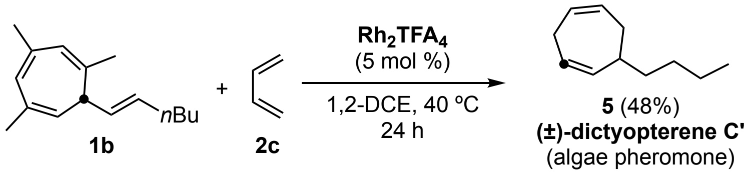

Under Ar, a microwave vial equipped with a Teflon-coated magnetic stirring bar was charged with 1,3,5-trimethyl-7-(hex-1-en-1-yl)-1,3,5-cycloheptatriene **1b** (120 mg, 0.56 mmol, 1.0 equiv) and 1,4-butadiene **2c** (0.75 mL of a 20% w/w solution in toluene, 2.22 mmol, 4 equiv). Both reagents were dissolved in anhydrous 1,2-DCE (0.15 M, 3.8 mL), before $[Rh(TFA)_2]_2$ (16 mg, 5 mol %) was added. The vial was sealed and then stirred at 40 ºC for 24 h. After confirming that the reaction was completed by TLC, the resulting mixture was concentrated in vacuum, adsorbed into silica gel, and purified by slow flash column chromatography on silica gel, using pentane as eluent, giving (±)-dictyopterene C' as an odorous colorless oil. Characterization data matched with the reported ones for the natural product.[18]

^{1}H NMR (500 MHz, Chloroform-*d*) δ 5.73 – 5.58 (m, 4H), 2.93 (ddq, J = 19.7, 4.3, 2.2 Hz, 1H), 2.69 (dt, J = 19.7, 5.7 Hz, 1H), 2.44 (s, 1H), 2.20 (ddt, J = 15.8, 6.3, 3.0 Hz, 1H), 2.13 – 2.05 (m, 1H), 1.34 – 1.28 (m, 7H), 0.89 – 0.87 (m, 3H).

^{13}C NMR (126 MHz, $CDCl_3$) δ 136.82, 129.87, 128.09, 127.19, 37.16, 35.98, 32.85, 29.40, 28.32, 22.81, 14.03.

HRMS (GC-MS, EI): calculated for $C_{11}H_{18}$ $[M]^+$: 150.1; found: 150.1.

10.2 Deprotection of the Hydroxylated Skeleton of (7fb)

(±)-2-((1*S*,3*R*)-3,6-Dimethyl-1,2,3,3,4,7-hexahydroazulen-1-yl)propan-2-ol (7fa)

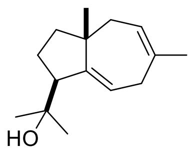

To a suspension of copper(II) acetylacetonate (12.5 mg, 0.048 mmol, 0.20 equiv.) and a mixture of **7f** and **8f** (81.3 mg, 0.238 mmol, 1.3:1 ratio, 1 equiv.) in ethanol (2.4 mL) was added sodium borohydride (27.0 mg, 0.71 mmol, 3 equiv.) at 0 °C and the mixture was stirred for 2 h at room temperature. Water was then added and the mixture was filtered through a short pad of Celite. The reaction mixture was extracted twice with EtOAc, and dried over $MgSO_4$ before removing the solvent under reduced pressure. The crude mixture was dissolved in acetonitrile (10 mL) and cooled to 0 °C. A solution of $(NH_4)_2Ce(NO_3)_6$ (CAN, 365 mg, 0.67 mmol, 2.8 equiv.) in distillated water (3.3 mL) was added dropwise to the reaction mixture. After stirring for 10 minutes at 0 °C, water was added, the mixture was extracted with EtOAc and washed with 10 % $NaHSO_3$, 10 % $NaHCO_3$ and brine. The organic layer was dried over Na_2SO_4, filtered and evaporated under reduced pressure. Column chromatography (silica gel, 10:1 cyclohexane/EtOAc) delivered the title compound as a red crystalline solid (8.3 mg, 30% yield based on **7f**, over two steps).

^{1}H NMR (400 MHz, $CDCl_3$) δ 5.69 (td, J = 5.0, 2.1 Hz, 1H), 5.49 – 5.41 (m, 1H), 2.84 (br s, 2H), 2.72 – 2.61 (m, 1H), 2.27 (d, J = 15.4 Hz, 1H), 1.96 (dd, J = 15.1, 8.2 Hz, 1H), 1.79 – 1.72 (m, 1H), 1.70 (t, J = 1.7 Hz, 3H), 1.53 – 1.46 (m, 2H), 1.45 – 1.35 (m, 1H), 1.24 (s, 3H), 1.17 (s, 3H), 1.08 (s, 3H), (the O*H* signal is missing).

^{13}C NMR (101 MHz, $CDCl_3$) δ 151.0, 134.4, 122.7, 120.5, 73.4, 57.0, 46.0, 41.8, 40.9, 35.1, 30.2, 26.4, 26.4, 25.8, 23.9.

HRMS (ESI+): m/z calc. for $[C_{15}H_{24}NaO]^+$: 243.1719, found: 243.1720.

MP (EtOAc): 69–72 °C.

10.3 Reactivity and Diversification of Cycloheptadienes (3)

(±)-(1*S*,4*S*,5*S*)-4-(2-Ethynylphenyl)-1,5,6,7,8-pentamethylbicyclo[3.2.1]octa-2,6-diene (3o)

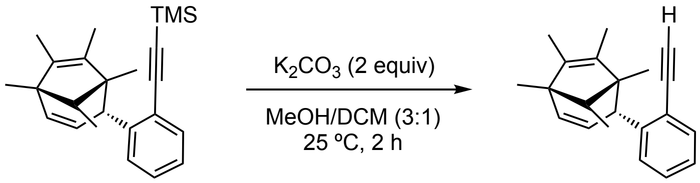

Under air, (**3n**) (32 mg, 0.092 mmol) was dissolved in 0.6 mL of 3:1 MeOH/DCM (HPLC grade) and the mixture was added K_2CO_3 (25 mg, 0.18 mmol, 2 equiv). The reaction was stirred for 2 h (after this time the reaction was finished by NMR, even though in TLC, both SM and product have identical R_f). Water was added, and the mixture was extracted 3 times with DCM. Organic fractions were dried over $MgSO_4$, filtrated, concentrated, resuspended in pentane, and filtered through an HPLC filter. Then, the solvent was removed in vacuum, obtaining pure (**3o**) without further purification.

^{1}H NMR (500 MHz, Chloroform-*d*) δ 7.48 – 7.42 (m, 1H), 7.18 (td, *J* = 7.6, 1.6 Hz, 1H), 7.12 (td, *J* = 7.5, 1.4 Hz, 1H), 6.81 (dd, *J* = 7.8, 1.5 Hz, 1H), 6.05 (dd, *J* = 9.4, 2.5 Hz, 1H), 5.26 (dd, *J* = 9.4, 2.6 Hz, 1H), 3.89 (t, *J* = 2.5 Hz, 1H), 3.22 (s, 1H), 2.08 (q, *J* = 6.8 Hz, 1H), 1.60 (q, *J* = 1.2 Hz, 3H), 0.99 (d, *J* = 3.7 Hz, 6H), 0.94 (q, *J* = 1.2 Hz, 3H), 0.74 (d, *J* = 6.8 Hz, 3H).

^{13}C NMR (126 MHz, $CDCl_3$) δ 145.22, 141.10, 141.03, 132.66, 130.63, 129.38, 128.76, 128.65, 126.38, 123.04, 84.01, 81.26, 57.86, 55.29, 49.34, 48.06, 19.11, 18.70, 12.90, 11.64, 10.82.

HRMS (APCI Pos): calculated for $C_{21}H_{25}$ $[M+H]^+$: 277.1951; found: 277.1952.

(±)-(1*S*,4*S*,5*S*)-4-(3',5'-Dimethoxy-[1,1'-biphenyl]-3-yl)-1,5,6,7,8-pentamethylbicyclo [3.2.1]octa-2,6-diene (3z)

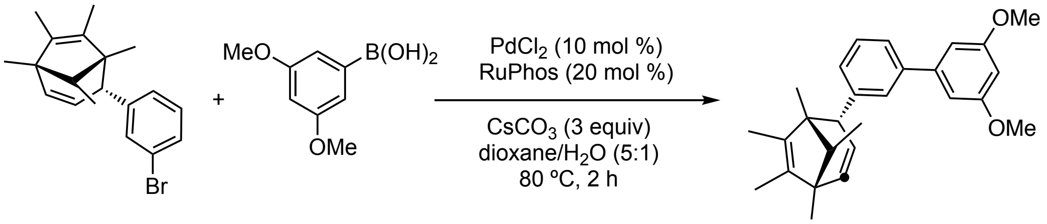

An HPLC vial was charged with (3,5-dimethoxyphenyl)boronic acid (17 mg, 0.091 mmol, 2 equiv), cesium carbonate (44 mg, 0.14 mmol, 3 equiv), $PdCl_2$ (0.8 mg, 10 mol %) and RuPhos (4.2 mg, 20 mol %). The vial was introduced in the glovebox, and then a solution of (**3m**) (15 mg, 0.045 mmol, 1 equiv) in 0.25 mL of dry dioxane (0.15 M) was added. The vial was closed, taken out of the glovebox, and then 0.05 mL of H_2O were added via syringe. The mixture was stirred at 80 ºC for 2 h. After this time, full conversion of (**3m**) is observed by TLC. The mixture was diluted with water and extracted 3 times with diethyl ether. Combined organic fractions were washed once with brine, dried over anhydrous Na_2SO_4. filtered, concentrated under vacuum, and purified by CombiFlash chromatography in silica gel, using a gradient between 100% cyclohexane and 99:1 cyclohexane/EtOAc, to give the title compound (**3z**, 13 mg, 74% yield) as an amorphous colorless residue.

^{1}H NMR (500 MHz, Chloroform-*d*) δ 7.39 (ddd, *J* = 7.7, 1.9, 1.1 Hz, 1H), 7.27 (t, *J* = 7.6 Hz, 1H), 7.19 (s, 1H), 6.99 (d, *J* = 7.5 Hz, 1H), 6.69 (d, *J* = 2.3 Hz, 2H), 6.44 (t, *J* = 2.3 Hz, 1H), 6.09 (dd, *J* = 9.4, 2.4 Hz, 1H), 5.38 (dd, *J* = 9.4, 2.6 Hz, 1H), 3.83 (s, 6H), 3.18 (t, *J* = 2.6 Hz, 1H), 2.02 – 1.98 (m, 1H), 1.61 (q, *J* = 1.2 Hz, 3H), 1.00 (s, 3H), 0.99 (s, 3H), 0.88 (q, *J* = 1.2 Hz, 3H), 0.75 (d, *J* = 6.8 Hz, 3H).

^{13}C NMR (126 MHz, $CDCl_3$) δ 161.36, 144.14, 142.77, 141.72, 140.80, 140.54, 129.61, 129.60, 129.08, 128.36, 128.06, 125.46, 105.71, 99.55, 57.47, 55.75, 53.68, 52.91, 48.09, 18.75, 18.64, 12.74, 11.82, 10.83.

HRMS (APCI Pos): calculated for $C_{27}H_{33}O_2$ $[M+H]^+$: 389.2475; found: 389.2478.

(±)-3,5-Dimethyl-4-((2-((1S,2S,5S)-1,5,6,7,8-pentamethylbicyclo[3.2.1]octa-3,6-dien-2-yl)phenyl)ethynyl)isoxazole (3aa)

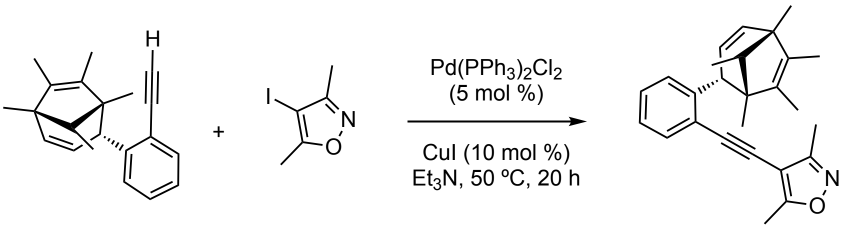

In the glovebox, a mixture of (**3o**) (20 mg, 0.072 mmol), 4-iodo-3,5-dimethylisoxazole (19 mg, 0.087, 1.2 equiv), $Pd(PPh_3)_3Cl_2$ (2.5 mg, 5 mol %), and copper iodide (1.4 mg, 10 mol %), was dissolved in 0.7 mL of anhydrous NEt_3 (0.1 M) in an HPLC vial. The vial was closed, taken out of the glovebox, and the mixture was stirred at 50 ºC for 20 h. After this time, the solvent was removed while adsorbing the crude product in silica gel, and it was purified by CombiFlash chromatography in SiO_2 using a gradient of cyclohexane/EtOAc 95:5 to 9:1, to give the title compound (**3aa**) as a yellow solid.

^{1}H NMR (500 MHz, Chloroform-*d*) δ 7.44 (dd, *J* = 7.4, 1.6 Hz, 1H), 7.21 – 7.14 (m, 2H), 6.84 (dd, *J* = 7.6, 1.4 Hz, 1H), 6.07 (dd, *J* = 9.4, 2.5 Hz, 1H), 5.29 (dd, *J* = 9.4, 2.5 Hz, 1H), 3.89 (t, *J* = 2.5 Hz, 1H), 2.50 (s, 3H), 2.35 (s, 3H), 2.05 (q, *J* = 6.8 Hz, 1H), 1.63 – 1.59 (m, 3H), 1.00 (s, 6H), 0.98 – 0.95 (m, 3H), 0.74 (d, *J* = 6.8 Hz, 3H).

^{13}C NMR (126 MHz, $CDCl_3$) δ 171.21, 160.92, 144.38, 141.32, 141.22, 131.99, 130.72, 129.21, 128.63, 128.54, 126.54, 123.70, 101.88, 94.78, 81.31, 58.07, 55.21, 49.75, 48.12, 19.37, 18.67, 12.91, 12.43, 11.66, 11.09, 10.83.

HRMS (ESI Pos): calculated for $C_{26}H_{30}NO$ $[M+H]^+$: 372.2322; found: 372.2334.

MP: 100– 102 ºC.

(±)-(1*R*,2*R*,5*S*)-2-Phenylbicyclo[3.2.2]nonane (4)

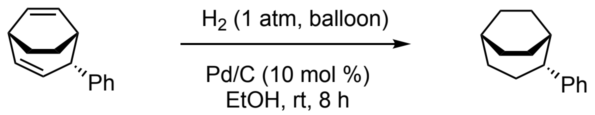

A vial was charged with a solution of (**3a**) (30 g, 0.153 mmol, 1 equiv) in 1.5 mL of HPLC grade EtOH. The vial was submitted to two vacuum-Ar cycles, before Pd/C (16 mg, 10% w/w on Pd, 10 mol %) was added. Then, the vial was submitted to three consecutive vacuum-H_2 cycles (using a hydrogen balloon). The mixture was then stirred under H_2 atmosphere at 25 ºC for 18 h. After this time, GCMS showed conversion to the tetrahydrogenated product. After filtration though Celite and evaporation of the solvent, flash column chromatography in silica gel using pentane as eluent gave the title compound (**4**) (25 mg, 82%) as a colorless oil.

^{1}H NMR (500 MHz, Chloroform-*d*) δ 7.29 – 7.22 (m, 4H), 7.18 – 7.13 (m, 1H), 2.79 (dd, *J* = 12.1, 4.2 Hz, 1H), 1.99 – 1.85 (m, 5H), 1.79 – 1.61 (m, 7H), 1.57 – 1.45 (m, 2H).
^{13}C NMR (126 MHz, $CDCl_3$) δ 149.78, 128.59, 127.20, 125.88, 51.72, 37.29, 35.62, 29.43, 28.83, 28.22, 24.12, 21.95.

HRMS (APCI Pos): calculated for $C_{15}H_{19}$ $[M-H]^+$: 199.1481; found: 199.1476.

11. X-Ray Crystal Data and Structure Refinement

11.1 (±)-(4-((1*R*,2*R*,5*S*)-Bicyclo[3.2.2]nona-3,6-dien-2-yl)phenyl ferrocenoylate (3b)

The single crystals of compound **3b** suitable for X-ray diffraction analysis were obtained by slow evaporation of a solution of **3b** in 9:1 MeCN/CH_2Cl_2 over 24 h. The crystal structure information for this compound has been deposited at the Cambridge Crystallographic Data Centre. CCDC2027382 contains the crystal structure information of this compound and can be obtained free of charge via http://www.ccdc.cam.ac.uk.

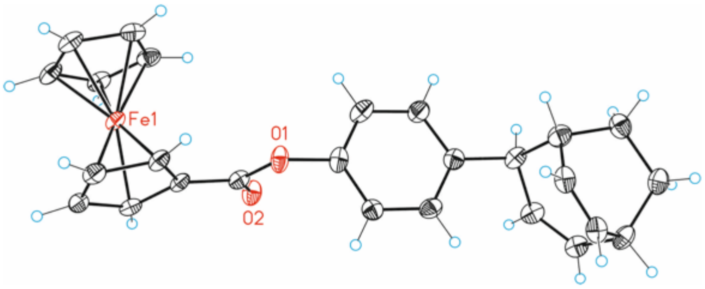

Identification code	MM-2-164	
Empirical formula	C26 H24 Fe O2	
Formula weight	424.30	
Temperature	100(2)K	
Wavelength	0.71073 Å	
Crystal system	triclinic	
Space group	P -1	
Unit cell dimensions	a = 6.7191(5)Å	a= 68.128(11)°.
	b = 12.4557(10)Å	b = 79.019(9)°.
	c = 12.803(2)Å	g = 77.481(7)°.
Volume	963.6(2) $Å^3$	
Z	2	
Density (calculated)	1.462 Mg/m^3	
Absorption coefficient	0.803 mm^{-1}	
F(000)	444	
Crystal size	0.250 x 0.050 x 0.010 mm^3	
Theta range for data collection	2.881 to 28.809°.	
Index ranges	-8<=h<=9,-16<=k<=16,-16<=l<=17	
Reflections collected	10838	
Independent reflections	4201[R(int) = 0.0556]	
Completeness to theta =28.809°	83.7%	
Absorption correction	Multi-scan	
Max. and min. transmission	1.00 and 0.65	
Refinement method	Full-matrix least-squares on F^2	
Data / restraints / parameters	4201/ 606/ 517	
Goodness-of-fit on F^2	1.024	
Final R indices [I>2sigma(I)]	R1 = 0.0543, wR2 = 0.1340	
R indices (all data)	R1 = 0.0833, wR2 = 0.1473	
Largest diff. peak and hole	1.215 and -0.463 e.$Å^{-3}$	

11.2 (±)-1-((1*R*,2*R*,5*S*)-Bicyclo[3.2.2]nona-3,6-dien-2-yl)-4-((1S,2S,5R)-bicyclo[3.2.2]nona-3,6-dien-2-yl)benzene (3v)

The single crystals of compound **3v** suitable for X-ray diffraction analysis were obtained by evaporation of a solution of **3v** in pentane. The crystal structure information for this compound has been deposited at the Cambridge Crystallographic Data Centre. CCDC2027380 contains the crystal structure information of this compound and can be obtained free of charge via http://www.ccdc.cam.ac.uk.

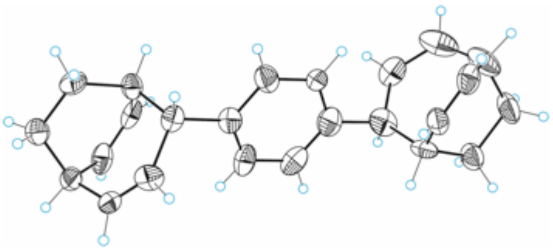

Identification code	mm-2-216	
Empirical formula	C24 H26	
Formula weight	314.45	
Temperature	100(2)K	
Wavelength	0.71073 Å	
Crystal system	monoclinic	
Space group	P 21	
Unit cell dimensions	a = 6.3048(5)Å	a= 90°.
	b = 11.2915(5)Å	b = 98.845(6)°.
	c = 12.2779(7)Å	g = 90°.
Volume	863.68(9) $Å^3$	
Z	2	
Density (calculated)	1.209 Mg/m^3	
Absorption coefficient	0.068 mm^{-1}	
F(000)	340	
Crystal size	0.150 x 0.150 x 0.030 mm^3	
Theta range for data collection	2.464 to 28.701°.	
Index ranges	-7<=h<=8,-13<=k<=14,-14<=l<=16	
Reflections collected	9489	
Independent reflections	3418[R(int) = 0.0242]	
Completeness to theta =28.701°	87.1%	
Absorption correction	Multi-scan	
Max. and min. transmission	1.00 and 0.85	
Refinement method	Full-matrix least-squares on F^2	
Data / restraints / parameters	3418/ 454/ 397	
Goodness-of-fit on F^2	1.839	
Final R indices [I>2sigma(I)]	R1 = 0.0798, wR2 = 0.2286	
R indices (all data)	R1 = 0.0926, wR2 = 0.2382	
Flack parameter	x =2.4(10)	
Largest diff. peak and hole	0.437 and -0.224 e.$Å^{-3}$	

11.3 (±)-4-((1*S*,6*R*,7*R*,*Z*)-Bicyclo[4.3.2]undeca-8,10-dien-7-yl)phenyl ferrocenoylate (3y)

The single crystals of compound **3y** suitable for X-ray diffraction analysis were obtained by slow evaporation of a solution of **3y** in 9:1 MeCN/CH_2Cl_2 over 2 days. The crystal structure information for this compound has been deposited at the Cambridge Crystallographic Data Centre. CCDC2027383 contains the crystal structure information of this compound and can be obtained free of charge via http://www.ccdc.cam.ac.uk.

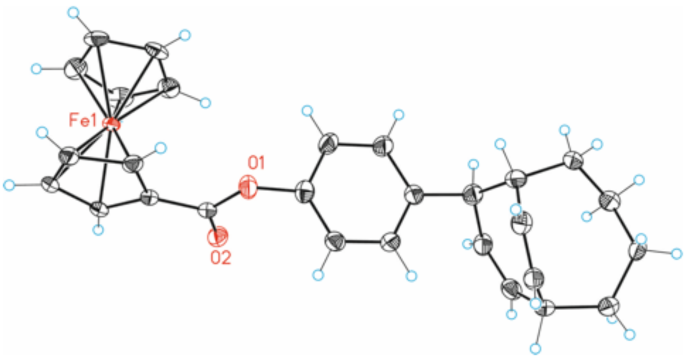

Identification code	MM-2-245_hklf5	
Empirical formula	C28 H28 Fe O2	
Formula weight	452.35	
Temperature	100(2)K	
Wavelength	0.71073 Å	
Crystal system	triclinic	
Space group	P -1	
Unit cell dimensions	a = 7.1763(2)Å	a= 79.583(3)°.
	b = 12.3501(4)Å	b = 83.597(3)°.
	c = 12.5306(6)Å	g = 86.888(3)°.
Volume	1084.78(7) $Å^3$	
Z	2	
Density (calculated)	1.385 Mg/m^3	
Absorption coefficient	0.718 mm^{-1}	
F(000)	476	
Crystal size	0.150 x 0.120 x 0.030 mm^3	
Theta range for data collection	2.561 to 31.963°.	
Index ranges	-10<=h<=10,-17<=k<=17,-18<=l<=18	
Reflections collected	15519	
Independent reflections	15519[R(int) = ?]	
Completeness to theta =31.963°	89.6%	
Absorption correction	Multi-scan	
Max. and min. transmission	1.00 and 0.66	
Refinement method	Full-matrix least-squares on F^2	
Data / restraints / parameters	15519/ 0/ 282	
Goodness-of-fit on F^2	2.005	
Final R indices [I>2sigma(I)]	R1 = 0.0801, wR2 = 0.2685	
R indices (all data)	R1 = 0.0906, wR2 = 0.2729	
Largest diff. peak and hole	1.781 and -1.130 e.$Å^{-3}$	

11.4 (±)-2-((1*S*,3*R*)-3,6-Dimethyl-1,2,3,3,4,7-hexahydroazulen-1-yl)propan-2-ol (7fa)

The single crystals of compound **7fa** suitable for X-ray diffraction analysis were obtained by slow evaporation of a solution of **7fa** in ethyl acetate. The crystal structure information for this compound has been deposited at the Cambridge Crystallographic Data Centre. CCDC2027381 contains the crystal structure information of this compound and can be obtained free of charge via http://www.ccdc.cam.ac.uk.

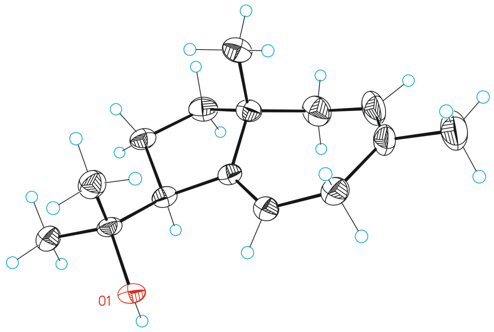

Identification code	HA6336_a	
Empirical formula	C15 H24 O	
Formula weight	220.34	
Temperature	100(2)K	
Wavelength	0.71073 Å	
Crystal system	orthorhombic	
Space group	P b c a	
Unit cell dimensions	a = 20.295(9)Å	a= 90°.
	b = 6.003(3)Å	b = 90°.
	c = 21.548(10)Å	g = 90°.
Volume	2625(2) Å^3	
Z	8	
Density (calculated)	1.115 Mg/m^3	
Absorption coefficient	0.067 mm^{-1}	
F(000)	976	
Crystal size	0.150 x 0.030 x 0.010 mm^3	
Theta range for data collection	2.007 to 23.284°.	
Index ranges	-22<=h<=22,-6<=k<=6,-23<=l<=23	
Reflections collected	13303	
Independent reflections	1895[R(int) = 0.1253]	
Completeness to theta =23.284°	99.9%	
Absorption correction	Multi-scan	
Max. and min. transmission	0.74 and 0.60	
Refinement method	Full-matrix least-squares on F^2	
Data / restraints / parameters	1895/ 0/ 149	
Goodness-of-fit on F^2	1.072	
Final R indices [I>2sigma(I)]	R1 = 0.0523, wR2 = 0.1134	
R indices (all data)	R1 = 0.1061, wR2 = 0.1330	
Largest diff. peak and hole	0.198 and -0.193 e.Å^{-3}	

12. NMR Spectra

Ethyl 2,4,6-trimethylcyclohepta-2,4,6-triene-1-carboxylate (S1)

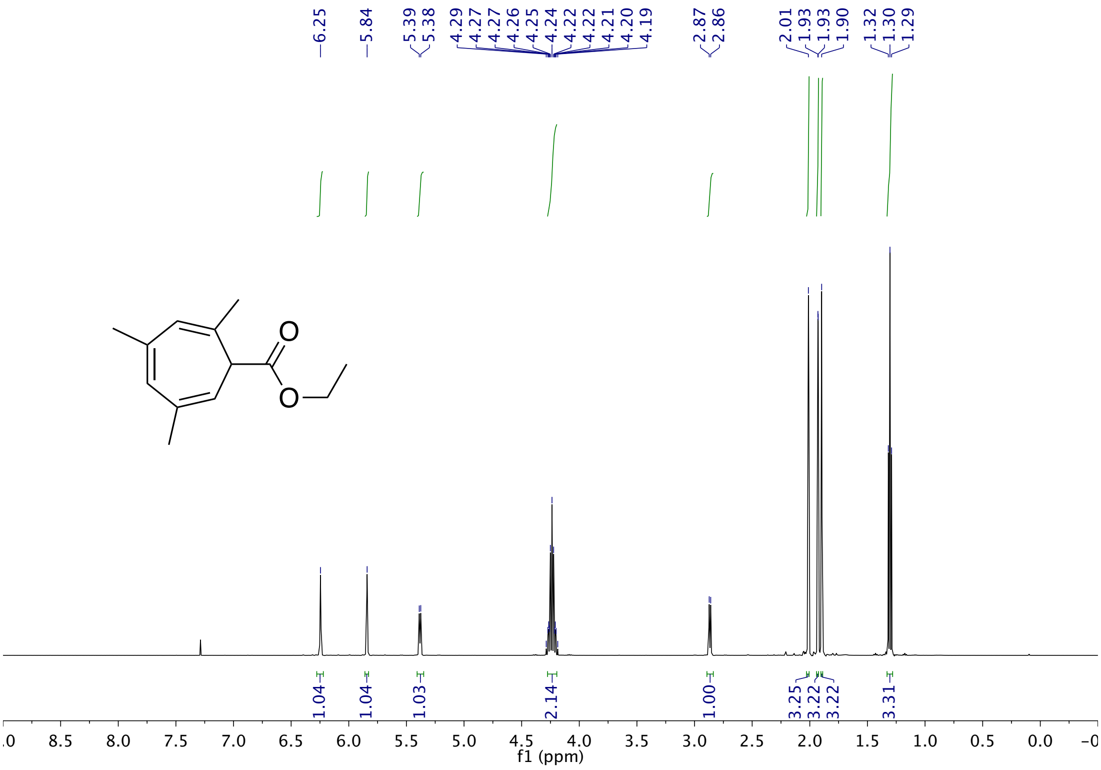

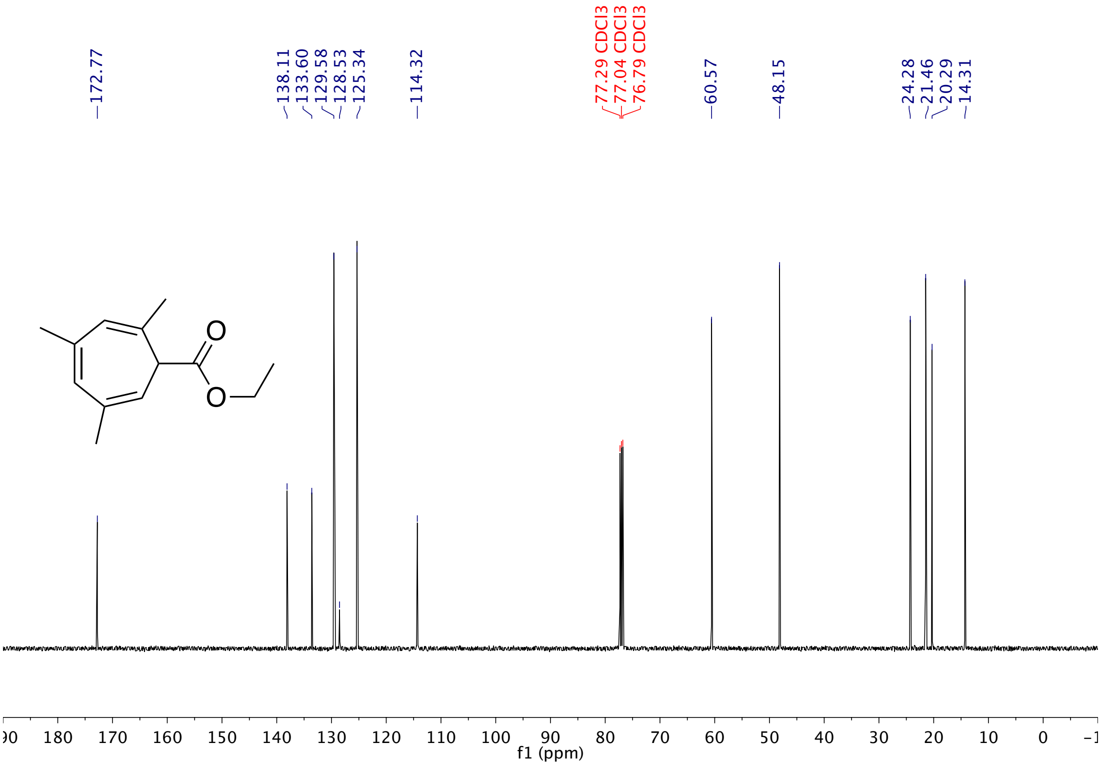

(2,4,6-Trimethylcyclohepta-2,4,6-trien-1-yl)methanol (S2)

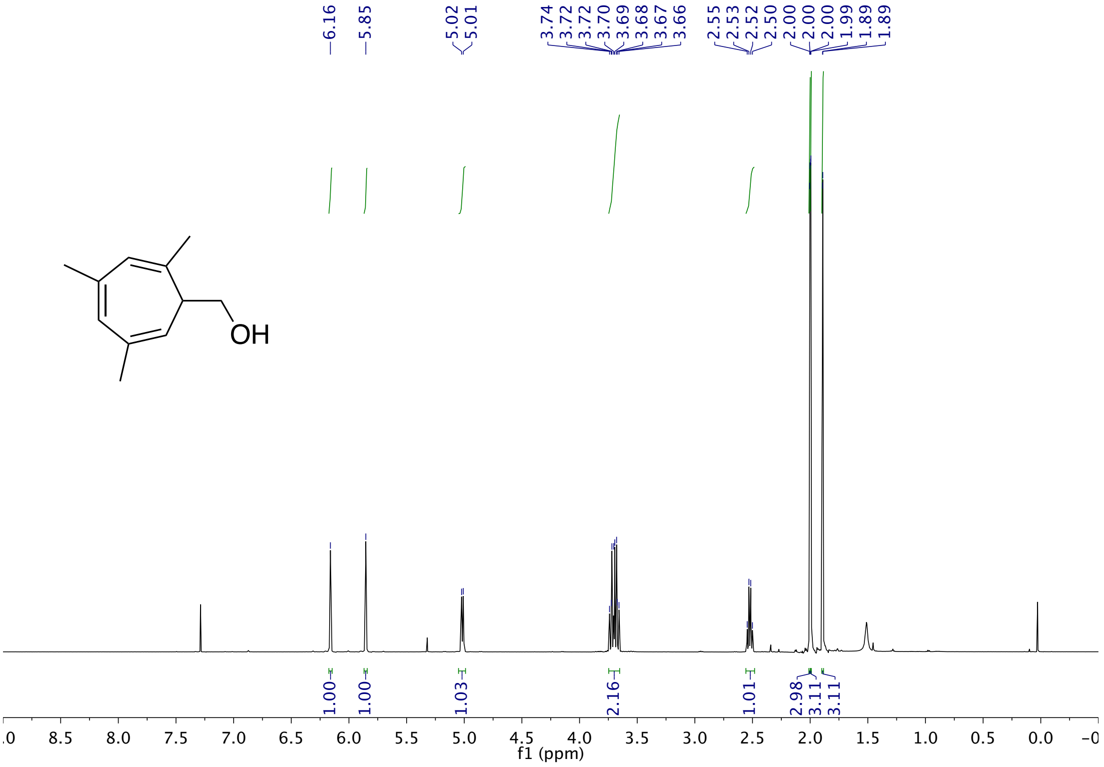

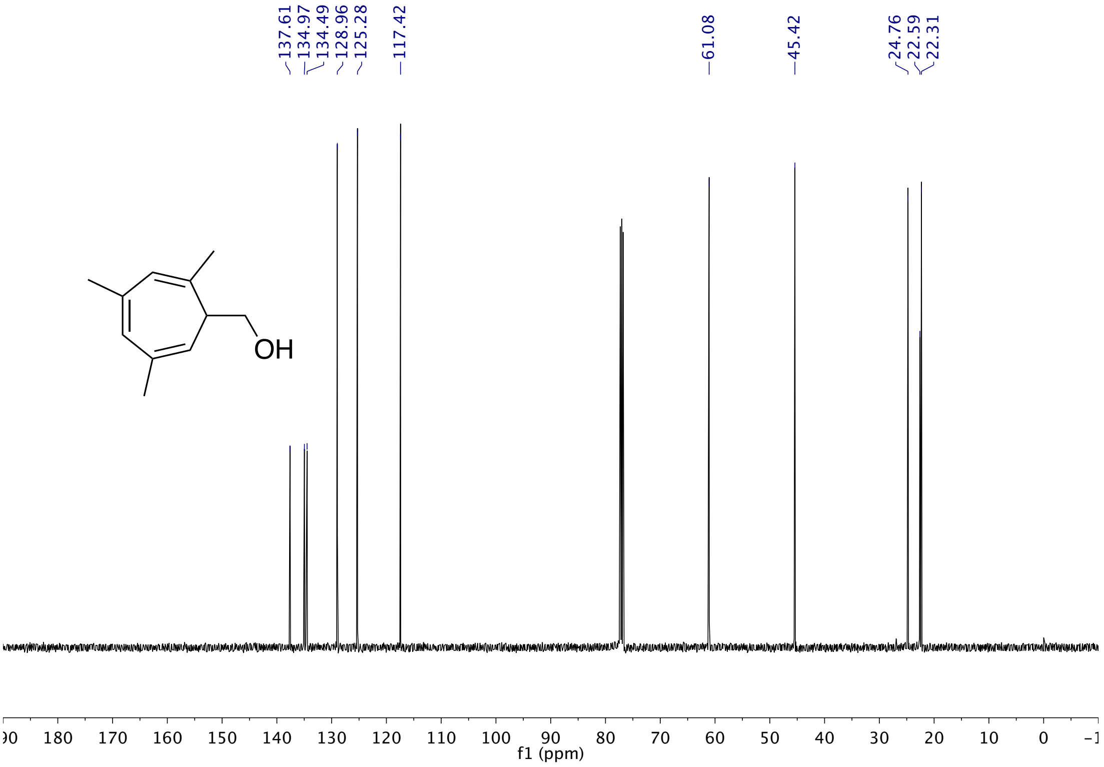

1-Phenyl-5-(((2,4,6-trimethylcyclohepta-2,4,6-trien-1-yl)methyl)thio)-1*H*-tetrazole (S3)

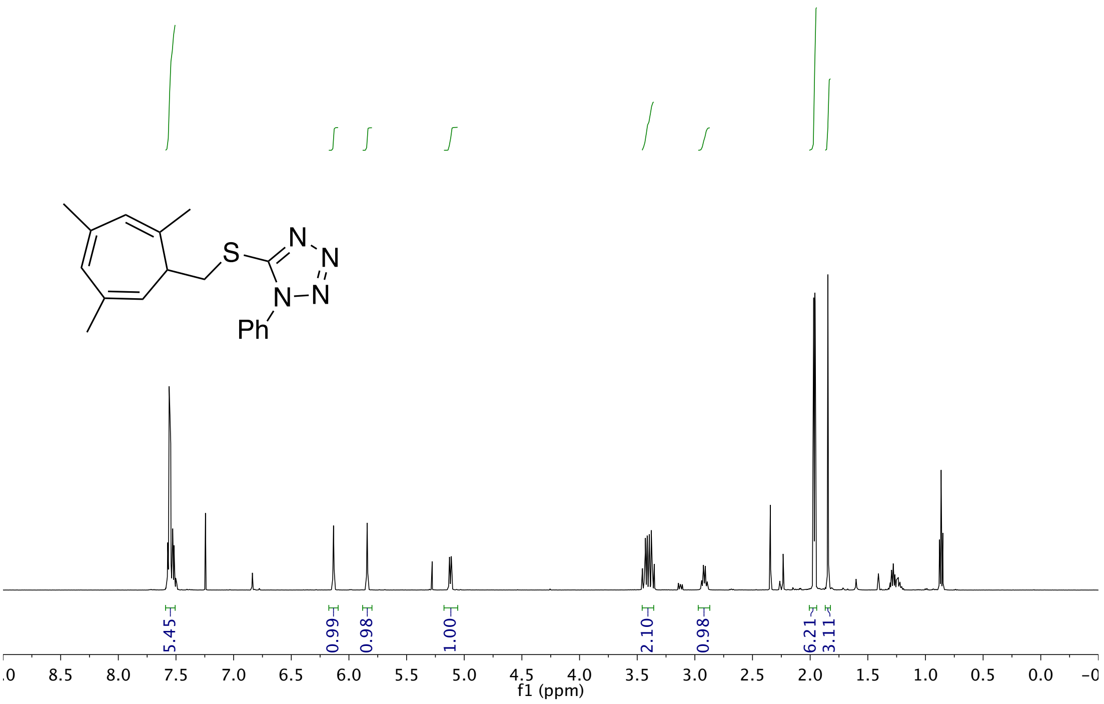

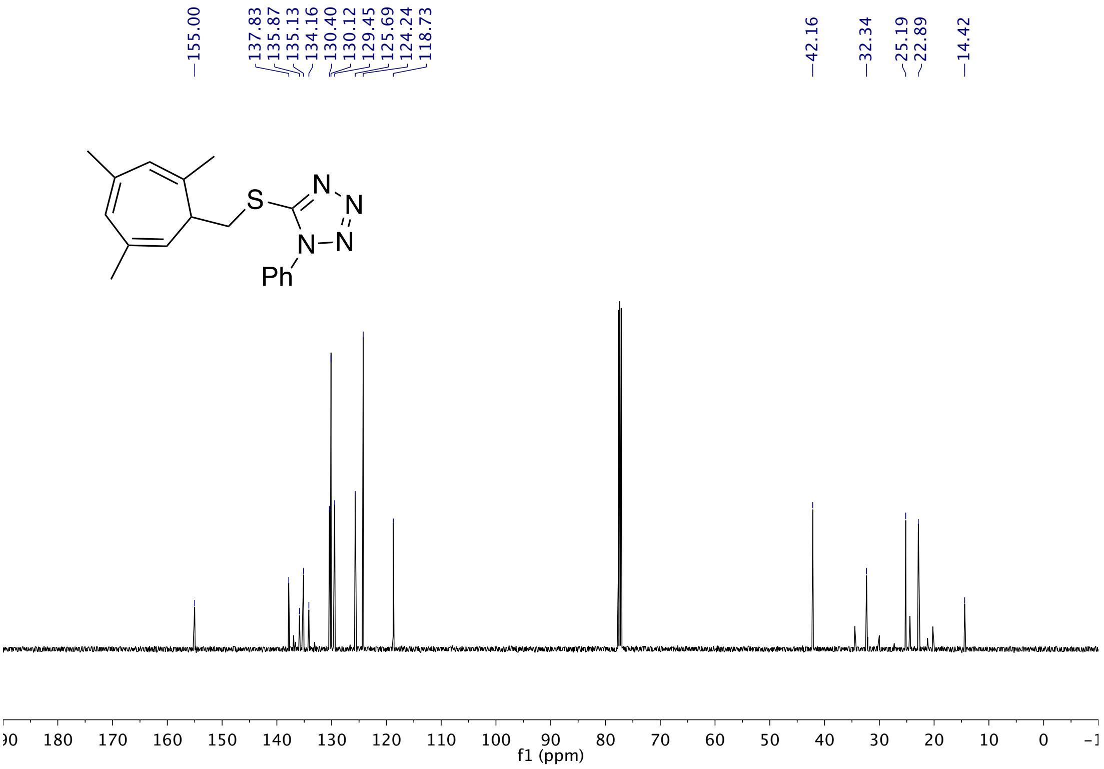

1-Phenyl-5-(((2,4,6-trimethylcyclohepta-2,4,6-trien-1-yl)methyl)sulfonyl)-1*H*-tetrazole (S4)

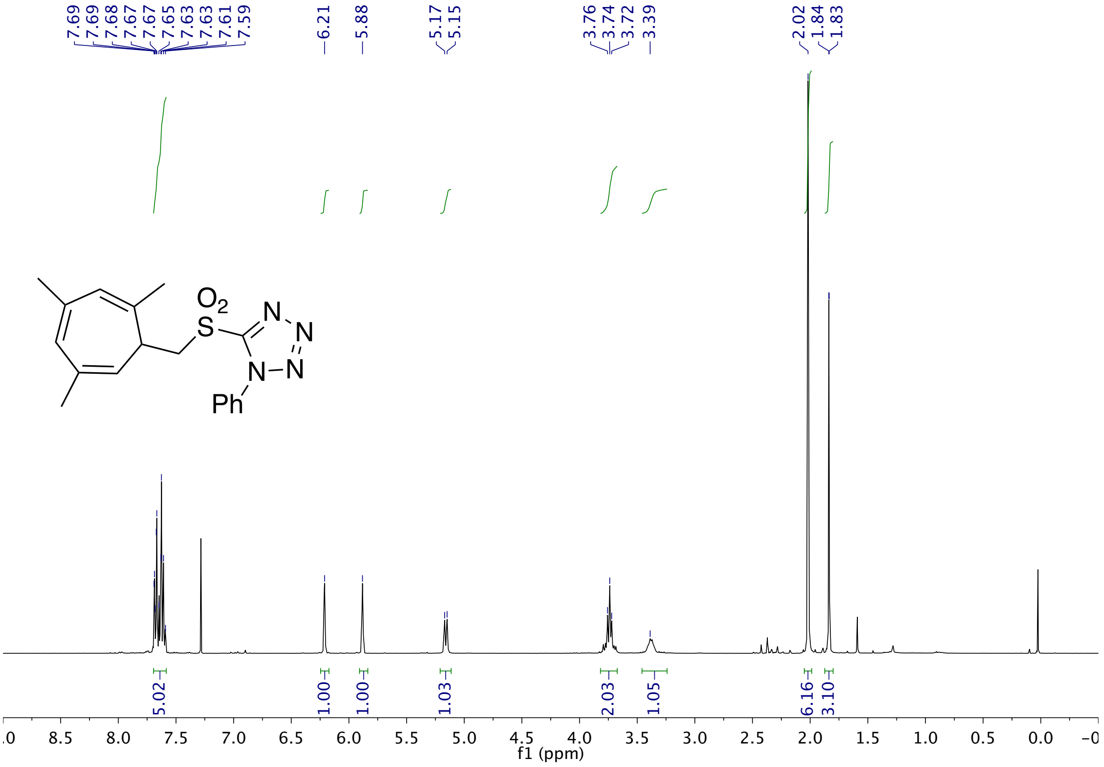

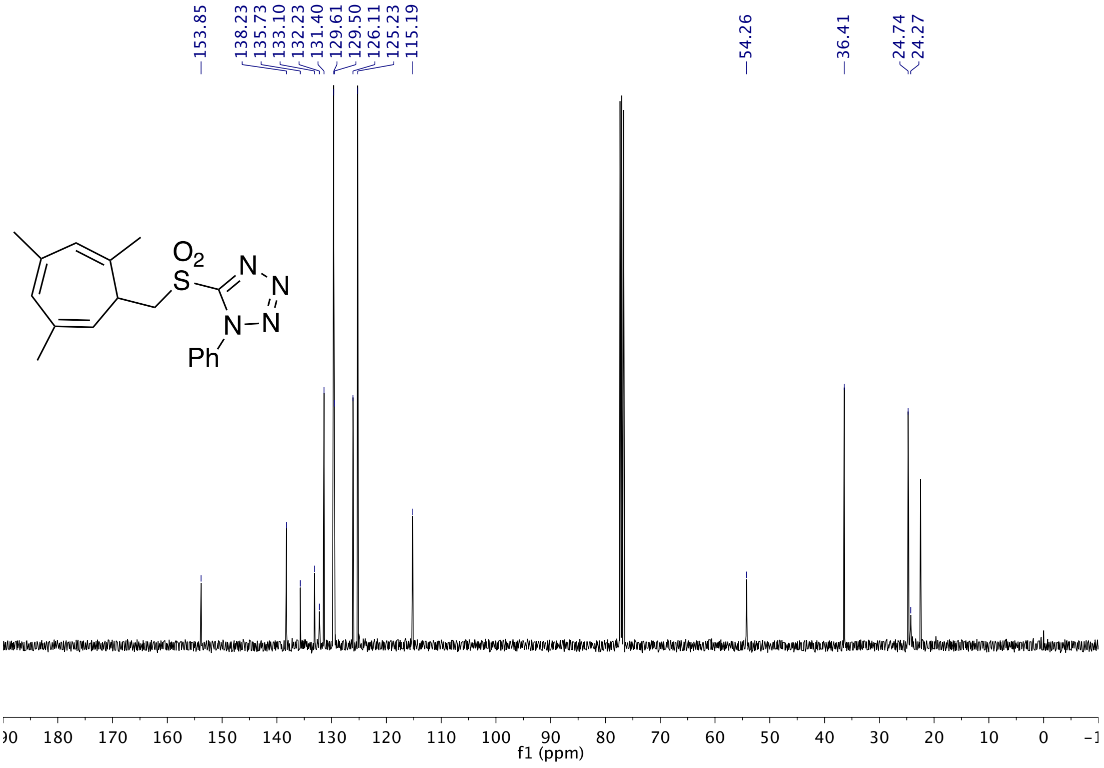

(*E*)-1,3,5-Trimethyl-7-styrylcyclohepta-1,3,5-triene (1a)

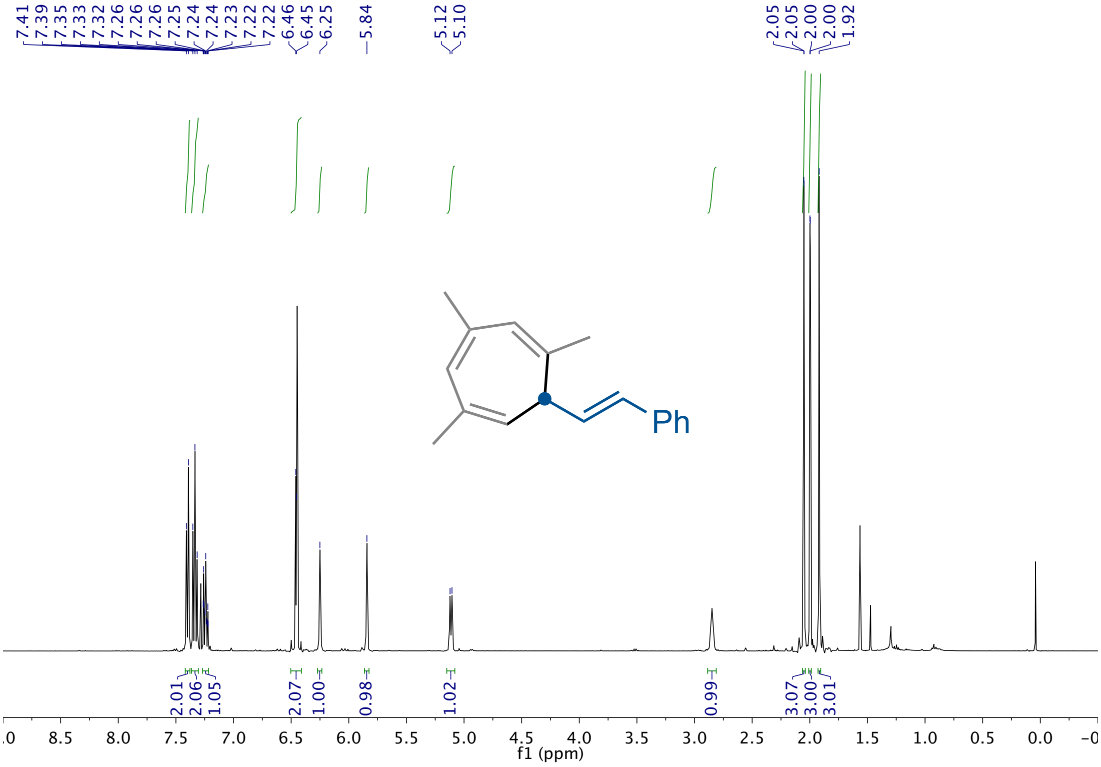

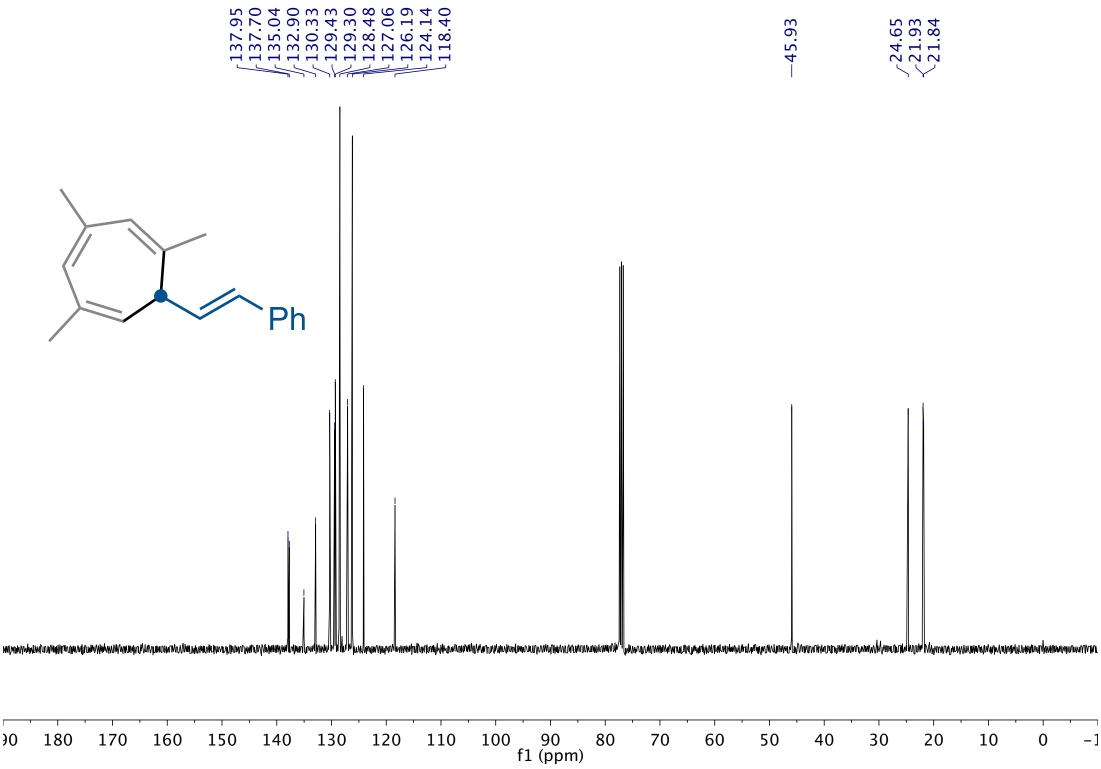

(*E*)-1,3,5-Trimethyl-7-(hex-1-en-1-yl)cyclohepta-1,3,5-triene (1b) (1:0.6 CHT/NCD)

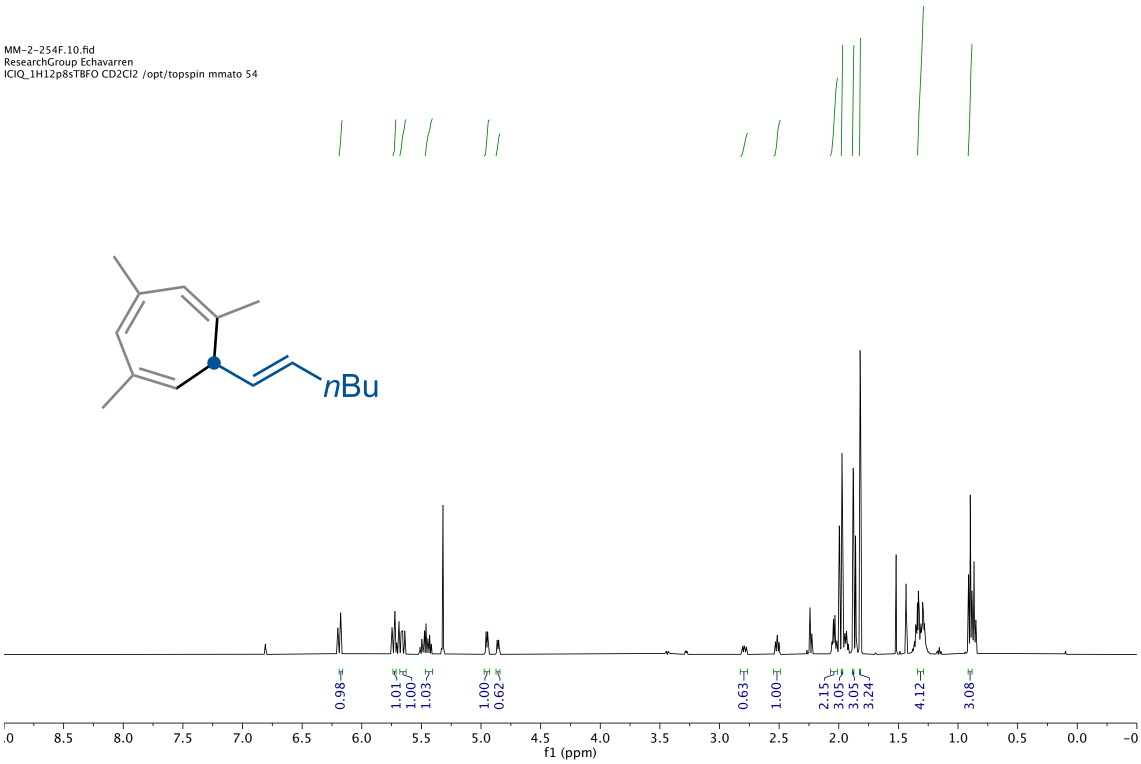

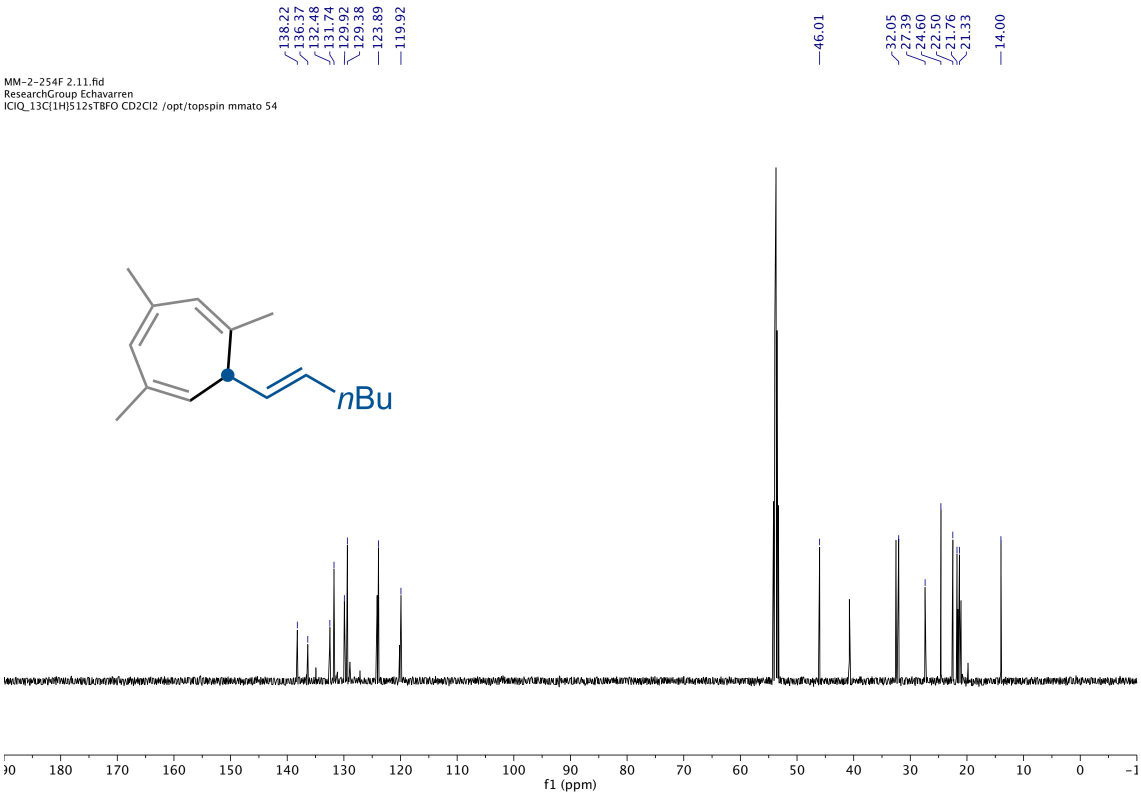

(*E*)-4-(2-(2,4,6-trimethylcyclohepta-2,4,6-trien-1-yl)vinyl)phenyl ferrocenecarboxylate (1c)

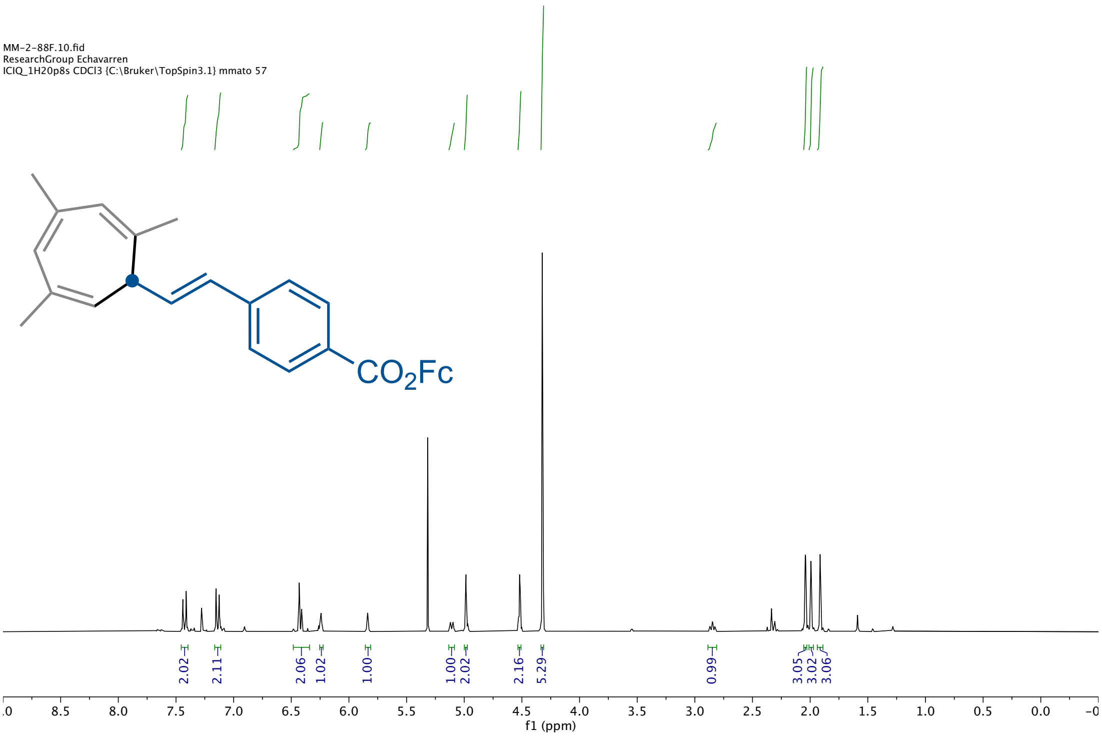

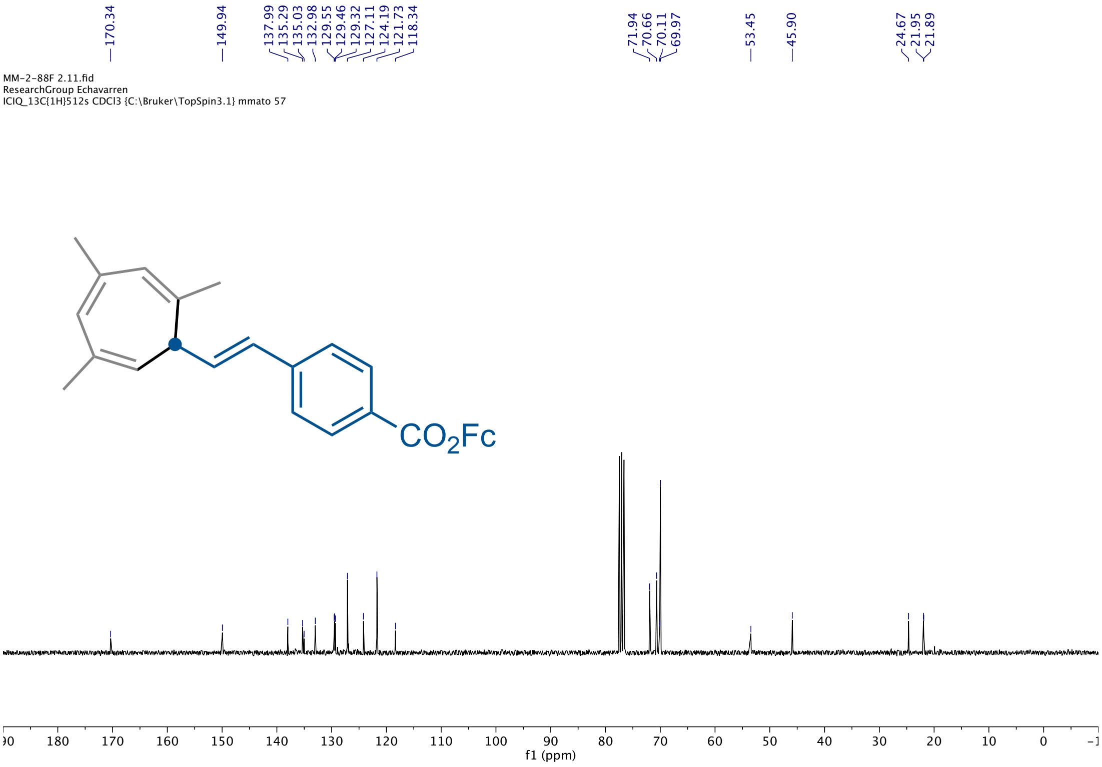

(*E*)-7-(3,5-Bis(trifluoromethyl)styryl)-1,3,5-trimethylcyclohepta-1,3,5-triene (1d)

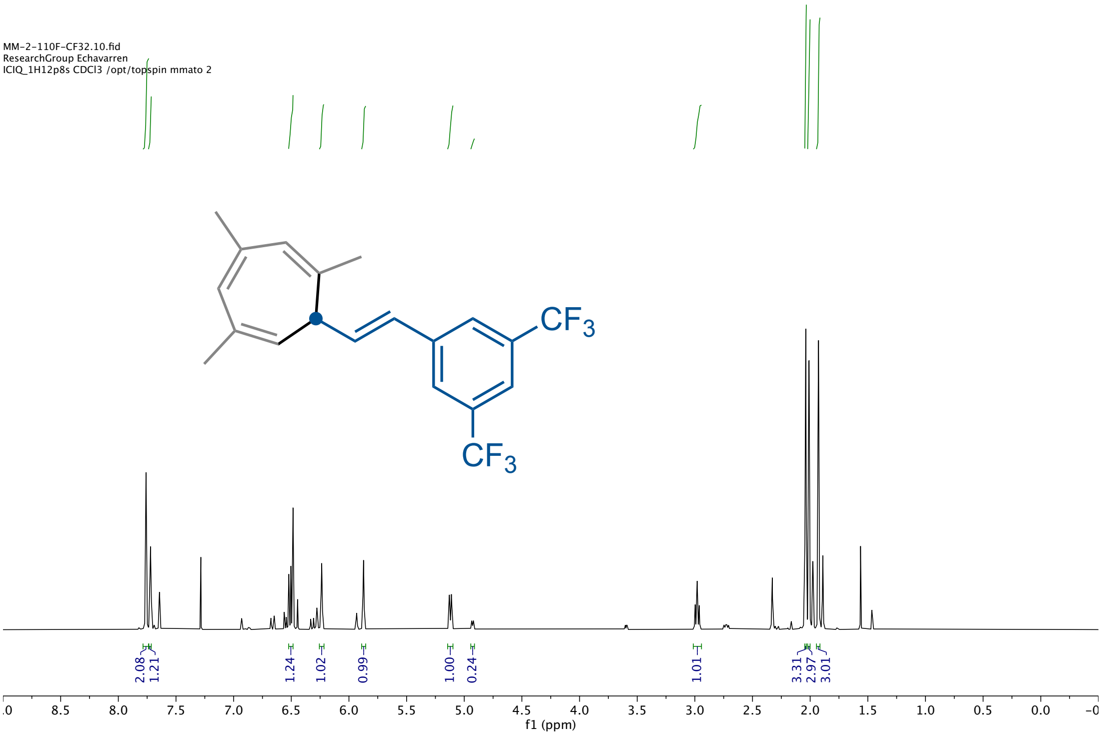

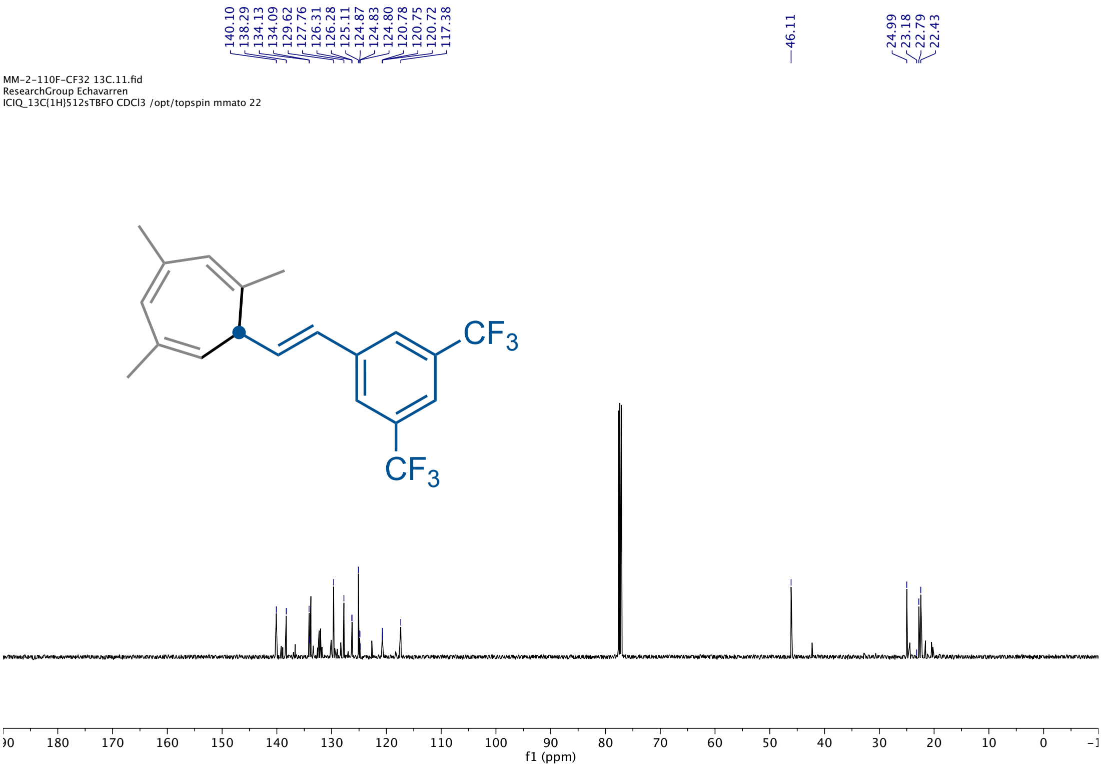

MM-2-110F-CF32 19F.12.fid
ResearchGroup Echavarren
ICIQ_19F{1H} CDCl3 /opt/topspin mmato 2

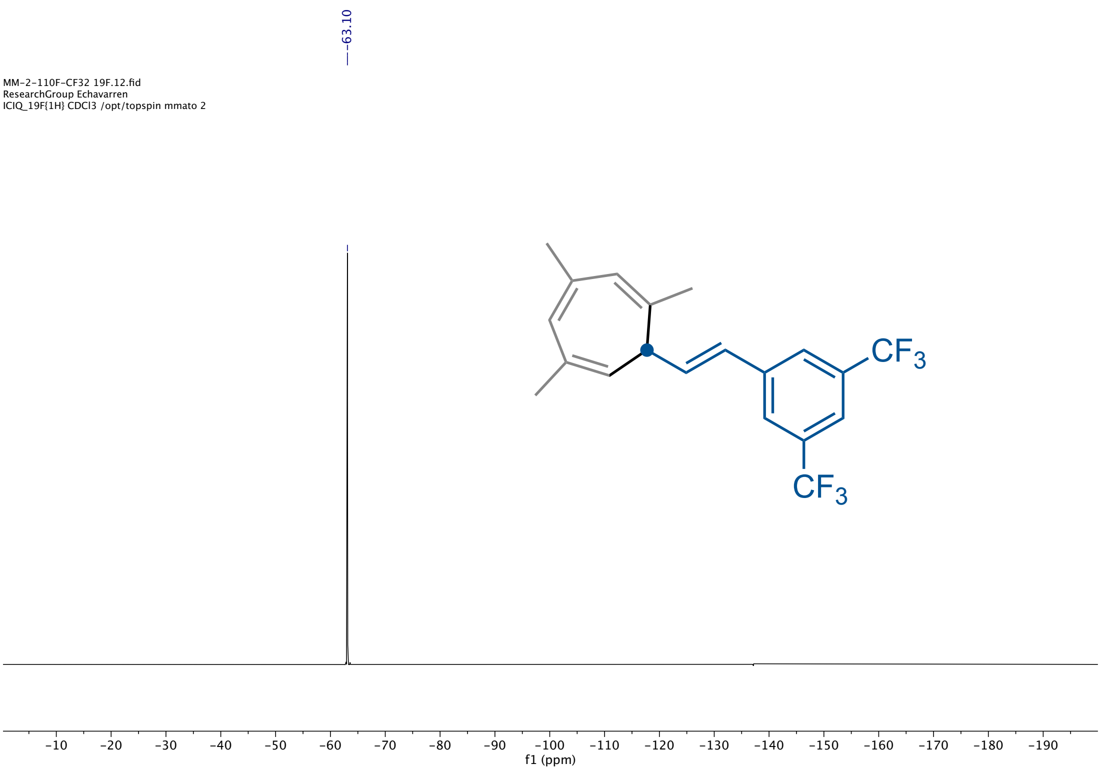

(*E*)-7-(4-Methoxystyryl)-1,3,5-trimethylcyclohepta-1,3,5-triene (1e)

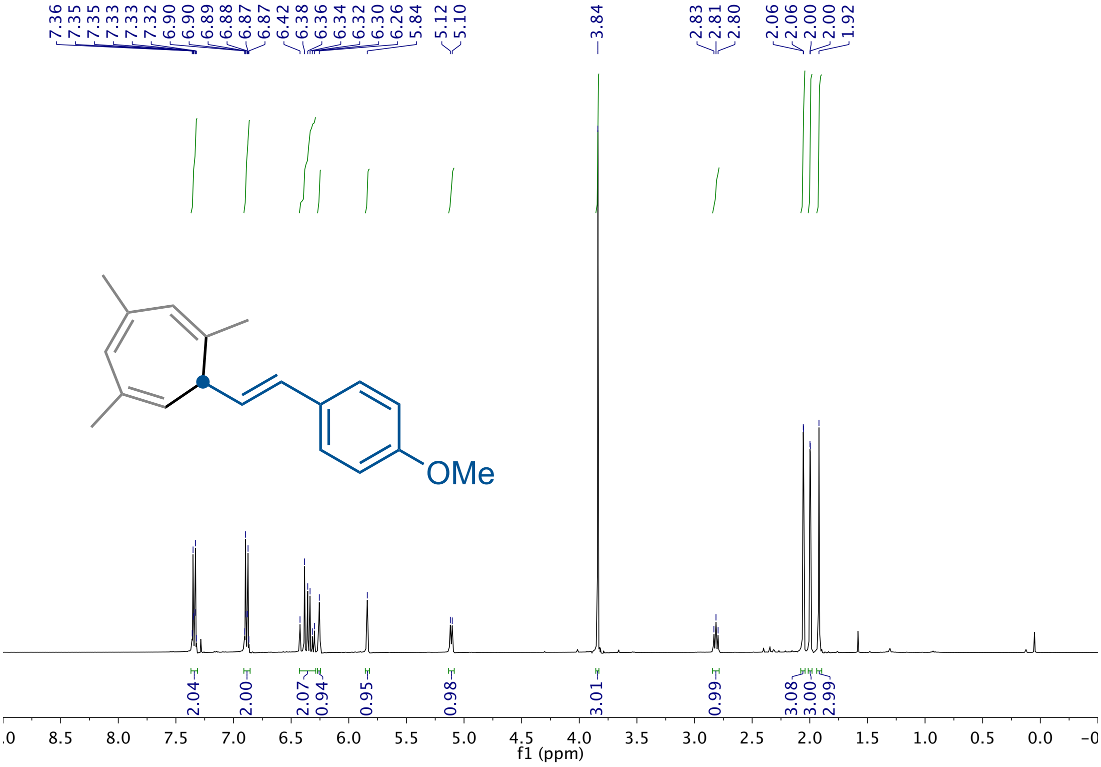

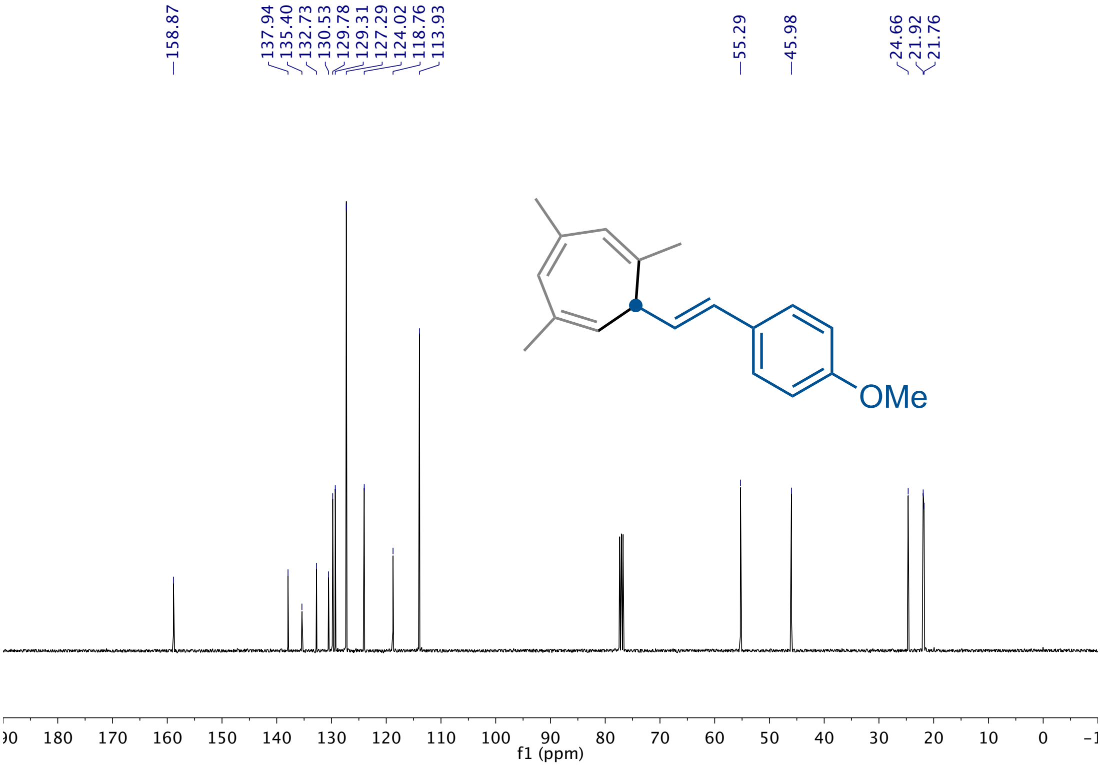

(*E*)-1,3,5-Trimethyl-7-(3,5-dichlorostyryl)cyclohepta-1,3,5-triene (1f)

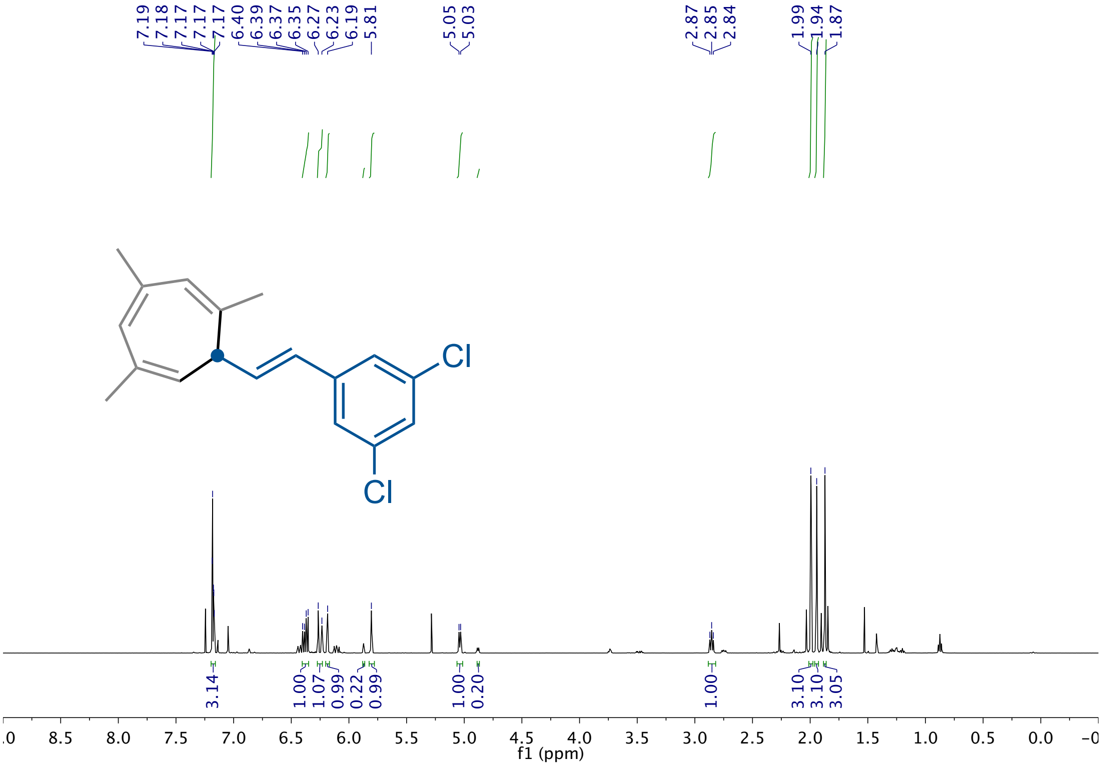

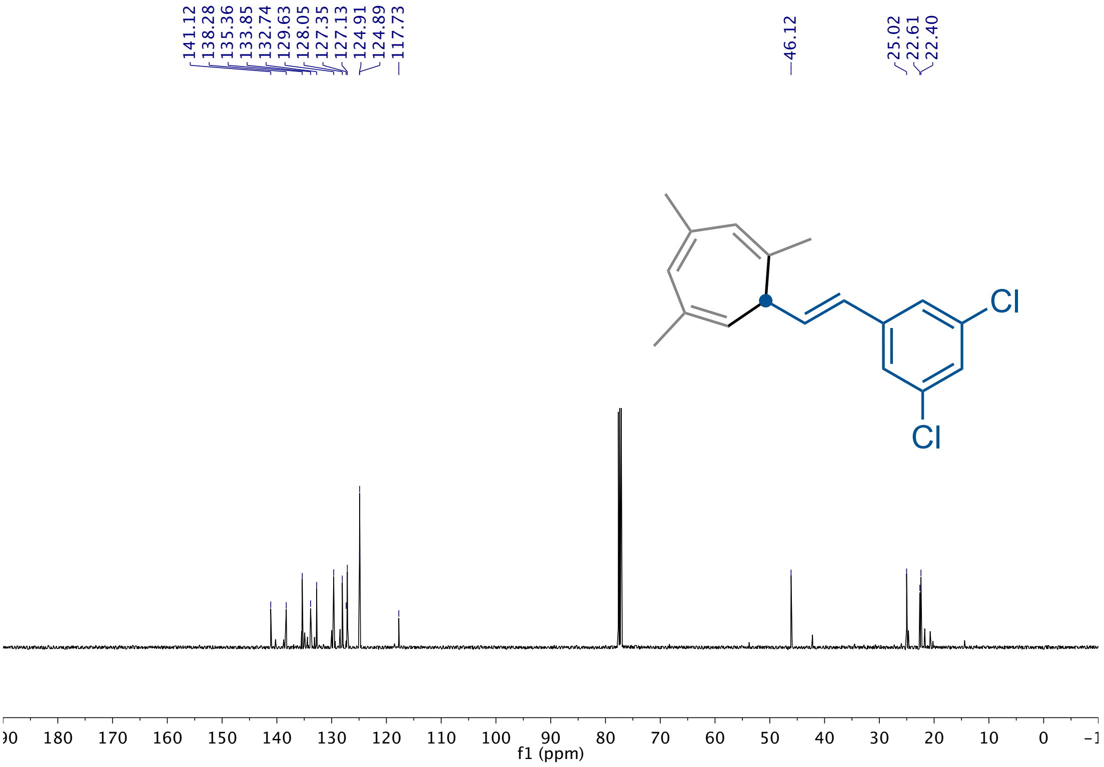

7-((1*E*,3*E*)-4-(4-Methoxyphenyl)buta-1,3-dien-1-yl)-1,3,5-trimethylcyclohepta-1,3,5-triene (1g)

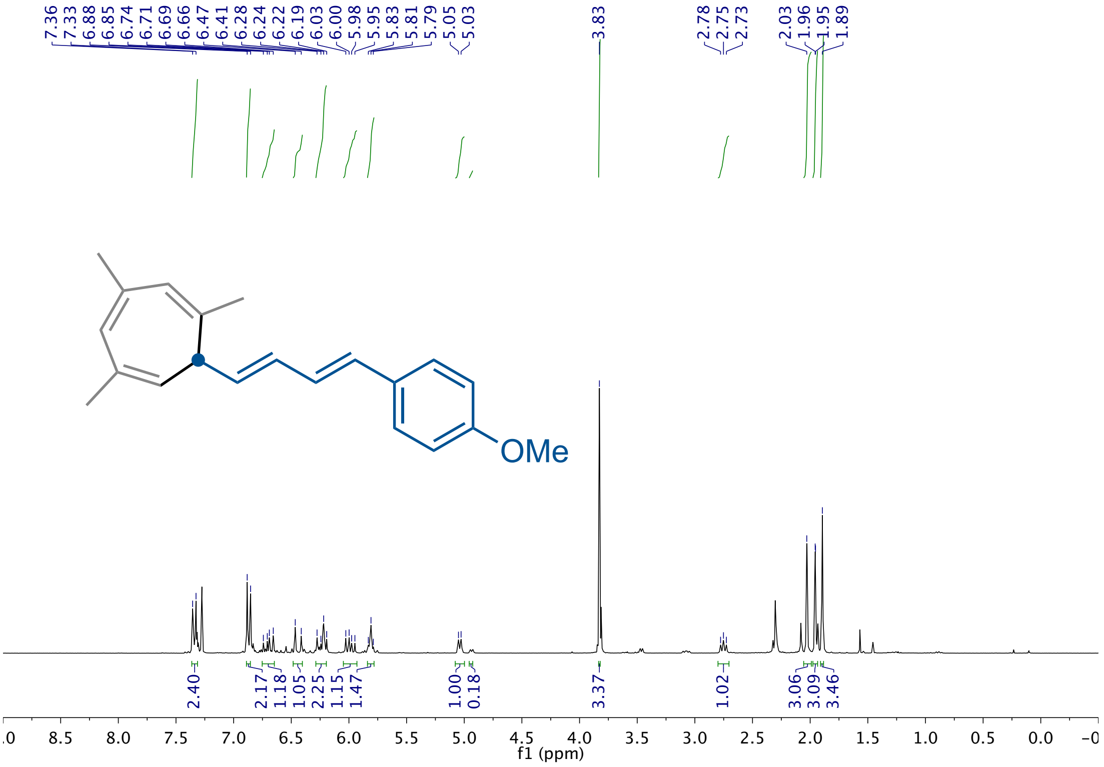

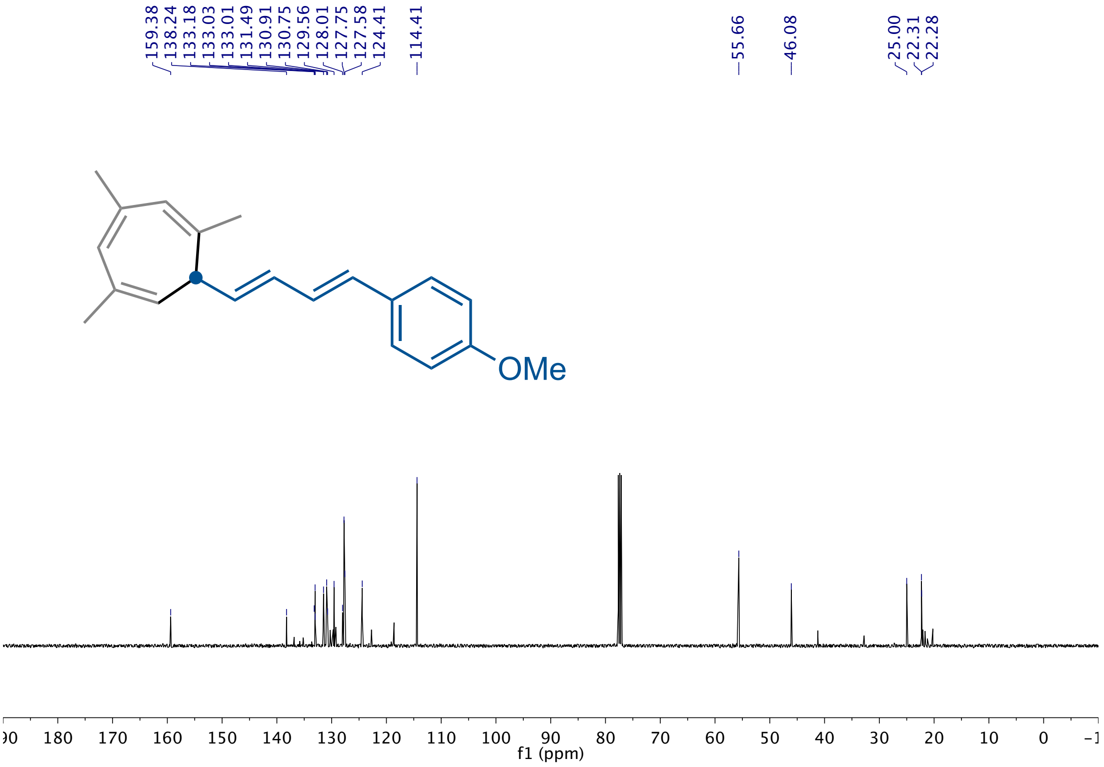

(*E*)-5-Bromo-6-(2-(2,4,6-trimethylcyclohepta-2,4,6-trien-1-yl)vinyl)benzo-[*d*][1,3]dioxole (1h)

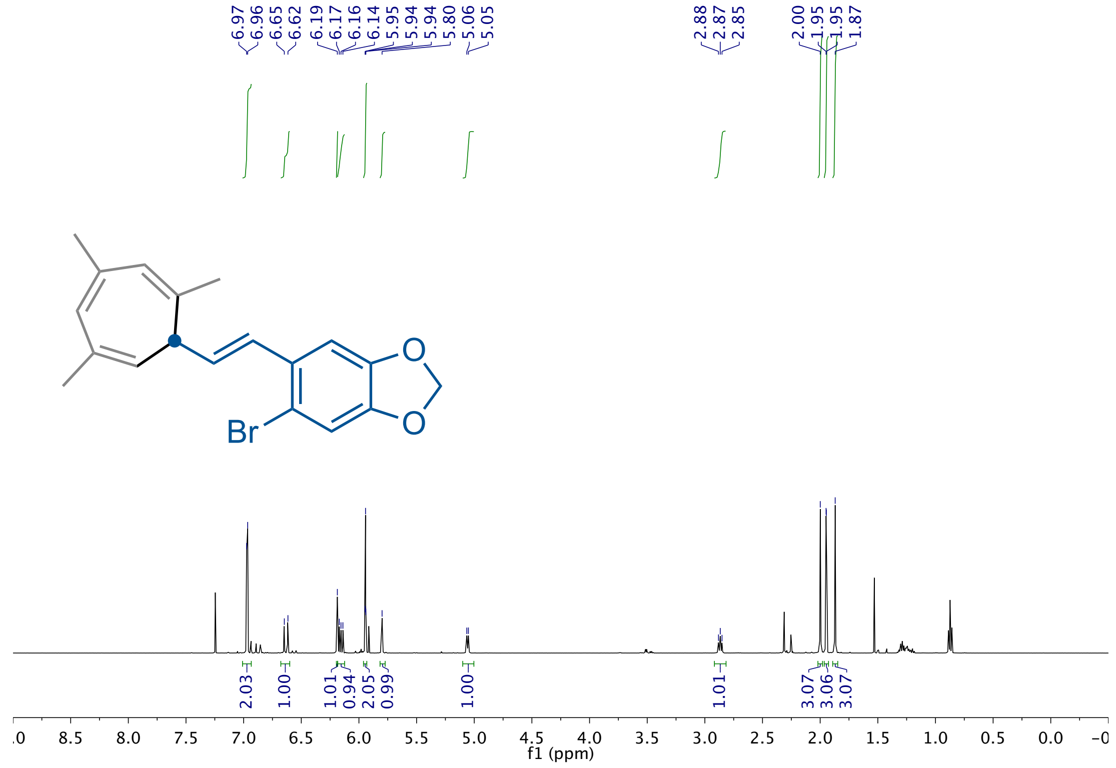

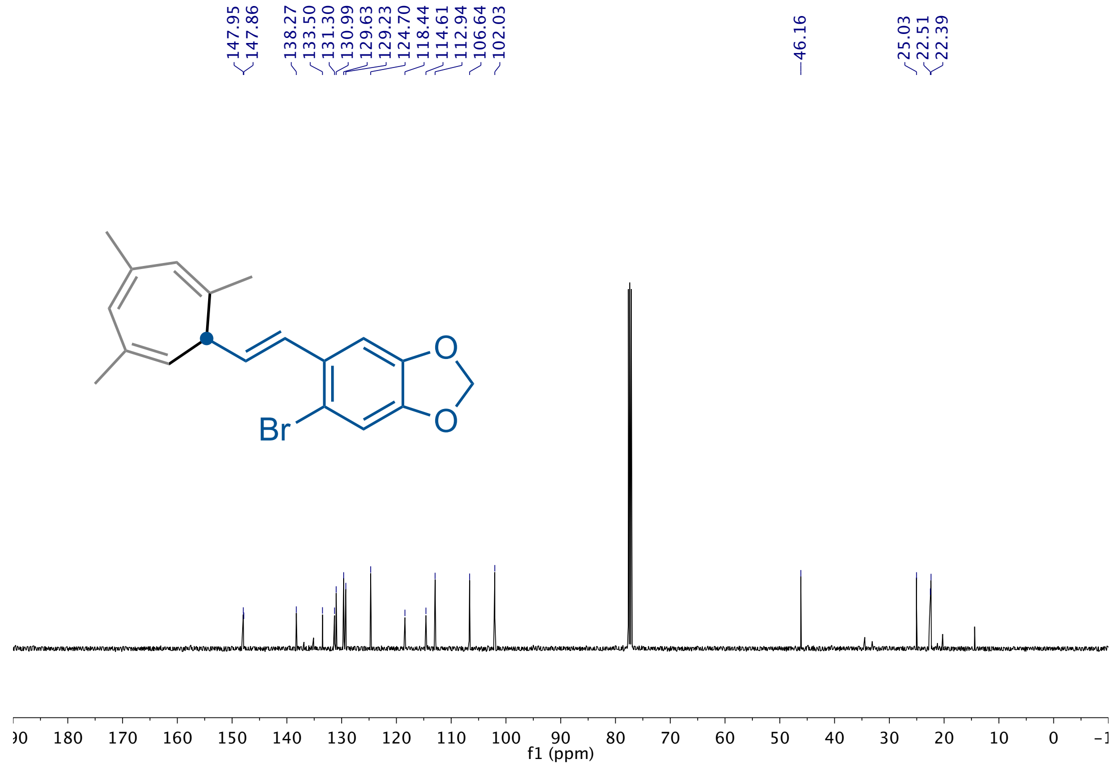

(*E*)-7-(3-Bromostyryl)-1,3,5-trimethylcyclohepta-1,3,5-triene (1i)

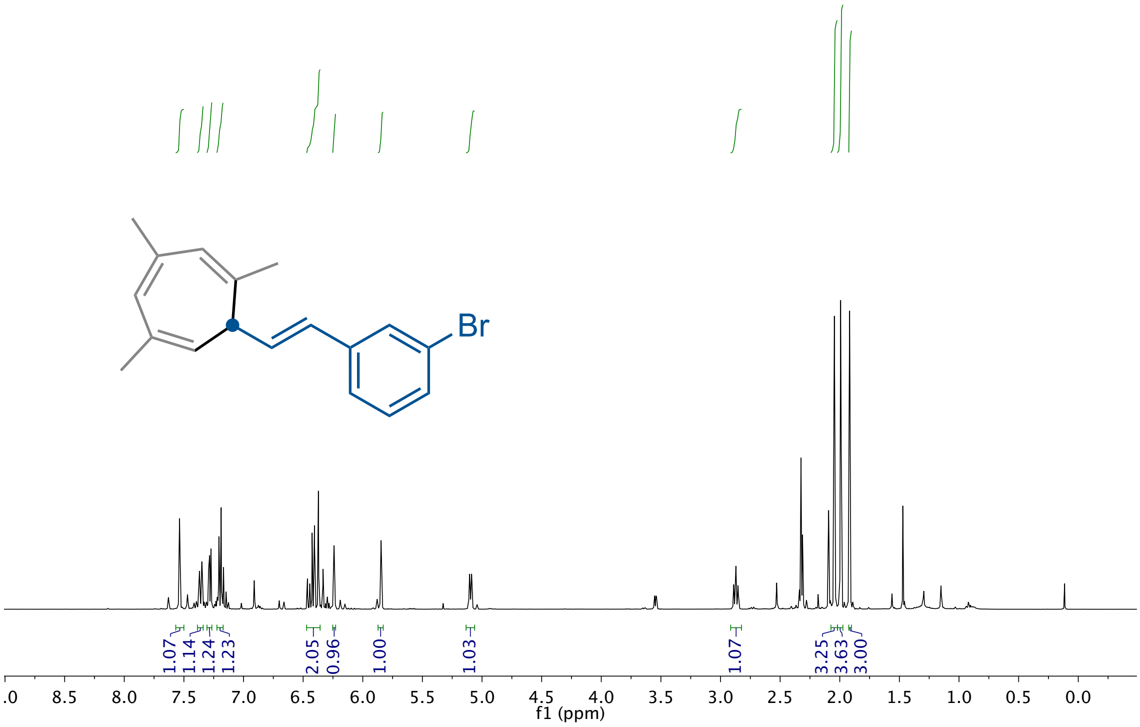

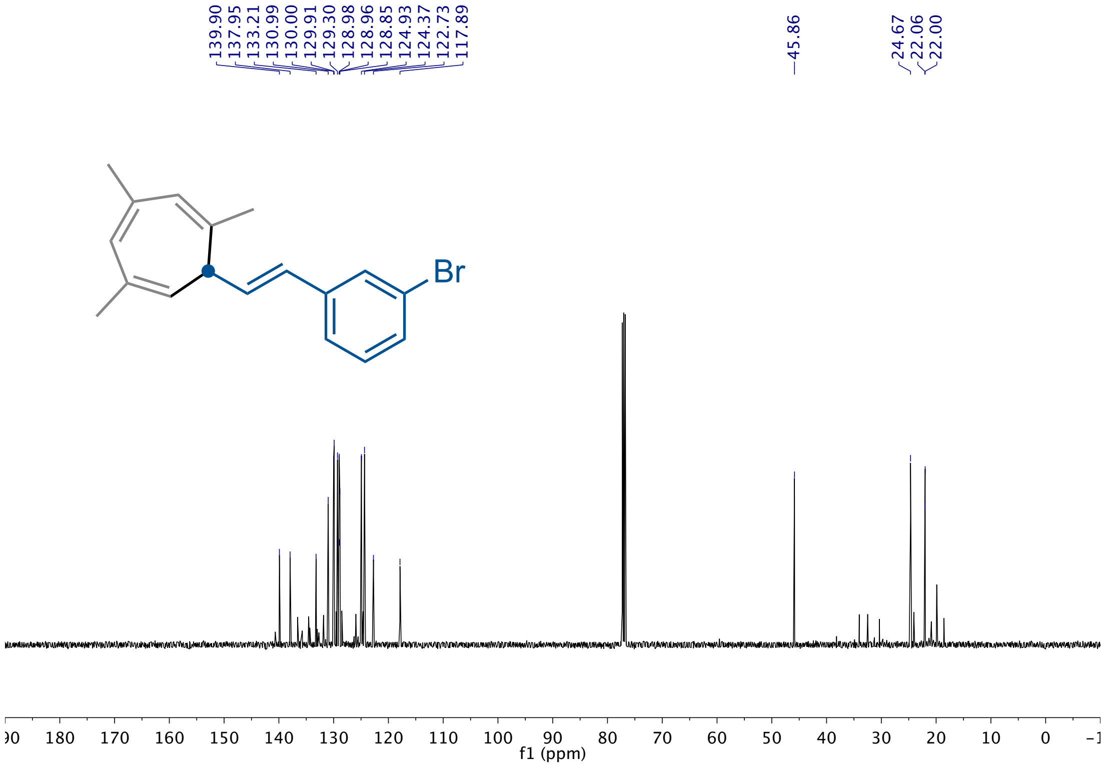

1,4-Bis((*E*)-2-(2,4,6-trimethylcyclohepta-2,4,6-trien-1-yl)vinyl)benzene (1j)

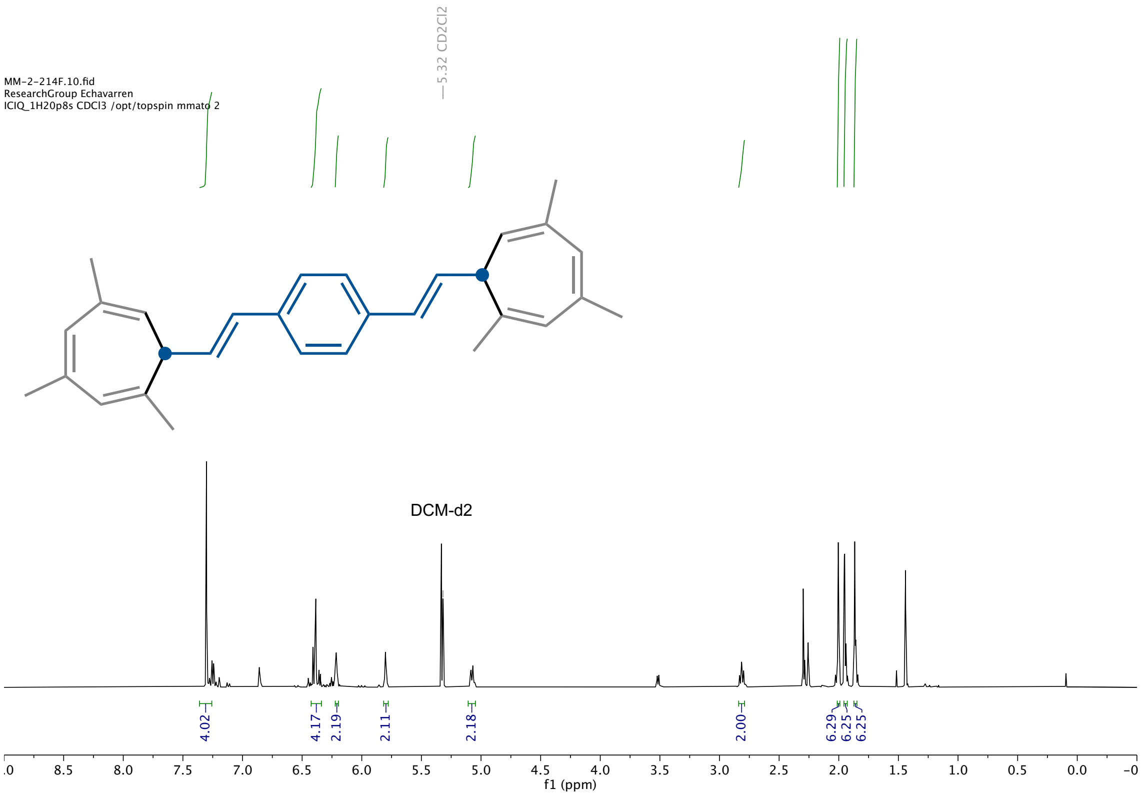

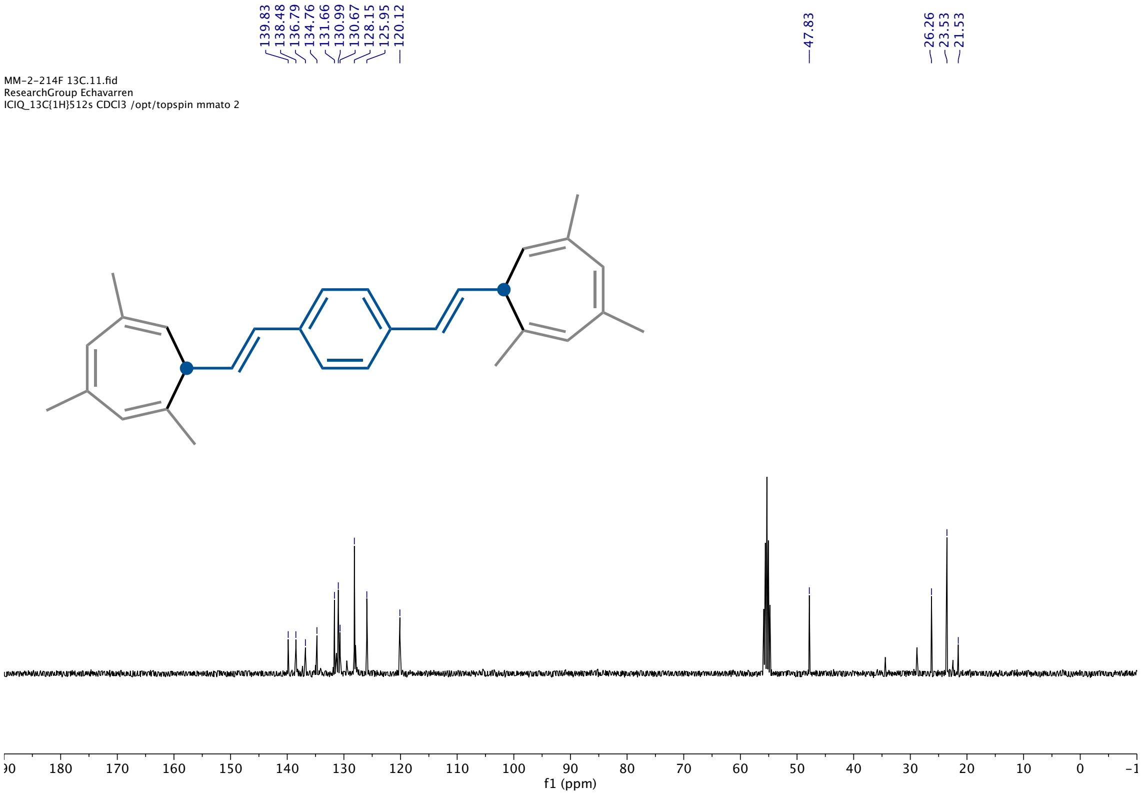

(*E*)-Trimethyl((2-(2-(2,4,6-trimethylcyclohepta-2,4,6-trien-1-yl)vinyl)phenyl)ethynyl)silane (1k)

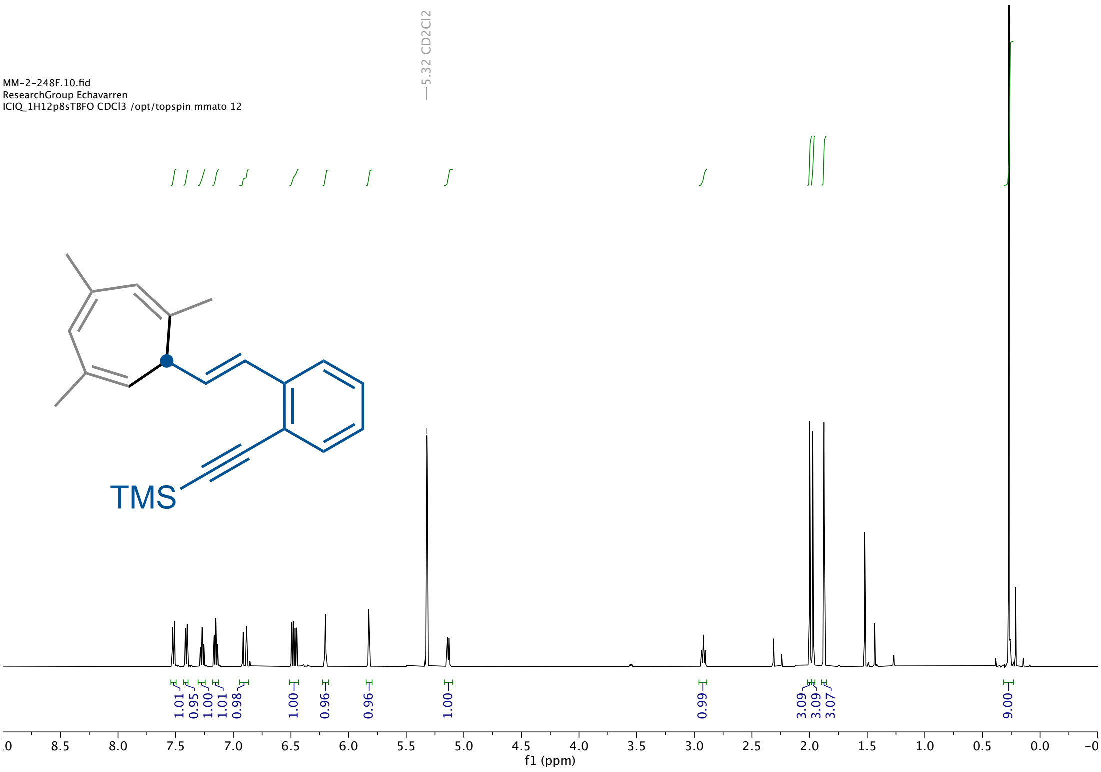

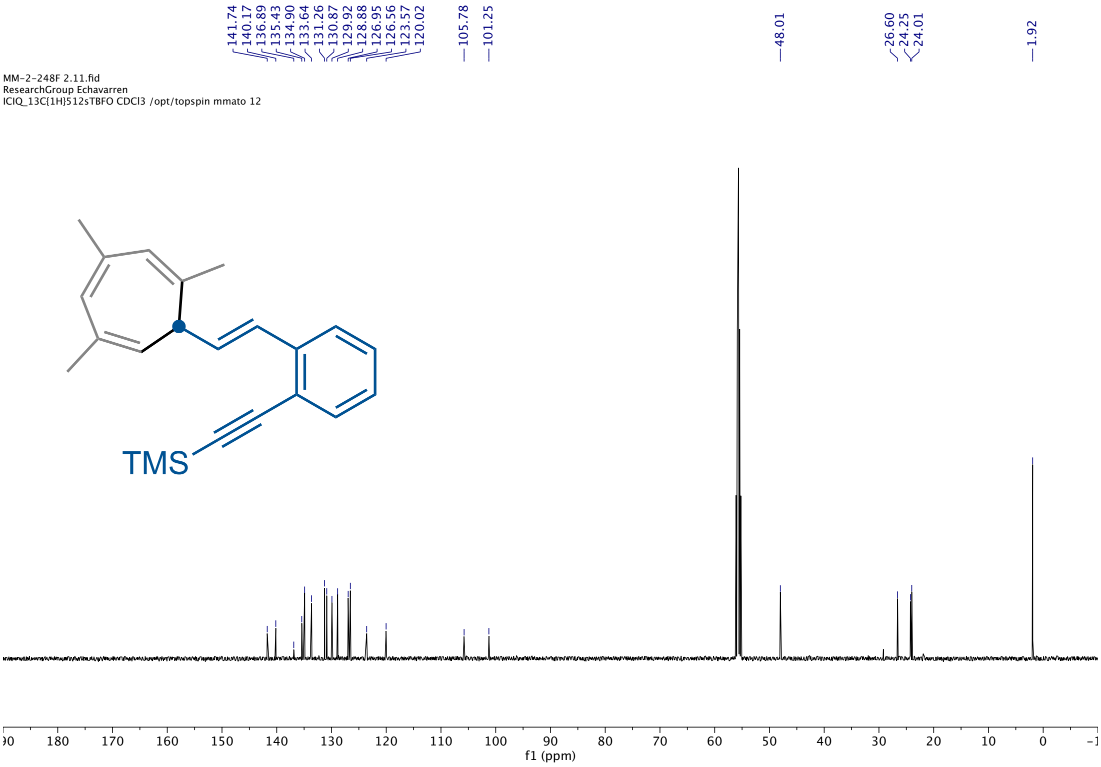

(*E*)-1,3,5-Trimethyl-7-(2-iodostyryl)cyclohepta-1,3,5-triene (1l)

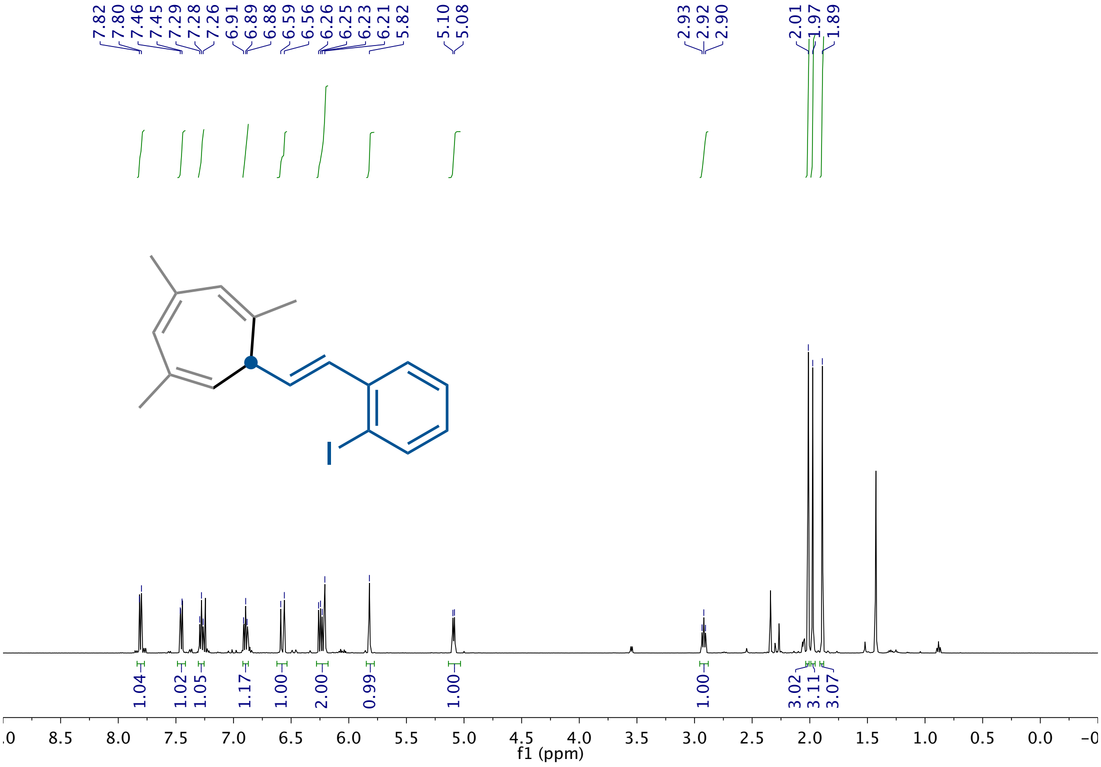

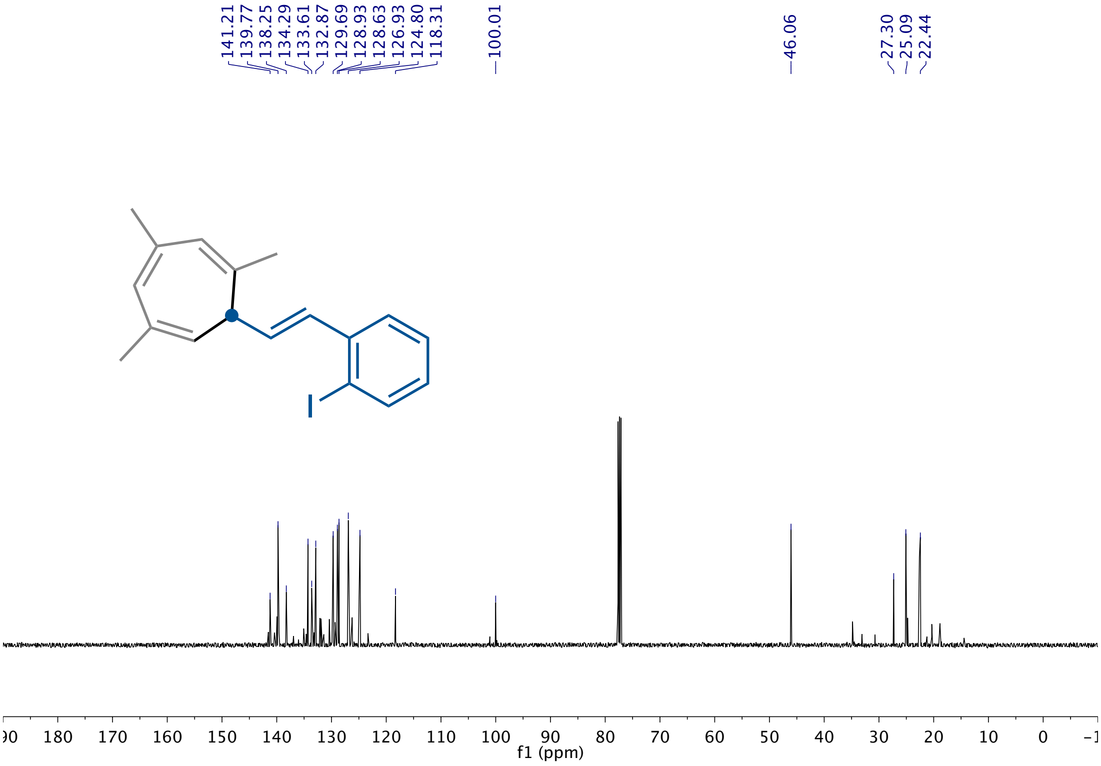

(±)-(1*S*,4*R*,5*R*)-4-Phenylbicyclo[3.2.2]nona-2,6-diene (3a)

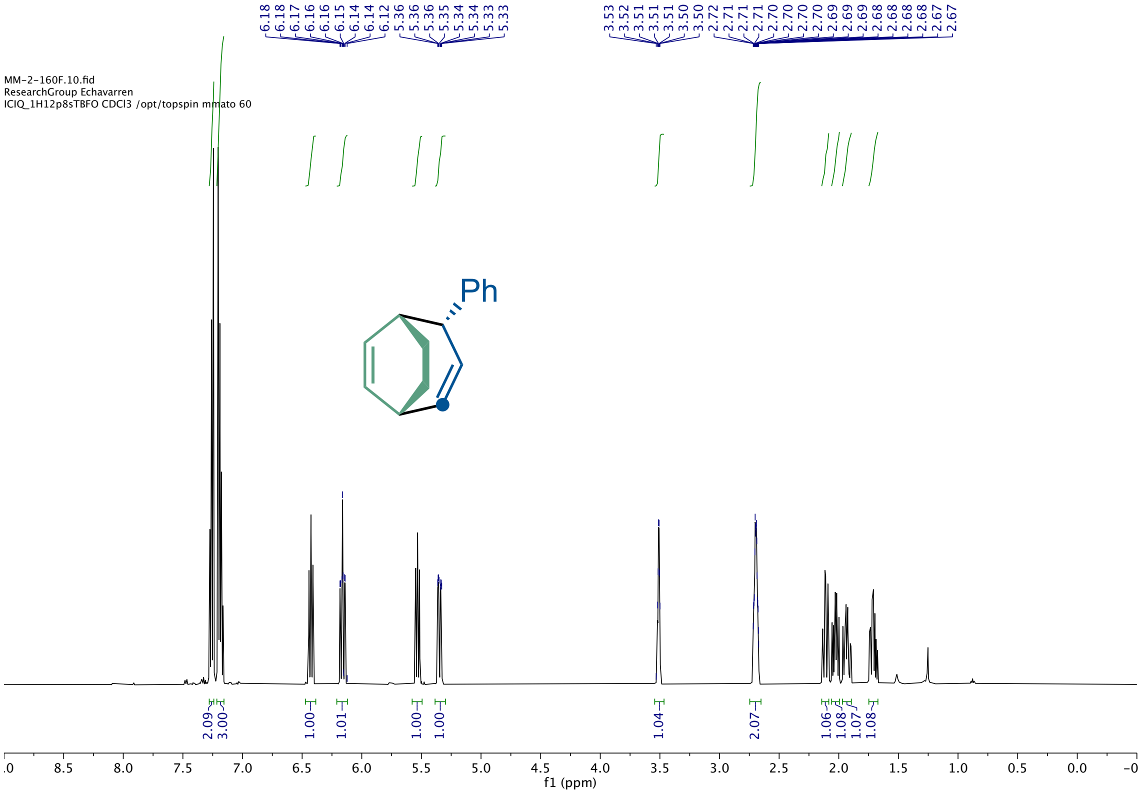

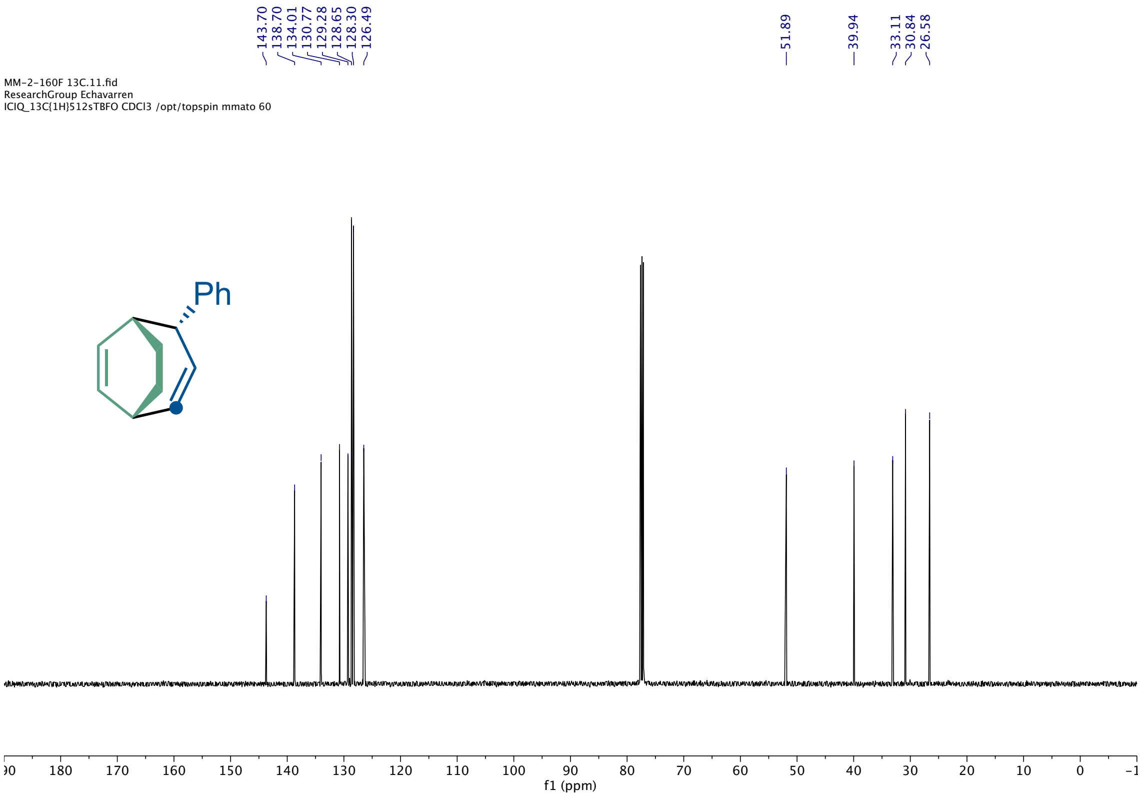

COSY of (3a) Detail

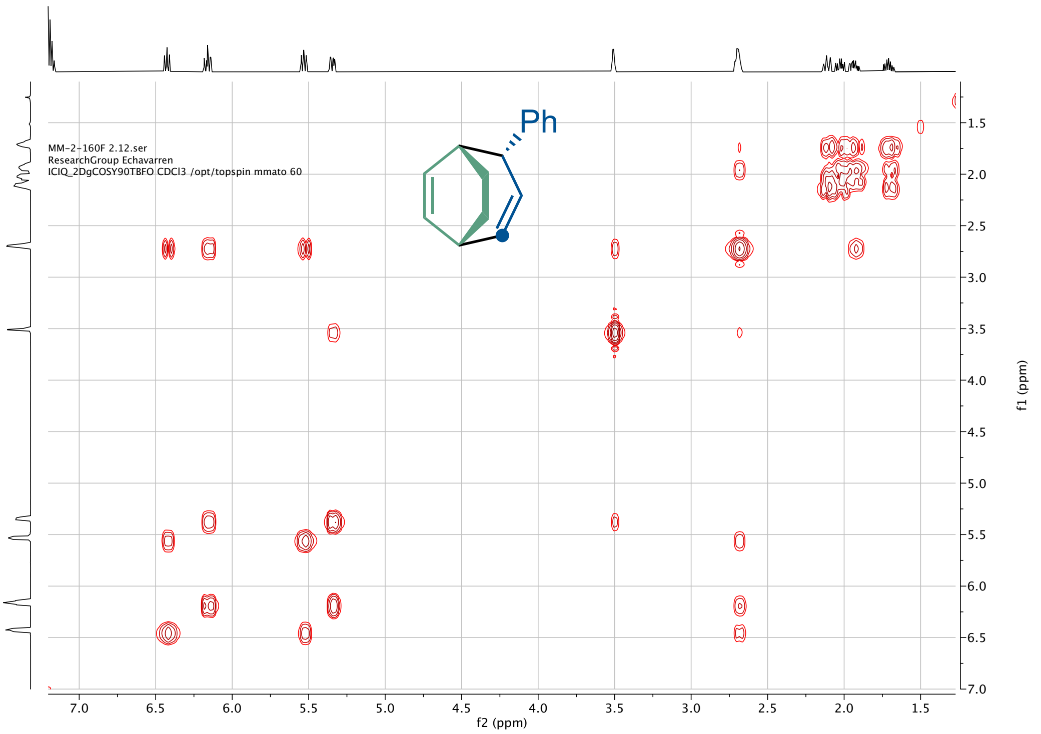

HMBC of (3a)

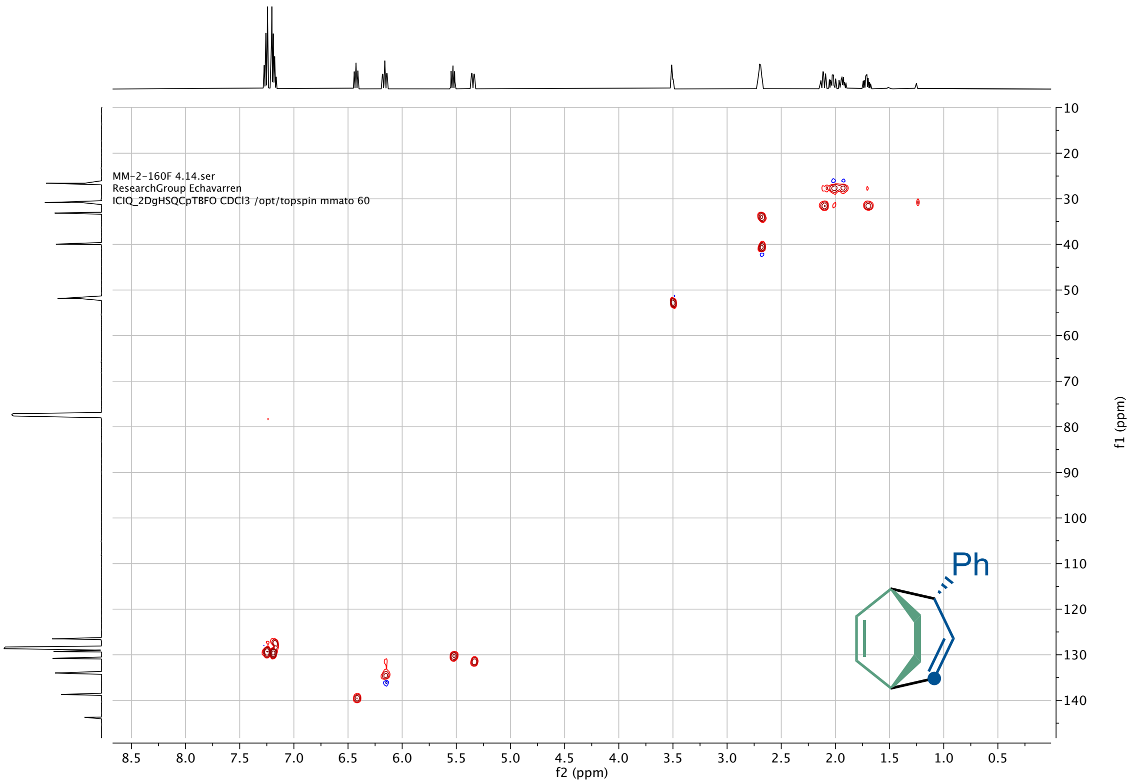

(±)-(4-((1*R*,2*R*,5*S*)-Bicyclo[3.2.2]nona-3,6-dien-2-yl)phenyl ferrocenoylate (3b)

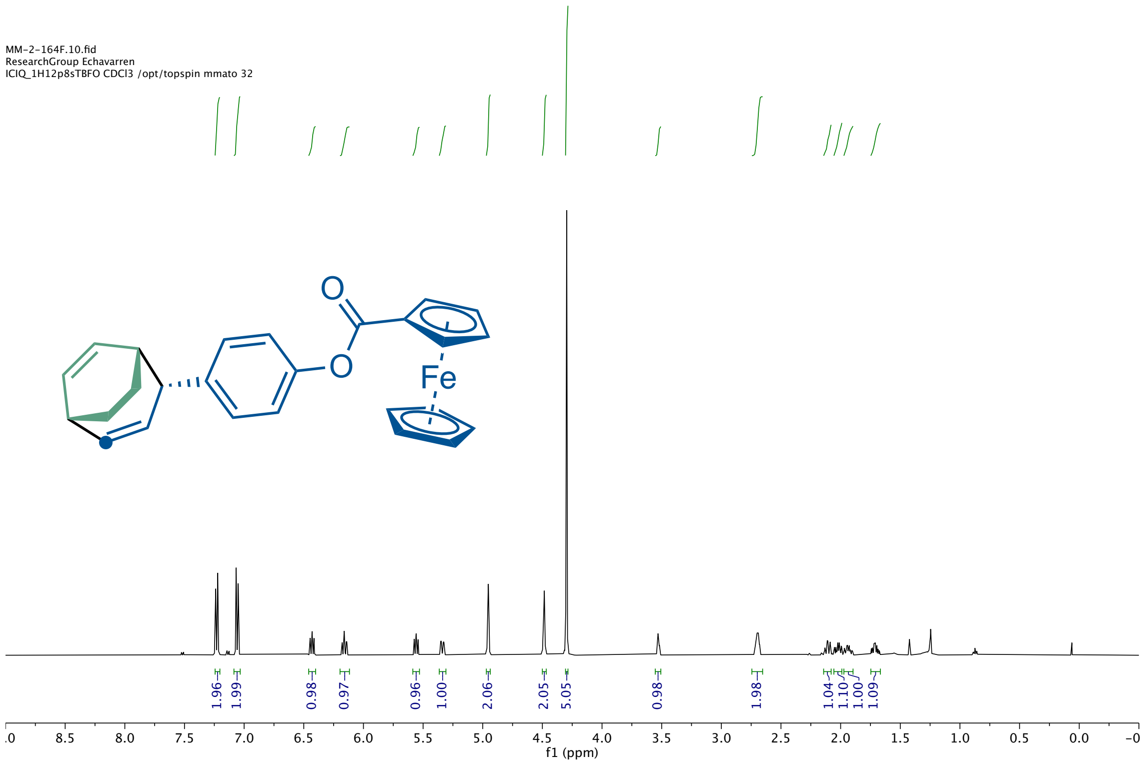

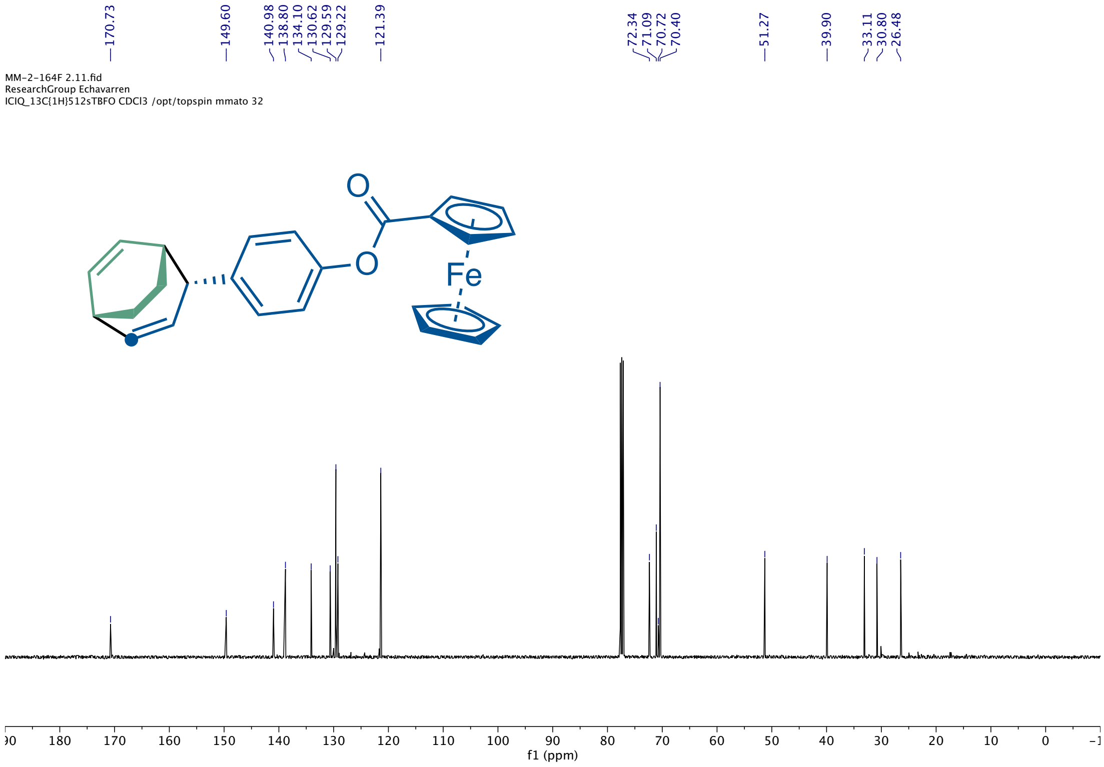

(±)-(1*S*,4*R*,5*R*)-4-(3,5-Bis(trifluoromethyl)phenyl)bicyclo[3.2.2]nona-2,6-diene (3c)

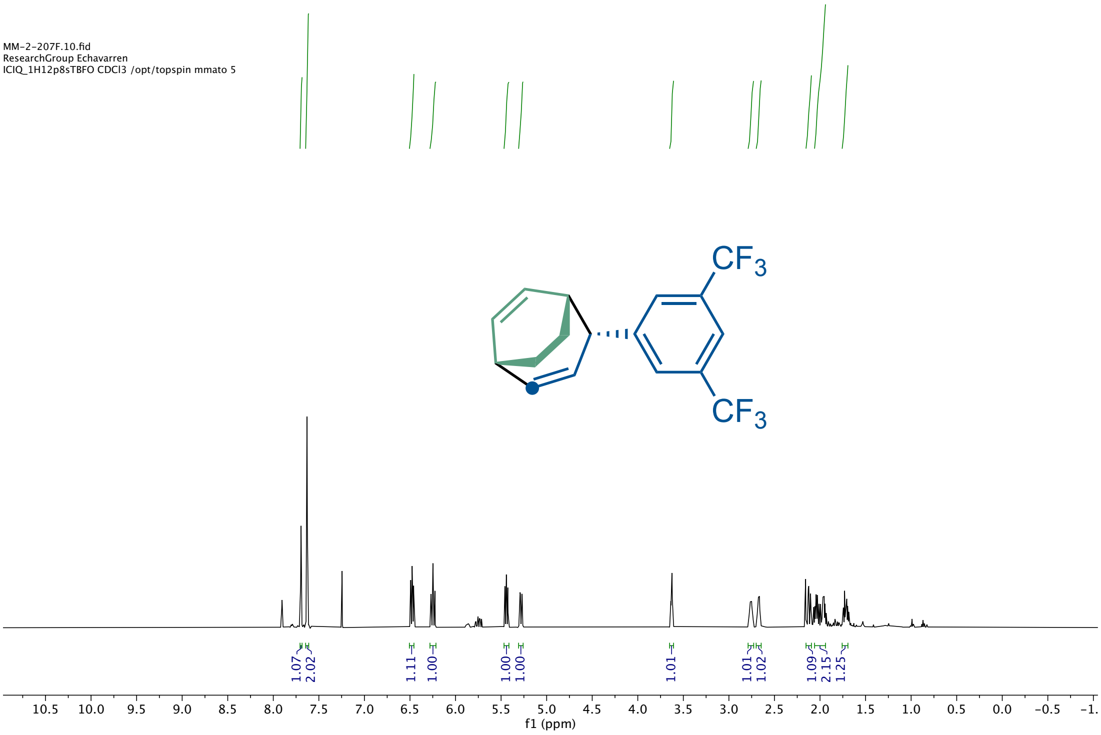

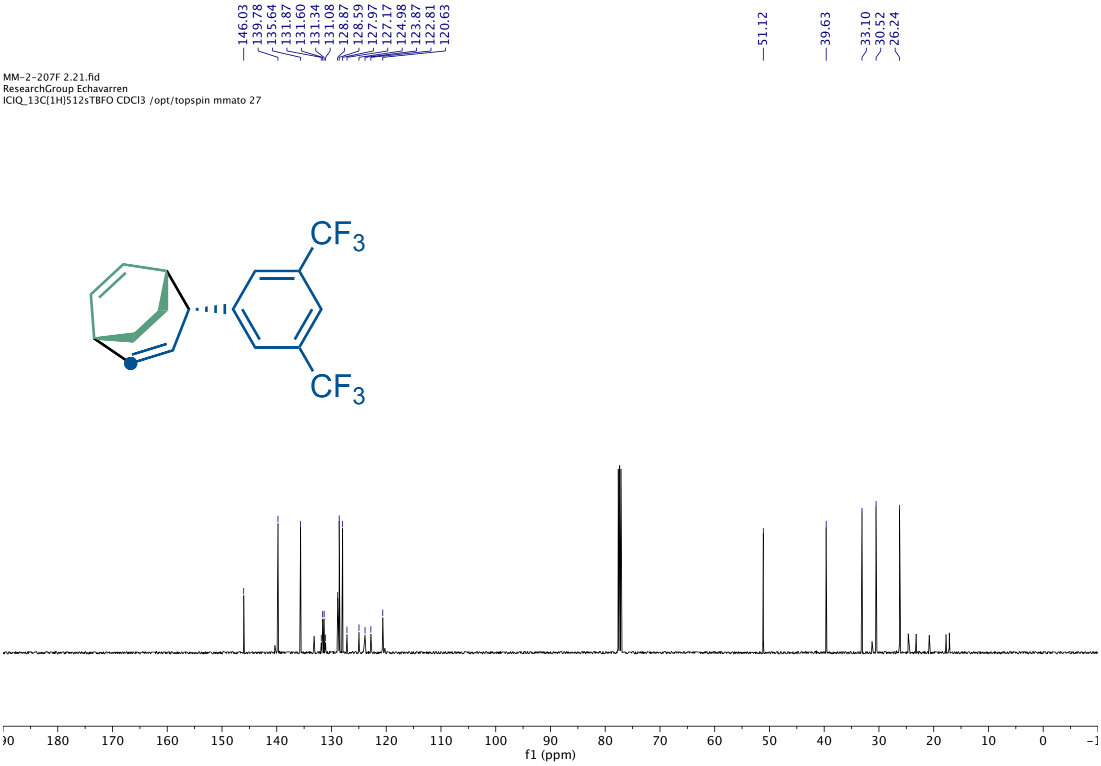

MM-2-207F 2.11.fid
ResearchGroup Echavarren
ICIQ_19F{1H}TBFO CDCl3 /opt/topspin mmato 5

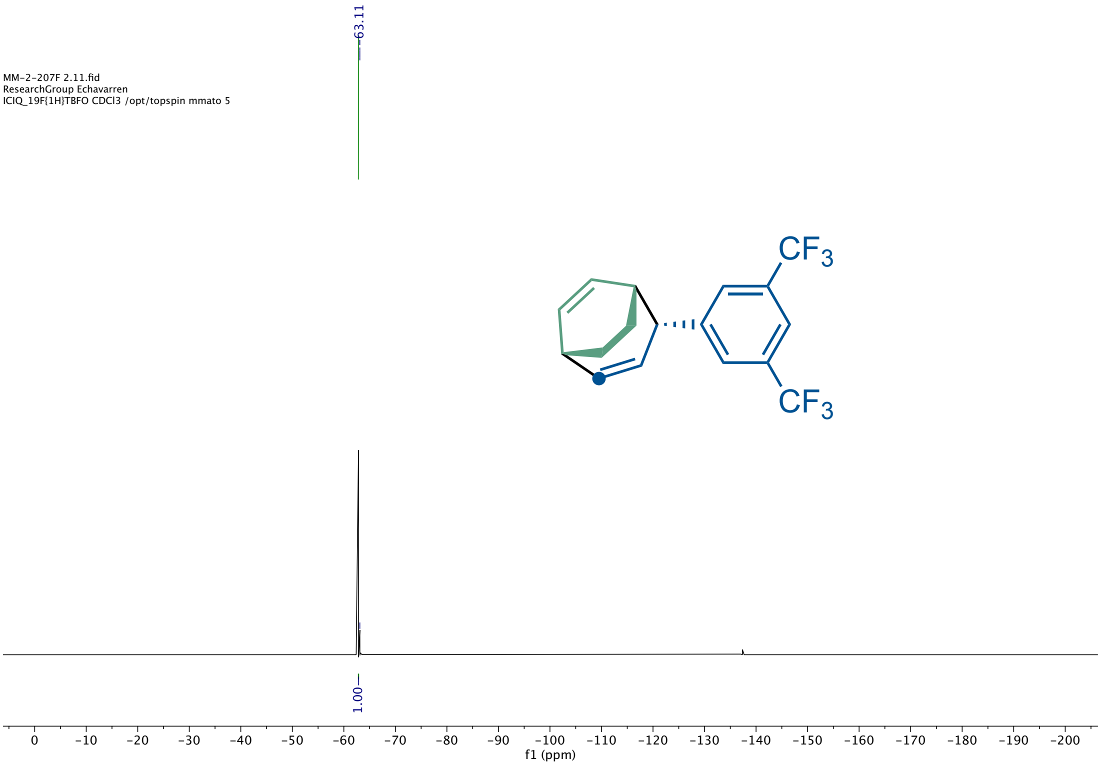

(±)-(1*S*,4*R*,5*R*)-4-(4-Methoxyphenyl)bicyclo[3.2.2]nona-2,6-diene (3d)

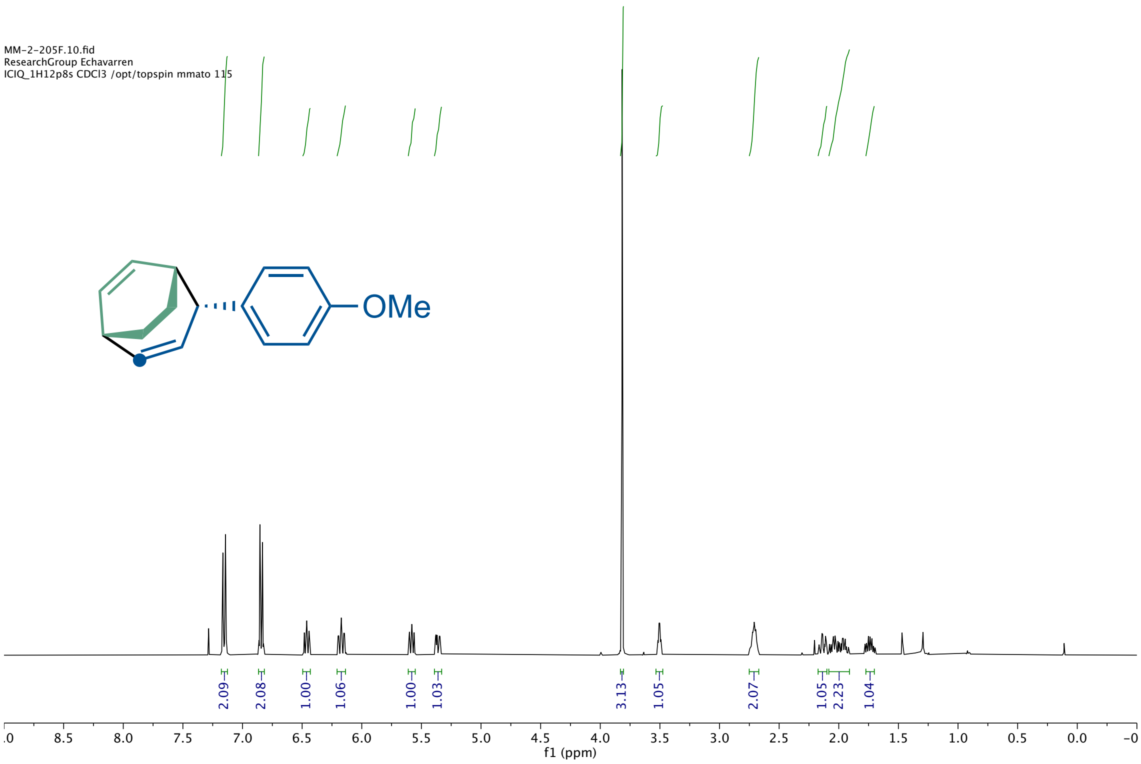

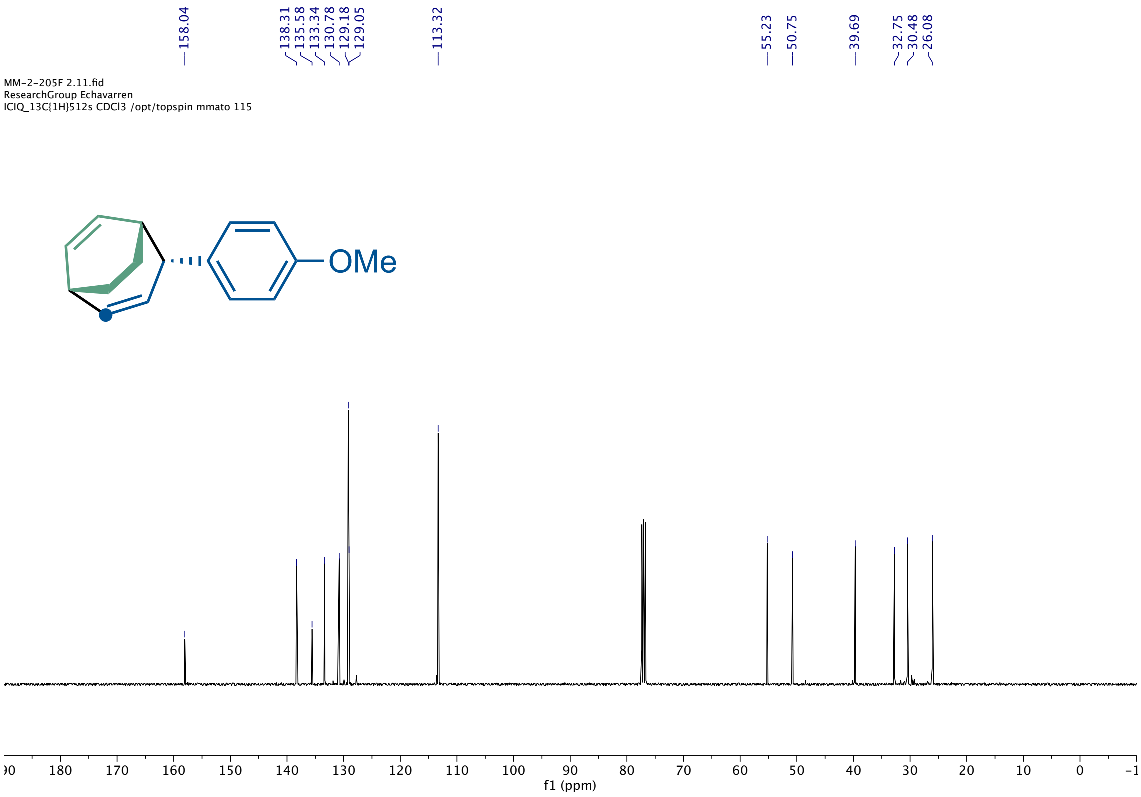

(±)-(1*S*,4*R*,5*R*)-4-(3,5-Dichlorophenyl)bicyclo[3.2.2]nona-2,6-diene (3e)

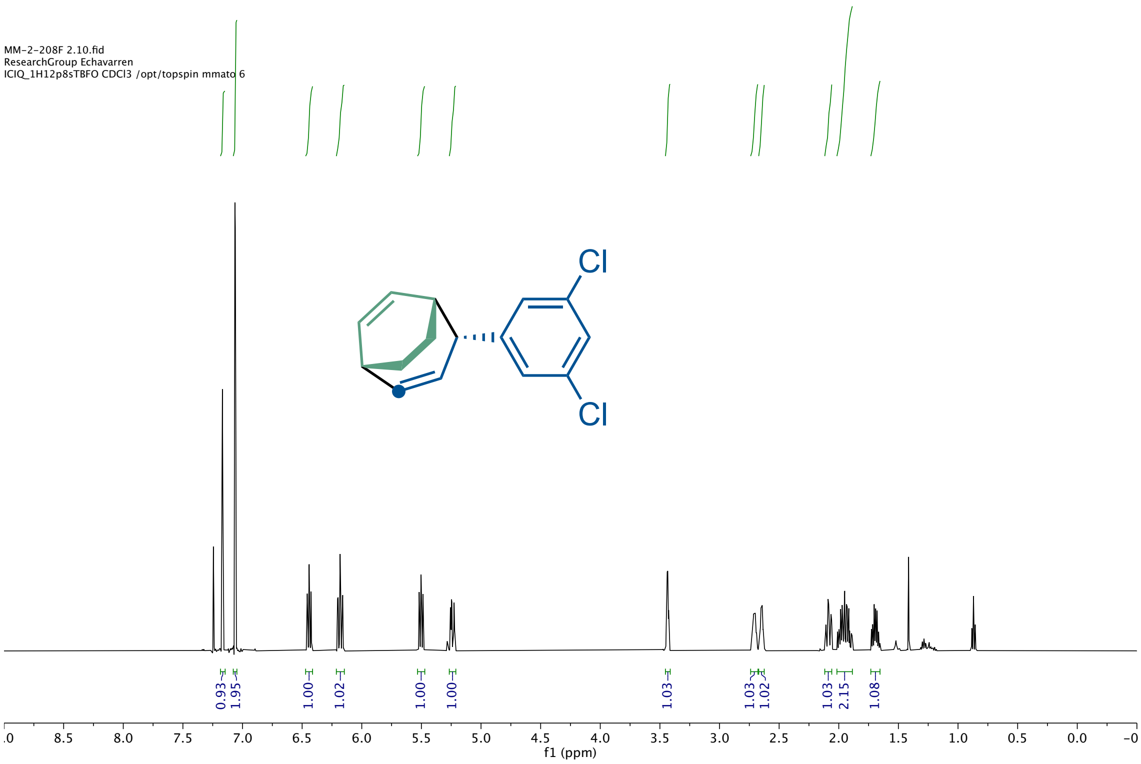

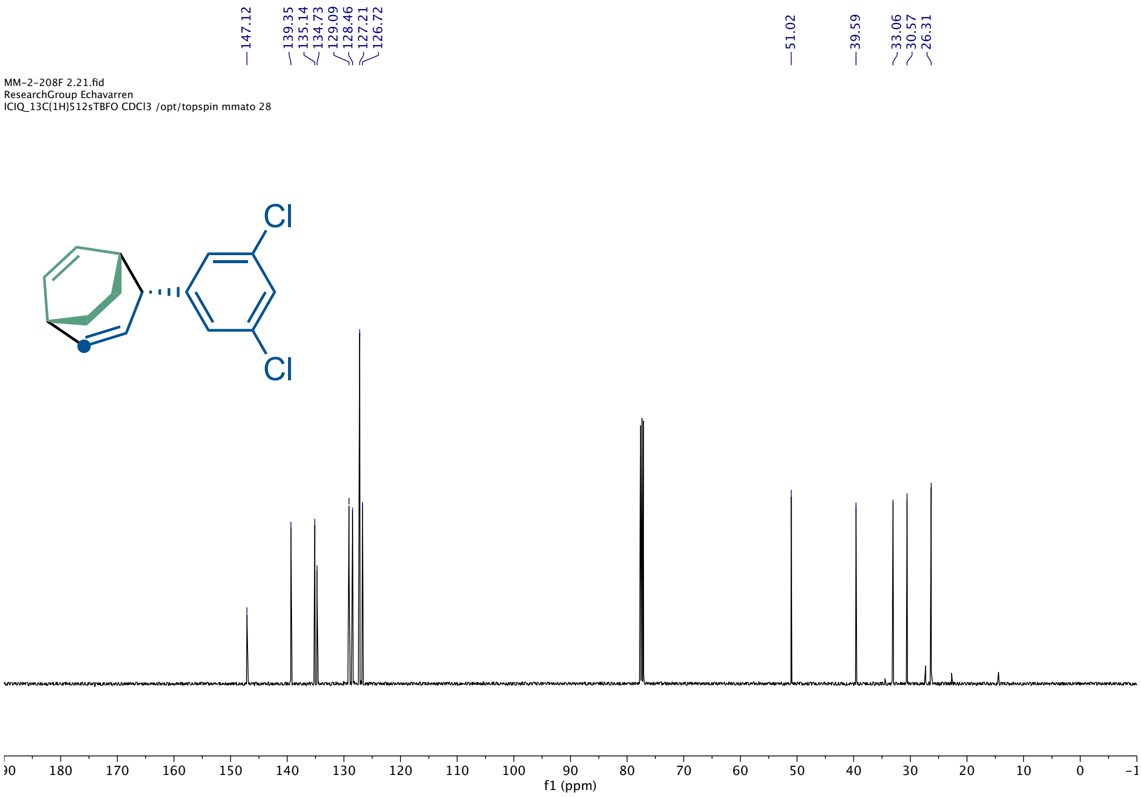

(±)-(1*S*,4*R*,5*R*)-4-(1-Iodophenyl)bicyclo[3.2.2]nona-2,6-diene (3f)

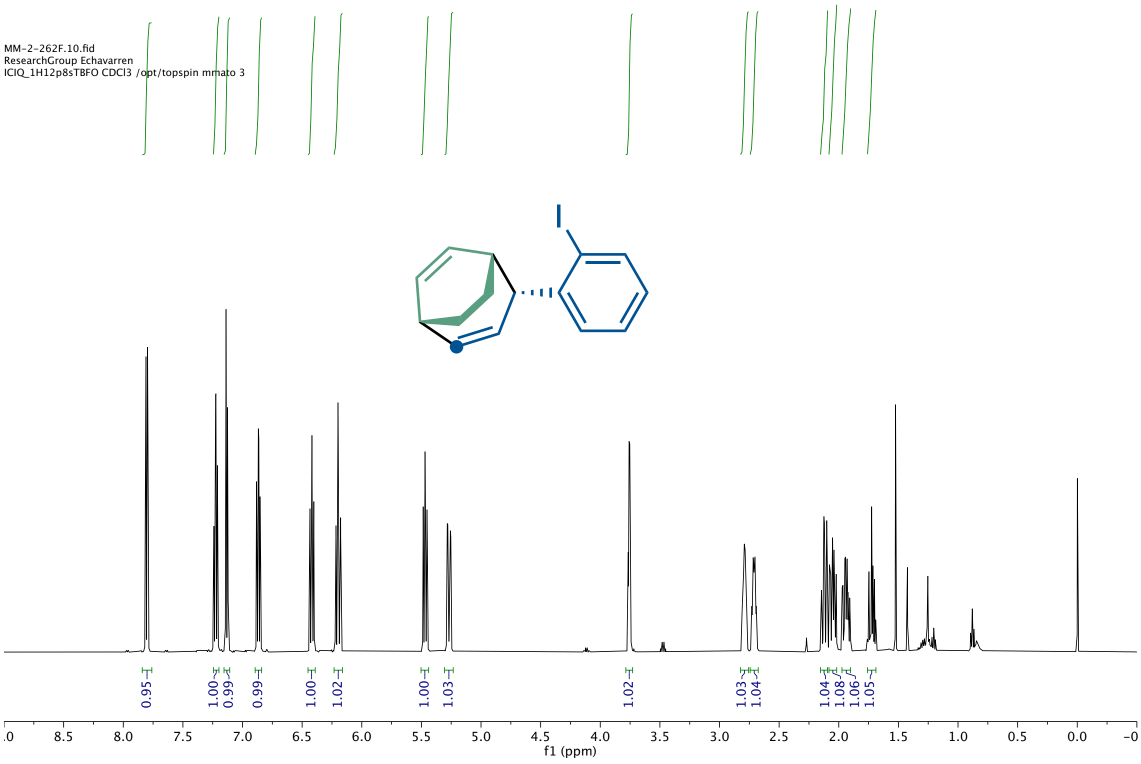

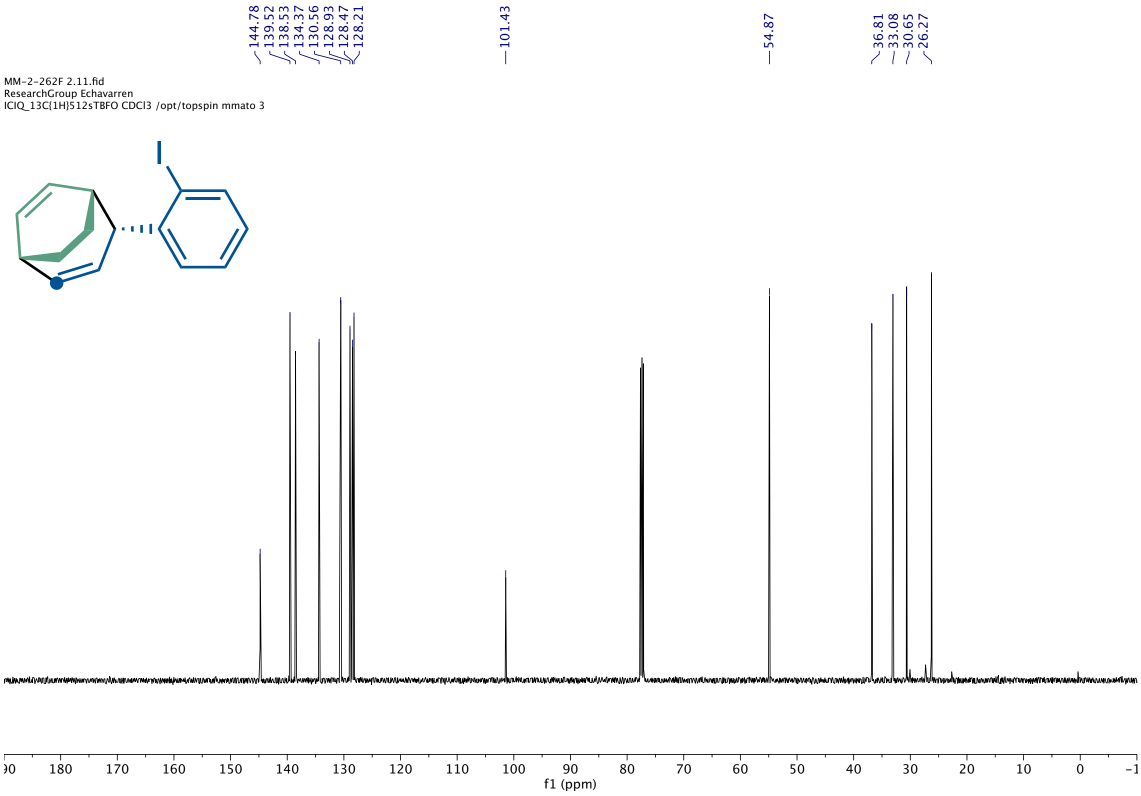

(±)-5-((1*R*,2*R*,5*S*)-Bicyclo[3.2.2]nona-3,6-dien-2-yl)-6-bromobenzo[*d*][1,3]dioxole (3g)

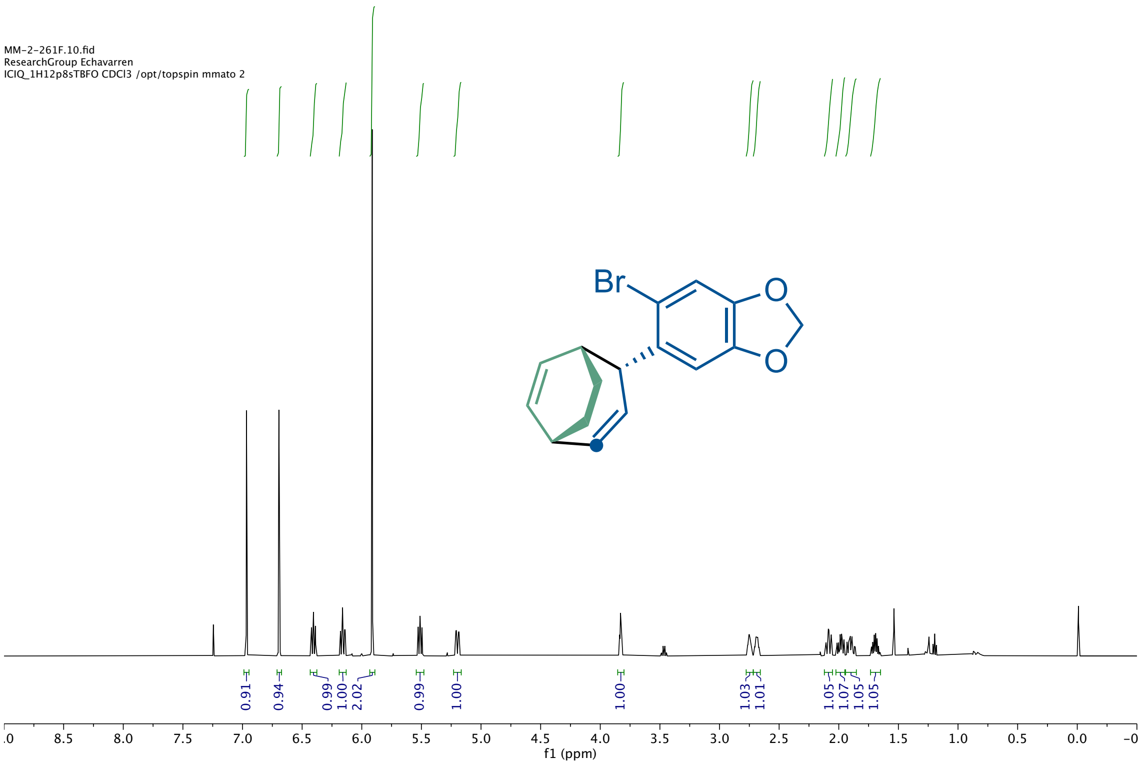

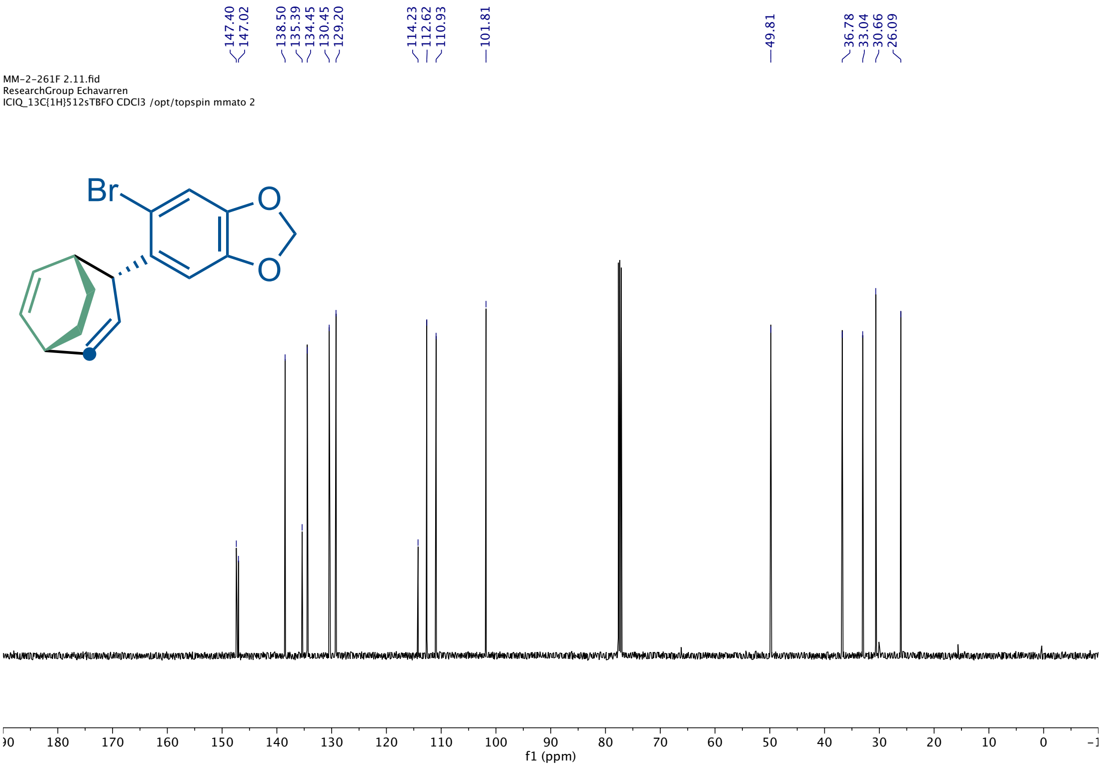

(±)-(1*S*,4*S*,5*R*)-4-((*E*)-4-Methoxystyryl)bicyclo[3.2.2]nona-2,6-diene (3h)

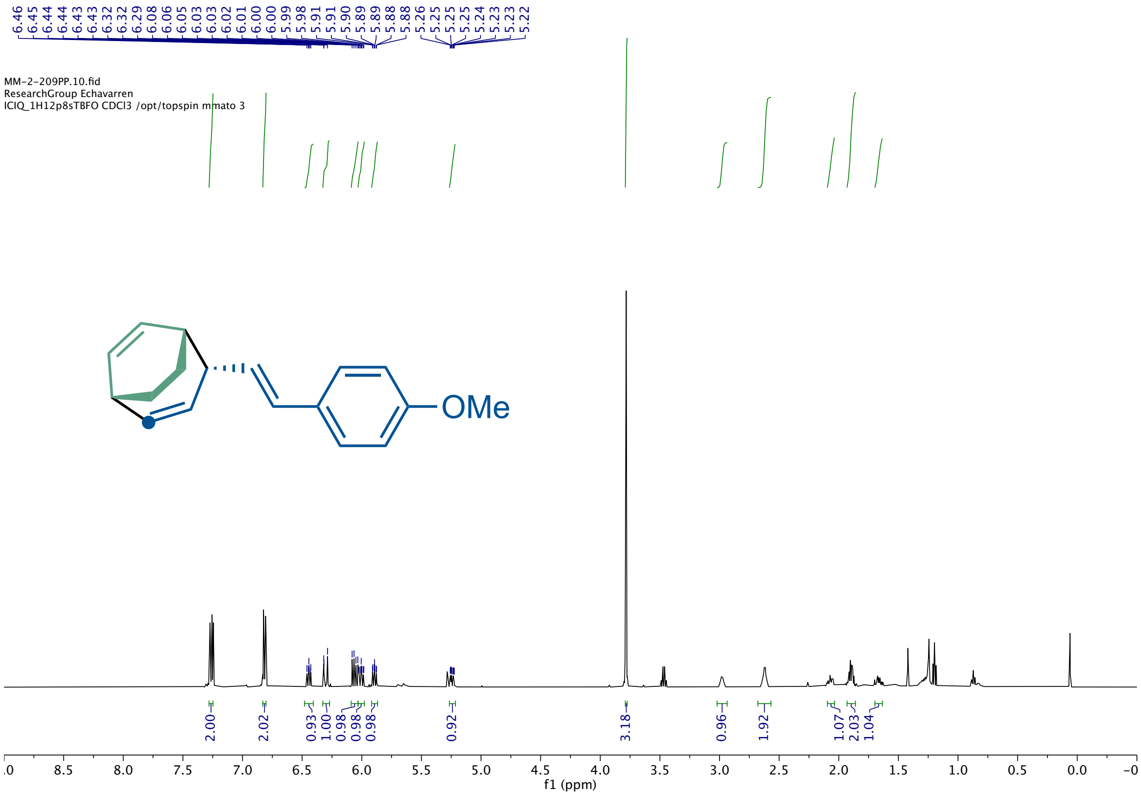

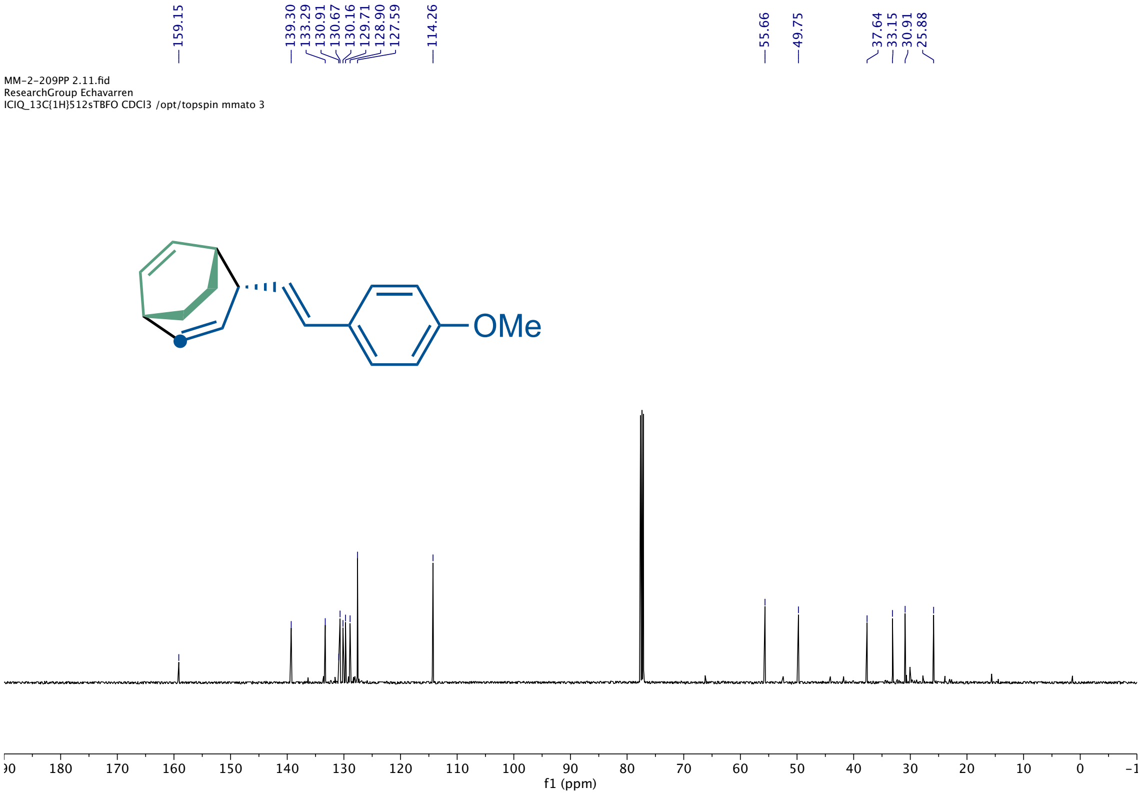

cis-Trimethyl((4-phenylcyclohepta-2,6-dien-1-yl)oxy)silane (3i)

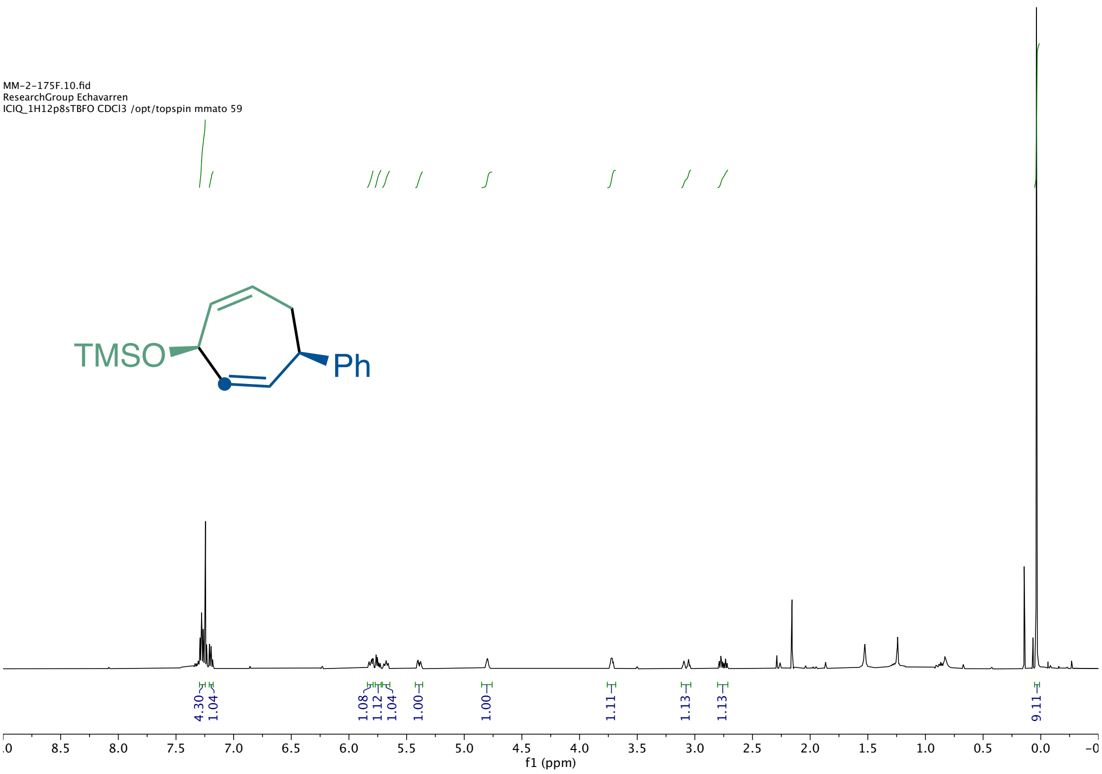

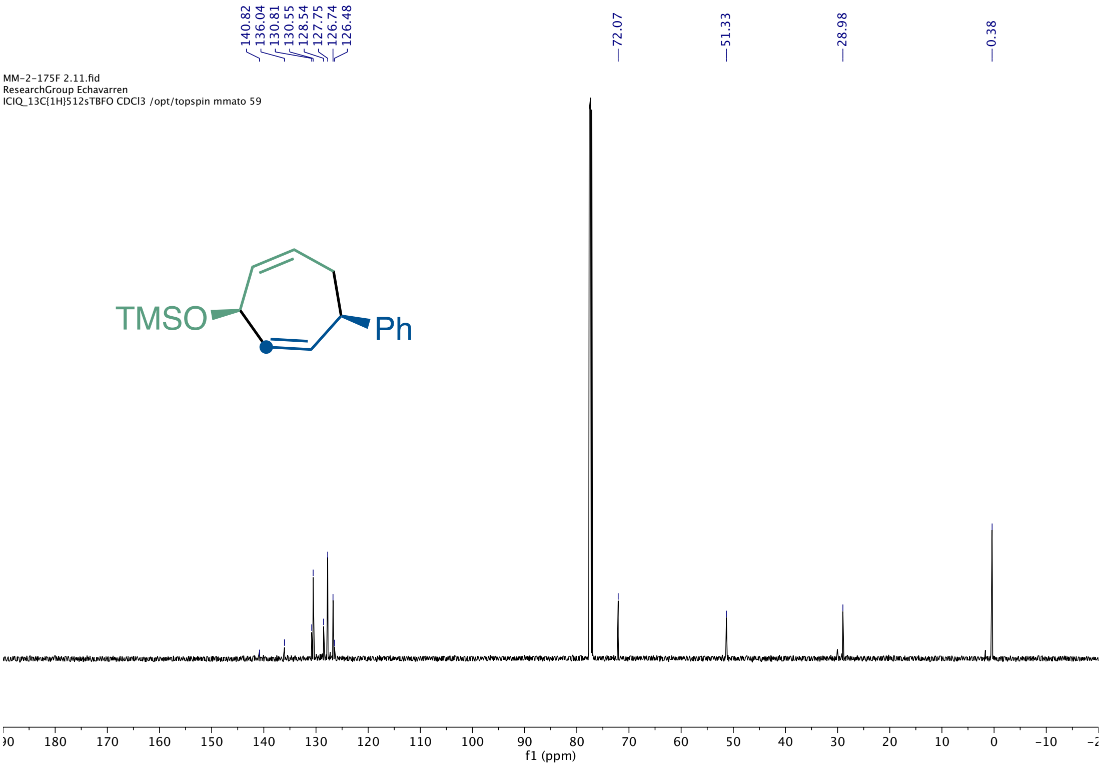

cis-3-Methoxy-6-phenylcyclohepta-1,4-diene (3j)

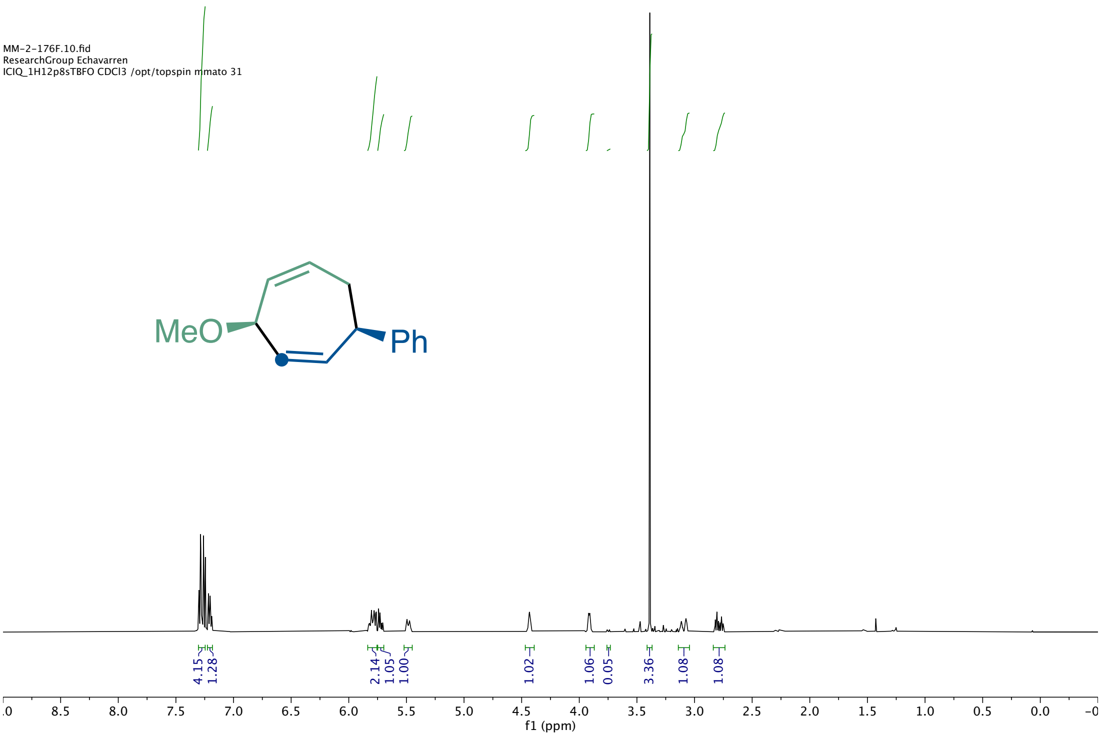

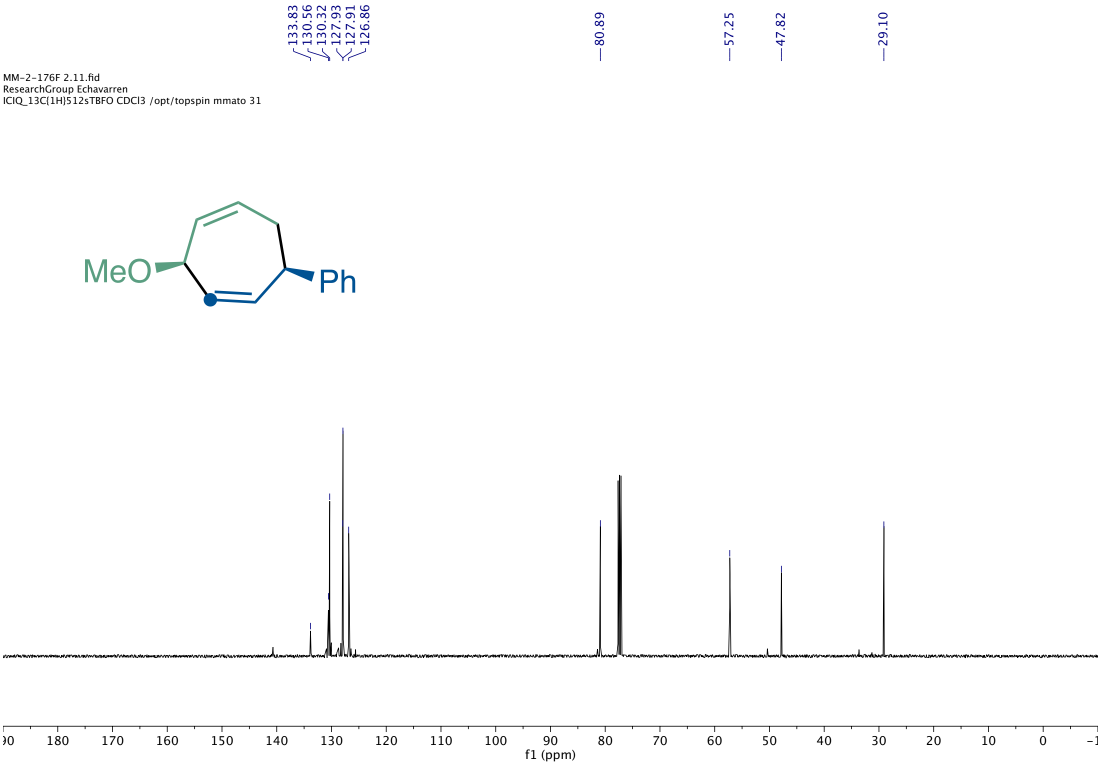

cis-4-(4-Methoxycyclohepta-2,5-dien-1-yl)phenyl ferrocenoylate (3k)

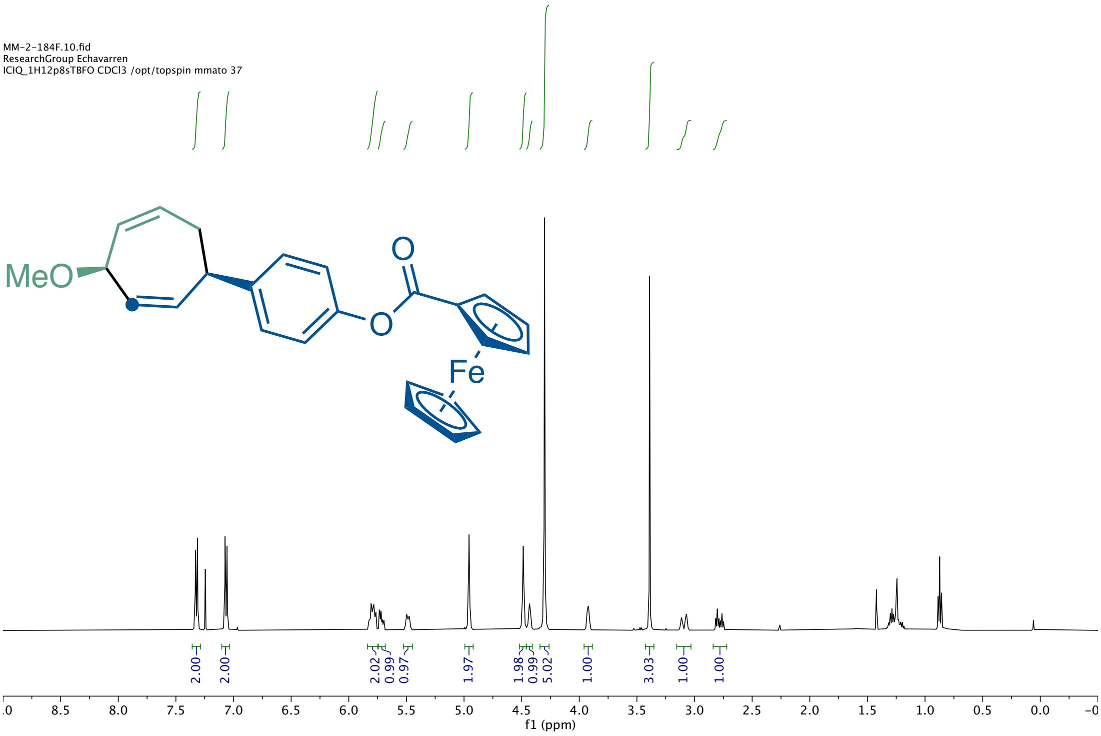

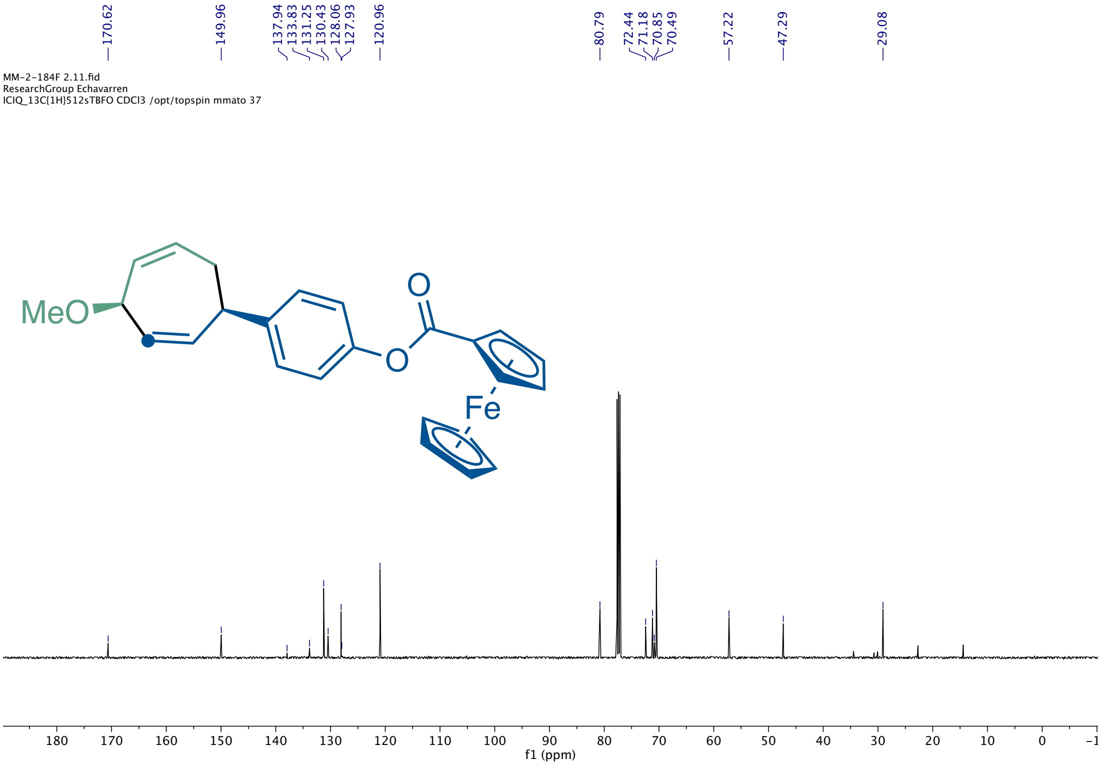

(±)-(1*S*,4*S*,5*S*)-1,5,6,7,8-Pentamethyl-4-phenylbicyclo[3.2.1]octa-2,6-diene (3l)

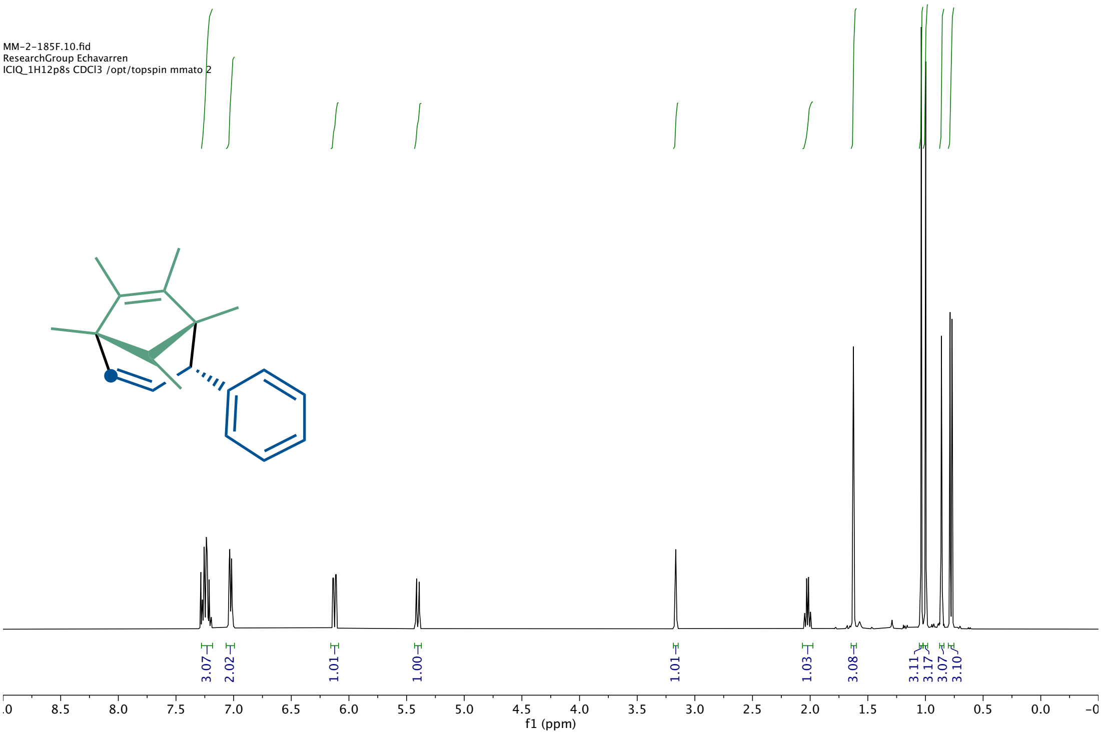

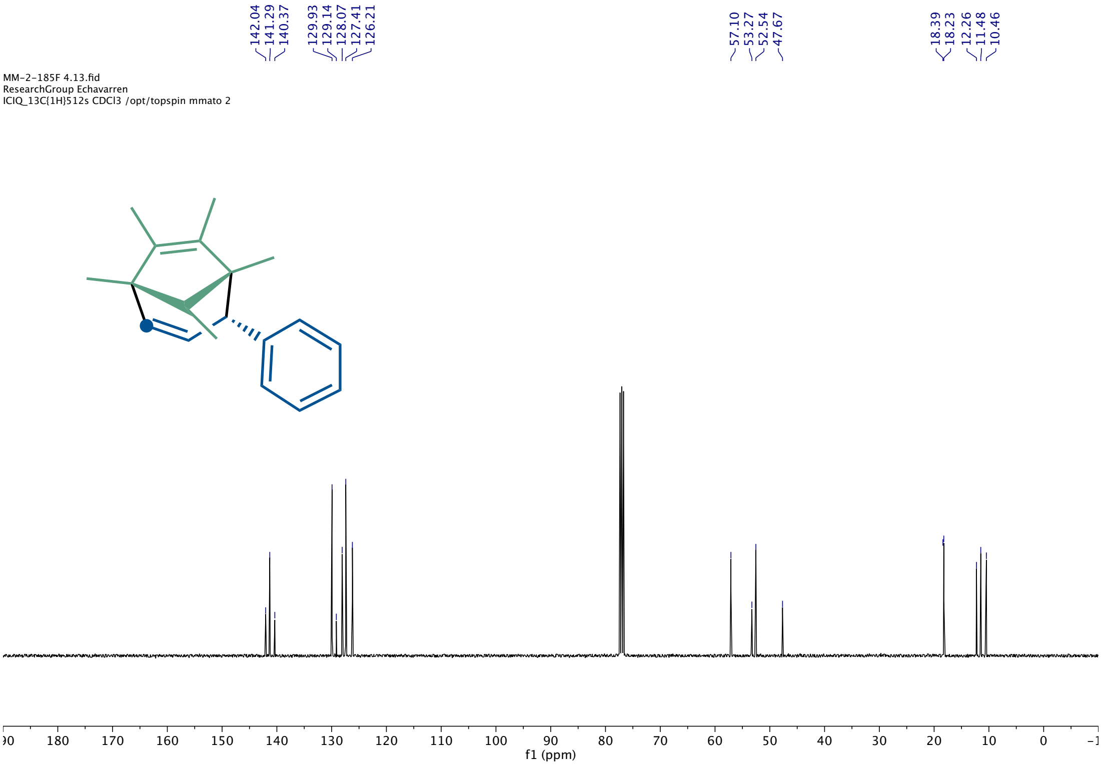

(±)-(1*S*,4*S*,5*S*)-1,5,6,7,8-Pentamethyl-4-(3-bromophenyl)bicyclo[3.2.1]octa-2,6-diene (3m)

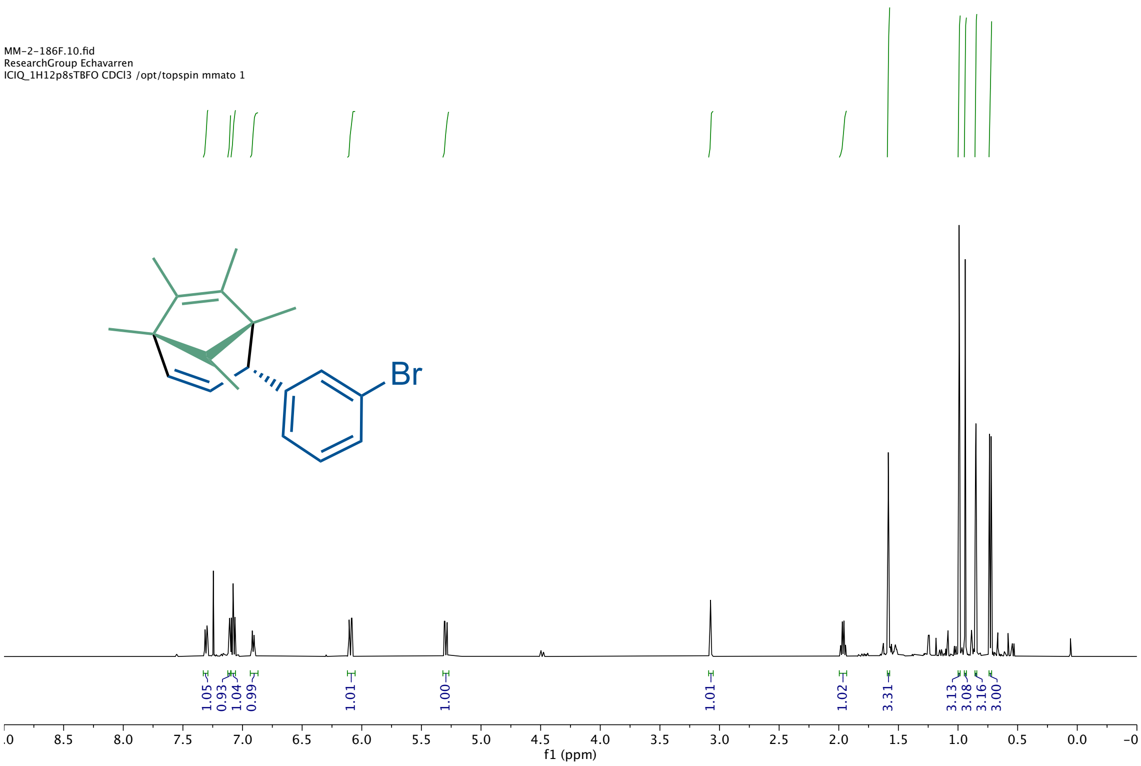

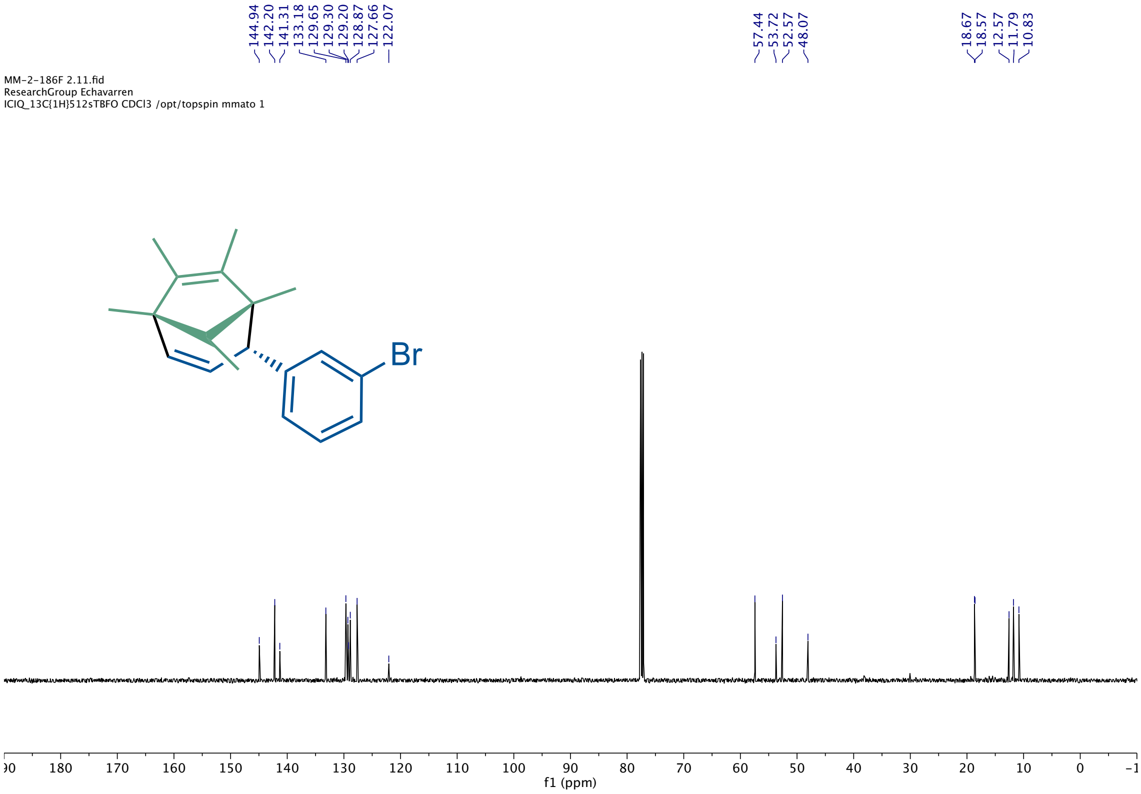

(±)-Trimethyl((2-((1*S*,2*S*,5*S*)-1,5,6,7,8-pentamethylbicyclo[3.2.1]octa-3,6-dien-2-yl)phenyl)ethynyl)silane (3n)

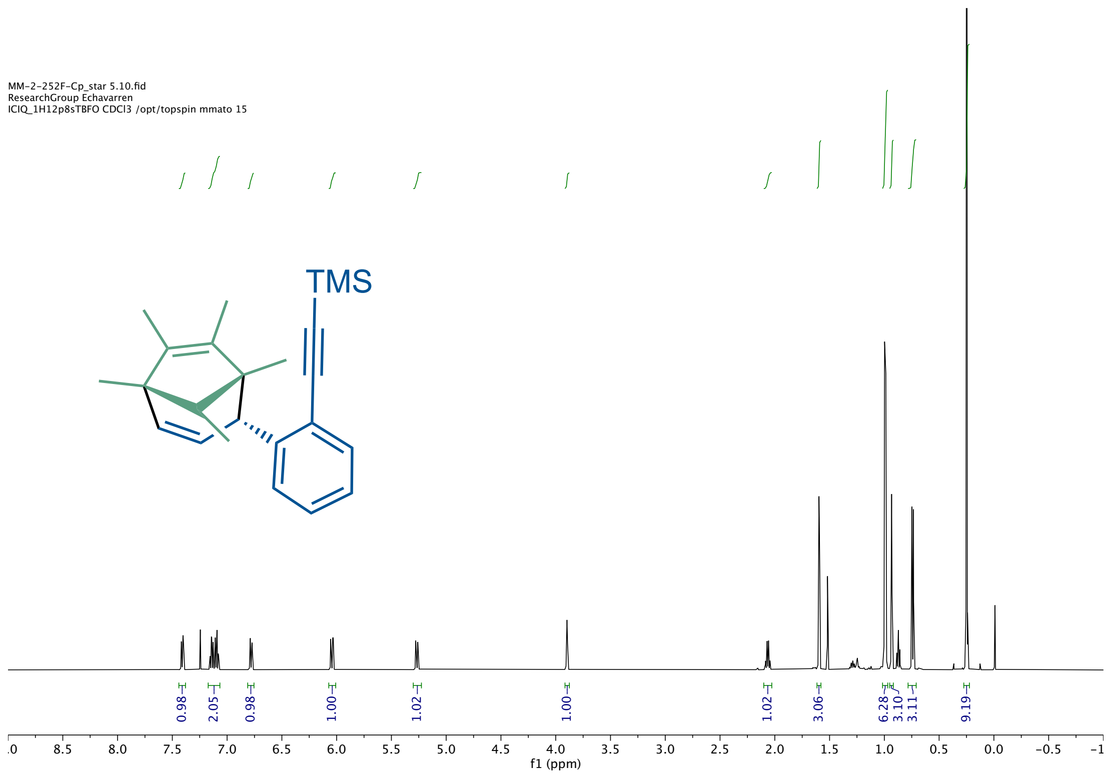

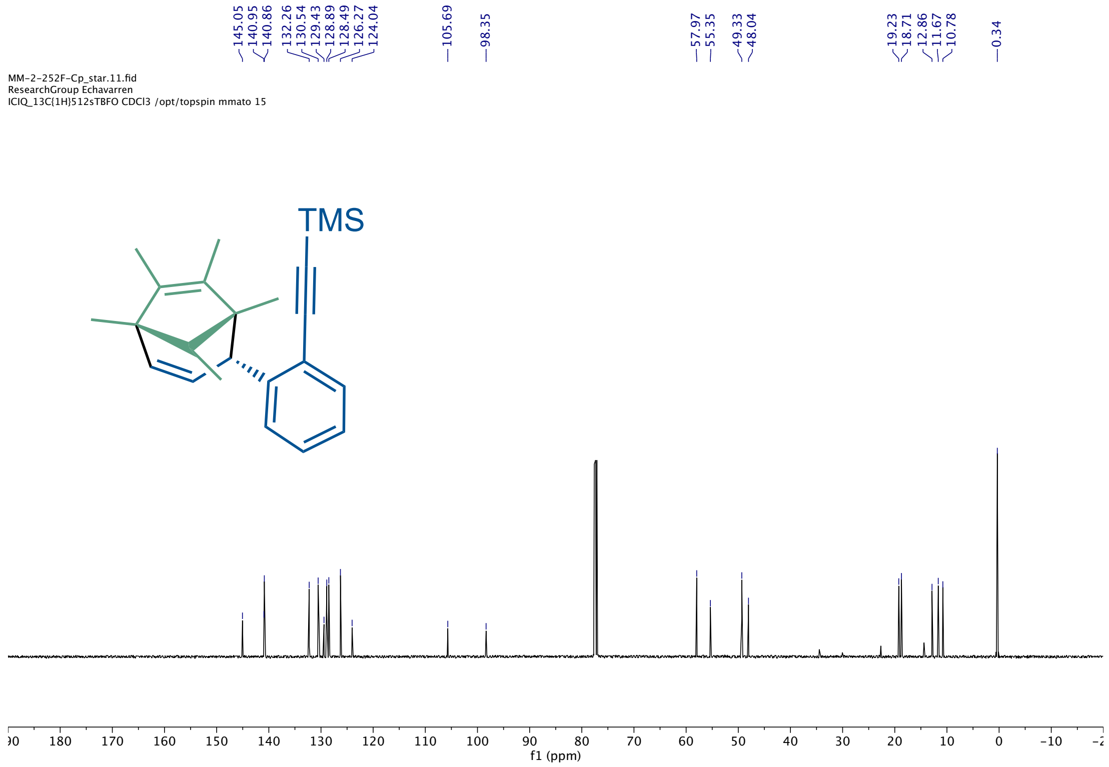

(±)-(1*S*,4*R*,5*R*)-5-Isopropyl-1-methyl-4-phenylbicyclo[3.2.2]nona-2,6-diene (3p)

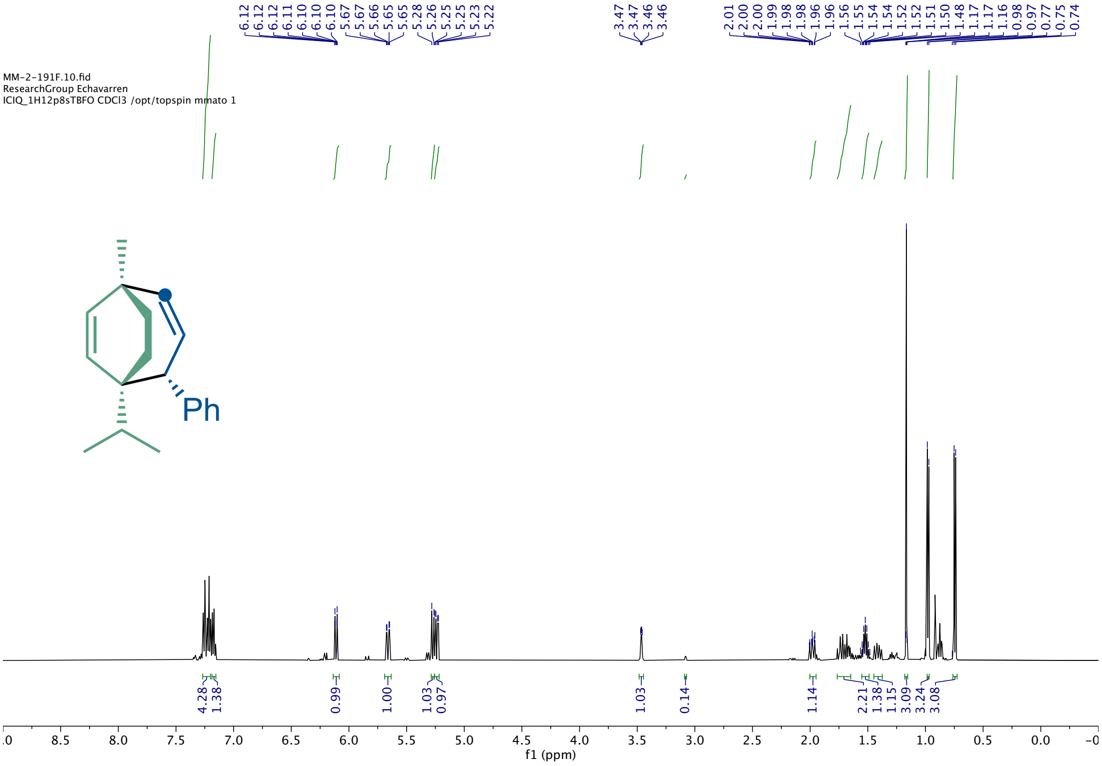

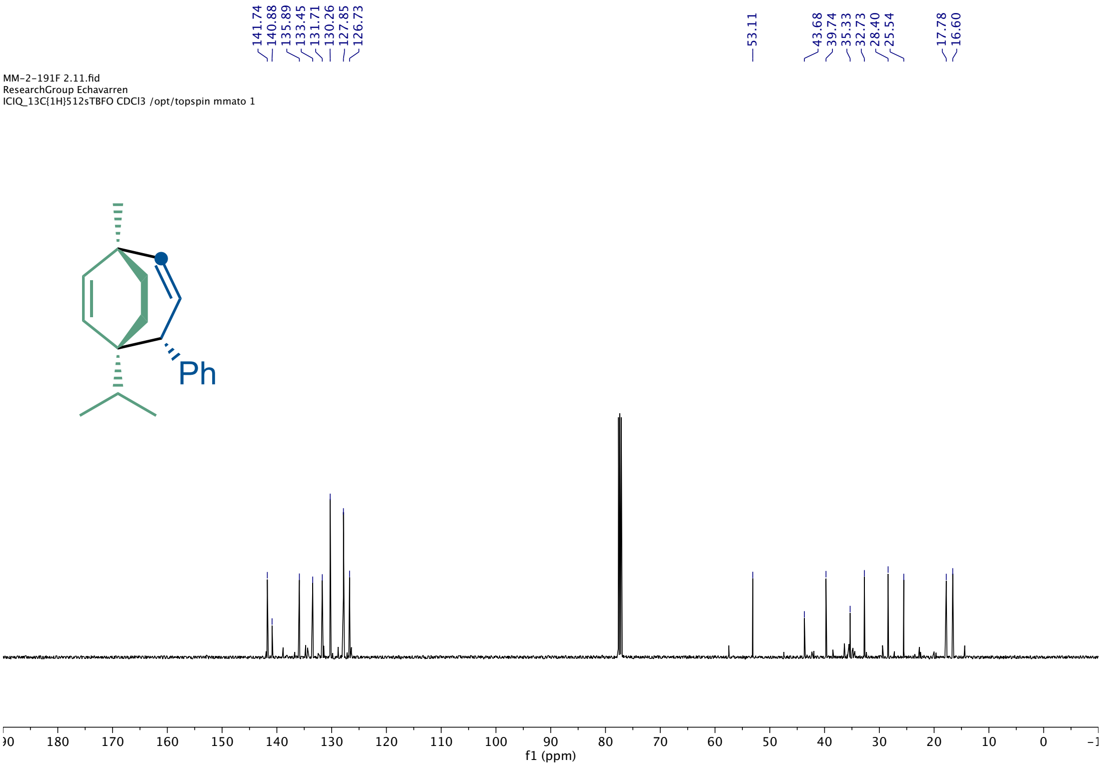

cis-6,7-Diphenylcyclohepta-1,4-diene (3q)

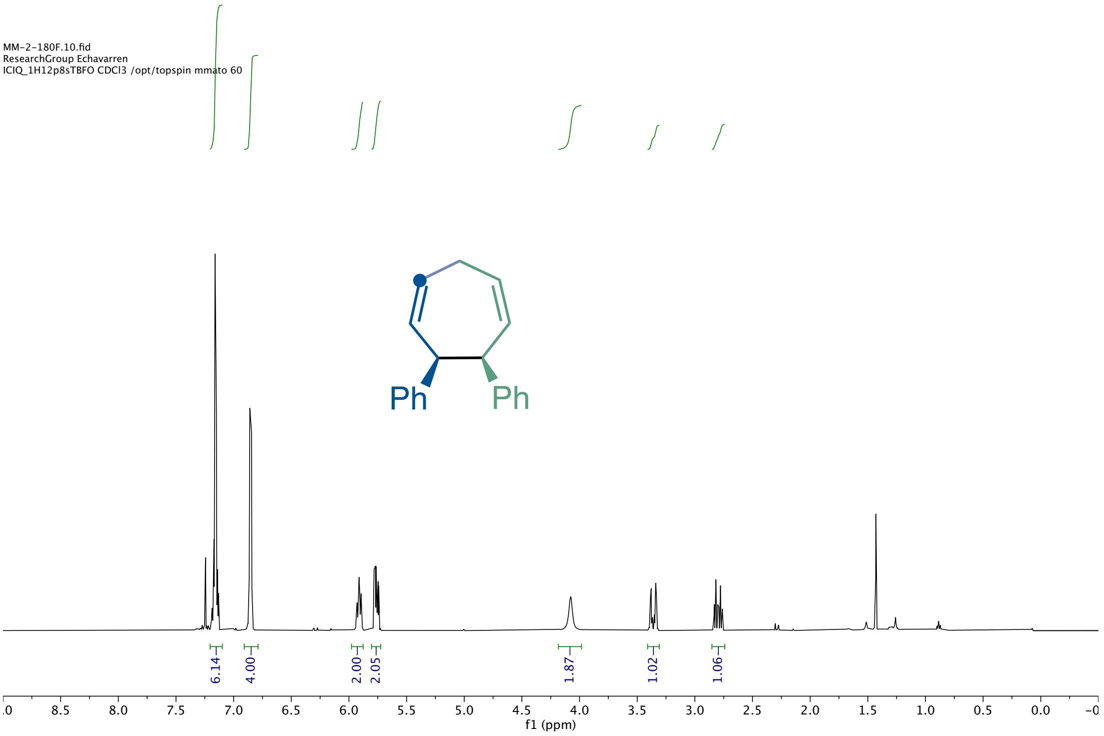

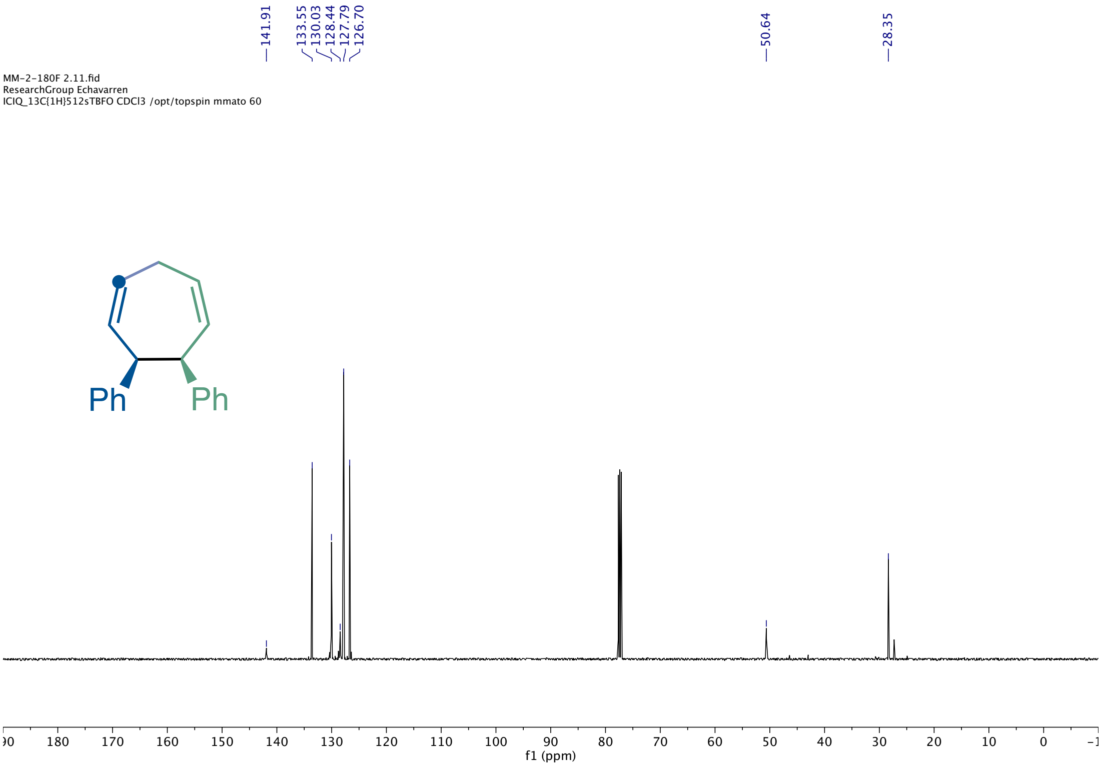

(±)-(1*S*,4*R*,5*R*)-4-Phenylbicyclo[3.2.1]octa-2,6-diene (3r)

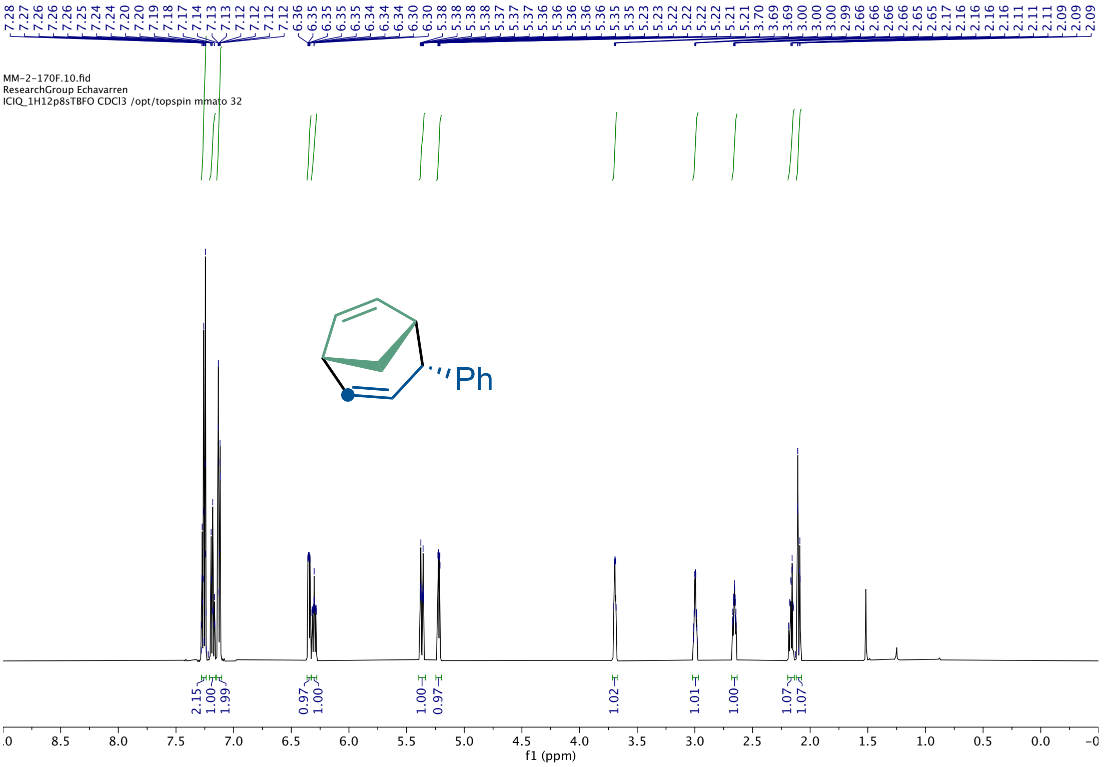

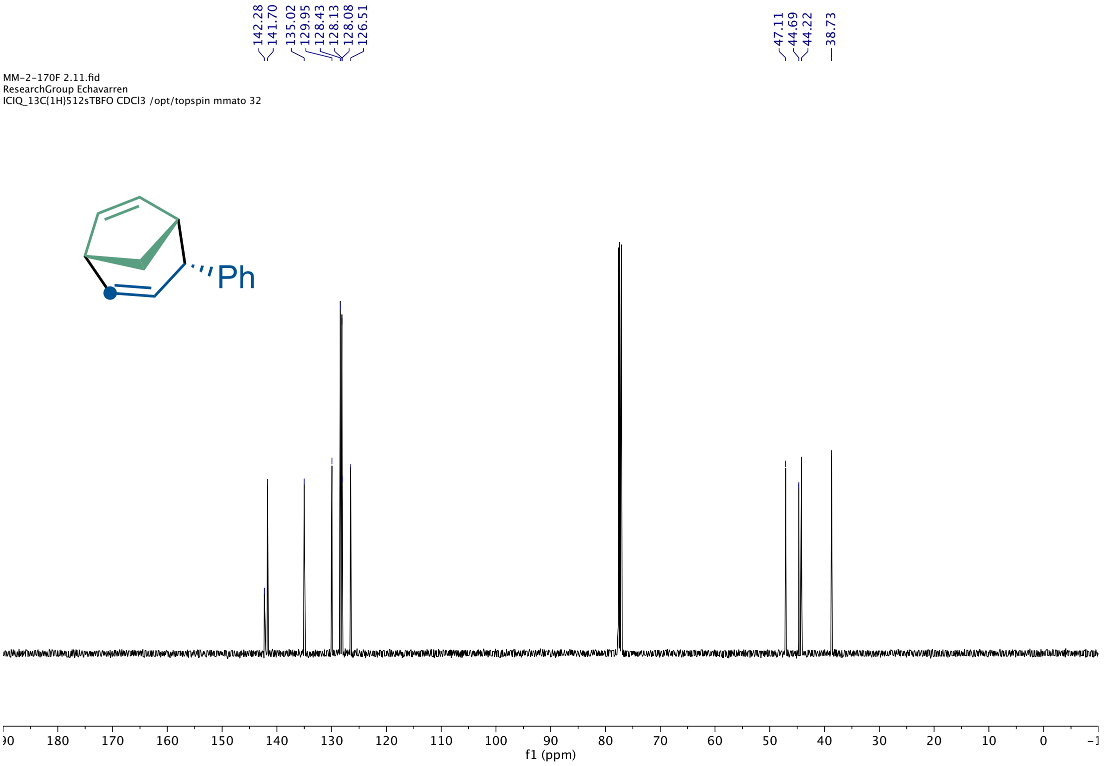

(±)-(1*R*,4*S*,5*S*)-1,5,6,7-Tetramethyl-4-phenylbicyclo[3.2.1]octa-2,6-diene (3s)

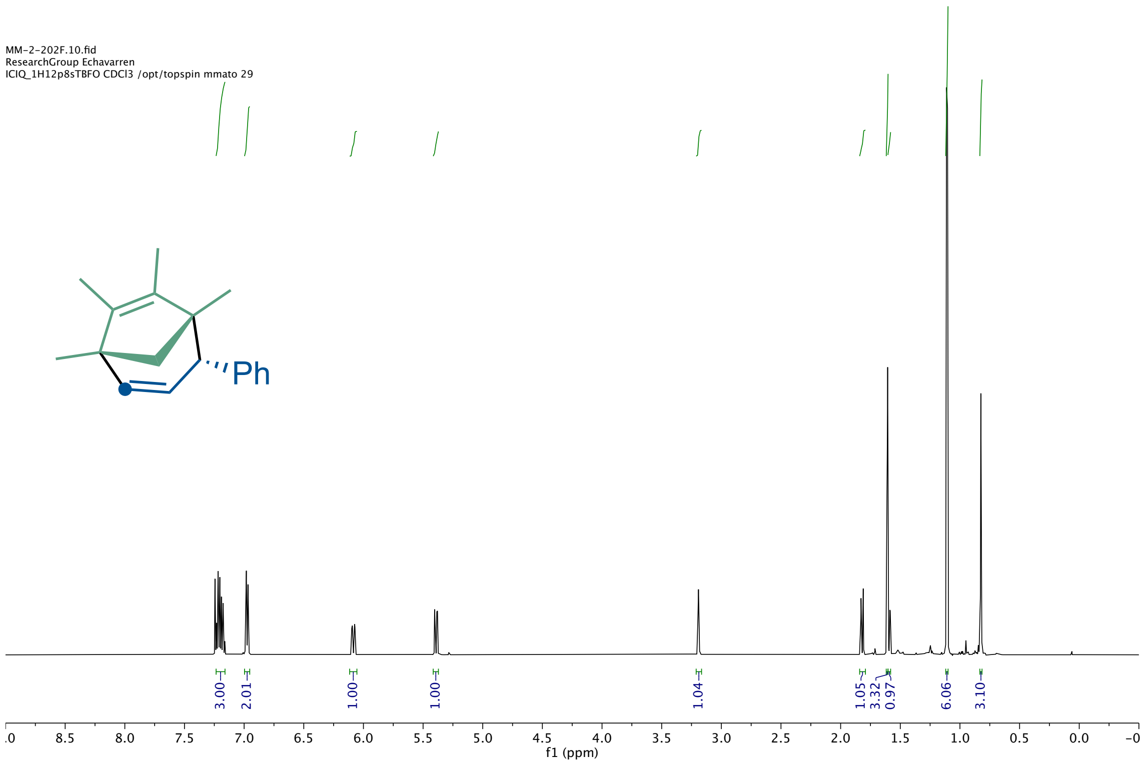

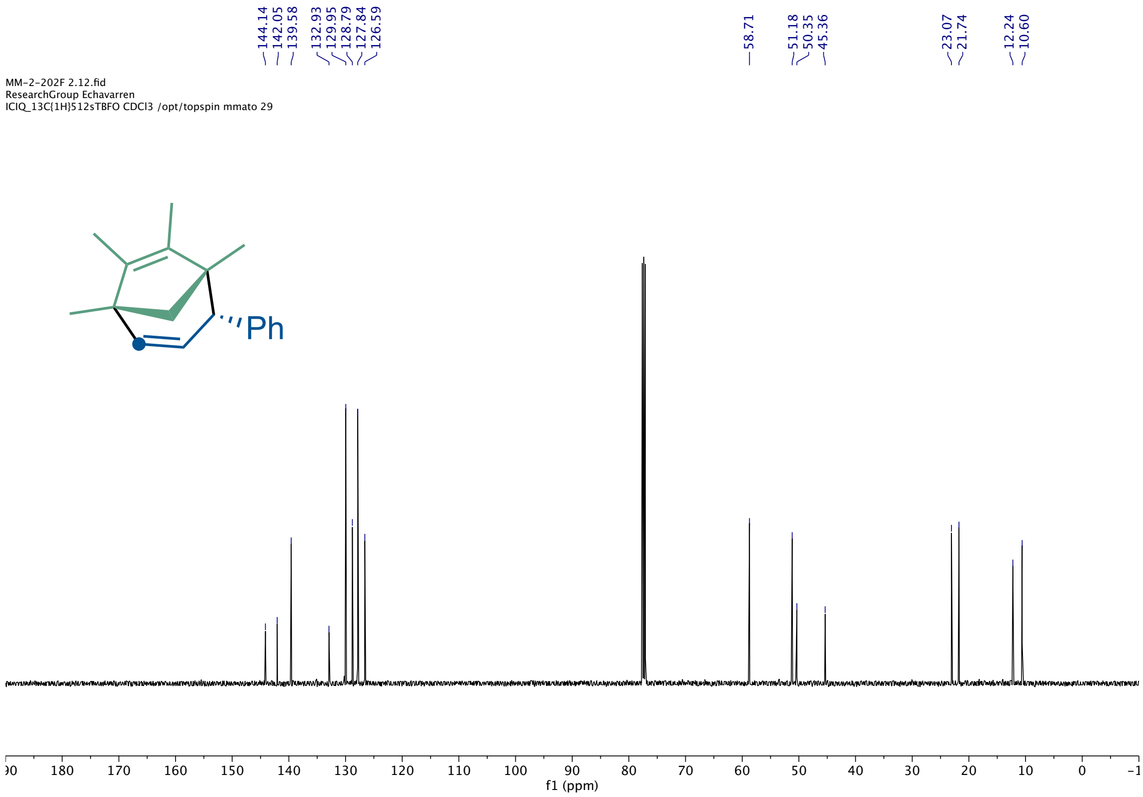

(±)-(1*S*,4*R*,5*R*)-4-Phenylbicyclo[3.3.2]deca-2,9-diene (3t)

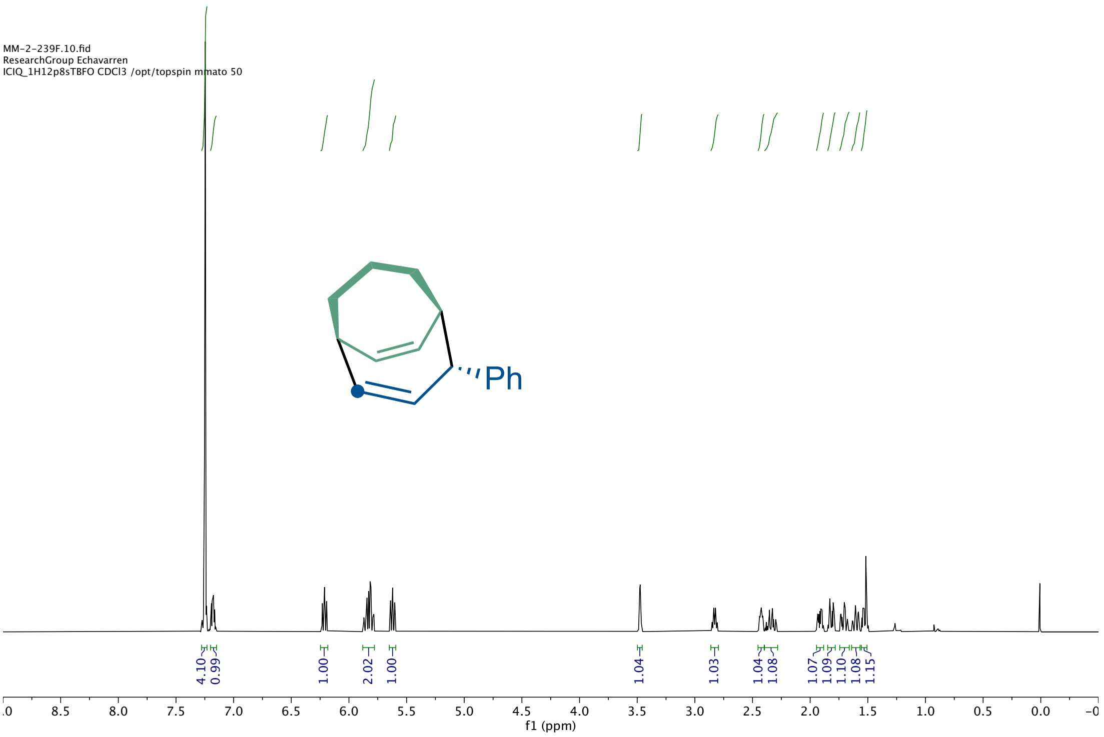

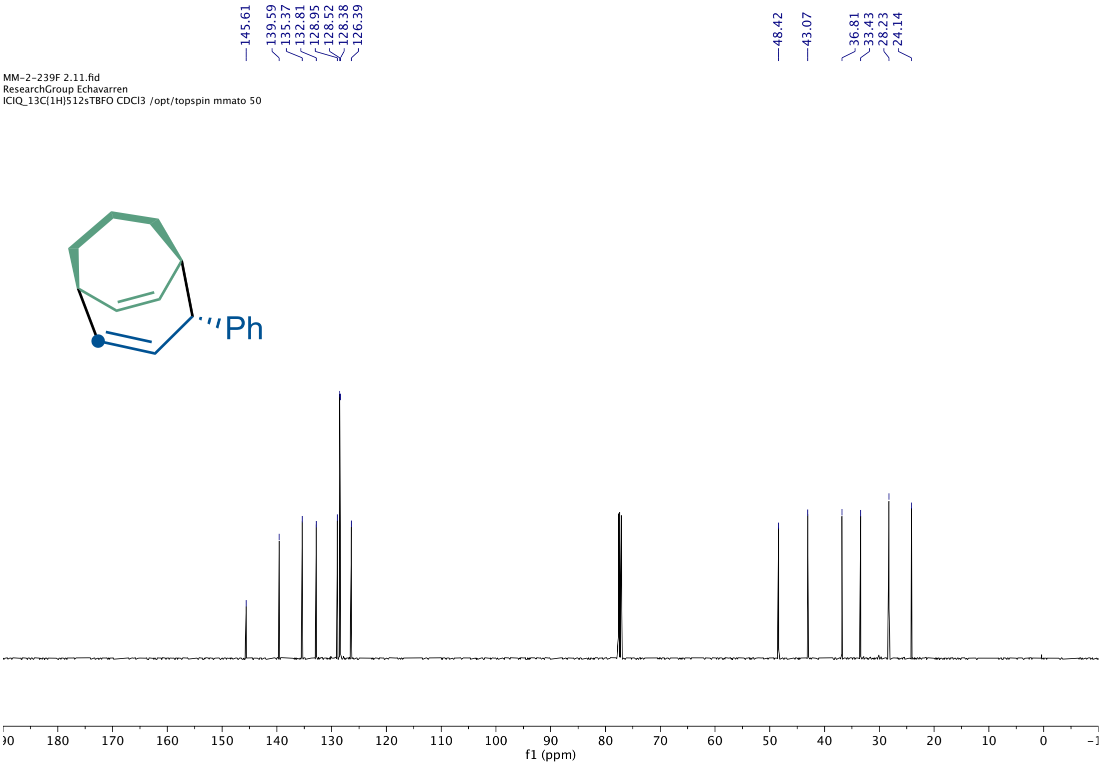

6-Phenylcyclohepta-1,4-diene (3u)

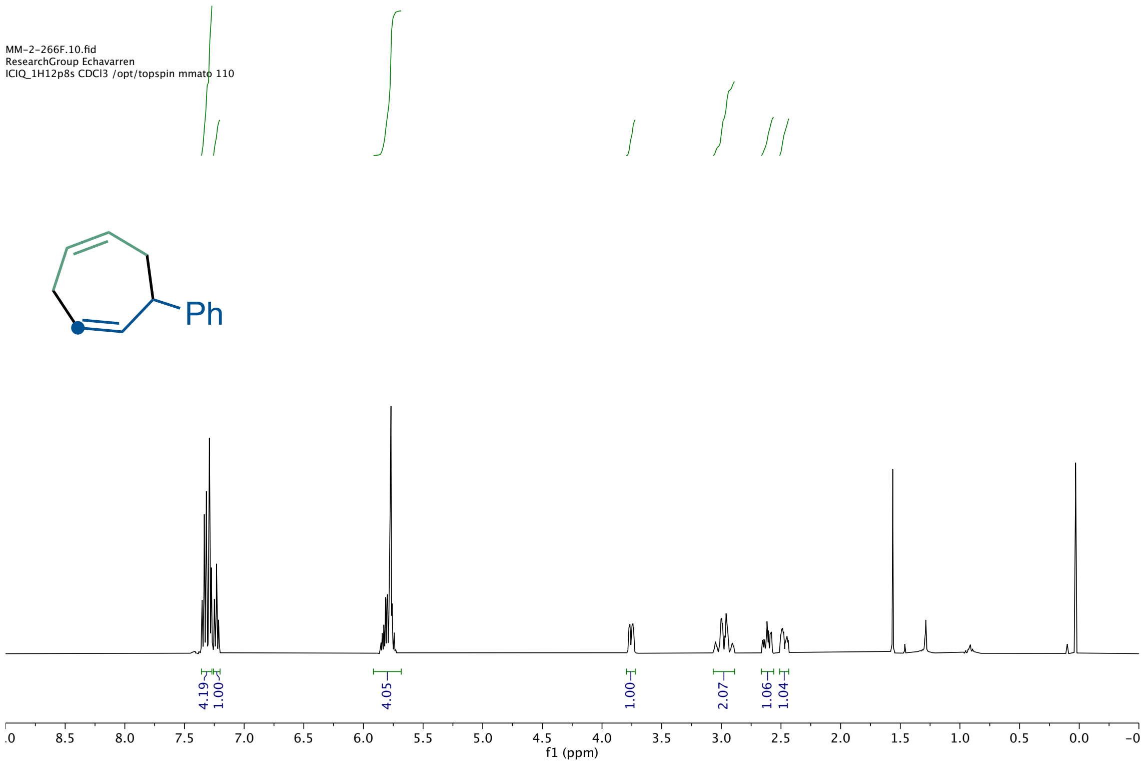

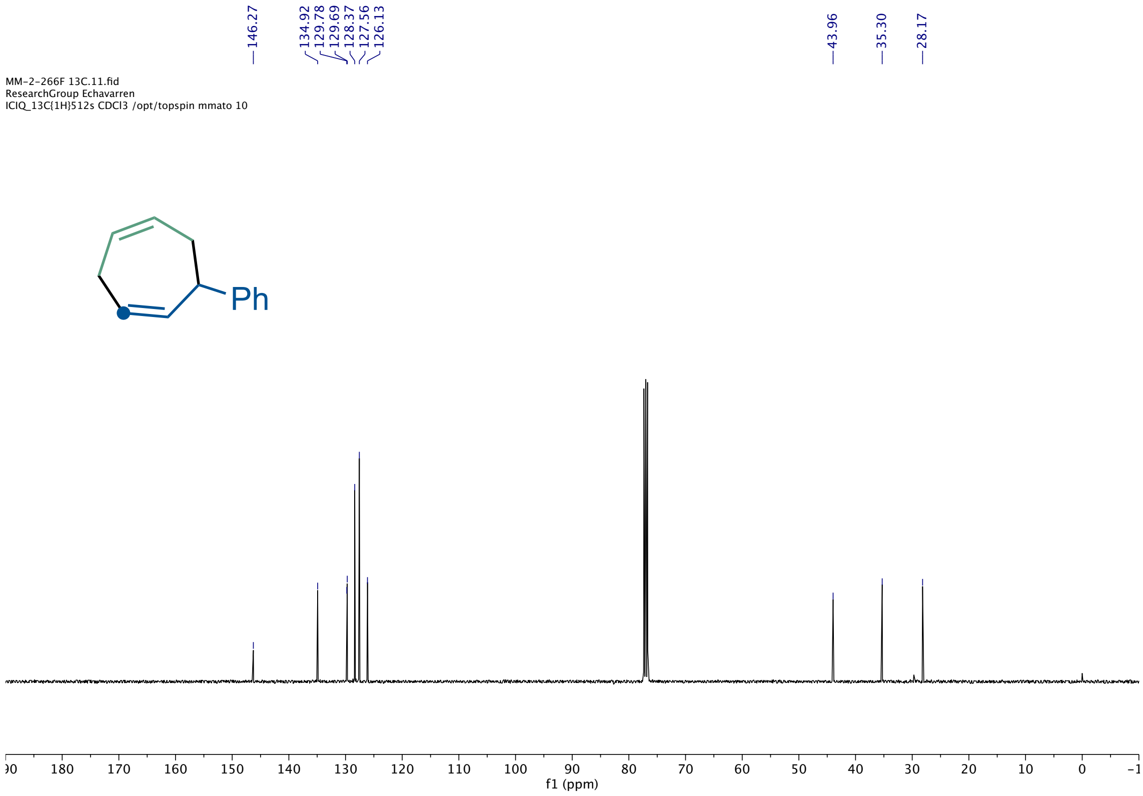

(±)-1-((1*R*,2*R*,5*S*)-Bicyclo[3.2.2]nona-3,6-dien-2-yl)-4-((1*S*,2*S*,5*R*)-bicyclo[3.2.2]nona-3,6-dien-2-yl)benzene (3v)

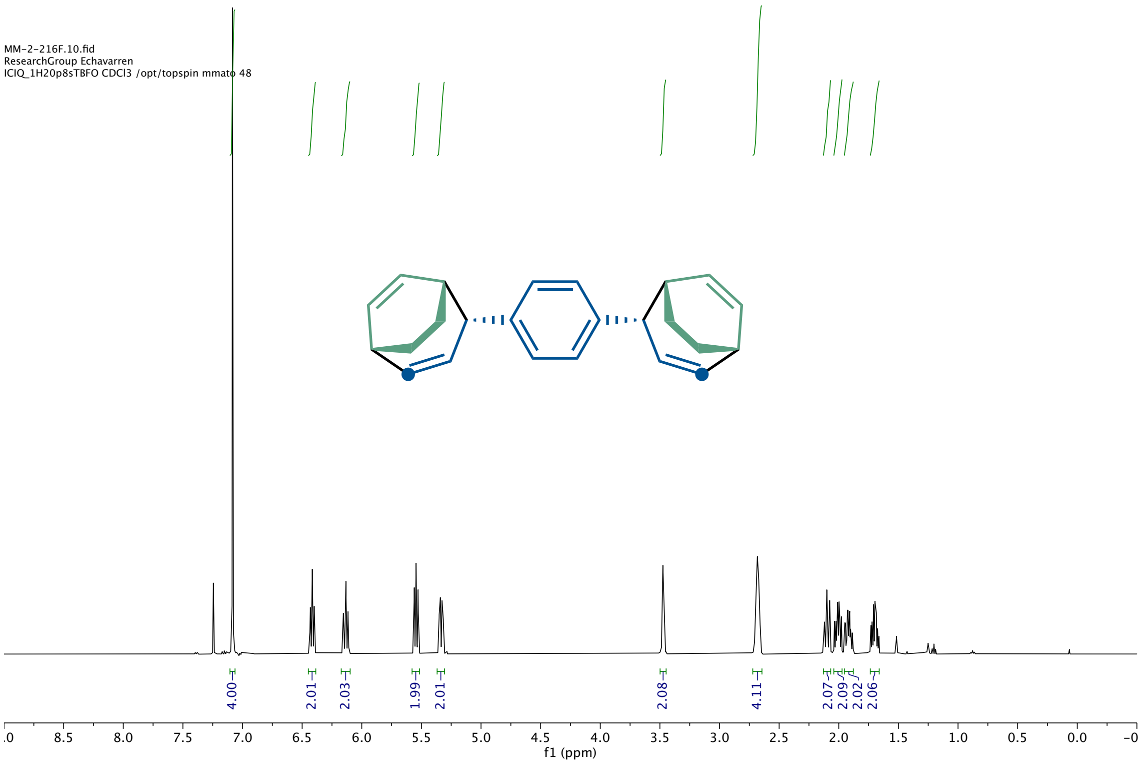

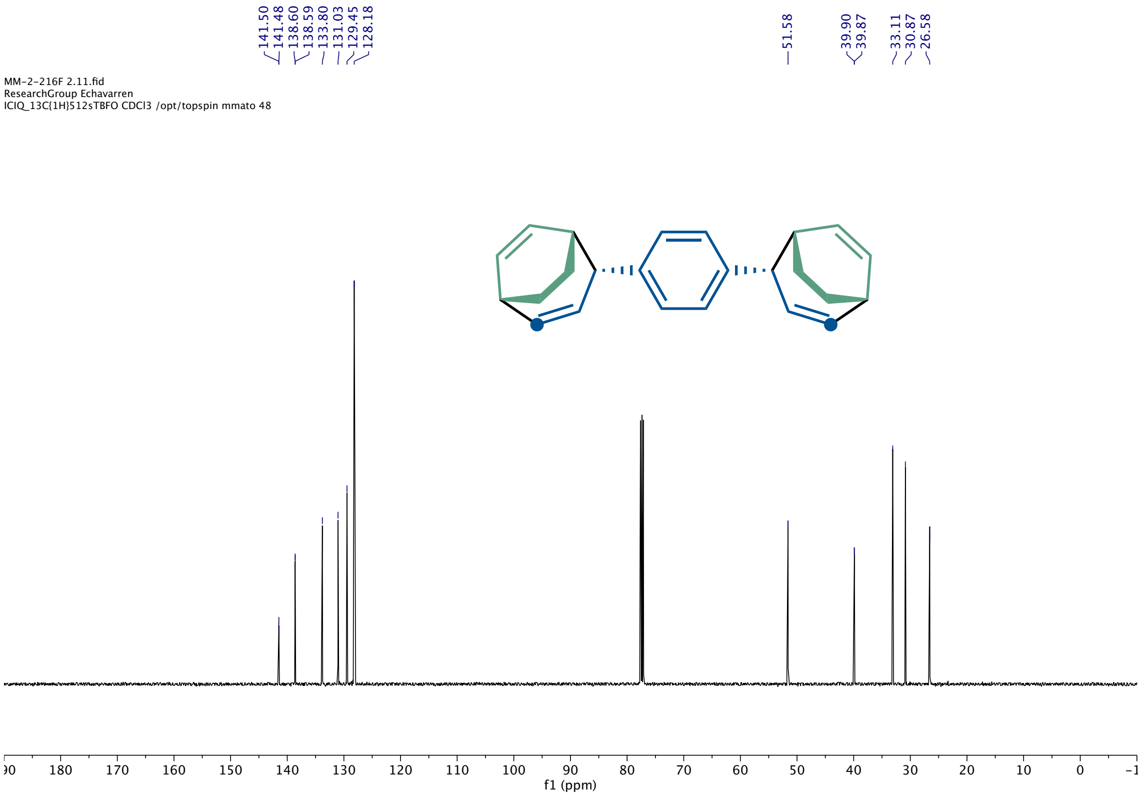

cis-((_E_)-2-(2,2-dimethyl-3-(2-methylprop-1-en-1-yl)cyclopropyl)vinyl)benzene (3w')

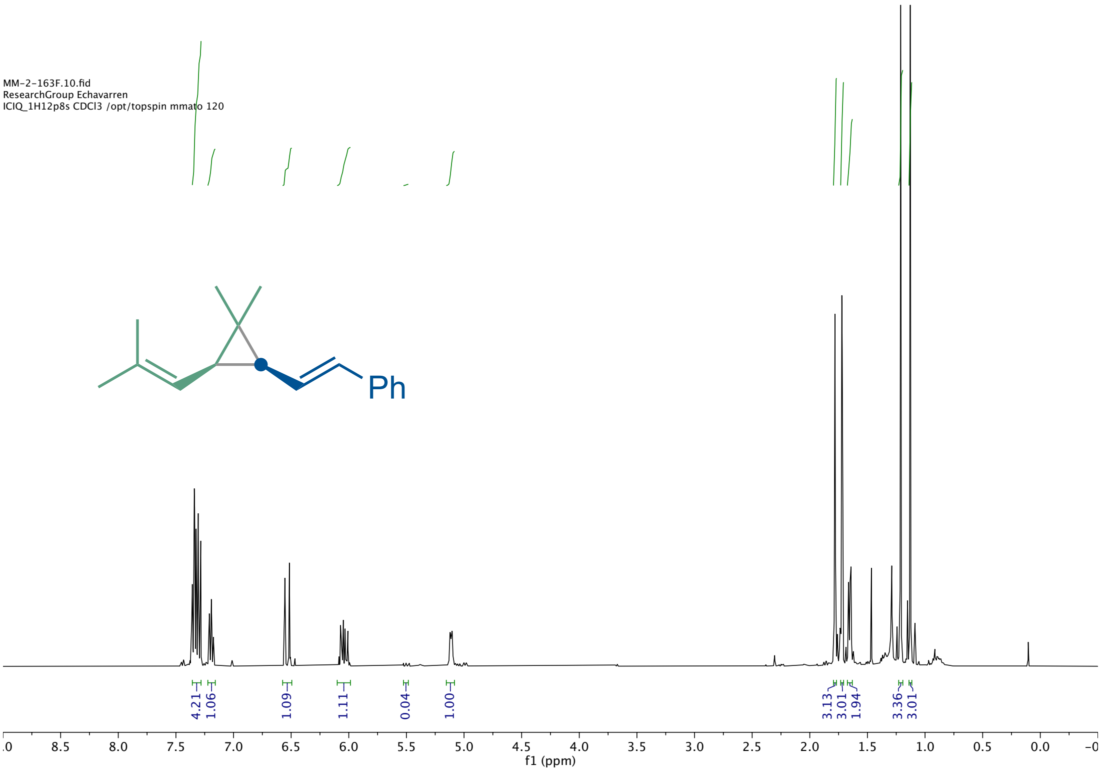

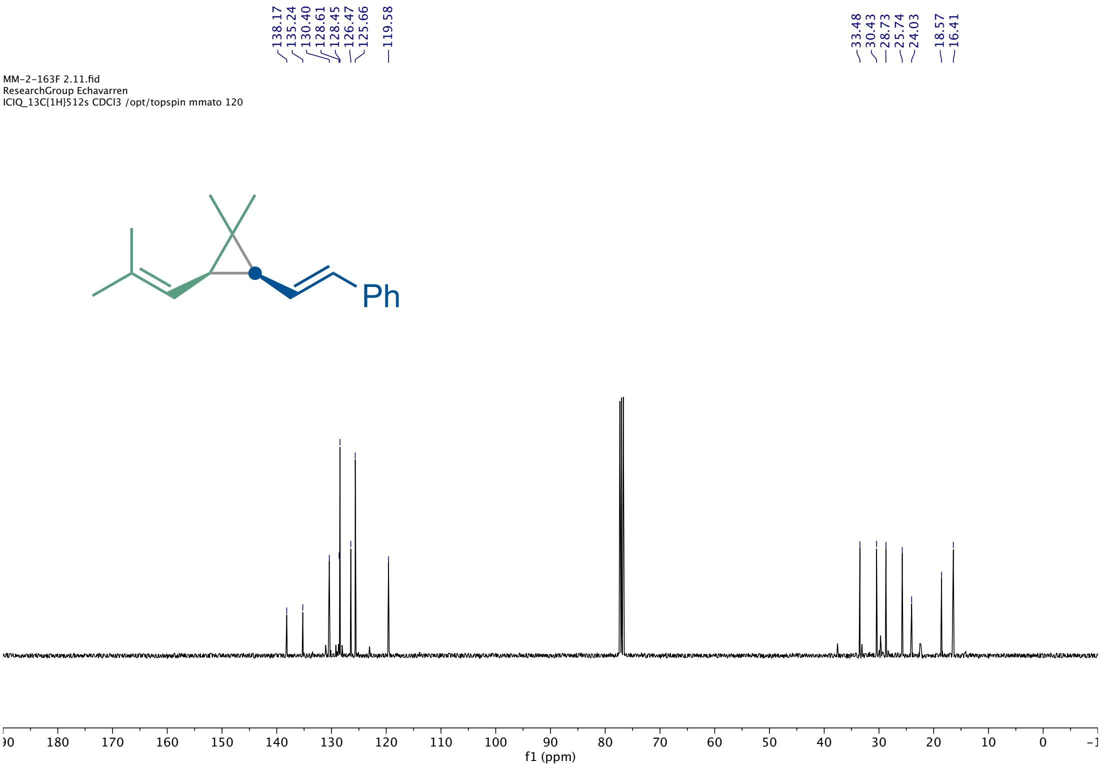

(*endo*,*Z*)-9-((*E*)-Styryl)bicyclo[6.1.0]non-2-ene (3x')

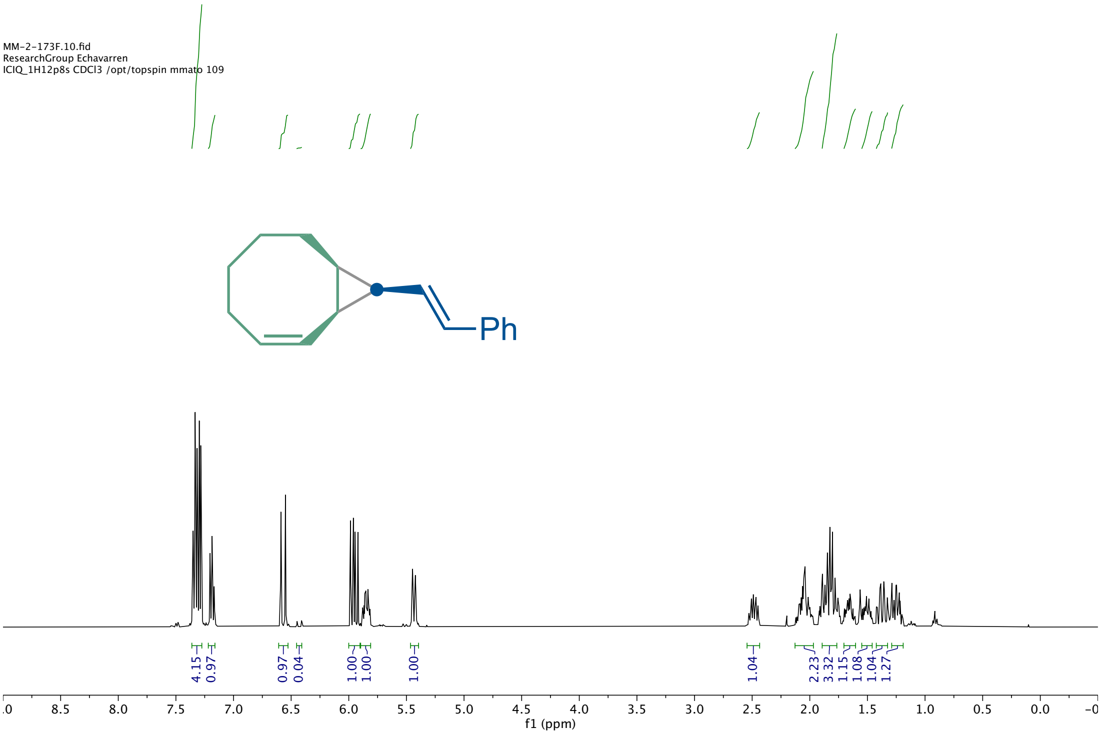

(±)-(1*R*,6*S*,9*R*,*Z*)-9-Phenylbicyclo[4.3.2]undeca-7,10-diene (3x)

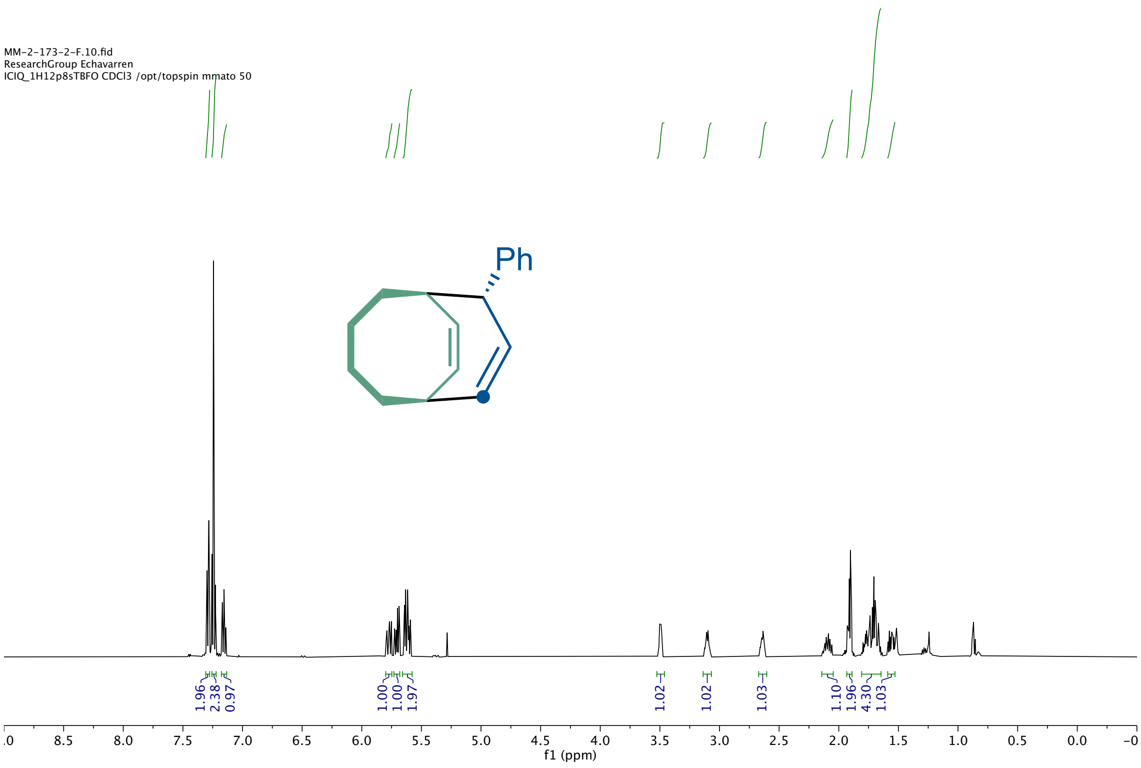

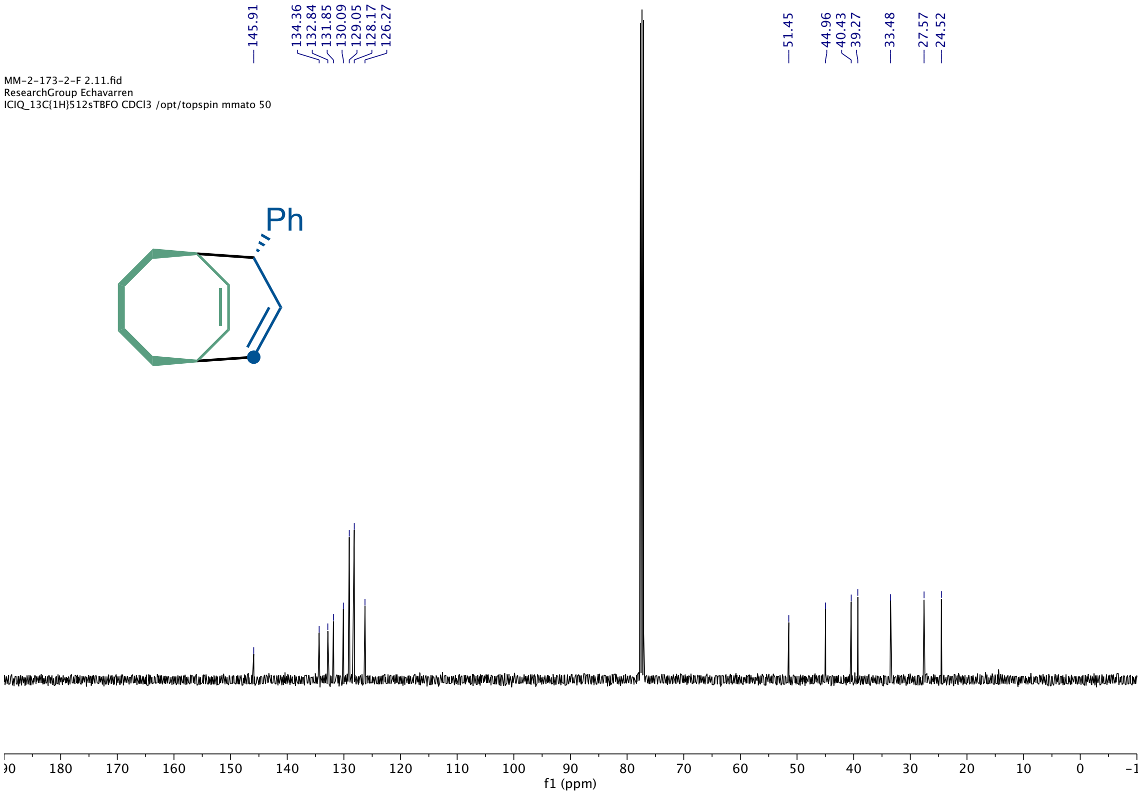

4-((*E*)-2-((*endo*,*Z*)-Bicyclo[6.1.0]non-2-en-9-yl)vinyl)phenyl 1-ferrocenoylate (3y')

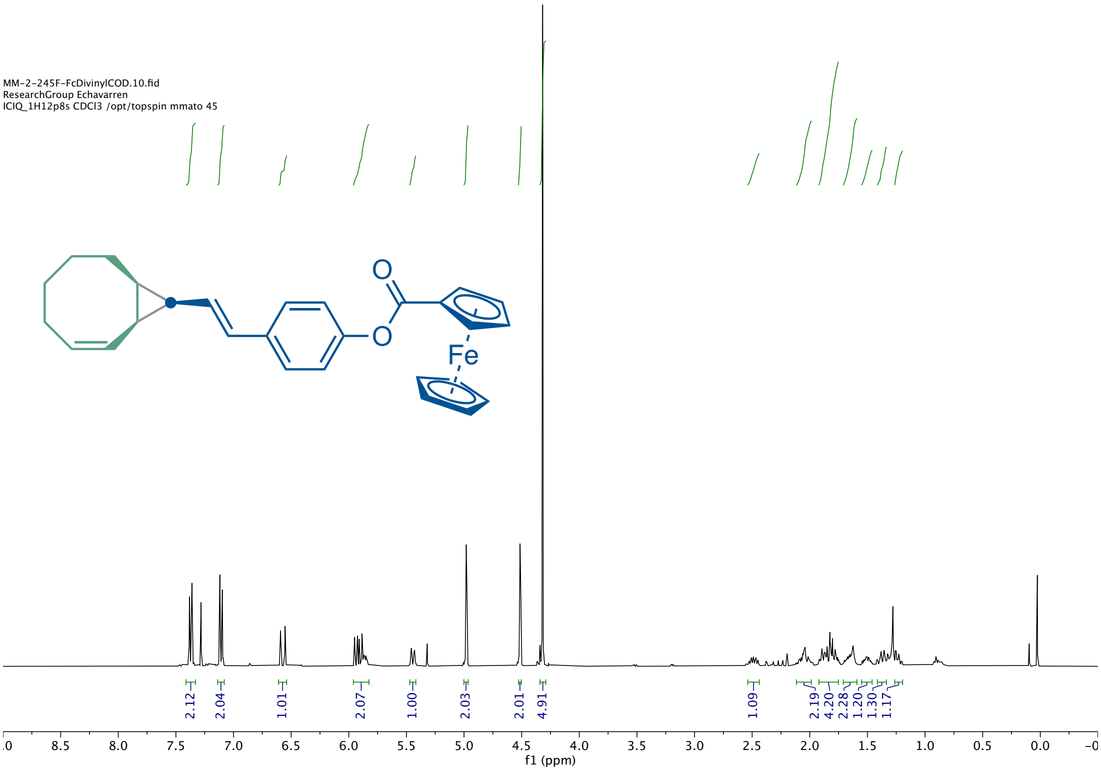

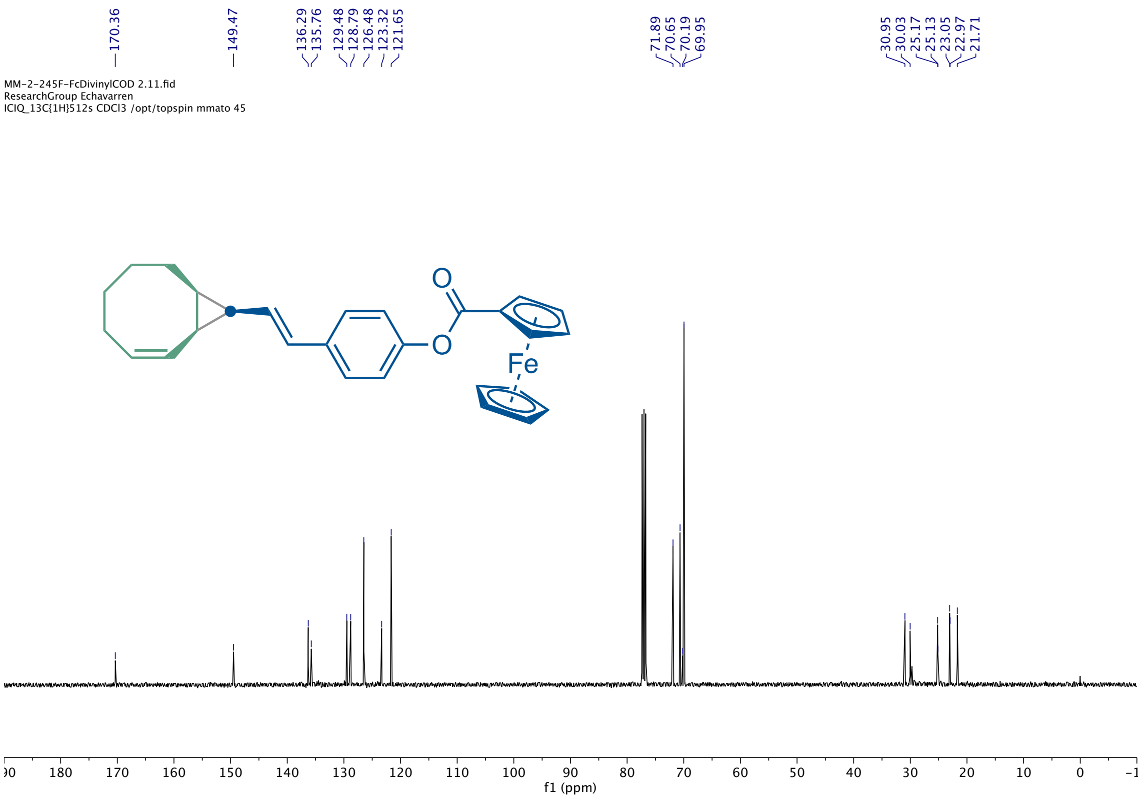

(±)-4-((1*S*,6*R*,7*R*,*Z*)-Bicyclo[4.3.2]undeca-8,10-dien-7-yl)phenyl ferrocenoylate (3y)

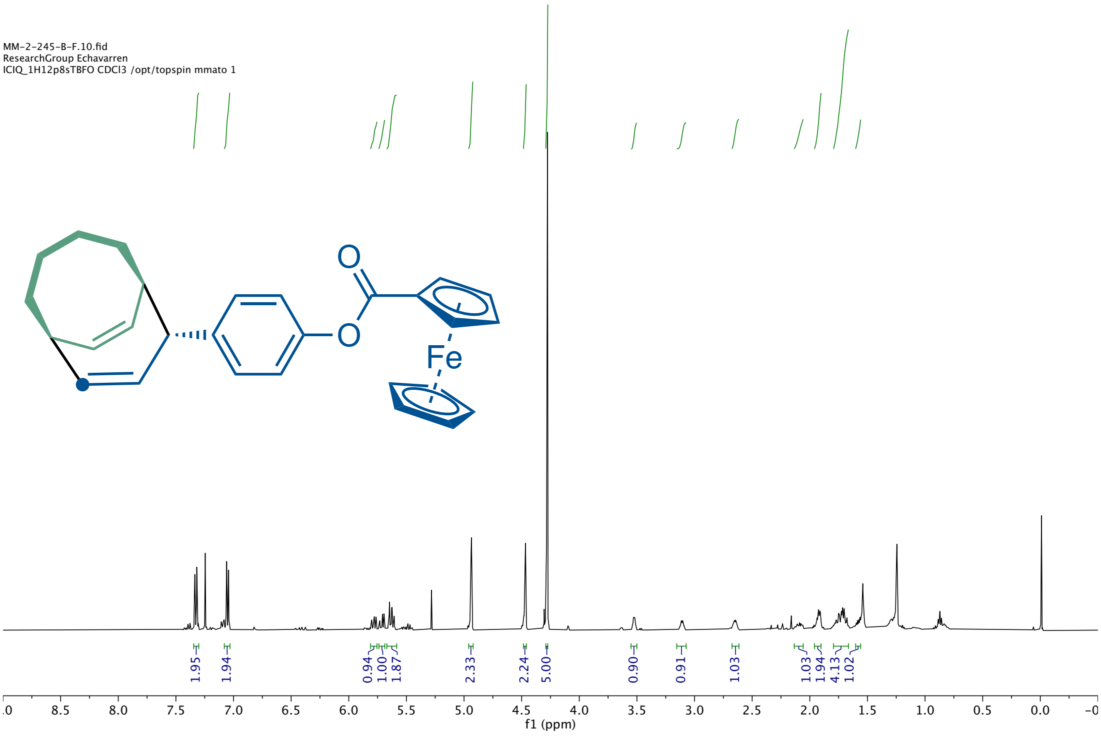

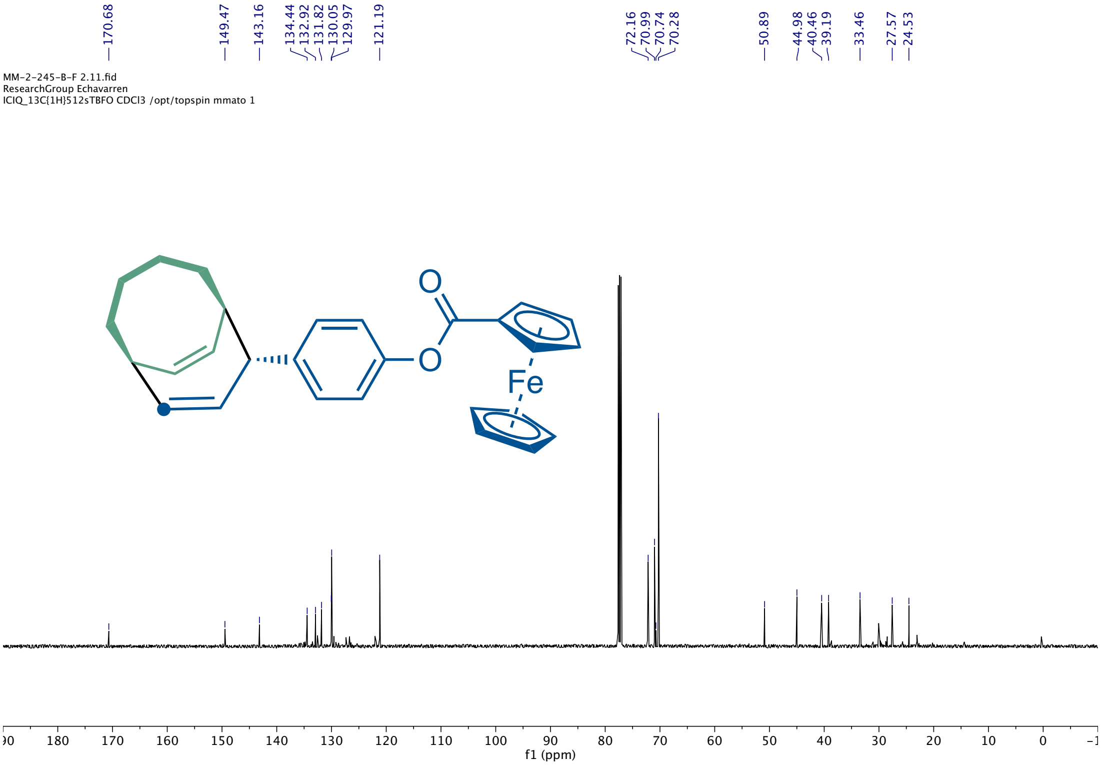

(±)-Dictyopterene C’ (5)

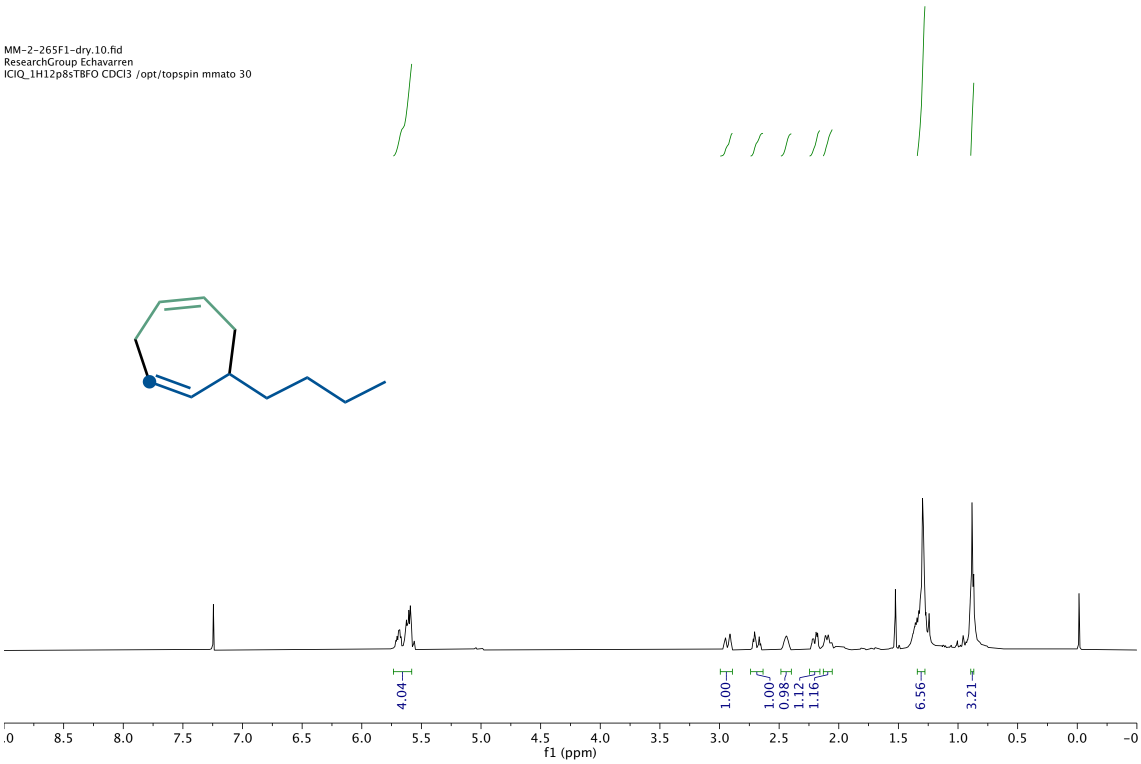

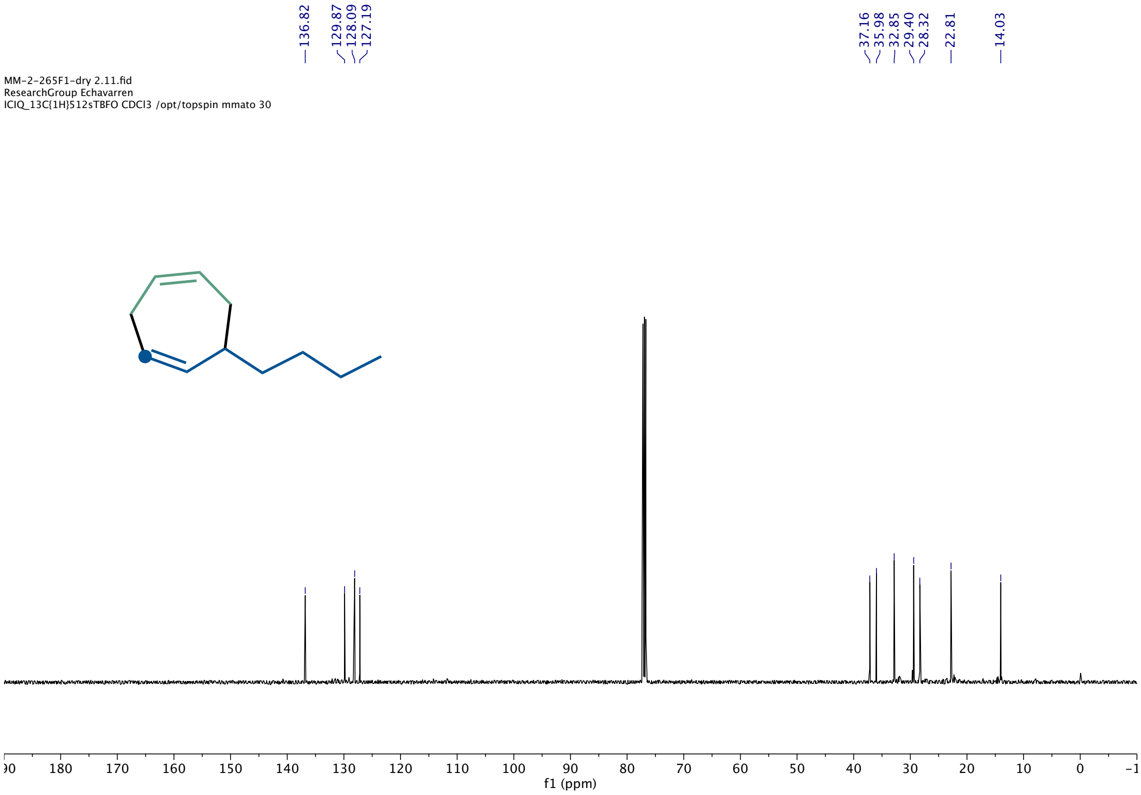

(±)-(1*S*,4*S*,5*S*)-4-(2-Ethynylphenyl)-1,5,6,7,8-pentamethylbicyclo[3.2.1]octa-2,6-diene (3o)

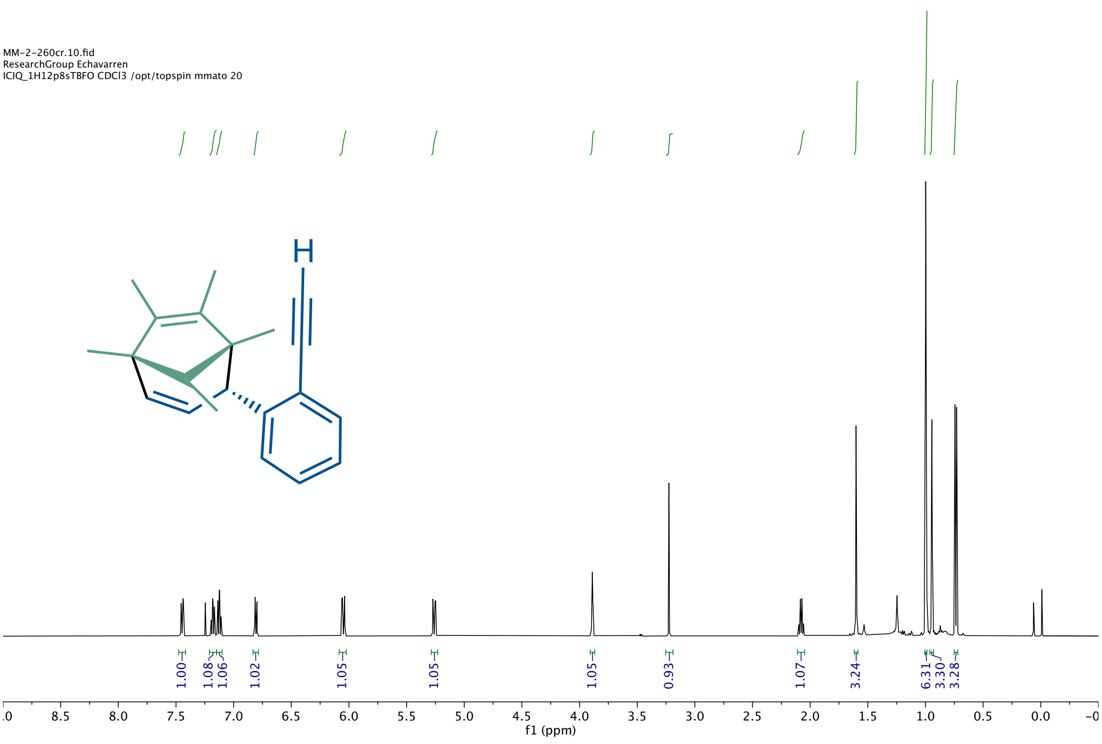

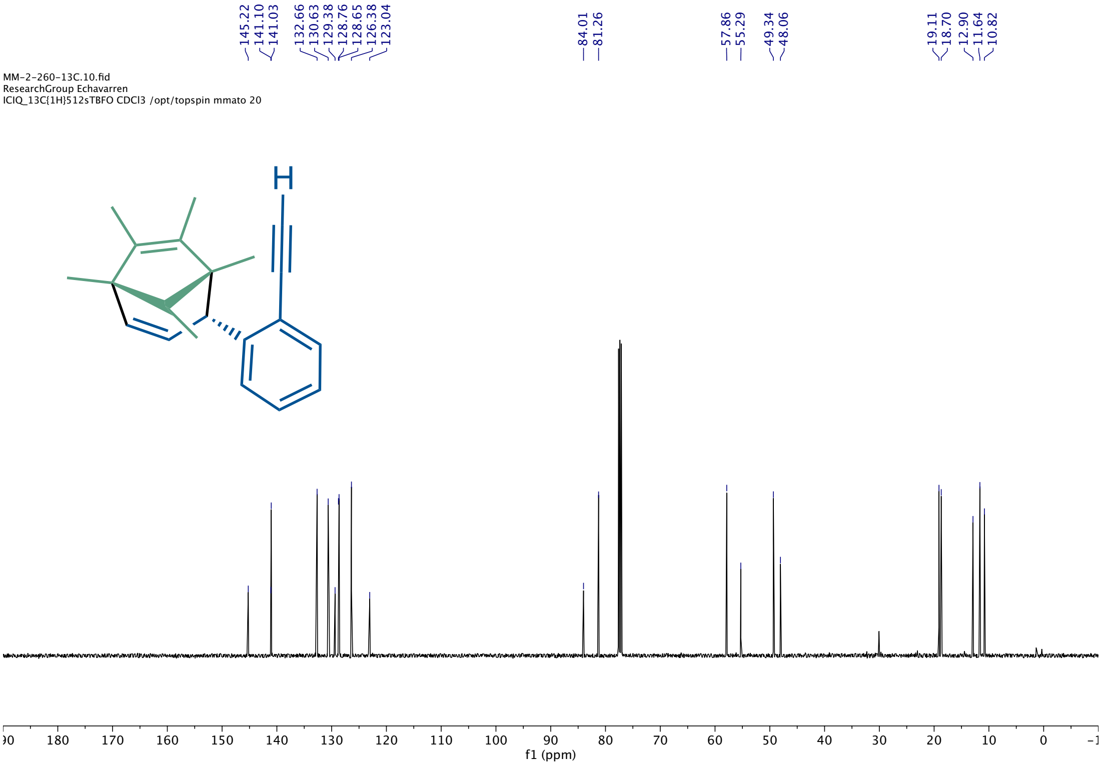

(±)-(1*S*,4*S*,5*S*)-4-(3',5'-Dimethoxy-[1,1'-biphenyl]-3-yl)-1,5,6,7,8-pentamethylbicyclo [3.2.1]octa-2,6-diene (3z)

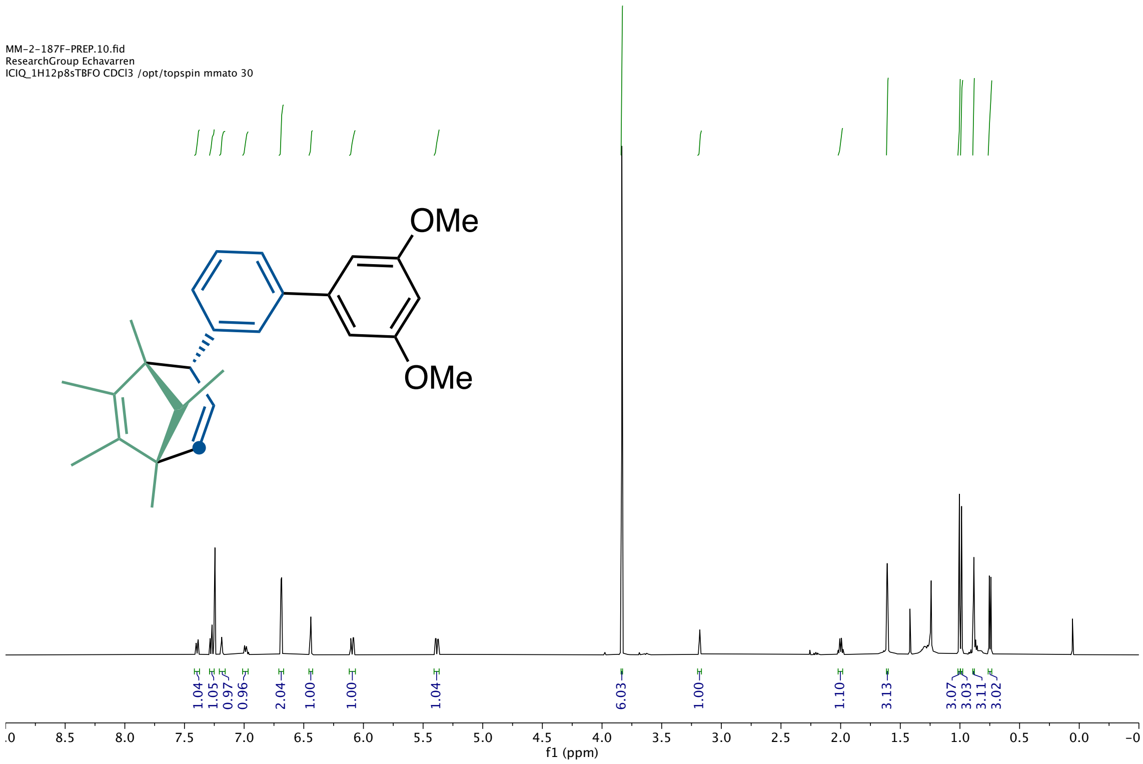

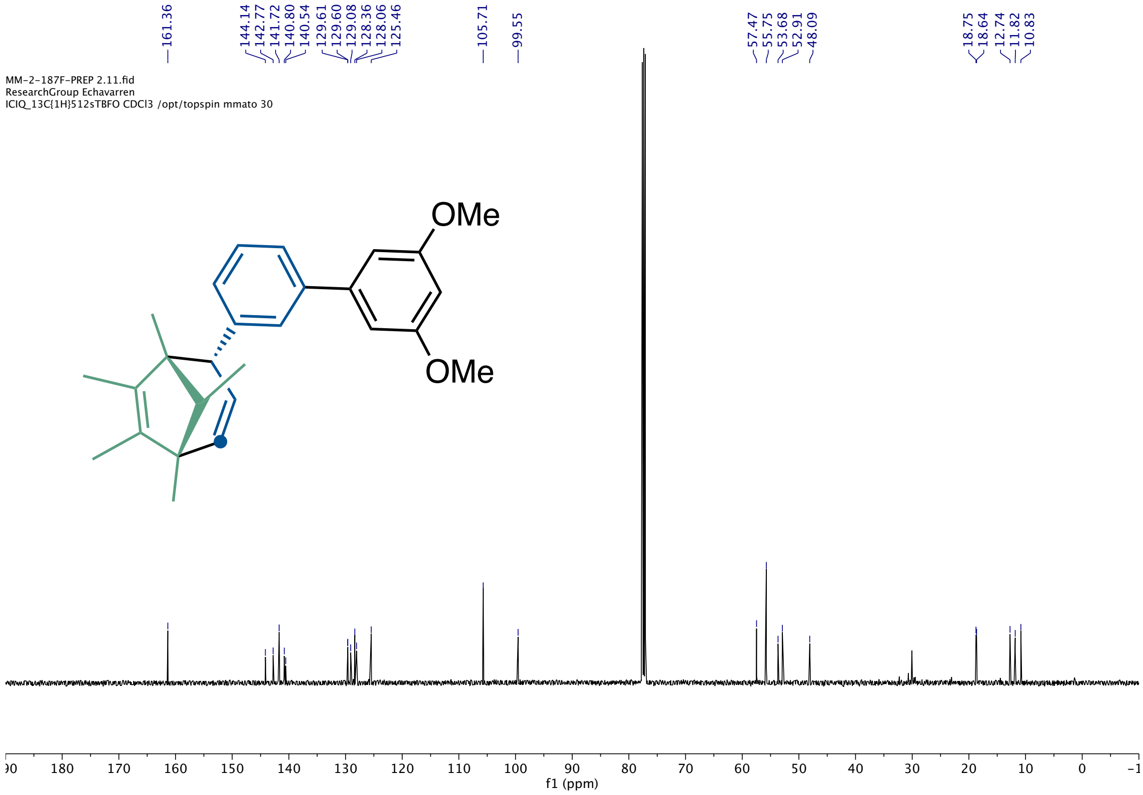

(±)-3,5-Dimethyl-4-((2-((1S,2S,5S)-1,5,6,7,8-pentamethylbicyclo[3.2.1]octa-3,6-dien-2-yl)phenyl)ethynyl)isoxazole (3aa)

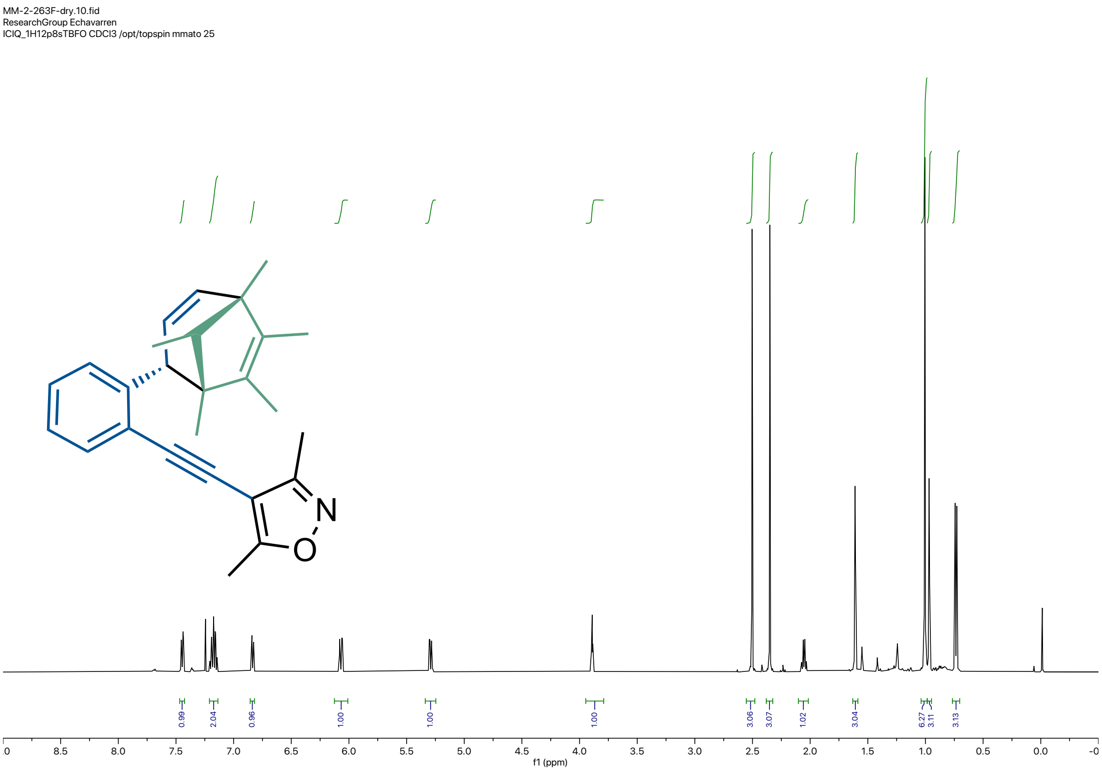

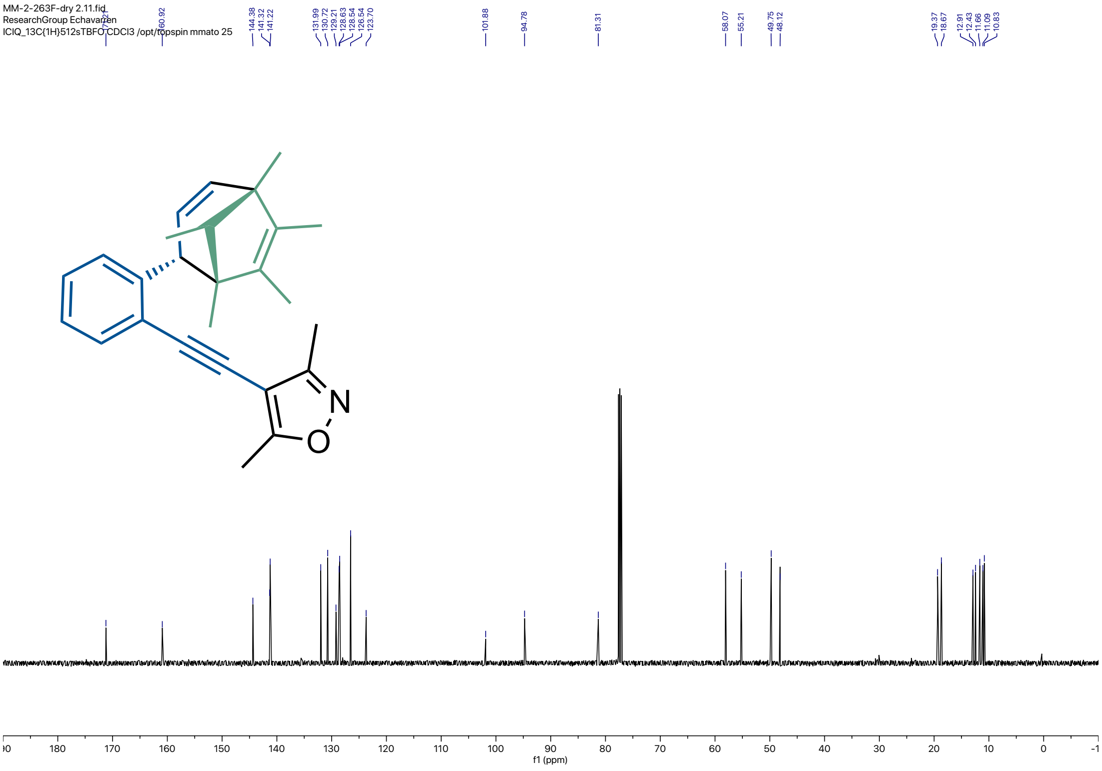

(±)-(1*R*,2*R*,5*S*)-2-Phenylbicyclo[3.2.2]nonane (4)

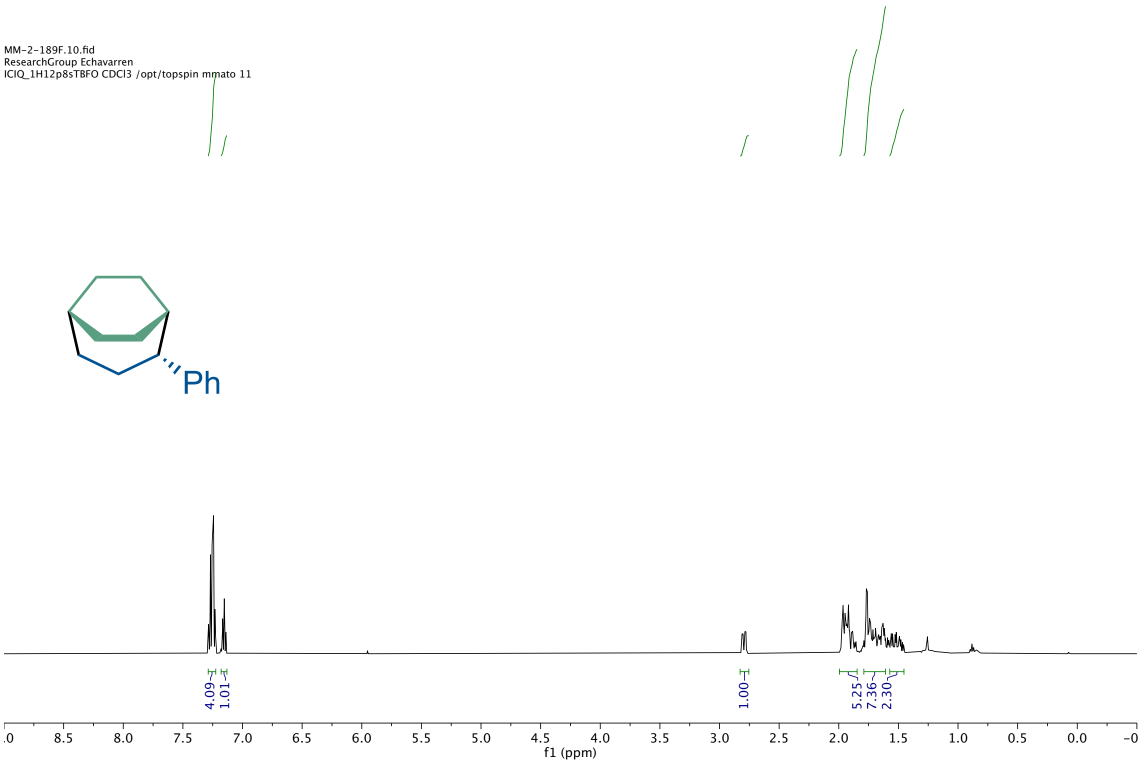

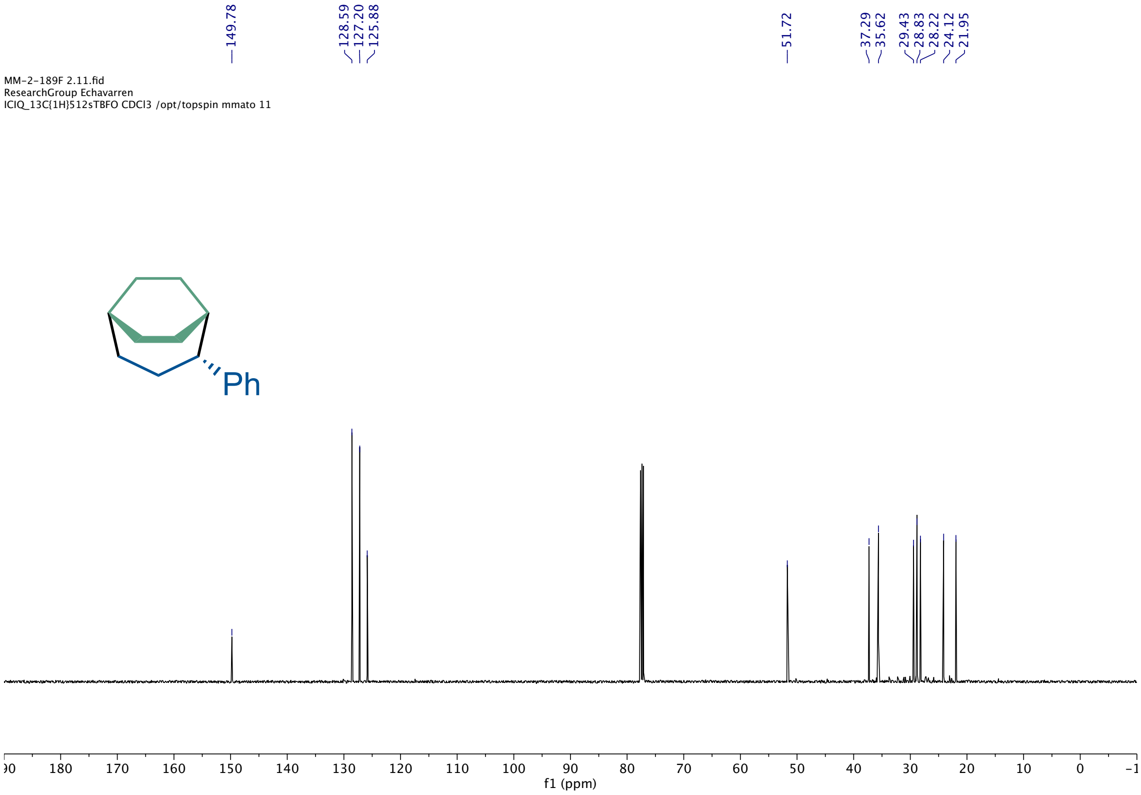

^{1}H NMR (400 MHz, $CDCl_3$) of **(7a)**

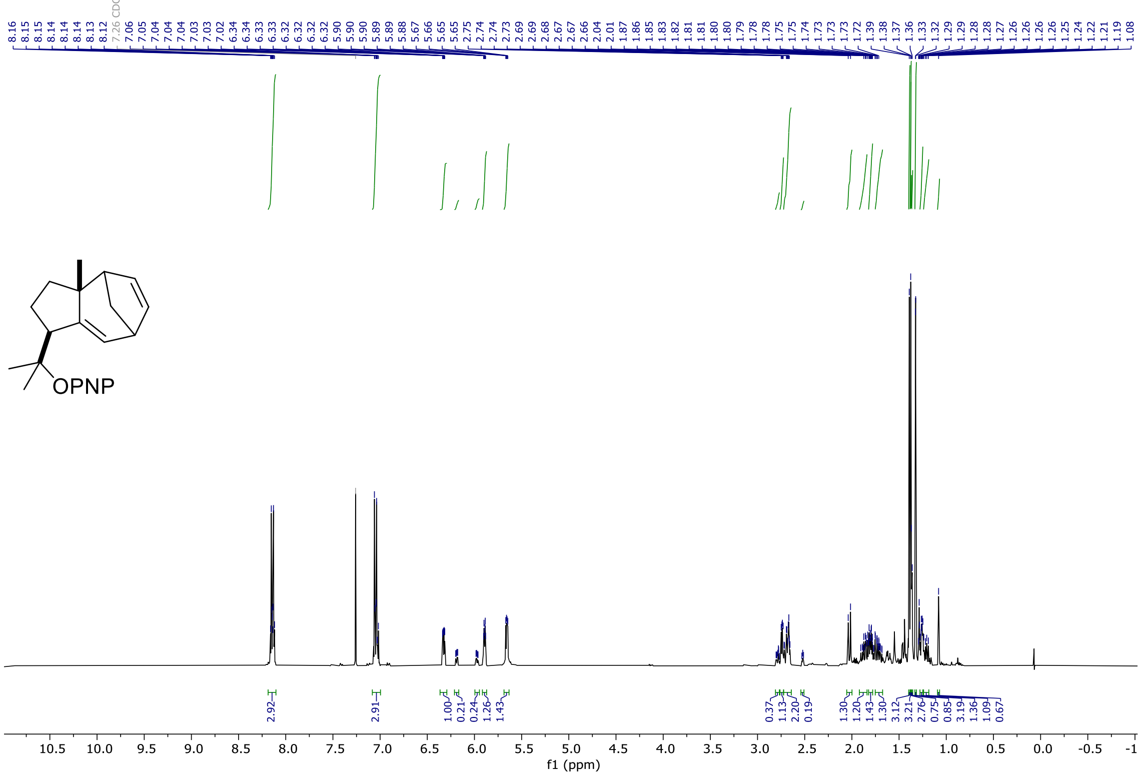

^{13}C NMR (101 MHz, $CDCl_3$) of **(7a)**

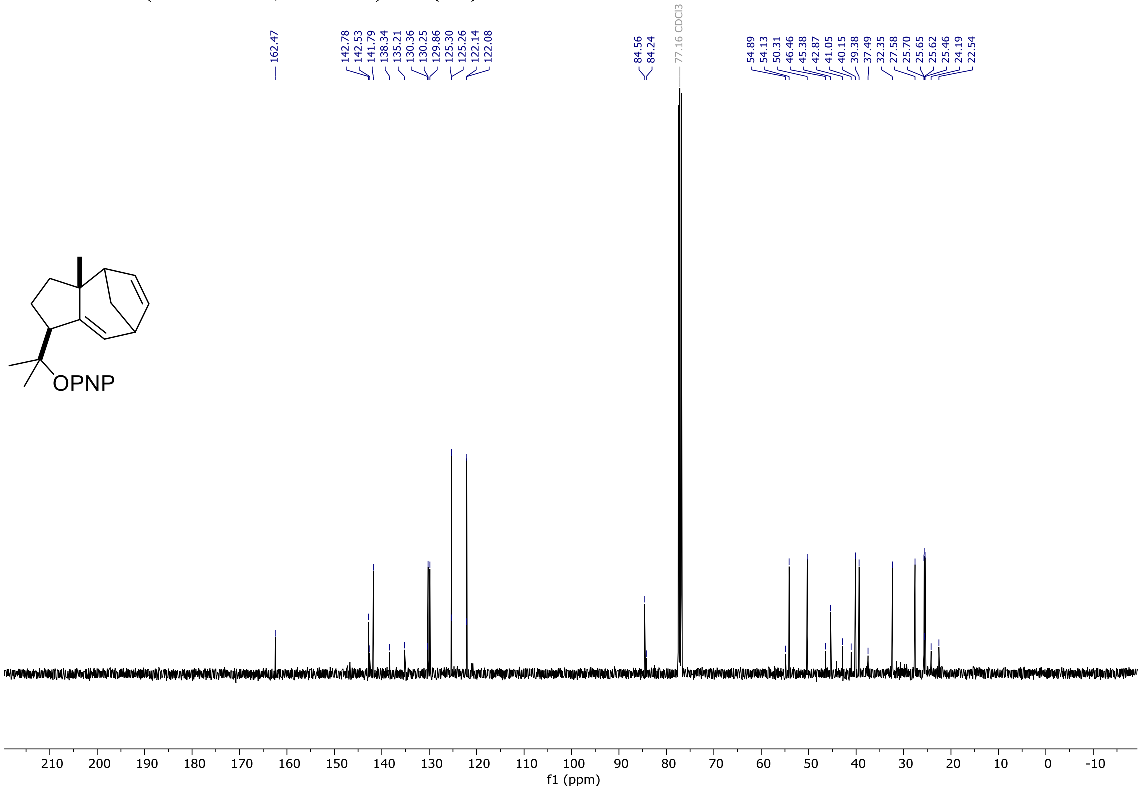

^{1}H NMR (500 MHz, $CDCl_3$) of **(7a)** and **(8a)**

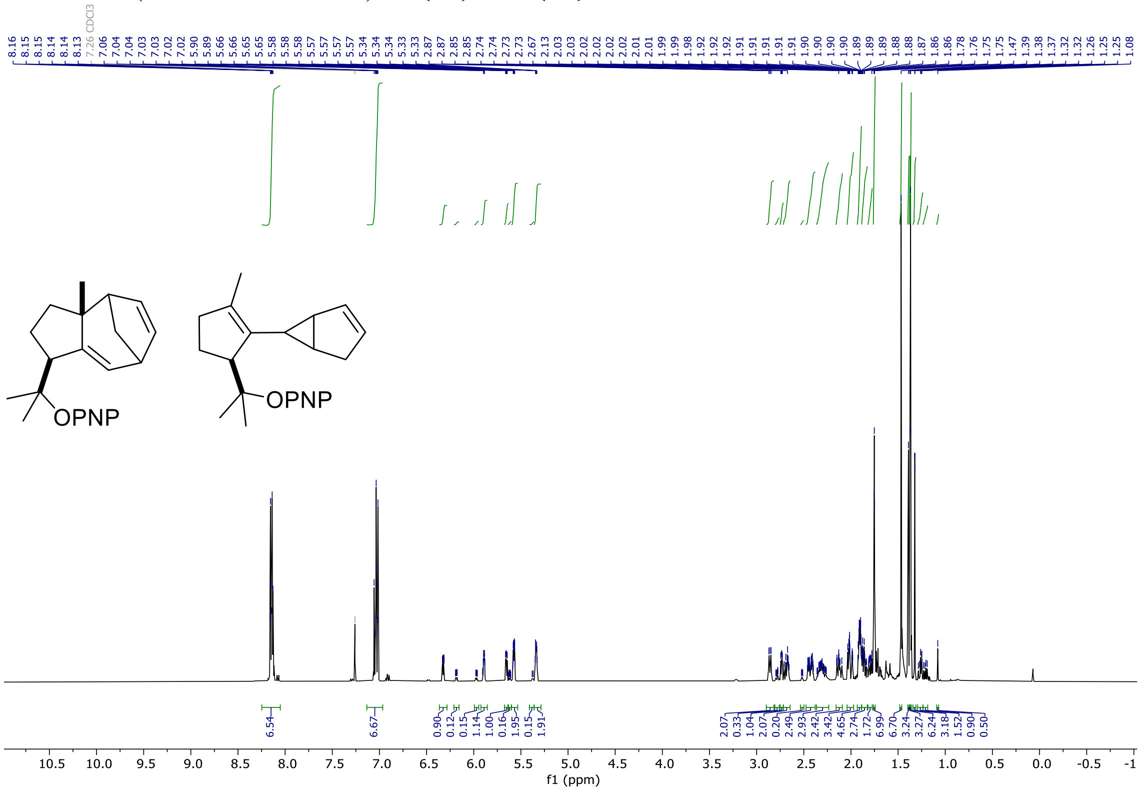

^{13}C NMR (126 MHz, $CDCl_3$) of **(7a)** and **(8a)**

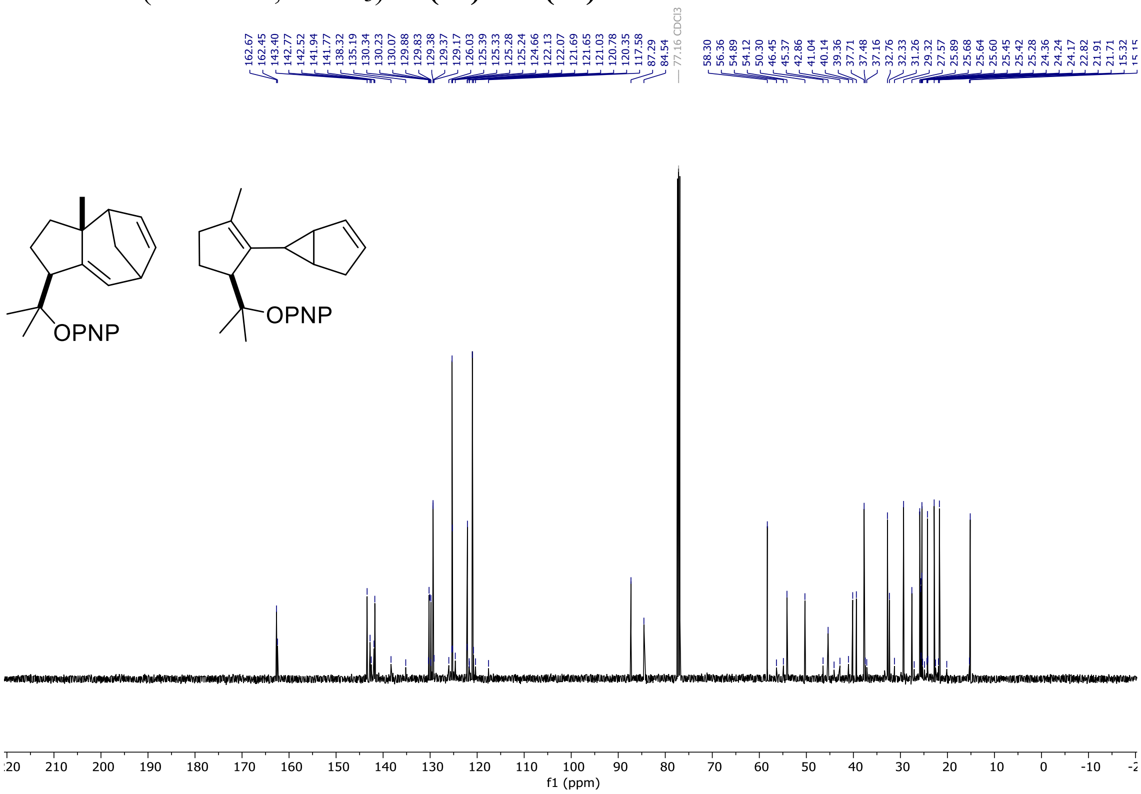

^{1}H NMR (500 MHz, $CDCl_3$) of **(7b)**

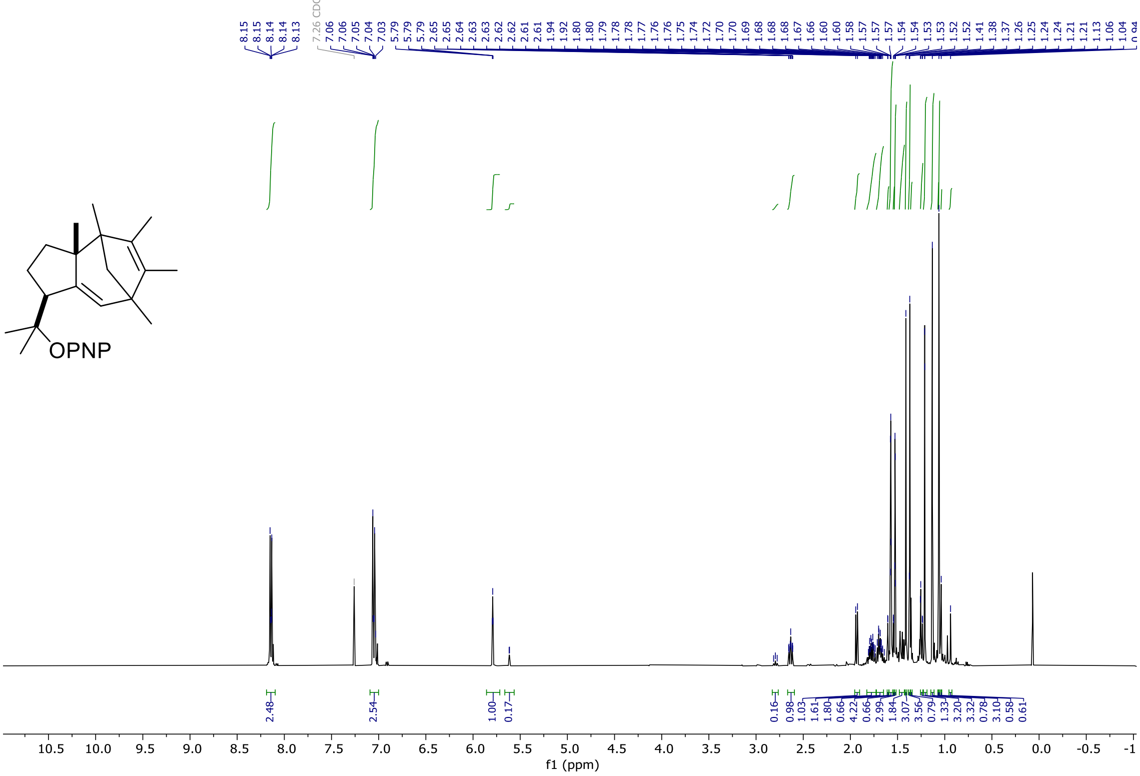

^{13}C NMR (126 MHz, $CDCl_3$) of **(7b)**

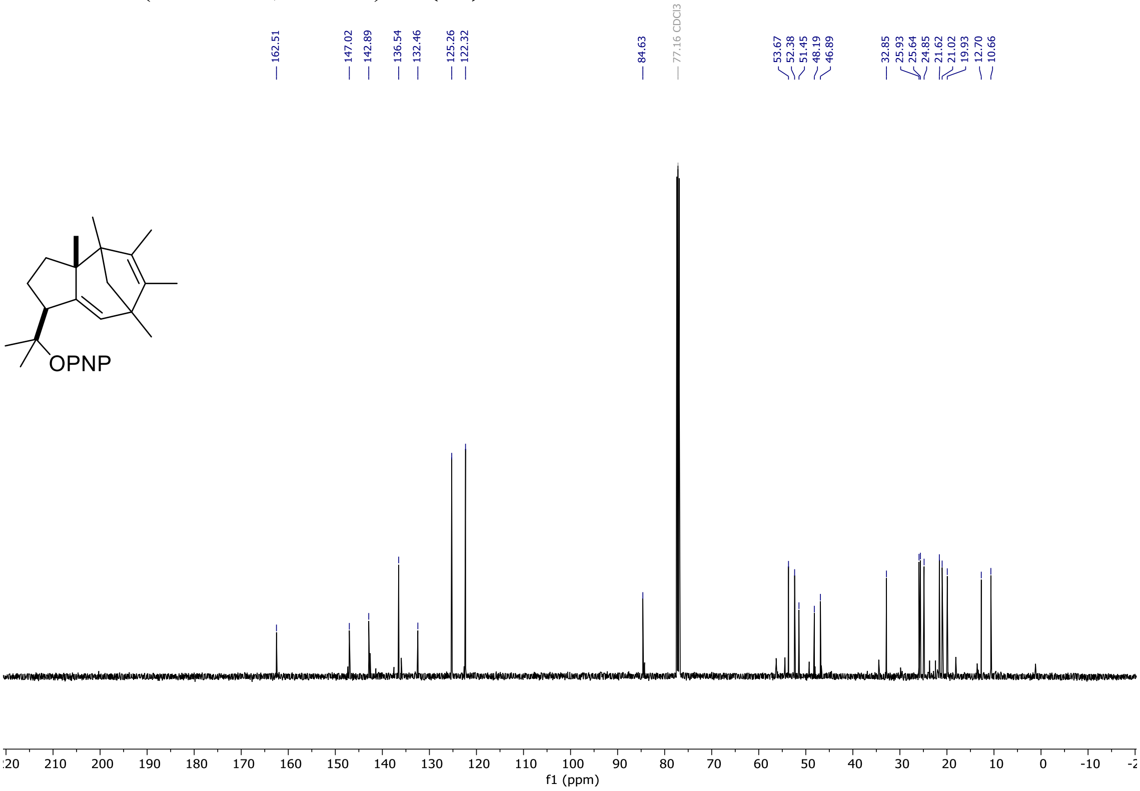

^{1}H NMR (500 MHz, $CDCl_3$) of **(7c)**

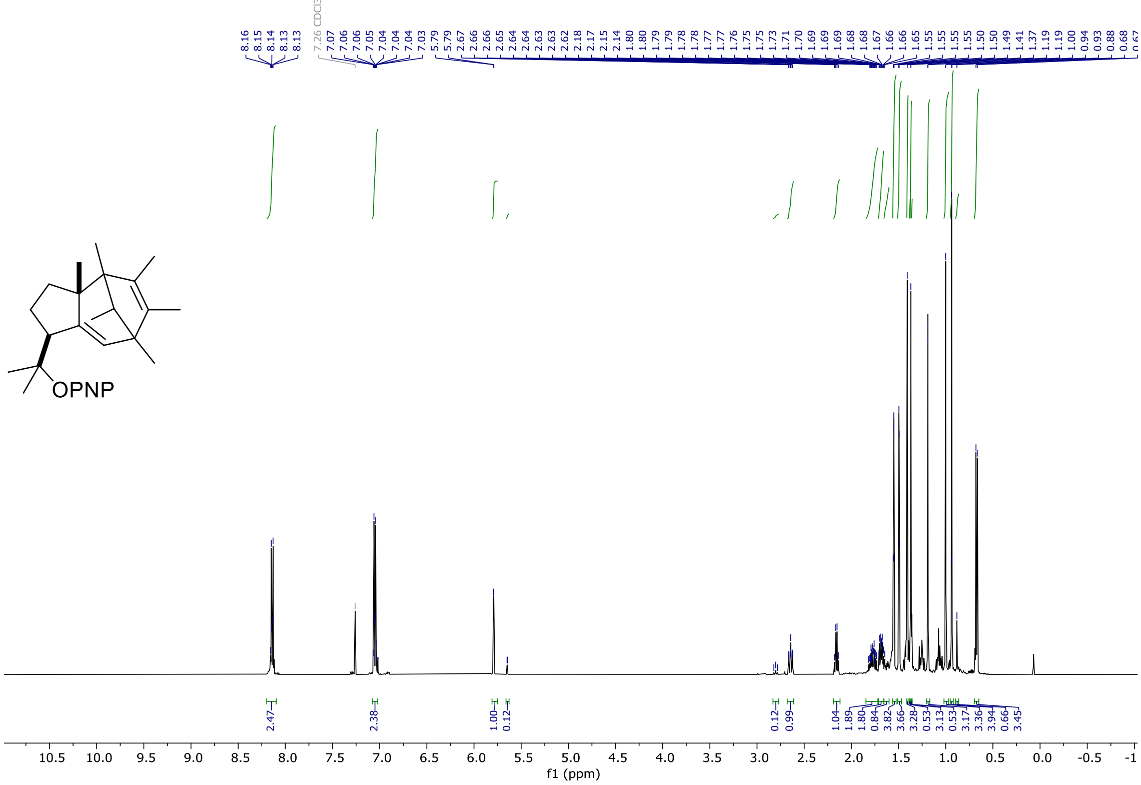

^{13}C NMR (126 MHz, $CDCl_3$) of **(7c)**

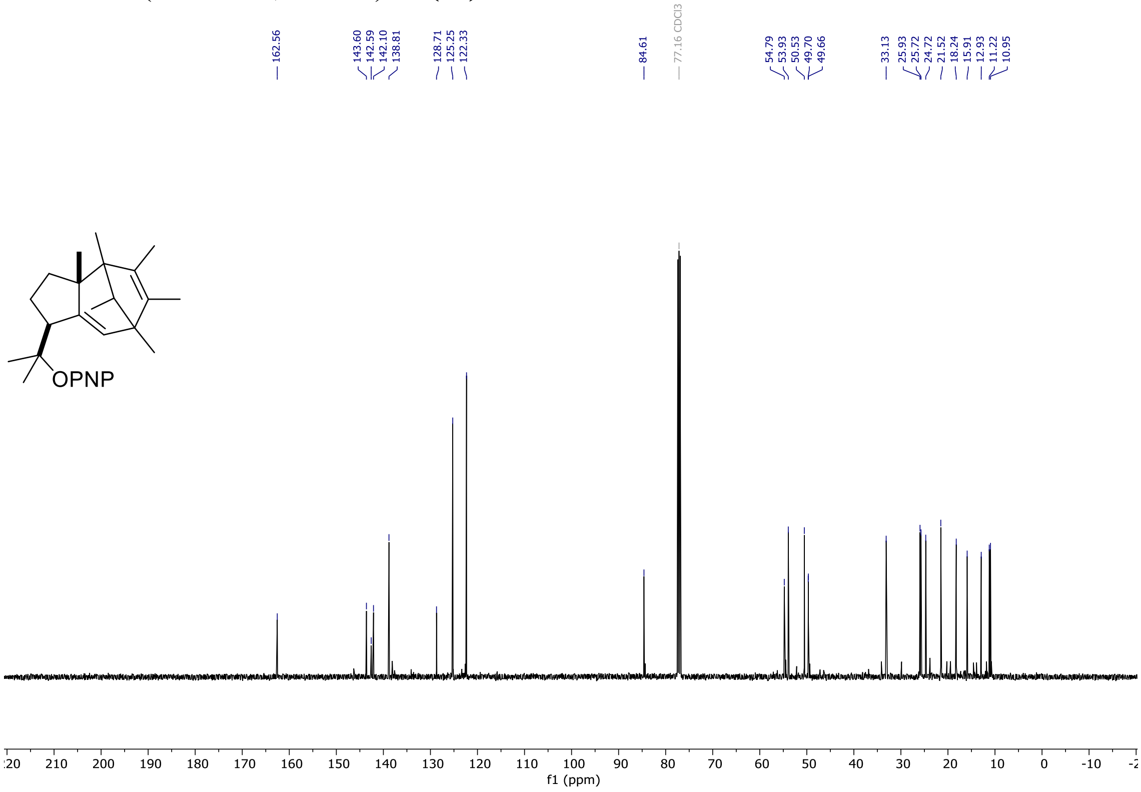

^{1}H NMR (400 MHz, $CDCl_3$) of **(8d)**

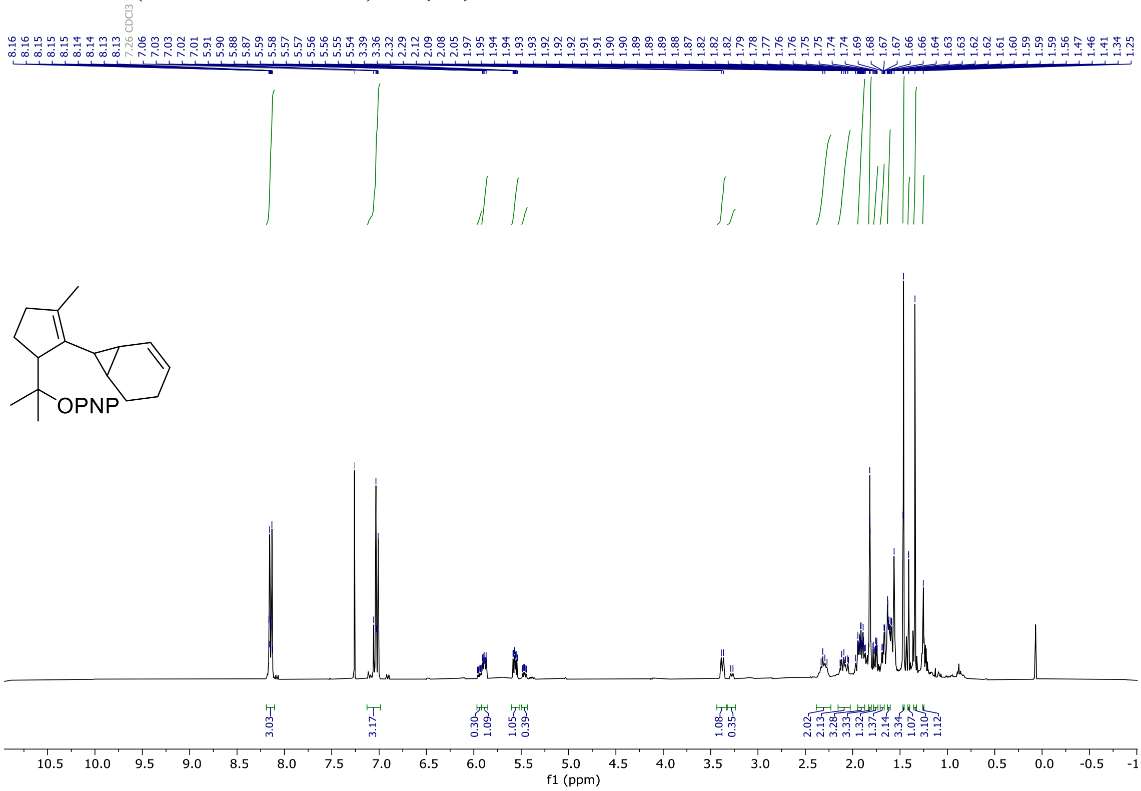

^{13}C NMR (101 MHz, $CDCl_3$) of **(8d)**

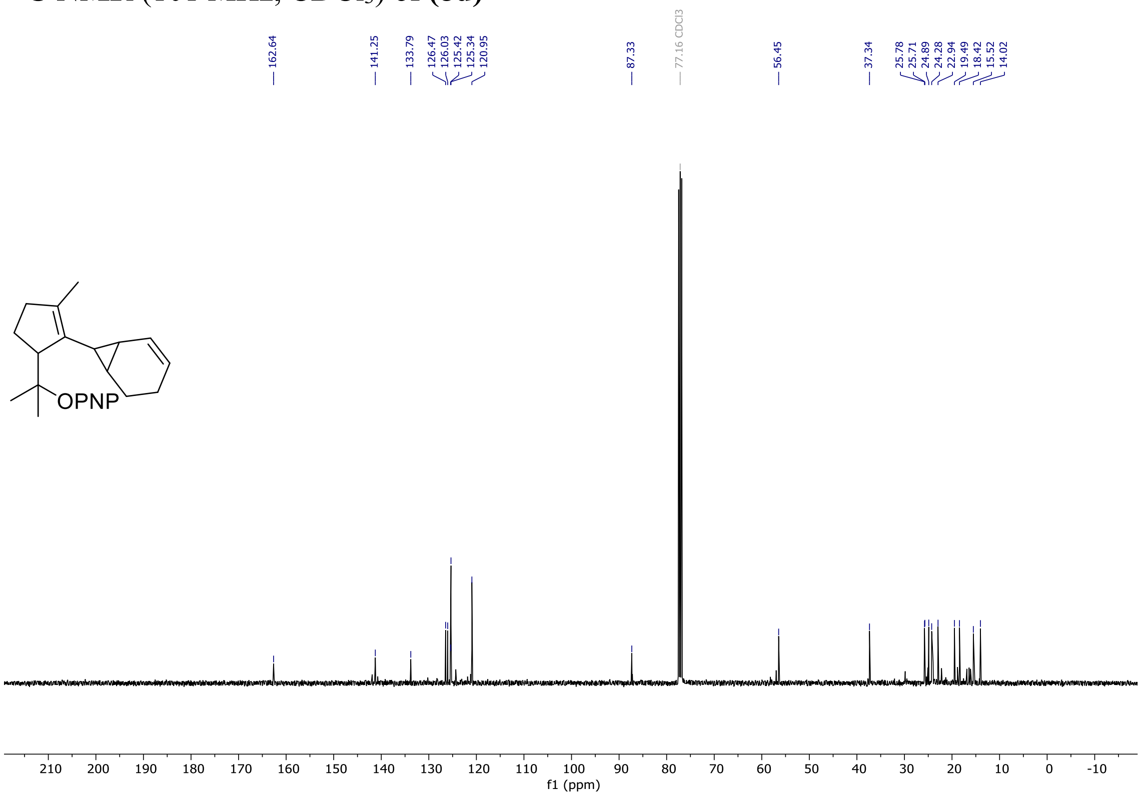

^{1}H NMR (500 MHz, $CDCl_3$) of **(7e) and (8e)**

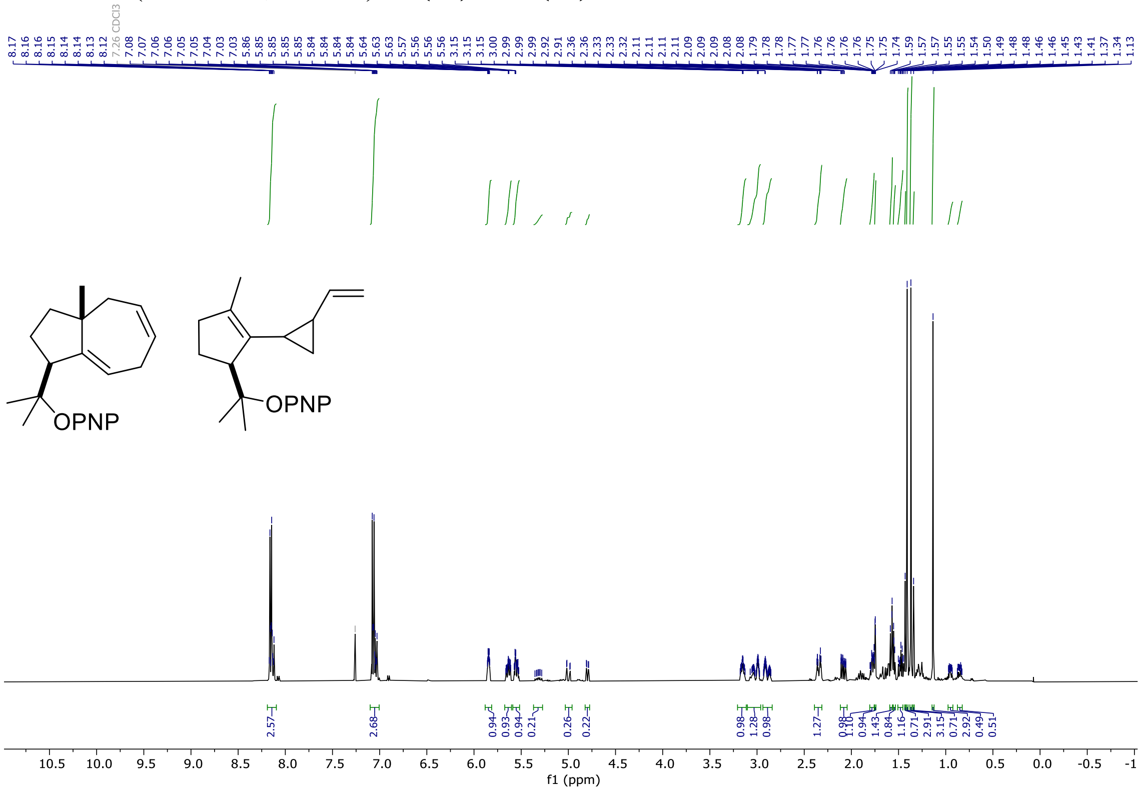

^{13}C NMR (126 MHz, $CDCl_3$) of **(7e) and (8e)**

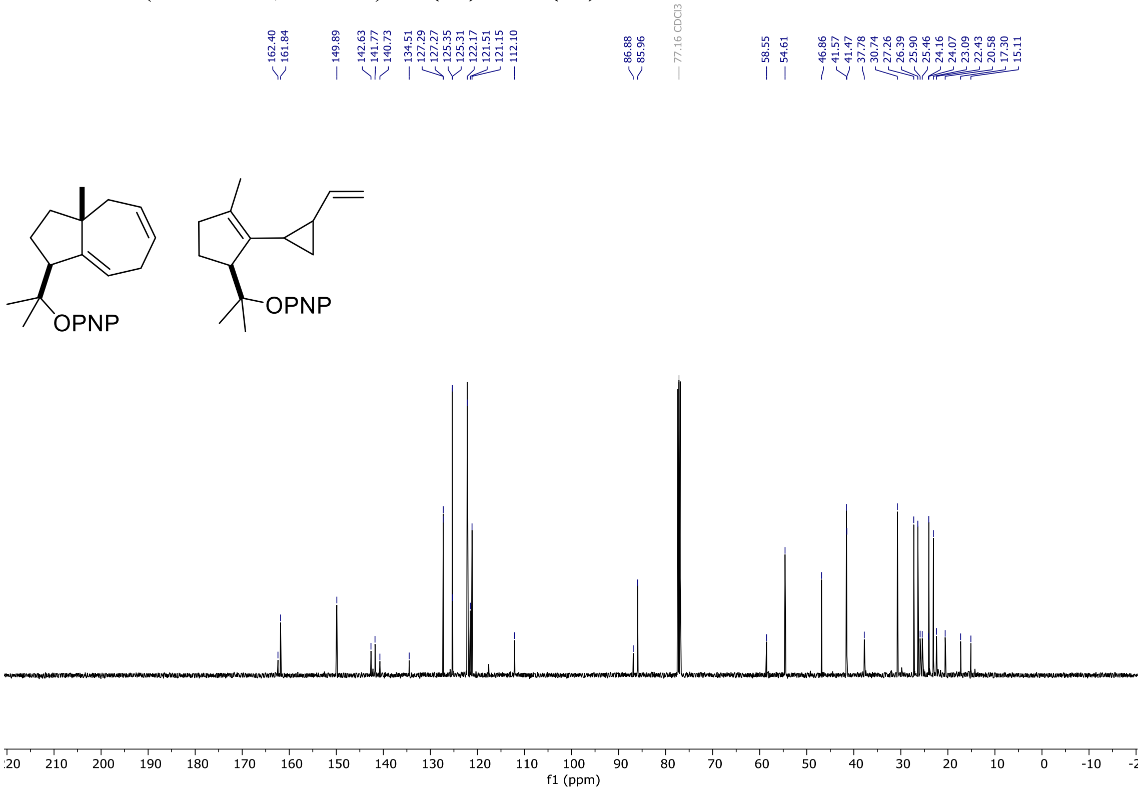

^{1}H NMR (400 MHz, $CDCl_3$) of **(7f)+ (8f)**

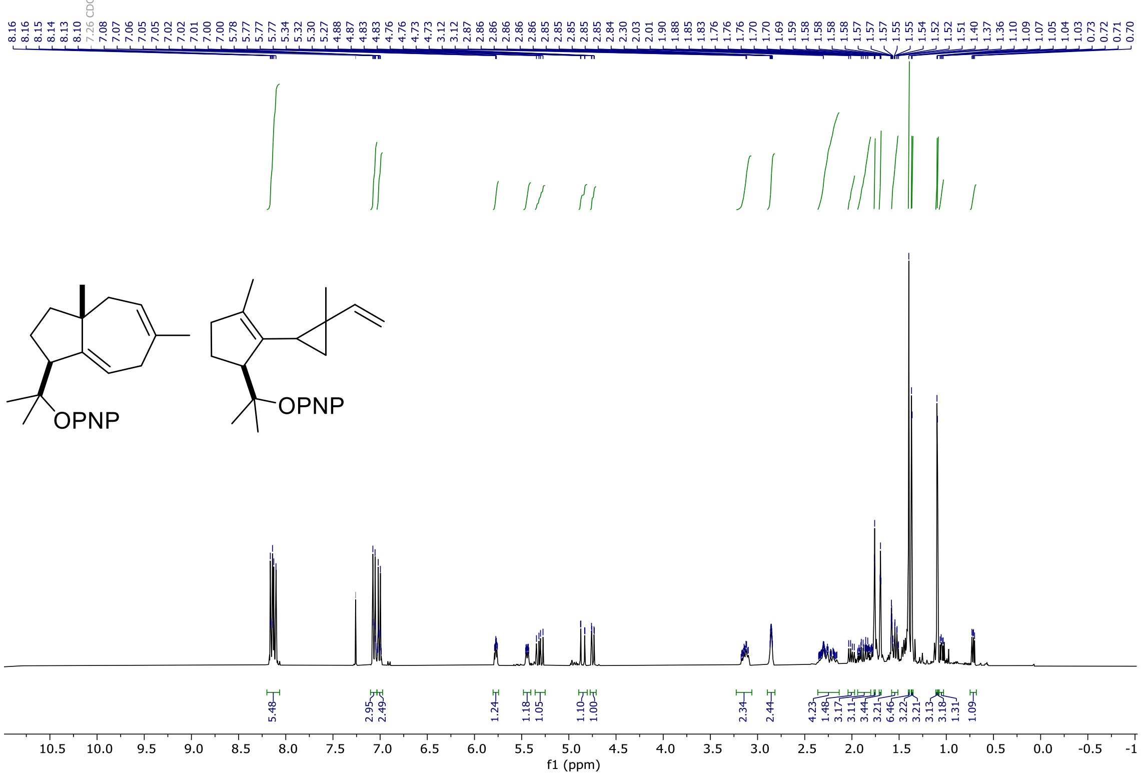

^{13}C NMR (101 MHz, $CDCl_3$) of **(7f) + (8f)**

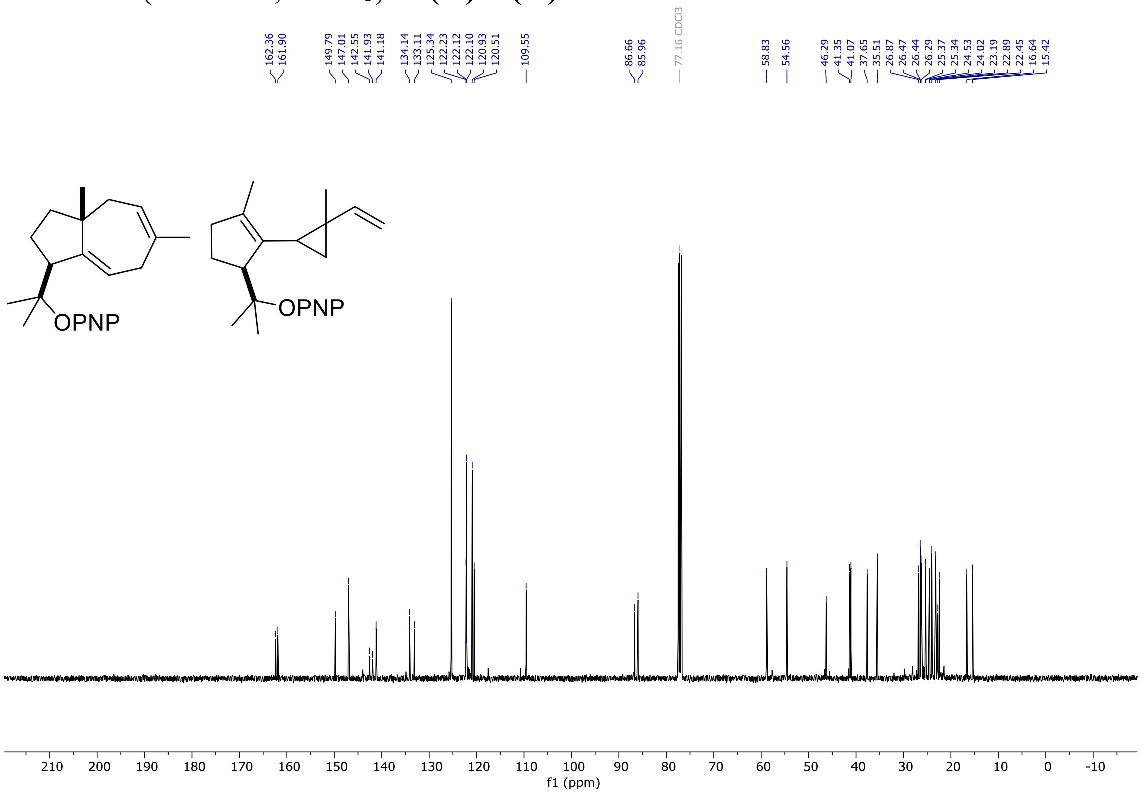

^{1}H NMR (500 MHz, $CDCl_3$) of **(7g) and (8g)**

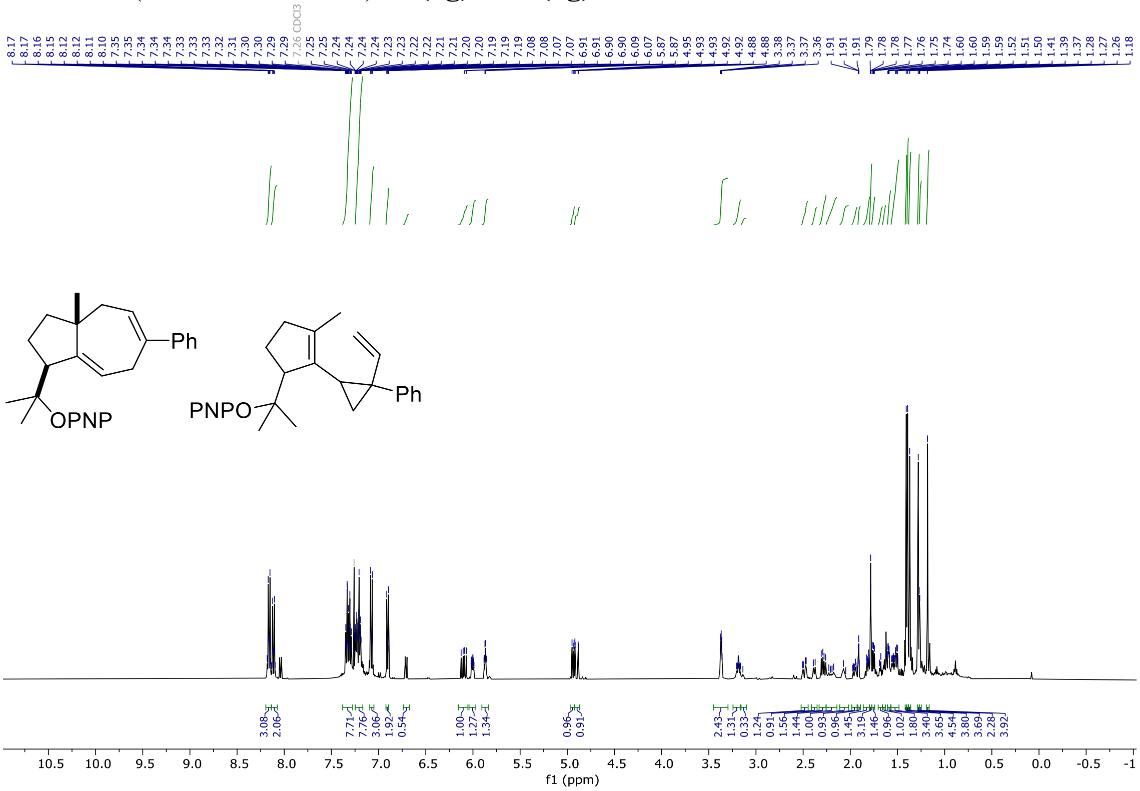

^{13}C NMR (126 MHz, $CDCl_3$) of **(7g) and (8g)**

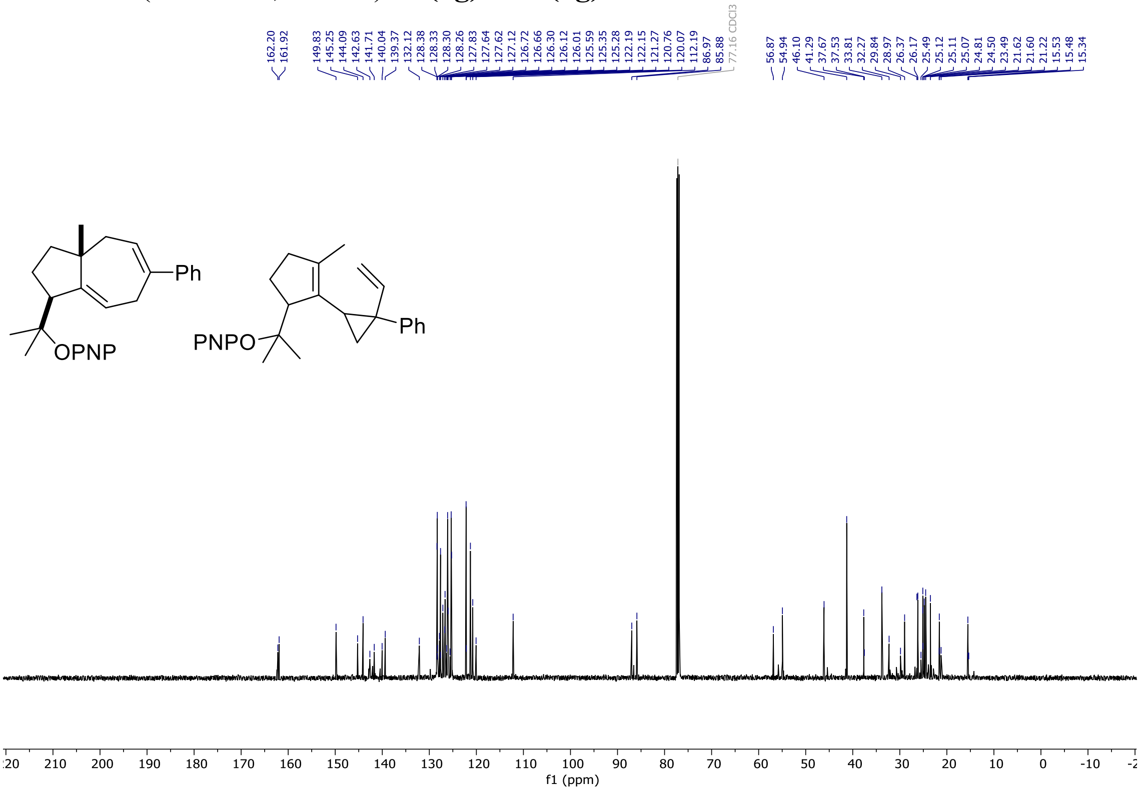

^{1}H NMR (500 MHz, CD_2Cl_2) of **(7h')**

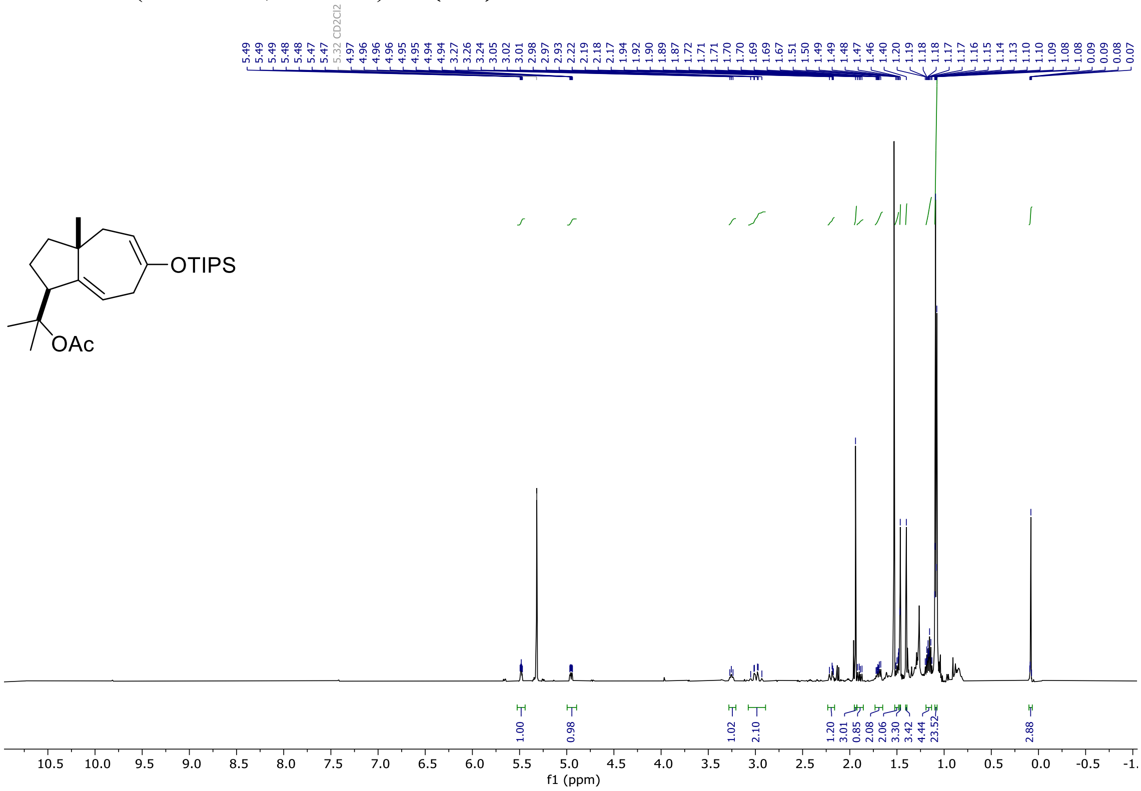

^{13}C NMR (126 MHz, $CDCl_3$) of **(7h')**

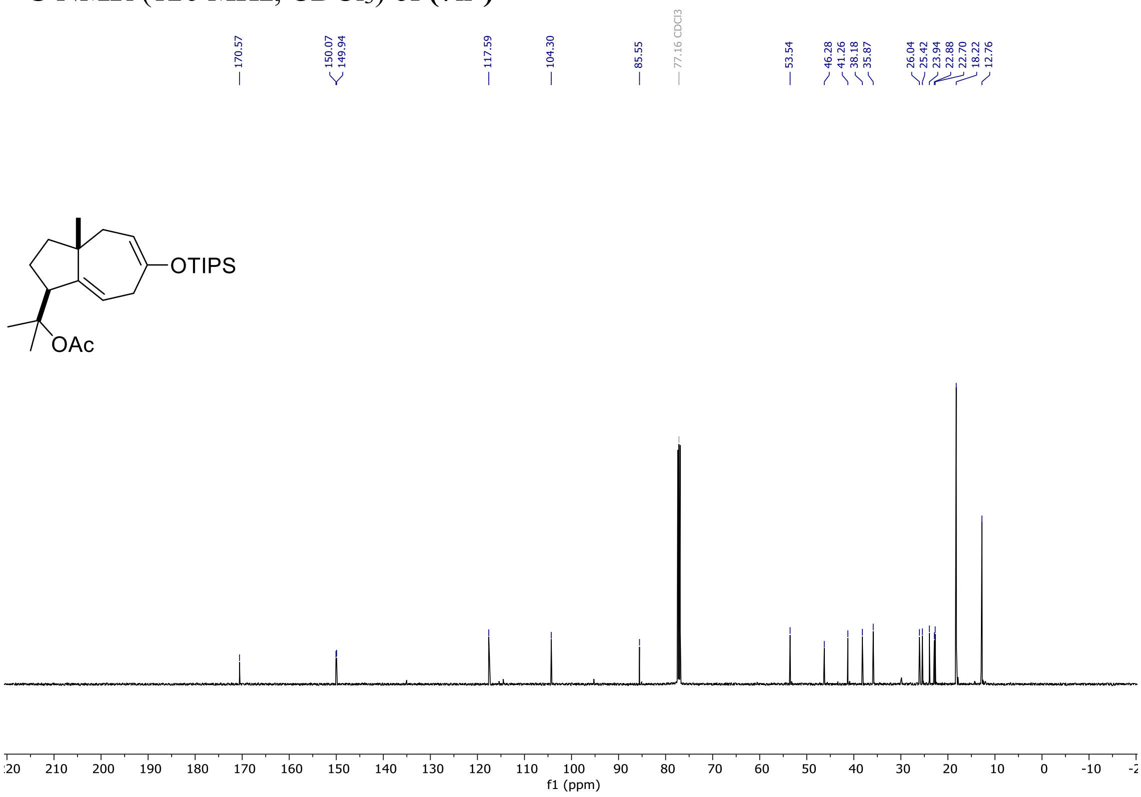

^{1}H NMR (400 MHz, CD_2Cl_2) **(7h)**

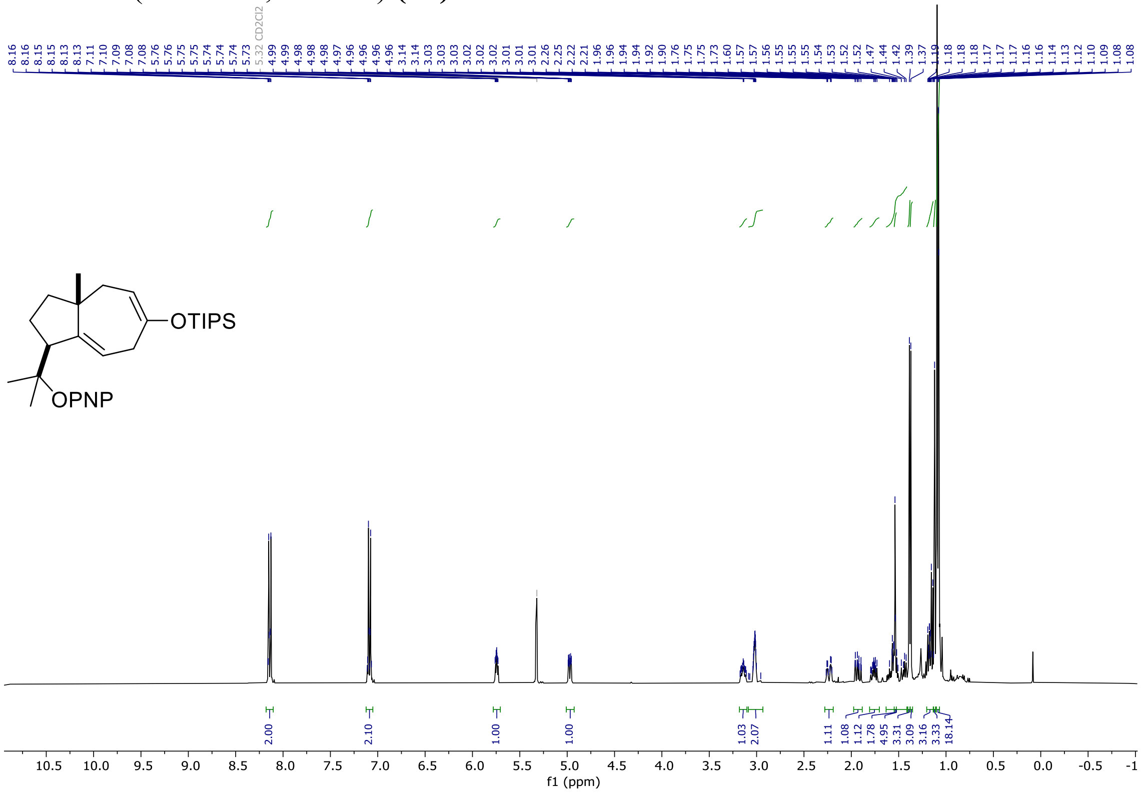

^{13}C NMR (101 MHz, CD_2Cl_2) of **(7h)**

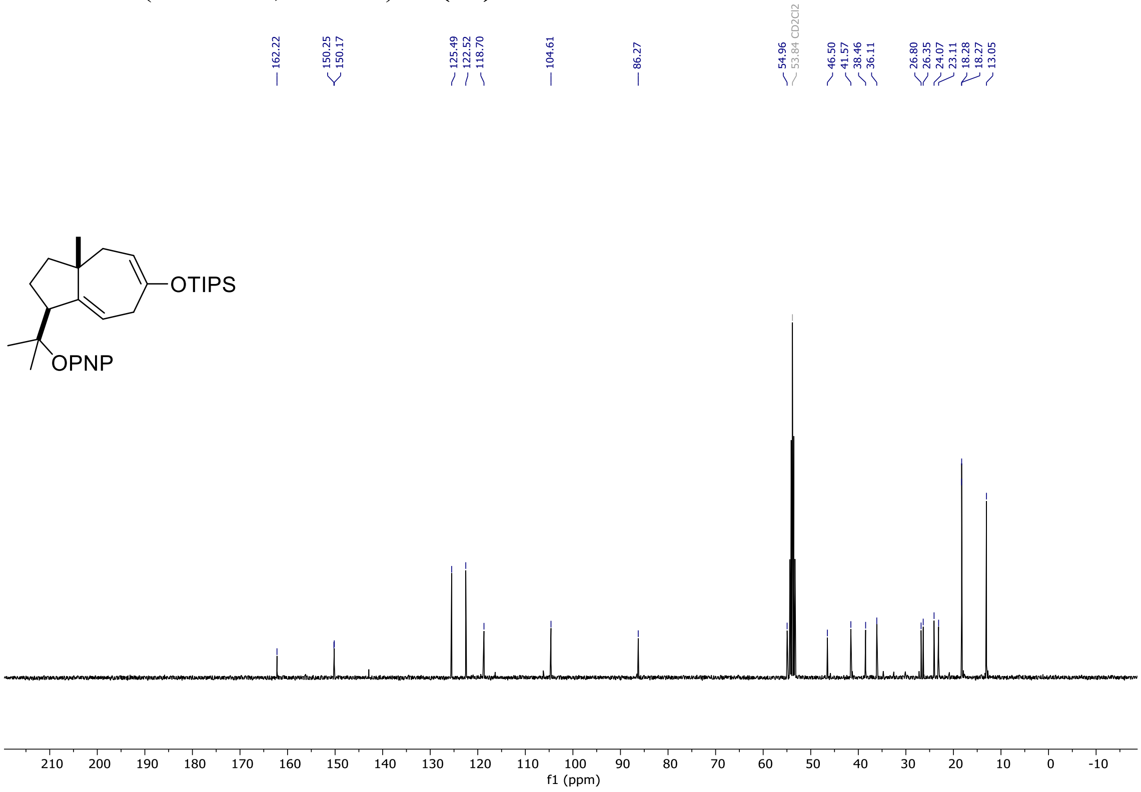

^{1}H NMR (500 MHz, $CDCl_3$) **(9)**

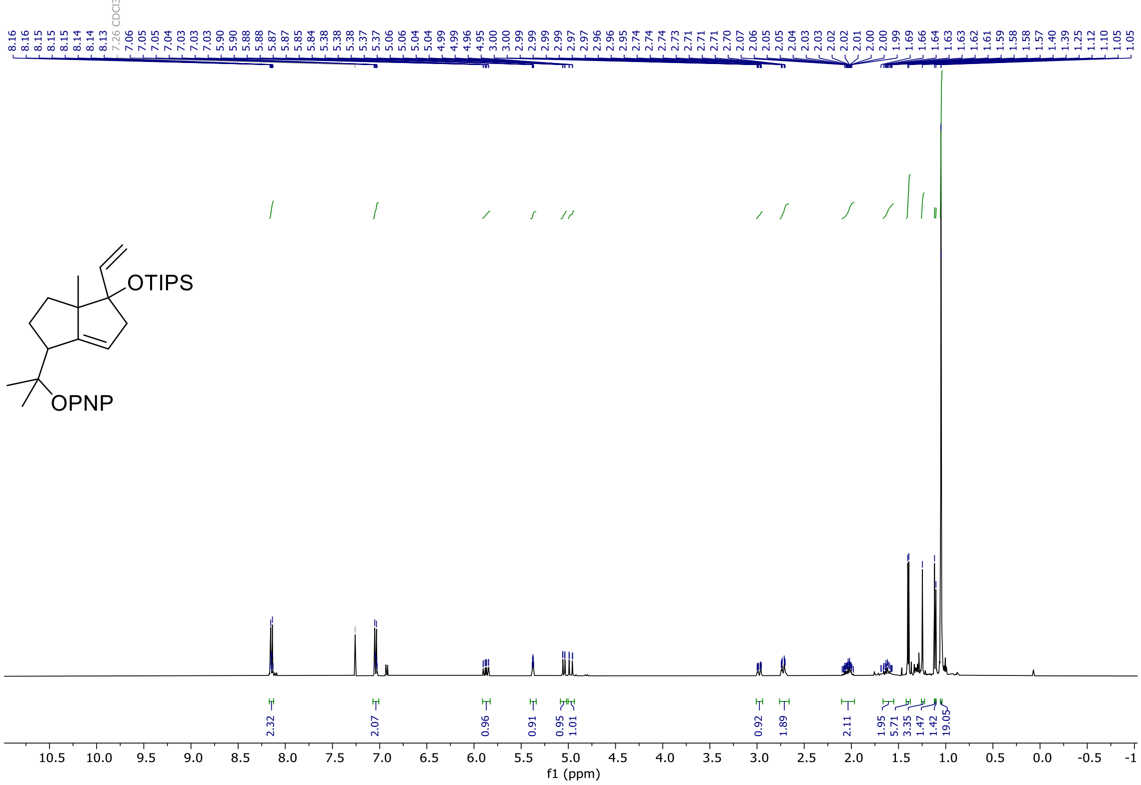

^{13}C NMR (126 MHz, $CDCl_3$) of **(9)**

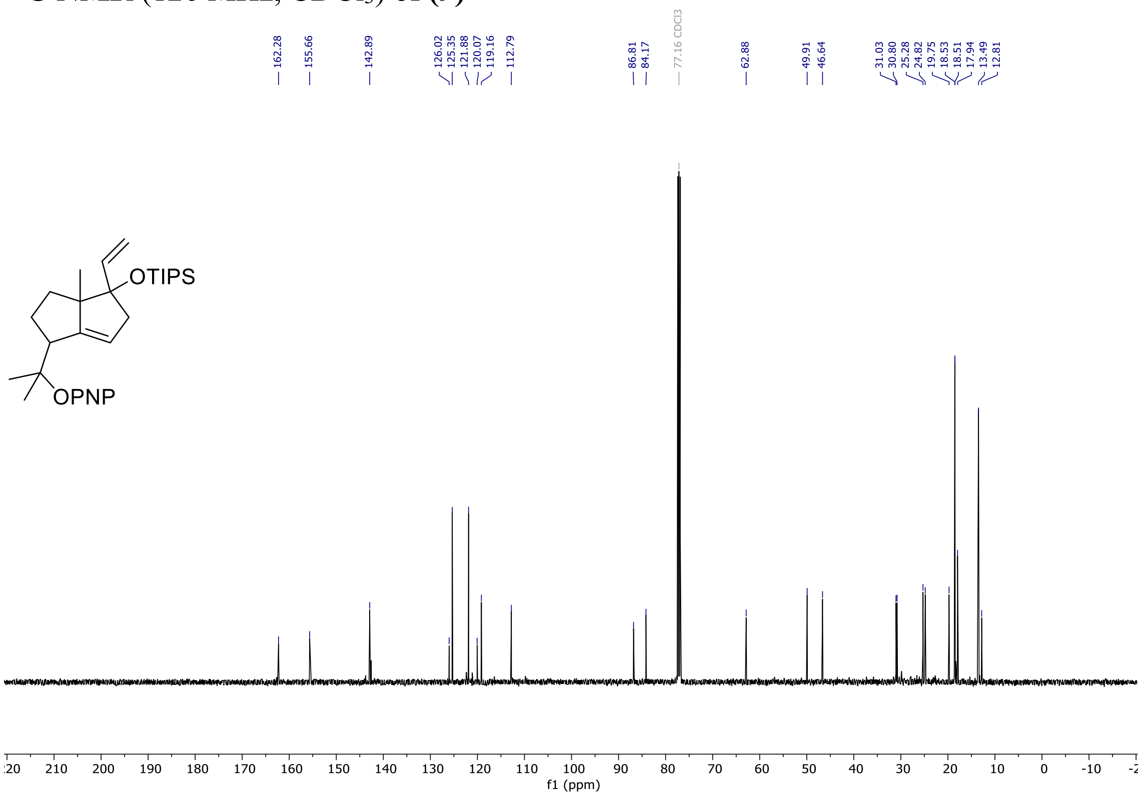

^{1}H NMR (400 MHz, $CDCl_3$) of (7i) + (8j)

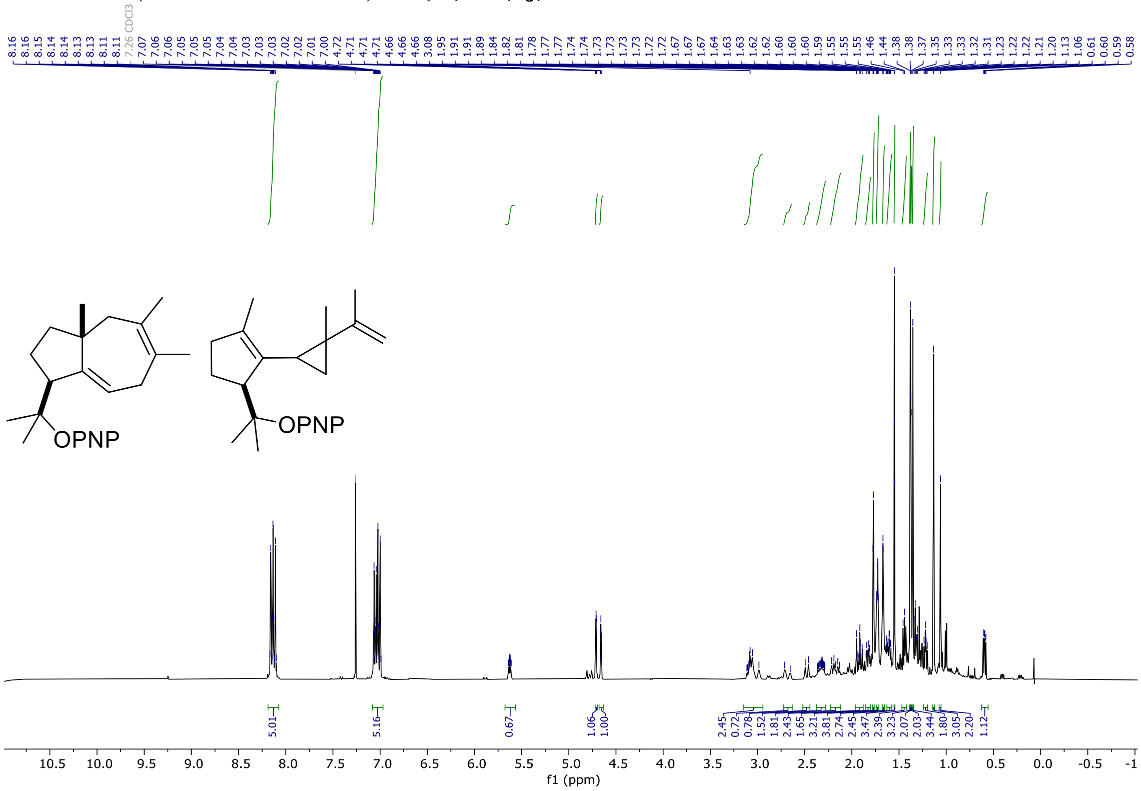

^{13}C NMR (101 MHz, $CDCl_3$) of (7i) + (8j)

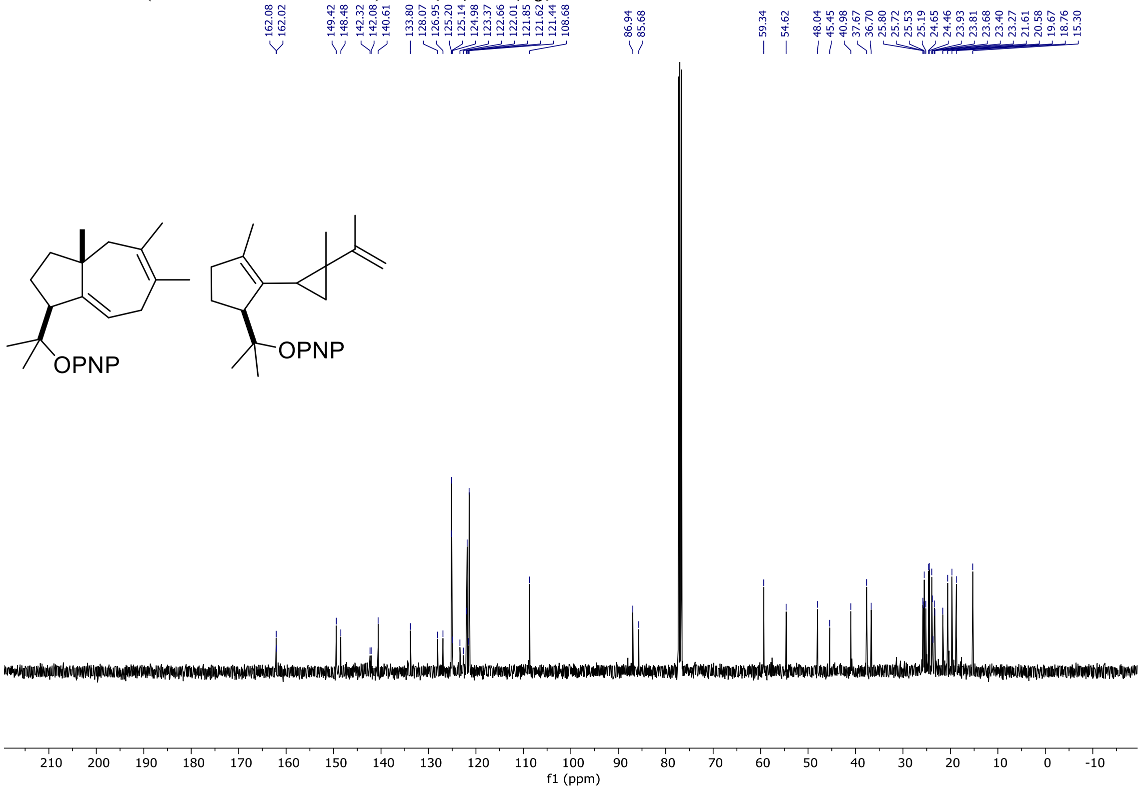

^{1}H NMR (500 MHz, $CDCl_3$) of **(7j)**

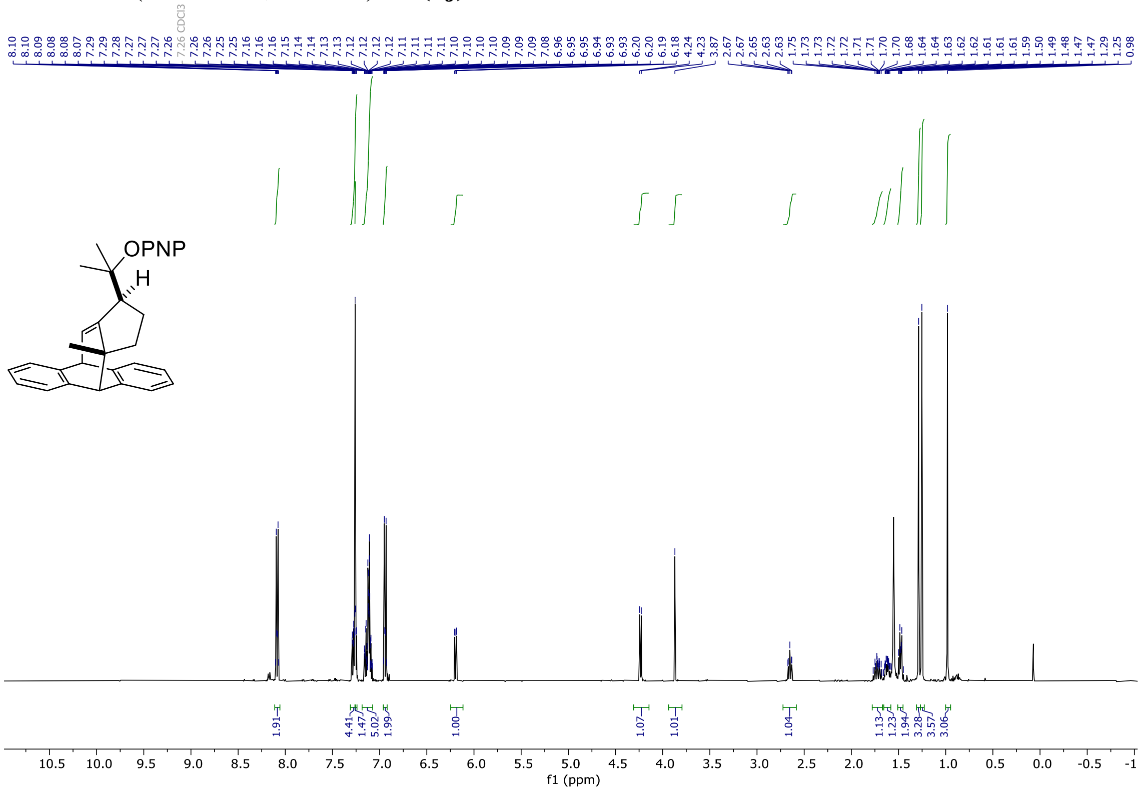

^{13}C NMR (126 MHz, $CDCl_3$) of **(7j)**

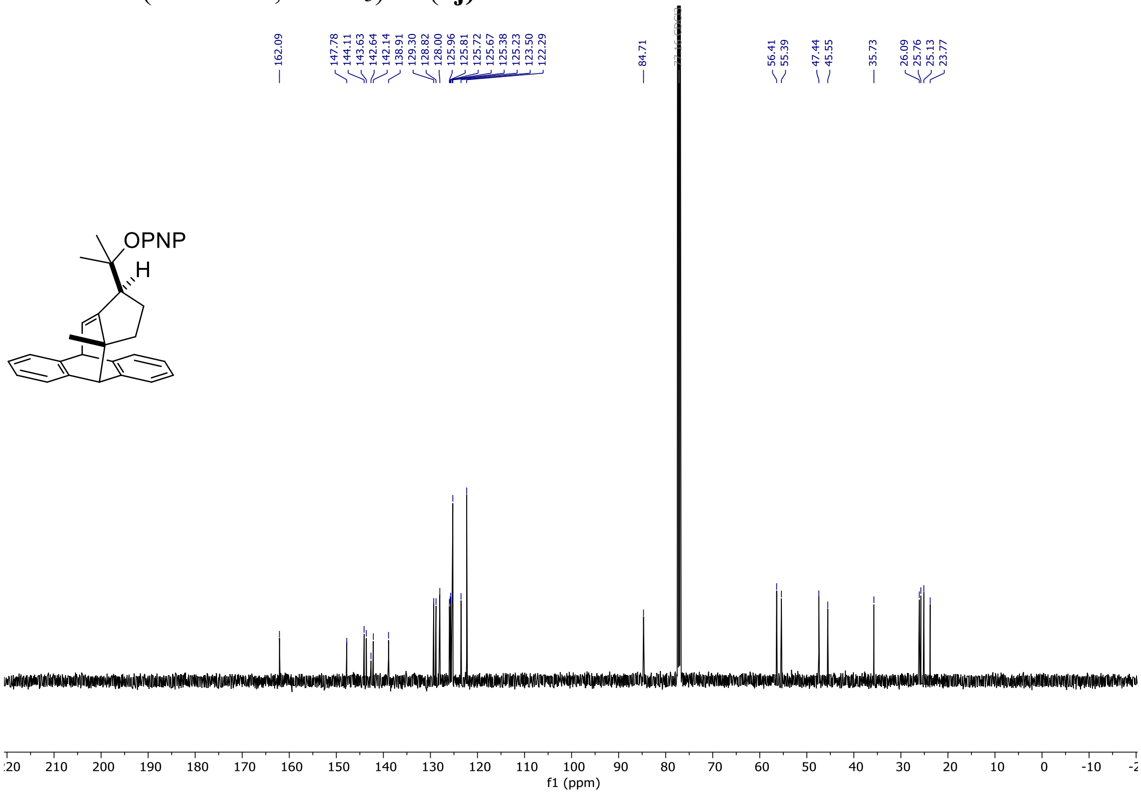

^{1}H NMR (400 MHz, CDCl$_3$) of **(7fa)**

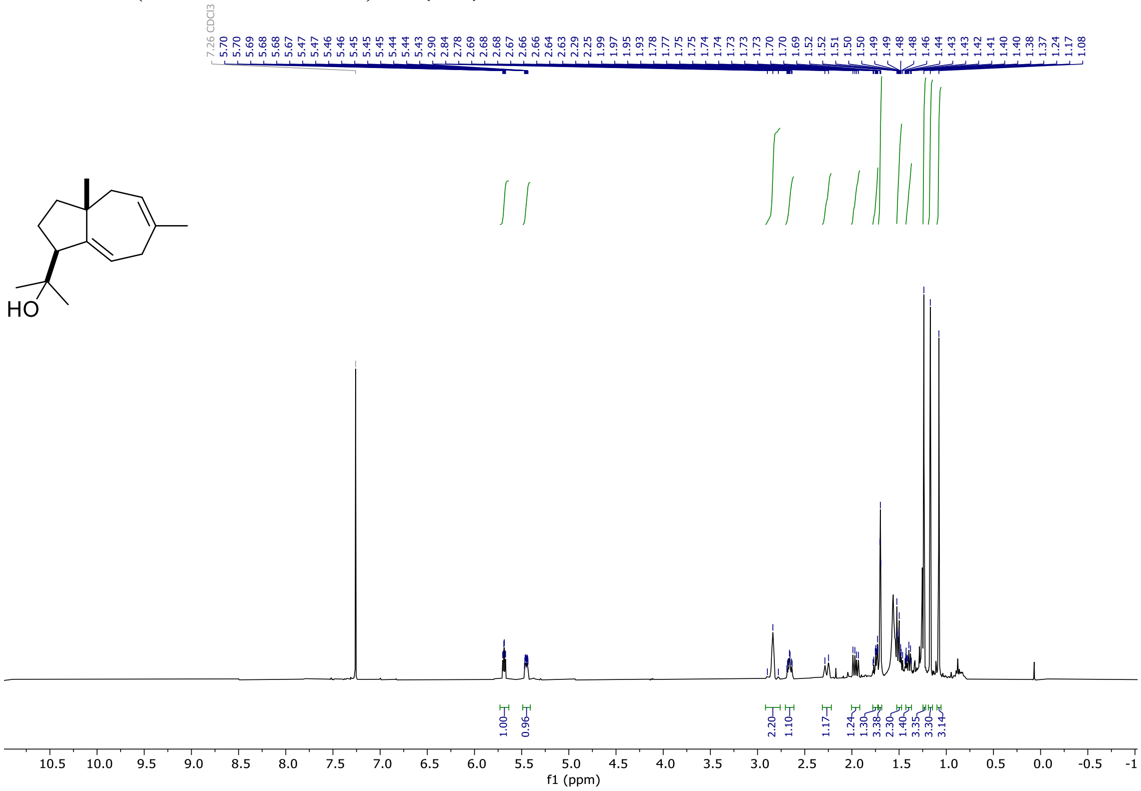

^{13}C NMR (101 MHz, CDCl$_3$) of **(7fa)**

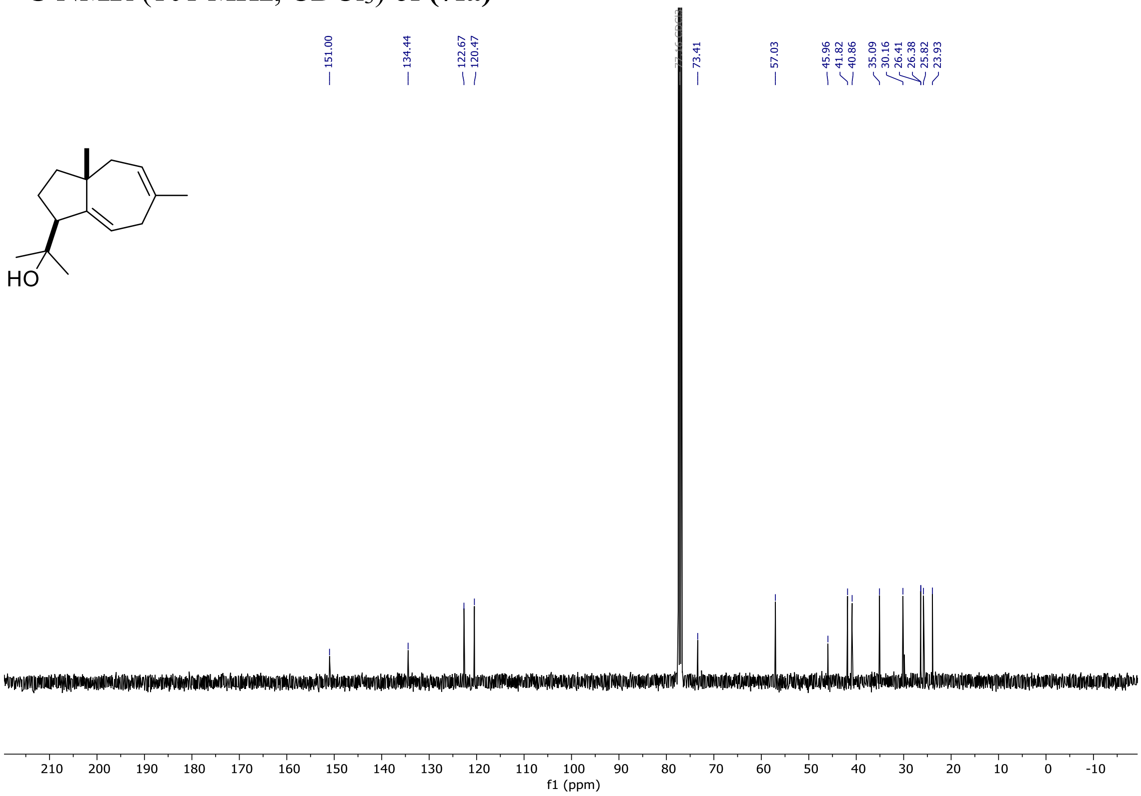

13. DFT Energies and Coordinates

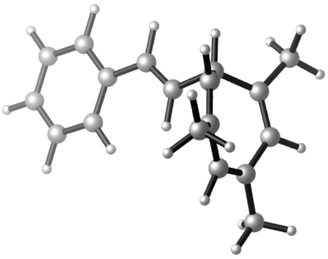

(1a)

E (opt) = -697.981886190 Hartrees
G (opt) = -697.701940 Hartrees
E (SP) = -698.147888691 Hartrees

C	2.60217500	-0.87081600	-1.12627300
C	2.65862900	-1.39817000	0.12991600
C	2.62870600	-0.68280000	1.39973700
C	2.47058200	0.54974600	-1.42678500
C	2.00918900	0.50348900	1.59949900
C	1.88646400	1.51699300	-0.68210800
C	1.11394900	1.21514300	0.60087600
H	2.86029800	-2.46680400	0.20519900
H	2.89756800	0.85384300	-2.38321800
H	2.11594200	0.98490300	2.56987700
C	1.89754400	2.95825700	-1.11173000
C	2.78321900	-1.76904900	-2.33009200
C	3.37030400	-1.35868200	2.53528600
H	2.95814200	-2.35861900	2.72329600
H	4.43142800	-1.49766600	2.29375400
H	3.30271000	-0.78683000	3.46510500
H	2.92087000	-2.81540800	-2.04295700
H	1.91552300	-1.71007000	-2.99951600
H	3.65473100	-1.46082000	-2.92228300
H	2.37819300	3.58610900	-0.34999400
H	2.42510800	3.10560600	-2.05864000
H	0.87166600	3.33515200	-1.22183900
H	0.82371700	2.17347000	1.04381300
C	-0.15593800	0.44851800	0.30581900
H	-0.01557800	-0.55129000	-0.09642000
C	-1.38788400	0.94268200	0.50439100
H	-1.47902100	1.94892700	0.91605000
C	-2.66775000	0.27040600	0.22632900
C	-3.86802200	0.94137100	0.52788700
C	-2.75672500	-1.01877300	-0.33720800
C	-5.10870700	0.35149800	0.28188400
H	-3.82235000	1.93761200	0.96144900
C	-3.99484300	-1.60799200	-0.58202100
H	-1.85140600	-1.56368700	-0.58729600
C	-5.17876700	-0.92733900	-0.27466500
H	-6.01973400	0.89203000	0.52425400
H	-4.03830400	-2.60291100	-1.01706300
H	-6.14225000	-1.39044400	-0.46801900

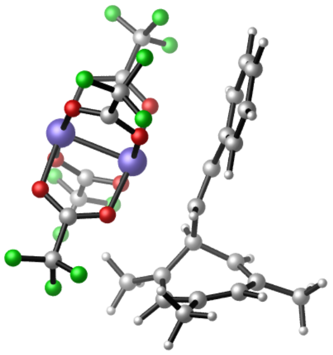

(II) (1a-Rh)

E (opt) = -3021.99506948 Hartrees
G (opt) = -3021.633602 Hartrees
E (SP) = -3022.88167338 Hartrees

Rh	-0.18105600	0.11701600	0.19822600
O	0.51489000	1.62607600	1.42377100
O	-0.96629700	-1.27397700	-1.10117600
O	-1.87621400	0.03568800	1.38623400
C	1.29039200	1.23015300	-2.03678100
C	2.57110700	1.37707200	-2.88847900
F	3.49193400	2.06385900	-2.18246600
F	3.08510000	0.17140600	-3.18284800
F	2.33626500	2.03279400	-4.03022200
O	1.38762100	0.36557400	-1.12490900
O	0.32786600	1.99365100	-2.31381200
Rh	-1.36586100	1.84163800	-1.11751900
O	-0.53912100	3.23345100	0.20011100
O	-3.00126000	1.57256500	0.14071400
O	-2.09595000	0.29386000	-2.30800200
C	0.19688900	2.81204700	1.12956700
C	-1.74810000	-0.87876700	-2.00868900
C	0.86736400	3.90218900	1.99385800
F	1.97807400	4.33013100	1.35740100
F	1.22747900	3.42655600	3.19277000
F	0.05253000	4.94992800	2.17188900
C	-2.39974000	-2.02739100	-2.80843600
F	-2.92414400	-1.59678900	-3.96163400
F	-3.38614900	-2.56485800	-2.06343400
F	-1.50445100	-2.99044400	-3.07582600
C	-2.87792600	0.73192900	1.06902000
C	-4.12892600	0.44650700	1.92781100
F	-4.68240300	-0.71129000	1.51441200
F	-3.79459400	0.31075700	3.22153800
F	-5.04168000	1.41756600	1.81984100
C	4.38035400	-2.14662500	-0.26717200
C	4.29943700	-3.05083300	0.75372100
C	4.13198700	-2.76196900	2.17053600
C	4.29543400	-0.70678900	-0.08885800
C	3.46480700	-1.68643000	2.65684700
C	3.60316300	-0.02121200	0.85073900
C	2.64774800	-0.71981300	1.80904700
H	4.49720200	-4.09414500	0.50839900
H	4.85489800	-0.11924100	-0.81644200
H	3.46166300	-1.50552800	3.72973700
C	3.71919900	1.47518300	0.96126500
C	4.67325100	-2.61112400	-1.67593000
C	4.77960100	-3.75050500	3.11814600
H	4.38722800	-4.76094200	2.94539900
H	5.86378300	-3.80231800	2.96026400
H	4.59681700	-3.49338500	4.16521900
H	4.80571600	-3.69536400	-1.73154500
H	3.85761600	-2.32856500	-2.35332400
H	5.58141500	-2.13459700	-2.06653700
H	3.84629500	1.78334000	2.00577500
H	4.56235900	1.86206800	0.38323500

H	2.80924800	1.96026200	0.60025500
H	2.20906200	0.02519300	2.47781600
C	1.50799300	-1.48684600	1.15802700
H	1.74278700	-2.00921000	0.23673000
C	0.34730500	-1.78601900	1.82550700
H	0.16518100	-1.27716300	2.77251800
C	-0.63705400	-2.81213700	1.45945300
C	-1.88080600	-2.82566400	2.11970100
C	-0.40204400	-3.75821400	0.44255200
C	-2.87417100	-3.72917500	1.74801300
H	-2.07543600	-2.09649800	2.89923300
C	-1.39347100	-4.66367300	0.07692700
H	0.55539300	-3.77954400	-0.06766400
C	-2.63639800	-4.64612600	0.72016400
H	-3.83518700	-3.71125700	2.25370800
H	-1.20162900	-5.38085100	-0.71584100
H	-3.41145900	-5.34793600	0.42570300

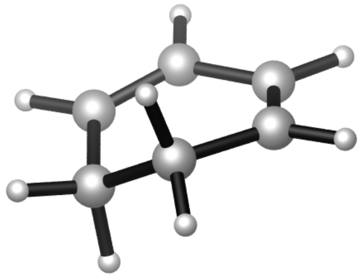

(2a)

E (opt) = -233.441394064 Hartrees
G (opt) = -233.346671 Hartrees
E (SP) = -233.499816974 Hartrees

C	0.30060100	0.70843200	-1.19464000
C	0.04822500	1.42641200	0.11198100
C	-0.04822500	0.73272600	1.26016600
C	0.04822500	-0.73272600	1.26016600
C	-0.04822500	-1.42641200	0.11198100
C	-0.30060100	-0.70843200	-1.19464000
H	-0.17878700	1.24462700	2.21092300
H	0.01252400	2.51353600	0.11287500
H	-1.39097000	-0.64689200	-1.34981800
H	0.17878700	-1.24462700	2.21092300
H	-0.01252400	-2.51353600	0.11287500
H	0.09304300	-1.28429000	-2.03902000
H	1.39097000	0.64689200	-1.34981800
H	-0.09304300	1.28429000	-2.03902000

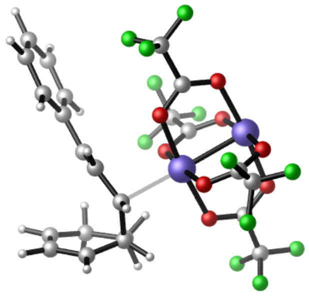

(Va)

E (opt) = -2905.20533312 Hartrees
G (opt) = -2904.903381 Hartrees
E (SP) = -2906.06971495 Hartrees

C	0.51899000	-1.00316500	-2.32005200
C	1.83627900	-0.44494400	-2.10308500
H	1.99172600	0.50719000	-2.60469300
C	2.80721500	-0.89136500	-1.26437700
H	2.60954500	-1.75832000	-0.65243000
C	4.03249100	-0.17111900	-0.92105200
C	4.71464800	-0.53698700	0.25837200
C	4.54221200	0.90738700	-1.67392200
C	5.84795600	0.15733600	0.67893700
H	4.32913300	-1.35655600	0.85869300
C	5.67405400	1.59978400	-1.25120300
H	4.05199300	1.20142200	-2.59716200
C	6.33310200	1.23102500	-0.07269600
H	6.34975000	-0.13599200	1.59702800
H	6.04782700	2.42946200	-1.84511800
H	7.21531100	1.77457000	0.25310800
Rh	-0.52680800	-0.06094600	-0.45008900
O	-1.94214700	0.91662100	-1.60505700
O	0.80245600	-0.96022500	0.85659500
O	0.66693900	1.64455300	-0.52768200
C	-2.64375600	-1.62036200	0.75788900
C	-3.53520100	-2.87789400	0.84241000
F	-4.26193800	-2.99865500	-0.28455800
F	-2.76114900	-3.97415400	0.95888100
F	-4.37198700	-2.83630000	1.88476200
O	-1.82973400	-1.65353300	-0.20815800
O	-2.81891600	-0.73378800	1.63171600
Rh	-1.58486000	0.95258300	1.56841900
O	-2.89371100	1.88287400	0.23275000
O	-0.23202700	2.52563200	1.37338900
O	-0.15181500	-0.05630500	2.71737700
C	-2.76559000	1.65869600	-0.99845600
C	0.69792200	-0.74294700	2.09346400
C	-3.72510500	2.41964900	-1.93956300
F	-4.28533400	1.57470000	-2.82277000
F	-3.02912600	3.34519500	-2.62929000
F	-4.70170100	3.03972800	-1.26736300
C	1.82601300	-1.37034600	2.94031500
F	1.39677000	-1.71148300	4.16233500
F	2.82405300	-0.47392800	3.07380200
F	2.32978100	-2.46587800	2.34351100
C	0.56757700	2.48988200	0.40187800
C	1.61647200	3.61943300	0.31231300
F	1.46644500	4.52779600	1.28356400
F	2.85136500	3.09092000	0.40768400
F	1.52068600	4.24606300	-0.87520700
H	-0.07055200	-0.43467600	-3.03970000
C	2.00877600	-4.04659400	-1.42155700
C	2.67029600	-3.71297100	-2.71642100
C	2.01748900	-3.16171500	-3.78614000
C	0.69937100	-2.68456500	-3.64429200
C	0.09535000	-2.50114000	-2.31825800
C	0.57832500	-3.48052400	-1.23228500

H	2.52471200	-3.05775900	-4.74079200
H	3.70545000	-4.02707900	-2.83130400
H	-0.13084600	-4.31305300	-1.24600200
H	0.15459400	-2.33876200	-4.51768300
H	-0.99355800	-2.51318800	-2.35788800
H	0.48640600	-3.01345400	-0.25217300
H	2.67272500	-3.76342800	-0.59677000
H	1.98238700	-5.14598800	-1.37086800

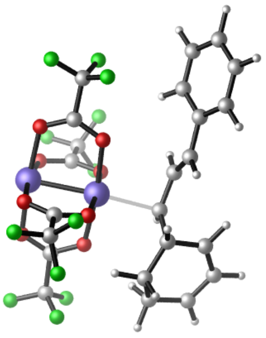

(Vb)

E (opt) = -2905.21192426 Hartrees
G (opt) = -2904.906928 Hartrees
E (SP) = -2906.07427445 Hartrees

C	-0.28687200	1.81157100	-1.66623600
C	-1.72163700	1.55856200	-1.67238200
H	-2.00445300	0.65494800	-2.20804800
C	-2.68506700	2.26310300	-1.02935400
H	-2.42961400	3.20555500	-0.54699600
C	-4.08883900	1.86603300	-0.91320300
C	-5.05762700	2.83110100	-0.57098600
C	-4.51737400	0.53286700	-1.08596600
C	-6.40206700	2.48670700	-0.43448200
H	-4.74629700	3.86167200	-0.41663100
C	-5.85924400	0.18951100	-0.94432900
H	-3.78704100	-0.24413400	-1.28141200
C	-6.81159000	1.16393300	-0.62484500
H	-7.13011800	3.25066300	-0.17497200
H	-6.16068600	-0.84775800	-1.06296300
H	-7.85698800	0.89155000	-0.51032900
Rh	0.26543100	0.09430500	-0.18968200
O	-0.06348400	-1.29925300	-1.68864200
O	0.63270400	1.35283400	1.40975400
O	-1.65348900	-0.27455100	0.56422500
C	3.10112300	-0.47339000	-0.25043100
C	4.53815300	-0.12104500	-0.69006100
F	4.63479900	-0.14604600	-2.03188000
F	4.82922800	1.13074000	-0.27715700
F	5.44508500	-0.95900500	-0.17976400
O	2.22589000	0.24444400	-0.81200800
O	2.96157200	-1.37977200	0.60696600
Rh	1.01628000	-1.79447800	1.26637100
O	0.68083000	-3.02332800	-0.38966300
O	-0.97146200	-2.05368700	1.81982300
O	1.28690200	-0.35930100	2.77009800
C	0.22250900	-2.50467500	-1.43952100
C	1.06963000	0.84417000	2.48121400
C	-0.05612100	-3.45821100	-2.62172100
F	0.59408300	-3.03160000	-3.71990400
F	-1.37561100	-3.46595500	-2.89349400
F	0.32963900	-4.71202100	-2.36050100
C	1.41320400	1.89751500	3.55572700
F	1.76465900	1.33903600	4.71888200
F	0.36385000	2.71015200	3.77011900
F	2.44240400	2.65058300	3.11552100
C	-1.81865900	-1.25463100	1.33865500
C	-3.29624100	-1.52689300	1.69921000
F	-3.41334400	-2.35695400	2.74376400
F	-3.93680600	-0.38450200	1.98905200
F	-3.90766600	-2.09666700	0.63779700
H	0.26739200	1.36383500	-2.48934100
C	1.80339100	3.25249700	-0.82799900
C	0.30055500	3.13655600	-1.12241600
C	-0.07560200	3.79053200	-2.38118100
C	0.85771600	4.05779000	-3.41510400
C	2.17352200	3.71797700	-3.27689700
C	2.68973300	3.03192100	-2.06218400
H	-1.12064400	4.03110400	-2.54518700
H	-0.29078700	3.45766500	-0.26353100
H	3.71975300	3.34494100	-1.85766300
H	0.49700200	4.50687100	-4.33543000
H	2.86086000	3.89826100	-4.10015900
H	2.76114000	1.95842100	-2.29588000
H	2.09782800	2.57879500	-0.02612700
H	1.96147100	4.27543900	-0.46572700

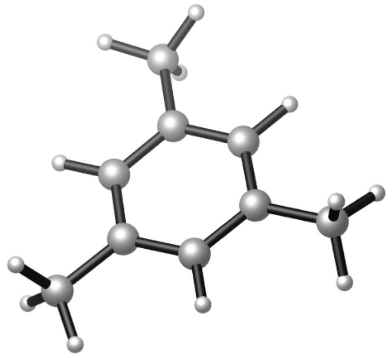

(Mes)

E (opt) = -350.235781599 Hartrees
G (opt) = -350.087583 Hartrees
E (SP) = -350.317575535 Hartrees

C	-1.85283300	1.05591200	0.01646700
C	-0.44967500	1.06922900	0.01547400
C	0.26768400	2.26987000	0.00044300
C	-0.44555600	3.47840300	-0.01081600
C	-1.84386400	3.49929200	-0.00623900
C	-2.53401800	2.27750400	0.00675900
H	0.09101500	0.12481400	0.02598100
H	0.10176500	4.41922800	-0.02074800
H	-3.62239100	2.28156300	0.01094500
C	-2.60615300	-0.25357000	0.00044100
C	1.77810300	2.27675500	-0.02301400
C	-2.60028400	4.80685200	0.01716100
H	-1.99852100	5.62620100	-0.38827100
H	-3.52737500	4.74414400	-0.56226300
H	-2.87947800	5.08258200	1.04235600
H	-2.10434500	-1.01192400	0.61073100
H	-3.62752600	-0.13509700	0.37531600
H	-2.67649600	-0.65637400	-1.01829600
H	2.15582400	2.59333500	-1.00348500
H	2.18673200	2.97515100	0.71600400
H	2.18868100	1.28414600	0.18484100

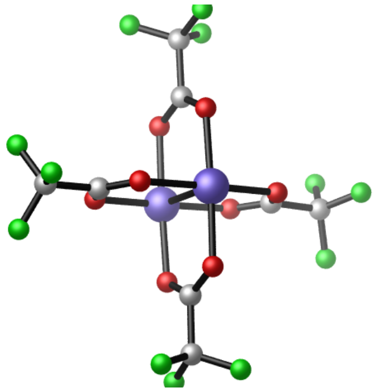

(Rh_2TFA_4)

E (opt) = -2323.96839904 Hartrees
G (opt) = -2323.915912 Hartrees
E (SP) = -2324.70026139 Hartrees

Rh	0.00890000	0.00390900	1.22856500
O	1.42836900	1.51184500	1.14615400
O	-1.41153500	-1.50430600	1.15143300
O	-1.50008600	1.42219800	1.16894500
C	1.90481600	-1.78190100	-0.01636700
C	2.99000600	-2.88161300	-0.00345500
F	3.99156500	-2.53216600	0.81758300
F	2.44820200	-4.02722200	0.44956000
F	3.48910500	-3.09719800	-1.22414000
O	1.51734800	-1.41496000	1.12764500
O	1.50870300	-1.39050400	-1.14749900
Rh	-0.00959500	0.01742200	-1.20371800
O	1.39740400	1.53612900	-1.12852600
O	-1.52982500	1.42252700	-1.10614700
O	-1.41471200	-1.50251900	-1.12361900
C	1.79254900	1.91498200	0.00885100
C	-1.78970100	-1.89737000	0.01544300
C	2.89641900	2.99567800	-0.01978000
F	4.05006300	2.43231900	-0.42393100
F	3.08500300	3.53921700	1.18655500
F	2.57241200	3.96704400	-0.88552200
C	-2.86098500	-3.01012900	-0.01710900
F	-2.38798900	-4.07104400	-0.69173900
F	-3.95725200	-2.55813200	-0.64991500
F	-3.20582700	-3.40165300	1.21275500
C	-1.90908300	1.79853200	0.03743600
C	-3.02512300	2.86663600	0.01847000
F	-4.09102400	2.39531500	-0.64931700
F	-3.40671300	3.20599500	1.25299600
F	-2.57948100	3.96618800	-0.61334400

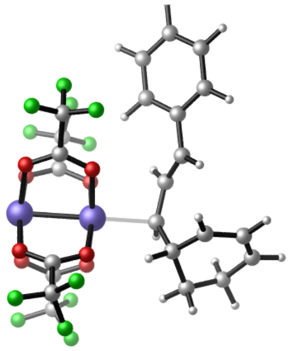

($TS_{Va\text{-}Vb}$)

E (opt) = -2905.18831489 Hartrees
G (opt) = -2904.888390 Hartrees
E (SP) = -2906.05359905 Hartrees

C	-0.31835100	2.10273900	-1.09867100
C	-1.72594100	1.78080000	-1.17479900
H	-1.90605400	0.95776600	-1.86292900
C	-2.80733200	2.20130700	-0.46594400
H	-2.77564600	3.12791600	0.10428900
C	-4.06578400	1.46161300	-0.39640900
C	-5.25796800	2.12018500	-0.03046600
C	-4.12614000	0.07234500	-0.64785600
C	-6.46599200	1.42863200	0.04650100
H	-5.22918700	3.18552200	0.18641700
C	-5.33447500	-0.61457700	-0.57621900
H	-3.21628500	-0.47218300	-0.87263400
C	-6.51235400	0.05882700	-0.23236200
H	-7.37343600	1.95824000	0.32410000
H	-5.35166200	-1.68333500	-0.77223700
H	-7.45299200	-0.48078100	-0.16819400
Rh	0.43800400	0.21765200	-0.17047400
O	-0.61807000	-0.92840900	-1.56586600
O	1.58263700	1.12271800	1.31435000
O	-1.14334500	-0.00662700	1.16750600
C	2.89786500	-0.71521400	-1.36530800
C	4.08094600	-0.58130500	-2.34959200
F	3.62118500	-0.57541800	-3.61652900
F	4.72899500	0.58085900	-2.13813400
F	4.96052600	-1.58210400	-2.22532900
O	2.08714300	0.25318500	-1.43135800
O	2.87803300	-1.74197600	-0.64204200
Rh	1.30526900	-1.98121300	0.72756800
O	0.11311400	-2.91502600	-0.71430500
O	-0.37012800	-1.99666600	1.98338300
O	2.38550200	-0.85699100	2.11947700
C	-0.58622500	-2.18654200	-1.46076100
C	2.27930700	0.39252000	2.07694200
C	-1.63086600	-2.91271700	-2.33561600
F	-1.77256000	-2.31849600	-3.53013600
F	-2.82573300	-2.86271400	-1.69979600
F	-1.31740800	-4.19876300	-2.53335000
C	3.13544100	1.20016300	3.07681800
F	3.74743400	0.41305100	3.96892500
F	2.36890700	2.08336900	3.74306900
F	4.07733200	1.89236800	2.40384500
C	-1.19569200	-1.05297300	1.87032100
C	-2.51653600	-1.23077500	2.65130800
F	-2.33084800	-1.91590200	3.78986000
F	-3.08090700	-0.05236400	2.95319700
F	-3.38090100	-1.92462100	1.87923300
H	0.17142200	1.96883000	-2.06650500
C	1.32694100	4.02200500	-1.29177700
C	0.32447100	3.33146800	-0.33826100
C	-0.70460200	4.30149200	0.08043000
C	-1.15508000	5.33367100	-0.74483200
C	-0.55673300	5.53854000	-1.97170900
C	0.59219600	4.72973600	-2.43942500

H	-1.14807300	4.19115900	1.06692100
H	0.84979800	2.97483200	0.54711200
H	1.26233300	5.34457700	-3.04949400
H	-1.97351800	5.96833100	-0.42103400
H	-0.95063700	6.30614400	-2.63426600
H	0.16546100	3.98595600	-3.14020100
H	2.02918500	3.28030100	-1.68194800
H	1.90613700	4.76025200	-0.72534400

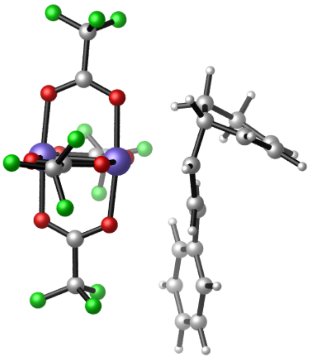

(TS$_{Va\text{-}VIa}$)

E (opt) = -2905.20487770 Hartrees
G (opt) = -2904.902311 Hartrees
E (SP) = -2906.06824292 Hartrees

C	0.46794600	1.15843300	2.41786200
C	1.78335500	0.60000400	2.16203200
H	1.94661200	-0.34301100	2.67873500
C	2.73688900	1.02512600	1.30036400
H	2.53776300	1.87956300	0.67062800
C	3.94658400	0.27255700	0.96312200
C	4.53424800	0.47603400	-0.30224400
C	4.52180000	-0.69265000	1.81439400
C	5.63480900	-0.27562500	-0.71220800
H	4.09921900	1.20989000	-0.97529300
C	5.62206600	-1.44168800	1.40303700
H	4.10991400	-0.84815000	2.80735900
C	6.18320100	-1.24100600	0.13692300
H	6.06187600	-0.11115700	-1.69785400
H	6.04877000	-2.18142200	2.07515100
H	7.04089700	-1.82728700	-0.18014100
Rh	-0.51576800	0.04721800	0.45186200
O	-1.82370400	-1.06967200	1.59907700
O	0.72022000	1.08914400	-0.83730300
O	0.83024000	-1.54171800	0.48342200
C	-2.77150500	1.42374500	-0.70580600
C	-3.79796200	2.57531100	-0.75477700
F	-4.60133100	2.50650700	0.32359200
F	-3.15889500	3.75897500	-0.72615600
F	-4.55595300	2.52638500	-1.85493000
O	-1.95435700	1.51927400	0.25244500
O	-2.86400400	0.53462400	-1.58975400
Rh	-1.47911900	-1.02227200	-1.56333700
O	-2.70250900	-2.08321700	-0.24656800
O	-0.00181100	-2.48103100	-1.42021000
O	-0.14975400	0.11696800	-2.70317900
C	-2.58816900	-1.86875300	0.98806600
C	0.63489400	0.87648200	-2.07535800
C	-3.48516600	-2.70843000	1.92434100
F	-4.14044800	-1.90590500	2.78115000
F	-2.71591400	-3.54967500	2.64179400
F	-4.38298400	-3.42983700	1.24474600
C	1.67632300	1.62141400	-2.93815200
F	1.11945700	2.10285500	-4.05832000
F	2.65772000	0.76542500	-3.28404400
F	2.23166000	2.64208500	-2.26334200
C	0.80386200	-2.38142000	-0.45547200
C	1.96789800	-3.39588500	-0.40608600
F	1.80092400	-4.39650300	-1.27940000

F	3.11874500	-2.75980100	-0.69900600
F	2.08101300	-3.91876800	0.82667800
H	-0.14562500	0.54358900	3.07300700
C	1.75265200	4.18343900	1.32596300
C	2.41864900	3.96625200	2.64972100
C	1.83717800	3.33939800	3.70527800
C	0.54901400	2.73344200	3.58610400
C	-0.09023900	2.59100300	2.27854100
C	0.32940000	3.59051300	1.19352500
H	2.36022800	3.26893800	4.65469400
H	3.40922400	4.39971000	2.77060400
H	-0.39611300	4.40684200	1.26827700
H	0.02208500	2.41073800	4.47714100
H	-1.17547300	2.52689400	2.32852200
H	0.19442300	3.14571100	0.20786500
H	2.41265700	3.81148900	0.53293700
H	1.71660000	5.26823200	1.15146300

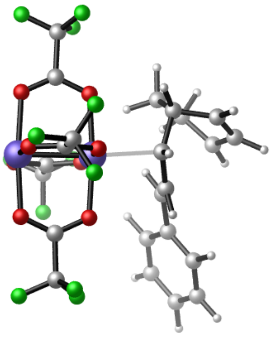

(TS$_{Va\text{-}VII}$)

E (opt) = -2905.20402067 Hartrees
G (opt) = -2904.900089 Hartrees
E (SP) = -2906.06787304 Hartrees

C	0.71864600	-0.95187400	-2.20294000
C	1.94533900	-0.29156400	-1.92551900
H	2.09638100	0.65708700	-2.43819400
C	2.96380400	-0.75060300	-1.10603900
H	2.70955100	-1.45346900	-0.32900000
C	4.22767900	-0.04135600	-0.89644000
C	4.88707000	-0.17241500	0.34268800
C	4.81822700	0.76782900	-1.88727200
C	6.08374500	0.49872600	0.58905400
H	4.43736200	-0.78594400	1.11948800
C	6.01365800	1.43883900	-1.63739600
H	4.34482800	0.85412300	-2.86131700
C	6.65203200	1.30832600	-0.39924100
H	6.57245700	0.39283400	1.55372200
H	6.45547800	2.05784500	-2.41352300
H	7.58618800	1.82919300	-0.20924300
Rh	-0.46275600	-0.03828200	-0.40038100
O	-1.91381600	0.67858900	-1.69315800
O	0.87703900	-0.69077800	1.03190900
O	0.44864100	1.83345400	-0.42460200
C	-2.36613700	-1.92845100	0.68378900
C	-2.99789000	-3.33501300	0.75439600
F	-3.55478200	-3.65178800	-0.42835100
F	-2.03651200	-4.24187000	1.01940900
F	-3.93564000	-3.41951300	1.70379500
O	-1.50965400	-1.81472700	-0.23698500
O	-2.74722500	-1.08326800	1.53148000
Rh	-1.84724000	0.80346100	1.49344700
O	-3.20508400	1.42893900	0.03326600
O	-0.82226800	2.60343300	1.30664000
O	-0.34782400	0.10640100	2.78025000
C	-2.92803700	1.22755800	-1.17674800
C	0.64573400	-0.45270700	2.24862200
C	-3.96393400	1.71249600	-2.21512700
F	-4.38991400	0.67126100	-2.95436800

F	-3.39512700	2.60865000	-3.04278900
F	-5.02547900	2.28379100	-1.63580800
C	1.79627300	-0.86199900	3.19387500
F	1.34724900	-1.17668200	4.41476900
F	2.65651900	0.16982500	3.30652600
F	2.47414400	-1.91492700	2.70184500
C	0.05939400	2.69330000	0.41253600
C	0.77384800	4.05627600	0.27801900
F	0.46813100	4.88473100	1.28323400
F	2.10644200	3.88387700	0.25636800
F	0.40716200	4.63887600	-0.88091700
H	0.11453800	-0.46165400	-2.96673200
C	2.56660900	-3.67042000	-1.11045300
C	3.29553500	-2.97327000	-2.22209400
C	2.66161800	-2.82272600	-3.46257200
C	1.28727400	-2.71303200	-3.49870400
C	0.53271400	-2.49032500	-2.22987100
C	1.05451300	-3.32615200	-1.03893400
H	3.25228000	-2.68484800	-4.36460500
H	4.37943500	-2.92893000	-2.16664500
H	0.47672100	-4.25390100	-1.01340000
H	0.76722600	-2.57966900	-4.44331400
H	-0.53293900	-2.67961100	-2.36002200
H	0.83423700	-2.80210200	-0.10897800
H	3.06456200	-3.50102400	-0.15165200
H	2.68701200	-4.74406600	-1.32012300

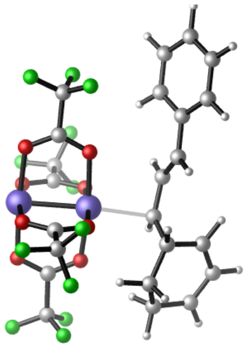

(TS$_{Vb\text{-}VIb}$)

E (opt) = -2905.21188828 Hartrees
G (opt) = -2904.908654 Hartrees
E (SP) = -2906.07405476 Hartrees

C	-0.23176700	1.86852900	-1.69383700
C	-1.66782500	1.61863400	-1.67915800
H	-1.96129200	0.72547200	-2.22600300
C	-2.61683600	2.32077300	-1.01403200
H	-2.34482800	3.25036900	-0.51588900
C	-4.02284200	1.93492500	-0.88753100
C	-4.97405700	2.89930800	-0.49756700
C	-4.47090700	0.61350000	-1.09685900
C	-6.32014500	2.56639900	-0.34875400
H	-4.64760500	3.92051500	-0.31485200
C	-5.81450000	0.28176200	-0.94453400
H	-3.75414700	-0.16597300	-1.33005300
C	-6.74928900	1.25584600	-0.57593700
H	-7.03394200	3.32981500	-0.05091000
H	-6.13091900	-0.74711300	-1.09343800
H	-7.79587700	0.99202300	-0.45265000
Rh	0.27505400	0.09596700	-0.19118100
O	-0.12972400	-1.28341000	-1.68353800
O	0.70524100	1.34184200	1.40072900
O	-1.64411400	-0.20440800	0.58646900
C	3.08603700	-0.56500400	-0.29751500
C	4.52992800	-0.25357200	-0.74618000
F	4.61531600	-0.26767700	-2.08860700
F	4.86208200	0.98408300	-0.32267200
F	5.41365300	-1.12447700	-0.25082600
O	2.22857400	0.18793000	-0.83979600
O	2.92466000	-1.47598400	0.55152600
Rh	0.98037600	-1.81019300	1.25180200
O	0.54977400	-3.03352000	-0.38568100
O	-0.99943000	-1.99081900	1.84980800
O	1.34233900	-0.38940800	2.74356400
C	0.09248700	-2.50086900	-1.42919300
C	1.14981100	0.82057600	2.46263400
C	-0.28714400	-3.45234200	-2.58472600
F	0.29743100	-3.05186800	-3.72772000
F	-1.62238800	-3.42431700	-2.76465800
F	0.08064000	-4.71454300	-2.33901800
C	1.53312600	1.86151100	3.53593600
F	1.91763700	1.28882700	4.68138200
F	0.49355600	2.67507400	3.79037900
F	2.55080500	2.61513500	3.07206200
C	-1.83019700	-1.17375600	1.36920300
C	-3.31138800	-1.41126500	1.73795200
F	-3.44042400	-2.22006700	2.79765000
F	-3.93119700	-0.25319300	2.00756200
F	-3.93250600	-1.98983500	0.68736900
H	0.32636400	1.38518500	-2.49138500
C	1.90541600	3.22510600	-0.83904900
C	0.39656200	3.14902800	-1.10675200
C	0.00448000	3.77159600	-2.37304700
C	0.93408600	4.00464700	-3.42937200
C	2.24419000	3.65558900	-3.30392300
C	2.76968500	2.97635600	-2.08537900
H	-1.03132300	4.05935100	-2.51322500
H	-0.18098900	3.47410300	-0.24093400
H	3.80491900	3.28178200	-1.89625300
H	0.56443300	4.44666700	-4.34974500
H	2.92478500	3.82222600	-4.13554000
H	2.82510100	1.89927900	-2.30171200
H	2.19336200	2.54614900	-0.03859900
H	2.09414300	4.24458600	-0.48181600

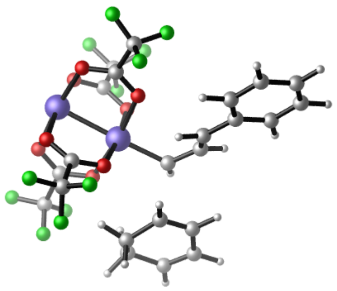

(TS$_{IV\text{-}Vb}$)

E (opt) = -2905.18381971 Hartrees
G (opt) = -2904.881464 Hartrees
E (SP) = -2906.04879948 Hartrees

C	0.63686100	-1.28982000	-1.63958000
C	2.05069500	-1.33251500	-1.85879500
H	2.39231500	-1.42899100	-2.89043300
C	2.96766100	-1.27164500	-0.85049400
H	2.59169000	-1.21102600	0.16630000
C	4.41583400	-1.25570300	-0.97421300
C	5.17458700	-0.90339600	0.16277200
C	5.09705300	-1.55176600	-2.17493000
C	6.56394300	-0.83509500	0.09959300
H	4.65948300	-0.65649400	1.08653100
C	6.48538600	-1.48879700	-2.23164600
H	4.53794100	-1.84417800	-3.05823500
C	7.22376200	-1.12962100	-1.09697500
H	7.13240300	-0.55378800	0.98121400
H	6.99816100	-1.72388800	-3.15984100
H	8.30782400	-1.08326400	-1.14685700
Rh	-0.23259800	0.01203300	-0.28668600

O	-0.44157400	1.39786500	-1.81994600
O	-0.09790100	-1.20913800	1.37882600
O	1.56057000	0.90690600	0.26612400
C	-3.12426700	-0.10406200	-0.16016900
C	-4.47255600	-0.77438900	-0.50395400
F	-4.58647000	-0.96055800	-1.83107100
F	-4.52374900	-1.98597400	0.09106100
F	-5.51635700	-0.05274600	-0.08374100
O	-2.13726700	-0.66300700	-0.72057700
O	-3.15576500	0.86228900	0.63849400
Rh	-1.33504300	1.71289300	1.22717700
O	-1.37748500	2.96621200	-0.44457300
O	0.58399800	2.38080900	1.70961600
O	-1.19183100	0.26473900	2.73750000
C	-0.94458300	2.52427600	-1.53669200
C	-0.62798000	-0.82044700	2.46117900
C	-1.03160400	3.47180600	-2.75419800
F	-1.82768100	2.92984700	-3.69598000
F	0.19034600	3.64690200	-3.29082300
F	-1.52417800	4.67108200	-2.42366200
C	-0.58541000	-1.89716000	3.56637400
F	-1.00667700	-1.42816200	4.74560000
F	0.66556600	-2.36854500	3.71540000
F	-1.37935400	-2.92763900	3.20740900
C	1.55149500	1.81547400	1.14390000
C	2.96833700	2.22450600	1.60237100
F	2.96403800	3.36070200	2.30774100
F	3.46811800	1.24138500	2.38684300
F	3.79114000	2.37278900	0.55210600
H	0.02399900	-1.39996900	-2.53597600
C	-1.34159800	-3.58664000	-0.33948000
C	0.09985800	-3.36773900	-0.72426700
C	0.55667800	-3.90445300	-1.90486200
C	-0.33915800	-4.50717700	-2.86556000
C	-1.67956600	-4.42352000	-2.70226800
C	-2.28242500	-3.67364200	-1.55245600
H	1.61863200	-3.89146200	-2.12735600
H	0.79764300	-3.10368900	0.06134600
H	-3.24014900	-4.11583600	-1.25991700
H	0.08844000	-4.99771600	-3.73504700
H	-2.34659700	-4.84185500	-3.45257000
H	-2.52092000	-2.66056900	-1.90562400
H	-1.68751300	-2.82413500	0.35675500
H	-1.36214300	-4.54079300	0.21205500

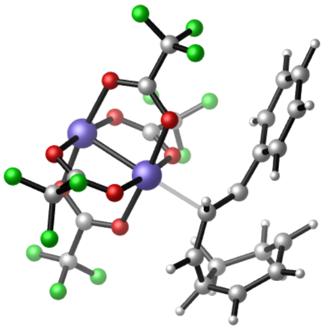

(TS$_{VI-VII}$)

E (opt) = -2905.20674028 Hartrees
G (opt) = -2904.901189 Hartrees
E (SP) = -2906.06779063 Hartrees

Rh	-1.30344100	-1.44124600	1.43808700
O	-2.20030300	-2.44674600	-0.15259600
O	-0.31441000	-0.29520900	2.88118900
O	0.39046400	-2.65937200	1.28426900
C	-2.79344200	0.93540000	0.67782300
F	-4.63886900	1.85360900	-0.47761100
F	-4.83585500	1.67343500	1.68488300
F	-3.52103500	3.18773900	0.83421500
O	-2.87090500	-0.05441600	1.44949900
O	-1.88860000	1.24977200	-0.14445100
Rh	-0.23839700	0.00188400	-0.29191700
O	-1.20977900	-1.12622800	-1.72846100
O	1.33181200	-1.33730500	-0.31724200
O	0.62943100	1.03753700	1.29040700
C	-1.95168600	-2.06618500	-1.32463000
C	0.39687800	0.65603000	2.47144100
C	-2.64844500	-2.83582500	-2.46815700
F	-3.37583900	-3.86161600	-2.01278500
F	-1.72980500	-3.31222800	-3.32816300
F	-3.46192700	-2.00220100	-3.14444600
C	1.11069700	1.51577300	3.53677000
F	0.71538000	2.79761600	3.42406300
F	2.44455900	1.47337900	3.33978100
F	0.85392600	1.09749200	4.77973400
C	1.29597400	-2.31228500	0.48162300
C	2.59099500	-3.15340500	0.51224200
F	3.17183500	-3.19998000	-0.69535400
F	3.45713800	-2.58317700	1.37573400
F	2.35551200	-4.40801800	0.92074500
C	-3.97553500	1.92934100	0.69320100
C	1.90645400	1.71965300	-1.27404800
C	0.78764500	1.17392300	-1.99594700
C	0.23632200	3.40703100	-3.42084600
C	-0.60868700	3.40155500	-2.16896200
C	-0.27838200	1.99212200	-2.51976600
H	-1.03334200	1.46066000	-3.09113400
H	1.01560100	0.32198700	-2.63764500
C	3.16729500	0.96159200	-1.19014200
H	1.67801800	2.24388700	-0.35883700
C	3.71483400	0.30134400	-2.30385500
C	3.81522200	0.84657100	0.05352500
C	4.86502200	-0.47439500	-2.17101600
C	5.48788500	-0.60059400	-0.92479800
C	4.96061800	0.06388200	0.18655400
H	3.38524400	1.33313900	0.92392900
H	3.23868900	0.40436000	-3.27498500
H	5.27829100	-0.98095300	-3.03866300
H	6.38001500	-1.21166900	-0.82154000
H	5.43667300	-0.03671500	1.15775500
C	-0.05256700	4.05464900	-0.89514300
H	-1.65663100	3.63491600	-2.34537800
C	1.63435100	3.57172900	-3.31141400
C	1.43955500	4.45235100	-0.97935300
H	-0.22848900	3.36807300	-0.06124000
H	-0.64805500	4.94797100	-0.68591000
C	2.21909800	3.70009900	-2.05421300
H	1.92279900	4.32206100	-0.00539700
H	1.52419400	5.51959000	-1.22088300
H	-0.24036100	3.41734400	-4.39440700
H	3.30192500	3.78170900	-2.00309900
H	2.25064700	3.41411200	-4.19362200

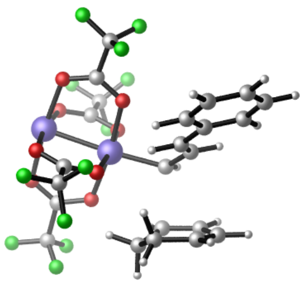

(TS^{cis}$_{IV\text{-}Va}$)

E (opt) = -2905.18666984 Hartrees
G (opt) = -2904.884581 Hartrees
E (SP) = -2906.05263362 Hartrees

C	0.69529000	-0.48616900	-2.09666900
C	2.09133300	-0.15063400	-2.14559500
H	2.50326100	0.11292700	-3.12040600
C	2.90974900	-0.15544800	-1.05791000
H	2.47601300	-0.43808700	-0.10494300
C	4.31637800	0.21172200	-0.99475100
C	4.93150400	0.23553700	0.27444000
C	5.09036300	0.54836800	-2.12562000
C	6.27138600	0.59151200	0.41270700
H	4.34308400	-0.02883700	1.14962300
C	6.42803500	0.90203300	-1.98424800
H	4.64383100	0.52703800	-3.11475600
C	7.02354800	0.92646100	-0.71638000
H	6.72876800	0.60701500	1.39766300
H	7.01331300	1.15796200	-2.86279900
H	8.06883600	1.20307900	-0.61269800
Rh	-0.47250300	0.02099200	-0.45304800
O	-1.66736500	1.20148700	-1.66948500
O	0.60232900	-1.08131600	0.92059400
O	0.76006200	1.64789000	-0.06834500
C	-2.78526400	-1.60024100	0.20215100
C	-3.66453700	-2.85226600	-0.00387300
F	-4.19600700	-2.84753600	-1.24031700
F	-2.90216200	-3.95834100	0.11685700
F	-4.65921600	-2.92499600	0.88687700
O	-1.85989100	-1.50654300	-0.65538400
O	-3.06408400	-0.85380900	1.17007900
Rh	-1.85852300	0.82473600	1.50950200
O	-2.94984200	1.90690900	0.08786000
O	-0.49212100	2.39984900	1.68802800
O	-0.58979500	-0.34197300	2.71990100
C	-2.60858900	1.84397600	-1.11864400
C	0.31873200	-0.98949000	2.15052800
C	-3.45718600	2.65657100	-2.12253900
F	-4.06975200	1.81763500	-2.98103400
F	-2.66806100	3.48049100	-2.83654200
F	-4.39283900	3.39193800	-1.51107200
C	1.27133300	-1.82976000	3.02724400
F	1.00321700	-1.71163000	4.33038000
F	2.54582600	-1.43897000	2.81378800
F	1.17598400	-3.12968100	2.68374600
C	0.48056400	2.41739800	0.89460500
C	1.54422000	3.51671500	1.10950300
F	1.19093300	4.38072400	2.06840200
F	2.71173300	2.94177000	1.46400500
F	1.74665100	4.20288200	-0.02933800
H	0.15832400	-0.29081700	-3.02678400
C	2.02937400	-3.52231300	-0.59223800
C	2.97601500	-3.50175100	-1.75503800
C	2.59795500	-3.14679700	-3.00787100
C	1.25717600	-2.69168900	-3.26484200
C	0.30358600	-2.67524200	-2.26804700
C	0.55035200	-3.47574600	-1.01222400

H	3.32046100	-3.12830300	-3.81811400
H	4.01452200	-3.76170900	-1.56190700
H	0.20384700	-4.49367400	-1.24877500
H	1.01602500	-2.30157200	-4.24969200
H	-0.72915500	-2.46504400	-2.52178200
H	-0.07101100	-3.12511900	-0.19020500
H	2.26779800	-2.67457000	0.06119300
H	2.21924600	-4.41306900	0.01805300

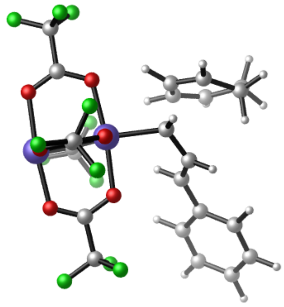

(TS^{cis}$_{IV'\text{-}Vc}$)

E (opt) = -2905.18019178 Hartrees
G (opt) = -2904.882789 Hartrees
E (SP) = -2906.04600316 Hartrees

C	-0.70705800	0.95497600	-1.93128000
C	-2.08353600	0.52174100	-2.00954200
H	-2.45503500	0.19266500	-2.97984300
C	-2.89185600	0.45883600	-0.91891000
H	-2.46940000	0.75750400	0.03484800
C	-4.25860400	-0.04270500	-0.84727700
C	-4.80163600	-0.28898900	0.43043200
C	-5.04857500	-0.32206400	-1.98125700
C	-6.08562100	-0.81160600	0.57195700
H	-4.19937300	-0.07398900	1.30976800
C	-6.33172700	-0.84107700	-1.83657800
H	-4.65958200	-0.12167000	-2.97496000
C	-6.85421400	-1.09044500	-0.56140100
H	-6.48617400	-1.00126400	1.56360800
H	-6.93077000	-1.05013400	-2.71833100
H	-7.85664700	-1.49495500	-0.45450400
Rh	0.49849500	0.11569000	-0.38104600
O	1.61026700	-0.89127000	-1.81356800
O	-0.49970500	1.01694800	1.18688500
O	-0.79699000	-1.50518600	-0.24128100
C	2.91418300	1.49091400	0.44467800
C	3.91429900	2.66445300	0.35801200
F	4.57591500	2.60343300	-0.81470300
F	3.25808900	3.83825800	0.40691700
F	4.80972300	2.63670700	1.35127800
O	1.94715100	1.60117200	-0.36453800
O	3.17410800	0.57837200	1.26483300
Rh	1.87840700	-1.06165100	1.37132300
O	2.87863200	-1.94988800	-0.23408900
O	0.44106800	-2.57372500	1.35073900
O	0.69339600	-0.07104800	2.79892900
C	2.52389000	-1.66479900	-1.40479300
C	-0.21119800	0.68510700	2.37256600
C	3.29750600	-2.35470800	-2.55069100
F	3.79781900	-1.43034900	-3.39183900
F	2.46272000	-3.14715400	-3.25027900
F	4.30895100	-3.10287200	-2.09539400
C	-1.17070500	1.31137600	3.40664300
F	-0.80609600	1.04940900	4.66483400
F	-2.41396800	0.82196100	3.20997000
F	-1.22302300	2.64770300	3.24450600
C	-0.54520400	-2.42403000	0.58700300
C	-1.66455700	-3.48532300	0.66832100
F	-1.32216700	-4.52159700	1.44293900

F	-2.77533200	-2.91804200	1.18243400
F	-1.96340700	-3.94957900	-0.55746800
H	-0.13369800	0.83196900	-2.85121300
C	-2.21557700	3.39383100	-3.29876300
C	-0.83665400	3.07907200	-2.81199900
C	-0.56330200	2.96697200	-1.45715600
C	-1.58795600	3.36009900	-0.47805500
C	-2.71607600	3.96939500	-0.87595400
C	-3.00687800	4.28597500	-2.31943600
H	0.46924500	2.94807100	-1.12984800
H	-0.03008300	2.98166800	-3.53214000
H	-2.77313400	5.34738300	-2.49097400
H	-1.38580900	3.17456300	0.56952900
H	-3.43347200	4.31349100	-0.13410300
H	-4.07823900	4.18053800	-2.52000900
H	-2.75468300	2.44296000	-3.42128400
H	-2.16385400	3.84354000	-4.29414600

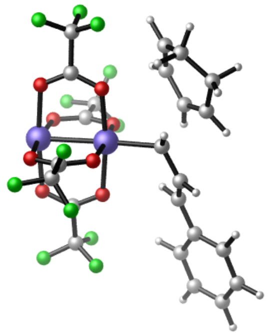

($TS^{trans}_{IV'-Vd}$)

E (opt) = -2905.18069784 Hartrees
G (opt) = -2904.879530 Hartrees
E (SP) = -2906.04474935 Hartrees

C	0.60815400	-0.95228700	-1.98903600
C	2.04955400	-0.87672400	-2.17380800
H	2.39438700	-0.56155200	-3.16042800
C	2.95399500	-1.16027700	-1.20710600
H	2.57696000	-1.45567200	-0.23143900
C	4.40799100	-1.05363600	-1.30827900
C	5.15581500	-0.95870300	-0.11725100
C	5.09731700	-1.02276400	-2.53771800
C	6.54185000	-0.81772200	-0.15260100
H	4.63557900	-0.96738600	0.83624100
C	6.48305600	-0.88722600	-2.56969500
H	4.54730900	-1.12380200	-3.46864100
C	7.21122100	-0.78243700	-1.37904700
H	7.09957800	-0.73528700	0.77601600
H	6.99990900	-0.87076000	-3.52517500
H	8.29214100	-0.67901100	-1.40873400
Rh	-0.24125100	0.00897700	-0.30186200
O	-0.40384900	1.71832500	-1.46693700
O	-0.12562200	-1.57603300	1.03356100
O	1.58599700	0.67780500	0.43843100
C	-3.13396100	-0.04680400	-0.16391000
C	-4.51791900	-0.58507800	-0.58887500
F	-4.57918200	-0.80415000	-1.91311300
F	-4.73478300	-1.76367600	0.03709600
F	-5.50338300	0.25572100	-0.25166200
O	-2.17638700	-0.46212000	-0.87356800
O	-3.12476600	0.68332600	0.85823100
Rh	-1.27596000	1.33334000	1.58713600
O	-1.31153900	2.93755500	0.23961500
O	0.66156800	1.84060400	2.17217300
O	-1.12440800	-0.41759900	2.72961900
C	-0.89617000	2.75283300	-0.93015800
C	-0.58932000	-1.42123200	2.19966200
C	-1.00900200	3.94659500	-1.90480500
F	-1.87795000	3.64043400	-2.88929300
F	0.18490000	4.20515200	-2.46863100
F	-1.43871100	5.05702200	-1.29448400
C	-0.48581100	-2.69573000	3.06618000
F	-0.81692500	-2.46878500	4.34128400
F	0.76560900	-3.18697500	3.02951700
F	-1.31767200	-3.64206400	2.57561900
C	1.60813700	1.38514600	1.48333700
C	3.03773200	1.67078900	1.99414400
F	3.06314900	2.65943600	2.89557000
F	3.52150800	0.55167900	2.58157600
F	3.85750300	1.99254500	0.98097300
H	0.01706500	-0.61796700	-2.84199000
C	-1.25617200	-3.40469500	-0.96906300
C	0.06990200	-3.10444700	-1.47215900
C	0.28572400	-2.86653600	-2.81950400
H	1.28715800	-2.95592300	-3.22372800
H	0.91516600	-3.19961200	-0.80047100
H	-1.35097600	-3.70356900	0.06741200
C	-2.33671100	-3.25903900	-1.76186100
H	-3.32897200	-3.45456900	-1.36512400
C	-2.23183400	-2.69981300	-3.15115600
H	-2.41525900	-1.62075700	-3.06563800
H	-3.02703600	-3.09566900	-3.79045500
C	-0.85733000	-2.95605600	-3.79169300
H	-0.69342100	-2.28908300	-4.64381700
H	-0.81687500	-3.98049900	-4.19402900

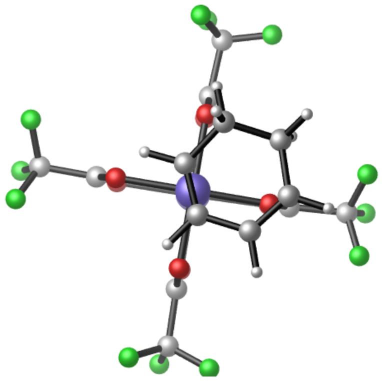

(I)

E (opt) = -2557.44508749 Hartrees
G (opt) = -2557.275250 Hartrees
E (SP) = -2558.22991796 Hartrees

Rh	-0.02394500	-0.01896500	0.74310900
O	1.63878900	-1.13816600	0.24750000
O	-1.72910600	1.11226800	1.05795700
O	1.08827700	1.70742600	0.48461200
C	-1.69031600	-2.14259100	-0.30255700
C	-2.49498200	-3.45470900	-0.17312000
F	-3.38471700	-3.35665100	0.82903800
F	-3.15194300	-3.74885900	-1.29990900
F	-1.65287400	-4.46662800	0.10922200
O	-1.18588400	-1.74143300	0.78293200
O	-1.61100300	-1.63944400	-1.45264100
Rh	-0.51308400	0.10589100	-1.68084300
O	1.23116200	-0.98826500	-1.98917600
O	0.62109100	1.85289300	-1.73916500
O	-2.20235800	1.21157000	-1.17065700
C	1.89151000	-1.32954800	-0.97471100
C	-2.40723000	1.45211500	0.04701000
C	3.21210300	-2.09225700	-1.21388400
F	3.14051400	-3.31355800	-0.65550700
F	4.22656300	-1.42351700	-0.63426400
F	3.48137100	-2.23176300	-2.51564600
C	-3.65003800	2.30328700	0.38937000
F	-4.39139500	1.68126900	1.32211200
F	-4.41414900	2.52541800	-0.68488800
F	-3.25470000	3.48986500	0.88747500
C	1.15410200	2.22743900	-0.66345400

C	2.06855700	3.46954000	-0.74346600
F	3.34289700	3.06165400	-0.90656600
F	1.74005700	4.25780800	-1.77340700
F	1.99817700	4.18694600	0.38798900
C	1.91676000	1.05146000	3.28732400
C	0.52117100	0.50720700	3.12233800
H	-0.31269500	1.19763300	3.21767000
C	0.29646100	-0.84609200	3.18395300
H	-0.71870400	-1.22589600	3.25035800
C	1.40351000	-1.78800200	3.29950100
H	2.03058000	1.27076500	4.36103300
H	2.02343100	2.00307100	2.76168800
C	2.67678000	-1.36782100	3.16233300
H	3.49480300	-2.08226700	3.21743200
H	1.17057200	-2.83508000	3.46768000
C	3.00844900	0.06330700	2.83626800
H	3.13893900	0.12361700	1.74593200
H	3.97240500	0.34381400	3.27307800

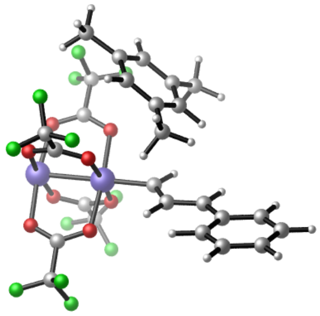

(III)

E (opt) = -3021.98855971 Hartrees
G (opt) = -3021.634693 Hartrees
E (SP) = -3022.87701519 Hartrees

Rh	0.37203800	0.42402000	-0.35386300
O	1.02226600	-1.42887800	-1.03245900
O	-0.09220000	2.29649700	0.40289100
O	-0.62931000	-0.37790300	1.27698400
C	2.74986500	1.52559400	-1.58082000
C	3.52119100	2.11996000	-2.78041300
F	3.57001100	1.21514700	-3.77761900
F	2.89168600	3.21625700	-3.24123100
F	4.77373300	2.45663900	-2.45329400
O	1.53435700	1.28133100	-1.84391800
O	3.39881600	1.34534000	-0.52370900
Rh	2.42789000	0.47371400	1.11308900
O	2.85697000	-1.41282800	0.32839300
O	1.23804600	-0.40013700	2.59308200
O	1.76557700	2.36250300	1.73385000
C	2.09770400	-1.88718100	-0.55309400
C	0.69762700	2.80828100	1.25253100
C	2.56339400	-3.24998200	-1.11558200
F	3.87714900	-3.21111800	-1.39513400
F	1.90833500	-3.59152900	-2.23403000
F	2.36195100	-4.21408300	-0.19355200
C	0.23683000	4.20062800	1.73882000
F	0.19727500	5.05765400	0.70083800
F	1.05405300	4.70204000	2.67117000
F	-1.00289300	4.11766100	2.25844900
C	0.02083300	-0.58221700	2.34101700
C	-0.79415800	-1.16148200	3.52033200
F	-0.48731100	-0.51898300	4.65956400
F	-2.11642600	-1.05638800	3.32317000
F	-0.49141100	-2.46578500	3.68312800
C	-2.58656300	-2.64264300	-1.14027400
C	-2.90214000	-2.59779600	0.22383700
C	-1.24505500	0.39222300	-1.50095500
H	-3.16735900	-2.04756600	-1.84046700
C	-3.98418300	-1.68109600	0.73972000
H	-3.54021500	-0.75533800	1.12341100
H	-4.69584400	-1.40892300	-0.04493700
H	-4.54074600	-2.13882700	1.56434200
C	-1.53714500	-3.43561600	-1.62159300
C	-2.14831400	-3.37500300	1.11104700
C	-1.09045400	-4.17484400	0.66172600
C	-0.80408300	-4.20222700	-0.70880100
H	-2.38080700	-3.34635200	2.17128400
H	0.01245200	-4.82023900	-1.06924400
C	-0.22910500	-4.93680400	1.64123300
C	-1.15744100	-3.40849000	-3.08175500
H	-0.82916800	-4.39188700	-3.43437400
H	-1.99137400	-3.08161400	-3.71130000
H	-0.32304700	-2.71517200	-3.24016600
H	0.17850400	-5.84924200	1.19478300
H	0.62242300	-4.32546700	1.96354700
H	-0.78797900	-5.21462000	2.54019700
H	-1.16159900	-0.06646900	-2.49163100
C	-2.47029000	0.92383000	-1.12978100
H	-2.57422000	1.38325100	-0.15072500
C	-3.56402900	0.81864300	-1.98076000
H	-3.38329900	0.36598500	-2.95534600
C	-4.91553700	1.21207700	-1.71835400
C	-5.87632300	1.02506600	-2.74327800
C	-5.33316600	1.75197200	-0.47453300
C	-7.20460800	1.37392600	-2.53801200
H	-5.55917600	0.60656100	-3.69408600
C	-6.66191000	2.09379100	-0.27660900
H	-4.61730400	1.89179400	0.32763000
C	-7.59766600	1.90757100	-1.30543700
H	-7.93451000	1.23084000	-3.32821700
H	-6.97989800	2.50331800	0.67676500
H	-8.63677400	2.17786000	-1.14193600

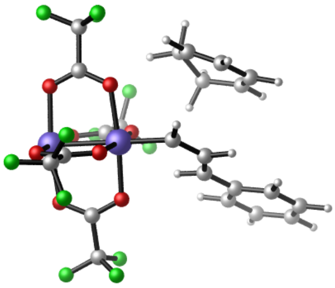

(IV-*cis*)

E (opt) = -2905.19181901 Hartrees
G (opt) = -2904.894354 Hartrees
E (SP) = -2906.05735450 Hartrees

C	0.72034900	-0.16789200	-2.07560600
C	2.05597800	0.18697600	-2.23338300
H	2.52495100	0.02676200	-3.20262400
C	2.82335100	0.65984800	-1.17736100
H	2.33301700	0.76363000	-0.21557000
C	4.21906900	0.98631100	-1.19039200
C	4.83162900	1.30399100	0.04664900
C	5.00915500	0.97756800	-2.36694500
C	6.18827700	1.59797100	0.10731600
H	4.22928000	1.29552400	0.95085100
C	6.36028700	1.28083100	-2.29942500
H	4.55671400	0.73890600	-3.32341700
C	6.95206400	1.58867000	-1.06475200
H	6.65313500	1.83347400	1.05937300
H	6.96235300	1.27809700	-3.20257600
H	8.01199100	1.82138300	-1.02005600
Rh	-0.51005300	0.10029200	-0.51679700
O	-2.01790200	0.75616800	-1.77936100

O	0.85125500	-0.51930400	0.90996600
O	0.12351200	2.04892300	-0.21072600
C	-2.08897000	-2.20793700	0.22414700
C	-2.38413600	-3.72229300	0.15375300
F	-2.45914800	-4.14329900	-1.11996500
F	-1.37105800	-4.38660600	0.75219400
F	-3.52373400	-4.04150400	0.77690300
O	-1.26619300	-1.81760800	-0.65411500
O	-2.64180300	-1.57072200	1.15246600
Rh	-2.11966200	0.44507700	1.40716200
O	-3.50089000	1.00831400	-0.05844700
O	-1.37699400	2.40608200	1.47577400
O	-0.55435700	-0.15104700	2.67010800
C	-3.13915100	1.03372400	-1.25876900
C	0.53130600	-0.47320300	2.13507600
C	-4.19485500	1.48225600	-2.29401300
F	-4.30496500	0.56491600	-3.27173600
F	-3.81235800	2.64700200	-2.85296900
F	-5.39921200	1.65555900	-1.73815100
C	1.67560400	-0.92024500	3.07180900
F	1.43564400	-0.59523000	4.34561100
F	2.83747200	-0.35063700	2.69636600
F	1.82433600	-2.25849200	2.99233200
C	-0.44613500	2.72363600	0.69861800
C	0.15677500	4.13535000	0.87188900
F	-0.58650500	4.90215100	1.67704700
F	1.38973700	4.02290100	1.40888900
F	0.27442900	4.75145300	-0.31644000
H	0.22452900	-0.58771100	-2.95533500
C	3.08700200	-2.79617500	0.10796200
C	3.96640100	-2.75279600	-1.11757600
C	3.44982500	-2.89349600	-2.35195300
C	2.01754700	-3.13600100	-2.54279900
C	1.22173000	-3.44118200	-1.49928000
C	1.78585800	-3.59614000	-0.10829100
H	4.08313300	-2.80562200	-3.23163800
H	5.02367200	-2.53421900	-0.98407900
H	1.97031800	-4.66996700	0.05980300
H	1.61130300	-3.08373000	-3.55007100
H	0.16790800	-3.65110300	-1.64822800
H	1.04182800	-3.30088600	0.63669500
H	2.83818000	-1.76301300	0.38029600
H	3.63634800	-3.20212600	0.96405100

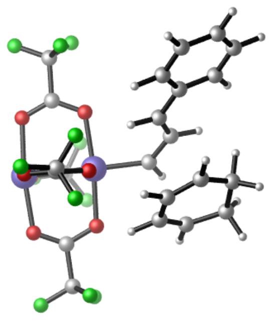

(IV’-*cis*)

E (opt) = -2905.19227305 Hartrees
G (opt) = -2904.892736 Hartrees
E (SP) = -2906.05858903 Hartrees

C	-0.76964900	0.51400700	-1.92279800
C	-2.11844300	0.18786200	-2.04998300
H	-2.57832100	0.28831200	-3.03234700
C	-2.90634500	-0.17513200	-0.96823200
H	-2.43491900	-0.19638300	0.00742800
C	-4.30255500	-0.53621800	-0.99094000
C	-4.93933200	-0.79450900	0.24554900
C	-5.06177000	-0.62669400	-2.18061800
C	-6.28799100	-1.12640100	0.29225700
H	-4.36143400	-0.71285300	1.16224400
C	-6.41020600	-0.96061700	-2.12717200
H	-4.59137700	-0.44766600	-3.14144700
C	-7.02629800	-1.20888600	-0.89424000
H	-6.76788600	-1.31902000	1.24693500
H	-6.98580700	-1.03122700	-3.04505500
H	-8.08034900	-1.46846300	-0.85909800
Rh	0.50142600	0.01012000	-0.44527500
O	1.86375900	-0.69468500	-1.84256500
O	-0.71431600	0.64907700	1.10521500
O	-0.34456400	-1.87426200	-0.26989100
C	2.40007500	2.02399900	0.39772000
C	2.93216500	3.47331700	0.38620400
F	3.33691900	3.81536700	-0.84984200
F	1.93778600	4.31187000	0.74686900
F	3.95462500	3.64412200	1.23124400
O	1.49257300	1.83051800	-0.46440600
O	2.88904800	1.24163500	1.24730500
Rh	2.14085300	-0.71154000	1.34495200
O	3.39036400	-1.26432900	-0.23869200
O	1.18900500	-2.57487000	1.27397800
O	0.69495100	-0.09457900	2.73937900
C	2.97330700	-1.13005400	-1.41398300
C	-0.36500000	0.40449100	2.29746900
C	3.93960700	-1.54288100	-2.54700900
F	4.20236300	-0.47988900	-3.33143200
F	3.37365300	-2.49563800	-3.31157900
F	5.09827900	-2.01406400	-2.07250100
C	-1.44988600	0.79871100	3.32345700
F	-1.10460500	0.48198600	4.57404900
F	-2.60611300	0.16938300	3.02626300
F	-1.67672300	2.12730100	3.26628300
C	0.18709200	-2.70016900	0.52995900
C	-0.56298000	-4.04943900	0.58715500
F	0.07523900	-4.94786900	1.34570500
F	-1.79326600	-3.85401800	1.10359500
F	-0.70573000	-4.55987600	-0.64896600
H	-0.27006500	0.88475200	-2.82196800
C	-2.47349300	3.51727800	-2.78353300
C	-1.27698800	3.33485800	-1.88599900
C	-1.40155700	3.10851000	-0.55771600
C	-2.70054900	2.86617400	0.04301400
C	-3.81442500	2.78394200	-0.71590500
C	-3.81015500	2.98157500	-2.20636800
H	-0.52028600	3.07195900	0.06999500
H	-0.29365600	3.50696100	-2.31599600
H	-4.62977200	3.65664900	-2.48011900
H	-2.74987000	2.69804000	1.11345000
H	-4.76961000	2.54573900	-0.25406300
H	-4.07834900	2.02021500	-2.66772100
H	-2.27754000	3.07042300	-3.76446000
H	-2.55948600	4.59797900	-2.97270600

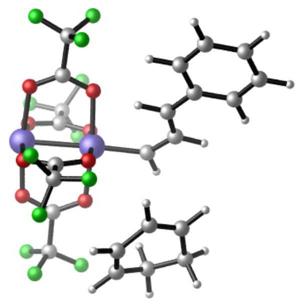

(IV'-*trans*)

E (opt) = -2905.18888310 Hartrees
G (opt) = -2904.890781 Hartrees
E (SP) = -2906.05361988 Hartrees

C	0.75998500	-0.59281800	-1.99244600
C	2.13085900	-0.67654900	-2.20412500
H	2.48570900	-1.14720300	-3.12012300
C	3.05476400	-0.19028700	-1.28463000
H	2.66287100	0.26679900	-0.38333300
C	4.48184000	-0.21317900	-1.38826900
C	5.22471200	0.45008500	-0.37892600
C	5.17690400	-0.85372000	-2.44538600
C	6.61216900	0.47763300	-0.42812800
H	4.69223500	0.95622100	0.42140500
C	6.56255600	-0.82802200	-2.48245900
H	4.62692300	-1.37352900	-3.22233100
C	7.28112400	-0.16310300	-1.47731100
H	7.17491000	0.99307500	0.34349300
H	7.09275100	-1.32352900	-3.28948500
H	8.36644300	-0.14636300	-1.51477700
Rh	-0.28314000	0.21298700	-0.48420600
O	-1.36361300	1.45486900	-1.74480500
O	0.71266500	-0.91100400	0.94921200
O	1.06122400	1.74754600	-0.13202900
C	-2.78964500	-1.13003600	0.03406800
C	-3.89936400	-2.17883300	-0.21064800
F	-3.71361000	-2.86226300	-1.34790800
F	-3.92218100	-3.05784900	0.81221200
F	-5.09954600	-1.57843500	-0.27076700
O	-1.77934100	-1.20677400	-0.72108000
O	-3.03784500	-0.33312200	0.97316300
Rh	-1.65036100	1.15579000	1.42343800
O	-2.53471600	2.39040600	-0.01844400
O	-0.08031700	2.53087500	1.68345800
O	-0.61810900	-0.18060300	2.65541200
C	-2.20106100	2.24747900	-1.21850200
C	0.30372700	-0.86287800	2.14443400
C	-2.88735200	3.17239000	-2.24871700
F	-3.50076100	2.43604800	-3.19369700
F	-1.96262500	3.94329900	-2.85229200
F	-3.79622300	3.97079100	-1.67769800
C	1.02998000	-1.79588900	3.14039100
F	1.42977700	-1.10467400	4.22050000
F	2.10403200	-2.38556800	2.59593700
F	0.18185200	-2.76047700	3.55192700
C	0.88568600	2.47853300	0.88932600
C	2.09749800	3.38701400	1.18958300
F	1.82087100	4.30806800	2.11688300
F	3.10909700	2.61229600	1.64869600
F	2.52283200	4.00709500	0.07821200
H	0.12678600	-1.02894100	-2.77154900
C	0.01629100	-4.13694100	0.38579000
C	1.26316600	-3.77568800	-0.29491600
C	1.42166900	-4.00891700	-1.61142600
H	2.35965000	-3.77186200	-2.10780700
H	2.05383200	-3.31908300	0.29121700
H	-0.00590900	-4.11921800	1.47167200
C	-1.08624600	-4.43516600	-0.32497600
H	-2.02844400	-4.64695200	0.17263100
C	-1.06117700	-4.35968400	-1.83348400
H	-1.33852700	-3.33265100	-2.11542900
H	-1.81944600	-5.01674800	-2.27240300
C	0.32851400	-4.69169500	-2.40029700
H	0.37944400	-4.41469400	-3.45890800
H	0.49751400	-5.78119000	-2.36054200

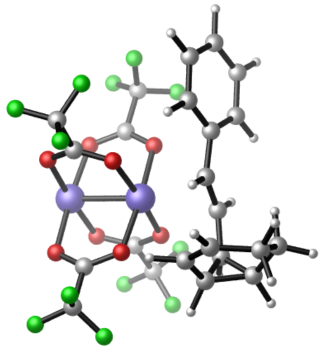

(VI) or (VIc)

E (opt) = -2905.23316170 Hartrees
G (opt) = -2904.927567 Hartrees
E (SP) = -2906.09389530 Hartrees

C	2.18374200	1.15849600	-2.36934000
C	0.72559700	1.33818800	-2.22040500
H	0.14426500	0.78741100	-2.95914800
C	0.05720400	2.22747500	-1.41287700
H	0.63469800	2.78763600	-0.68461500
C	-1.37011200	2.55917400	-1.47894700
C	-1.94716600	3.25024000	-0.39590000
C	-2.19885900	2.17248300	-2.55007800
C	-3.31540000	3.51012800	-0.36422900
H	-1.31979700	3.54589900	0.43795700
C	-3.56414400	2.44073900	-2.52068400
H	-1.77483800	1.65543300	-3.40502300
C	-4.12992200	3.10049500	-1.42373500
H	-3.74626300	4.02458300	0.48992700
H	-4.19180000	2.12995400	-3.35085400
H	-5.19753000	3.29954900	-1.39985100
Rh	0.08177400	-0.08295700	-0.22502300
O	0.61536100	-1.55738600	-1.58347300
O	-0.45724700	1.22943900	1.28120300
O	-1.87873500	-0.28759600	-0.82813500
C	2.33565300	-0.81558100	1.41800700
C	3.78955700	-0.62509100	1.90083500
F	4.63178300	-0.63970400	0.85234500
F	3.89804200	0.57442300	2.50740800
F	4.16332100	-1.57437100	2.76365400
O	2.01408800	-0.02490000	0.48990500
O	1.64322700	-1.69164900	1.99842100
Rh	-0.34444700	-1.90091000	1.39801200
O	0.24897100	-3.23342700	-0.08217100
O	-2.30314300	-1.94362700	0.67668600
O	-0.92160700	-0.43571200	2.76182100
C	0.57653000	-2.76010400	-1.20132400
C	-0.83856900	0.76254100	2.38909100
C	0.97820000	-3.77102600	-2.29748100
F	2.17435000	-3.43740100	-2.81309400
F	0.06792300	-3.73812900	-3.28881400
F	1.04986600	-5.01965900	-1.82547900
C	-1.25220600	1.85179300	3.40254400
F	-1.66022500	1.32861300	4.56262000
F	-2.25256100	2.59275800	2.88909900
F	-0.20859100	2.66701300	3.64049900
C	-2.60633200	-1.12848200	-0.23397900
C	-4.08772100	-1.08699800	-0.66723900
F	-4.74270100	-2.19657900	-0.30508400

F	-4.68089100	-0.02894500	-0.07977900
F	-4.19253500	-0.94091400	-1.99722800
H	2.42149600	0.16957800	-2.75232400
C	2.63727500	3.70294000	-3.07637200
C	3.11873800	2.26163300	-2.92281700
C	3.30248000	1.75370400	-1.52098200
C	3.12511700	2.72551200	-0.40521300
C	3.14103700	4.04740900	-0.62048600
C	3.24243200	4.63724800	-2.00857500
H	4.10293100	1.03762200	-1.34355600
H	3.81841200	1.91359200	-3.67796100
H	4.29928300	4.84076900	-2.23897200
H	3.04782800	2.31886700	0.59933200
H	3.07913500	4.73505500	0.22115500
H	2.73706300	5.60964200	-2.04063200
H	1.54593500	3.74938400	-2.98915800
H	2.88335000	4.06647600	-4.07902600

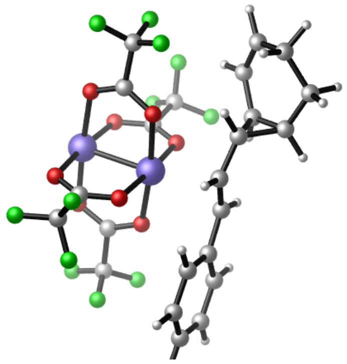

(VId)

E (opt) = -2905.23852204 Hartrees
G (opt) = -2904.933444 Hartrees
E (SP) = -2906.10050890 Hartrees

C	-2.08122800	-1.74115300	-1.98875700
C	-0.61185300	-1.71218600	-2.09623300
H	-0.23189200	-1.10098100	-2.91313300
C	0.28052500	-2.45939700	-1.36827200
H	-0.11278600	-3.10597900	-0.58542100
C	1.72262800	-2.59376700	-1.60988600
C	2.45798200	-3.45051200	-0.76956200
C	2.41645900	-1.85959100	-2.59204600
C	3.84040800	-3.56795200	-0.89713200
H	1.93675500	-4.00871500	0.00351200
C	3.79832600	-1.97259200	-2.71401900
H	1.88259100	-1.17837900	-3.24490700
C	4.51726900	-2.82411500	-1.86686600
H	4.38988100	-4.22703400	-0.23131900
H	4.31863700	-1.38945700	-3.46851600
H	5.59626500	-2.90444900	-1.96281800
Rh	0.11009800	-0.15113800	-0.15734200
O	1.26231100	0.85750400	-1.53682700
O	-1.02609300	-1.02866400	1.32979600
O	1.81771400	-0.90858300	0.72921600
C	-1.96973300	1.86389100	-0.33368000
C	-3.37290300	2.34172400	-0.76726000
F	-3.70461000	1.86218900	-1.97413900
F	-4.27410200	1.89179900	0.12901900
F	-3.44270900	3.67941100	-0.80249400
O	-1.58113200	0.79990300	-0.88252600
O	-1.40177800	2.55352300	0.55460400
Rh	0.36961800	1.79350000	1.34424900
O	1.50023400	2.66958300	-0.17355400
O	2.11093800	0.91662500	2.05656900
O	-0.78908800	0.75425500	2.72870600
C	1.69045700	2.00428700	-1.22461100
C	-1.22866600	-0.37648000	2.38973500
C	2.57296100	2.64559200	-2.31735100
F	1.85592700	2.79613000	-3.44612400

F	3.61540300	1.83916900	-2.58730800
F	3.04171400	3.84100600	-1.94631000
C	-2.20813200	-1.03510200	3.38516200
F	-1.85758000	-0.78345300	4.65263700
F	-2.26930900	-2.36103000	3.20826500
F	-3.44143600	-0.52440400	3.17692100
C	2.42890900	-0.20269200	1.57526700
C	3.77525500	-0.80849100	2.02856500
F	4.27556900	-0.17093800	3.09306900
F	3.62782600	-2.10741700	2.33511600
F	4.65995900	-0.71343400	1.01703900
H	-2.55043900	-0.85688700	-2.40303900
C	-4.00399800	-1.57510300	-0.28686500
C	-2.81360600	-2.32089900	-0.76944600
C	-2.90198100	-3.02383900	-2.09342600
H	-2.31751200	-3.93655600	-2.19001700
H	-2.18210000	-2.75287700	-0.00139200
H	-3.98124300	-1.21557600	0.73869600
C	-5.05095000	-1.31661800	-1.08222400
H	-5.88765300	-0.73209200	-0.70556600
C	-5.10003800	-1.76628400	-2.52376200
H	-4.75687700	-0.94200800	-3.16554800
H	-6.13628200	-1.95829300	-2.82534900
C	-4.25083000	-3.02869300	-2.79455100
H	-4.12386000	-3.16528900	-3.87454400
H	-4.79899000	-3.90627400	-2.42720900

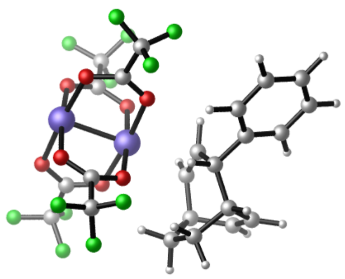

(VII)

E (opt) = -2905.25115412 Hartrees
G (opt) = -2904.942942 Hartrees
E (SP) = -2906.11196205 Hartrees

C	0.75347900	-1.21664700	-2.38280400
C	1.71084800	-0.56642700	-1.65958700
H	1.84567900	0.49717400	-1.83948900
C	2.77725800	-1.22001400	-0.79476000
H	2.44336400	-1.14845400	0.24621600
C	4.05689500	-0.39906900	-0.88648100
C	4.44808900	0.39684900	0.19899300
C	4.83432900	-0.37190500	-2.05391100
C	5.59243900	1.19436900	0.12724900
H	3.84201200	0.40667400	1.09986000
C	5.98371700	0.41827400	-2.12578300
H	4.53568800	-0.96746600	-2.91168100
C	6.36771600	1.20365100	-1.03481000
H	5.87263200	1.80966700	0.97797100
H	6.57673300	0.42446600	-3.03637800
H	7.25996400	1.82077100	-1.09288400
Rh	-0.31908300	0.01084900	-0.33354500
O	-0.91034700	1.47714100	-1.66391300
O	0.17897100	-1.36767800	1.11293300
O	1.11871800	1.28271000	0.45175800
C	-2.99665900	-1.07578800	-0.37245600
C	-4.07778800	-2.04071900	-0.90496700
F	-4.27823900	-1.81851500	-2.21606800
F	-3.65989000	-3.31044600	-0.75224700
F	-5.24045000	-1.89286000	-0.26283900
O	-1.88797800	-1.18408500	-0.96762100

O	-3.32278700	-0.31201400	0.57235600
Rh	-1.87546600	0.98288400	1.31108000
O	-2.36802100	2.37253900	-0.15599400
O	-0.31690500	2.19768600	1.96331200
O	-1.26075900	-0.49551100	2.64538600
C	-1.78092400	2.30316400	-1.26697500
C	-0.39746600	-1.31218200	2.23523000
C	-2.14084700	3.37975000	-2.31445400
F	-2.51389400	2.79427700	-3.46542400
F	-1.05911300	4.14175500	-2.55992500
F	-3.13386300	4.17069800	-1.89619900
C	0.03585900	-2.43959200	3.19577000
F	-0.50822600	-2.30512900	4.40794400
F	1.37555400	-2.44298600	3.31993800
F	-0.33874800	-3.62589400	2.68093200
C	0.80181500	2.04243200	1.40765700
C	1.98878300	2.83947200	1.99317500
F	1.57575200	3.87442200	2.73172800
F	2.71372300	2.01497300	2.78005600
F	2.78773100	3.29394600	1.01749200
H	0.14646500	-0.62405300	-3.06414600
C	1.84936900	-3.59409200	-0.40676200
C	2.98430500	-2.75852000	-1.05662400
C	3.05431400	-3.13803100	-2.52078100
C	1.91941600	-3.15031800	-3.23275200
C	0.63977600	-2.72555700	-2.52182400
C	0.51470600	-3.51219400	-1.18652300
H	4.00664100	-3.42194900	-2.96116200
H	3.92486900	-3.01821600	-0.56171600
H	0.17531100	-4.52003100	-1.44539300
H	1.88691400	-3.42083200	-4.28473400
H	-0.23612900	-2.93952200	-3.13819100
H	-0.27067500	-3.06333600	-0.57724100
H	1.70758500	-3.27494600	0.62911900
H	2.19093600	-4.63384700	-0.37839200

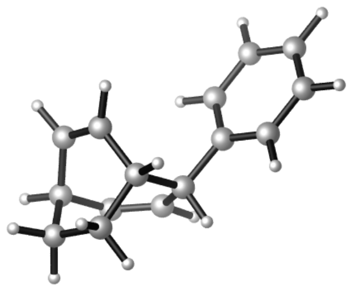

(3a)

E (opt) = -581.246405416 Hartrees
G (opt) = -581.015567 Hartrees
E (SP) = -581.383238242 Hartrees

C	1.90834700	-1.05586400	1.28684200
C	2.74568000	-0.93206200	0.02572000
C	1.90462500	-1.15608700	-1.22510700
C	0.78125400	-0.49660300	-1.53469800
H	2.27188900	-1.90328500	-1.92723400
C	0.10553500	0.59581200	-0.73106700
H	0.26749500	-0.75588300	-2.46027800
C	-1.33989600	0.24408700	-0.39121800
H	0.06715800	1.48512400	-1.37824600
C	-1.72278500	-1.06682400	-0.07353700
C	-2.31099000	1.25476800	-0.33614700
C	-3.03987700	-1.35952700	0.28925200
C	-3.99760700	-0.34355000	0.34198300
C	-3.62832000	0.96731500	0.02694000
H	-2.02950500	2.27652600	-0.58174000
H	-0.98117500	-1.85915300	-0.11024000
H	-3.31750900	-2.38211800	0.53134600
H	-5.02255700	-0.57133400	0.62169900
H	-4.36654000	1.76436000	0.05946900
C	3.37521300	0.48929200	0.00914200
H	3.55118000	-1.67244400	0.03713700
C	1.02235600	-0.08875000	1.54417500
C	2.29817400	1.59333800	0.17387500
H	3.93424100	0.62971200	-0.92223900
H	4.10040100	0.54652600	0.82859400
C	0.89177000	1.04269900	0.54779400
H	2.20857600	2.18508500	-0.74396000
H	2.61074700	2.28748200	0.96065800
H	2.04876900	-1.90881200	1.94697300
H	0.31052400	1.85904100	0.98951600
H	0.38542800	-0.10909100	2.42485400

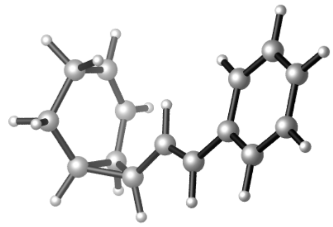

(3a')

E (opt) = -581.229027278 Hartrees
G (opt) = -581.002382 Hartrees
E (SP) = -581.366344752 Hartrees

C	2.98406800	0.77872700	-0.95636500
C	2.92154000	1.11108300	0.51078900
C	1.68651600	1.36762800	-0.34864700
C	0.43359400	0.59402000	-0.27657000
H	1.53439500	2.42311300	-0.56752400
C	-0.78182200	1.15687000	-0.14113300
H	0.51967300	-0.48498000	-0.35985000
C	-2.07299800	0.45658700	-0.07664600
H	-0.84330500	2.24265400	-0.05879300
C	-2.21370100	-0.93218700	-0.27984600
C	-3.23690400	1.19932200	0.20283300
C	-3.46194700	-1.54491900	-0.19880000
C	-4.60720400	-0.79186000	0.08489800
C	-4.48728700	0.58522200	0.28399800
H	-3.15357000	2.27207900	0.36082100
H	-1.34267300	-1.53790900	-0.51173200
H	-3.54383700	-2.61639100	-0.36152100
H	-5.57885600	-1.27373400	0.14569000
H	-5.36765500	1.18361500	0.50289900
C	3.07654100	-0.01424200	1.52304000
H	3.42971200	2.02587000	0.80953400
C	3.06276100	-0.65994900	-1.31911700
C	2.64049900	-1.41321300	1.03452800
H	2.54694700	0.23335300	2.45025900
H	4.14322700	-0.05603300	1.77759800
C	2.92371800	-1.65204600	-0.42705200
H	1.56579500	-1.55132400	1.21822300
H	3.13682900	-2.18021800	1.64147700
H	3.52317400	1.45493600	-1.61533800
H	2.98406200	-2.68649300	-0.76051100
H	3.24183300	-0.89479100	-2.36668700

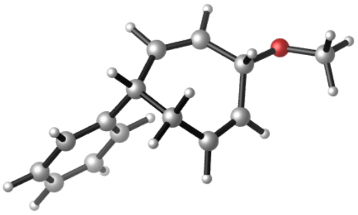

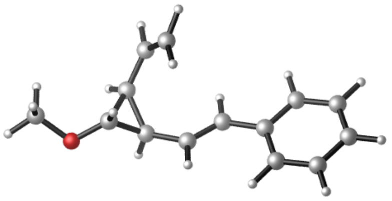

(3i)

E (opt) = -618.340220152 Hartrees
G (opt) = -618.115712 Hartrees
E (SP) = -618.497265857 Hartrees

C	1.81260200	-0.36205200	1.08426400
C	2.55400700	0.11450700	-0.15197600
C	1.68096000	0.19881100	-1.38926300
C	0.43028200	0.66089000	-1.50078000
H	2.18865500	-0.14545600	-2.28888800
C	-0.49147600	1.23408900	-0.44332200
H	-0.01694600	0.63069900	-2.49422800
C	-1.74909500	0.38490100	-0.26634000
H	-0.82591300	2.20953100	-0.82454000
C	-1.70364200	-1.01590900	-0.30735900
C	-2.97884300	1.00633800	-0.00434000
C	-2.85593000	-1.77477700	-0.08667100
C	-4.07467900	-1.14435400	0.17616700
C	-4.13269500	0.25164800	0.21653200
H	-3.03040700	2.09252600	0.02728700
H	-0.75946000	-1.51082000	-0.51277500
H	-2.80090500	-2.85968200	-0.12240500
H	-4.97154600	-1.73417600	0.34465100
H	-5.07637400	0.75310400	0.41457300
C	0.75154800	0.27860400	1.57963600
C	0.19029800	1.51750900	0.92546100
H	2.18207600	-1.27941000	1.53808400
H	0.23964800	-0.12042500	2.45249300
O	3.63186500	-0.76513700	-0.47942700
C	4.76099200	-0.60869100	0.36390900
H	5.51367800	-1.32919100	0.03144800
H	5.18432400	0.40593600	0.29141900
H	4.53124300	-0.80810400	1.42115300
H	2.96939200	1.12124300	0.04756900
H	-0.54212300	1.99920300	1.58011300
H	0.99376100	2.24702600	0.75396000

(3i')

E (opt) = -618.310154072 Hartrees
G (opt) = -618.091008 Hartrees
E (SP) = -618.467580099 Hartrees

C	2.46398000	-0.74858600	-0.58884900
C	2.79674800	0.51308900	0.19867700
C	1.68063300	0.57423700	-0.78151700
C	0.27191800	0.78783900	-0.35308800
H	1.95856700	1.07746700	-1.70853400
C	-0.73954200	-0.04003400	-0.66136000
H	0.09008800	1.68817400	0.23380000
C	-2.15745700	0.11128200	-0.29886300
H	-0.50233700	-0.94520800	-1.21755800
C	-2.69113000	1.28274900	0.27518800
C	-3.03295600	-0.96557600	-0.53570200
C	-4.04127300	1.36277600	0.60935200
C	-4.89618400	0.27973300	0.37479200
C	-4.38516200	-0.88534900	-0.20137300
H	-2.64095500	-1.87626900	-0.98224300
H	-2.04884900	2.13994900	0.45378000
H	-4.43164700	2.27620500	1.05001600
H	-5.94868300	0.34724400	0.63511600
H	-5.03873700	-1.73221600	-0.39214800
C	1.94091100	-1.97534800	0.06046300
C	1.16767500	-2.05728200	1.15062200
H	3.15658200	-0.93663100	-1.40509200
H	2.25431900	-2.90306500	-0.41814200
O	3.95567800	1.22362200	-0.11119000
C	5.11919200	0.64292500	0.46873500
H	5.97078800	1.26126300	0.17233400
H	5.05960500	0.62402000	1.56728100
H	5.27927400	-0.38428100	0.11146500
H	2.54226200	0.48224200	1.26260000
H	0.86635500	-3.02278500	1.54792400
H	0.80382800	-1.17980900	1.67646300

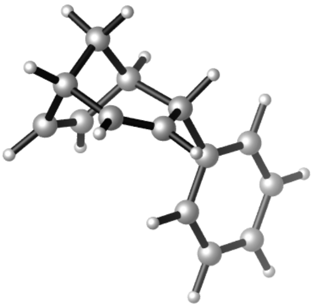

(3r)

E (opt) = -541.921365626 Hartrees
G (opt) = -541.718166 Hartrees
E (SP) = -542.049682507 Hartrees

H	-4.75639300	-0.74540500	0.50167100
C	-3.73684400	-0.45046300	0.26903800
C	-3.40632900	0.90178500	0.14418000
H	-4.16950300	1.66397500	0.27790600
C	-2.09585900	1.27754400	-0.15944800
H	-1.84564900	2.33157200	-0.25842500
C	-1.09325000	0.31415200	-0.34211900
C	-1.43741800	-1.03994900	-0.21427200
H	-0.67053000	-1.79679200	-0.35228500
C	-2.74682600	-1.42026400	0.08851000
H	-2.99391000	-2.47457500	0.18180700
C	0.33874000	0.74500900	-0.62081900
H	0.28966900	1.72686500	-1.11354500
C	1.15803600	0.96568800	0.70314200
H	0.73178800	1.81497900	1.24576300
C	2.64351700	1.14306300	0.31425000
H	3.22361000	1.50543500	1.16966000
H	2.79596700	1.82046600	-0.53205500
C	3.00095200	-0.32597900	-0.01444400
H	4.07373100	-0.52781400	-0.07259500
C	2.28623800	-0.69169200	-1.31295900
H	2.76961500	-1.37042000	-2.01229700
C	1.06638700	-0.20015700	-1.56639400
H	0.53716300	-0.47250900	-2.47801200
C	1.23610600	-0.31163800	1.52921900
H	0.48861100	-0.60423200	2.25899800
C	2.28335100	-1.04541300	1.13321500
H	2.54503200	-2.04165600	1.47681000

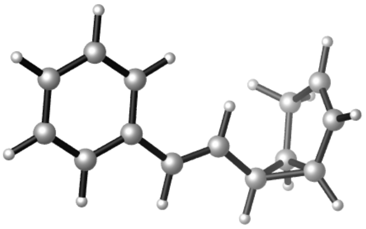

(3r')

E (opt) = -541.902927014 Hartrees
G (opt) = -541.704719 Hartrees
E (SP) = -542.031908837 Hartrees

C	3.20948900	-0.71533600	0.77803200
C	3.16210900	-0.71847900	-0.73638100
C	1.93973700	-1.19425200	0.04462800
C	0.66287900	-0.46052600	0.06810100
H	1.82794600	-2.27691000	0.04333600
C	-0.54005400	-1.05949500	-0.01431800
H	0.72763100	0.62087100	0.15860300
C	-1.85709500	-0.40633300	-0.00730700
H	-0.56946000	-2.14703400	-0.09166500
C	-2.03161800	0.99330500	0.00883700
C	-3.01361200	-1.21067000	-0.02165200
C	-3.30574900	1.55606100	0.01734400
C	-4.44371400	0.74107200	0.00693300
C	-4.28987500	-0.64677800	-0.01388500
H	-2.90388500	-2.29248200	-0.03766400
H	-1.16564700	1.64847900	0.00960300
H	-3.41324500	2.63744300	0.02948000
H	-5.43541000	1.18432200	0.01228800
H	-5.16375900	-1.29270600	-0.02438900
H	3.69148900	-1.47148500	-1.31198200
C	3.27356400	0.72454900	1.16812700
C	3.23121100	0.74106500	-1.19947700
C	3.26915500	1.52838500	0.09543800
H	2.37250500	1.02443700	-1.82353800
H	4.12843900	0.92129800	-1.80669400
H	3.75502000	-1.45999200	1.35164300
H	3.25848400	2.61419200	0.12171400
H	3.29133300	1.05413800	2.20274000

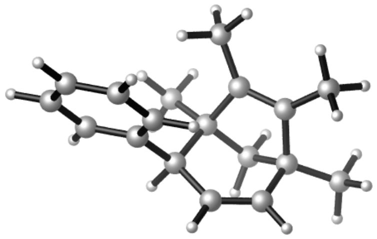

(3s)

E (opt) = -699.218969493 Hartrees
G (opt) = -698.911395 Hartrees
E (SP) = -699.379158524 Hartrees

H	5.15538800	1.33135700	-0.09921100
C	4.18733700	0.85205400	-0.21623700
C	4.04364400	-0.51533800	0.03174500
H	4.90151500	-1.10667800	0.34079500
C	2.79789400	-1.12888800	-0.12288100
H	2.69506500	-2.19441600	0.06675800
C	1.67325600	-0.39148600	-0.52112000
C	1.83448700	0.97935600	-0.77720500
H	0.97395000	1.56500700	-1.08725500
C	3.07694600	1.59728100	-0.62329900
H	3.17824000	2.66075600	-0.82338300
C	0.31231000	-1.05949100	-0.65350100
H	0.49911500	-2.12805600	-0.83839400
C	-0.56322500	-1.01073300	0.66883000
C	-1.98681000	-1.44970900	0.26170400
H	-2.58962300	-1.63451300	1.15937300
H	-2.00637700	-2.34910500	-0.36430000
C	-2.48935200	-0.18955900	-0.47403800
C	-1.72163400	-0.11536700	-1.79175700
H	-2.22630000	0.28265500	-2.67088200
C	-0.44874200	-0.52028100	-1.85469300
H	0.10963000	-0.45506000	-2.78711800
C	-0.84054300	0.43870000	1.10034300
C	-1.92337400	0.90863300	0.45255000
C	0.04942000	-1.88440400	1.76232700
H	0.12919900	-2.92557100	1.42806400
H	1.05202800	-1.54585400	2.04113200
H	-0.57363900	-1.86770700	2.66353200
C	0.02621500	1.15226500	2.09469400
H	-0.24004100	2.20882100	2.19007200
H	-0.06438300	0.69695400	3.08979300
H	1.08468100	1.09647400	1.82020400
C	-2.50796500	2.28663900	0.46708700
H	-3.55113500	2.27773300	0.80755000
H	-1.95022100	2.96959300	1.11369700
H	-2.51485600	2.71356900	-0.54510900
C	-4.00139100	-0.14616500	-0.68493300
H	-4.30934000	0.76999600	-1.20163400
H	-4.33089100	-0.99625800	-1.29341400
H	-4.53285000	-0.19073300	0.27203600

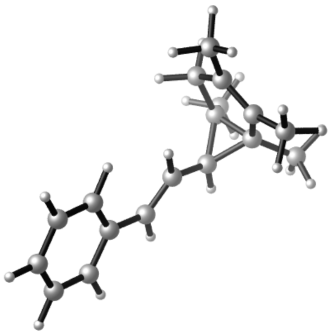

(3s')

E (opt) = -699.199973162 Hartrees
G (opt) = -698.897969 Hartrees
E (SP) = -699.361243343 Hartrees

C	2.37054600	-0.62955500	0.60096600
C	2.27307100	-0.89056900	-0.88604900
C	1.07671200	-1.24186000	0.01082900
C	-0.21360100	-0.53869400	-0.03511200
H	0.98857500	-2.31270100	0.19365100
C	-1.40891400	-1.15309400	0.05635100
H	-0.17185500	0.54252100	-0.14201700
C	-2.73002600	-0.51024000	0.01948300
H	-1.42611900	-2.23586900	0.18643200
C	-2.91920400	0.84949600	-0.30558000
C	-3.87345300	-1.27784300	0.31626900
C	-4.19285500	1.41307900	-0.31759000
C	-5.31699600	0.63704700	-0.01203500
C	-5.14947700	-0.71329400	0.30324800
H	-3.75315800	-2.32977500	0.56464000
H	-2.06360100	1.46833400	-0.55958300
H	-4.31150100	2.46300800	-0.57235200
H	-6.30885200	1.07985200	-0.02480800
H	-6.01306000	-1.32939500	0.53939800
C	2.35692800	0.86925300	0.75853400
C	2.25165200	0.47857500	-1.57197000
C	2.28471900	1.48539600	-0.43751300
H	1.36448300	0.61621300	-2.20660400
H	3.12215600	0.59602600	-2.23443600
C	3.00149400	-2.01246400	-1.59072400
H	2.52984500	-2.23887000	-2.55475200
H	4.04509700	-1.73806600	-1.78935000
H	3.00362900	-2.93438700	-1.00105900
C	3.19267600	-1.46069800	1.56158700
H	4.24155800	-1.13946900	1.56425900
H	2.81375200	-1.37173500	2.58616700
H	3.16812200	-2.52080800	1.29326300
C	2.36040200	1.48686000	2.12455900
H	3.24147800	1.17799200	2.70107800
H	2.35099100	2.57945800	2.08969800
H	1.48258000	1.16020400	2.69864300
C	2.17851400	2.94984400	-0.73499200
H	2.96659100	3.26640100	-1.43164500
H	1.22277000	3.18319700	-1.22427700
H	2.25338300	3.57044200	0.16208200

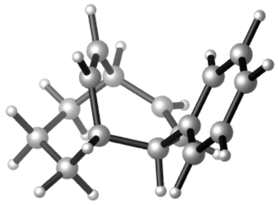

(3t)

E (opt) = -620.560959775 Hartrees
G (opt) = -620.301639 Hartrees
E (SP) = -620.705644369 Hartrees

C	0.79339200	0.03821900	1.46410300
C	0.65432200	1.10486800	0.39462100
C	1.52820700	-1.06582600	1.30054300
C	2.33780400	-1.33372400	0.04646600
C	3.56606900	-0.36575100	-0.04604100
C	2.01525300	1.72575900	-0.08863200
H	2.03191500	2.77944900	0.21171800
H	2.04064800	1.72175200	-1.18464500
C	3.29855500	1.06779000	0.44116700
H	3.90933700	-0.34788000	-1.08805300
H	4.38975000	-0.78231500	0.54624200
H	3.27047500	1.07428400	1.53658900
H	4.15399900	1.69259000	0.15447700
H	0.24221100	0.18078200	2.39154800
H	0.05901800	1.91874700	0.82059000
C	1.52533800	-1.31277100	-1.23963000
H	1.58775000	-1.79948600	2.10274300
H	2.74732600	-2.34700500	0.11989700
C	0.48997300	-0.53988500	-1.59040000
H	1.87734400	-2.02438600	-1.98707600
C	-0.17407000	0.57703100	-0.81780800
H	0.05228000	-0.71258600	-2.57394700
C	-1.60532500	0.23495700	-0.41055200
H	-0.24863400	1.42399600	-1.51610100
C	-1.97714800	-1.05857900	-0.01773300
C	-2.57615100	1.24726500	-0.37384700
C	-3.28170900	-1.33197600	0.40140100
C	-4.23845400	-0.31445300	0.43605200
C	-3.88076400	0.97906300	0.04496000
H	-2.30427800	2.25575700	-0.67818000
H	-4.61817000	1.77737200	0.06163000
H	-3.55052800	-2.34185400	0.70024200
H	-5.25384100	-0.52790900	0.75864900
H	-1.23835800	-1.85323200	-0.04434300

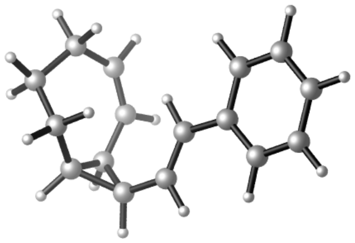

(3t')

E (opt) = -620.540882545 Hartrees
G (opt) = -620.286072 Hartrees
E (SP) = -620.686057832 Hartrees

C	2.29750000	0.14089900	1.63000600
C	2.34589700	1.42032700	1.23032400
C	2.56382600	-1.11304000	0.87677700
C	2.57963000	-1.26217700	-0.61571100
C	2.32792200	-0.09871300	-1.55848700
C	2.61097200	2.01861300	-0.12655600
H	3.26294500	2.89280400	0.01159800
H	1.65624700	2.43550600	-0.48927500
C	3.19746800	1.12650900	-1.22896300
H	1.27233500	0.19556100	-1.54880200
H	2.53600000	-0.43892700	-2.57951200
H	4.20530600	0.79701800	-0.94513000
H	3.31451000	1.74499200	-2.12759200
H	2.05095800	-0.03225300	2.67866900
H	2.11169800	2.16740500	1.99036000
C	1.46457800	-1.95430100	0.18874900
H	3.27037800	-1.75357800	1.40558600
H	3.33829100	-1.95162900	-0.98186100
C	0.02584800	-1.61679000	0.09912800
H	1.62110600	-3.02421000	0.30103800
C	-0.56664900	-0.40747400	0.16773600
H	-0.60514900	-2.49146100	-0.05526900
C	-2.00632300	-0.12634000	0.05590100
H	0.04980300	0.46838800	0.33862500
C	-2.99358000	-1.12016500	-0.10790800
C	-2.43234300	1.21525300	0.11819700
C	-4.34144600	-0.78177900	-0.20602200
C	-4.74574800	0.55671000	-0.14368200
C	-3.78198000	1.55438600	0.01930500
H	-1.68865700	1.99868200	0.24519400
H	-4.08004900	2.59826100	0.07030700
H	-5.08275400	-1.56685400	-0.33021000
H	-5.79798400	0.81574400	-0.22003400
H	-2.70842000	-2.16694600	-0.15645700

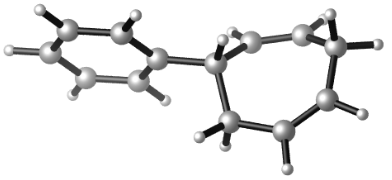

(3u)

E (opt) = -503.818522385 Hartrees
G (opt) = -503.624163 Hartrees
E (SP) = -503.940830585 Hartrees

C	-3.43232500	-0.76997600	0.02815500
C	-3.40575700	0.58278200	-0.65333500
C	-2.44848800	-1.44662300	0.63413700
H	-4.41206900	-1.24790600	0.00686000
C	-0.98749900	-1.09961900	0.78063300
H	-2.71835700	-2.40542100	1.07833200
C	-0.43692400	-0.04978900	-0.21958900
C	-2.36313900	1.57820700	-0.19438100
C	1.08108600	-0.00703200	-0.14825500
C	-1.06794300	1.31363800	-0.00350500
H	-0.71643200	-0.39917800	-1.22303800
H	-0.40972700	2.11805500	0.31927000
H	-2.71370500	2.59642700	-0.03153500
C	1.86282900	-0.51226000	-1.19528400
C	1.73406600	0.50405100	0.98489200
C	3.12748900	0.51285800	1.06617500
C	3.89597400	0.00757100	0.01304200
C	3.25848400	-0.50610900	-1.11838800
H	1.37333600	-0.91319400	-2.07976900
H	3.84597800	-0.90025500	-1.94340500
H	1.14522700	0.89834900	1.80928300
H	3.61362600	0.91446000	1.95132000
H	4.98072000	0.01525900	0.07441400
H	-0.78297800	-0.76015400	1.80632900
H	-0.40902300	-2.02523600	0.65927800
H	-3.30643600	0.42870500	-1.74195400
H	-4.39727500	1.03388900	-0.53117600

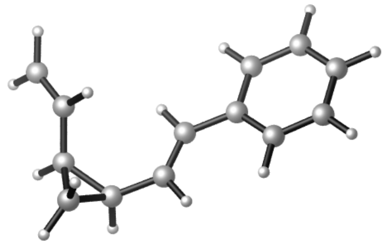

(3u')

E (opt) = -503.790393893 Hartrees
G (opt) = -503.601092 Hartrees
E (SP) = -503.912655544 Hartrees

C	-2.26353500	-1.23182200	-0.60480500
C	-3.24995500	-0.12568200	-0.23355700
C	-2.84953400	1.08205100	0.52196500
C	-3.34819300	2.30528700	0.31348900
H	-2.10586000	0.93338000	1.30349200
C	-3.28192700	-1.47142100	0.47932600
C	-0.80393800	-1.19447100	-0.30461100
H	-2.49724300	-1.72470800	-1.54969800
H	-4.03711900	0.04493400	-0.96517600
C	-0.01614300	-0.11411100	-0.43231100
H	-0.37494500	-2.14970700	-0.00028500
C	1.43321700	-0.03281400	-0.18942600
H	-0.47812000	0.82080300	-0.74581200
C	2.08288200	1.19864800	-0.39818100
C	2.21353900	-1.12622300	0.23786000
C	3.58420100	-0.98850500	0.44401700
C	3.45602700	1.33679800	-0.19190000
C	4.21448300	0.24284600	0.23007200
H	3.93270300	2.29856800	-0.36094100
H	5.28371300	0.34571200	0.39220100
H	1.49846200	2.05450900	-0.72732500
H	1.74637000	-2.09105200	0.41066300
H	4.16569000	-1.84547300	0.77329500
H	-4.11407400	-2.14108200	0.28137100
H	-2.92351800	-1.48169900	1.50586500
H	-4.09237400	2.49513300	-0.45774700
H	-3.03043100	3.15719700	0.90846200

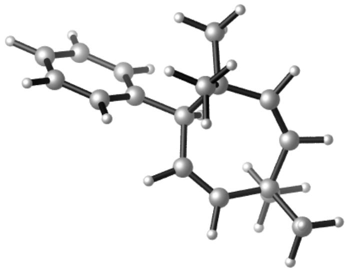

(3w)

E (opt) = -661.095961715 Hartrees
G (opt) = -660.793266 Hartrees
E (SP) = -661.250805678 Hartrees

C	2.74508100	0.78360100	-0.48926600
C	2.94385200	-0.65620500	-0.04262800
C	1.68826200	1.60570500	-0.47310000
H	3.66289600	1.21211800	-0.89630300
C	0.23838700	1.41169400	-0.05045700
H	1.88176700	2.61814300	-0.83204700
C	-0.16360100	-0.11108400	-0.12209400
C	1.89124800	-1.18661700	0.91534400
C	-1.66776600	-0.34736300	-0.05936800
C	0.57522700	-0.96288500	0.89140800
H	0.15867100	-0.42762400	-1.12159300
H	-0.03086500	-1.45135700	1.65132700
H	2.27267300	-1.85932600	1.68353600
C	-2.38233700	-0.61003200	-1.23793200
C	-2.38399500	-0.30307000	1.14807400
C	-3.76659200	-0.50010300	1.17283500
C	-4.46390300	-0.75349000	-0.01097500
C	-3.76447500	-0.80973500	-1.21883800
H	-1.84691100	-0.65662500	-2.18312300
H	-4.29295100	-1.01274200	-2.14655000
H	-1.85977100	-0.11750800	2.08059100
H	-4.29803900	-0.45922000	2.11995100
H	-5.53883600	-0.91023400	0.00860300
C	4.31877500	-0.72960000	0.66605600
H	4.54317900	-1.75919100	0.96701400
H	5.12178300	-0.39142600	0.00215400
H	4.33422300	-0.10281800	1.56465800
C	2.99572300	-1.57052100	-1.29681800
H	3.77466100	-1.23233200	-1.99050300
H	3.22241900	-2.60426600	-1.01197000
H	2.04009200	-1.56363500	-1.82911300
C	-0.62946500	2.21924000	-1.04141100
H	-0.54860200	1.82062200	-2.05906100
H	-1.68391100	2.20120500	-0.75362300
H	-0.30677100	3.26606300	-1.06391600
C	0.05346400	2.00821900	1.36076300
H	0.40020500	3.04747800	1.37972400
H	-1.00061600	2.00738200	1.65569500
H	0.62740800	1.44876300	2.10499900

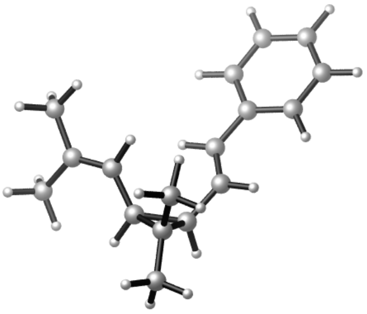

(3w')

E (opt) = -661.080562034 Hartrees
G (opt) = -660.785991 Hartrees
E (SP) = -661.235025820 Hartrees

C	-1.23092700	-1.42134900	-0.86114100
C	-2.28308700	-0.38129800	-0.45430700
C	-1.95727900	0.84424000	0.31117600
C	-2.48199200	2.07313100	0.14555600
H	-1.20996700	0.72926300	1.09228000
C	-2.26599300	-1.77070700	0.19078100
C	0.21955000	-1.31942500	-0.54073800
H	-1.41923000	-1.88349200	-1.83250600
H	-3.06783200	-0.25654500	-1.19548400
C	0.95112500	-0.19387700	-0.59891500
H	0.69712700	-2.26138900	-0.26840000
C	2.38751400	-0.05137700	-0.31113800
H	0.44409900	0.73155800	-0.86696700
C	2.95291200	1.23807100	-0.31691200
C	3.23483300	-1.14123700	-0.02530800
C	4.58647800	-0.94359900	0.24711100
C	4.30701500	1.43634300	-0.04411400
C	5.13136500	0.34564200	0.24059700
H	4.71705800	2.44257800	-0.05386900
H	6.18612300	0.49523500	0.45312800
H	2.31682900	2.09248900	-0.53568900
H	2.83619500	-2.15116600	-0.01990900
H	5.22007400	-1.79940300	0.46426400
C	-3.37899000	-2.72480100	-0.20514500
H	-3.06193300	-3.76869100	-0.08445800
H	-4.26723000	-2.57412000	0.42113200
H	-3.67840200	-2.58510400	-1.24943300
C	-1.83199900	-1.92178400	1.63877900
H	-2.64411200	-1.64031600	2.31977100
H	-1.57221300	-2.96657600	1.84841000
H	-0.95844100	-1.31362300	1.88611600
C	-2.04171500	3.22185300	1.02107700
H	-1.61886800	4.03681500	0.41714300
H	-2.89324900	3.65384600	1.56512700
H	-1.28852500	2.91833800	1.75492800
C	-3.52188200	2.44405700	-0.88219800
H	-4.42620200	2.82995100	-0.39218700
H	-3.15357800	3.25292700	-1.52769600
H	-3.81928500	1.61411800	-1.52667500

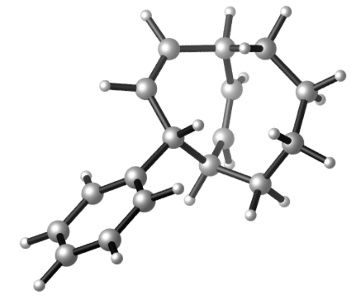

(3x)

E (opt) = -659.864942957 Hartrees
G (opt) = -659.578502 Hartrees
E (SP) = -660.019044157 Hartrees

C	1.56921200	-0.53563600	1.70516200
C	0.40846300	0.22304500	1.09792300
C	2.59178800	-1.11773700	1.07631600
C	2.71105300	-1.23043400	-0.42984800
C	2.97143800	0.14143300	-1.17557800
C	0.77045000	1.73297100	1.03698500
C	1.70134400	2.19573400	-0.10290300
H	1.21555100	2.01003100	2.00097900
H	-0.16190700	2.30777300	0.95454600
C	3.03055700	1.45188100	-0.35378800
H	1.94010300	3.24652700	0.10739400
H	1.14462700	2.21624700	-1.04924400
H	2.22800900	0.25996600	-1.97240800
H	3.93390400	0.03974300	-1.68882900
H	3.55653600	1.28338900	0.59253600
H	3.66141900	2.14149800	-0.92937000
H	1.57821600	-0.51969200	2.79532500
H	-0.40290800	0.13859900	1.82982100
C	1.50121800	-1.99257200	-0.95487400
H	3.38577900	-1.56568200	1.67157200
H	3.58568000	-1.85460900	-0.63202600
C	0.23118100	-1.59455100	-0.82827000
H	1.69763000	-2.95533300	-1.42436900
C	-0.20599300	-0.24654200	-0.28687500
H	-0.55771600	-2.23699700	-1.21551500
C	-1.72078100	-0.12213800	-0.21789200
H	0.12580100	0.47892800	-1.03604200
C	-2.49278600	-1.08457400	0.45306000
C	-2.37591200	0.97770500	-0.78677200
C	-3.87845800	-0.95177800	0.54946900
C	-4.52015400	0.15210000	-0.02195800
C	-3.76392300	1.11709000	-0.68993600
H	-1.79412300	1.73033600	-1.31334400
H	-4.25204500	1.97787200	-1.13927100
H	-4.45851700	-1.70876300	1.07075300
H	-5.59905000	0.25664700	0.05275000
H	-1.99916700	-1.94247200	0.90340100

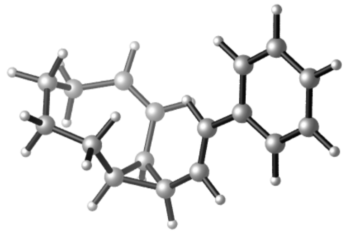

(3x')

E (opt) = -659.858941570 Hartrees
G (opt) = -659.576856 Hartrees
E (SP) = -660.012507800 Hartrees

C	1.81269100	-0.07331100	1.66499100
C	2.62641400	0.98554100	1.57535000
C	2.03204600	-1.34973600	0.92461200
C	2.09315800	-1.40886600	-0.58013600
C	1.90934300	-0.20164000	-1.48075500
C	3.87023000	1.07407500	0.72281200
C	3.62215700	1.62417400	-0.70348800
H	4.35304100	0.09239800	0.64751100
H	4.58310500	1.73360100	1.23187400
C	3.20809900	0.58275900	-1.75729700
H	4.54270100	2.10600400	-1.05695000
H	2.86176900	2.41533700	-0.65530700
H	1.15384700	0.47850900	-1.07938000
H	1.50932800	-0.56628000	-2.43609500
H	4.03404100	-0.12803400	-1.90269900
H	3.09392400	1.11075400	-2.71295900
H	0.95144000	-0.02654600	2.33018100
H	2.38136900	1.87631400	2.15442000
C	0.91450800	-2.06568400	0.15835700
H	2.69862100	-2.04333800	1.43941900
H	2.80664600	-2.12617700	-0.98414600
C	-0.49800100	-1.62477900	0.05609400
H	0.99227200	-3.14910400	0.20207300
C	-1.01896000	-0.38884700	0.19154200
H	-1.17991800	-2.44441800	-0.16786900
C	-2.43753000	-0.01837000	0.06522700
H	-0.35758800	0.44254500	0.41764400
C	-3.47728200	-0.95080200	-0.13135500
C	-2.78770100	1.34371400	0.15157400
C	-4.80194000	-0.53443200	-0.24205300
C	-5.13053100	0.82348400	-0.15790100
C	-4.11442100	1.76099100	0.04004700
H	-2.00344700	2.08116000	0.30619700
H	-4.35377900	2.81885400	0.10899100
H	-5.58460100	-1.27358000	-0.39160900
H	-6.16515100	1.14340000	-0.24352600
H	-3.25130400	-2.01102900	-0.19348000

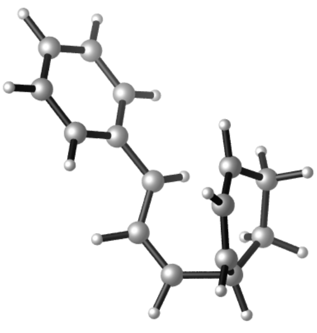

(TS$_{3a'-3a}$)

E (opt) = -581.194027887 Hartrees
G (opt) = -580.966638 Hartrees
E (SP) = -581.332809082 Hartrees

C	2.85013500	-0.93209200	0.80795100
C	3.07936700	-0.33316000	-0.54903300
C	2.03248300	-1.30163900	-1.04775100
C	0.65026500	-1.16643500	-1.00928800
H	2.42563700	-2.26652000	-1.35960200
C	-0.06138000	-0.01095800	-0.64934100
H	0.08253700	-2.08936900	-1.13425800
C	-1.49618200	-0.02342000	-0.34277000
H	0.31023700	0.94643700	-0.98244200
C	-2.14498900	-1.15382600	0.19700600
C	-2.26905000	1.13689600	-0.55809900
C	-3.51052400	-1.13139000	0.47739400
C	-4.26395300	0.02377900	0.24393200
C	-3.63290900	1.16043900	-0.27166500
H	-1.78772100	2.02370500	-0.96411900
H	-1.56851600	-2.04792800	0.41638600
H	-3.98753100	-2.01560400	0.89230900
H	-5.32658500	0.04044400	0.46907600
H	-4.20571500	2.06611400	-0.45351400
C	2.85066000	1.18770300	-0.67008600
H	4.05478700	-0.59860300	-0.96131100
C	1.86860500	-0.40027800	1.63930000
C	1.94605800	1.79350100	0.43591700
H	2.43989600	1.39797300	-1.66339300
H	3.82871200	1.67806800	-0.63492700
C	1.20635200	0.76281000	1.26192500
H	1.24534000	2.51895600	0.00926600
H	2.57298500	2.36538200	1.13533400
H	3.38643600	-1.83698600	1.07535900
H	0.39016000	1.13045600	1.87868000
H	1.50969800	-0.98409200	2.48480700

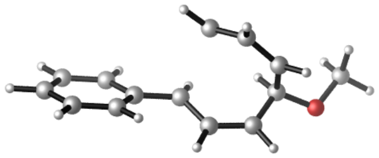

(TS$_{3i'-3i}$)

E (opt) = -618.289674726 Hartrees
G (opt) = -618.069034 Hartrees
E (SP) = -618.448499908 Hartrees

C	2.51454500	-0.76754500	0.85647200
C	2.62427700	0.15141600	-0.32676300
C	1.73523100	-0.78170200	-1.08088900
C	0.34954000	-0.75458100	-1.05700600
H	2.25232600	-1.60017900	-1.57362100
C	-0.41405800	0.23227900	-0.41451300
H	-0.16043100	-1.63706100	-1.44018300
C	-1.87504500	0.16757500	-0.27526100
H	-0.00526100	1.23565500	-0.39053600
C	-2.58224400	-1.05190400	-0.24189500
C	-2.61355300	1.36125700	-0.14938200
C	-3.96997400	-1.07088400	-0.11393900
C	-4.68897000	0.12398900	-0.00246400
C	-4.00133900	1.34106800	-0.01648800
H	-2.08643500	2.31242700	-0.16276000
H	-2.03877400	-1.99052000	-0.29788000
H	-4.49316900	-2.02325300	-0.09133700
H	-5.77004300	0.10537400	0.10227500
H	-4.54714100	2.27644500	0.07445500
C	1.47455700	-0.72758900	1.78100300
C	0.49322500	0.25766600	1.80311700
H	3.21830500	-1.59568900	0.85833300
H	1.32358000	-1.61895900	2.38949200
O	3.90655800	0.22209600	-0.91435500
C	4.82581400	0.96652800	-0.12626300
H	5.78045800	0.96474200	-0.65892800
H	4.49594100	2.00855700	0.00686100
H	4.97170500	0.52114700	0.86867000
H	2.20794200	1.15332900	-0.16115800
H	-0.35965300	0.15296700	2.46720600
H	0.72615200	1.27282600	1.50957700

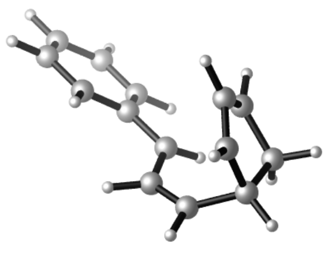

($TS_{3r'-3r}$)

E (opt) = -541.867811533 Hartrees
G (opt) = -541.668666 Hartrees
E (SP) = -541.998098210 Hartrees

C	2.96646000	-0.77657700	0.80097200
C	3.18407500	0.06646000	-0.43273000
C	2.27006300	-0.91225600	-1.13045100
C	0.88527800	-0.86133500	-1.16139100
H	2.76117900	-1.80876000	-1.50008000
C	0.10363300	0.23076000	-0.73073800
H	0.37136800	-1.80015200	-1.37305100
C	-1.30812600	0.08654600	-0.36410200
H	0.38485600	1.23004000	-1.02856500
C	-1.83766100	-1.12529900	0.12851000
C	-2.18048100	1.19094400	-0.46654600
C	-3.18517500	-1.23348000	0.46987900
C	-4.03798500	-0.13172000	0.34827800
C	-3.52541600	1.08373700	-0.11798800
H	-1.79151200	2.13863000	-0.83223000
H	-1.18136500	-1.98053000	0.26143500
H	-3.56950200	-2.17855800	0.84483500
H	-5.08594700	-0.21641300	0.62163100
H	-4.17706200	1.94850800	-0.21197700
H	4.19217200	0.11123900	-0.84513100
C	1.98654000	-0.18055300	1.59826200
C	2.57909700	1.42224900	-0.02808400
C	1.54844100	1.00441800	1.01212400
H	2.19080300	2.00113300	-0.86883700
H	3.35546100	2.03540600	0.45289200
H	3.47430900	-1.71350100	0.99316000
H	0.85016700	1.69879700	1.46449900
H	1.53262300	-0.64921400	2.46545000

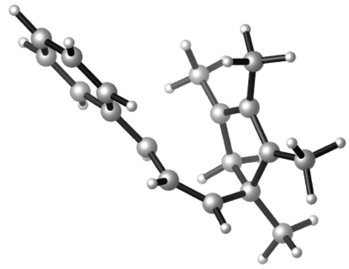

($TS_{3s'-3s}$)

E (opt) = -699.168069245 Hartrees
G (opt) = -698.865737 Hartrees
E (SP) = -699.330441920 Hartrees

C	-2.37378100	0.71625500	0.23407400
C	-2.54760700	-0.58235300	-0.53069200
C	-1.69207400	0.01837900	-1.63399800
C	-0.31024400	0.05939500	-1.69556600
H	-2.24933800	0.63778800	-2.33368600
C	0.56718800	-0.67110000	-0.86796500
H	0.12140900	0.83490900	-2.33156900
C	1.94776900	-0.27043400	-0.62059300
H	0.36009500	-1.71137700	-0.66552700
C	2.38804300	1.06612300	-0.76079000
C	2.89193400	-1.22225300	-0.17243200
C	3.70944600	1.42072500	-0.49623200
C	4.63074800	0.46085900	-0.06251100
C	4.21007100	-0.86308400	0.10217600
H	2.57907500	-2.25661100	-0.05103000
H	1.67997500	1.83311000	-1.06088300
H	4.02007800	2.45587900	-0.61432000
H	5.65781500	0.74224000	0.15218400
H	4.91344200	-1.61882800	0.44258900
C	-1.32647000	0.57380500	1.15996500
C	-1.83279700	-1.59340200	0.38482400
C	-0.83146300	-0.73374400	1.13636000
H	-1.40155100	-2.44304800	-0.15057900
H	-2.56035300	-2.00692200	1.10161700
C	0.14677500	-1.33125500	2.09752100
H	0.63001800	-2.21661400	1.67169200
H	0.93238300	-0.62564200	2.38098700
H	-0.36312000	-1.65484900	3.01737300
C	-0.72340300	1.72298800	1.91677500
H	-0.03867900	2.29472000	1.27459200
H	-1.49194700	2.42018300	2.26660000
H	-0.15455900	1.38728000	2.78795800
C	-3.17302900	1.96657500	0.01880700
H	-3.82713600	1.88709400	-0.85286600
H	-3.81281200	2.16848200	0.88920100
H	-2.53454900	2.84722900	-0.11646900
C	-3.94182900	-1.00615600	-0.96934900
H	-3.88843200	-1.92023300	-1.57186200
H	-4.57878900	-1.20552500	-0.10010500
H	-4.42867900	-0.23720900	-1.57814700

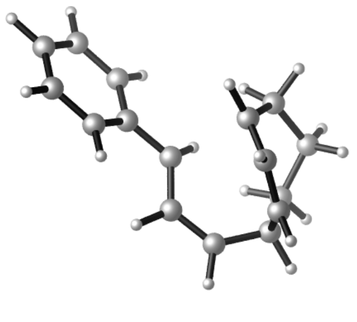

(TS$_{3t'-3t}$)

E (opt) = -620.508567559 Hartrees
G (opt) = -620.252744 Hartrees
E (SP) = -620.655300172 Hartrees

C	1.67323100	-0.45440700	1.68779900
C	0.87683300	0.63441600	1.32231000
C	2.64653100	-1.04846700	0.89098300
C	2.89208400	-0.76763400	-0.57054800
C	2.85933900	0.67832300	-1.12537600
C	1.39750400	1.88657300	0.64866900
H	1.49142100	2.64818100	1.43814800
H	0.64430000	2.29192300	-0.04066200
C	2.75585600	1.77307300	-0.05612100
H	2.05781300	0.78677100	-1.86310600
H	3.78975900	0.83764000	-1.68105600
H	3.53360600	1.59711300	0.69755400
H	2.98373400	2.74283900	-0.51478100
H	1.37261100	-1.01741300	2.57234400
H	0.00370700	0.81784300	1.94682600
C	1.77303900	-1.74937700	-0.84448400
H	3.17950300	-1.90484100	1.29588400
H	3.83533300	-1.23144600	-0.86513100
C	0.40422900	-1.52219800	-0.81241600
H	2.09680400	-2.77957000	-0.97165100
C	-0.21947400	-0.28287200	-0.59561700
H	-0.22258000	-2.41429700	-0.80498000
C	-1.65860700	-0.13905000	-0.34671500
H	0.24565800	0.59392000	-1.01677300
C	-2.42394200	-1.14398800	0.28164300
C	-2.31396400	1.05743700	-0.70526500
C	-3.78691200	-0.96770900	0.51417300
C	-4.42248900	0.22239000	0.14366500
C	-3.67560900	1.23616600	-0.46410700
H	-1.74324000	1.84708900	-1.18809000
H	-4.15618100	2.16680700	-0.75449400
H	-4.35480500	-1.75768200	0.99888000
H	-5.48340600	0.35953000	0.33270600
H	-1.93993800	-2.06047100	0.60683100

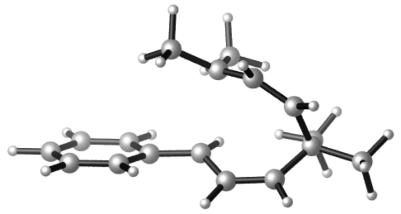

(TS$_{3w'-3w}$)

E (opt) = -661.036834620 Hartrees
G (opt) = -660.737640 Hartrees
E (SP) = -661.193385128 Hartrees

C	-2.63085200	0.43570100	-0.92883400
C	-2.97062500	-0.52235200	0.20013800
C	-1.98047500	-1.51867200	-0.37930500
C	-0.59666000	-1.51487400	-0.31320800
H	-2.42725200	-2.26012200	-1.03893900
C	0.22531700	-0.59988200	0.36823800
H	-0.10167900	-2.22739700	-0.97331200
C	1.68396700	-0.59306000	0.25625200
H	-0.15108100	-0.15857700	1.27763500
C	2.36089800	-1.02328400	-0.90560000
C	2.46350400	-0.08144100	1.31571700
C	3.75084600	-0.97144000	-0.98794400
C	4.50800100	-0.47116100	0.07727900
C	3.85425400	-0.02150500	1.22818700
H	1.96490600	0.25917200	2.21981100
H	1.78923500	-1.38055100	-1.75709500
H	4.24684800	-1.31064100	-1.89370600
H	5.59109000	-0.42595300	0.00721500
H	4.42924000	0.37269000	2.06206400
C	-1.52077700	1.22139400	-1.21345800
C	-0.41886100	1.62627300	-0.43653300
H	-3.28841500	0.29011500	-1.78447700
H	-1.40012200	1.40106600	-2.28509700
C	0.79497100	2.12915200	-1.18494200
H	1.70182900	2.00572600	-0.58409000
H	0.94569500	1.60619100	-2.13356400
H	0.69722400	3.20395100	-1.39923300
C	-0.54546500	2.24445300	0.93580700
H	-0.50881900	3.33758900	0.81797300
H	-1.48267800	2.00897400	1.43446900
H	0.29112700	1.97894400	1.59270400
C	-4.41160500	-1.03612400	0.01197700
H	-4.59670400	-1.90186400	0.65673900
H	-5.13350500	-0.25417700	0.27202000
H	-4.60194900	-1.34115800	-1.02275300
C	-2.82318200	-0.14171200	1.67856300
H	-1.81324400	0.11327400	1.98398300
H	-3.47553000	0.70533800	1.91800900
H	-3.13575700	-0.99138400	2.29564100

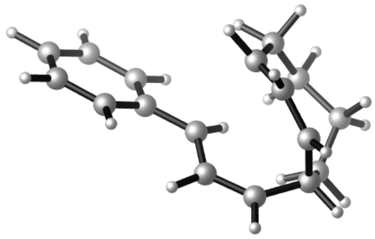

(TS$_{3x'-3x}$)

E (opt) = -659.810856802 Hartrees
G (opt) = -659.528222 Hartrees
E (SP) = -659.965996547 Hartrees

C	1.57702800	-0.66706400	1.76261400
C	0.67705600	0.37601000	1.55381700
C	2.53502500	-1.24835800	0.92947800
C	2.78352900	-1.08574100	-0.54412800
C	2.85269500	0.26227700	-1.29647700
C	0.87126500	1.81204500	1.09683600
C	1.67311500	2.25172400	-0.14060800
H	1.32222300	2.30385900	1.97394100
H	-0.12844800	2.25685100	1.01158200
C	2.99908900	1.54138800	-0.44956000
H	1.87033400	3.32134200	0.00325100
H	1.04091400	2.19924000	-1.03641500
H	1.99025000	0.36619400	-1.96311400
H	3.71898300	0.19284700	-1.96292100
H	3.54613000	1.32455300	0.47667400
H	3.62351700	2.24365800	-1.01398800
H	1.34358000	-1.28672900	2.63141700
H	-0.14673400	0.35377500	2.26928900
C	1.61402400	-2.03093700	-0.71055000
H	3.08118600	-2.08117700	1.36639800
H	3.69280400	-1.64214500	-0.77858000
C	0.24927500	-1.74115700	-0.68724200
H	1.88415800	-3.08103300	-0.78771900
C	-0.32914800	-0.48217700	-0.51406600
H	-0.40807300	-2.60966400	-0.65883600
C	-1.76663200	-0.24669700	-0.34155200
H	0.22548600	0.37580700	-0.85509300
C	-2.65286000	-1.22847400	0.15017300
C	-2.30031300	1.02326400	-0.64706600
C	-4.01079000	-0.95641700	0.30516700
C	-4.52376200	0.30692300	-0.00964900
C	-3.65777900	1.29684400	-0.48321100
H	-1.63650800	1.79686200	-1.02501500
H	-4.04094600	2.28374800	-0.72944900
H	-4.67225500	-1.73081500	0.68509800
H	-5.58162300	0.51757100	0.11911400
H	-2.26988800	-2.20546200	0.43050600

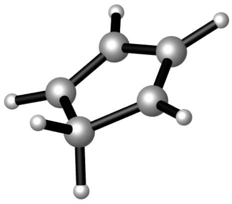

Cp

E (opt) = -194.119230101 Hartrees
G (opt) = -194.053421 Hartrees
E (SP) = -194.16950150 Hartrees

C	1.19716500	0.20374900	0.00007200
H	2.24910300	0.46533100	0.00013800
C	0.66741600	-1.03899200	0.00014600
C	-0.79956600	-0.94115600	-0.00013100
H	-1.46972700	-1.79489900	-0.00040800
C	-1.15937800	0.36087700	0.00013700
H	-2.16734200	0.75970400	0.00055900
H	1.21820200	-1.97418500	-0.00009400
C	0.08096600	1.21358000	-0.00008200
H	0.12496500	1.87838900	0.87582300
H	0.12518000	1.87731200	-0.87687300

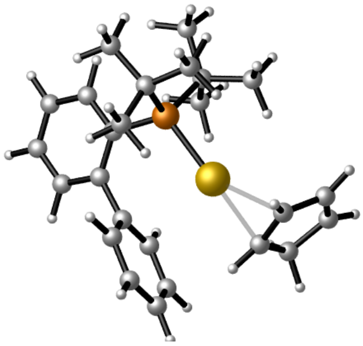

Cp-Au complex

E (opt) = -1,449.37610488 Hartrees
G (opt) = -1,448.920289 Hartrees
E (SP) = -1449.63082645 Hartrees

C	-3.33528700	0.26502500	0.18394700
H	-3.27635700	1.16418400	0.78791200
C	-3.43030100	-1.03079300	0.67197500
C	-3.88797600	-1.92358100	-0.39610100
H	-4.01400700	-2.99351000	-0.28115100
C	-4.12453200	-1.18960500	-1.50691700
H	-4.46749400	-1.56583100	-2.46389100
H	-3.32188300	-1.32390200	1.71167800
C	-3.82142300	0.25661600	-1.24916100
H	-4.72331700	0.87983500	-1.34041300
H	-3.09855300	0.67009700	-1.96506100
Au	-1.08555400	-0.32264800	0.22878600
P	1.20856500	-0.81050600	0.06707400
C	1.75888400	-1.55143500	1.73103900
C	1.39796500	-1.99068100	-1.43129000
C	2.29094000	0.65065000	-0.25057800
C	0.97892600	-2.85855600	1.96361900
C	3.27110900	-1.81652500	1.84522600
C	1.36603500	-0.51822100	2.80754700
C	0.17474400	-2.93384200	-1.47682900
C	1.37289800	-1.09889800	-2.68895800
C	2.66868300	-2.85915100	-1.41736300
C	1.81694700	1.98439600	-0.30240200
C	3.65304400	0.41099600	-0.52535900
H	-0.10233200	-2.71169700	1.86926200

H	1.28565300	-3.64561200	1.27015800
H	1.18222900	-3.21322700	2.98054700
H	3.85069400	-0.89212100	1.79178800
H	3.46404500	-2.26508500	2.82684000
H	3.64425200	-2.50969400	1.08978100
H	0.28828400	-0.32757100	2.82208400
H	1.65542100	-0.90821600	3.79033700
H	1.88555200	0.43354200	2.66110300
H	0.30236300	-3.62434100	-2.31862200
H	0.06926500	-3.53284700	-0.56981300
H	-0.75852600	-2.38555600	-1.63513800
H	0.49988300	-0.43578800	-2.69623900
H	2.27244300	-0.48523100	-2.77961100
H	1.31005200	-1.74159300	-3.57440300
H	2.67247400	-3.56034000	-0.57900900
H	2.68862300	-3.45117800	-2.33992700
H	3.58997300	-2.27563900	-1.39259200
C	2.71557200	3.00836700	-0.65226500
C	0.42228900	2.43727600	-0.00311500
C	4.52839400	1.44030700	-0.85982500
H	4.04413700	-0.59629800	-0.48507600
C	4.05454600	2.74970100	-0.93128500
H	2.34348400	4.02753900	-0.69504000
C	0.00308500	2.63725100	1.32248500
C	-0.43520900	2.81205200	-1.05062000
H	5.57004200	1.21497100	-1.06721300
H	4.72043600	3.56506400	-1.19798500
C	-1.24836100	3.19569800	1.59301000
H	0.66589600	2.36914900	2.13865000
C	-1.68754800	3.36774800	-0.77788300
H	-0.11064000	2.67463500	-2.07802700
C	-2.09442200	3.56701300	0.54414300
H	-1.55479500	3.35409800	2.62313400
H	-2.33876400	3.65566800	-1.59822400
H	-3.06227500	4.01241200	0.75569400

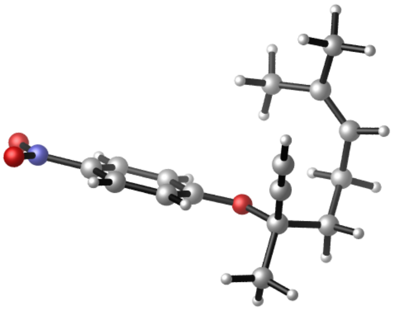

Enyne (6a)

E (opt) = -901.45214409 Hartrees
G (opt) = -901.182515 Hartrees
E (SP) = -901.69537630 Hartrees

C	1.67295600	-1.60824800	-0.04874700
C	1.23190200	-3.08030400	0.02745400
H	1.05072700	-3.45202700	-0.98488700
H	2.02644300	-3.67534700	0.48583000
H	0.32362400	-3.20876900	0.61810000
O	0.71483200	-0.84604900	-0.84205600
C	2.98673900	-1.50466200	-0.86638500
H	3.78889300	-1.91882900	-0.24633400
H	2.87406800	-2.16589200	-1.73258700
C	3.36589100	-0.09769600	-1.37621400
H	4.26347700	-0.22709100	-1.99649600
H	2.57685200	0.25625600	-2.04295000
C	3.66008900	0.90286700	-0.29238100
H	4.56768400	0.69969200	0.27884600
C	2.92561400	1.96589900	0.07180800

C	1.61469300	2.36692800	-0.55740000
H	1.34013800	1.75649600	-1.41843100
H	0.80391900	2.28259600	0.17873700
H	1.63912700	3.41847700	-0.87282800
C	3.34281000	2.82793800	1.23796000
H	2.58700400	2.78611500	2.03507000
H	4.30083300	2.51336500	1.66328100
H	3.42603800	3.88368000	0.94648700
C	1.83514400	-1.05019100	1.30659000
C	1.96855300	-0.67258900	2.44681100
H	2.09614900	-0.30796100	3.44335800
C	-0.56458800	-0.58397100	-0.49282300
C	-1.30021900	0.09037900	-1.48987500
C	-1.18016700	-0.90269800	0.73141400
C	-2.62160400	0.43850100	-1.27767700
H	-0.80493900	0.32746100	-2.42502700
C	-2.50882700	-0.55414300	0.94350800
H	-0.63423100	-1.39885500	1.52022000
C	-3.22176200	0.11018900	-0.05568500
H	-3.19091200	0.95581100	-2.03981600
H	-2.99407100	-0.79279800	1.88173200
N	-4.61012300	0.47024800	0.17795800
O	-5.12511700	0.15698200	1.25762800
O	-5.21637700	1.07490400	-0.71409300

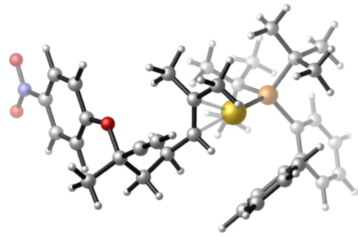

VIII (6a-Au alkene complex)

E (opt) = -2156.71356883 Hartrees
G (opt) = -2156.048983 Hartrees
E (SP) = -2157.16341302 Hartrees

C	-3.89681300	-2.61665600	-0.19877000
C	-5.13685100	-3.25455100	-0.84624000
H	-5.56460700	-2.60112900	-1.60843400
H	-4.85211400	-4.19681300	-1.32174800
H	-5.89437400	-3.45832500	-0.08580300
O	-4.26743300	-1.42348300	0.56479300
C	-2.78300900	-2.33860900	-1.24288900
H	-2.30843400	-3.30030600	-1.46357800
H	-3.24107200	-2.00399500	-2.17711200
C	-1.71079000	-1.30854500	-0.83485200
H	-1.03947100	-1.20745800	-1.69360300
H	-2.16960400	-0.33061500	-0.67638200
C	-0.90409800	-1.74241500	0.36771000
H	-0.39705300	-2.69899900	0.24545200
C	-0.99574600	-1.25849500	1.66104600
C	-1.86299100	-0.09894500	2.07706200
H	-2.18723200	0.52285300	1.24323400
H	-2.75641800	-0.49833100	2.57037500
H	-1.33677400	0.52977500	2.80188100
C	-0.41672100	-2.05707000	2.80352500
H	-1.24634600	-2.54836900	3.32890700
H	0.27796400	-2.82711400	2.45956600
H	0.09148400	-1.41375800	3.52793800
C	-3.39750700	-3.52184600	0.85339800
C	-3.01433600	-4.30759100	1.68663800
H	-2.67320600	-5.00054900	2.42551500
C	-4.85370300	-0.30801500	0.07154000
C	-5.28762600	0.58926400	1.07053600
C	-5.02364200	0.02946300	-1.28336900
C	-5.87068700	1.79663600	0.73186400
H	-5.15443500	0.30872000	2.10918000
C	-5.61438700	1.24142900	-1.62432800
H	-4.70362900	-0.63111400	-2.07474200
C	-6.03221600	2.11620300	-0.62155800
H	-6.20494400	2.48778500	1.49543500
H	-5.74829200	1.51102100	-2.66464600
N	-6.64567200	3.38287000	-0.98809400
O	-6.98597800	4.15083400	-0.08156900
O	-6.79738500	3.63538300	-2.18839200
Au	0.93971200	-0.32708200	0.51676600
P	2.70193500	1.21983100	0.37238800
C	1.90377400	2.86713300	-0.19135100
C	3.54150600	1.29992900	2.07891000
C	4.01802800	0.80266500	-0.85409800
C	0.52646200	3.01604900	0.49420300
C	2.73440800	4.12444200	0.11807000
C	1.67671400	2.73809600	-1.71108100
C	2.52120000	1.82847700	3.10439700
C	3.92194000	-0.15269800	2.43613800
C	4.81755800	2.15989100	2.12424600
C	4.04181400	-0.39922600	-1.60383000
C	5.01912400	1.76479200	-1.09764300
H	0.08285600	3.96402000	0.16889400
H	-0.16032300	2.21246900	0.21175000
H	0.59487700	3.03771900	1.58339100
H	2.18765800	4.99857000	-0.25463300
H	2.88102900	4.26321100	1.19172000
H	3.70835600	4.12399500	-0.37347700
H	2.61486400	2.73789500	-2.27177600
H	1.12412200	1.82494600	-1.96088500
H	1.08068700	3.59247800	-2.05154300
H	1.57671200	1.27565000	3.06374200
H	2.93874700	1.70500700	4.11025400
H	2.31075100	2.89163800	2.96230200
H	3.04322900	-0.79876000	2.52767900
H	4.59968000	-0.58979800	1.69651100
H	4.44088200	-0.15129300	3.40167500
H	4.64550900	3.19518300	1.82488200
H	5.18329500	2.17160600	3.15757900
H	5.61228400	1.74161100	1.50234000
C	5.04907600	-0.57449500	-2.56966600
C	3.08345500	-1.54040400	-1.46671600
C	6.01327700	1.56798600	-2.05192500
H	5.02615200	2.68990000	-0.53925700
C	6.02599600	0.39041200	-2.79825100
H	5.05839300	-1.49699300	-3.14214500
C	2.09205300	-1.74249300	-2.44035000
C	3.24100900	-2.49540500	-0.44905500
H	6.76680800	2.33349600	-2.20977100
H	6.79062900	0.22168900	-3.55070300
C	1.26858200	-2.86995600	-2.39139800
H	1.97621400	-1.01677700	-3.24010700
C	2.42105300	-3.62550700	-0.40553100
H	4.01566700	-2.35640600	0.29843500
C	1.43431400	-3.81682700	-1.37714200
H	0.50629200	-3.01285200	-3.15183100
H	2.55974500	-4.36075200	0.38202800
H	0.80189800	-4.69935900	-1.34624500

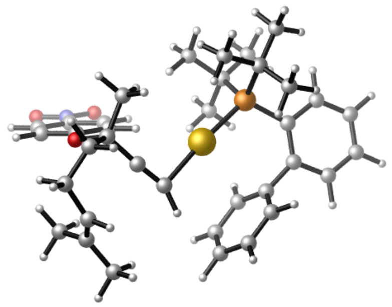

6a-Au alkyne complex

E (opt) = -2156.711238 Hartrees
G (opt) = -2156.04846 Hartrees
E (SP) = -2157.15758247 Hartrees

C	-2.73171800	-1.00426000	1.86939400
C	-1.91760900	-0.15739400	2.86296700
H	-2.61118200	0.27788400	3.58684300
H	-1.20363900	-0.79452300	3.38846300
H	-1.36905000	0.64403500	2.36900200
O	-3.82233100	-0.24154600	1.30056100
C	-3.44614900	-2.15331000	2.63589800
H	-2.67112000	-2.84041900	2.99104600
H	-3.90814100	-1.69572800	3.51684400
C	-4.52059000	-2.90946500	1.82887100
H	-4.95284500	-3.65775600	2.50558500
H	-5.32285100	-2.21519200	1.57262200
C	-3.96445700	-3.59151200	0.61040900
H	-3.30313500	-4.43286600	0.82351200
C	-4.13456000	-3.24992200	-0.67931500
C	-4.97880400	-2.10005700	-1.16766200
H	-5.52371600	-1.59117200	-0.37216200
H	-4.34837600	-1.35426000	-1.66983700
H	-5.70330100	-2.44784000	-1.91527200
C	-3.40677800	-3.99399900	-1.77075200
H	-2.72304700	-3.31717600	-2.30281700
H	-2.82153200	-4.83341000	-1.38292100
H	-4.10524000	-4.38155700	-2.52388800
C	-1.86160400	-1.57707900	0.82344600
C	-1.07043500	-2.03735200	-0.00468500
H	-0.80785800	-2.73522400	-0.78298700
C	-3.70754900	0.88378300	0.54861700
C	-4.93661500	1.52469100	0.29909200
C	-2.52285700	1.41200100	0.01032000
C	-4.98600500	2.67695700	-0.46612600
H	-5.83914800	1.09819000	0.72253200
C	-2.57149700	2.56978500	-0.75792900
H	-1.56430300	0.94232000	0.17365400
C	-3.79577500	3.19499300	-0.98946800
H	-5.92640900	3.17828400	-0.65832000
H	-1.66471200	2.98856300	-1.17470100
N	-3.83355500	4.41246900	-1.78717300
O	-2.76940100	4.84858300	-2.23951000
O	-4.92764400	4.95381200	-1.97404900
Au	0.58748800	-0.63318400	0.38581600
P	2.45114600	0.76100000	0.67928200
C	1.92078000	2.55278200	0.30564900
C	3.07182700	0.46295800	2.46587100
C	3.84974900	0.36526900	-0.45772100
C	0.85346900	2.97212700	1.33257100
C	3.07418300	3.57180300	0.30860300
C	1.29763000	2.52916600	-1.10625700
C	1.84636200	0.27142300	3.38792500
C	3.86035700	-0.86101900	2.43021700
C	3.94418200	1.58739400	3.04822900
C	3.78861300	-0.61450300	-1.48000900
C	5.07328700	1.03133500	-0.23936100
H	0.02456500	2.25952200	1.37999000
H	1.27325900	3.08722900	2.33506900
H	0.43973000	3.94178300	1.03317200
H	3.80033400	3.36420000	-0.48051500
H	2.65133100	4.56361600	0.11060100
H	3.59862000	3.62435900	1.26403400
H	0.44693100	1.84438700	-1.17256100
H	0.94473000	3.53733100	-1.35192500
H	2.03003000	2.24043200	-1.86592300
H	2.20523000	0.15582400	4.41716100
H	1.15977800	1.12037800	3.37137200
H	1.28543700	-0.63125500	3.13023300
H	3.27810300	-1.66430500	1.96394000
H	4.80723000	-0.76481200	1.89372100
H	4.08360400	-1.16342200	3.45960700
H	3.38831100	2.52218100	3.15217500
H	4.26707000	1.28430800	4.05116100
H	4.84751500	1.77606700	2.46616800
C	4.95472400	-0.89566600	-2.21550900
C	2.58327100	-1.40287700	-1.88979100
C	6.21229500	0.74556500	-0.98655700
H	5.15009700	1.78327300	0.53321800
C	6.15445300	-0.23085400	-1.97978600
H	4.90217700	-1.64987800	-2.99487300
C	1.58746700	-0.83188000	-2.70167700
C	2.52050300	-2.77753000	-1.60615400
H	7.13555700	1.28078300	-0.78676400
H	7.03372600	-0.47283300	-2.56943000
C	0.55494400	-1.61894000	-3.21660100
H	1.63806500	0.22435600	-2.94293000
C	1.48677900	-3.56323200	-2.12152900
H	3.29694600	-3.22973800	-0.99586600
C	0.50563500	-2.98775500	-2.93480100
H	-0.20167400	-1.16573800	-3.85066600
H	1.45711100	-4.62628000	-1.90002100
H	-0.28810300	-3.60199600	-3. 35019400

T

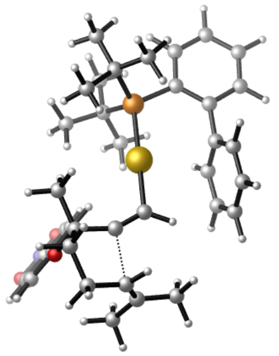

TS **(5-exo-dig)**

E (opt) = -2156.709409 Hartrees
G (opt) = -2156.044649 Hartrees
E (SP) = -2157.15389655 Hartrees

C	-2.86594900	-1.18976300	1.83807400
C	-1.92736200	-0.43599300	2.79887900
H	-2.53349100	-0.04589100	3.62075100
H	-1.17821600	-1.12106800	3.19905700
H	-1.41506400	0.39318000	2.31381800
O	-3.95015100	-0.33215500	1.39901600
C	-3.58682300	-2.32218800	2.59330100
H	-2.81955000	-2.98859700	2.99985100
H	-4.13870200	-1.88695700	3.43127200
C	-4.52221900	-3.08922700	1.64166800
H	-4.85936400	-4.00727100	2.13514700
H	-5.40616800	-2.48485300	1.43245900
C	-3.77343800	-3.42999000	0.38645100
H	-3.05082600	-4.23952300	0.47806000
C	-3.96571300	-2.90479800	-0.85951300
C	-4.97919100	-1.85184500	-1.20486600
H	-5.55311900	-1.49728700	-0.35036200
H	-4.48354100	-0.98992300	-1.66652000
H	-5.67412900	-2.24997900	-1.95487100
C	-3.14534900	-3.39394900	-2.01758400
H	-2.62089900	-2.55780400	-2.49666900
H	-2.41460500	-4.15167100	-1.72454100
H	-3.80155200	-3.82867600	-2.78217300
C	-2.10994000	-1.77668800	0.70087500
C	-1.05236400	-1.95830900	0.01920300
H	-0.86899200	-2.65571900	-0.79089500
C	-3.82144500	0.77576600	0.62532000
C	-5.03654200	1.45197700	0.39881300
C	-2.63447600	1.26415800	0.05357100
C	-5.07090900	2.59793800	-0.37640100
H	-5.94124600	1.05468800	0.84558700
C	-2.66878800	2.41573500	-0.72469600

H	-1.68688700	0.76916000	0.19910900
C	-3.87897600	3.07553300	-0.93372300
H	-6.00082600	3.12428300	-0.55210200
H	-1.75882300	2.80262600	-1.16476600
N	-3.90011400	4.28554000	-1.74235300
O	-2.83756800	4.67745100	-2.23743200
O	-4.97957500	4.86581400	-1.89657200
Au	0.60838900	-0.69126800	0.36917100
P	2.43292200	0.77053100	0.70242000
C	1.82414700	2.54404000	0.35622400
C	3.05656200	0.47486500	2.48834900
C	3.87158700	0.47984800	-0.42035300
C	0.71864100	2.88797300	1.37163000
C	2.91730200	3.62688500	0.39751200
C	1.22683900	2.51496000	-1.06734400
C	1.83345600	0.21503800	3.39615200
C	3.90091900	-0.81453900	2.43800700
C	3.87630600	1.62310300	3.10036900
C	3.87636000	-0.48641900	-1.45729400
C	5.05445000	1.20870500	-0.18071500
H	-0.06633800	2.12595100	1.40111400
H	1.11807200	3.01698800	2.38109600
H	0.25142300	3.83485700	1.07760200
H	3.66071800	3.48609100	-0.39058500
H	2.44107500	4.59944200	0.22572900
H	3.43186200	3.67988200	1.35858600
H	0.40898300	1.79404500	-1.15737900
H	0.83335000	3.50981700	-1.30643000
H	1.98433600	2.26834200	-1.81752800
H	2.18758900	0.09003000	4.42618700
H	1.11565800	1.03787400	3.39086000
H	1.30854900	-0.70120100	3.11185900
H	3.35516000	-1.63446800	1.95681600
H	4.84531200	-0.67031200	1.90769000
H	4.13237400	-1.12271100	3.46400600
H	3.27814300	2.52977300	3.22140200
H	4.20904900	1.31400200	4.09838400
H	4.77210200	1.86665100	2.52680300
C	5.06383400	-0.69824700	-2.18089400
C	2.71391900	-1.32626500	-1.88619600
C	6.21663600	0.99158800	-0.91582200
H	5.08030900	1.95788800	0.59840000
C	6.22456500	0.02424100	-1.91977600
H	5.05954000	-1.44482400	-2.96952400
C	1.71014200	-0.79877700	-2.71554900
C	2.68682400	-2.69544700	-1.57556700
H	7.10773700	1.57257600	-0.69810200
H	7.12396200	-0.16501500	-2.49836600
C	0.70137600	-1.62328400	-3.21946800
H	1.73018200	0.25459600	-2.97433200
C	1.67340800	-3.51750000	-2.07463400
H	3.46933400	-3.11318100	-0.94862100
C	0.68232500	-2.98501100	-2.90406100
H	-0.06256700	-1.20356500	-3.86790700
H	1.66792100	-4.57539900	-1.82766900
H	-0.09593100	-3.62697800	-3.30640700

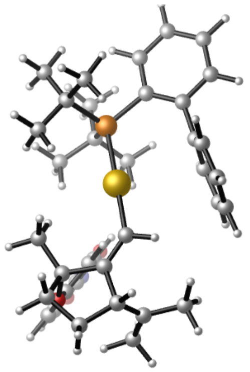

Int (5-exo-dig)

E (opt) = -2156.72183 Hartrees
G (opt) = -2156.052651 Hartrees
E (SP) = -2157.16475590 Hartrees

C	-2.99730400	-1.19166000	1.74001000
C	-2.06040000	-0.36546000	2.61224500
H	-2.65178500	0.11310800	3.39875800
H	-1.31347000	-1.01258900	3.07481300
H	-1.53732800	0.40756700	2.04985800
O	-4.05576800	-0.33027200	1.19840600
C	-3.78997500	-2.22158800	2.55812300
H	-3.08555900	-2.74935200	3.20918600
H	-4.53859300	-1.73210000	3.18551000
C	-4.39448000	-3.18712700	1.52888200
H	-4.60382300	-4.17166800	1.95240300
H	-5.33317900	-2.79238600	1.13629300
C	-3.33281500	-3.31146200	0.42089600
H	-2.76624700	-4.24097100	0.45606300
C	-3.60321900	-2.87863900	-0.93222100
C	-4.63559400	-1.88357600	-1.31280800
H	-5.24543300	-1.52853100	-0.48776100
H	-4.16270900	-1.03016300	-1.81238800
H	-5.27313400	-2.35731600	-2.07159300
C	-2.86309600	-3.50626000	-2.05566600
H	-2.67444100	-2.80929300	-2.87573000
H	-1.94837700	-4.01301100	-1.74845400
H	-3.54706200	-4.28200200	-2.43795900
C	-2.32467600	-2.06995800	0.66628700
C	-1.07544500	-2.03474900	0.16964600
H	-0.84719800	-2.82630900	-0.54404100
C	-3.84547300	0.78730100	0.46324000
C	-4.93480700	1.68036800	0.42979400
C	-2.68486400	1.07553300	-0.27498600
C	-4.86158600	2.85245800	-0.30404000
H	-5.82423500	1.43496100	0.99986300
C	-2.60772800	2.25406600	-1.00621500
H	-1.84741200	0.39431400	-0.28064000
C	-3.68860100	3.13568600	-1.01375600
H	-5.68957500	3.55017600	-0.32552600
H	-1.71531700	2.49112600	-1.56965200
N	-3.58709300	4.37592300	-1.76712600
O	-2.52357600	4.62342600	-2.34744800
O	-4.56683900	5.12819500	-1.79042000
Au	0.51587000	-0.75811900	0.42651300
P	2.31357300	0.80458900	0.69092800
C	1.68580200	2.52720200	0.16661200
C	2.86376500	0.71672500	2.52816400
C	3.82680600	0.49433600	-0.33142800
C	0.50953000	2.91438100	1.08205600
C	2.74191200	3.64596900	0.18110200
C	1.17551000	2.36886400	-1.28112100
C	1.60994700	0.45321300	3.39368200
C	3.79079800	-0.51080800	2.64150000
C	3.56874000	1.96730200	3.08199700
C	3.90409600	-0.51363400	-1.32445400
C	4.97969600	1.26270200	-0.07265800
H	-0.26436500	2.14178500	1.10298500
H	0.83686000	3.10979400	2.10637700
H	0.05001300	3.83356400	0.70014600
H	3.54733800	3.45608900	-0.53225800
H	2.25528300	4.58174900	-0.11928000
H	3.18024000	3.80571900	1.16707400
H	0.40583000	1.59604200	-1.36309900
H	0.74097700	3.32003700	-1.61075700
H	1.98921100	2.11814000	-1.96836300
H	1.91411300	0.42493600	4.44682800
H	0.85017800	1.23156500	3.29037000
H	1.14856600	-0.50845500	3.15263200
H	3.33117300	-1.40455700	2.20286200
H	4.75592900	-0.34833500	2.15539700
H	3.97806900	-0.71771700	3.70165200
H	2.90601200	2.83627100	3.09233400

H	3.86042500	1.76660700	4.12001200
H	4.47954300	2.22959000	2.54089200
C	5.12500900	-0.71902500	-1.99233300
C	2.78351300	-1.40819400	-1.75238300
C	6.17797200	1.04966500	-0.74895400
H	4.95030700	2.04068600	0.67764700
C	6.25428900	0.04561600	-1.71347600
H	5.17331300	-1.49671600	-2.74895100
C	1.80983300	-0.96340600	-2.65978800
C	2.76485600	-2.75048400	-1.34237700
H	7.04401200	1.66212500	-0.51637900
H	7.18164800	-0.13919800	-2.24778800
C	0.83044700	-1.83884400	-3.13527300
H	1.82572100	0.06672200	-2.99850700
C	1.78444000	-3.62512800	-1.81687700
H	3.52614100	-3.10654200	-0.65412900
C	0.81701700	-3.17255800	-2.71777200
H	0.08765900	-1.48057200	-3.84271300
H	1.78481100	-4.66169000	-1.49150600
H	0.06415200	-3.85581900	-3.09924300

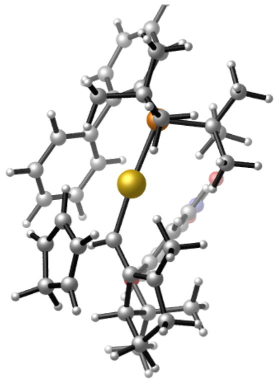

IXa

E (opt) = -2350.88232379 Hartrees
G (opt) = -2350.125518 Hartrees
E (SP) = -2351.37058260 Hartrees

Au	0.16992800	-0.99308400	0.01467900
P	2.45943100	-1.04041100	-0.68806200
C	-1.80318400	-0.85794500	0.46751300
H	-2.02217000	-0.32794400	1.39353100
C	-2.29369400	-2.83906500	2.30228900
H	-1.95019700	-2.15540900	3.06763700
C	-1.51289100	-3.64410100	1.52614500
C	-2.94915600	-1.06314000	-0.32489300
C	-3.07160800	-1.90602900	-1.41548300
C	3.08033800	-2.83758100	-0.57675500
C	2.47853800	-0.32841300	-2.47051300
C	-2.36065600	-4.45213800	0.66326400
H	-1.99100400	-5.16457400	-0.06529300
C	-3.66124900	-4.15308500	0.90795100
H	-4.52893800	-4.59323100	0.43091000
C	-4.46754600	-1.86917800	-1.96576300
H	-4.43333500	-1.36582400	-2.94367200
H	-4.85077000	-2.87657500	-2.16051100
C	-4.25253500	-0.29818300	-0.08899900
H	-4.51195200	-0.29039100	0.97514000
C	-2.02903100	-2.72000900	-2.09262100
H	-1.11519100	-2.81417900	-1.50732100
H	-2.41349600	-3.71382800	-2.34043500
H	-1.78096200	-2.23241200	-3.04664200
C	-5.29261200	-1.08429300	-0.92359700
H	-5.81454300	-1.78794300	-0.26886700
H	-6.04988100	-0.44719100	-1.38525800
C	-4.09307300	1.19907300	-0.49226900
C	-3.76747000	1.39484700	-1.97271200
H	-4.60337200	1.04533500	-2.58358100
H	-3.61541100	2.45370400	-2.19848700
H	-2.86699300	0.84920300	-2.26528100
C	-5.32162400	2.01417100	-0.08712600
H	-5.50571800	1.91983700	0.98711200
H	-5.17199900	3.07143900	-0.32470000
H	-6.21088300	1.67382100	-0.62529900
O	-2.96438200	1.62966600	0.35373200
H	-0.43018100	-3.68289300	1.53976100
C	-3.73730900	-3.14760300	2.02096300
H	-4.21501900	-3.58817000	2.91005500
H	-4.33713800	-2.26654700	1.77024700
C	1.18774800	-0.78086700	-3.19266200
H	0.29242600	-0.37622000	-2.71228900
H	1.21533700	-0.40297000	-4.22168100
H	1.08746400	-1.86726800	-3.23999600
C	2.42685100	1.20554800	-2.31557900
H	1.61425000	1.51174600	-1.64776700
H	3.36216600	1.61424200	-1.92573800
H	2.24257300	1.65680900	-3.29760900
C	3.67862300	-0.73436800	-3.34250300
H	3.69673600	-1.80962600	-3.53663600
H	3.58867400	-0.22773800	-4.31089700
H	4.63887000	-0.43936600	-2.91691200
C	2.21942500	-3.70538500	-1.51374600
H	2.45763600	-4.76095400	-1.33691200
H	1.14832200	-3.56985100	-1.33046100
H	2.42037700	-3.49476500	-2.56731600
C	4.57029100	-3.04386600	-0.90136200
H	4.79139400	-4.11607200	-0.83413300
H	4.83869200	-2.71632700	-1.90688000
H	5.21672700	-2.53367800	-0.18329300
C	2.84311600	-3.27330400	0.88498800
H	3.44107100	-2.67913800	1.58256500
H	1.79147500	-3.19065700	1.17612000
H	3.14477600	-4.32154600	0.99658000
C	3.64918000	-0.01913200	0.29614100
C	3.28731700	0.67212000	1.47942600
C	4.96356600	0.13494300	-0.18966800
C	4.23925500	1.49952600	2.10177300
C	5.89594700	0.94956900	0.44727300
H	5.27151300	-0.38294500	-1.08724800
C	5.52924500	1.64454500	1.59909900
H	3.94967700	2.02953900	3.00439400
H	6.89794200	1.04212400	0.03916800
H	6.24040900	2.29110900	2.10484100
C	1.95565100	0.59957800	2.15738100
C	1.62556600	-0.48732900	2.98184900
C	1.05870900	1.67435800	2.05942500
C	0.41518600	-0.50594000	3.67842100
H	2.32060300	-1.31457000	3.07792500
C	-0.15276500	1.65371300	2.75405900
H	1.30811200	2.52253300	1.42925700
C	-0.47762700	0.56392600	3.56562500
H	0.17455200	-1.35282000	4.31486300
H	-0.83999200	2.48842900	2.65760900
H	-1.41676100	0.55133000	4.11147300
C	-2.23310500	2.74336800	0.05940600
C	-2.59405300	3.98299200	0.60851400
C	-1.05853600	2.62156400	-0.69982600
C	-1.78970600	5.09765800	0.39859500
H	-3.49430000	4.05529100	1.20775300
C	-0.25011200	3.72939100	-0.91446300
H	-0.77970600	1.64996400	-1.09047900
C	-0.62659200	4.95623600	-0.36168400
H	-2.04739300	6.06191800	0.81859800
H	0.66361100	3.65320000	-1.49027700
N	0.22890800	6.12156400	-0.57616600
O	-0.11017100	7.19490400	-0.07026100
O	1.25121500	5.97716300	-1.25303200

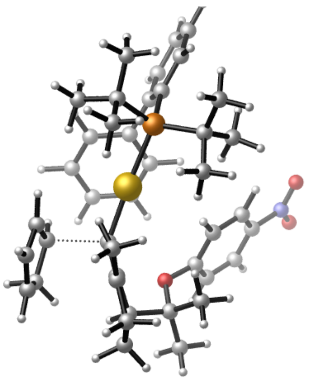

TS$_{IXa-Xa}$

E (opt) = -2350.87978591 Hartrees
G (opt) = -2350.121142 Hartrees
E (SP) = -2351.36584257 Hartrees

Au	0.01835700	-1.03021200	0.08084400
P	2.25703900	-1.26876300	-0.73372000
C	-1.95653900	-0.81329200	0.63893500
H	-2.09369300	-0.05353800	1.40639800
C	-2.37075900	-2.25255700	2.14850500
H	-1.72369000	-1.75706800	2.86109600
C	-2.02473900	-3.44692100	1.51366600
C	-3.08944700	-0.87670500	-0.24858600
C	-3.30932200	-1.77904200	-1.26047800
C	2.68523000	-3.12664600	-0.64574400
C	2.28550300	-0.56321600	-2.51956400
C	-3.18572200	-4.07449800	0.97816400
H	-3.18302800	-4.96594700	0.36331600
C	-4.28631100	-3.33325800	1.32321000
H	-5.31147600	-3.53787900	1.03738000
C	-4.62919300	-1.53565400	-1.94136000
H	-4.44310300	-1.13954100	-2.95027700
H	-5.19242400	-2.46548300	-2.08063800
C	-4.22922700	0.14519200	-0.19196800
H	-4.55304700	0.31770000	0.84066500
C	-2.37639100	-2.81090200	-1.79304100
H	-1.56890400	-3.04868400	-1.10124900
H	-2.90814900	-3.72716800	-2.06809900
H	-1.91494100	-2.42159200	-2.71195700
C	-5.34914800	-0.51777900	-1.03220100
H	-6.02783000	-1.04797000	-0.35729200
H	-5.95153000	0.19608900	-1.59803300
C	-3.78246300	1.54383800	-0.71230600
C	-3.25409900	1.51201800	-2.14587800
H	-4.05324500	1.20678000	-2.82586100
H	-2.91704100	2.50360600	-2.45934700
H	-2.41994800	0.81363800	-2.24912000
C	-4.90153500	2.57638500	-0.56331600
H	-5.26774000	2.59951200	0.46743900
H	-4.53434900	3.57283000	-0.82600200
H	-5.74002900	2.34819400	-1.22648900
O	-2.71191200	1.90281600	0.23556300
H	-1.01116200	-3.81343400	1.39913800
C	-3.88158300	-2.24229200	2.25857900
H	-4.14650900	-2.55348700	3.28294700
H	-4.37834700	-1.28471500	2.09878000
C	0.90266400	-0.82296000	-3.16161000
H	0.10547700	-0.28057100	-2.64448000
H	0.92747600	-0.47117800	-4.19992100
H	0.63970700	-1.88325800	-3.17397700
C	2.47575400	0.96041100	-2.38002200
H	1.77736900	1.38533000	-1.65313600
H	3.49032800	1.22441500	-2.07188300
H	2.28180600	1.43517300	-3.34894800
C	3.36153600	-1.14098600	-3.45476000
H	3.21228300	-2.20697800	-3.64312600
H	3.29259500	-0.62466100	-4.41989500
H	4.37647000	-0.99011900	-3.08439900
C	1.70829800	-3.89813500	-1.55338800
H	1.84130100	-4.97339600	-1.38331700
H	0.66430400	-3.65165300	-1.33611700
H	1.89557400	-3.70728100	-2.61326500
C	4.13210200	-3.49568900	-1.01581300
H	4.23780700	-4.58484800	-0.94338100
H	4.39975900	-3.20831600	-2.03381400
H	4.85366800	-3.05309900	-0.32528500
C	2.44738700	-3.53609100	0.82445200
H	3.11260300	-2.99651400	1.50585000
H	1.41462500	-3.35453900	1.13881000
H	2.65483700	-4.60724500	0.93406500
C	3.61315100	-0.40827500	0.19010000
C	3.40775400	0.28390800	1.40821700
C	4.91094000	-0.41303100	-0.36127900
C	4.49654500	0.93515800	2.01514000
C	5.97761000	0.23692900	0.25361500
H	5.10064000	-0.92992700	-1.29176800
C	5.76950600	0.91827300	1.45237900
H	4.32632700	1.46328000	2.94873200
H	6.96125500	0.21130600	-0.20572500
H	6.58905700	1.43270800	1.94559500
C	2.10300900	0.39565600	2.13131900
C	1.69281500	-0.59946700	3.03082200
C	1.32350500	1.55393600	1.99575800
C	0.51494700	-0.44548100	3.76588400
H	2.30063300	-1.49008500	3.15663300
C	0.14150900	1.70358700	2.72434200
H	1.63957500	2.33452600	1.31096700
C	-0.26651700	0.70356400	3.61039400
H	0.21206400	-1.22038100	4.46472500
H	-0.45645700	2.60063200	2.59621600
H	-1.18242300	0.82211500	4.18258700
C	-1.86856000	2.94575200	-0.00595000
C	-2.13791000	4.20127400	0.56037200
C	-0.68006200	2.73560100	-0.72263000
C	-1.22992900	5.24331400	0.40982500
H	-3.05332800	4.34184100	1.12378900
C	0.23051800	3.77145800	-0.88131300
H	-0.47491900	1.75082800	-1.12502900
C	-0.05553900	5.01498400	-0.31132100
H	-1.41845300	6.21776200	0.84279700
H	1.15505500	3.62538300	-1.42571900
N	0.90515700	6.10427000	-0.46855600
O	0.63695200	7.19691500	0.03909600
O	1.94115400	5.88089700	-1.10194400

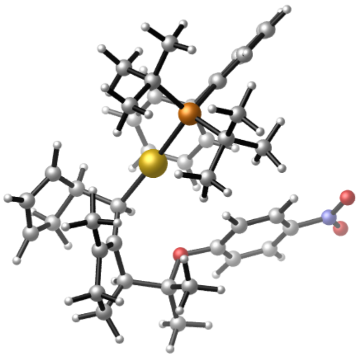

Xa

E (opt) = -2350.88666210 Hartrees
G (opt) = -2350.126485 Hartrees
E (SP) = -2351.37050837 Hartrees

Au	-0.27748100	-0.96861200	0.11672700
P	1.83766500	-1.69914000	-0.74964800
C	-2.20883000	-0.46294300	0.82224600
H	-2.08788800	0.44055900	1.41970200
C	-2.72229600	-1.53465100	1.95742200
H	-2.02079400	-1.41379900	2.78303000

C	-2.83936300	-2.92018900	1.49074100
C	-3.25337400	-0.20686400	-0.19169900
C	-3.70808700	-1.04969300	-1.17354000
C	1.83081900	-3.60763600	-0.65184800
C	2.01247400	-1.05281900	-2.55110600
C	-4.13176800	-3.21154400	1.09262000
H	-4.45483600	-4.11354300	0.58853500
C	-4.92788800	-2.09018100	1.35197400
H	-5.95698200	-1.96208200	1.03435100
C	-4.86230600	-0.45388200	-1.93835300
H	-4.53193300	-0.18224100	-2.95126400
H	-5.68075600	-1.17159800	-2.06983100
C	-4.01970600	1.11798800	-0.24706700
H	-4.30471300	1.44062400	0.76146300
C	-3.07483600	-2.31007500	-1.65612200
H	-2.32433400	-2.69728700	-0.96676400
H	-3.81711700	-3.08828200	-1.86415400
H	-2.55565100	-2.10001500	-2.60248300
C	-5.26668100	0.77762600	-1.10320300
H	-6.08868300	0.51053000	-0.43167100
H	-5.61330900	1.60891100	-1.72060400
C	-3.16822100	2.29177100	-0.81686300
C	-2.62679800	2.01006500	-2.21803800
H	-3.46053100	1.90409200	-2.91667100
H	-2.00793400	2.83814200	-2.57274800
H	-2.03273400	1.09314500	-2.23939200
C	-3.94895100	3.60801300	-0.78838000
H	-4.35533900	3.79411300	0.21033000
H	-3.29291500	4.44122200	-1.05756800
H	-4.77415000	3.59379500	-1.50477300
O	-2.07672500	2.40038900	0.16783000
H	-1.99116700	-3.59110100	1.40026600
C	-4.20653800	-1.17532400	2.26052600
H	-4.42215900	-1.49490500	3.29253300
H	-4.47686200	-0.12281900	2.18368200
C	0.58840400	-0.93091400	-3.14184400
H	-0.00722300	-0.18403700	-2.60911700
H	0.66707400	-0.61266400	-4.18840600
H	0.04168400	-1.87655200	-3.11823500
C	2.62506200	0.36017400	-2.46564100
H	2.13028100	0.97064000	-1.70599000
H	3.69189200	0.33387100	-2.23219600
H	2.50232500	0.86182000	-3.43274300
C	2.85373900	-1.92055200	-3.50367100
H	2.41253200	-2.90695800	-3.66271100
H	2.89698000	-1.41906700	-4.47818700
H	3.88337100	-2.04999200	-3.16612500
C	0.68549300	-4.13982300	-1.53437900
H	0.58568000	-5.21983100	-1.37240600
H	-0.27137400	-3.67345500	-1.28266800
H	0.87535600	-3.98289600	-2.59919300
C	3.15211000	-4.29112200	-1.04688100
H	3.01511300	-5.37659200	-0.96856100
H	3.45798900	-4.07392600	-2.07082400
H	3.96851600	-4.01784200	-0.37413500
C	1.53473500	-3.95825700	0.82228600
H	2.30683700	-3.57103200	1.49385100
H	0.56672900	-3.56763400	1.15105700
H	1.51617400	-5.04935400	0.93057800
C	3.37180800	-1.17371500	0.14627000
C	3.35163400	-0.48623900	1.38396600
C	4.62309200	-1.44985700	-0.44169400
C	4.57118300	-0.11052000	1.97510800
C	5.82080200	-1.06822800	0.15660900
H	4.66999000	-1.96863400	-1.38955400
C	5.79584600	-0.39298300	1.37667600
H	4.54326200	0.41736000	2.92374700
H	6.76360900	-1.29660400	-0.33156300
H	6.71983100	-0.08656700	1.85836700
C	2.11954400	-0.10314100	2.14179800
C	1.54104500	-0.98738300	3.06408400
C	1.59145300	1.19026900	2.01630400
C	0.44130100	-0.59239200	3.82918200
H	1.95902900	-1.98181900	3.18416400
C	0.48995900	1.58411400	2.77921400
H	2.03923100	1.88371900	1.31197400
C	-0.08904500	0.69325900	3.68621600
H	0.00638900	-1.28560600	4.54398300
H	0.08661800	2.58495200	2.66095900
H	-0.94290100	1.00027100	4.28365100
C	-1.01203900	3.22278400	-0.04078300
C	-0.96678900	4.46225100	0.61653800
C	0.07950800	2.79594200	-0.81362600
C	0.15384400	5.27607400	0.49860900
H	-1.81323300	4.76706100	1.22162100
C	1.19918100	3.60619500	-0.94502400
H	0.04153500	1.82140000	-1.28378000
C	1.22354000	4.83865200	-0.28648000
H	0.20712300	6.23340900	1.00182200
H	2.05051700	3.29273100	-1.53610300
N	2.40623800	5.68601500	-0.41391600
O	2.40672900	6.77846900	0.16087700
O	3.35172300	5.26941300	-1.09013700

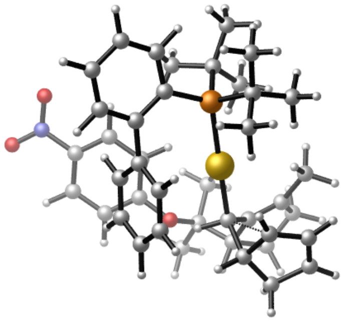

TS$_{Xa\text{-}XIa}$

E (opt) = -2350.88001903 Hartrees
G (opt) = -2350.120867 Hartrees
E (SP) = -2351.36452711 Hartrees

Au	-0.06336200	-1.09201800	0.12762300
P	2.16213400	-1.33337600	-0.73287900
C	-2.07066300	-0.85660300	0.79176600
H	-1.98441800	0.11296700	1.28229300
C	-2.55479100	-1.79468300	2.06818500
H	-2.24016900	-1.28214500	2.97443600
C	-1.79012400	-2.98048300	1.71828100
C	-3.12246800	-0.81868100	-0.26450500
C	-3.33684600	-1.64402800	-1.32080100
C	2.62235000	-3.18297900	-0.62982500
C	2.15275600	-0.65270600	-2.52907700
C	-2.61440900	-3.94061400	1.09228700
H	-2.27173900	-4.84671600	0.61002900
C	-3.91718500	-3.52165400	1.21021300
H	-4.78131700	-4.05624200	0.82837600
C	-4.62671700	-1.31043500	-2.03882900
H	-4.41337200	-0.86348900	-3.02016000
H	-5.22203100	-2.21035700	-2.23813400
C	-4.21464500	0.25926100	-0.19842900
H	-4.55260000	0.40618400	0.83540300
C	-2.47244600	-2.73404400	-1.87326400
H	-1.56222600	-2.88234600	-1.29113900
H	-3.01538700	-3.68521600	-1.92698900
H	-2.18112800	-2.48364500	-2.90275000
C	-5.34000800	-0.33021700	-1.08450600
H	-6.03233000	-0.88643300	-0.44409100
H	-5.92829800	0.42541100	-1.61085000
C	-3.72999500	1.66520800	-0.66836800
C	-3.24914100	1.68012200	-2.11852800
H	-4.08790900	1.46374600	-2.78403200
H	-2.86205000	2.66473000	-2.39295300
H	-2.46820400	0.93583800	-2.28834700
C	-4.81148700	2.72565900	-0.44336500
H	-5.13844600	2.72330600	0.60082300
H	-4.42118300	3.71945200	-0.68362800

H	-5.68053500	2.54688900	-1.08184000
O	-2.62619400	1.97141100	0.26297100
H	-0.73273900	-3.09595600	1.92017200
C	-4.01334300	-2.27232900	2.01698700
H	-4.34370300	-2.54752900	3.03106700
H	-4.74656400	-1.56131100	1.63693100
C	1.60926500	-3.98085500	-1.47275500
H	0.57648300	-3.75125300	-1.19278300
H	1.72638700	-3.79319700	-2.54332600
H	1.77309700	-5.05222000	-1.30508600
C	4.05369000	-3.54396800	-1.06496600
H	4.80533900	-3.06106100	-0.43636000
H	4.18341900	-4.62716800	-0.95231600
H	4.25841100	-3.29766700	-2.10768200
C	2.46523300	-3.56988400	0.85684900
H	1.44896000	-3.39476100	1.22239600
H	2.68843600	-4.63723900	0.97262300
H	3.15962000	-3.01296800	1.49348800
C	0.74678700	-0.90256300	-3.12292500
H	0.74409500	-0.57208900	-4.16857900
H	0.46457600	-1.95796600	-3.10525700
H	-0.02458700	-0.33571600	-2.59283800
C	2.36771800	0.86971300	-2.41343500
H	1.73346900	1.30251700	-1.63537500
H	3.40475500	1.12426200	-2.18247600
H	2.10509000	1.34298900	-3.36662600
C	3.19090300	-1.25633500	-3.48949200
H	3.01724900	-2.32085900	-3.66473900
H	3.10346400	-0.74647800	-4.45668400
H	4.21846800	-1.12112300	-3.14894400
C	3.52581800	-0.44190100	0.15066400
C	3.33818100	0.26680600	1.36255700
C	4.80874200	-0.43093200	-0.43427000
C	4.42922000	0.95177500	1.92743300
C	5.87853000	0.24915800	0.14138700
H	4.98201200	-0.95637000	-1.36338900
C	5.68755900	0.94867700	1.33245400
H	4.27320000	1.49435400	2.85513900
H	6.85023400	0.23438300	-0.34315800
H	6.50889400	1.48819000	1.79500000
C	2.05280500	0.36486000	2.12239900
C	1.66479400	-0.64569500	3.01539000
C	1.27342500	1.52755800	2.03180400
C	0.50845600	-0.50296400	3.78573700
H	2.27332700	-1.53974100	3.10755900
C	0.11271100	1.66626700	2.79606800
H	1.57282500	2.31946200	1.35279800
C	-0.27298000	0.65134200	3.67471900
H	0.22120400	-1.29166500	4.47569500
H	-0.48628200	2.56672800	2.70187200
H	-1.17112200	0.76249600	4.27574900
C	-1.73982700	2.97328800	0.01021000
C	-0.60202600	2.72729800	-0.77557900
C	-1.91276600	4.22162600	0.62811700
C	0.34727100	3.72381500	-0.95722800
H	-0.47015100	1.74715500	-1.21854500
C	-0.96263100	5.22247400	0.45890400
H	-2.78956000	4.38801400	1.24364200
C	0.15595700	4.96113300	-0.33597200
H	1.23263400	3.55256400	-1.55631900
H	-1.07771300	6.19025000	0.93094800
N	1.16049600	6.00587400	-0.51758700
O	2.13194000	5.76142800	-1.23956600
O	0.99228000	7.08454100	0.05850700

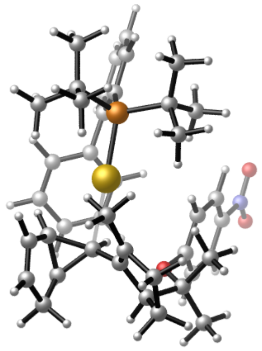

XIa

E (opt) = -2350.89746608 Hartrees
G (opt) = -2350.132989 Hartrees
E (SP) = -2351.37913207 Hartrees

Au	0.00305300	-1.05785200	0.14563300
P	2.11212200	-1.44267500	-0.71976000
C	-2.34168300	-0.85974700	0.96723500
H	-1.77931300	0.07329700	1.09168000
C	-3.02646000	-1.12815600	2.32286600
H	-2.86138400	-0.38182700	3.09166200
C	-1.93998900	-2.07414000	1.93229700
C	-3.17929700	-0.91548700	-0.26996800
C	-3.26415700	-1.85473200	-1.23228900
C	2.48443700	-3.29482600	-0.45803000
C	2.09200600	-0.89137800	-2.55830000
C	-2.59822600	-3.38977600	1.69109300
H	-2.05180500	-4.27383300	1.38212300
C	-3.91965000	-3.30775100	1.88988900
H	-4.63070500	-4.11438300	1.74076000
C	-4.38776700	-1.55333800	-2.20074300
H	-3.98518400	-1.23963800	-3.17387200
H	-4.99737300	-2.44482100	-2.39459300
C	-4.18588300	0.21681800	-0.50851700
H	-4.67836600	0.49276200	0.43145700
C	-2.42324400	-3.07016000	-1.46085200
H	-1.62020400	-3.17037200	-0.73095300
H	-3.03239000	-3.98198700	-1.42932700
H	-1.97296100	-3.02650700	-2.46151500
C	-5.18558600	-0.43356700	-1.49977400
H	-6.00372200	-0.87822300	-0.92466400
H	-5.63281500	0.27635300	-2.19987300
C	-3.55480700	1.53154700	-1.05226500
C	-2.89607700	1.35951800	-2.42006700
H	-3.65211100	1.10317000	-3.16546800
H	-2.42027300	2.28779100	-2.74567000
H	-2.14451100	0.56668300	-2.39842800
C	-4.58172900	2.66493800	-1.07725500
H	-5.02375400	2.80344100	-0.08602700
H	-4.11003400	3.60362700	-1.38342900
H	-5.38430900	2.44916600	-1.78752500
O	-2.54319900	1.86436600	-0.02516500
H	-0.97040000	-2.03370800	2.41524500
C	-4.33865500	-1.91886100	2.31449100
H	-4.78637000	-1.92021700	3.31650900
H	-5.09097300	-1.49595400	1.63666400
C	1.49485800	-4.12421600	-1.29590000
H	0.45588600	-3.85832800	-1.08268200
H	1.66796300	-4.01561200	-2.36908700
H	1.62690100	-5.18304200	-1.04513500
C	3.93025000	-3.68883900	-0.80991000
H	4.65534000	-3.20607300	-0.15096200
H	4.02957900	-4.77114100	-0.66873200
H	4.19220300	-3.46710300	-1.84545000
C	2.25447500	-3.58310800	1.03959300
H	1.21441800	-3.41587200	1.33472100
H	2.49474000	-4.63457300	1.23403400
H	2.90039200	-2.97048900	1.67498900

C	0.68121700	-1.17043000	-3.12441300
H	0.67013100	-0.88645200	-4.18292100
H	0.40338700	-2.22462900	-3.05868200
H	-0.08657100	-0.58256000	-2.61273100
C	2.33708000	0.63105500	-2.56022400
H	1.70765100	1.14320900	-1.82753400
H	3.37814800	0.88073200	-2.34391800
H	2.09068100	1.02624200	-3.55196500
C	3.12293500	-1.58645400	-3.46734800
H	2.94580400	-2.66095600	-3.54843900
H	3.02654600	-1.16013500	-4.47264000
H	4.15398700	-1.42339900	-3.14982800
C	3.46436400	-0.50720800	0.12240100
C	3.29493700	0.29311600	1.27949800
C	4.74108300	-0.56407400	-0.47342200
C	4.39917800	1.01441100	1.76799500
C	5.82293700	0.14999400	0.03284900
H	4.90078000	-1.17136400	-1.35334600
C	5.64963000	0.95180300	1.16009800
H	4.25976300	1.63103200	2.65071700
H	6.78953400	0.08086000	-0.45658000
H	6.47976500	1.52231200	1.56592300
C	2.02890500	0.45995900	2.05963100
C	1.64616400	-0.48263900	3.02726900
C	1.27579500	1.63523900	1.92077400
C	0.51752700	-0.26257700	3.82038100
H	2.23687900	-1.38283600	3.16162400
C	0.13954900	1.84838500	2.70496100
H	1.57809800	2.37850200	1.19129200
C	-0.24290400	0.89941000	3.65622600
H	0.23405100	-0.99694900	4.56893700
H	-0.43939600	2.75690700	2.57186100
H	-1.11832800	1.07065100	4.27564200
C	-1.68104100	2.90575100	-0.19697400
C	-0.49167400	2.72740200	-0.92028200
C	-1.93480400	4.12481900	0.44940100
C	0.43946500	3.75375600	-1.00124800
H	-0.30554200	1.77391400	-1.39739000
C	-1.00730700	5.15790100	0.37818600
H	-2.85472500	4.24203900	1.01095600
C	0.17109900	4.95847900	-0.34591300
H	1.36636400	3.62611100	-1.54656200
H	-1.18264200	6.10331300	0.87635700
N	1.15782300	6.03403600	-0.40893300
O	2.20736500	5.82599800	-1.02483100
O	0.89679300	7.09913800	0.15699800

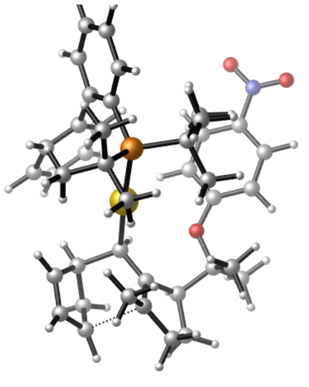

TS$_{Xa\text{-}XIIa}$

E (opt) = -2350.88636051 Hartrees
G (opt) = -2350.119417 Hartrees
E (SP) = -2351.36950945 Hartrees

Au	-0.45526600	-0.75698800	0.06389400
P	1.52143300	-1.37002200	-1.15017000
C	-2.30999500	-0.20448800	0.95939400
H	-2.06911000	0.58277400	1.67536200
C	-2.98285000	-1.35351100	1.87854200
H	-2.36427100	-1.42001300	2.77188000
C	-3.21851200	-2.66544300	1.23017400
C	-3.28142000	0.33437200	0.00877800
C	-3.99003500	-0.34620100	-0.98182300
C	1.12227900	-2.98612900	-2.08839600
C	1.95250000	0.09230500	-2.32134000
C	-4.45744800	-2.70927100	0.65288200
H	-4.83809600	-3.49625900	0.01420700
C	-5.10227800	-1.46149200	0.89850700
H	-6.07520200	-1.17321200	0.51921500
C	-5.06839900	0.53238700	-1.57834000
H	-4.76855300	0.83656200	-2.59000300
H	-6.01803700	-0.00286700	-1.68800900
C	-3.78495500	1.77582200	0.10359400
H	-3.88785600	2.07177900	1.15352200
C	-3.49967400	-1.50829100	-1.78889300
H	-2.74791400	-2.10400200	-1.27285600
H	-4.31770500	-2.15662200	-2.11761300
H	-3.02833700	-1.09779400	-2.69242600
C	-5.15633700	1.73029000	-0.61377200
H	-5.93272400	1.54711200	0.13509200
H	-5.41349100	2.66102600	-1.12377700
C	-2.76841300	2.80026400	-0.51188200
C	-2.52192700	2.53843000	-1.99950500
H	-3.44186800	2.72737800	-2.55776900
H	-1.75578200	3.19403600	-2.41224400
H	-2.21520500	1.50460900	-2.16833800
C	-3.23833000	4.23012700	-0.23560900
H	-3.33582200	4.39380100	0.84160900
H	-2.54106900	4.96896500	-0.63600300
H	-4.20946100	4.40862300	-0.70320600
O	-1.57464900	2.52109400	0.28572400
H	-2.46330600	-3.44156700	1.16048000
C	-4.45208000	-0.86969100	2.10050200
H	-4.85516500	-1.37968800	2.98703400
H	-4.58082600	0.20365500	2.23658500
C	-0.00544200	-2.69725800	-3.09695500
H	-0.84578500	-2.17480700	-2.63300500
H	0.34531000	-2.10420200	-3.94515200
H	-0.37925600	-3.64923200	-3.49288800
C	2.30915400	-3.63873100	-2.81818000
H	3.07663100	-3.98159200	-2.12058200
H	1.93891900	-4.51986800	-3.35629100
H	2.77397300	-2.97812400	-3.55205400
C	0.61035700	-3.97811000	-1.02220900
H	-0.29459400	-3.61377000	-0.52632800
H	0.37108700	-4.93152600	-1.50839500
H	1.36842200	-4.17609400	-0.25793700
C	0.62934600	0.74945900	-2.77026100
H	0.86322800	1.60227300	-3.41872100
H	-0.01268900	0.06898400	-3.33440900
H	0.06412800	1.12664500	-1.91625900
C	2.72117000	1.11731500	-1.46426600
H	2.18139800	1.35378300	-0.54327400
H	3.72277900	0.77080500	-1.19778200
H	2.82784700	2.04643900	-2.03550900
C	2.76943300	-0.25800800	-3.57685200
H	2.23461500	-0.94109400	-4.24077900
H	2.95065400	0.66745500	-4.13723600
H	3.74634100	-0.68747500	-3.34910400
C	3.06975000	-1.71274500	-0.19050600
C	3.13573500	-1.69265900	1.22338200
C	4.26329400	-1.93794600	-0.90686100
C	4.38151600	-1.87713100	1.85090200
C	5.48574400	-2.12948300	-0.26918500
H	4.24703200	-1.95916800	-1.98705600
C	5.54782200	-2.09313900	1.12327600
H	4.41994000	-1.85558600	2.93599900
H	6.38096300	-2.29943300	-0.85992100
H	6.49293900	-2.23376400	1.63962600
C	1.97860400	-1.50236500	2.15111100
C	1.13956800	-2.57812800	2.47944400
C	1.80426200	-0.27810200	2.81218600
C	0.14578400	-2.42949100	3.44882400
H	1.27817900	-3.53369500	1.98392100
C	0.80187600	-0.12623100	3.77321100
H	2.45861500	0.55347500	2.57036100

C	-0.02542400	-1.20355100	4.09879800
H	-0.48956500	-3.27346700	3.70266300
H	0.67911300	0.82844400	4.27709900
H	-0.79376500	-1.09191300	4.85853100
C	-0.37999500	3.13912800	0.25945500
C	0.49330100	2.71223900	1.28516800
C	0.06154100	4.10842000	-0.66124700
C	1.77096800	3.22809100	1.39072200
H	0.13898000	1.95974200	1.97869000
C	1.34867000	4.62351600	-0.55796600
H	-0.57836400	4.46537100	-1.45378100
C	2.19612200	4.18423900	0.45992900
H	2.44326900	2.89686400	2.17230400
H	1.69971700	5.36285800	-1.26712200
N	3.54672100	4.71020500	0.54213500
O	4.27823800	4.30449400	1.45302000
O	3.90835000	5.53709600	-0.30333600

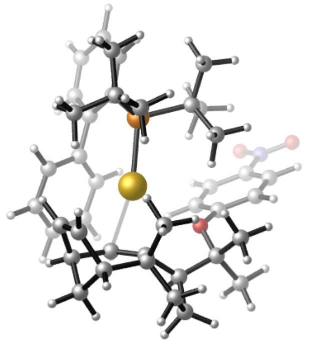

XIIa

E (opt) = -2350.92755390 Hartrees
G (opt) = -2350.156802 Hartrees
E (SP) = -2351.40809075 Hartrees

Au	-0.91031500	-0.32270700	-0.01170500
P	0.53435000	-1.88005700	-1.10552300
C	-2.49355700	0.75328300	1.36976600
H	-1.82086700	1.04460000	2.17473700
C	-3.69611500	-0.10692800	1.77079900
H	-3.60322900	-0.38813900	2.81958300
C	-3.95952400	-1.28043700	0.83667500
C	-2.56023600	1.57786700	0.26059100
C	-3.76441700	1.50695400	-0.71911000
C	-0.56705700	-3.13642800	-2.02892200
C	1.71661900	-0.90658200	-2.26788000
C	-4.65307100	-0.85409000	-0.22300400
H	-4.93657200	-1.44775100	-1.08535800
C	-4.92609900	0.64418500	-0.09181400
H	-5.86333000	0.95214000	-0.56527500
C	-4.16345800	3.01221400	-0.85343300
H	-4.01396000	3.33582200	-1.88760800
H	-5.22181800	3.16463900	-0.62392200
C	-1.97968500	3.00092500	0.36482700
H	-1.66784500	3.13450200	1.40356500
C	-3.43285800	0.92473000	-2.10347200
H	-3.03353600	-0.08588600	-2.01764200
H	-4.34815500	0.88027200	-2.70510400
H	-2.71000000	1.53215400	-2.64582800
C	-3.26798200	3.82939700	0.10164200
H	-3.77842200	3.96864200	1.05919500
H	-3.05229500	4.82533500	-0.29214800
C	-0.72558100	3.44442800	-0.43900300
C	-0.88438500	3.37456500	-1.95389400
H	-1.77206500	3.93405000	-2.26081400
H	-0.02813800	3.81401800	-2.46989800
H	-0.99032200	2.34200100	-2.28035800
C	-0.30730700	4.84669800	0.01273000
H	-0.16874200	4.88225600	1.09742400
H	0.62577400	5.15108100	-0.46585700
H	-1.06951400	5.57737200	-0.26547700
O	0.26734500	2.46517600	0.03244700
H	-3.57816600	-2.27838100	1.01853000
C	-4.92667200	0.78593800	1.44265200
H	-5.83559400	0.35427100	1.87237700
H	-4.82290300	1.81696900	1.79578000
C	-1.34540800	-2.39130700	-3.12770500
H	-1.87769700	-1.52420200	-2.73095800
H	-0.69652600	-2.05617600	-3.94050100
H	-2.09006500	-3.07218300	-3.55617400
C	0.18267200	-4.32946700	-2.64914100
H	0.65139700	-4.95748400	-1.88851300
H	-0.55114800	-4.94966500	-3.17710300
H	0.94007900	-4.03135600	-3.37527000
C	-1.55945200	-3.68161900	-0.98342300
H	-2.19055500	-2.88788000	-0.57933900
H	-2.21053800	-4.42035100	-1.46529500
H	-1.04580400	-4.17991700	-0.15523100
C	0.94829000	0.32307100	-2.79177600
H	1.59890200	0.88119000	-3.47498600
H	0.04424400	0.05083400	-3.34225300
H	0.67014900	0.98956500	-1.97569900
C	2.89373600	-0.41845900	-1.39979200
H	2.55101600	0.06762800	-0.48236900
H	3.56907100	-1.23225100	-1.12592200
H	3.46848400	0.31869800	-1.97064500
C	2.25320300	-1.69157000	-3.47782600
H	1.45619500	-2.00307100	-4.15634100
H	2.91987800	-1.02667100	-4.03993700
H	2.84076200	-2.56749600	-3.19974200
C	1.62936900	-2.88220900	-0.00314700
C	1.61067200	-2.82934200	1.40975900
C	2.59205900	-3.70456000	-0.62604300
C	2.55862400	-3.57935500	2.13093600
C	3.51477900	-4.44716900	0.10401700
H	2.63585800	-3.76437200	-1.70405700
C	3.50279800	-4.38012300	1.49667100
H	2.53758000	-3.52789000	3.21527600
H	4.23962400	-5.06598400	-0.41614700
H	4.21926200	-4.94609200	2.08465900
C	0.65599700	-2.04955900	2.25550700
C	-0.61303600	-2.56555000	2.56249100
C	1.08835500	-0.87902700	2.89762500
C	-1.42752600	-1.92504300	3.49806200
H	-0.94660700	-3.48265700	2.08736400
C	0.26460600	-0.22952200	3.81962400
H	2.07706500	-0.48770400	2.67907000
C	-0.99212500	-0.75636500	4.12874800
H	-2.39851000	-2.34560400	3.74136100
H	0.61360100	0.67472600	4.31048000
H	-1.62640800	-0.26312500	4.85956600
C	1.60671000	2.66233800	0.08673800
C	2.23611500	2.18803400	1.25149700
C	2.37912200	3.20260100	-0.95571500
C	3.61631200	2.22681800	1.37226300
H	1.61721000	1.78305500	2.04289500
C	3.76204400	3.25548300	-0.83380900
H	1.90961300	3.55862500	-1.86187300
C	4.36973000	2.76175400	0.32262900
H	4.11127000	1.85789900	2.26199000
H	4.37195700	3.66075300	-1.63153200
N	5.82191300	2.80017900	0.43541100
O	6.33884800	2.36631100	1.47005400
O	6.46993000	3.26266500	-0.50913400

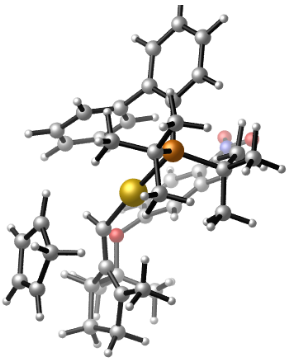

IXb

E (opt) = -2350.88343487 Hartrees
G (opt) = -2350.127208 Hartrees
E (SP) = -2351.37141291 Hartrees

Au	0.27427800	-0.93207400	-0.03954600
P	2.58220000	-0.85290400	-0.66944300
C	-1.71112900	-0.94075100	0.39495500
H	-1.96197800	-0.42708500	1.32007900
C	-1.54507800	-3.11513500	1.93742100
H	-0.62399900	-2.72494400	2.34755600
C	-1.61583000	-4.16831900	0.86783800
C	-2.84626800	-1.22277200	-0.38934900
C	-2.90684800	-1.99762000	-1.53593000
C	3.25656400	-2.63207600	-0.58014400
C	2.64174400	-0.09467600	-2.42986700
C	-3.09196700	-4.38779500	0.70221500
H	-3.52506000	-5.07684200	-0.01312600
C	-3.76093700	-3.64848800	1.62451000
H	-4.83452900	-3.62551600	1.77297300
C	-4.31914200	-2.10798800	-2.03261400
H	-4.39866100	-1.51574900	-2.95654400
H	-4.56932700	-3.13700800	-2.31306600
C	-4.23136800	-0.67890500	-0.04036900
H	-4.42309000	-0.80558000	1.03085500
C	-1.80740600	-2.62484700	-2.31266700
H	-0.82246600	-2.45819700	-1.87510600
H	-1.98486100	-3.70098200	-2.42125200
H	-1.81977000	-2.21062700	-3.33014900
C	-5.19209600	-1.54413000	-0.89206700
H	-5.56301200	-2.36991800	-0.28045700
H	-6.06022700	-0.99539400	-1.26309900
C	-4.32604900	0.85175300	-0.32372000
C	-4.17420500	1.19834700	-1.80396800
H	-5.02221000	0.79307200	-2.36081800
H	-4.17354500	2.28154400	-1.95057500
H	-3.25285700	0.79334200	-2.22837400
C	-5.61600000	1.44397500	0.24428500
H	-5.68446800	1.24257700	1.31725000
H	-5.63025500	2.52754400	0.09153600
H	-6.49624600	1.02143000	-0.24899300
O	-3.22208900	1.41012500	0.48143400
H	-1.10498500	-3.87914600	-0.05569700
C	-2.80785000	-2.86134700	2.38994000
C	1.38510600	-0.55578500	-3.20401800
H	0.46591900	-0.18656300	-2.74060700
H	1.43479600	-0.14812900	-4.22077200
H	1.31288000	-1.64251900	-3.28508600
C	2.55175000	1.43361300	-2.23958600
H	1.71289500	1.70840100	-1.59043200
H	3.46579300	1.85233000	-1.81181400
H	2.38884000	1.90452800	-3.21616300
C	3.87728100	-0.45228300	-3.27379800
H	3.92248200	-1.52114000	-3.49614800
H	3.80690600	0.07908700	-4.23054300
H	4.81826000	-0.15149800	-2.81081200
C	2.47538300	-3.49769400	-1.58681000
H	2.73484700	-4.55056200	-1.42435700
H	1.39217800	-3.39775000	-1.46040000
H	2.72892600	-3.25159300	-2.62116900
C	4.76659400	-2.77802600	-0.83608900
H	5.02114700	-3.84414500	-0.79586900
H	5.07066400	-2.40535100	-1.81553400
H	5.36022800	-2.27357200	-0.07011500
C	2.95893500	-3.12195400	0.85375800
H	3.47314600	-2.51324900	1.60376200
H	1.88780200	-3.11285200	1.07903400
H	3.31824200	-4.15271600	0.95810300
C	3.70399500	0.17538900	0.38379800
C	3.28217200	0.81836000	1.57380500
C	5.03156600	0.37529800	-0.04629000
C	4.19394100	1.63521700	2.26615100
C	5.92224000	1.18267100	0.65606700
H	5.38407900	-0.10197200	-0.94971000
C	5.49933200	1.82228500	1.82057100
H	3.85896500	2.12635500	3.17484700
H	6.93638000	1.31118500	0.28993200
H	6.17823600	2.46020600	2.37893900
C	1.92223900	0.70766000	2.18875300
C	1.59867300	-0.37229800	3.02472800
C	0.99007500	1.74372900	2.02585700
C	0.35982100	-0.42313900	3.66805200
H	2.32142600	-1.16862000	3.17147600
C	-0.24956800	1.69010500	2.66691800
H	1.23708200	2.58996500	1.39174100
C	-0.56859100	0.60640000	3.48861300
H	0.12334200	-1.26358600	4.31447600
H	-0.96261800	2.49537600	2.52333400
H	-1.53076500	0.56773800	3.99130400
C	-2.53925800	2.52846400	0.10846500
C	-2.87096300	3.76131600	0.68948100
C	-1.43835000	2.42968800	-0.75793100
C	-2.10673000	4.88986500	0.41301600
H	-3.71535300	3.81499100	1.36762700
C	-0.67353400	3.55186500	-1.04406600
H	-1.18037300	1.46441700	-1.17597800
C	-1.01617800	4.77076600	-0.45167600
H	-2.34163200	5.84820300	0.85920600
H	0.18381200	3.49193800	-1.70250200
N	-0.19742000	5.94796000	-0.73122200
O	-0.51754900	7.01919200	-0.20860300
O	0.77805900	5.81517600	-1.47648300
H	-1.12401400	-5.09356700	1.20717100
H	-3.07175300	-2.17655300	3.18814000

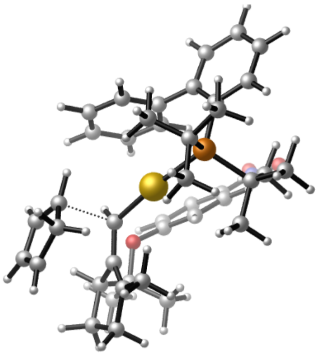

TS$_{IXb-Xb}$

E (opt) = -2350.88278541 Hartrees
G (opt) = -2350.124460 Hartrees
E (SP) = -2351.36961909 Hartrees

Au	0.24602900	-1.05008600	0.04996400
P	2.52065400	-0.97841000	-0.67554900
C	-1.75875700	-1.02482700	0.51242600
H	-2.00425800	-0.30618300	1.28808400
C	-1.81782900	-2.72439800	2.02011400
H	-0.90172000	-2.40626900	2.49715800

C	-1.93185100	-3.96022800	1.16678300
C	-2.85524000	-1.26228300	-0.37119500
C	-2.91142300	-2.12878300	-1.44059200
C	3.23365300	-2.74138700	-0.55094200
C	2.49088700	-0.28173200	-2.46378800
C	-3.41128300	-4.09911200	0.97452200
H	-3.87193200	-4.83897300	0.33067100
C	-4.06110900	-3.22186800	1.79410100
H	-5.13414600	-3.12832100	1.91074200
C	-4.27949000	-2.12841900	-2.06782500
H	-4.21954600	-1.61397400	-3.03800100
H	-4.62369600	-3.14558800	-2.28652800
C	-4.19582100	-0.54682200	-0.19467400
H	-4.48628300	-0.56122300	0.86295200
C	-1.82811900	-2.93070400	-2.07489800
H	-0.86956600	-2.83291500	-1.56365300
H	-2.10903500	-3.98900300	-2.12532100
H	-1.70435200	-2.59605400	-3.11377700
C	-5.17388800	-1.38019400	-1.05915300
H	-5.68194100	-2.10534900	-0.41806800
H	-5.94501600	-0.77985900	-1.54657100
C	-4.12219900	0.95754900	-0.59595600
C	-3.75154700	1.16644900	-2.06371800
H	-4.54124700	0.76316300	-2.70235000
H	-3.65623000	2.23117300	-2.29306600
H	-2.81102100	0.67174000	-2.31713500
C	-5.42056300	1.68839600	-0.25080400
H	-5.64932600	1.57592400	0.81310200
H	-5.32527900	2.75472000	-0.47674200
H	-6.26038500	1.29796100	-0.83212500
O	-3.07450600	1.48667000	0.29721800
H	-1.34677600	-3.94628700	0.24723200
C	-3.09041300	-2.39806800	2.46024600
C	1.23263200	-0.82062200	-3.18314900
H	0.31157800	-0.50698300	-2.68374300
H	1.21648500	-0.41622700	-4.20231800
H	1.22284600	-1.90963700	-3.25974500
C	2.34126100	1.24703200	-2.31839500
H	1.50290800	1.50448000	-1.66248400
H	3.24417400	1.71631500	-1.91986100
H	2.14116000	1.68089100	-3.30510100
C	3.71636500	-0.61387700	-3.33073800
H	3.81893200	-1.68849100	-3.49928100
H	3.58631600	-0.13876100	-4.31055700
H	4.65061000	-0.23391500	-2.91503600
C	2.44261500	-3.65494700	-1.50582200
H	2.72126600	-4.69722300	-1.31021200
H	1.36129900	-3.56589300	-1.35937500
H	2.67016000	-3.44439100	-2.55409500
C	4.73840000	-2.86815200	-0.84613100
H	5.01257100	-3.92856800	-0.78815500
H	5.00799700	-2.51361600	-1.84255900
H	5.34444400	-2.33554800	-0.10942400
C	2.98781800	-3.18868200	0.90625000
H	3.51010800	-2.54351400	1.61940400
H	1.92347800	-3.19340700	1.16214900
H	3.37260300	-4.20745200	1.03485200
C	3.66889500	0.11226000	0.28525600
C	3.28162200	0.81019300	1.45622900
C	4.97659300	0.30899100	-0.20382800
C	4.20239100	1.68155800	2.06553100
C	5.87756200	1.16877300	0.41869800
H	5.30526700	-0.21252800	-1.09150500
C	5.48596700	1.86701100	1.56019500
H	3.89298000	2.21478400	2.95963500
H	6.87521700	1.29207300	0.00809500
H	6.17265900	2.54709000	2.05570000
C	1.95412500	0.70391500	2.13819400
C	1.68623200	-0.35046100	3.02524000
C	1.00328800	1.72453700	1.98959300
C	0.48538300	-0.38810900	3.73729100
H	2.42330800	-1.13567700	3.16039500
C	-0.20124000	1.68139100	2.69523300
H	1.20583900	2.54893300	1.31330500
C	-0.46273100	0.62612200	3.57202600
H	0.29401800	-1.20575100	4.42665400
H	-0.93095100	2.47358100	2.55989000
H	-1.39438500	0.59895400	4.12990900
C	-2.38198100	2.62201700	0.00092300
C	-2.80881700	3.85385500	0.52011900
C	-1.18196000	2.53909200	-0.72408800
C	-2.04570400	4.99818900	0.31650800
H	-3.72860000	3.89545900	1.09232800
C	-0.41509200	3.67687300	-0.93398800
H	-0.85264400	1.57422000	-1.09143800
C	-0.85641200	4.89461900	-0.40886400
H	-2.35495200	5.95669700	0.71444100
H	0.51774100	3.63021200	-1.48170800
N	-0.04076500	6.08959300	-0.61200100
O	-0.43143300	7.15297800	-0.12224000
O	1.00276300	5.97953300	-1.26275200
H	-1.56563600	-4.82174100	1.74929800
H	-3.32570400	-1.61759400	3.17463900

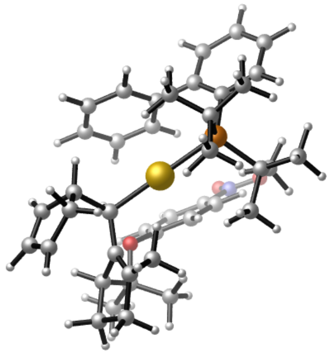

Xb

E (opt) = -2350.89185843 Hartrees
G (opt) = -2350.129395 Hartrees
E (SP) = -2351.37558918 Hartrees

Au	-0.03365800	-1.07531900	0.07322800
P	2.18880900	-1.39024400	-0.70158100
C	-2.06351600	-0.90308700	0.74924400
H	-2.04651700	0.03482400	1.30286100
C	-2.30640100	-2.09208500	1.85978800
H	-1.41984900	-2.17603300	2.48089000
C	-2.83501400	-3.38952200	1.24208600
C	-3.08708000	-0.82625500	-0.30999400
C	-3.32615500	-1.68649800	-1.34976400
C	2.56513800	-3.25633800	-0.56546400
C	2.28753000	-0.72097600	-2.49913200
C	-4.29362200	-3.09555500	1.09392400
H	-4.97565100	-3.68424400	0.49040900
C	-4.65821800	-2.07062900	1.95051600
H	-5.66675700	-1.71451800	2.12046800
C	-4.58650800	-1.31686800	-2.09542000
H	-4.32500000	-0.89098900	-3.07446400
H	-5.20700700	-2.19684300	-2.30334900
C	-4.14298800	0.28152600	-0.29325600
H	-4.48376200	0.43690200	0.73917000
C	-2.45782300	-2.76620300	-1.91066100
H	-1.51241200	-2.86725500	-1.37585800
H	-2.97497200	-3.73338300	-1.92644600
H	-2.22602400	-2.52553700	-2.95719200
C	-5.27793900	-0.29167100	-1.17689300
H	-5.99161200	-0.80922600	-0.52871300
H	-5.83293600	0.46761600	-1.73220200
C	-3.61708400	1.66751400	-0.77072000
C	-3.11559600	1.64879800	-2.21420400
H	-3.94789800	1.43791800	-2.88976500
H	-2.70377900	2.62061000	-2.49805400
H	-2.34597400	0.88704700	-2.35864300
C	-4.67515000	2.75619200	-0.57394200
H	-5.01549600	2.77508700	0.46575100
H	-4.25916800	3.73713600	-0.82247400

H	-5.53980500	2.58886400	-1.22129400
O	-2.51492100	1.94765900	0.16853000
H	-2.33308700	-3.72614000	0.34004700
C	-3.49275000	-1.51675000	2.49711000
C	0.90557200	-0.93152100	-3.16051600
H	0.12415500	-0.34824700	-2.66442000
H	0.96243600	-0.59693100	-4.20320100
H	0.59756900	-1.97966900	-3.16177500
C	2.54327400	0.79452700	-2.37749500
H	1.88802800	1.24929700	-1.62968400
H	3.57625200	1.01881000	-2.10130400
H	2.33914500	1.27210900	-3.34283700
C	3.35115100	-1.36361600	-3.40679800
H	3.15055500	-2.42221800	-3.58930600
H	3.32563800	-0.85357500	-4.37738400
H	4.36706500	-1.26245900	-3.02112700
C	1.59001700	-4.02509500	-1.47663900
H	1.72039100	-5.10040900	-1.30531100
H	0.54670700	-3.77674800	-1.25917300
H	1.77882600	-3.83531700	-2.53621400
C	4.01203800	-3.65435200	-0.90419000
H	4.09990400	-4.74336800	-0.81058000
H	4.29870100	-3.38865400	-1.92249500
H	4.72918200	-3.20949800	-0.21052600
C	2.29356800	-3.63954800	0.90499400
H	2.95800900	-3.10461600	1.59029100
H	1.25852500	-3.43667300	1.19745700
H	2.47984200	-4.71275000	1.03094700
C	3.53569400	-0.54711500	0.25202600
C	3.32257100	0.14700300	1.46824500
C	4.84034600	-0.56836600	-0.28252600
C	4.41292700	0.78460000	2.08746000
C	5.90762700	0.06786900	0.34512600
H	5.03292000	-1.08456100	-1.21285800
C	5.69260200	0.75195900	1.54100500
H	4.23787300	1.31545200	3.01858800
H	6.89632400	0.03032000	-0.10234200
H	6.51181500	1.25699600	2.04436900
C	2.01267400	0.28135800	2.17986800
C	1.57004600	-0.70977800	3.06904800
C	1.26580900	1.46223100	2.05261600
C	0.39245400	-0.53104100	3.79887500
H	2.15300700	-1.61681900	3.19256600
C	0.08463000	1.63702800	2.77708400
H	1.60624700	2.24011300	1.37675300
C	-0.35584900	0.64045200	3.65099400
H	0.06405200	-1.30402000	4.48817900
H	-0.48724500	2.55159100	2.65522200
H	-1.26992500	0.78019400	4.22131000
C	-1.62149900	2.94806300	-0.06860800
C	-1.80044800	4.19842000	0.54327300
C	-0.47087400	2.69558800	-0.83257700
C	-0.84148800	5.19369700	0.39159100
H	-2.68777800	4.37089500	1.14172700
C	0.48819000	3.68598300	-0.99611600
H	-0.33715000	1.71352900	-1.27069200
C	0.29174200	4.92484800	-0.37962500
H	-0.96032100	6.16250500	0.86056900
H	1.38432200	3.50861200	-1.57720100
N	1.30739400	5.96268000	-0.53915900
O	1.13508000	7.04182700	0.03469500
O	2.29149800	5.71204600	-1.24150900
H	-2.73651900	-4.19780700	1.98385000
H	-3.45660600	-0.69384100	3.20255700

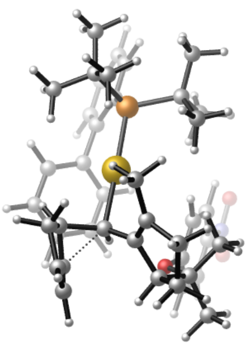

TS$_{Xb\text{-}XIa}$

E (opt) = -2350.88984334 Hartrees
G (opt) = -2350.126020 Hartrees
E (SP) = -2351.37292383 Hartrees

Au	-0.09920000	-1.04640000	0.12333400
P	2.05098400	-1.53147300	-0.69589700
C	-2.21506100	-0.80954700	0.82926700
H	-1.93112400	0.18469700	1.18054300
C	-2.33464900	-1.90841800	1.98264100
H	-1.40065300	-2.11051500	2.49328600
C	-3.24854200	-3.10699000	1.70380600
C	-3.15691700	-0.77452900	-0.31463600
C	-3.35265000	-1.71544200	-1.27019900
C	2.31771900	-3.40940300	-0.48366200
C	2.13486800	-0.94233900	-2.52115900
C	-4.63124300	-2.51385200	1.76194500
H	-5.52314500	-3.05358900	1.46311700
C	-4.59538100	-1.25761900	2.26939800
H	-5.44469000	-0.61063600	2.45237100
C	-4.48120900	-1.33637000	-2.20031900
H	-4.08107300	-1.05181300	-3.18394500
H	-5.15854700	-2.18063200	-2.37885400
C	-4.09776200	0.41709300	-0.52219400
H	-4.54059100	0.70943000	0.43739900
C	-2.56483000	-2.95355800	-1.55404500
H	-1.75989500	-3.11339000	-0.83515900
H	-3.20933600	-3.84133700	-1.57449600
H	-2.11207400	-2.87767100	-2.55195900
C	-5.17136600	-0.15904100	-1.48115400
H	-6.00177500	-0.54006700	-0.87888900
H	-5.58416400	0.57826600	-2.17348800
C	-3.40549900	1.69848100	-1.06376700
C	-2.74545100	1.48791000	-2.42511800
H	-3.51036500	1.25736800	-3.17031300
H	-2.22877200	2.39176600	-2.75738000
H	-2.02972100	0.66312400	-2.39483000
C	-4.37622500	2.88058800	-1.10328000
H	-4.83641000	3.03177700	-0.12216400
H	-3.84812100	3.79633000	-1.38597100
H	-5.16909600	2.71577100	-1.83747600
O	-2.39645700	1.99102300	-0.02198500
H	-3.03907100	-3.65001000	0.78375800
C	-3.22141400	-0.87172000	2.48780700
C	0.72835400	-1.10811100	-3.14007700
H	-0.01224700	-0.47290500	-2.64620000
H	0.77463700	-0.81023800	-4.19406300
H	0.37173800	-2.13997200	-3.09994500
C	2.47993800	0.55939300	-2.47089100
H	1.87729700	1.08193800	-1.72392300
H	3.53234100	0.73285000	-2.23474700
H	2.27184700	1.00815000	-3.44881800
C	3.14202300	-1.68080800	-3.42078400
H	2.88988300	-2.73658500	-3.54380400
H	3.11211700	-1.21866200	-4.41468700
H	4.17111100	-1.60684200	-3.06567100
C	1.29471500	-4.15668800	-1.35773000
H	1.35224300	-5.22755200	-1.12996100

H	0.27110800	-3.82834400	-1.15907100
H	1.49653200	-4.03541100	-2.42468800
C	3.73987600	-3.89158200	-0.82156200
H	3.76600800	-4.98204300	-0.71043300
H	4.03647400	-3.65854200	-1.84523600
H	4.48434400	-3.47704900	-0.13787400
C	2.04325600	-3.72133100	1.00204100
H	2.72823000	-3.18046400	1.66183700
H	1.01608800	-3.47651900	1.29027800
H	2.19593900	-4.79379200	1.17017900
C	3.45632100	-0.72210100	0.19599700
C	3.31327000	0.04383200	1.37927500
C	4.74286300	-0.84646100	-0.36757000
C	4.45389900	0.65108000	1.93539000
C	5.86010500	-0.24122800	0.20056600
H	4.88238100	-1.42148700	-1.27229300
C	5.71476700	0.51686600	1.36156200
H	4.33429400	1.23833100	2.84083600
H	6.83286600	-0.36048000	-0.26691400
H	6.57360700	1.00003200	1.81803300
C	2.03533200	0.28868200	2.11895200
C	1.53598300	-0.64928000	3.03632500
C	1.38549800	1.52518200	1.99036700
C	0.40015800	-0.35989200	3.79587700
H	2.04392500	-1.59984600	3.16080800
C	0.24377000	1.81049800	2.74268400
H	1.77317400	2.26139400	1.29424900
C	-0.25001700	0.86919000	3.64903400
H	0.02809400	-1.09067900	4.50834700
H	-0.25312300	2.76798200	2.62046700
H	-1.12645700	1.09703700	4.24857200
C	-1.45733700	2.96134800	-0.20441700
C	-1.63403700	4.21428400	0.40194100
C	-0.27202500	2.67651500	-0.89920800
C	-0.63590400	5.17812900	0.31420000
H	-2.55139300	4.41250800	0.94477900
C	0.72835800	3.63360800	-0.99731400
H	-0.14460200	1.69403400	-1.33542400
C	0.53450500	4.87552900	-0.38653100
H	-0.75213000	6.14903400	0.77942800
H	1.65210600	3.42519100	-1.52297100
N	1.59336600	5.87852000	-0.47123200
O	1.39785400	6.97958800	0.05085000
O	2.63566200	5.57646100	-1.06008700
H	-3.12836700	-3.82887500	2.52425200
H	-2.89774800	-0.02098000	3.07187200

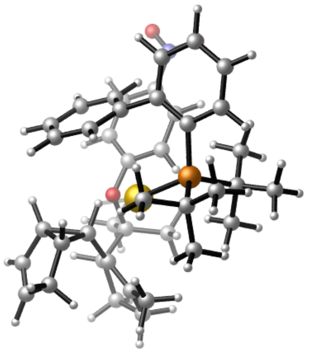

XIb

E (opt) = -2350.89757419 Hartrees
G (opt) = -2350.131779 Hartrees
E (SP) = -2351.37991552 Hartrees

Au	0.04687800	-1.01838200	0.21876100
P	2.15015000	-1.51741800	-0.62211500
C	-2.40352100	-0.85701000	0.93431500
H	-1.65864700	-0.03408800	0.89793400
C	-2.25117900	-1.94144800	2.02372500
H	-1.27446900	-2.08355700	2.47174000
C	-3.22468500	-3.12419500	2.01612500
C	-3.24863100	-0.96824700	-0.29056000
C	-3.27786500	-1.96250600	-1.19888900
C	2.46985900	-3.36346100	-0.26951700
C	2.13604300	-1.05642800	-2.48431700
C	-4.57689400	-2.47954300	2.23340200
H	-5.50504200	-3.04127000	2.19639000
C	-4.47113600	-1.16066900	2.44533700
H	-5.29254800	-0.47687000	2.63189400
C	-4.35940600	-1.73279600	-2.23303700
H	-3.91545100	-1.46520800	-3.20192000
H	-4.94936900	-2.64141500	-2.40583900
C	-4.25328900	0.13086000	-0.63275400
H	-4.78969200	0.44661800	0.26895300
C	-2.37989900	-3.15367900	-1.31429700
H	-1.66147000	-3.21241400	-0.49476400
H	-2.95663100	-4.08675600	-1.34563500
H	-1.81552600	-3.10586600	-2.25517900
C	-5.20038600	-0.58587900	-1.62889100
H	-6.03460200	-1.01123300	-1.06242800
H	-5.62630800	0.07979100	-2.38404900
C	-3.60157700	1.41945500	-1.21121900
C	-2.92972600	1.19714600	-2.56493300
H	-3.68233000	0.94106100	-3.31393800
H	-2.42631300	2.10510100	-2.90614400
H	-2.19934500	0.38609000	-2.51446200
C	-4.61373700	2.56307600	-1.28289900
H	-5.05200100	2.74716900	-0.29750700
H	-4.13300900	3.48317300	-1.62926100
H	-5.42025600	2.32238300	-1.98123000
O	-2.59770200	1.76592400	-0.18232800
H	-3.19176100	-3.71239400	1.09491500
C	-3.05407100	-0.71097900	2.34060400
C	0.72289600	-1.33702700	-3.04180600
H	-0.03010300	-0.70068000	-2.56806800
H	0.72244200	-1.11371600	-4.11472500
H	0.41891300	-2.37819200	-2.91613800
C	2.40618400	0.46022200	-2.55999200
H	1.78389600	1.01864400	-1.85529300
H	3.45078300	0.70253300	-2.35289800
H	2.16900000	0.80962200	-3.57102300
C	3.15834000	-1.81264300	-3.35190200
H	2.96247800	-2.88649200	-3.38080400
H	3.07331300	-1.43469700	-4.37742200
H	4.19085100	-1.65132600	-3.03846100
C	1.45215700	-4.19911700	-1.06596000
H	1.54424700	-5.24705000	-0.75838600
H	0.42366500	-3.88531800	-0.87141500
H	1.63139400	-4.15484800	-2.14269600
C	3.90016200	-3.82471200	-0.60614300
H	3.96029500	-4.90254600	-0.41662800
H	4.16645400	-3.65961600	-1.65137800
H	4.64522800	-3.34023600	0.02897900
C	2.23657200	-3.57278200	1.24059100
H	2.91856400	-2.96687300	1.84447400
H	1.20758700	-3.34357400	1.53532500
H	2.42797800	-4.62515800	1.47941400
C	3.52333100	-0.55810300	0.15671700
C	3.36599100	0.37402400	1.21347300
C	4.79950400	-0.70398600	-0.42546600
C	4.48153900	1.13364800	1.61186000
C	5.89279600	0.04668500	-0.00402400
H	4.94880100	-1.40494000	-1.23425400
C	5.73068700	0.98063100	1.01822000
H	4.35251900	1.85120700	2.41635500
H	6.85795600	-0.09473800	-0.48065900
H	6.56892400	1.58359300	1.35438900
C	2.10456600	0.64837300	1.96455100
C	1.57605100	-0.28069400	2.87675500
C	1.48900500	1.90306500	1.84210900
C	0.45011500	0.03766500	3.64011900
H	2.05822700	-1.24457200	2.99498100
C	0.35576500	2.21638500	2.59582900
H	1.89866600	2.63211400	1.15081400
C	-0.16467600	1.28573300	3.49820000
H	0.05653100	-0.68642700	4.34746000

H	-0.11554700	3.18665800	2.47580100
H	-1.03615900	1.53472100	4.09643000
C	-1.70815200	2.78119500	-0.36059700
C	-1.93800300	4.01317000	0.27024500
C	-0.51170000	2.56149400	-1.06146200
C	-0.97730600	5.01605100	0.21293900
H	-2.86358400	4.16073600	0.81515900
C	0.45247700	3.55767200	-1.12708700
H	-0.34238200	1.59958200	-1.52882400
C	0.21037900	4.77326100	-0.48244600
H	-1.13180900	5.96918900	0.70333700
H	1.38635600	3.39689100	-1.65134100
N	1.24080500	5.80795600	-0.51478900
O	0.98826100	6.89983500	0.00156900
O	2.31909700	5.53824700	-1.05273700
H	-2.97829000	-3.81330900	2.83491600
H	-2.67239400	0.09079300	2.96266800

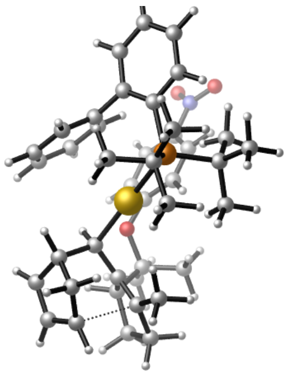

TS$_{Xb-XIIb}$

E (opt) = -2350.89006190 Hartrees
G (opt) = -2350.125583 Hartrees
E (SP) = -2351.37271615 Hartrees

Au	-0.28837300	-1.00174300	0.13374300
P	1.80122300	-1.81790800	-0.66097500
C	-2.23581800	-0.46961600	0.86286500
H	-2.07077600	0.41911300	1.47156900
C	-2.77802800	-1.63119400	1.85141400
H	-1.99735600	-1.89640000	2.56071800
C	-3.38112300	-2.79163200	1.05579000
C	-3.16120400	-0.16419900	-0.21290900
C	-3.69760700	-1.03356700	-1.17455100
C	1.76398300	-3.71697600	-0.47814000
C	2.01033200	-1.24507900	-2.48279700
C	-4.67449700	-2.16598200	0.59864400
H	-5.35643900	-2.62712500	-0.10708900
C	-5.08453000	-1.27584300	1.63503700
H	-6.06937000	-0.83251200	1.71918000
C	-4.84112100	-0.36758900	-1.91324400
H	-4.49605000	-0.08071100	-2.91578100
H	-5.68415600	-1.05122700	-2.06148800
C	-3.91924600	1.15876100	-0.22903300
H	-4.16975800	1.42372500	0.80624300
C	-3.00746000	-2.16800600	-1.87967300
H	-2.05425300	-2.43064200	-1.42258700
H	-3.63933900	-3.06002600	-1.94871900
H	-2.80371300	-1.84463800	-2.90879900
C	-5.19591400	0.85802200	-1.05476100
H	-6.00791500	0.60788500	-0.36686500
H	-5.53043000	1.70526900	-1.65663500
C	-3.07604800	2.34806500	-0.77737000
C	-2.63427100	2.14288200	-2.22667700
H	-3.51045300	2.09675300	-2.87816300
H	-2.01881600	2.97962700	-2.56660000
H	-2.06084400	1.21940800	-2.34229400
C	-3.82997200	3.66952400	-0.61410100
H	-4.12417700	3.81772900	0.42903800
H	-3.19484300	4.50617300	-0.91969500
H	-4.72872900	3.69120400	-1.23577600
O	-1.91941500	2.36881800	0.12995300
H	-2.74229000	-3.20855600	0.28448700
C	-3.99825800	-0.99393700	2.42711100
C	0.60136600	-1.16413100	-3.11512800
H	-0.02345500	-0.41646800	-2.61689400
H	0.70534800	-0.86897700	-4.16616600
H	0.07261700	-2.11955200	-3.08648500
C	2.60427400	0.17715600	-2.43123300
H	2.09700600	0.79823000	-1.68873400
H	3.66965000	0.16973900	-2.19026300
H	2.48205600	0.65215100	-3.41160400
C	2.88684800	-2.14118800	-3.37423100
H	2.46008100	-3.13821000	-3.50484300
H	2.95328100	-1.67934700	-4.36682700
H	3.90722600	-2.24416600	-3.00150600
C	0.61938600	-4.26829000	-1.34927700
H	0.52633600	-5.34605100	-1.17057700
H	-0.33994300	-3.80482500	-1.09917400
H	0.80480600	-4.12505000	-2.41664300
C	3.08162700	-4.43000300	-0.83340800
H	2.93444300	-5.50838200	-0.69858600
H	3.39213000	-4.26974000	-1.86623400
H	3.89759400	-4.12944100	-0.17180100
C	1.45128600	-4.00523500	1.00525000
H	2.23854700	-3.62730400	1.66439500
H	0.49804100	-3.56801700	1.31787500
H	1.39142800	-5.09068500	1.14903100
C	3.30893800	-1.23956500	0.24329000
C	3.25964000	-0.43572600	1.40978200
C	4.57159300	-1.54379900	-0.30481300
C	4.46436600	0.04874800	1.95087500
C	5.75395700	-1.06529100	0.25329100
H	4.63973500	-2.15289800	-1.19591000
C	5.70011100	-0.25425100	1.38639100
H	4.41599400	0.67040800	2.83992600
H	6.70636600	-1.31893200	-0.20250200
H	6.61079400	0.13715900	1.83021800
C	2.01930100	-0.04535000	2.14984300
C	1.38947800	-0.94560100	3.02364100
C	1.53646600	1.26974500	2.06751700
C	0.28818300	-0.54428300	3.78274900
H	1.76868800	-1.95787900	3.11158600
C	0.43063500	1.66808200	2.82206000
H	2.02277800	1.97582700	1.40188300
C	-0.19737400	0.76244900	3.68009800
H	-0.18459000	-1.25054900	4.45953100
H	0.06285800	2.68549900	2.73592600
H	-1.05364700	1.07411100	4.27162200
C	-0.84582800	3.17273200	-0.11332600
C	-0.74075300	4.40337400	0.55232100
C	0.20014900	2.72347200	-0.93389200
C	0.39955300	5.18371500	0.40057500
H	-1.55291400	4.72636900	1.19362400
C	1.34104800	3.49825300	-1.09577400
H	0.11066900	1.75945800	-1.41909200
C	1.42724000	4.72118600	-0.42504600
H	0.50171000	6.13262200	0.91209600
H	2.16127900	3.16328100	-1.71851200
N	2.63291600	5.53166200	-0.58146300
O	2.68939100	6.61355700	0.00976700
O	3.53926600	5.09547200	-1.29724400
H	-3.64104000	-3.60365900	1.75021800
H	-3.98165800	-0.29684900	3.25813400

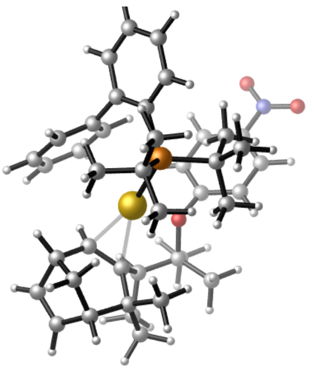

XIIb

E (opt) = -2350.93297690 Hartrees
G (opt) = -2350.163901 Hartrees
E (SP) = -2351.41420741 Hartrees

Au	-0.90181300	-0.38849200	-0.01652800
P	0.47011400	-2.05866500	-0.98849300
C	-2.59225400	0.60440300	1.18224400
H	-2.05268700	0.83693800	2.09915400
C	-3.79712800	-0.32366900	1.33312000
H	-3.57597700	-1.16203800	1.99475000
C	-4.35313900	-0.70415400	-0.04741000
C	-2.50559400	1.50224500	0.13021600
C	-3.58547400	1.52970100	-0.98293700
C	-0.69228300	-3.42234900	-1.64235700
C	1.52648400	-1.22445500	-2.35594300
C	-4.83581400	0.68559700	-0.52469100
H	-5.54019600	0.64836400	-1.36153500
C	-5.46308500	1.21256200	0.75839700
H	-6.21644300	1.99306800	0.78513500
C	-3.89819000	3.05506500	-1.09454500
H	-3.51639500	3.43551100	-2.04539400
H	-4.97497400	3.24588100	-1.08719600
C	-1.92154500	2.89890600	0.38915400
H	-1.68128700	2.94615700	1.45454700
C	-3.11779800	0.98431600	-2.34510000
H	-2.95888800	-0.09441400	-2.32181300
H	-3.87974400	1.19821200	-3.10289300
H	-2.18476000	1.44813100	-2.66513700
C	-3.18728600	3.74626200	0.08657200
H	-3.83220600	3.72271000	0.97017300
H	-2.95556400	4.79437800	-0.11886700
C	-0.62808600	3.41494400	-0.30844600
C	-0.73766000	3.71422500	-1.80176700
H	-1.53643700	4.43394600	-1.99398200
H	0.18991800	4.16155200	-2.16603900
H	-0.93168500	2.80837300	-2.37482600
C	-0.14402400	4.66201200	0.44193600
H	0.03364100	4.44043800	1.49834200
H	0.78387600	5.04336600	0.00741600
H	-0.89260500	5.45562600	0.37620200
O	0.32866300	2.31665800	-0.09744000
H	-3.61248400	-1.17285700	-0.69757600
C	-4.89561200	0.62910000	1.82324800
C	0.69586500	-0.06336800	-2.94694800
H	0.50677100	0.71539100	-2.20456100
H	1.26194400	0.38751900	-3.77046500
H	-0.26319900	-0.39954800	-3.34744200
C	2.77055100	-0.63930400	-1.65893700
H	2.50469200	-0.05667800	-0.77194600
H	3.47875900	-1.41595100	-1.36145400
H	3.27990400	0.03430700	-2.35609800
C	1.95843500	-2.14531400	-3.51143900
H	1.10387600	-2.51798700	-4.08081700
H	2.58061500	-1.55862600	-4.19778100
H	2.55897500	-2.99644600	-3.18497900
C	-1.58581100	-2.81827100	-2.73978600
H	-2.34300800	-3.55738700	-3.02583400
H	-2.10925000	-1.92488600	-2.38930600
H	-1.02059000	-2.55775800	-3.63807900
C	0.02710900	-4.67212000	-2.18039900
H	-0.73395900	-5.36891800	-2.55105300
H	0.70255900	-4.45298500	-3.00804700
H	0.58739800	-5.18834300	-1.39757400
C	-1.56912800	-3.84992600	-0.44649100
H	-0.96622000	-4.25974300	0.36948100
H	-2.16456800	-3.01956900	-0.05587100
H	-2.25915500	-4.63418400	-0.77890900
C	1.64219200	-2.91291600	0.15319100
C	1.65070000	-2.73702400	1.55665600
C	2.59970400	-3.77502800	-0.42024100
C	2.61538400	-3.42048200	2.31952400
C	3.54441400	-4.44462100	0.35134800
H	2.61691200	-3.92516200	-1.49120900
C	3.55415500	-4.26474400	1.73422100
H	2.61694900	-3.27692700	3.39582200
H	4.26690900	-5.09792200	-0.12835800
H	4.28571800	-4.77581100	2.35312200
C	0.71873000	-1.87066700	2.34423800
C	-0.50572100	-2.37159000	2.81266400
C	1.13780700	-0.59846100	2.76361200
C	-1.29236500	-1.61520100	3.68442700
H	-0.82903600	-3.36214800	2.50915700
C	0.34732000	0.15907900	3.63021800
H	2.08942300	-0.21027900	2.41458300
C	-0.86641600	-0.35041500	4.09960900
H	-2.23148700	-2.02027300	4.05006100
H	0.68731400	1.14027600	3.94998500
H	-1.47410700	0.23132800	4.78675900
C	1.67404200	2.54574700	-0.06197800
C	2.32250000	2.42744200	1.17640200
C	2.41770600	2.80021300	-1.22468400
C	3.70276500	2.55603100	1.26102200
H	1.72594900	2.22855000	2.05848300
C	3.79784400	2.94342200	-1.14727600
H	1.92058900	2.85481300	-2.18409700
C	4.42509800	2.81772700	0.09405900
H	4.21770500	2.46359200	2.20899800
H	4.38867600	3.13438800	-2.03435900
N	5.87802300	2.95358500	0.17304800
O	6.41481900	2.83615500	1.27819400
O	6.50006700	3.17797000	-0.86905900
H	-5.20087400	-1.38746500	0.06229100
H	-5.08065700	0.85144300	2.86902000

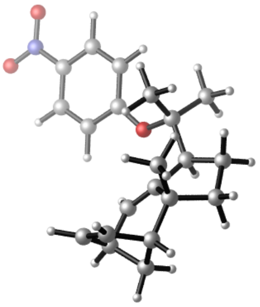

7a''

E (opt) = -1095.66255274 Hartrees
G (opt) = -1095.288937 Hartrees
E (SP) = -1095.94067521 Hartrees

C	-1.82915900	1.22666000	-0.78261000
H	-0.92177200	1.32430400	-1.37295000
C	-2.79469000	2.40655900	-0.70147200
H	-2.47302300	3.21630900	-1.36237300
C	-3.05715300	2.85775000	0.73468500
C	-2.20012900	0.02642700	-0.31368000
C	-3.55164500	-0.20873100	0.37873700

C	-4.02834500	2.10965700	1.26749600
H	-4.38681300	2.16016300	2.29029100
C	-4.47724500	1.05193000	0.25557900
H	-5.52292800	0.74523500	0.37128100
C	-4.03193900	-1.45843400	-0.39971500
H	-4.84587500	-1.98499200	0.11117200
H	-4.39466700	-1.16190200	-1.39130800
C	-1.60050100	-1.31070100	-0.75870300
H	-1.41192600	-1.23541400	-1.83581600
C	-3.40918300	-0.58152000	1.87468500
H	-2.82359400	0.15912100	2.42427900
H	-4.40415000	-0.63799500	2.33321900
H	-2.93056200	-1.55147200	2.01441800
C	-2.75928700	-2.32594500	-0.54008500
H	-2.83007000	-3.04293300	-1.36233500
H	-2.60264600	-2.90767000	0.37334700
C	-0.22365900	-1.71547200	-0.16365000
C	-0.18543400	-1.64477900	1.36031300
H	-0.89562100	-2.36137700	1.77877000
H	0.79927200	-1.89327000	1.75917500
H	-0.45758100	-0.64326000	1.70103600
C	0.18294400	-3.08814000	-0.70670600
H	0.19336100	-3.07496700	-1.80078200
H	1.16783800	-3.40141100	-0.35696100
H	-0.53484700	-3.84413200	-0.37712700
O	0.64182400	-0.67075500	-0.74280600
H	-2.48111100	3.62807900	1.23853000
C	-4.18784300	1.81164200	-1.05675900
H	-4.92815700	2.60532200	-1.20617600
H	-4.16347600	1.17596900	-1.94667800
C	1.92126900	-0.39980200	-0.42607200
C	2.35801600	0.87924000	-0.84009300
C	2.83556800	-1.25873100	0.21762600
C	3.65764500	1.29495600	-0.61874600
H	1.64428000	1.53065700	-1.33253900
C	4.14050300	-0.84033900	0.44514900
H	2.54719800	-2.24991800	0.53403800
C	4.54702300	0.42901300	0.03010700
H	3.99019400	2.27672900	-0.93227700
H	4.84780900	-1.49624400	0.93752300
N	5.91283200	0.85606400	0.27191200
O	6.24937800	1.98574800	-0.10331100
O	6.68236200	0.07315500	0.84241100

H	5.19134900	-2.16451300	-1.42065600
C	3.67118500	2.12386300	0.65771200
H	3.31660100	2.86135900	1.39250200
H	4.75745800	2.26572300	0.58471300
C	1.86389500	1.16988800	-0.73093000
H	1.81659300	0.68548700	-1.71286200
C	3.99703900	0.12683300	2.29406000
H	3.55894200	-0.82712100	2.59268800
H	5.06618000	-0.02987300	2.09863300
H	3.93496000	0.81405000	3.14854900
C	2.96706700	2.26447000	-0.70913400
H	3.68136800	2.04965400	-1.50983800
H	2.58731300	3.27383700	-0.88510600
C	0.41884000	1.71610600	-0.49277200
C	0.27776200	2.38044600	0.87636700
H	0.97842600	3.21656300	0.94315100
H	-0.72164800	2.78167000	1.04785500
H	0.51107000	1.67160900	1.67410700
C	0.00403900	2.63349300	-1.64728800
H	0.10380300	2.10602200	-2.60079300
H	-1.02833100	2.97429800	-1.55113500
H	0.63903300	3.52205900	-1.67617300
O	-0.37336300	0.47916400	-0.56457600
H	2.14403700	-3.18078400	1.40607000
C	2.92248300	-2.08975700	-1.72688200
H	2.80971800	-2.86410800	-2.49804900
H	2.89252000	-1.12694700	-2.25352000
C	-1.68526200	0.32061700	-0.32390600
C	-2.10178900	-1.03126800	-0.32060300
C	-2.64280000	1.33152800	-0.10184000
C	-3.42327200	-1.36932600	-0.10029100
H	-1.35316800	-1.79606800	-0.49602300
C	-3.97141800	0.99179800	0.12079500
H	-2.36906200	2.37572700	-0.10335600
C	-4.35839200	-0.34949900	0.12117700
H	-3.74091300	-2.40463600	-0.09536200
H	-4.71122600	1.76407500	0.29211100
N	-5.74784900	-0.69238100	0.35841300
O	-6.06763800	-1.88759500	0.34696300
O	-6.55407500	0.22361000	0.56293700

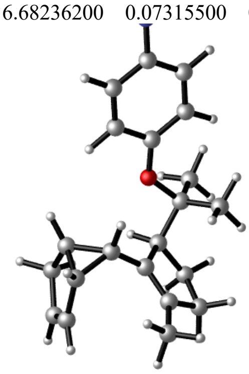

8a’

E (opt) = -1095.64858603 Hartrees
G (opt) = -1095.280715 Hartrees
E (SP) = -1095.92746241 Hartrees

C	1.75952900	-1.18237000	0.45177300
H	0.78634600	-1.18641400	0.93995600
C	1.83141400	-2.21266100	-0.65605700
H	0.91538100	-2.73537300	-0.91509800
C	2.53634300	-2.48134000	0.67185600
C	2.35537000	0.18031700	0.32993800
C	3.31433800	0.72367300	1.10218400
C	3.99332000	-2.49747900	0.34414400
H	4.76311000	-2.65046800	1.09422600
C	4.21459200	-2.26109800	-0.95497900

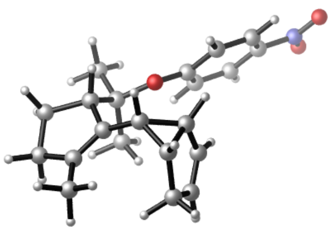

8a’ (turned)

E (opt) = -1095.64264743 Hartrees
G (opt) = -1095.272656 Hartrees
E (SP) = -1095.92061458 Hartrees

C	2.23209000	-1.21610900	-1.11681800
H	2.26270100	-1.17301700	-2.20862800
C	2.41251200	-2.64166100	-0.61675000
H	2.71926900	-3.38016600	-1.35184000
C	1.01750900	-2.02635400	-0.66605300
C	2.77363200	0.05180400	-0.53448300
C	4.01147500	0.23288700	-0.02915200
C	0.57996800	-1.92711700	0.75603300
H	-0.37180600	-1.49874300	1.04896000
C	1.50595200	-2.40469200	1.59673900
H	1.43960300	-2.39310600	2.68108200
C	4.27616800	1.68213600	0.31371400

H	4.19815200	1.84493800	1.39891100
H	5.29030400	1.99048400	0.03076400
C	2.06612600	1.40223100	-0.76028900
H	1.76067100	1.45821200	-1.81388800
C	5.12746900	-0.75466400	0.13602900
H	4.90963500	-1.72259700	-0.31592400
H	6.03516800	-0.35852400	-0.33837400
H	5.37961400	-0.90945100	1.19390000
C	3.19186800	2.44056300	-0.47267100
H	3.60715300	2.78098100	-1.42620100
H	2.84010000	3.32977000	0.05319700
C	0.75355700	1.68626100	0.03208000
C	0.95647800	1.47596000	1.53389100
H	1.79294800	2.09841200	1.86425000
H	0.08879100	1.76477000	2.12948900
H	1.20086100	0.43457900	1.74605700
C	0.21458800	3.08400800	-0.29881600
H	0.18580500	3.23629600	-1.38231100
H	-0.79540000	3.22348800	0.09114700
H	0.84174200	3.85961800	0.14539900
O	-0.16401900	0.70023500	-0.55746200
H	0.28696400	-2.28739300	-1.42688500
C	2.70655500	-2.95198600	0.85824500
H	2.79857900	-4.03774400	1.00507100
H	3.64004400	-2.51636900	1.22462500
C	-1.47528400	0.54462900	-0.31765700
C	-2.12148700	-0.32353700	-1.22892100
C	-2.22609400	1.11390200	0.73154000
C	-3.46400300	-0.62284000	-1.09964900
H	-1.53194900	-0.74832500	-2.03333400
C	-3.57770400	0.81793000	0.85870800
H	-1.77211800	1.78025800	1.44863100
C	-4.19124400	-0.04628500	-0.04987500
H	-3.95690400	-1.28909800	-1.79669500
H	-4.15859400	1.25138400	1.66351100
N	-5.60210300	-0.35051500	0.09361600
O	-6.11990800	-1.12799600	-0.71772800
O	-6.22824800	0.17932800	1.02002700

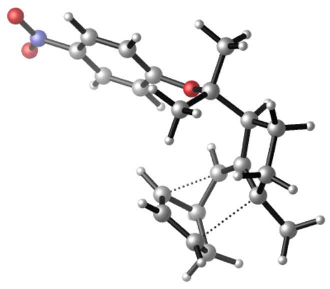

TS$_{8a'\text{-}7a'}$

E (opt) = -1095.59536415 Hartrees
G (opt) = -1095.225923 Hartrees
E (SP) = -1095.87439656 Hartrees

C	1.75850200	-1.04623300	-1.10246900
H	0.86966400	-0.86432900	-1.69372100
C	1.91243900	-2.48374800	-0.66005100
H	1.53078500	-3.21140300	-1.37616600
C	0.99800300	-2.04893000	0.46138400
C	2.49955400	0.06018400	-0.68372700
C	3.73411500	0.00265400	-0.00496100
C	1.77467200	-1.80101600	1.60656800
H	1.39134100	-1.35858300	2.52016000
C	3.11600000	-2.02279200	1.33001600
H	3.92252900	-1.95358300	2.05119300
C	3.92973900	1.31435300	0.74430800
H	3.49910800	1.25645500	1.75078100
H	4.98333900	1.59117700	0.86023200
C	1.96852300	1.51350000	-0.74221300

H	1.84462600	1.77866400	-1.80001100
C	4.99550800	-0.63193900	-0.54986100
H	4.80276500	-1.30899000	-1.38268300
H	5.62952900	0.17553600	-0.94605300
H	5.59437100	-1.15453400	0.20537400
C	3.14991000	2.32043000	-0.12775800
H	3.80013500	2.66757600	-0.93769800
H	2.83379500	3.20616500	0.42847600
C	0.59153700	1.85499800	-0.09110600
C	0.58905000	1.55953300	1.40763900
H	1.35632300	2.16532600	1.89501600
H	-0.36027700	1.80047300	1.88655100
H	0.81495900	0.50906400	1.58251100
C	0.20314300	3.30422100	-0.41156200
H	0.20263600	3.46455500	-1.49401300
H	-0.79049500	3.54898500	-0.03114200
H	0.90901300	4.00460600	0.04089900
O	-0.33375700	0.97415600	-0.82048900
H	-0.07641800	-1.95325900	0.37418600
C	3.18186800	-2.90293500	0.09011200
H	3.07361100	-3.95512700	0.39686000
H	4.09425900	-2.84291700	-0.49500300
C	-1.59906300	0.66335000	-0.49214700
C	-2.10470900	-0.47877500	-1.15636900
C	-2.44548000	1.36855100	0.38925200
C	-3.39759100	-0.91740000	-0.93881900
H	-1.45247500	-1.00757800	-1.84210500
C	-3.74377900	0.92687600	0.61029500
H	-2.10959400	2.25987100	0.89754700
C	-4.21429000	-0.21164300	-0.04662100
H	-3.78039400	-1.79617200	-1.44272400
H	-4.39692700	1.46504800	1.28622700
N	-5.57033500	-0.66813100	0.19461900
O	-5.96541100	-1.67522200	-0.40553900
O	-6.27330300	-0.03262600	0.98996800

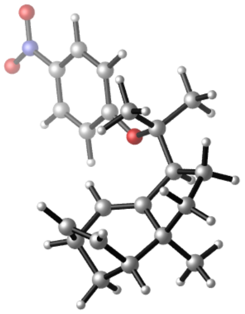

7a'

E (opt) = -1095.66570286 Hartrees
G (opt) = -1095.292598 Hartrees
E (SP) = -1095.94385963 Hartrees

C	1.56746700	-1.19879700	-0.16252500
H	0.48552500	-1.23827800	-0.10293700
C	2.35860700	-2.44961300	0.21609000
H	1.76364000	-3.35827800	0.08913100
C	2.86521900	-2.21949300	1.64661300
C	2.20909100	-0.04705700	-0.41088000
C	3.74138900	0.05268100	-0.40118200
C	4.01587900	-1.53552800	1.59844700
H	4.56807400	-1.15405000	2.45162800
C	4.39628000	-1.25901300	0.14916700
H	5.47857200	-1.22138100	-0.01615300
C	3.92750700	1.31875400	0.46248600
H	3.77051400	1.06778300	1.51738000
H	4.92891300	1.75316200	0.36884900
C	1.65042300	1.36887600	-0.54598400
H	1.50489900	1.58118700	-1.61293200
C	4.29251600	0.32684300	-1.82189300

H	4.08173000	-0.49810400	-2.50736100
H	3.86409000	1.23205800	-2.26245200
H	5.37957100	0.46403200	-1.77824400
C	2.81273700	2.27824100	-0.01223500
H	3.17055900	2.93459600	-0.81009000
H	2.48768600	2.93095200	0.80135900
C	0.27500700	1.67005400	0.10944900
C	0.26177900	1.34644200	1.60592000
H	1.07411800	1.88868200	2.09733000
H	-0.66696400	1.64842500	2.09100400
H	0.41922300	0.28071100	1.77612500
C	-0.13088800	3.11929100	-0.18404600
H	-0.11188700	3.30561300	-1.26219400
H	-1.13369000	3.34261200	0.18394600
H	0.55919000	3.81527000	0.29891900
O	-0.61598200	0.77556300	-0.64860600
H	2.30373800	-2.48541900	2.53730300
C	3.69823500	-2.45634600	-0.54649200
H	4.24924300	-3.38367100	-0.35622000
H	3.57278500	-2.34055800	-1.62570000
C	-1.90207800	0.48249900	-0.38476300
C	-2.39729500	-0.61699000	-1.12287200
C	-2.76566700	1.15754600	0.50179600
C	-3.70638100	-1.03757800	-0.98220100
H	-1.71989400	-1.12295500	-1.80235400
C	-4.08111500	0.73392400	0.64490400
H	-2.43032100	2.00813200	1.07582700
C	-4.54644700	-0.35709200	-0.09131800
H	-4.08449100	-1.88246300	-1.54430800
H	-4.75063200	1.24849600	1.32316300
N	-5.92112100	-0.79337000	0.06975500
O	-6.31165500	-1.75960700	-0.59670100
O	-6.64360400	-0.18205800	0.86653400

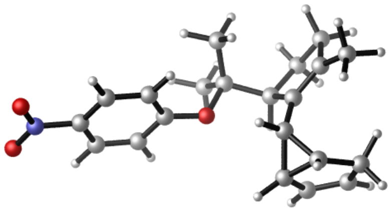

8a

E (opt) = -1095.64910218 Hartrees
G (opt) = -1095.281881 Hartrees
E (SP) = -1095.92754044 Hartrees

C	2.00639800	-1.17466300	0.69839600
H	1.17076700	-1.23012100	1.39443300
C	2.97401600	-2.33663000	0.82916200
H	2.83385900	-3.01639400	1.66420400
C	4.37393000	-2.21731100	0.21825400
C	2.42247500	0.21769000	0.35702200
C	3.30636700	0.96759100	1.04204000
C	4.11132500	-2.04034200	-1.26358600
H	4.90443200	-1.84528300	-1.97985500
C	2.80562800	-2.12405700	-1.55362400
H	2.37581700	-2.03696700	-2.54741400
C	3.49140100	2.33104700	0.41408700
H	3.02405100	3.10925900	1.03495000
H	4.55174300	2.60271200	0.32986100
C	1.82802900	0.98977200	-0.81866700
H	1.83292800	0.36770200	-1.72058200
C	4.02943400	0.61392300	2.30521500
H	3.80661100	-0.40149400	2.64058200
H	5.11607800	0.71056600	2.18172900

H	3.74953300	1.30573500	3.11182200
C	2.80645900	2.18766600	-0.96349800
H	3.55869400	1.92821100	-1.71508000
H	2.32475700	3.11064600	-1.29658700
C	0.34422300	1.41130900	-0.62352400
C	0.12982400	2.31787200	0.58757000
H	0.65005400	3.26778500	0.43889400
H	-0.93138700	2.54320000	0.72526300
H	0.51282100	1.85127700	1.49835900
C	-0.21392100	2.05281000	-1.89588300
H	-0.08874500	1.38205500	-2.75144000
H	-1.27807400	2.28134100	-1.78456600
H	0.30357100	2.99041900	-2.11421100
O	-0.33156800	0.11508800	-0.41257000
H	4.94399400	-1.37599500	0.63065600
C	1.97487300	-2.30333100	-0.32579000
C	-1.67611100	0.04070800	-0.22026500
C	-2.51969100	-0.22051600	-1.31304400
C	-2.21296700	0.11523000	1.07632700
C	-3.88449700	-0.39125100	-1.12019200
H	-2.08627300	-0.29433700	-2.30384400
C	-3.57665900	-0.05386500	1.27923400
H	-1.54891800	0.29639100	1.91353100
C	-4.39845900	-0.30250400	0.17647500
H	-4.54695500	-0.59385300	-1.95259000
H	-4.00582200	-0.00204100	2.27200600
N	-5.83169400	-0.48449400	0.38717700
O	-6.54440700	-0.70409300	-0.59710800
O	-6.26689500	-0.40964700	1.54051300
H	4.96739500	-3.12144900	0.41376300
H	1.09142100	-2.93559100	-0.35505900

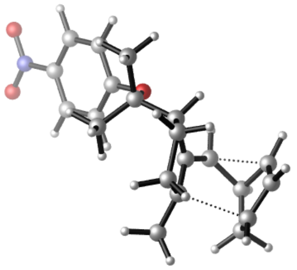

TS_{8a-7a}

E (opt) = -1095.60167330 Hartrees
G (opt) = -1095.233133 Hartrees
E (SP) = -1095.88099470 Hartrees

C	2.04513300	-1.09977400	-0.90589800
H	1.32062300	-1.03703000	-1.71212900
C	3.08106000	-2.18696100	-1.07306300
H	2.71731400	-3.10165800	-1.54056500
C	4.11991400	-2.38061500	0.04021400
C	2.09998200	-0.03939200	0.00055200
C	2.92406200	0.02423900	1.14239600
C	4.80170400	-1.01820000	0.07285900
H	5.53624300	-0.74620200	0.82278700
C	4.75599400	-0.50620500	-1.22042500
H	5.28981100	0.37472600	-1.56333200
C	3.20317600	1.48109900	1.48459700
H	2.67585100	1.76158600	2.40802500
H	4.26739900	1.66303700	1.67501400
C	1.63570800	1.35799600	-0.41895900
H	1.69179900	1.46814500	-1.50813900
C	2.85040400	-0.92514400	2.31664100
H	2.35427400	-1.86501200	2.07048900
H	3.82723600	-1.14439700	2.76334400
H	2.24745100	-0.44735300	3.10258500
C	2.68005600	2.27517400	0.26925000
H	3.49828800	2.45358900	-0.43541700

H	2.28053100	3.25322400	0.55186600
C	0.15865600	1.65659500	-0.05396800
C	-0.13501900	1.53063000	1.43946500
H	0.41857200	2.29898900	1.98528300
H	-1.19838600	1.68161100	1.64557300
H	0.15982600	0.55086700	1.82223900
C	-0.28805500	3.01939500	-0.58598100
H	-0.12263100	3.08196800	-1.66574100
H	-1.35069500	3.18301000	-0.38379700
H	0.26787700	3.82773200	-0.10271700
O	-0.55886900	0.61257400	-0.82235000
H	3.71043900	-2.73540900	0.98185400
C	3.74440300	-1.14744600	-1.95142900
C	-1.84736800	0.28840000	-0.53037600
C	-2.91092200	0.95620600	-1.15965300
C	-2.10805500	-0.78659000	0.33611200
C	-4.22246500	0.56946500	-0.91185800
H	-2.69508500	1.76565100	-1.84740700
C	-3.41497700	-1.18072500	0.58899600
H	-1.27211100	-1.30223600	0.79508400
C	-4.45963100	-0.49258700	-0.03542100
H	-5.05443800	1.07355000	-1.38760900
H	-3.63327000	-2.00579900	1.25561400
N	-5.83656900	-0.89799300	0.23161600
O	-6.74809200	-0.26377700	-0.30881100
O	-6.02792400	-1.85539900	0.98776200
H	4.84809200	-3.13610400	-0.29202500
H	3.46750300	-0.95105500	-2.97930300

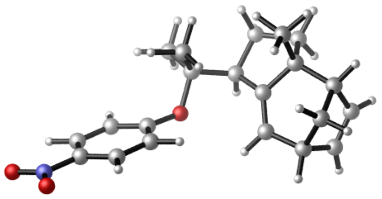

8a

E (opt) = -1095.66537441 Hartrees
G (opt) = -1095.293127 Hartrees
E (SP) = -1095.94314848 Hartrees

C	1.82656200	-1.13965300	-0.81472800
H	1.01647700	-1.13925300	-1.53951600
C	2.74541500	-2.35583600	-0.74302300
H	2.23336000	-3.28097200	-1.02114400
C	3.43690200	-2.39918700	0.63298500
C	2.14591800	-0.00959400	-0.16536900
C	3.39806400	0.11239400	0.71332500
C	4.33097600	-1.13510700	0.51739300
H	5.14240800	-1.09899300	1.25233100
C	4.82768600	-1.29077400	-0.91310600
H	5.73048900	-0.82210100	-1.29240800
C	3.94631200	1.47395100	0.21640900
H	4.68249600	1.91040100	0.90050400
H	4.43092900	1.34306500	-0.75818700
C	1.60166500	1.38103500	-0.49750900
H	1.58293200	1.47244100	-1.59047700
C	3.07027500	0.23022300	2.22211300
H	2.51667600	-0.63538200	2.59417600
H	4.00315100	0.30757500	2.79355300
H	2.47409900	1.11580000	2.44670700
C	2.68429200	2.35039200	0.05324000
H	2.84814500	3.19895800	-0.61653400
H	2.38419700	2.76414400	1.02120500
C	0.14886000	1.68235800	-0.06221700
C	-0.12427000	1.39895500	1.41213600

H	0.47162000	2.07301600	2.03210400
H	-1.17614200	1.57317400	1.65564500
H	0.13057700	0.36985700	1.67360800
C	-0.24735700	3.11481300	-0.42490900
H	-0.08946800	3.29781500	-1.49208700
H	-1.30030700	3.29564800	-0.19007200
H	0.34812500	3.83461200	0.14453300
O	-0.62576600	0.75995800	-0.92485100
H	2.72856400	-2.37684900	1.46407800
C	3.94469800	-1.99587700	-1.63326300
C	-1.89032800	0.38537500	-0.58857600
C	-2.99529200	1.15087900	-0.99512200
C	-2.08750000	-0.82592300	0.09522000
C	-4.28504400	0.72289400	-0.70383300
H	-2.82977100	2.06888800	-1.54706000
C	-3.37228200	-1.26230300	0.38890800
H	-1.22029800	-1.40971400	0.38297500
C	-4.45830600	-0.47704400	-0.00945200
H	-5.14914500	1.30070000	-1.00726200
H	-3.54201600	-2.19220100	0.91731500
N	-5.81178100	-0.92797700	0.30170300
O	-6.76066300	-0.23167800	-0.07210600
O	-5.94625700	-1.98485200	0.92611700
H	4.05564200	-3.29804400	0.72673200
H	3.98773400	-2.19601100	-2.69965300

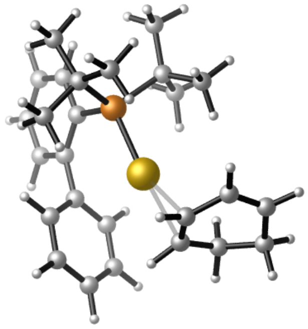

Ch-Au

E (opt) = -1488.698727 Hartrees
G (opt) = -1488.215512 Hartrees
E (SP) = -1488.961729 Hartrees

C	-3.19451500	0.66965100	0.21181600
H	-3.01853400	1.60278400	0.74140900
C	-3.27929700	-0.51445000	0.91357200
C	-3.77439200	-1.73712000	0.26511600
H	-3.62346000	-2.68701100	0.76868600
C	-4.46391600	-1.64722800	-0.88562500
H	-4.90252800	-2.53762500	-1.32988200
H	-3.13356100	-0.52205900	1.99232300
Au	-0.96629200	-0.15485300	0.32069600
P	1.24521000	-0.89503100	0.05482700
C	1.79040000	-1.67905800	1.70069400
C	1.21610000	-2.10562700	-1.43220600
C	2.47360500	0.42257000	-0.34262400
C	0.87544800	-2.88315600	1.99100700
C	3.26417300	-2.12212800	1.74099800
C	1.57867600	-0.59695100	2.78051100
C	-0.11327700	-2.89483200	-1.40872800
C	1.23456800	-1.23233800	-2.70279000
C	2.37553800	-3.11645500	-1.46193000
C	2.16180600	1.80310600	-0.38772400
C	3.77998100	0.01684900	-0.68279300
H	-0.18456400	-2.61033400	1.95080500
H	1.05119300	-3.70710800	1.29438200
H	1.08890100	-3.25139500	3.00100300
H	3.94725200	-1.27490700	1.64531800
H	3.45384500	-2.58475300	2.71672400
H	3.50926100	-2.85931200	0.97505400

H	0.53505100	-0.27261700	2.84082600
H	1.86150700	-1.01328900	3.75426100
H	2.20538500	0.28153100	2.59928700
H	-0.09935100	-3.61545700	-2.23471600
H	-0.26129600	-3.45489600	-0.48283300
H	-0.97795400	-2.24044700	-1.55373400
H	0.45026400	-0.46667100	-2.67737800
H	2.19601400	-0.73539300	-2.85231100
H	1.04713300	-1.87386100	-3.57136200
H	2.34818300	-3.79156500	-0.60329500
H	2.27261800	-3.72827100	-2.36577600
H	3.35775300	-2.64339200	-1.50744800
C	3.15898200	2.70849100	-0.79263200
C	0.84684200	2.41864200	-0.02521000
C	4.75627500	0.93022100	-1.07006600
H	4.04295100	-1.03136200	-0.65541200
C	4.44161800	2.28722500	-1.13215500
H	2.91155200	3.76512800	-0.82928100
C	0.51487000	2.66204600	1.31749700
C	-0.01244600	2.88633700	-1.03306300
H	5.75144500	0.57946000	-1.32593100
H	5.18807900	3.01376300	-1.43916700
C	-0.65317500	3.35557900	1.64363000
H	1.18041500	2.31883400	2.10244000
C	-1.18093600	3.57794500	-0.70480600
H	0.24569800	2.71582000	-2.07421100
C	-1.50149800	3.81904100	0.63382200
H	-0.89240100	3.54534800	2.68617100
H	-1.83312500	3.93777400	-1.49537500
H	-2.40314900	4.36886900	0.88799500
C	-3.62672800	0.71546900	-1.24735700
C	-4.73217700	-0.31462900	-1.54246700
H	-2.76263900	0.51573000	-1.89473800
H	-3.96626900	1.72521900	-1.49375700
H	-4.83981600	-0.43720900	-2.62476400
H	-5.70608300	0.05231500	-1.18162500

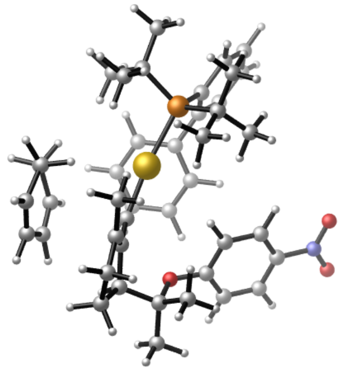

XIII

E (opt) = -2390.20660479 Hartrees
G (opt) = -2389.421790 Hartrees
E (SP) = -2390.703351 Hartrees

Au	0.19124500	-0.94403400	-0.02301300
P	2.49252100	-0.97583100	-0.71455100
C	-1.78443600	-0.74109100	0.36407900
H	-2.01370900	-0.25780600	1.31184100
C	-2.03045200	-2.87388300	2.44387000
H	-1.67825100	-2.18306700	3.20318100
C	-2.91983700	-0.88780800	-0.44822200
C	-3.03704500	-1.64585400	-1.60423200
C	3.13033400	-2.76709200	-0.61343200
C	2.50350000	-0.24844300	-2.49284200
C	-2.93182000	-4.53454100	0.31448000
H	-3.28997400	-5.19825100	-0.46891800
C	-3.79671400	-3.76743600	1.00913400
H	-4.86362300	-3.81081100	0.80730500
C	-4.44724900	-1.62393000	-2.11433000
H	-4.45827900	-1.04828300	-3.05209700
H	-4.79119800	-2.63092600	-2.37627100
C	-4.24492400	-0.19753400	-0.12671300
H	-4.47401300	-0.29327600	0.93951000
C	-1.99920000	-2.38926600	-2.35830000
H	-1.00137600	-2.28214600	-1.93599400
H	-2.26185500	-3.45236300	-2.40732600
H	-1.99145900	-2.02774500	-3.39513200
C	-5.27593100	-0.95541000	-0.99714800
H	-5.75624400	-1.72692300	-0.38975400
H	-6.06675000	-0.31430500	-1.39255400
C	-4.15678000	1.33320900	-0.41101800
C	-3.90837900	1.65871400	-1.88284900
H	-4.76314900	1.33127400	-2.47935600
H	-3.79789500	2.73649200	-2.02872300
H	-3.00915900	1.16710500	-2.26139800
C	-5.39262500	2.06384300	0.11353500
H	-5.51765000	1.87769200	1.18426700
H	-5.29324100	3.14183100	-0.04539500
H	-6.29502200	1.72964600	-0.40665100
O	-3.01263100	1.74589800	0.42424600
C	-3.31132900	-2.83050500	2.01768800
C	1.22188400	-0.70824200	-3.22454700
H	0.31996400	-0.31832300	-2.74581300
H	1.25049200	-0.32094700	-4.25005200
H	1.13367200	-1.79514500	-3.28291600
C	2.43369000	1.28395800	-2.32609300
H	1.61207800	1.57792600	-1.66313300
H	3.36123500	1.70019600	-1.92598100
H	2.25291400	1.74006200	-3.30655600
C	3.70984600	-0.63441300	-3.36525200
H	3.73868700	-1.70817600	-3.56685100
H	3.61570200	-0.12188600	-4.33010500
H	4.66626300	-0.33227800	-2.93658400
C	2.26690400	-3.63952900	-1.54347300
H	2.52020700	-4.69315300	-1.37655600
H	1.19713400	-3.52001300	-1.34211100
H	2.45032900	-3.41968300	-2.59851400
C	4.61853300	-2.96294100	-0.95230400
H	4.84670700	-4.03393100	-0.88966100
H	4.87538800	-2.63152800	-1.95955200
H	5.26857000	-2.45088800	-0.23893400
C	2.90876800	-3.20402500	0.84992900
H	3.51202700	-2.60904500	1.54218400
H	1.86016300	-3.12340500	1.15074500
H	3.21243200	-4.25202400	0.95837400
C	3.67670300	0.05520800	0.26664700
C	3.30896700	0.75224700	1.44420900
C	4.98995100	0.21749100	-0.22001400
C	4.25131300	1.59682200	2.05798000
C	5.91366000	1.04777700	0.40926000
H	5.30446500	-0.30534500	-1.11226900
C	5.53930200	1.75145200	1.55330500
H	3.95641800	2.13093900	2.95645100
H	6.91495900	1.14588500	0.00075000
H	6.24342900	2.41086900	2.05222600
C	1.98320700	0.66068400	2.12983600
C	1.68248400	-0.42863400	2.96217100
C	1.06624200	1.71856600	2.03729500
C	0.48281600	-0.46360300	3.67556000
H	2.39376300	-1.24257000	3.05403600
C	-0.13732500	1.67861700	2.74518700
H	1.29438600	2.56938500	1.40274800
C	-0.43222000	0.58770700	3.56626100
H	0.26813900	-1.30869000	4.32323100
H	-0.84034900	2.50066100	2.65442200
H	-1.36467200	0.56007600	4.12284000
C	-2.26431300	2.84648200	0.12680500
C	-2.58146600	4.08400300	0.70619400
C	-1.11390900	2.71128800	-0.66649800
C	-1.75444200	5.18210400	0.49663600
H	-3.46453400	4.16630500	1.32951800
C	-0.28317300	3.80225400	-0.88170200
H	-0.87112600	1.74096700	-1.08273500
C	-0.61446800	5.02688800	-0.29548700
H	-1.97748400	6.14420000	0.94073800
H	0.61358600	3.71415000	-1.48215000

N	0.26387200	6.17506200	-0.50986500
O	-0.04585200	7.25118800	0.00872800
O	1.27495400	6.01406800	-1.19974700
H	-4.00160000	-2.09290300	2.41762200
C	-1.10315700	-3.96435400	1.97326800
C	-1.44646700	-4.44259500	0.55288400
H	-1.18614600	-4.80506400	2.68105900
H	-0.06214600	-3.63091300	2.01491200
H	-0.96078600	-5.40215200	0.34638700
H	-1.02914500	-3.72937800	-0.17530200

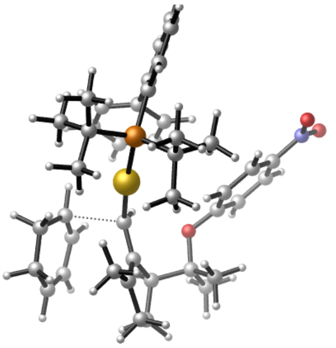

TS$_{XIII-XIVb}$

E (opt) = -2390.20335557 Hartrees
G (opt) = -2389.414465 Hartrees
E (SP) = -2390.698032 Hartrees

Au	-0.01936600	-1.02763400	0.00952900
P	2.19989400	-1.51871900	-0.70952000
C	-1.95885500	-0.48462800	0.47350400
H	-1.98380200	0.27093200	1.25213400
C	-2.34231200	-2.15169400	1.96012600
H	-1.41563500	-1.92382000	2.47273400
C	-3.04860300	-0.34324200	-0.45151700
C	-3.30754600	-1.09001400	-1.57115500
C	2.44873500	-3.40260700	-0.57233300
C	2.36028100	-0.85411400	-2.50428200
C	-4.90481000	-2.88634400	0.96206200
H	-5.88092200	-3.11935200	0.54186300
C	-4.77075900	-1.82855500	1.79364800
H	-5.62887300	-1.21296800	2.04546900
C	-4.58323400	-0.65105800	-2.23975800
H	-4.34407700	-0.14266800	-3.18475600
H	-5.20727300	-1.51122000	-2.50955300
C	-4.10545500	0.75227200	-0.28540600
H	-4.43006000	0.80389900	0.76066200
C	-2.49519700	-2.17265500	-2.19075800
H	-1.66112500	-2.49353000	-1.56628600
H	-3.12192500	-3.03838600	-2.43559700
H	-2.09182900	-1.81283700	-3.14727700
C	-5.25383700	0.28575100	-1.21315500
H	-5.97361000	-0.28538500	-0.62021300
H	-5.79763800	1.10820300	-1.68288400
C	-3.55909300	2.16967100	-0.63538500
C	-3.10990000	2.29530700	-2.09071100
H	-3.97191400	2.18225500	-2.75201700
H	-2.67920700	3.28157900	-2.28169800
H	-2.36839800	1.53587400	-2.34972900
C	-4.57477200	3.25739500	-0.28185700
H	-4.84726200	3.19434800	0.77587000
H	-4.15202300	4.24805100	-0.47460900
H	-5.48352900	3.15965300	-0.88203800
O	-2.41602000	2.32352700	0.28626300
C	-3.47608400	-1.45641600	2.30746500
C	0.99582200	-1.02809600	-3.21081200
H	0.20986000	-0.44141200	-2.72728400
H	1.09130600	-0.67239300	-4.24364500
H	0.66954200	-2.06945900	-3.24704500
C	2.64298000	0.65567600	-2.36930400
H	1.94578700	1.12666800	-1.66927300
H	3.65990700	0.85853100	-2.02511400
H	2.51423900	1.13447700	-3.34702400
C	3.43386300	-1.52173300	-3.38072100
H	3.21838400	-2.57780600	-3.55983800
H	3.44129200	-1.01907400	-4.35546200
H	4.44170000	-1.43493800	-2.97216300
C	1.43705000	-4.10012900	-1.50144700
H	1.46832700	-5.17957500	-1.31164600
H	0.41203000	-3.75885400	-1.32174600
H	1.67348400	-3.94442800	-2.55694400
C	3.87175200	-3.89340000	-0.88937500
H	3.88677400	-4.98710600	-0.80982600
H	4.19614800	-3.63275800	-1.89819500
H	4.60357600	-3.50492100	-0.17701600
C	2.12543700	-3.76638300	0.89244700
H	2.81574900	-3.28380000	1.59082300
H	1.10322600	-3.48885400	1.16969200
H	2.22906100	-4.85060500	1.01844100
C	3.57899900	-0.75504200	0.26294200
C	3.38216700	-0.03777700	1.46886400
C	4.89041800	-0.85449500	-0.24536900
C	4.49248400	0.55566500	2.09628400
C	5.97802600	-0.26405700	0.39228200
H	5.07449600	-1.39738400	-1.16186700
C	5.77758500	0.45159200	1.57196800
H	4.32941800	1.10556800	3.01845300
H	6.97147900	-0.36194800	-0.03504000
H	6.61251000	0.92354600	2.08156800
C	2.07334100	0.15567100	2.16918000
C	1.58271200	-0.81582200	3.05486800
C	1.37975300	1.36867300	2.04109400
C	0.41306800	-0.58435500	3.78254600
H	2.12268700	-1.74875800	3.18003600
C	0.20444900	1.59514900	2.76091700
H	1.75685300	2.13189000	1.36835500
C	-0.28271300	0.61906600	3.63318800
H	0.04987900	-1.34137100	4.47214300
H	-0.32541400	2.53458500	2.63874900
H	-1.19155600	0.79833900	4.20075500
C	-1.42178300	3.22196500	0.04023200
C	-1.46850400	4.49265500	0.63350900
C	-0.30371400	2.83714100	-0.71686900
C	-0.41019100	5.37896100	0.46711000
H	-2.33131600	4.76525000	1.23055500
C	0.75574300	3.71683100	-0.89185600
H	-0.27422800	1.83992800	-1.14037800
C	0.68907900	4.98004400	-0.29703500
H	-0.42595400	6.36185900	0.92122900
H	1.62815000	3.43459000	-1.46788500
N	1.80905800	5.90252600	-0.46824400
O	1.72749200	7.02180200	0.04535800
O	2.78573000	5.51851200	-1.11878000
H	-3.39663800	-0.58792300	2.95434800
C	-2.43327400	-3.50700600	1.30179900
C	-3.77519900	-3.80088800	0.58484200
H	-2.28484000	-4.23485500	2.11201800
H	-1.59148900	-3.64699800	0.61797000
H	-4.08724300	-4.83338200	0.78707500
H	-3.65504100	-3.75961900	-0.50249500

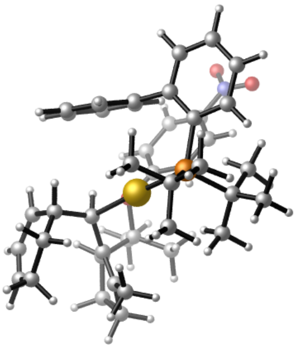

XIVb

E (opt) = -2390.21567887 Hartrees
G (opt) = -2389.427602 Hartrees
E (SP) = -2390.707092 Hartrees

Au	-0.33159700	-0.95277500	0.05624000
P	1.66449600	-1.95946100	-0.71352400
C	-2.26313600	-0.14394100	0.73227000
H	-1.84016700	0.75890300	1.17463000
C	-2.72117100	-1.13258700	1.90557400
H	-1.85344900	-1.28633400	2.54155300
C	-3.13985700	0.20003800	-0.41217600
C	-3.57304800	-0.58630500	-1.43246100
C	1.45664400	-3.84490400	-0.49803500
C	1.92445700	-1.43144700	-2.54214500
C	-5.52472700	-1.06717900	1.32866600
H	-6.55471800	-0.97980600	0.98889800
C	-4.96531400	-0.03521600	2.02394700
H	-5.54441800	0.85633200	2.24418100
C	-4.53017100	0.15067000	-2.33898000
H	-4.05182600	0.36351400	-3.30557800
H	-5.41572500	-0.45626900	-2.56583200
C	-3.68865300	1.62527700	-0.56928900
H	-4.03808200	1.99296000	0.40333300
C	-3.16041700	-1.97242300	-1.80485500
H	-2.48655500	-2.42570400	-1.07847900
H	-4.03057800	-2.62635100	-1.94446900
H	-2.64278700	-1.94651800	-2.77345300
C	-4.86953500	1.43266400	-1.55374500
H	-5.78159800	1.26997500	-0.97121800
H	-5.04801400	2.29406300	-2.20143600
C	-2.64899500	2.67449900	-1.05917000
C	-2.05807000	2.33733300	-2.42737300
H	-2.84368000	2.37221000	-3.18558400
H	-1.29605800	3.06506600	-2.71688700
H	-1.61367700	1.33947200	-2.43232700
C	-3.24527100	4.08443500	-1.05490500
H	-3.65479000	4.32489400	-0.06912800
H	-2.47691600	4.82327900	-1.30246200
H	-4.04364900	4.17663700	-1.79555500
O	-1.61066200	2.63226000	-0.00834800
C	-3.60286500	-0.09369800	2.41090000
C	0.52896300	-1.20730700	-3.16865500
H	-0.00922500	-0.39184200	-2.67659500
H	0.65830400	-0.93776200	-4.22343400
H	-0.09746300	-2.10120700	-3.12523800
C	2.66983600	-0.08130800	-2.51322800
H	2.23869700	0.60130500	-1.77684600
H	3.73015000	-0.19927200	-2.27913500
H	2.59013100	0.39177700	-3.49871300
C	2.69114900	-2.43036000	-3.42692900
H	2.15631800	-3.37603400	-3.53852600
H	2.79460700	-1.99144500	-4.42656600
H	3.69914300	-2.63976200	-3.06494400
C	0.26644200	-4.30403500	-1.36063900
H	0.05716900	-5.35794100	-1.14253700
H	-0.63860500	-3.73007200	-1.14042400
H	0.47421000	-4.22374900	-2.43056700
C	2.70804400	-4.67185500	-0.84298100
H	2.47301700	-5.73070500	-0.68149800
H	3.02110500	-4.56155600	-1.88162700
H	3.55149700	-4.42310400	-0.19474700
C	1.12433700	-4.07856800	0.99076100
H	1.95143700	-3.77640100	1.63956700
H	0.22341600	-3.54176300	1.30411400
H	0.95272400	-5.14994500	1.14907300
C	3.21395300	-1.52415400	0.20021500
C	3.24305600	-0.79257000	1.41282000
C	4.43916300	-1.93750600	-0.36189700
C	4.48895200	-0.50404700	1.99890700
C	5.66172500	-1.64614400	0.23614800
H	4.44463700	-2.49255800	-1.29040200
C	5.68744700	-0.92211500	1.42762100
H	4.50320700	0.05970900	2.92684300
H	6.58333100	-1.98160800	-0.22981500
H	6.63101900	-0.68298600	1.90933800
C	2.05032800	-0.27844400	2.15785400
C	1.36333800	-1.09549800	3.06897600
C	1.68410100	1.07128100	2.04822000
C	0.31780900	-0.57786600	3.83637000
H	1.65702200	-2.13334500	3.18366200
C	0.63018500	1.58562900	2.80721300
H	2.22042500	1.71612600	1.36080600
C	-0.05641400	0.76268500	3.70286800
H	-0.19850500	-1.22011300	4.54452700
H	0.35308100	2.62953400	2.69887800
H	-0.86734600	1.16518200	4.30322000
C	-0.43286600	3.30041300	-0.15322200
C	-0.25177100	4.52665200	0.50470600
C	0.63069700	2.71876700	-0.86074300
C	0.97950100	5.17033700	0.45602300
H	-1.08070100	4.95490100	1.05689600
C	1.86106600	3.35802700	-0.92395200
H	0.48084300	1.75741000	-1.33538900
C	2.02190700	4.57814500	-0.26158100
H	1.13875700	6.11411500	0.96254300
H	2.69282300	2.91854500	-1.46078000
N	3.32074800	5.24472900	-0.31069700
O	3.45168600	6.31320400	0.29318900
O	4.22776200	4.70713800	-0.95314800
H	-3.18376400	0.69433300	3.02886000
C	-3.39875200	-2.47065900	1.60248500
C	-4.84462100	-2.37097600	1.06584700
H	-3.41944600	-3.01274100	2.55402700
H	-2.77327400	-3.05785400	0.92553300
H	-5.46541900	-3.15708300	1.51947600
H	-4.89812600	-2.57557300	-0.00712700

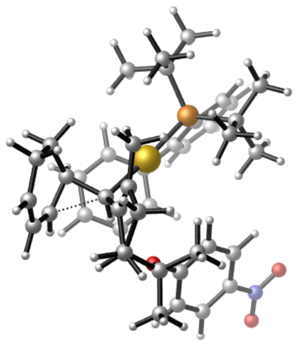

TS$_{XIVb-XVb}$

E (opt) = -2390.21564270 Hartrees
G (opt) = -2389.426048 Hartrees
E (SP) = -2390.706967 Hartrees

Au	-0.33018200	-0.94967200	0.04250700
P	1.65985800	-1.96029000	-0.71264500
C	-2.28334200	-0.14557900	0.72380500

H	-1.81323800	0.73809400	1.15794100
C	-2.71452400	-1.16935300	1.86914600
H	-1.83883800	-1.37060600	2.47958700
C	-3.14540500	0.21265100	-0.42765800
C	-3.56960700	-0.56448400	-1.45597900
C	1.45199200	-3.84505300	-0.49348900
C	1.93030900	-1.43588700	-2.54097500
C	-5.54513200	-0.98316200	1.40262900
H	-6.59234300	-0.86266200	1.13294700
C	-4.90895800	0.02623100	2.05460100
H	-5.44007500	0.93943900	2.30531300
C	-4.51982300	0.18077900	-2.36358100
H	-4.03798300	0.39626500	-3.32773900
H	-5.40729200	-0.42171800	-2.59534800
C	-3.68177600	1.64253600	-0.58040500
H	-4.03432900	2.01018400	0.39064200
C	-3.16753600	-1.95496700	-1.82393600
H	-2.45835900	-2.39082900	-1.12026800
H	-4.03911800	-2.61675700	-1.90815700
H	-2.69665000	-1.94600900	-2.81628700
C	-4.85822700	1.46000000	-1.57217300
H	-5.77290500	1.29610700	-0.99402500
H	-5.03103500	2.32628700	-2.21508900
C	-2.63123800	2.68452900	-1.06142900
C	-2.04614800	2.35583700	-2.43413700
H	-2.83184500	2.40986700	-3.19107600
H	-1.27491400	3.07687600	-2.71586300
H	-1.61507400	1.35221800	-2.45156400
C	-3.21195500	4.10062000	-1.04075100
H	-3.61137700	4.33696400	-0.04986100
H	-2.43853000	4.83402300	-1.28869700
H	-4.01562200	4.20595600	-1.77406900
O	-1.59015100	2.61815500	-0.01338500
C	-3.52554600	-0.08232500	2.38305100
C	0.53799200	-1.20993300	-3.17439800
H	0.00042300	-0.38981900	-2.68915600
H	0.67317200	-0.94630200	-4.22988400
H	-0.09196400	-2.10136600	-3.12944300
C	2.68019700	-0.08839800	-2.51243200
H	2.25025500	0.59738300	-1.77865400
H	3.73942300	-0.20974900	-2.27541500
H	2.60466300	0.38273900	-3.49911700
C	2.69859900	-2.43942800	-3.41937500
H	2.16037100	-3.38281300	-3.53385700
H	2.81054200	-2.00164200	-4.41852800
H	3.70316800	-2.65316900	-3.05013000
C	0.26630600	-4.30789600	-1.36035900
H	0.06047700	-5.36264700	-1.14329100
H	-0.64201000	-3.73790800	-1.14304300
H	0.47704800	-4.22642800	-2.42956300
C	2.70741000	-4.66862600	-0.83232900
H	2.47568400	-5.72752600	-0.66676900
H	3.02226900	-4.56160400	-1.87064100
H	3.54843200	-4.41404300	-0.18316200
C	1.11506400	-4.07811200	0.99407700
H	1.94007300	-3.77580400	1.64534100
H	0.21321800	-3.54169500	1.30459700
H	0.94304500	-5.14945500	1.15180800
C	3.19670200	-1.51651500	0.21751000
C	3.21142200	-0.78802600	1.43268600
C	4.42851500	-1.92202600	-0.33568100
C	4.45136100	-0.49374800	2.02851500
C	5.64463700	-1.62535000	0.27269700
H	4.44446200	-2.47527800	-1.26515100
C	5.65672300	-0.90353200	1.46564400
H	4.45530100	0.06757000	2.95801400
H	6.57171500	-1.95497700	-0.18649200
H	6.59506700	-0.66016800	1.95536300
C	2.01100000	-0.28433700	2.17328300
C	1.32061500	-1.11146400	3.07296900
C	1.64148200	1.06549100	2.07443800
C	0.26738600	-0.60458000	3.83693600
H	1.61727100	-2.14903600	3.18100000
C	0.57960000	1.56907500	2.82980400
H	2.18103900	1.71848000	1.39738300
C	-0.11170800	0.73542200	3.71191300
H	-0.25173600	-1.25509400	4.53537800
H	0.30017600	2.61310000	2.72902700
H	-0.92961300	1.12895500	4.30873000
C	-0.40703300	3.27820000	-0.15437400
C	-0.21476300	4.49815300	0.51176400
C	0.65027400	2.69340400	-0.86852500
C	1.02148300	5.13247800	0.46453900
H	-1.03883300	4.92906600	1.06908200
C	1.88571600	3.32305000	-0.93015800
H	0.49127800	1.73709700	-1.35037700
C	2.05742500	4.53737300	-0.25992800
H	1.18941300	6.07137500	0.97729400
H	2.71292800	2.88123600	-1.47203200
N	3.36129700	5.19435700	-0.30820000
O	3.50186900	6.25806300	0.30181800
O	4.26248600	4.65369600	-0.95619900
H	-3.05854100	0.67004400	3.01012700
C	-3.46058200	-2.46771600	1.56003000
C	-4.91689100	-2.29997600	1.07017800
H	-3.47620100	-3.02293700	2.50427800
H	-2.88383300	-3.07251600	0.85635400
H	-5.54684600	-3.08865300	1.50475800
H	-5.00010700	-2.45358300	-0.00991100

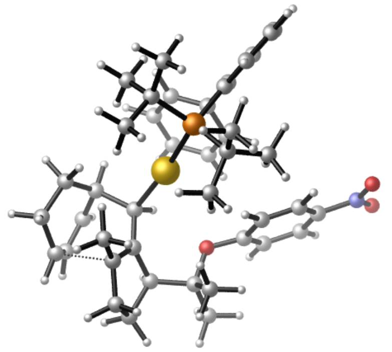

TS$_{XIVb-XVIb}$

E (opt) = -2390.21266218 Hartrees
G (opt) = -2389.417793Hartrees
E (SP) = -2390.703149 Hartrees

Au	-0.45857600	-0.83802800	0.07537100
P	1.40765700	-2.08777900	-0.72685600
C	-2.25800600	0.09624800	0.84880400
H	-1.85012600	0.94336100	1.40059600
C	-2.94224200	-0.85728900	1.92283500
H	-2.18917100	-1.00878100	2.69487400
C	-3.06350800	0.58430000	-0.25709400
C	-3.78216200	-0.13229900	-1.21745500
C	0.91998000	-3.93066000	-0.59228400
C	1.78915900	-1.54304600	-2.53039800
C	-5.31868900	-0.73289900	0.53455900
H	-6.14481500	-0.57493300	-0.15299600
C	-5.16753200	0.14900000	1.63575200
H	-5.87267600	0.96208900	1.78391500
C	-4.62883200	0.80181900	-2.05752700
H	-4.13868500	0.95310900	-3.02915200
H	-5.61577500	0.37922300	-2.27403600
C	-3.41381400	2.07174200	-0.35282300
H	-3.62487300	2.43694700	0.66026200
C	-3.41265000	-1.44678300	-1.83712100
H	-2.80771900	-2.08234400	-1.19487700
H	-4.29464500	-1.99903100	-2.17518400
H	-2.81911700	-1.22616500	-2.73507500
C	-4.68853800	2.09742800	-1.23468700
H	-5.56507500	2.07843800	-0.58062300
H	-4.76867600	2.98895800	-1.85969200
C	-2.26551500	2.97241200	-0.89315000
C	-1.82494900	2.58599300	-2.30504900
H	-2.64838800	2.74485600	-3.00517100

H	-0.99050300	3.20876200	-2.63685900
H	-1.52046700	1.53750600	-2.35254200
C	-2.65701400	4.45036100	-0.82418100
H	-2.96281400	4.71889600	0.19125300
H	-1.80897400	5.07916400	-1.11062100
H	-3.47952600	4.67422600	-1.50754100
O	-1.18503600	2.74461900	0.07927300
C	-4.03953700	0.02708500	2.40467600
C	0.46017200	-1.06301500	-3.15436400
H	0.07641600	-0.17681100	-2.64130600
H	0.63695700	-0.79523100	-4.20266900
H	-0.31505400	-1.83304000	-3.13003900
C	2.75366000	-0.34231700	-2.44621400
H	2.44694200	0.37110300	-1.67839200
H	3.77719300	-0.65133100	-2.22295800
H	2.75996300	0.17879000	-3.41044300
C	2.39030700	-2.62305400	-3.44703300
H	1.69805600	-3.45061800	-3.61757800
H	2.60080200	-2.16602200	-4.42163300
H	3.33222900	-3.02817200	-3.07317100
C	-0.31687800	-4.17436300	-1.47736400
H	-0.67976600	-5.19466900	-1.30570900
H	-1.13114800	-3.48448200	-1.23709100
H	-0.08993000	-4.07992800	-2.54228200
C	2.03090500	-4.92945900	-0.96202000
H	1.63082800	-5.94379100	-0.84460900
H	2.37292900	-4.83088200	-1.99274700
H	2.89361700	-4.84255100	-0.29735400
C	0.54142700	-4.16479200	0.88515100
H	1.39724200	-4.00627600	1.54781800
H	-0.27415900	-3.51217500	1.21030600
H	0.21389800	-5.20419700	1.00619900
C	2.99028000	-1.92458300	0.21942000
C	3.10930000	-1.20903800	1.43635400
C	4.15133900	-2.50950600	-0.32657800
C	4.37629900	-1.09769500	2.03739800
C	5.39556900	-2.39455700	0.28719500
H	4.09221400	-3.05909500	-1.25629300
C	5.51090700	-1.67825700	1.47788900
H	4.45806700	-0.54263200	2.96718000
H	6.26573600	-2.85785600	-0.16805100
H	6.47375600	-1.57218500	1.96906100
C	1.98997100	-0.54143700	2.17311700
C	1.17638700	-1.26903100	3.05547700
C	1.82081300	0.84830000	2.08410500
C	0.20175000	-0.62101500	3.81720200
H	1.31615100	-2.34038000	3.15214300
C	0.83828100	1.49382900	2.83825000
H	2.45590100	1.42215000	1.41709700
C	0.02514300	0.76125500	3.70570400
H	-0.41465800	-1.19574400	4.50288500
H	0.71313000	2.56791900	2.74605400
H	-0.73503200	1.26351400	4.29739100
C	0.04975200	3.29233300	-0.10228300
C	0.39183600	4.46818000	0.58290300
C	1.00815600	2.62609100	-0.87971900
C	1.68148300	4.97879700	0.49028200
H	-0.35817200	4.96155400	1.19063900
C	2.29665200	3.13237500	-0.98590300
H	0.72948400	1.70397700	-1.37291200
C	2.61828900	4.30498900	-0.29752100
H	1.96595100	5.88149500	1.01647300
H	3.05011700	2.62763300	-1.57733800
N	3.97691700	4.83363000	-0.39631000
O	4.24828700	5.86520800	0.22452900
O	4.79015600	4.22305700	-1.09633500
H	-3.84952300	0.70802300	3.23018000
C	-3.45950500	-2.22391800	1.44300900
C	-4.82457300	-2.14109100	0.71198500
H	-3.56063300	-2.87274900	2.31793700
H	-2.70196700	-2.67785000	0.79803400
H	-5.59691700	-2.62198700	1.32983000
H	-4.82488600	-2.69195000	-0.22803500

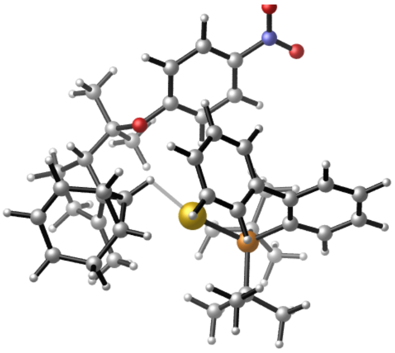

XVb

E (opt) = -2390.22501755 Hartrees
G (opt) = -2389.431700 Hartrees
E (SP) = -2390.715140 Hartrees

Au	-0.09764000	-0.93213700	0.08409700
P	1.88747100	-1.86849800	-0.63947000
C	-2.49654100	-0.31360600	0.84026200
H	-1.59358100	0.33156100	0.87303200
C	-2.60926100	-1.40180400	1.92296700
H	-1.67195600	-1.61589000	2.42926700
C	-3.27322800	-0.20503400	-0.43074000
C	-3.48818200	-1.15692100	-1.35906000
C	1.81735600	-3.73822100	-0.27456700
C	2.05051400	-1.44614300	-2.50396500
C	-5.49395000	-0.95689900	1.94390100
H	-6.57084400	-0.80775500	1.99186400
C	-4.67334600	0.05157600	2.26964100
H	-5.08073300	1.00985200	2.58375100
C	-4.43145900	-0.67528100	-2.43927400
H	-3.89534700	-0.53232000	-3.38765900
H	-5.21705100	-1.41456100	-2.64026000
C	-3.95240300	1.11028900	-0.81713300
H	-4.44201400	1.55232400	0.05663400
C	-2.92595200	-2.54021200	-1.43251100
H	-2.21066100	-2.74003300	-0.63369000
H	-3.72247800	-3.29419600	-1.38732300
H	-2.41395800	-2.68620400	-2.39260300
C	-4.99395800	0.64516900	-1.86798200
H	-5.93592500	0.44161200	-1.34937500
H	-5.20310100	1.39063300	-2.63945200
C	-2.97826000	2.19788200	-1.35395200
C	-2.29821300	1.80367800	-2.66434900
H	-3.04493300	1.71351000	-3.45630000
H	-1.58257400	2.56638800	-2.98080400
H	-1.77714500	0.84821800	-2.56598500
C	-3.68462800	3.54754700	-1.48903000
H	-4.14109300	3.83849300	-0.53829900
H	-2.97627700	4.32587000	-1.78951100
H	-4.46881900	3.49906200	-2.24967200
O	-1.98640300	2.31230400	-0.26254000
C	-3.19491300	-0.06028400	2.21863700
C	0.63508400	-1.46457600	-3.12498500
H	-0.00721300	-0.68592100	-2.70287700
H	0.72717800	-1.27419400	-4.20025900
H	0.13154600	-2.42525800	-2.99738900
C	2.60841600	-0.01217600	-2.58376100
H	2.05858800	0.66947700	-1.92963000
H	3.66603200	0.03546600	-2.31470400
H	2.50306600	0.34987200	-3.61246900
C	2.94256300	-2.40392700	-3.31486100
H	2.54085500	-3.41895800	-3.34430900
H	2.97875600	-2.03518200	-4.34644600
H	3.97156700	-2.44277800	-2.95445000
C	0.70022800	-4.37016400	-1.12366300
H	0.59164500	-5.42015100	-0.82906400
H	-0.26275300	-3.87766600	-0.96226300
H	0.92821000	-4.34883600	-2.19177700

C	3.15290600	-4.45905900	-0.53660300
H	3.00571000	-5.52542200	-0.33042500
H	3.49368800	-4.36781500	-1.56850400
H	3.94348700	-4.10384000	0.12850400
C	1.47198100	-3.89381100	1.21960800
H	2.24390200	-3.45574800	1.85825000
H	0.50820000	-3.44020500	1.47033800
H	1.41468300	-4.96309700	1.45331900
C	3.37439400	-1.19864700	0.22707700
C	3.34305600	-0.31832000	1.33878100
C	4.62743100	-1.54914300	-0.31672000
C	4.56079100	0.19012100	1.82704700
C	5.82015200	-1.04452600	0.19247900
H	4.68124300	-2.22376000	-1.15955000
C	5.78654100	-0.15950800	1.26886400
H	4.52964800	0.86886400	2.67394000
H	6.76398600	-1.33861100	-0.25615500
H	6.70520400	0.25254500	1.67555100
C	2.12656600	0.13786200	2.07978100
C	1.46388800	-0.70797100	2.98433100
C	1.71509300	1.47590800	1.97777400
C	0.40010700	-0.22950700	3.75269900
H	1.79248400	-1.73468300	3.09631200
C	0.64575600	1.95142600	2.74026300
H	2.23366500	2.14304800	1.29753000
C	-0.01384500	1.10045800	3.63027600
H	-0.09707100	-0.89239400	4.45496700
H	0.33611400	2.98687900	2.64123400
H	-0.83245700	1.47491700	4.23766400
C	-0.88671600	3.10758800	-0.37342000
C	-0.86815300	4.34406800	0.28990300
C	0.26280100	2.65024400	-1.03724800
C	0.28871500	5.11453000	0.29900600
H	-1.76134200	4.67733900	0.80631000
C	1.42167800	3.41427900	-1.03882900
H	0.24117200	1.68484500	-1.52600300
C	1.42203200	4.63843900	-0.36593800
H	0.32380100	6.06700800	0.81286800
H	2.32004300	3.06616700	-1.53332400
N	2.64785900	5.43258600	-0.34142600
O	2.63017300	6.51426200	0.25234700
O	3.64504900	4.98254800	-0.91391500
H	-2.64533800	0.60609800	2.87539100
C	-3.54850900	-2.59951100	1.89238200
C	-5.00431200	-2.30089800	1.47369000
H	-3.56092700	-2.97902900	2.92187500
H	-3.13752100	-3.40328500	1.27462300
H	-5.65838100	-3.09181500	1.86074800
H	-5.10039600	-2.34468000	0.38316600

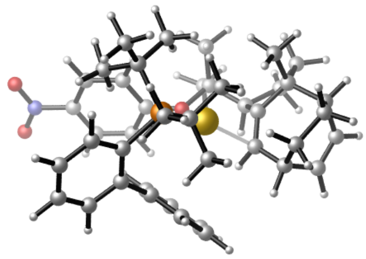

XVIb

E (opt) = -2390.25168581 Hartrees
G (opt) = -2389.453933 Hartrees
E (SP) = -2390.740709 Hartrees

Au	-0.81488400	-0.35577800	-0.05737100
P	0.34890000	-2.29178400	-0.78439200
C	-2.37535700	0.88737300	1.17575300
H	-1.67722700	1.22147500	1.94275900
C	-3.51738500	0.03685200	1.73652000
H	-3.13475700	-0.46193000	2.62865800
C	-2.44432200	1.66761400	0.03955500
C	-3.61864000	1.61441800	-0.96910700
C	-0.89232600	-3.74131200	-0.81531000
C	1.12182400	-1.91570200	-2.50538400
C	-4.98091500	1.12001900	-0.31166700
H	-5.76105100	1.48530300	-0.98684300
C	-5.22666300	1.65583500	1.08372100
H	-5.95734300	2.44465600	1.24332200
C	-3.76759200	3.10951000	-1.39807800
H	-3.35782200	3.24678700	-2.40249900
H	-4.81682900	3.41335200	-1.44231600
C	-1.77824400	3.05991200	0.05624700
H	-1.51659400	3.27908900	1.09484400
C	-3.31894400	0.75096000	-2.21027100
H	-3.10120900	-0.28704600	-1.95392100
H	-4.18144500	0.76320200	-2.88662500
H	-2.46189000	1.14418800	-2.75863400
C	-2.97903200	3.93870500	-0.37698300
H	-3.60044100	4.11928400	0.50606400
H	-2.67899500	4.91401100	-0.76550700
C	-0.45399000	3.32925100	-0.70697000
C	-0.48144500	3.03304400	-2.20178100
H	-1.24243200	3.64130600	-2.69613100
H	0.47997100	3.27619300	-2.66106800
H	-0.69685000	1.97980600	-2.38126300
C	0.00989500	4.76709000	-0.45187600
H	0.02977500	4.99428200	0.61816000
H	1.01319800	4.92133500	-0.85775000
H	-0.65478000	5.47847700	-0.94636000
O	0.47255600	2.40090000	-0.03276700
C	-4.56256600	1.08606600	2.09879100
C	0.21744500	-0.87441900	-3.20087900
H	0.22241700	0.07972700	-2.66844000
H	0.60257800	-0.69372300	-4.21119300
H	-0.81805300	-1.21186800	-3.29296000
C	2.50318000	-1.27969600	-2.25153700
H	2.47113100	-0.52863700	-1.45899300
H	3.25492600	-2.02313800	-1.97875400
H	2.83742400	-0.78530100	-3.17082000
C	1.27693600	-3.11978400	-3.45215800
H	0.31372800	-3.54148400	-3.74621800
H	1.77348800	-2.76720200	-4.36406200
H	1.89696800	-3.91707800	-3.03863000
C	-2.02213700	-3.39621400	-1.80270000
H	-2.80222500	-4.16277100	-1.73059400
H	-2.48202900	-2.43067900	-1.57982400
H	-1.67164800	-3.37765300	-2.83766700
C	-0.27718100	-5.10261400	-1.19049700
H	-1.07631600	-5.85266300	-1.15536800
H	0.14241500	-5.12339700	-2.19601900
H	0.49156900	-5.41586800	-0.48044000
C	-1.46051200	-3.86550900	0.61324300
H	-0.68953300	-4.18069200	1.32199400
H	-1.90279400	-2.93554500	0.97550100
H	-2.24399800	-4.63229300	0.61176100
C	1.72616100	-2.85053300	0.31369400
C	1.97997300	-2.32765000	1.60418200
C	2.59489400	-3.83947400	-0.19180500
C	3.10274400	-2.79363100	2.31321600
C	3.69401700	-4.29502600	0.52999500
H	2.42109800	-4.25953400	-1.17316200
C	3.95582400	-3.76124800	1.79122400
H	3.29297700	-2.38406700	3.30069900
H	4.34088200	-5.05593800	0.10414800
H	4.81264200	-4.09752300	2.36756600
C	1.14464600	-1.32602700	2.33900300
C	0.02899700	-1.74500500	3.08158100
C	1.56700600	0.00849400	2.44224100
C	-0.65177800	-0.84759400	3.90638800
H	-0.28602400	-2.78120400	3.03221000
C	0.88335300	0.90490900	3.26608400
H	2.43443400	0.34015300	1.88024800
C	-0.22468200	0.47985100	4.00322900

H	-1.50423000	-1.18969300	4.48618300
H	1.22412500	1.93241300	3.34011700
H	-0.74675700	1.17516400	4.65441700
C	1.82472100	2.55414200	-0.14159200
C	2.52474700	3.12037500	0.93495600
C	2.52272600	2.07627200	-1.25958000
C	3.91116000	3.19514200	0.90727400
H	1.96535400	3.49275300	1.78483400
C	3.91050700	2.14787600	-1.29832500
H	1.97619300	1.62903800	-2.07883200
C	4.58858500	2.70394900	-0.21207800
H	4.46639700	3.62371100	1.73222000
H	4.46578500	1.77080200	-2.14802700
N	6.04842100	2.76880400	-0.24478400
O	6.63183800	3.25544400	0.72772500
O	6.62890500	2.33290000	-1.24289900
H	-4.69404300	1.38540000	3.13512300
C	-4.12036700	-1.02473300	0.77975800
C	-5.12569200	-0.41766000	-0.21400100
H	-4.60566700	-1.79791300	1.38340900
H	-3.30363800	-1.51146700	0.24404400
H	-6.14733400	-0.61248700	0.12774000
H	-5.03599600	-0.88838800	-1.19636600

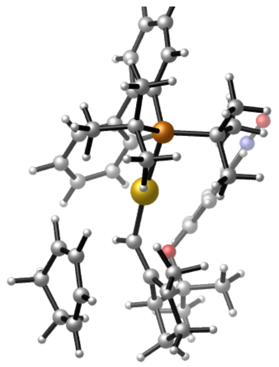

XIIIa

E (opt) = -2390.20515974 Hartrees
G (opt) = -2389.421267 Hartrees
E (SP) = -2390.701266 Hartrees

Au	-0.04600200	-0.95209300	-0.05805100
P	2.17025300	-1.62098200	-0.69065800
C	-1.93253600	-0.32047600	0.32551500
H	-2.07427500	0.15333100	1.29834900
C	-2.58579700	-2.49045700	2.08519400
H	-1.62978400	-2.29458400	2.55996100
C	-3.04024400	-0.14486800	-0.52878600
C	-3.34301500	-0.86574500	-1.66803300
C	2.27045500	-3.52093400	-0.58983500
C	2.44716200	-0.92749100	-2.46017400
C	-5.04594900	-2.86413700	0.70075000
H	-5.98591700	-3.03457600	0.18082700
C	-3.92060200	-3.48810300	0.29593100
H	-3.94057100	-4.17535900	-0.54516000
C	-4.63240200	-0.39552200	-2.27523600
H	-4.39956500	0.10270300	-3.22834900
H	-5.28831100	-1.23595300	-2.52733300
C	-4.06450900	0.96891200	-0.31111500
H	-4.37116400	1.01922100	0.73903800
C	-2.56653800	-1.95300000	-2.31630800
H	-1.76958900	-2.34214100	-1.68443100
H	-3.22621500	-2.77114000	-2.62152200
H	-2.11618900	-1.55483100	-3.23704600
C	-5.24111600	0.56785900	-1.23259300
H	-5.99141500	0.03578300	-0.64112900
H	-5.73995100	1.42220500	-1.69480500
C	-3.43736500	2.35920500	-0.63372500
C	-3.01889200	2.50847000	-2.09622000
H	-3.90177800	2.46386600	-2.73848300
H	-2.53962800	3.47640900	-2.26418100
H	-2.32337700	1.72282400	-2.40061700
C	-4.37071500	3.49442500	-0.21231200
H	-4.61431300	3.41438200	0.85114500
H	-3.89834500	4.46475900	-0.39168500
H	-5.30113300	3.46573000	-0.78667400
O	-2.25954200	2.38315200	0.25427500
C	-2.64459100	-3.20960400	0.94084400
C	1.11248800	-1.02045300	-3.23532600
H	0.32718200	-0.42023800	-2.76733300
H	1.27211000	-0.63182900	-4.24820600
H	0.74781200	-2.04546100	-3.32728200
C	2.79505700	0.56478100	-2.27975500
H	2.07368300	1.06519900	-1.62594000
H	3.79343700	0.71035600	-1.86039100
H	2.76219200	1.05935000	-3.25755700
C	3.53214500	-1.62504000	-3.29672600
H	3.27932300	-2.66656500	-3.51019300
H	3.61303800	-1.10440000	-4.25865700
H	4.51948000	-1.59227500	-2.83415100
C	1.24589600	-4.11563400	-1.57413400
H	1.17367800	-5.19571000	-1.40077700
H	0.24806700	-3.68650500	-1.43382400
H	1.54380100	-3.96865100	-2.61549400
C	3.66151600	-4.11843200	-0.86282600
H	3.58640100	-5.21073300	-0.79978900
H	4.04316100	-3.87209500	-1.85529700
H	4.39359600	-3.79832200	-0.11785200
C	1.85984500	-3.88577600	0.85264100
H	2.56219000	-3.47862100	1.58628500
H	0.85604500	-3.52594200	1.09918200
H	1.86467500	-4.97727900	0.95744400
C	3.56267300	-0.97467300	0.34220100
C	3.36894100	-0.22784300	1.53043300
C	4.88201600	-1.17787900	-0.11029500
C	4.49142700	0.30817400	2.18652500
C	5.98221100	-0.64890400	0.55914400
H	5.05971600	-1.75628200	-1.00604900
C	5.78524600	0.10865700	1.71316900
H	4.33178200	0.88351100	3.09366200
H	6.98280500	-0.82594400	0.17626500
H	6.63023600	0.53663100	2.24458100
C	2.05044400	0.03753200	2.18598100
C	1.45769600	-0.92806000	3.01457700
C	1.44553200	1.29858800	2.07303500
C	0.27522900	-0.64249800	3.70105200
H	1.92881600	-1.89898700	3.12643000
C	0.25928500	1.58049900	2.75480100
H	1.90138600	2.05526100	1.44227900
C	-0.32962500	0.61099600	3.56961900
H	-0.16684200	-1.39599400	4.34679700
H	-0.20015200	2.55797400	2.64881300
H	-1.24724800	0.83331900	4.10706600
C	-1.23142600	3.25490500	0.03855500
C	-1.22570000	4.50158200	0.68128800
C	-0.13595400	2.85608100	-0.74198000
C	-0.13312800	5.35058800	0.54197800
H	-2.07253700	4.78496100	1.29574900
C	0.95869500	3.69752900	-0.88761500
H	-0.15097500	1.87755700	-1.20718200
C	0.94591400	4.93714400	-0.24273200
H	-0.10674900	6.31449600	1.03462600
H	1.81572400	3.40400800	-1.48088100
N	2.10205100	5.82020100	-0.38493100
O	2.06730200	6.92116000	0.17120100
O	3.05954100	5.42171500	-1.05451800
H	-1.73338800	-3.57451000	0.47555000
C	-3.84747300	-2.07912900	2.80031000
C	-5.03429100	-1.87666000	1.83883500
H	-3.68053800	-1.17253200	3.39081800
H	-4.08855400	-2.87259600	3.52566900
H	-4.99378900	-0.86677500	1.40871800
H	-5.97843800	-1.92422500	2.39186300

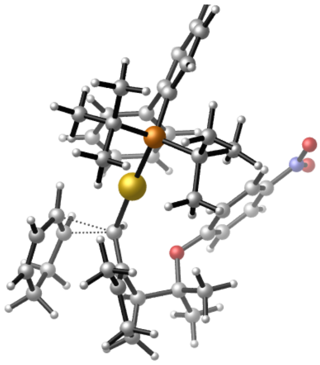

TS$_{XIIIa\text{-}XVa}$

E (opt) = -2390.19702047 Hartrees
G (opt) = -2389.408475 Hartrees
E (SP) = -2390.690848 Hartrees

Au	-0.16787800	-0.99242500	0.06510300
P	1.94902400	-1.77490200	-0.68929600
C	-2.09706700	-0.32005000	0.59116200
H	-1.93518000	0.55535800	1.21333400
C	-2.41844400	-2.03630900	1.86137800
H	-1.40026500	-2.21562200	2.18286700
C	-3.13670000	-0.12909400	-0.42331600
C	-3.45945500	-0.94613400	-1.46054000
C	1.93473400	-3.67636100	-0.52161800
C	2.17546400	-1.18616700	-2.50377900
C	-5.30347900	-1.84740100	1.37868300
H	-6.22838600	-2.20548300	1.84597700
C	-4.59345400	-0.85181600	2.32470000
H	-4.86579600	-1.07867100	3.36636600
C	-4.61579100	-0.39589300	-2.25923500
H	-4.27275500	-0.10533700	-3.26250200
H	-5.38917600	-1.15908700	-2.41421200
C	-3.96163700	1.16600000	-0.47400400
H	-4.33153700	1.43836100	0.52007300
C	-2.81425800	-2.21168500	-1.92552300
H	-2.01056800	-2.54496900	-1.27008100
H	-3.55036400	-3.01863400	-2.01168600
H	-2.40050600	-2.05592600	-2.93185600
C	-5.12494900	0.80462200	-1.43304300
H	-5.99395400	0.50588700	-0.83948000
H	-5.44495600	1.63858700	-2.06176800
C	-3.13173900	2.39458500	-0.95328500
C	-2.52400800	2.20221600	-2.34243500
H	-3.32440100	2.10843000	-3.08033000
H	-1.91757000	3.06538500	-2.62840000
H	-1.90127600	1.30593900	-2.38871900
C	-3.95765300	3.68181400	-0.89523100
H	-4.39145100	3.81610900	0.10023400
H	-3.32595400	4.54657100	-1.11979400
H	-4.76688200	3.66484400	-1.62984700
O	-2.08082800	2.50357300	0.08017400
C	-3.08958200	-0.91609000	2.32936100
C	0.77839100	-1.13792600	-3.16533700
H	0.12205300	-0.41176200	-2.67601400
H	0.89763500	-0.82984100	-4.21106800
H	0.27568300	-2.10736100	-3.15886400
C	2.73135800	0.25072300	-2.44067600
H	2.15439400	0.87663900	-1.75391400
H	3.77723900	0.27663700	-2.12648500
H	2.66597700	0.70068400	-3.43810400
C	3.09103500	-2.06009100	-3.37833700
H	2.69247900	-3.06834900	-3.51108500
H	3.16052500	-1.60079900	-4.37175100
H	4.10778700	-2.13548500	-2.98986700
C	0.82846700	-4.23830300	-1.43451400
H	0.72043600	-5.31187800	-1.23964100
H	-0.13889300	-3.76479200	-1.24310200
H	1.06633700	-4.11791100	-2.49437700
C	3.27179500	-4.37147200	-0.83653900
H	3.13000200	-5.45319000	-0.72500000
H	3.61946400	-4.19083500	-1.85426700
H	4.05885900	-4.07388900	-0.13965800
C	1.57731100	-3.97503300	0.95003500
H	2.32970500	-3.57700600	1.63775500
H	0.60235600	-3.56343700	1.22819300
H	1.54019400	-5.06167900	1.09213200
C	3.43793100	-1.18984100	0.24189000
C	3.36608900	-0.39248200	1.41136300
C	4.71217900	-1.49051000	-0.28142100
C	4.55992500	0.08440800	1.98287200
C	5.88320100	-1.01874300	0.30538000
H	4.80027500	-2.09301900	-1.17483900
C	5.80711000	-0.21821700	1.44455400
H	4.49317400	0.70192200	2.87354500
H	6.84440900	-1.27054600	-0.13256100
H	6.70870900	0.16723200	1.91150000
C	2.11192200	0.01620400	2.11671300
C	1.43957000	-0.86371200	2.97932000
C	1.65888900	1.33925400	2.00979000
C	0.32974500	-0.42954900	3.70801000
H	1.79231600	-1.88423500	3.08367600
C	0.54297700	1.77046800	2.72984800
H	2.17958700	2.02820400	1.35324200
C	-0.12192100	0.88815700	3.58418000
H	-0.17678000	-1.11843400	4.37819000
H	0.19928300	2.79454500	2.62291700
H	-0.97849900	1.22751400	4.15979100
C	-0.98585300	3.29305000	-0.10361700
C	-0.94870300	4.56863800	0.48060100
C	0.13984300	2.79309600	-0.77687200
C	0.20062900	5.34550000	0.38844200
H	-1.82221600	4.93083600	1.01085200
C	1.28873000	3.56473900	-0.88119900
H	0.10405600	1.79163400	-1.18815000
C	1.30607700	4.83342100	-0.29535700
H	0.24913700	6.33046600	0.83579100
H	2.16834200	3.19308700	-1.39174700
N	2.52040800	5.63978600	-0.39011400
O	2.52095600	6.75783000	0.13281700
O	3.49049700	5.16472300	-0.98826900
H	-2.58150900	-0.26768200	3.03424100
C	-3.13674600	-3.10946500	1.18887400
C	-4.46289300	-3.02707900	0.97894100
H	-5.62143100	-1.34291500	0.46014200
H	-4.94127700	0.16930800	2.15150900
H	-4.97470700	-3.84085800	0.46983200
H	-2.56921800	-3.97176500	0.85501800

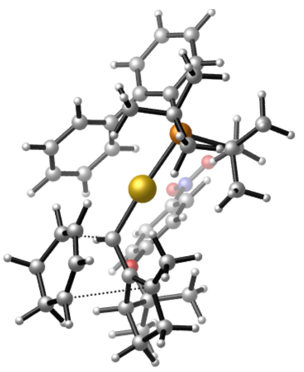

TS$_{XIIIa\text{-}XVIa}$

E (opt) = -2390.20085149 Hartrees
G (opt) = -2389.412762 Hartrees
E (SP) = -2390.694552 Hartrees

Au	-0.14297300	-0.98040400	0.05393500
P	2.01186900	-1.73307500	-0.68458800
C	-2.06593800	-0.36057900	0.53971600

H	-2.04659500	0.48718200	1.22292900
C	-2.72216000	-1.42807700	2.11740700
H	-1.81826400	-1.21900100	2.67969600
C	-3.12183300	-0.19646900	-0.45237600
C	-3.46109200	-1.04919100	-1.46450000
C	2.02047000	-3.63526600	-0.53241500
C	2.23365400	-1.13099000	-2.49658300
C	-5.15739800	-2.42446000	1.06207300
H	-6.05944000	-2.76126600	0.55548800
C	-4.02174600	-3.16973300	0.96554400
H	-4.01839100	-4.09861000	0.40540300
C	-4.60233900	-0.51370700	-2.29023800
H	-4.22982300	-0.22186700	-3.28303400
H	-5.36995600	-1.27635900	-2.46758900
C	-3.97218200	1.07839800	-0.51980700
H	-4.34817100	1.34724800	0.47492300
C	-2.79008300	-2.31907500	-1.86804600
H	-2.09137100	-2.68537100	-1.11630800
H	-3.52275400	-3.10141600	-2.09315700
H	-2.21809800	-2.14644800	-2.79115300
C	-5.12799400	0.68937000	-1.47879900
H	-5.99478900	0.38035300	-0.88720900
H	-5.45845100	1.51262800	-2.11582100
C	-3.16066600	2.31843500	-1.00120600
C	-2.53970300	2.12411100	-2.38457100
H	-3.33309100	2.00517300	-3.12640500
H	-1.94981900	2.99690600	-2.67599000
H	-1.89731500	1.24114100	-2.41698600
C	-4.00921000	3.59157500	-0.96052700
H	-4.46215200	3.72139100	0.02691100
H	-3.38642400	4.46526500	-1.17566800
H	-4.80489400	3.56017400	-1.70940500
O	-2.12433000	2.45117500	0.04008800
C	-2.79798400	-2.67186000	1.49503600
C	0.83801000	-1.09804400	-3.16232100
H	0.17026400	-0.38444100	-2.67049200
H	0.95571300	-0.78187500	-4.20592300
H	0.34884700	-2.07456900	-3.16332500
C	2.76579600	0.31448500	-2.42175800
H	2.17931100	0.92372800	-1.72790800
H	3.81147000	0.35520000	-2.10844700
H	2.69158400	0.77318700	-3.41469800
C	3.16233400	-1.98411500	-3.37768100
H	2.77675000	-2.99628900	-3.51957200
H	3.22715000	-1.51558900	-4.36722800
H	4.17962400	-2.05062700	-2.98929300
C	0.92146300	-4.20321300	-1.45112500
H	0.81586300	-5.27690400	-1.25485700
H	-0.04943700	-3.73231100	-1.26893800
H	1.16712400	-4.08504100	-2.50959500
C	3.36435300	-4.31560800	-0.84905100
H	3.23494800	-5.39959300	-0.74322800
H	3.71093000	-4.12525000	-1.86559800
H	4.14768600	-4.01232100	-0.15031300
C	1.66195200	-3.94449100	0.93716500
H	2.40921400	-3.54418900	1.62908000
H	0.68458100	-3.53883600	1.21618400
H	1.63154700	-5.03203400	1.07466700
C	3.50341900	-1.14134600	0.24036600
C	3.42516900	-0.36007300	1.41940600
C	4.78041800	-1.42016800	-0.28817500
C	4.61266200	0.12496100	1.99638400
C	5.94625200	-0.93917300	0.30181100
H	4.87475500	-2.01433100	-1.18643800
C	5.86248300	-0.15340400	1.45080600
H	4.53936600	0.72878700	2.89597300
H	6.90987600	-1.17353900	-0.14060000
H	6.76019200	0.23812100	1.92026900
C	2.16453300	0.00871100	2.13476700
C	1.56915200	-0.87945200	3.04284000
C	1.62751400	1.29619300	1.99184500
C	0.44917700	-0.49067000	3.78089900
H	1.99331600	-1.86914500	3.17710200
C	0.49831700	1.68009300	2.71856200
H	2.09120800	1.99322700	1.30201300
C	-0.09321400	0.78787900	3.61586600
H	0.00464900	-1.18404700	4.48957200
H	0.08587200	2.67501900	2.58243200
H	-0.96621800	1.08729200	4.18898700
C	-1.04277900	3.26155700	-0.12852500
C	-1.03001900	4.52329300	0.48613400
C	0.09344800	2.79877200	-0.81108200
C	0.10505600	5.32291200	0.41658700
H	-1.91112700	4.85606900	1.02314300
C	1.22778900	3.59451700	-0.89429000
H	0.07962300	1.80623800	-1.24422000
C	1.22086400	4.84863300	-0.27752000
H	0.13433600	6.29691900	0.88877600
H	2.11498500	3.25155000	-1.41192500
N	2.42081500	5.67836800	-0.34852800
O	2.39908100	6.78455900	0.19879800
O	3.40216500	5.23367600	-0.95173400
H	-1.88109900	-3.22662300	1.31757300
C	-3.99791700	-0.82363500	2.67333800
C	-5.25911900	-1.15688800	1.84860200
H	-3.88846300	0.25816900	2.78888700
H	-4.10998700	-1.23181000	3.68658800
H	-5.50995600	-0.35003100	1.15123700
H	-6.13435000	-1.23225000	2.50660100

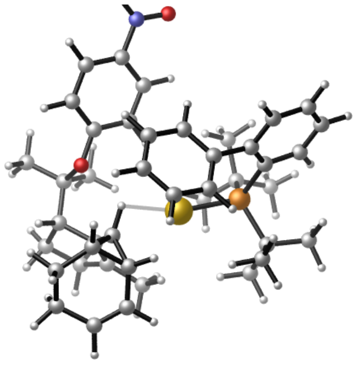

XVa

E (opt) = -2390.22451268 Hartrees
G (opt) = -2389.436808 Hartrees
E (SP) = -2390.714453 Hartrees

Au	0.00313700	-0.95145200	0.12963800
P	2.04112500	-1.67915200	-0.68771600
C	-2.42280800	-0.51916600	0.83860800
H	-1.61692200	0.24010600	0.85421800
C	-2.33612700	-1.70941500	1.87214900
H	-1.36641700	-1.85718700	2.33902400
C	-3.23409200	-0.49563400	-0.41522400
C	-3.35272800	-1.44181200	-1.36677100
C	2.11655300	-3.56769900	-0.42052400
C	2.14135200	-1.13232100	-2.52266400
C	-5.28973800	-1.69452000	1.71046800
H	-6.24798700	-1.90621900	2.19905800
C	-4.57168100	-0.56005900	2.47029200
H	-4.68547100	-0.73760400	3.54739900
C	-4.36458800	-1.04485800	-2.41955000
H	-3.86203700	-0.82083500	-3.37081500
H	-5.06581600	-1.86263400	-2.62868500
C	-4.07342700	0.74044600	-0.75202300
H	-4.60410200	1.10306400	0.13466300
C	-2.62747700	-2.74144200	-1.50523900
H	-1.86768100	-2.87641000	-0.73527800
H	-3.32189900	-3.58885600	-1.45739800
H	-2.13464900	-2.79163800	-2.48528500
C	-5.06384800	0.19074000	-1.81223500
H	-5.97839100	-0.12461900	-1.30078900
H	-5.35493900	0.92924100	-2.56329600
C	-3.23767700	1.94958700	-1.26328800
C	-2.50939400	1.65927300	-2.57477400
H	-3.23781900	1.50615200	-3.37426900

H	-1.87707700	2.50021300	-2.86912100
H	-1.89145800	0.76201600	-2.49363600
C	-4.09905200	3.20741600	-1.38296700
H	-4.59425000	3.42466600	-0.43179500
H	-3.48454100	4.06934800	-1.66099200
H	-4.86540800	3.08407900	-2.15301500
O	-2.27365800	2.17075100	-0.16117700
C	-3.07252700	-0.46125900	2.22753300
C	0.71741600	-1.18670500	-3.11960400
H	0.05628200	-0.45035600	-2.65331700
H	0.78000700	-0.94811100	-4.18733600
H	0.25593600	-2.17221500	-3.02356100
C	2.61910300	0.33302100	-2.52136100
H	2.04570300	0.94353500	-1.81767400
H	3.67756900	0.42399500	-2.26732100
H	2.47362700	0.75113100	-3.52372400
C	3.06913900	-1.98944500	-3.40204200
H	2.71843500	-3.01985800	-3.48975800
H	3.07311600	-1.55740500	-4.40935300
H	4.10293500	-1.99817500	-3.05350600
C	1.02216800	-4.24047600	-1.26744400
H	0.99660000	-5.30718500	-1.01687400
H	0.03134300	-3.82816500	-1.06217400
H	1.21807300	-4.15895200	-2.33893400
C	3.48738600	-4.18542800	-0.75602800
H	3.41450400	-5.26771700	-0.59841400
H	3.78794800	-4.02390200	-1.79211500
H	4.27474600	-3.81270600	-0.09727800
C	1.82882800	-3.81035700	1.07501800
H	2.57423500	-3.32785300	1.71389500
H	0.83555800	-3.45492100	1.36557200
H	1.87150200	-4.88844600	1.26765600
C	3.50326500	-0.96988300	0.18982500
C	3.43928500	-0.15011400	1.34433800
C	4.76748100	-1.23425600	-0.37667000
C	4.63639600	0.37163700	1.86721100
C	5.94001700	-0.71374300	0.16261200
H	4.84771000	-1.85209800	-1.26022900
C	5.87443100	0.09968600	1.29283100
H	4.57893000	1.00245800	2.74893400
H	6.89338500	-0.94023200	-0.30477500
H	6.77758100	0.51973700	1.72532400
C	2.19735100	0.24703100	2.07615100
C	1.55849100	-0.62593000	2.97164500
C	1.72214600	1.56111600	1.95190900
C	0.45352800	-0.19508600	3.71050700
H	1.93404900	-1.63598500	3.09557100
C	0.61112900	1.98809100	2.68246700
H	2.22313800	2.24559000	1.27544700
C	-0.02481700	1.11111400	3.56452500
H	-0.02878600	-0.87711200	4.40466900
H	0.24864500	3.00441200	2.56327100
H	-0.87864900	1.44631100	4.14582600
C	-1.25505800	3.06746900	-0.28508000
C	-1.37388200	4.33109800	0.31314600
C	-0.05228500	2.69514600	-0.90645600
C	-0.30392100	5.21837900	0.28851500
H	-2.30504400	4.59756200	0.80026300
C	1.01996400	3.57546800	-0.94110500
H	0.03406900	1.70663600	-1.33990600
C	0.88141300	4.82930900	-0.34113500
H	-0.37610300	6.19656700	0.74726700
H	1.95533300	3.29858500	-1.41126600
N	2.01323700	5.75298600	-0.36396800
O	1.86891300	6.86579900	0.14942200
O	3.06199400	5.37511400	-0.89485300
H	-2.56468700	0.20858300	2.91537000
C	-3.12845000	-2.94827500	1.66475400
C	-4.46669400	-2.95125900	1.61506700
H	-5.53900600	-1.36236300	0.69479400
H	-5.05876000	0.39812200	2.26188500
H	-4.99627200	-3.88948200	1.46203500
H	-2.57161800	-3.87641100	1.56039900

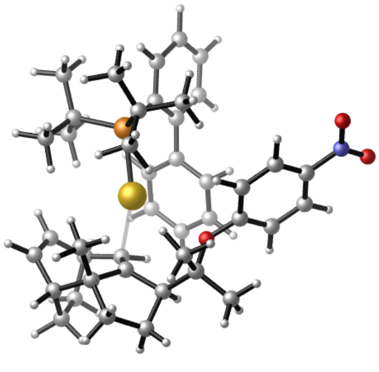

XVIa

E (opt) = -2390.25621811 Hartrees
G (opt) = -2389.456761 Hartrees
E (SP) = -2390.744754 Hartrees

Au	-0.80387900	-0.36458700	-0.04498000
P	0.38850200	-2.27522200	-0.77166600
C	-2.38984800	0.82619500	1.15836300
H	-1.74022700	1.20348500	1.94835900
C	-3.44907000	-0.14458600	1.67803200
H	-3.01654500	-0.66779300	2.53342700
C	-2.49599700	1.62203100	0.03614800
C	-3.62163600	1.50608700	-1.02322700
C	-0.83813100	-3.73746400	-0.74148400
C	1.11400000	-1.92293600	-2.51745800
C	-4.91756300	0.83575500	-0.43467200
H	-5.70588900	1.05849000	-1.16121500
C	-4.75980500	-0.66313700	-0.31298800
H	-5.22875700	-1.31842600	-1.04236200
C	-3.88763100	2.99798400	-1.40667900
H	-3.55069100	3.17286500	-2.43231000
H	-4.95584000	3.23194500	-1.38221800
C	-1.88829000	3.03826900	0.06109800
H	-1.67296700	3.27308600	1.10687700
C	-3.21417700	0.72473900	-2.29063700
H	-2.96469600	-0.31316700	-2.06659500
H	-4.05134600	0.72849400	-2.99885400
H	-2.35869700	1.18040100	-2.78874900
C	-3.09576900	3.88139000	-0.42818800
H	-3.71333300	4.12487700	0.44155900
H	-2.79597700	4.82950100	-0.87936000
C	-0.54705600	3.35288300	-0.65590400
C	-0.52623400	3.07707200	-2.15494200
H	-1.34323600	3.60817000	-2.64929700
H	0.41078000	3.42203400	-2.59994100
H	-0.62886900	2.00953400	-2.34831100
C	-0.14065000	4.80319900	-0.37238000
H	-0.16084100	5.01842300	0.70013300
H	0.86850900	4.99453200	-0.74666100
H	-0.81278600	5.49798600	-0.88010600
O	0.38954600	2.45311800	0.03841100
C	-4.01850600	-1.13686900	0.69029200
C	0.19077100	-0.88950700	-3.19888500
H	0.20591700	0.06944500	-2.67551700
H	0.54831300	-0.71812800	-4.22087200
H	-0.84615000	-1.22871000	-3.25860100
C	2.50689000	-1.29065300	-2.32079500
H	2.51201500	-0.53687700	-1.53075900
H	3.26608200	-2.03559300	-2.07506800
H	2.80642600	-0.80263300	-3.25538200
C	1.23284700	-3.14276300	-3.44902400
H	0.25861500	-3.56302500	-3.70726800
H	1.70268600	-2.80752900	-4.38146700
H	1.86045500	-3.93741700	-3.04218900
C	-2.01809000	-3.40686500	-1.67424000
H	-2.75686700	-4.21409900	-1.60584400
H	-2.51617600	-2.47759000	-1.38620800
H	-1.71110300	-3.33087900	-2.72022700

C	-0.22568500	-5.09485400	-1.13322200
H	-1.00743200	-5.85714700	-1.03270200
H	0.12738100	-5.12687700	-2.16348000
H	0.59374300	-5.38305200	-0.47073100
C	-1.34311700	-3.85221600	0.71105600
H	-0.54484100	-4.17310700	1.38603200
H	-1.75502600	-2.91253300	1.08477000
H	-2.13501400	-4.60932100	0.74765100
C	1.79592300	-2.79192200	0.30920600
C	2.06023500	-2.24859900	1.58988600
C	2.67452100	-3.77085600	-0.19860500
C	3.20373100	-2.68383300	2.28491900
C	3.79443900	-4.19571400	0.51017100
H	2.49263300	-4.20680300	-1.17189100
C	4.06701300	-3.64087800	1.75989000
H	3.40237200	-2.25879200	3.26420900
H	4.44898600	-4.94925600	0.08295600
H	4.94020800	-3.95261200	2.32537700
C	1.21528200	-1.25659800	2.32779300
C	0.12320700	-1.69203000	3.09571900
C	1.60453500	0.08946400	2.40459300
C	-0.57199600	-0.79842000	3.91244600
H	-0.16377700	-2.73692600	3.07039800
C	0.90804500	0.98169100	3.22223500
H	2.45521200	0.43411800	1.82540200
C	-0.18004000	0.54154700	3.97959100
H	-1.40765500	-1.15266300	4.50919600
H	1.22276200	2.01856400	3.27517400
H	-0.71260800	1.23456300	4.62476000
C	1.73923200	2.60396800	-0.10663700
C	2.46531000	3.22238800	0.92274300
C	2.40927500	2.06981000	-1.21559800
C	3.85109300	3.28995000	0.85859500
H	1.92947700	3.64020700	1.76651100
C	3.79572600	2.13365300	-1.29101000
H	1.84046600	1.58977400	-2.00000700
C	4.50075800	2.74003300	-0.24967500
H	4.42666300	3.75764000	1.64765900
H	4.32904300	1.71190100	-2.13365900
N	5.95972600	2.79580900	-0.31831300
O	6.56680300	3.32353700	0.61747200
O	6.51567700	2.31153500	-1.30809200
H	-3.86102300	-2.19868900	0.84957600
C	-4.63136100	0.75492100	2.15456000
C	-5.34933900	1.41856500	0.94676000
H	-4.26118800	1.51375600	2.85259800
H	-5.32767400	0.12019000	2.71174800
H	-5.18510100	2.49987400	0.95145800
H	-6.42885000	1.27496400	1.04923500

C	2.22726600	0.38413600	0.39573800
C	3.07083100	1.19812200	1.05733900
C	3.79324300	-1.78562100	-1.67643200
H	4.19319500	-1.71198300	-2.68624800
C	2.50112800	-2.09402300	-1.49487400
H	1.86267400	-2.27690400	-2.35764200
C	3.21920200	2.53551700	0.36718200
H	2.72563700	3.32731000	0.94934800
H	4.27197400	2.83359600	0.27636100
C	1.61211700	1.08472000	-0.81626000
H	1.64578800	0.42631200	-1.69007300
C	3.80518400	0.92574700	2.33390900
H	3.54251500	-0.04214200	2.76571700
H	4.89217200	0.95511100	2.17951500
H	3.57960400	1.70284300	3.07690600
C	2.54920900	2.30869800	-1.00603700
H	3.31429200	2.04567600	-1.74352100
H	2.03770400	3.19934000	-1.37990300
C	0.11374700	1.46337000	-0.64492400
C	-0.13873000	2.40396800	0.53224000
H	0.35384500	3.36333100	0.35306000
H	-1.20724700	2.60157000	0.65453600
H	0.25166900	1.98277000	1.46185800
C	-0.45615300	2.04166600	-1.94211200
H	-0.29963200	1.34775500	-2.77364700
H	-1.52885700	2.23425800	-1.84599400
H	0.02805300	2.99015400	-2.18761100
O	-0.52243700	0.15331200	-0.39510700
C	1.86306100	-2.19410600	-0.15744100
C	-1.86589500	0.04450900	-0.21110300
C	-2.68964300	-0.28657500	-1.30008000
C	-2.41863400	0.15630700	1.07619500
C	-4.05095100	-0.48909600	-1.11337500
H	-2.24368300	-0.38722700	-2.28294700
C	-3.77899900	-0.04404100	1.27284000
H	-1.76985600	0.39214000	1.91173800
C	-4.58124700	-0.36190800	0.17349400
H	-4.69849300	-0.74438700	-1.94301300
H	-4.22011600	0.03667400	2.25841100
N	-6.01101400	-0.57638400	0.37751700
O	-6.70760900	-0.84921600	-0.60497900
O	-6.45978200	-0.47433800	1.52355500
H	0.98547000	-2.83455400	-0.11429000
C	4.28441800	-2.15579400	0.79083600
C	4.73054600	-1.49541900	-0.53247200
H	4.59419900	-3.20893400	0.76794700
H	4.81832700	-1.69477200	1.62872800
H	5.74444700	-1.83373000	-0.78133700
H	4.79995100	-0.40950500	-0.39768500

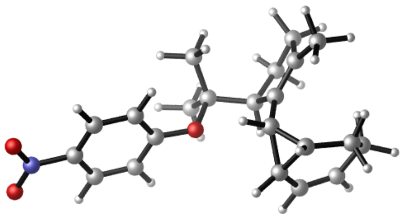

8d

E (opt) = -1134.975498 Hartrees
G (opt) = -1134.579427 Hartrees
E (SP) = -1135.261775 Hartrees

C	1.83826600	-0.99848300	0.80317200
H	0.95718600	-1.01793000	1.44309900
C	2.78333100	-2.15527700	1.04983400
H	2.49713300	-2.79865100	1.88030500

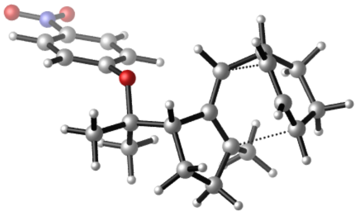

$TS_{8d\text{-}7d}$

E (opt) = -1134.925696 Hartrees
G (opt) = -1134.528794 Hartrees
E (SP) = -1135.213379 Hartrees

C	1.73825600	-0.81813700	-0.98367000
H	1.02399400	-0.62806700	-1.77982100
C	2.64015500	-1.99751700	-1.27309300
H	2.13916700	-2.66863100	-1.97409500

C	1.85783700	0.19878800	-0.03202100
C	2.63733400	0.21616000	1.13880100
C	4.70615700	-0.65364300	0.09104900
H	5.41907500	-0.07306300	0.67298100
C	4.48680300	-0.31142500	-1.23882600
H	4.92886400	0.59389000	-1.65209100
C	2.89110800	1.65412100	1.56507300
H	2.30069700	1.89251700	2.46207800
H	3.93905600	1.82219700	1.83854500
C	1.42719600	1.62522000	-0.41077800
H	1.52674300	1.78123000	-1.49128600
C	2.53081200	-0.77611600	2.26825900
H	2.23274600	-1.77383000	1.94903700
H	3.44952500	-0.84745900	2.85986300
H	1.75083000	-0.41468500	2.95553700
C	2.45185300	2.49985900	0.35535400
H	3.31006300	2.67909400	-0.30007200
H	2.05931300	3.47770400	0.64740800
C	-0.06043600	1.93214700	-0.09040200
C	-0.42019400	1.72830600	1.37985100
H	0.11610800	2.46112700	1.98787700
H	-1.48997100	1.88115100	1.54809300
H	-0.15251600	0.72676200	1.72444500
C	-0.46498800	3.32979100	-0.56253300
H	-0.26462700	3.44379700	-1.63212300
H	-1.53171000	3.49785700	-0.38709700
H	0.08518000	4.10470700	-0.02145300
O	-0.77433200	0.96176000	-0.95537900
C	3.50664700	-0.98517500	-1.96955000
C	-1.99792100	0.47976200	-0.61128400
C	-3.16888200	1.16680700	-0.97084300
C	-2.08218300	-0.76280700	0.03855200
C	-4.41350600	0.62585100	-0.67158900
H	-3.08998900	2.11272500	-1.49406400
C	-3.32113700	-1.31214200	0.33913900
H	-1.16280900	-1.27881600	0.29279900
C	-4.47494500	-0.60651200	-0.01576300
H	-5.32742200	1.14106900	-0.93995800
H	-3.40539200	-2.26808700	0.84084200
N	-5.78086100	-1.17806900	0.29902900
O	-6.79021300	-0.56079800	-0.05510100
O	-5.81738900	-2.25333300	0.90537000
H	3.30343100	-0.73869700	-3.00642200
C	3.34771600	-2.82447400	-0.18781300
C	4.53187800	-2.10148200	0.50261600
H	3.72193100	-3.73192100	-0.67406300
H	2.60629000	-3.15960300	0.54471300
H	5.46586500	-2.61254900	0.22522700
H	4.46970600	-2.19924500	1.58760600

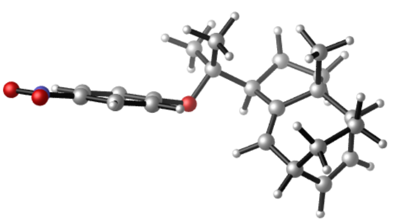

7d

E (opt) = -1134.988980 Hartrees
G (opt) = -1134.587503 Hartrees
E (SP) = -1135.275066 Hartrees

C	1.71410100	-0.95868600	-0.83326300
H	0.80542100	-0.97456100	-1.42913000
C	2.61380200	-2.18663000	-0.92050400
H	2.05666700	-2.98767600	-1.41453900

C	2.05783500	0.18626400	-0.22846600
C	3.36303400	0.43290200	0.53636200
C	4.52591600	-0.58497300	0.23921500
H	5.44012200	-0.10894800	0.61468800
C	4.69308300	-0.92796100	-1.22772300
H	5.53971700	-0.53779300	-1.78777100
C	3.70462700	1.86750600	0.05497000
H	4.44026400	2.35960000	0.70112000
H	4.12711200	1.81859000	-0.95643500
C	1.34823200	1.52463900	-0.48386900
H	1.25039700	1.63829600	-1.57069500
C	3.11484100	0.46865200	2.06513500
H	2.62667500	-0.44181800	2.42116000
H	4.06702000	0.57981900	2.59771000
H	2.48009600	1.30818700	2.35304400
C	2.34913000	2.59616100	0.02204000
H	2.36031900	3.48003700	-0.62156100
H	2.08231000	2.93913500	1.02662200
C	-0.09756500	1.67037400	0.04814400
C	-0.25810300	1.27441400	1.51312400
H	0.31898000	1.95489300	2.14394400
H	-1.30405300	1.34422500	1.82493700
H	0.09317200	0.25523600	1.68749600
C	-0.63199900	3.08326900	-0.19721600
H	-0.54382300	3.35112200	-1.25453300
H	-1.68375300	3.15424700	0.09489700
H	-0.07501500	3.81411300	0.39571100
O	-0.84477300	0.73497900	-0.82428900
C	3.80082300	-1.76142900	-1.77716600
C	-2.11647600	0.35742600	-0.51852000
C	-3.21050500	1.09769600	-0.99583300
C	-2.33206600	-0.83295000	0.19574500
C	-4.50768000	0.66816400	-0.74361600
H	-3.02915900	1.99776800	-1.57168600
C	-3.62484100	-1.27112500	0.45078100
H	-1.47454500	-1.40204400	0.53672400
C	-4.69979200	-0.50937400	-0.01675000
H	-5.36263100	1.22699100	-1.10332200
H	-3.80859100	-2.18566500	1.00086900
N	-6.06139100	-0.96269400	0.25305300
O	-6.99942400	-0.28936400	-0.18472500
O	-6.21312700	-1.99855300	0.90787100
H	3.85960900	-2.08038400	-2.81528100
C	3.09398300	-2.69390300	0.47319100
C	4.34808900	-1.94229200	0.97010800
H	3.30578600	-3.76705700	0.41355900
H	2.26782700	-2.57314700	1.18221200
H	5.24242100	-2.53774200	0.75781000
H	4.31516500	-1.81190600	2.05489700

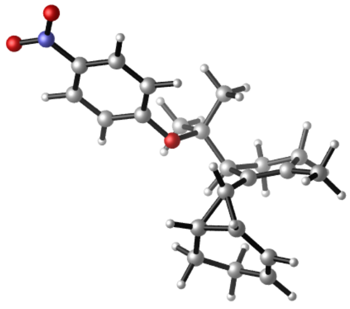

8d'

E (opt) = -1134.97594026 Hartrees
G (opt) = -1134.580069 Hartrees
E (SP) = -1135.262474 Hartrees

C	1.81324800	-1.01505200	0.76014600
H	0.92118800	-1.04224700	1.38380700
C	2.78158800	-2.17693400	1.05883600
H	2.49679800	-2.79958900	1.90397000

C	2.22591700	0.37299400	0.39995000
C	3.08467300	1.15822000	1.07640400
C	3.93561300	-1.47820200	-1.57666300
H	4.47111700	-1.77399600	-2.48724300
C	2.57001900	-2.19806100	-1.53079600
H	2.72823100	-3.24820900	-1.80955100
C	3.24741900	2.51059700	0.41989800
H	2.77428600	3.29355500	1.03059300
H	4.30380000	2.79418700	0.32455000
C	1.61668700	1.11041200	-0.79433600
H	1.64401200	0.48112100	-1.69073400
C	3.81145600	0.85569800	2.35021200
H	3.53772600	-0.11648900	2.76402800
H	4.89868500	0.87292900	2.19977200
H	3.58961300	1.62354000	3.10407600
C	2.55826300	2.33603600	-0.95112400
H	3.31169400	2.09992000	-1.70928100
H	2.04558100	3.24179500	-1.28465500
C	0.11865400	1.48785800	-0.61620600
C	-0.13283400	2.38380200	0.59539000
H	0.36785600	3.34556100	0.45490700
H	-1.20042000	2.58446500	0.72113600
H	0.25091600	1.92457600	1.50957500
C	-0.44468900	2.11703200	-1.89243000
H	-0.28488700	1.45578300	-2.74966300
H	-1.51774400	2.30594700	-1.79312400
H	0.04004500	3.07443300	-2.09907200
O	-0.52133600	0.17092600	-0.42220600
C	1.88769500	-2.19982600	-0.17011900
C	-1.86326600	0.05735500	-0.22870700
C	-2.70013600	-0.21816200	-1.32293900
C	-2.40046200	0.10684000	1.06891700
C	-4.05961200	-0.42742900	-1.13039400
H	-2.26579100	-0.27189800	-2.31462400
C	-3.75882700	-0.10115100	1.27145500
H	-1.74128000	0.30069200	1.90710000
C	-4.57443600	-0.36347800	0.16729000
H	-4.71742500	-0.64095400	-1.96372600
H	-4.18824300	-0.06884300	2.26494100
N	-6.00244600	-0.58432500	0.37710100
O	-6.71015400	-0.81238500	-0.60883800
O	-6.43840600	-0.53162500	1.53134400
H	1.02988200	-2.86817800	-0.12449200
C	4.24407200	-2.06329100	0.82959400
C	4.77948100	-1.75661100	-0.36041400
H	3.78294900	-0.39447900	-1.66071100
H	1.90237800	-1.77387100	-2.28994100
H	5.86008400	-1.67606000	-0.46339600
H	4.89211300	-2.24062400	1.68586600

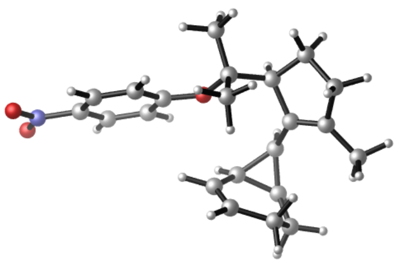

8d' turned

E (opt) = -1134.968263 Hartrees
G (opt) = -1134.571247 Hartrees
E (SP) = -1135.254333 Hartrees

C	2.13240400	-0.91227400	-1.27727000
H	2.02455200	-0.69996500	-2.34415100
C	0.99420900	-1.85248700	-0.83843700
H	0.24883100	-2.03683600	-1.60792800

C	2.64940000	0.31445600	-0.59414000
C	3.88526600	0.51965000	-0.09228800
C	2.67571300	-2.47156900	1.52154000
H	2.98571800	-3.16127700	2.31605100
C	3.04483600	-3.07133600	0.15184100
H	2.69297400	-4.11191600	0.13345700
C	4.08968400	1.95460300	0.34012300
H	4.01322600	2.04525400	1.43367000
H	5.08770200	2.32251500	0.07197900
C	1.88603600	1.64810800	-0.73638700
H	1.57708500	1.75208600	-1.78552600
C	5.04078600	-0.42888700	0.00774600
H	4.89280200	-1.33141800	-0.58316100
H	5.95160800	0.06548100	-0.35318000
H	5.24214600	-0.72434600	1.04636600
C	2.96880500	2.71369800	-0.39114800
H	3.36199800	3.12925000	-1.32401900
H	2.58509700	3.55334700	0.19096200
C	0.56479200	1.83577500	0.06880800
C	0.77691400	1.55397900	1.55736200
H	1.62493900	2.14722900	1.91084000
H	-0.08212100	1.82854700	2.17217700
H	1.00351500	0.49922200	1.71620200
C	-0.02900500	3.22683000	-0.18942100
H	-0.06161000	3.43578200	-1.26324900
H	-1.04488400	3.30411400	0.20294900
H	0.56524600	4.00229400	0.29845900
O	-0.31507800	0.84795300	-0.57342500
C	2.39707000	-2.39168600	-1.04841400
C	-1.61411900	0.62158800	-0.32638400
C	-2.23764900	-0.22360200	-1.27445000
C	-2.37172300	1.10303000	0.76107500
C	-3.56440900	-0.58597400	-1.14266100
H	-1.64369100	-0.58093100	-2.10802100
C	-3.70743900	0.74420700	0.89067800
H	-1.93245600	1.74871400	1.50561900
C	-4.29867000	-0.09661300	-0.05416100
H	-4.04013200	-1.23511100	-1.86721000
H	-4.29326900	1.11042000	1.72479300
N	-5.69294800	-0.46756200	0.09177500
O	-6.19259000	-1.21738400	-0.75630100
O	-6.32431100	-0.01925000	1.05695000
H	2.55526900	-2.94933400	-1.97041100
C	0.46024600	-1.88958300	0.54563600
C	1.20274400	-2.17982000	1.62270300
H	3.23749600	-1.54547700	1.68945100
H	4.13278100	-3.11656200	0.04535300
H	0.74524100	-2.18417900	2.61033000
H	-0.59894300	-1.67708300	0.66388100

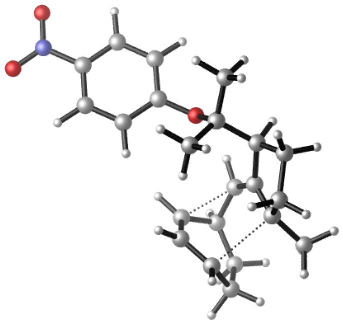

TS$_{8d'-7d'}$

E (opt) = -1134.918364 Hartrees
G (opt) = -1134.520180 Hartrees
E (SP) = -1135.205987 Hartrees

C	1.89305400	-0.55815600	-1.35796300
H	0.99975500	-0.36434600	-1.94235500
C	1.33769100	-2.06322800	-0.06773300
H	0.26760700	-2.18545500	-0.18912100

C	2.38852800	0.53474000	-0.64352200
C	3.57315400	0.61789200	0.11065200
C	4.19038500	-2.44230200	0.36040700
H	4.32091500	-3.43802800	0.81069400
C	3.62391000	-2.61687700	-1.07214100
H	3.55343400	-3.68718100	-1.29547200
C	3.48559900	1.83186100	1.02817600
H	3.05324500	1.55907500	1.99779500
H	4.46291200	2.28490200	1.22848100
C	1.56638100	1.84397900	-0.50885200
H	1.38831100	2.22695900	-1.52174800
C	4.95623100	0.31896400	-0.40835100
H	4.97434300	-0.41363300	-1.21306400
H	5.35707600	1.25678100	-0.82408000
H	5.65608600	0.01329600	0.37643500
C	2.54682400	2.77770000	0.25409400
H	3.13584900	3.34455000	-0.47499500
H	2.05013000	3.50639300	0.89930700
C	0.15614600	1.79071600	0.14390800
C	0.19072800	1.33393200	1.60058400
H	0.74920600	2.05823500	2.19863300
H	-0.81659900	1.27517800	2.02166900
H	0.67475100	0.36268100	1.69282900
C	-0.55721800	3.14280700	0.01913500
H	-0.58642200	3.46934800	-1.02494200
H	-1.58555900	3.06563800	0.38599100
H	-0.05421500	3.91258100	0.60965000
O	-0.58112800	0.83476400	-0.70423400
C	2.21624000	-2.03522000	-1.28245000
C	-1.85708600	0.46307600	-0.42031800
C	-2.90715800	1.02275400	-1.16748900
C	-2.12973500	-0.54162100	0.52539000
C	-4.21474000	0.59934400	-0.96856400
H	-2.67649500	1.78582100	-1.90239800
C	-3.43477400	-0.96893100	0.73302100
H	-1.31189100	-0.98400200	1.08002600
C	-4.46463500	-0.39057200	-0.01421500
H	-5.03414100	1.02364000	-1.53534300
H	-3.66061400	-1.74340600	1.45534200
N	-5.83654400	-0.83881400	0.20211900
O	-6.73244900	-0.32994800	-0.47888800
O	-6.04015300	-1.70648700	1.05721700
H	1.75132800	-2.53285800	-2.13659100
C	1.91163500	-1.83021100	1.18320700
C	3.28620800	-1.67512700	1.29913500
H	5.19346500	-2.01406700	0.33845100
H	4.30371300	-2.19769300	-1.82099400
H	3.70813300	-1.41629000	2.26799900
H	1.26711900	-1.61617700	2.03353900

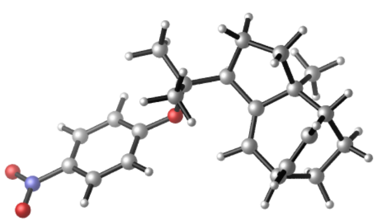

7d’

E (opt) = -1134.988160 Hartrees
G (opt) = -1134.586868 Hartrees
E (SP) = -1135.274287 Hartrees

C	1.64586200	-1.03591700	-0.31337400
H	0.57243400	-1.18278500	-0.35925100
C	3.14447800	-2.23667700	1.21902000
H	2.76300900	-2.86634400	2.01983200

C	2.12691300	0.21367200	-0.28751900
C	3.60934900	0.59139200	-0.20456200
C	4.85799000	-1.58320000	-0.92675100
H	5.64825200	-2.25327400	-0.57140900
C	3.59385500	-2.40780500	-1.26370600
H	3.86318100	-3.46229600	-1.38995500
C	3.55737700	1.71237700	0.85827900
H	3.48533900	1.25718800	1.85297600
H	4.45571700	2.33978400	0.84533900
C	1.32030500	1.51497600	-0.21302900
H	1.17952000	1.88898500	-1.23563900
C	4.06099800	1.20151700	-1.55535900
H	3.87964900	0.52031600	-2.39108000
H	3.52857200	2.13134900	-1.77556000
H	5.13183400	1.43570900	-1.52825600
C	2.26916400	2.50401400	0.54877400
H	2.47795300	3.37891900	-0.07302600
H	1.81517900	2.87947000	1.46828900
C	-0.10520200	1.46069800	0.38754000
C	-0.15069700	0.87435700	1.79927600
H	0.43129100	1.50528600	2.47722100
H	-1.17609600	0.84654000	2.17764200
H	0.26855100	-0.13213400	1.82902500
C	-0.75620900	2.84786700	0.35746400
H	-0.72417100	3.26660500	-0.65317200
H	-1.80093400	2.78800100	0.67646700
H	-0.24465900	3.53609600	1.03473300
O	-0.83811100	0.60252200	-0.57068300
C	2.50023600	-2.29105900	-0.15876200
C	-2.14896700	0.28911500	-0.37330100
C	-3.13681600	1.01243700	-1.06159100
C	-2.51267300	-0.80903300	0.42447000
C	-4.47477000	0.65834100	-0.94446000
H	-2.83607900	1.84520000	-1.68711800
C	-3.84845000	-1.16923400	0.54930900
H	-1.74148800	-1.37515700	0.93326000
C	-4.81592400	-0.42791200	-0.13440800
H	-5.24835100	1.20777500	-1.46631000
H	-4.14549000	-2.01359700	1.15883900
N	-6.22122100	-0.80177800	-0.00343100
O	-7.06100200	-0.14364400	-0.62495000
O	-6.50627700	-1.75782400	0.72430900
H	1.83862200	-3.15935400	-0.23065400
C	4.13147300	-1.35269900	1.40609500
C	4.59763900	-0.55483900	0.20473200
H	5.25073300	-1.09305500	-1.82178700
H	3.15767200	-2.07706300	-2.21221000
H	5.54736700	-0.05740000	0.43755000
H	4.61849100	-1.22506100	2.37003600

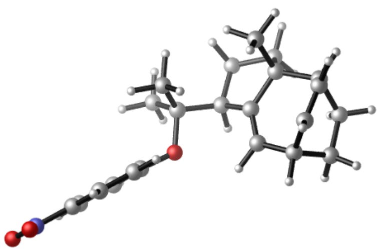

7d’’

E (opt) = -1134.99104826 Hartrees
G (opt) = -1134.588898 Hartrees
E (SP) = -1135.27683610 Hartrees

C	1.75093200	-1.04758300	-0.65016400
H	0.82587700	-1.12261700	-1.21629400
C	2.63453000	-2.28923700	-0.66318400
H	2.02884600	-3.14265100	-0.98440900

C	2.09485000	0.15592700	-0.17143300
C	3.40692100	0.49521900	0.54563000
C	4.55301700	-0.53271200	0.25786300
H	5.48299700	-0.05878800	0.59360900
C	4.30855400	-1.80180700	1.05108800
H	4.87991200	-1.99494600	1.95582900
C	3.69047300	1.91629800	-0.00485500
H	4.40527700	2.46415500	0.61907600
H	4.11083200	1.85676400	-1.01512900
C	1.33933400	1.45349100	-0.49740000
H	1.22924700	1.50122300	-1.58798500
C	3.20242900	0.60399500	2.07816000
H	2.79615700	-0.31930500	2.49592300
H	4.16353100	0.80589400	2.56753100
H	2.52210500	1.41520700	2.34320200
C	2.30515600	2.58722100	-0.06085000
H	2.27476100	3.43548400	-0.74993500
H	2.03412500	2.97216200	0.92713900
C	-0.10489800	1.58861300	0.04274400
C	-0.23387900	1.27944800	1.53212100
H	0.33040000	2.01405800	2.11201600
H	-1.27697600	1.33849400	1.85493700
H	0.15096500	0.28382000	1.76268900
C	-0.68205500	2.96905700	-0.28113400
H	-0.59627500	3.18336800	-1.35094600
H	-1.73756400	3.02113500	0.00143500
H	-0.15255100	3.74819300	0.27396300
O	-0.83596200	0.58130000	-0.75924300
C	3.33534300	-2.61935300	0.64023500
C	-2.12916900	0.26325200	-0.47438600
C	-3.17593200	0.92944900	-1.13251100
C	-2.41315900	-0.79792400	0.40128100
C	-4.49483100	0.55693900	-0.90393500
H	-2.93927900	1.72884500	-1.82519100
C	-3.72822900	-1.17743700	0.63593800
H	-1.59176400	-1.31492900	0.88371100
C	-4.75591800	-0.48992100	-0.01619800
H	-5.31388400	1.06146200	-1.40109900
H	-3.96490300	-1.99190200	1.30918500
N	-6.14102100	-0.87989600	0.23286300
O	-7.03719300	-0.24747300	-0.33441400
O	-6.35362400	-1.82284500	1.00123800
H	3.07862100	-3.53217900	1.17341600
C	3.76267500	-2.04292500	-1.71054900
C	4.69492800	-0.88888200	-1.25512200
H	3.32231800	-1.81509900	-2.68781700
H	4.33291600	-2.97223300	-1.82316600
H	4.50328000	0.00591900	-1.85412400
H	5.73597900	-1.16688400	-1.44831000

14. References

[1] J. Lindh, J. Sävmarker, P. Nilsson, P. J.R. Sjöberg, M. Larhed, *Chem. Eur. J.* **2009**, *15*, 4630–4636.

[2] S. Mukherjee, E. J. Corey, *Org. Lett.* **2010**, *12*, 632–635.

[3] a) M. Mato, B. Herlé, A. M. Echavarren, *Org. Lett.* **2018**, *20*, 4341–4345. b) M. Mato, A. M. Echavarren, *Angew. Chem. Int. Ed.* **2019**, *58*, 2088–2092; *Angew. Chem.* **2019**, *131*, 2110–2114. c) M. Mato, C. García-Morales, A. M. Echavarren, *ACS Catal.* **2020**, *10*, 3564–3570.

[4] P. Calleja, Ó. Pablo, B. Ranieri, M.Gaydou, A. Pitaval, M. Moreno, M. Raducan, A. M. Echavarren, *Chem. Eur. J.* **2016**, *22*, 13613–13618.

[5] A. Fürstner, P. Hannen, *Chem. Eur. J.* **2006**, *12*, 3006 – 3019

[6] O. A. McNamara, A. R. Maguire, *Tetrahedron* **2011**, *67*, 9–40.

[7] a) M. Buchert, H.-U. Reißig, *Liebigs Ann.* **1996**, 2007–2013; b) R. C. Conyers, B. W. Gung, *Chem. Eur. J.* **2013**, *19*, 654–664; c) R. C. Conyers, C. L. Barnes, B. W. Gung, *Tetrahedron Lett.* **2015**, *56*, 3318–3321.

[8] Gaussian 09, Revision D.01, Frisch, M. J.; Trucks, G. W.; Schlegel, H. B.; Scuseria, G. E.; Robb, M. A.; Cheeseman, J. R.; Scalmani, G.; Barone, V.; Mennucci, B.; Petersson, G. A.; Nakatsuji, H.; Caricato, M.; Li, X.; Hratchian, H. P.; Izmaylov, A. F.; Bloino, J.; Zheng, G.; Sonnenberg, J. L.; Hada, M.; Ehara, M.; Toyota, K.; Fukuda, R.; Hasegawa, J.; Ishida, M.; Nakajima, T.; Honda, Y.; Kitao, O.; Nakai, H.; Vreven, T.; Montgomery, Jr., J. A.; Peralta, J. E.; Ogliaro, F.; Bearpark, M.; Heyd, J. J.; Brothers, E.; Kudin, K. N.; Staroverov, V. N.; Keith, T.; Kobayashi, R.; Normand, J.; Raghavachari, K.; Rendell, A.; Burant, J. C.; Iyengar, S. S.; Tomasi, J.; Cossi, M.; Rega, N.; Millam, J. M.; Klene, M.; Knox, J. E.; Cross, J. B.; Bakken, V.; Adamo, C.; Jaramillo, J.; Gomperts, R.; Stratmann, R. E.; Yazyev, O.; Austin, A. J.; Cammi, R.; Pomelli, C.; Ochterski, J. W.; Martin, R. L.; Morokuma, K.; Zakrzewski, V. G.; Voth, G. A.; Salvador, P.; Dannenberg, J. J.; Dapprich, S.; Daniels, A. D.; Farkas, O.; Foresman, J. B.; Ortiz, J. V.; Cioslowski, J.; Fox, D. J. Gaussian, Inc., Wallingford CT, **2013**.

[9] (a) A. D. Becke, *J. Chem. Phys.* **1993**, *98*, 5648–5652; (b) C. Lee, W. Yang, R. G. Parr, *Phys. Rev. B.* **1988**, *37*, 785; (c) P. J. Stephens, F. J. Devlin, C. F. Chabalowsky, M. J. Frisch, *J. Phys. Chem*. **1994**, *98*, 11623–11627.

[10] (a) Hay, P. J.; Wadt, W. R. *J. Chem. Phys.*, **1985**, *82*, 299–310. (b) Wadt, W. R.; Hay, P. J. *J. Chem. Phys.*, **1985**, *82*, 284–298.

[11] (a) Ditchfield, R.; Hehre, W. J.; Pople, J. A. *J. Chem. Phys.*, **1971**, *54*, 724–728. (b) Francl, Michelle M.; Pietro, W. J.; Hehre, W. J.; Binkley, J. Stephen, G. M. S.; DeFrees, D. J.; Pople, J. A. *J. Chem. Phys*., **1982**, *77*, 3654–3665. (c) Gordon, M. S.; Binkley, J. S.; Pople, J. A.; Pietro, W. J.; Hehre, W. J. *J. Am. Chem. Soc.*, **1982**, *104*, 2797–2803. (d) Hariharan, P. C.; Pople, J. A. *Theor. Chim. Acta*, **1973**, *28*, 213–222. (e) Hehre, W. J.; Ditchfield, R.; Pople, J. A. *J. Chem. Phys*., **1972**, *56*, 2257–2261.

[12] (a) Hay, P. J.; Wadt, W. R. *J. Chem. Phys*., **1985**, *82*, 299–310. (b) Roy, Lindsay E.; Hay, P. J.; Martin, R. L *J. Chem. Theory Comput*., S186 **2008**, *4*, 1029–1031.

[13] (a) Clark, T.; Chandrasekhar, J.; Spitznagel, G. W.; Schleyer, P. V. R. *J. Comput. Chem*., **1983**, *4*, 294–301. (b) Ditchfield, R.; Hehre, W. J.; Pople, J. A. *J. Chem. Phys*., **1971**, *54*, 724–728. (c) Francl, M. M.; Pietro, W. J.; Hehre, W. J.; Binkley, J. S.; G., Mark S.; DeFrees, D. J.; Pople, J. A. *J. Chem. Phys*., **1982**, *77*, 3654–3665. (d) Gordon, M. S.; Binkley, J. S.; Pople, J. A.; Pietro, W. J.; Hehre, W. J. *J. Am. Chem. Soc*., **1982**, *104*, 2797–2803. (e) Hariharan, P. C.; Pople, J. A. *Theor. Chim. Acta*, **1973**, *28*, 213–222. (f)

Hehre, W. J.; Ditchfield, R.; Pople, J. A. *J. Chem. Phys.*, **1972**, *56*, 2257–2261 (g) Spitznagel, G. W.; Clark, T.; Schleyer, P. V. R.; Hehre, W. J. *J. Comput. Chem.*, **1987**, *8*, 1109–1116

[14] Marenich, A. V.; Cramer, C. J.; Truhlar, D. G. *J. Phys. Chem. B*, **2009**, *113*, 6378–6396.

[15] Legault, C. Y. CYLview; Universite de Sherbrooke: Sherbrooke, Canada, **2009**; http://www.cylview.org.

[16] P. Calleja, Ó. Pablo, B. Ranieri, M. Gaydou, A. Pitaval, M. Moreno, M. Raducan, A. M. Echavarren, *Chem. Eur. J.* **2016**, *22*, 13613–13618.

[17] B. Herlé, P. M. Holstein, A. M. Echavarren, *ACS Catal.* **2017**, *7*, 3668–3675.

[18] a) R. E. Moore, J. A. Pettus, J. Mistysyn, *J. Org. Chem.* **1973**, *39*, 2201–2207; b) D. Grandjean, P. Pale, J. Chuche, *Tetrahedron* **1991**, *47*, 1215–1230.
